# Primary activation of *para*-quinone methides by chiral phosphoric acid for enantioselective construction of tetraarylmethanes†

**DOI:** 10.1039/d3sc05014a

**Published:** 2023-12-02

**Authors:** Zhengyu Han, Biao Zhu, Yu Zang, Chaoshen Zhang, Xiu-Qin Dong, Hai Huang, Jianwei Sun

**Affiliations:** a Jiangsu Key Laboratory of Advanced Catalytic Materials & Technology, School of Petrochemical Engineering, Changzhou University Changzhou China huanghai@cczu.edu.cn sunjw@ust.hk; b Shenzhen Bay Laboratory Shenzhen 518132 China zhangcs@ust.hk; c Shenzhen Research Institute, HKUST No. 9 Yuexing 1st Rd Shenzhen 518057 China; d Department of Chemistry, The Hong Kong University of Science and Technology Clear Water Bay, Kowloon Hong Kong SAR China; e College of Chemistry and Molecular Sciences, Engineering Research Center of Organosilicon Compounds & Materials, Ministry of Education, Wuhan University Wuhan Hubei China

## Abstract

Demonstrated here is an asymmetric nucleophilic addition *via* primary activation of *para*-quinone methides (*p*-QMs) based on a chiral phosphoric acid catalytic system. In sharp contrast to previous CPA-based bifunctional activation processes that all required the nucleophiles to have an effective hydrogen bond donor unit (*e.g.*, OH, NH), here no such unit is required in the nucleophile. *N*-protected indole nucleophiles were successfully utilized for the synthesis of chiral tetraarylmethanes with high efficiency and enantioselectivity under mild conditions. Therefore, this protocol significantly expanded the scope of asymmetric transformations of *p*-QMs.

## Abstract 2

Demonstrated here is an asymmetric nucleophilic addition *via* primary activation of *para*-quinone methides (*p*-QMs) based on a chiral phosphoric acid catalytic system.

## Graphical abstract

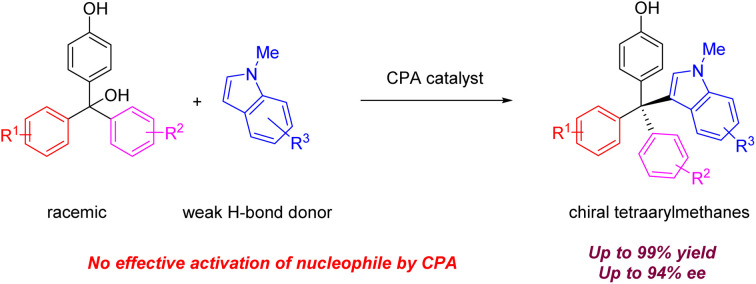

## Introduction

**Scheme 1:**
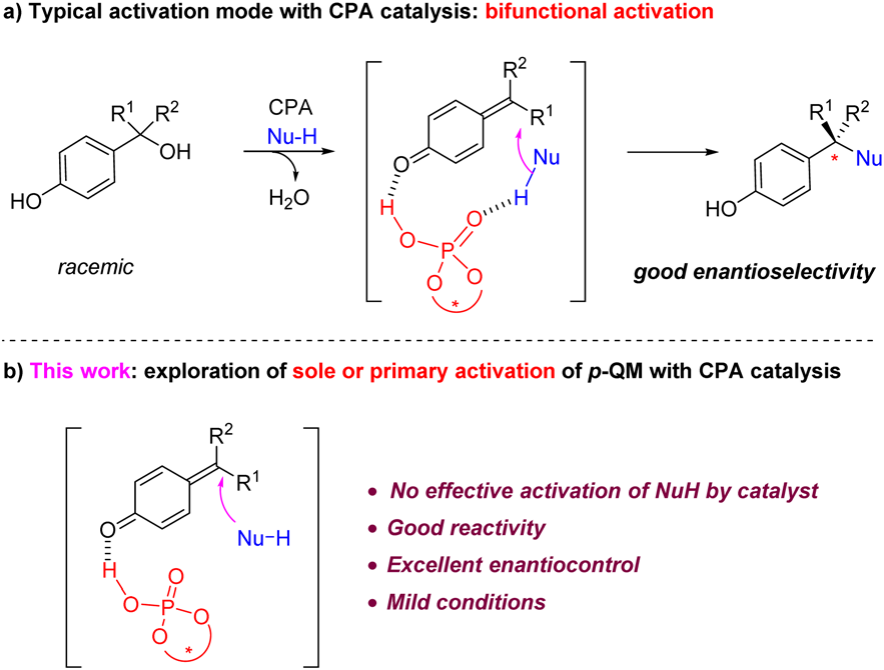
Modes of activation in CPA-catalyzed asymmetric addition to *p*-QMs.

*Para*-Quinone methides (*p*-QMs) are versatile intermediates in organic synthesis and biological processes.^1^ Recently, they have been demonstrated to be particularly powerful in asymmetric synthesis,^2^ especially for the construction of quaternary stereogenic centers.^3^ Their strong tendency for aromatization permits the catalytic asymmetric 1,6-conjugate nucleophilic addition to the remote methide position, enabling rapid construction of a range of a benzylic stereogenic centers.^4,5^ While a range of catalytic asymmetric systems have been reported for these reactions, many of them required the presynthesis of those generally unstable QMs and thus limited the utility of such reactions.^4^ In 2015, we reported the first example of using chiral phosphoric acids (CPAs) as catalysts for the asymmetric nucleophilic addition to *p*-QMs (Scheme 1a).^5*a*^ Notably, this catalytic system does not require the presynthesis of *p*-QMs, as it allows the *in situ* generation of QMs from the corresponding stable, racemic benzylic alcohols and subsequent asymmetric addition to take place in a one-pot fashion. Consequently, this protocol has been widely utilized since then.^6^

However, despite the broad utility of CPA catalysts^7^ in *p*-QM chemistry,^4–6^ it is worth noting that, due to the typically long distance between the carbonyl activation site and the nucleophilic addition site, so far all the CPA-catalyzed *p*-QM addition reactions took advantage of the bifunctional activation mode, *i.e.*, the CPA catalyst not only activates the QM carbonyl, but also has an effective hydrogen bonding interaction with the nucleophile partner (Scheme 1a).^4*h*,5,6*a*–*k*^ This hydrogen bond interaction with the nucleophile not only helps achieve a tight enantiodetermining transition state, thereby facilitating enantiocontrol, but also increases the nucleophilicity of the nucleophile and reduces the reaction barrier. However, to the best of our knowledge, an efficient example of sole or primary activation of *p*-QM but without effective activation of the nucleophile by the CPA catalyst has not been demonstrated.^8^ Herein we report a highly efficient example of this type (Scheme 1b).

Nucleophiles currently widely used CPA-catalyzed asymmetric *p*-QM addition reactions include those with an N–H functionality (*e.g.*, amines, indoles, pyrroles)^5,6*h*–*k*^ or an O–H functionality (*e.g.*, naphthols, carboxylic acids, 1,3-dicarbonyl compounds with enol tautomer).^6*a*,*b*,*d*,*e*^ These functionalities are well-known hydrogen bond donors. In many of these reactions, control experiments by protecting these free N–H or O–H functionalities normally led to either dramatically low reactivity or low enantioselectivity, which confirmed the key role of their involvement in the secondary hydrogen bond interaction.

For example, in 2020, we reported the first catalytic asymmetric synthesis of chiral tetraarylmethanes *via* conjugate addition of pyrroles to *in situ* generated *p*-QMs bearing two aryl groups at the methide position.^6*h*^ The unique three-dimensional structure together with their interesting biological activities promoted us to further develop new reactions for the synthesis of their analogues.^6*j*,9^ In particular, indoles are extremely versatile pharmacophores in drugs.^10^ However, indoles with a free N–H motif were not generally effective nucleophiles in this process. Therefore, we employed this reaction as an example to examine the possibility of sole or primary activation of *p*-QMs, which means the use of *N*-protected indoles to achieve high enantioselectivity in the absence of the secondary hydrogen bond interaction.

## Results and discussion

The initial study was carried out with racemic triarylmethanol 1a as the model substrate and *N*-methylindole 2a as the nucleophile. In the presence of 10 mol% of CPA (*R*)-A1, the desired tetraarylmethane 3aa was smoothly obtained, with encouraging conversion and enantioselectivity (56% ee). It is worth noting that the reaction of 1a with free indole (without substitution in the N−1 position) only resulted in 12% ee. It is also notable that this reaction was clean and the remaining substrate accounted for the remainder of mass balance. Encouraged by the initial results, we performed further optimization aiming to improve the enantiocontrol for this challenging situation. Various CPAs were evaluated (Table 1, entries 2–6). The steric hindrance of the 3,3′-substituents seemed to be crucial for enantiocontrol, as catalysts A1 (ref. ^11^) and A2,^12^ bearing the bulky 2,4,6-triisopropylphenyl or 2,4,6-tricyclohexylphenyl groups, respectively, could give similar enantioselectivity, but the less hindered A3 and A4 could only give moderate enantiocontrol. Along these lines, we utilized a more hindered CPA A5 bearing 2,6-diisopropyl-4-adamantylphenyl groups in the 3,3′-positions,^13^ which proved superior and gave the best enantiocontrol (80% ee, Table 1, entry 5), though with moderate conversion. Extending the reaction time did not significantly enhance the reaction conversion. Then, our attention was turned to solvent screening (Table 1, entries 6–10). Toluene proved to be the optimal choice (97% conversion, 92% ee). After systematic optimization of reaction parameters, including concentration, nucleophile equivalence, and temperature, we were able to achieve 99% conversion and 92% ee, which were determined to be the standard reaction conditions (Table 1, entry 11).

**Table 1:** Evaluation of reaction conditions^a^

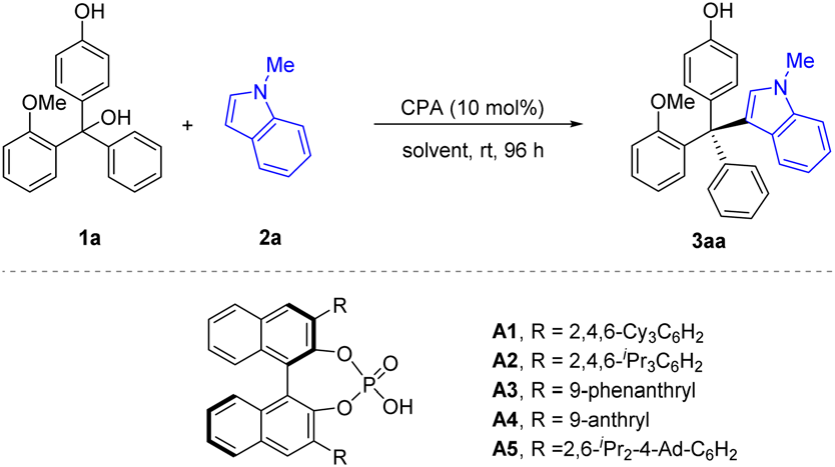				
Entry	CPA	Solvent	Conv.^b^ (%)	ee^c^ (%)
1	(*R*)-A1	DCE	48	56
2	(*R*)-A2	DCE	41	55
3	(*R*)-A3	DCE	92	16
4	(*R*)-A4	DCE	55	27
5	(*R*)-A5	DCE	58	80
6	(*R*)-A5	DCM	41	60
7	(*R*)-A5	Toluene	97	92
8	(*R*)-A5	CHCl_3_	65	91
9	(*R*)-A5	1,4-Dioxane	NR	—
10	(*R*)-A5	CH_3_CN	90	25
11^d^	(*R*)-A5	Toluene	99	92

a Reaction conditions: 1a (0.1 mmol), *N*-methylindole (0.2 mmol), CPA (10 mol%), solvent (1 mL), rt, 96 h.

b Conversion was determined by ^1^H NMR.

c The ee value was determined by chiral HPLC.

d *N*-methylindole (1.5 equiv.), toluene (0.5 mL), 72 h. NR = no reaction.

**Scheme 2:**
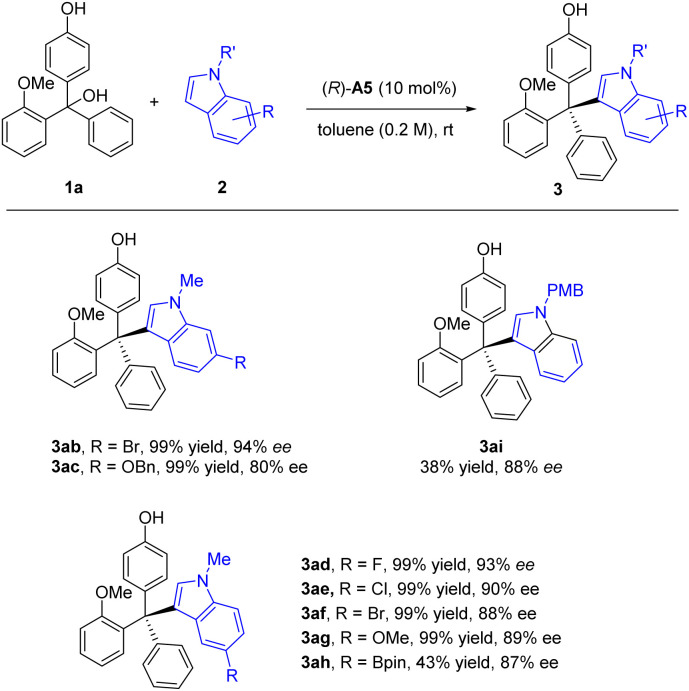
Scope of *N*-protected indoles 2. Standard conditions: 1a (0.3 mmol), *N*-protected indole 2 (0.45 mmol), CPA (10 mol%), solvent (1.5 mL), rt, 1–7 days. Isolated yield. The ee value was determined by chiral HPLC.

Next, we proceeded to examine the nucleophile scope regarding *N*-protected indoles under the standard conditions (Scheme 2). Substitution with various electron-withdrawing or electron-donating substitutes at the 5 or 6 position on the *N*-methyl indole skeleton successfully reacted to form the corresponding tetraarylmethane products in essentially quantitative yields and good to excellent enantioselectivities (80–94% ee, Scheme 2, 3ab – 3ah). In addition to the methyl group in the N−1 position, we also examined the PMB group, which maintained a high ee value, but at a reduced reaction rate.

**Scheme 3:**
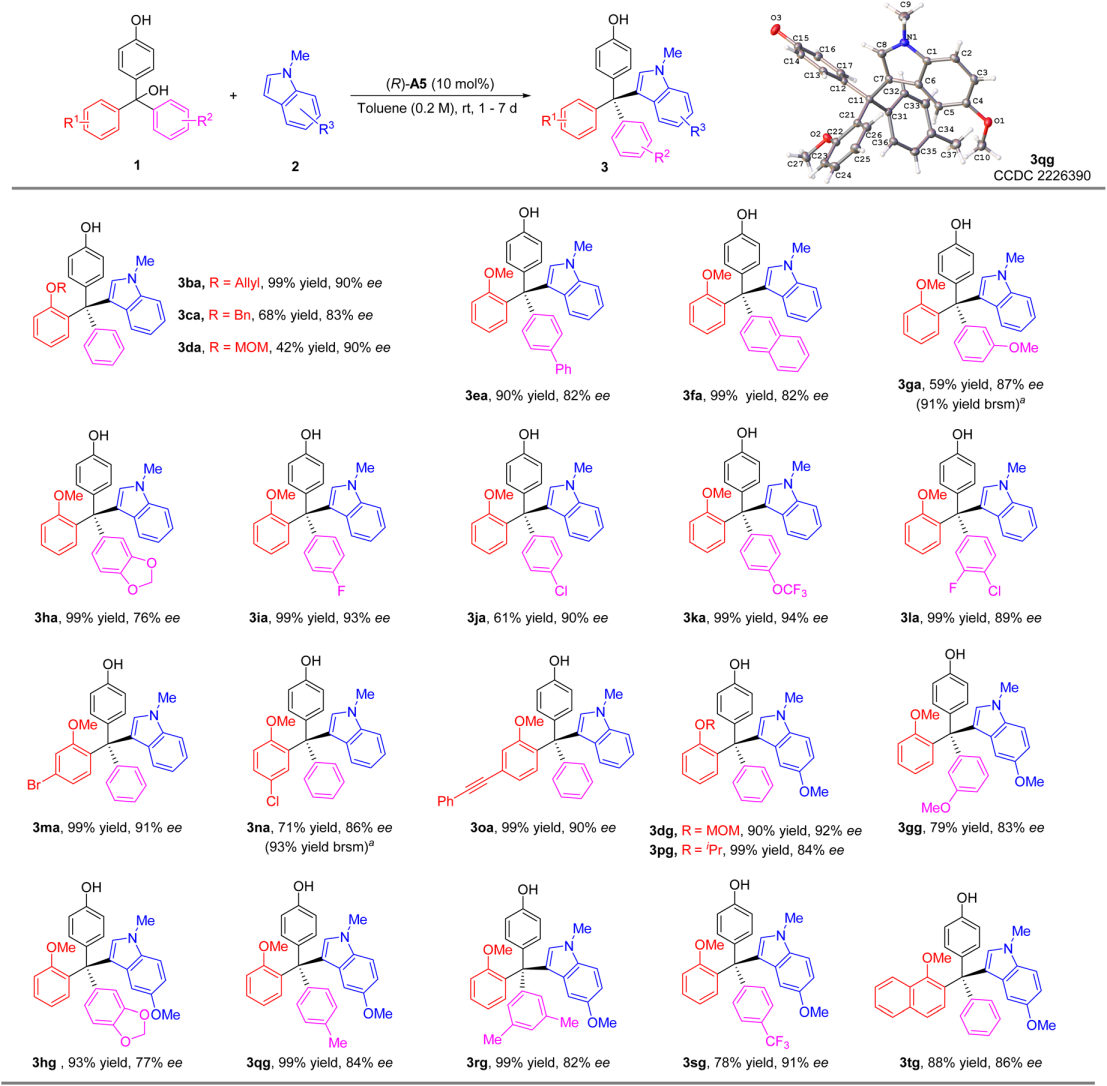
Scope of triarylmethanols 1. Standard conditions: 1a (0.3 mmol), *N*-protected indole 2 (0.45 mmol), CPA (10 mol%), solvent (1.5 mL), rt, 1–7 days. Isolated yield. The ee value was determined by chiral HPLC. ^*a*^Yield in parentheses was based on recovered starting material.

We then investigated the reaction scope with various substituted triarylmethanols (Scheme 3). Different *ortho*-alkoxyl groups, including the methoxylmethoxyl (MOMO) group, in one of the aryl groups could serve as the hydrogen bond acceptor for the differentiation between the two aryl substituents on the methide carbon of the *in situ* generated QM intermediates. The corresponding tetraarylmethane products were all obtained with high ee values (3aa–3da). Electron-donating substituents on the aryl group resulted in diminished enantioselectivity (3ea–3ha). However, electron-withdrawing groups were found to be beneficial to enantiocontrol (3ia–3la). The effect of substitutes on the 2-methoxy-substituted phenyl ring was also explored. The corresponding products 3ma–3oa could be synthesized smoothly with good enantioselectivities (86–91% ee). Moreover, 5-methoxyl-1-methyl-1*H*-indole 2g could also serve as a good nucleophile, leading to a range of substrates with diverse substituents in different positions (3dg–3tg). The low yields of 3ga and 3na were due to relatively slow conversion, but the reactions were clean themselves. Notably, in all these cases, the indole nucleophiles lack an obvious hydrogen bond donor motif, which means the CPA catalyst primarily activates the *p*-QM for the reactivity.

**Fig. 1:**
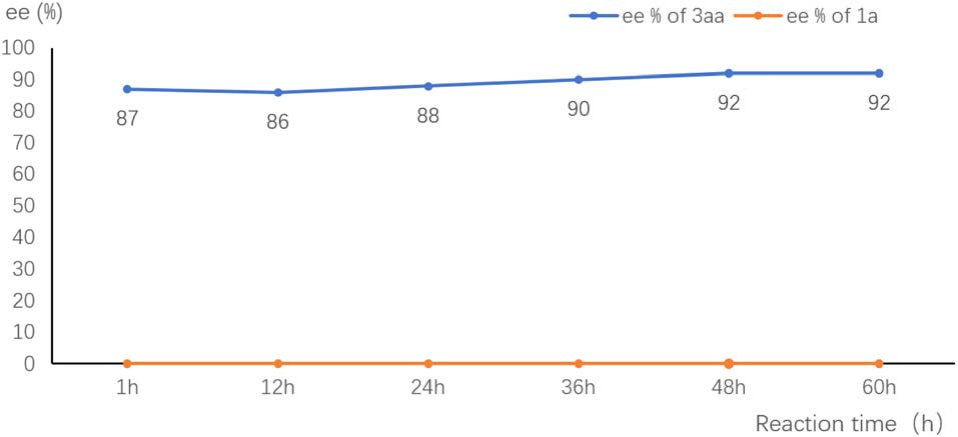
ee value–time plot of 2a and 3aa in the model reaction.

To gain insight into the mechanism, we monitored the ee value of the substrate and product in the standard reaction of 1a and 2a. The ee value of 3aa was essentially constant throughout the reaction. The remained starting material 1a was kept racemic (Fig. 1). These results are consistent with the involvement of a *p*-QM intermediate and no kinetic resolution of the substrate.

**Scheme 4:**
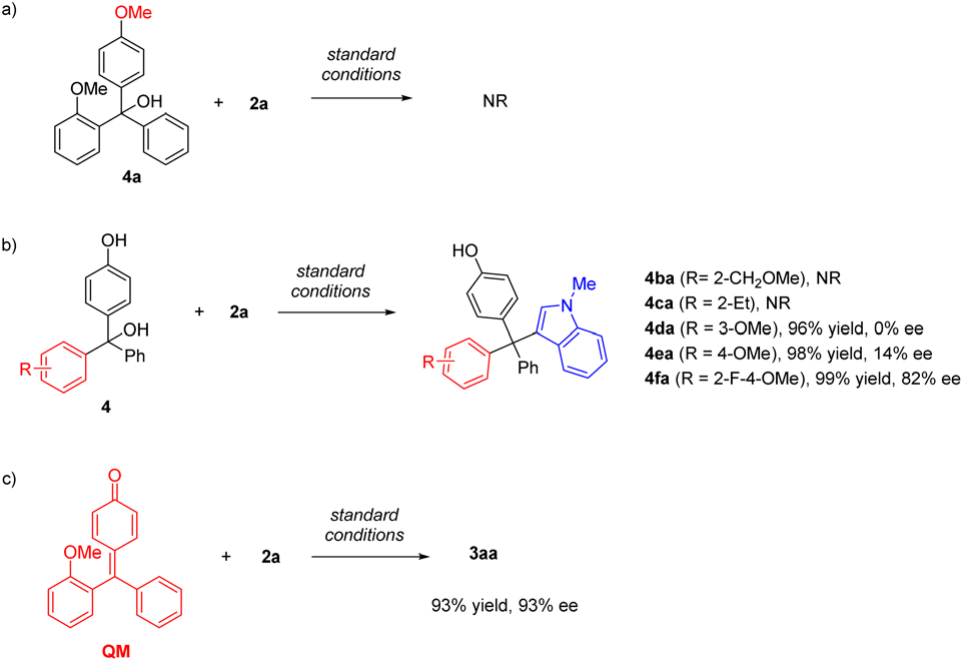
(a) Control experiment with a methyl-protected substrate. (b) Control experiments with different hydrogen bond acceptors.

To obtain more information about the key intermediate, we carried out several control experiments (Scheme 4). Firstly, upon protection of the free OH group in the substrate, the reaction of 4a was completely shut down, suggesting that *p*-QM is likely the key intermediate. The importance of the hydrogen bond acceptor at the *ortho*-position was also investigated by employing substrates 4b and 4c bearing a methoxylmethyl group and an ethyl group on the *ortho*-position, respectively. These substrates also didn't show reactivity, highlighting the role of the appropriate directing group in this catalytic system. We also evaluated the substrates 4d and 4e, in which the methoxy group was installed on the *meta* and *para* position, respectively. Although both transformations proceeded efficiently, the enantiocontrol was poor in both cases. Interestingly, the *ortho*-fluorine group could induce enantiocontrol effectively. The desired product 4fa was obtained in 99% yield and 82% ee, demonstrating the same role of fluorine as that of the alkoxyl group.

**Fig. 2:**
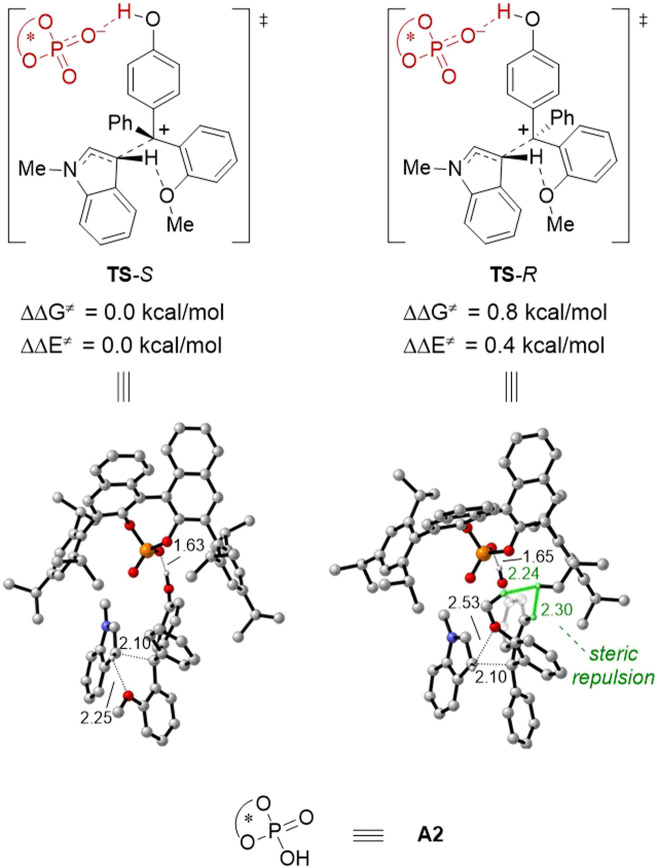
DFT-optimized enantiodetermining transition states for the reaction of 1a and 2a using A2 as a model catalyst. ΔΔ*G*^≠^ and ΔΔ*E*^≠^ are relative activation energies. The key values of the geometry structure are given in Å.

DFT studies were performed to gain more insights into the enantiodetermining transition states (Fig. 2). For the sake of computational cost, we employed (*R*)-A2 (Table 1) as the model catalyst. Based on the computed transition state structures (TS-*S* and TS-*R*), the CPA activation on the *p*-QM intermediate is so strong that almost complete protonation of the carbonyl group can be observed. In contrast, there is no effective activation of the indole nucleophile by the CPA catalyst, which is consistent with our initial proposal. This is also in contrast to previous asymmetric nucleophilic additions to *p*-QMs by CPA catalysis, where strong nucleophile-CPA hydrogen bonding was typically observed. Moreover, during C–C bond formation, the hydrogen atom in the indole 3-position has interaction with the *ortho*-OMe directing group, and this interaction is more effective in TS-*S* than that in TS-*R*. Indeed, TS-*R* suffers great steric repulsion between the methoxy, phenyl group of 1a and the CPA catalyst, but there is no such steric clash in TS-*S*. These factors are probably responsible for the higher computed energy of TS-*R* than TS-*S* (by 0.8 kcal mol^−1^), which is in qualitative agreement with the experimental outcome.

**Scheme 5:**
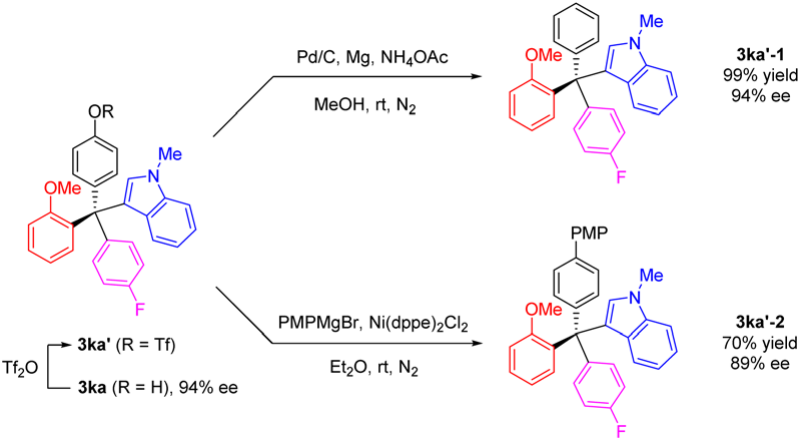
Transformations of indole-based tetraarylmethane 3ka.

To further explore the versatility of our method, we selected the chiral product 3ka as an example for derivatization. The phenolic hydroxyl group was readily transformed to a triflate group, which could be further modified into various functional groups. Under mild conditions, the triflate group of 3ka′ could be removed using Pd/C catalyzed reduction conditions, yielding a new tetraarylmethane 3ka′-1 in quantitative yield and 93% ee. Additionally, with nickel as catalyst, the triflate 3ka′ reacted with a Grignard reagent, PMPMgBr, resulting in the coupling product 3ka′-2 with a high retention of enantiomeric purity (Scheme 5).

## Conclusions

In conclusion, we have demonstrated an example of primary activation of *para*-quinone methides for their asymmetric nucleophilic addition with the CPA catalytic system. This is in sharp contrast to those previous CPA-based bifunctional activation processes that all required the nucleophiles to have an effective hydrogen bond donor unit (*e.g.*, OH, NH). In the present reaction, *N*-protected indole nucleophiles were proved to be capable of such reactions. Thus, a range of previously inaccessible tetraarylmethanes were synthesized with high efficiency and enantioselectivity under mild conditions. Therefore, this protocol also expanded the power of CPA catalysis as well as the versatility of *p*-QMs in asymmetric synthesis. Further expansion of *p*-QM chemistry by means of sole/primary activation is underway in our laboratory.

## Data availability

Experimental procedures, spectral data and DFT data can be found in the ESI.†

## Author contributions

Jianwei Sun and Zhengyu Han supervised the project and wrote the manuscript. Jianwei Sun conceived the project. Biao Zhu and Yu Zang performed condition optimization, scope study and product derivatizations. Chaoshen Zhang performed DFT studies. Xiu-Qin Dong and Hai Huang conducted scientific discussions. All the authors proofread and commented on the manuscript.

## Conflicts of interest

There are no conflicts to declare.

## Supplementary Material

SC-015-D3SC05014A-s001

SC-015-D3SC05014A-s002
