# Supplementary material for: Primary activation of para-quinone methides by chiral phosphoric acid for enantioselective construction of tetraarylmethanes
Source: Chem Sci. 2023 Dec 2;15(2):720–5. doi: 10.1039/d3sc05014a (PMC10763553; doi:10.1039/d3sc05014a)
Supplement: SC-015-D3SC05014A-s001 [file SC-015-D3SC05014A-s001.pdf]

Electronic Supplementary Material (ESI) for Chemical Science.
This journal is © The Royal Society of Chemistry 2023

Supporting Information

Primary Activation of *para*-Quinone Methides by Chiral Phosphoric Acid for Enantioselective Construction of Tetraarylmethanes

Zhengyu Han,[a] Biao Zhu,[a] Yu Zang,[a] Chaoshen Zhang,*[b,c,d] Xiu-Qin Dong,[e]
Hai Huang*[a] and Jianwei Sun*[a,c,d]

[a]*Jiangsu Key Laboratory of Advanced Catalytic Materials & Technology, School of Petrochemical Engineering, Changzhou University, Changzhou (China)*

[b]*Shenzhen Bay Laboratory, Shenzhen 518132 (China)*

[c]*Shenzhen Research Institute, HKUST, No. 9 Yuexing 1st Rd, Shenzhen 518057, China*

[d]*Department of Chemistry, The Hong Kong University of Science and Technology, Clear Water Bay, Kowloon, Hong Kong SAR (China)*

[e]*College of Chemistry and Molecular Sciences, Engineering Research Center of Organosilicon Compounds & Materials, Ministry of Education, Wuhan University, Wuhan, Hubei (China)*

Table of Contents

I. General Information S-2

II. Synthesis of Tertiary Alcohol Substrates S-3

III. Catalytic Asymmetric Synthesis of Tetraarylmethanes S-11

IV. Product Derivatizations S-38

V. Mechanistic Experiments S-41

VI. DFT Calculations S-42

VII. Determination of the Absolute Stereochemistry S-54

VIII. References S-61

NMR and HPLC Traces

I. General Information

Flash column chromatography was performed over silica gel (200-300 mesh) purchased from Qindao Puke Co., China. All air or moisture sensitive reactions were conducted in oven-dried glassware under nitrogen atmosphere using anhydrous solvents. Anhydrous dichloromethane and tetrahydrofuran were purified by the Innovative® solvent purification system. Anhydrous solvents were purchased from Sigma-Aldrich® and J&K® and used as received. ^{1}H, ^{13}C and ^{19}F spectra were collected on a Bruker AV 400 MHz or 300 MHz NMR spectrometer using residue solvent peaks as an internal standard (^{1}H NMR: $CDCl_3$ at 7.26 ppm, DMSO-d_6 at 2.5 ppm, ^{13}C NMR: $CDCl_3$ at 77.0 ppm, DMSO-d_6 at 39.5 ppm). Mass spectra were collected on an Agilent GC/MS 5975C system or an API QSTAR XL System. Optical rotations were measured on a RUDOLPH® AUTOPOL I automatic polarimeter with $[\alpha]_D$ values reported in degrees; concentration (*c*) is in 10 mg/mL. The enantiomeric excesses were determined by chiral HPLC using an Agilent 1260 LC instrument with Daicel Chiralpak AD-H, IC, AS-H or Daicel Chiralcel OD-H column. Unless otherwise noted, the racemic samples in this study were prepared using the racemic phosphoric acid catalyst 1,1′-binaphthyl-2,2′-diyl hydrogenphosphate (10 mol%).

II. Synthesis of Tertiary Alcohol Substrates

All the substrates were synthesized according to the literature procedures,[1] and the characterization data of **1a – 1d**, **1f – 1i**, **1p**, **1t**, and **4a – 4f** were consistent with those reported in the literature.[1,2]

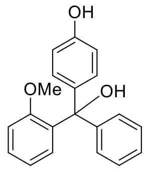

1a

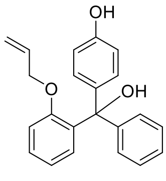

1b

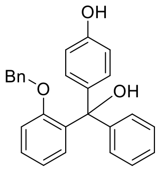

1c

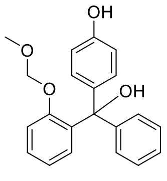

1d

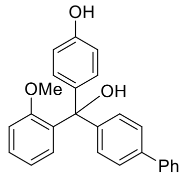

1e

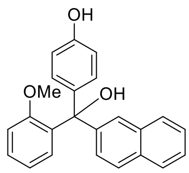

1f

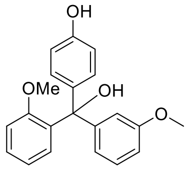

1g

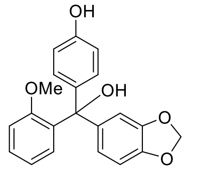

1h

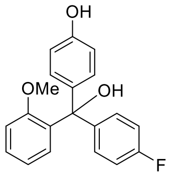

1i

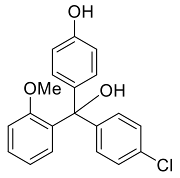

1j

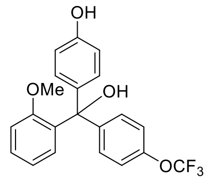

1k

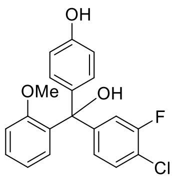

1l

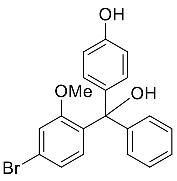

1m

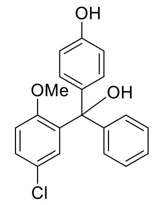

1n

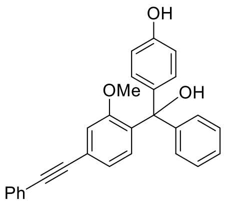

1o

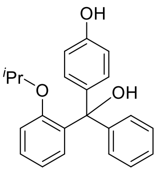

1p

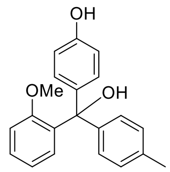

1q

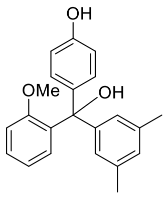

1r

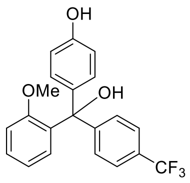

1s

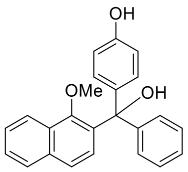

1t

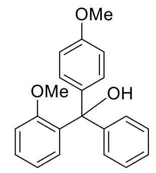

4a

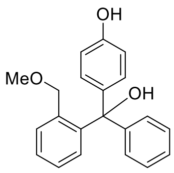

4b

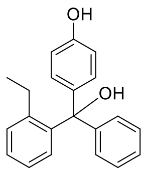

4c

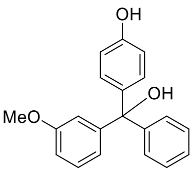

4d

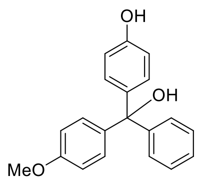

4e

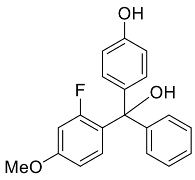

4f

General Procedure A: Preparation of Tertiary Alcohol Substrates

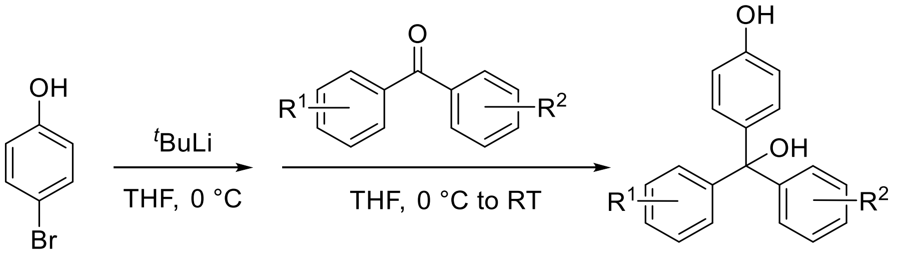

1

Under N_2, *tert*-butyllithium (1.6 M in pentane, 15.5 mL, 24.8 mmol, 8.2 equiv.) was slowly added to a stirred solution of 4-bromophenol (1.30 g, 7.5 mmol, 2.5 equiv.) in THF (50 mL) at 0 °C. The resulting mixture was stirred at 0 °C for 2 hours. Subsequently, a solution of the corresponding diaryl ketone (3.0 mmol, 1.0 equiv.) in THF (10 mL) was added and the reaction mixture was kept stirring overnight at room temperature. A saturated aqueous solution of NH_4Cl was added to quench the reaction. The reaction mixture was extracted with EtOAc (3 × 30 mL). The combined organic layers were dried over anhydrous Na_2SO_4 and filtered, then the filtrate was concentrated and the residue was purified by silica gel flash chromatography to afford the desired tertiarymethanol **1**.

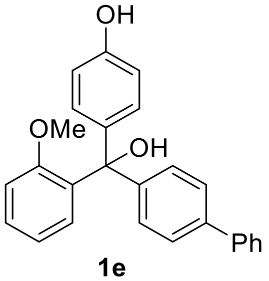

1e

4-([1,1'-Biphenyl]-4-yl(hydroxy)(2-methoxyphenyl)methyl)phenol (1e) was prepared according to the General Procedure A as yellow solid (chromatography eluent: hexanes/EtOAc = 15:1 → 10:1) in 63% yield (722 mg).

^{1}H NMR (300 MHz, $CDCl_3$) δ 7.65–7.57 (m, 2H), 7.57–7.49 (m, 2H), 7.49–7.38 (m, 2H), 7.38–7.24 (m, 4H), 7.17–7.10 (m, 2H), 6.97 (dd, J_1 = 8.2 Hz, J_2 = 1.1 Hz, 1H), 6.89–6.81 (m, 1H), 6.79–6.72 (m, 2H), 6.59 (dd, J_1 = 7.7 Hz, J_2 = 1.7 Hz, 1H), 5.39 (s, 1H), 3.69 (s, 3H) ppm.

^{13}**C NMR** (75 MHz, $CDCl_3$) δ 157.3, 154.6, 145.9, 140.8, 139.6, 138.8, 135.2, 130.2, 129.2, 129.0, 128.7, 128.2, 127.2, 127.0, 126.3, 120.5, 114.5, 112.0, 81.8, 55.8 ppm.

HRMS (ES+) Calcd for $C_{26}H_{22}O_3Na$ [M + Na]$^+$: 405.1467, Found: 405.1465.

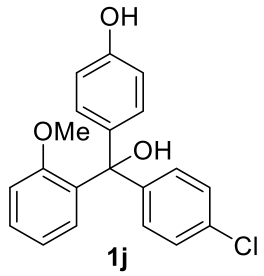

1j

4-((4-Chlorophenyl)(hydroxy)(2-methoxyphenyl)methyl)phenol (1j) was prepared according to the General Procedure A as a yellow solid (chromatography eluent: hexanes/EtOAc = 15:1 → 10:1) in 73% yield (746.0 mg).

^{1}**H NMR** (400 MHz, DMSO-d_6) δ 9.34 (s, 1H), 7.31 (d, J = 8.6 Hz, 2H), 7.29–7.26 (m, 1H), 7.15 (d, J = 8.6 Hz, 2H), 7.01 (d, J = 8.0 Hz, 1H), 6.94 (d, J = 8.7 Hz, 2H), 6.91–6.86 (m, 2H), 6.67 (d, J = 8.7 Hz, 2H), 5.68 (s, 1H), 3.49 (s, 3H) ppm.

^{13}**C NMR** (101 MHz, DMSO-d_6) δ 156.7, 156.1, 146.6, 136.5, 134.9, 131.0, 129.3, 128.9, 128.7, 128.4, 127.2, 120.0, 114.2, 112.4, 79.5, 55.3 ppm.

HRMS (ES+) Calcd for $C_{20}H_{17}ClO_3$ [M + H]$^+$: 341.0944, Found: 341.0947.

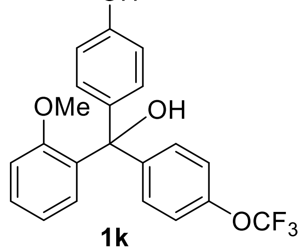

1k

4-(Hydroxy(2-methoxyphenyl)(4-(trifluoromethoxy)phenyl)methyl)phenol (1k) was prepared according to the General Procedure A as a yellow solid (chromatography eluent: hexanes/EtOAc = 15:1 → 10:1) in 66% yield (773.0 mg).

^{1}**H NMR** (300 MHz, $CDCl_3$) δ 7.34–7.22 (m, 3H), 7.15–7.08 (m, 2H), 7.06–6.99 (m, 2H),

6.95 (dd, J_1 = 8.3 Hz, J_2 = 1.1 Hz, 1H), 6.83 (td, J_1 = 7.5 Hz, J_2 = 1.1 Hz, 1H), 6.74–6.67 (m, 2H), 6.50 (dd, J_1 = 7.7 Hz, J_2 = 1.7 Hz, 1H), 3.66 (s, 3H) ppm.

^{13}C NMR (75 MHz, $CDCl_3$) δ 157.1, 154.9, 148.2, 145.4, 137.9, 134.7, 130.1, 129.2 (2C), 129.1, 120.6, 120.5 (q, J = 255.7 Hz), 119.9, 114.7, 112.0, 81.7, 55.7 ppm.

^{19}F NMR (282 MHz, $CDCl_3$) δ -57.7 ppm.

HRMS (ES+) Calcd for $C_{21}H_{17}F_3O_4Na$ [M + Na]$^+$: 413.0977, Found: 413.0973.

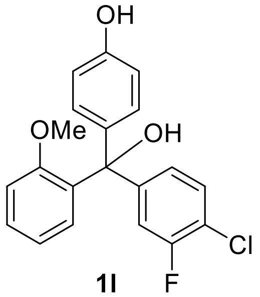

1l

4-((4-Chloro-3-fluorophenyl)(hydroxy)(2-methoxyphenyl)methyl)phenol (1l) was prepared according to the General Procedure A as a yellow solid (chromatography eluent: hexanes/EtOAc = 15:1 → 10:1) in 51% yield (550.0 mg).

^{1}H NMR (400 MHz, DMSO-d_6) δ 9.38 (s, 1H), 7.46 (t, J = 8.2 Hz, 1H), 7.32–7.28 (m, 1H), 7.11 (dd, J_1 = 1.9 Hz, J_2 = 11.2 Hz, 1H), 7.02–6.93 (m, 5H), 6.90 (t, J = 7.5 Hz, 1H), 6.68 (d, J = 8.6 Hz, 2H), 5.85 (s, 1H), 3.49 (s, 3H) ppm.

^{13}C NMR (101 MHz, DMSO-d_6) δ 156.6, 156.5 (d, J = 243.0 Hz), 156.2, 149.8 (d, J = 6.0 Hz), 135.9, 134.4, 129.3, 129.1, 128.7, 128.3, 124.7 (d, J = 3.0 Hz), 120.1, 117.1 (d, J = 17.0 Hz), 115.5 (d, J = 21.0 Hz), 114.3, 112.4, 79.2, 55.3 ppm.

^{19}F NMR (282 MHz, DMSO-d_6) δ -117.4 ppm.

HRMS (ES+): Calcd for $C_{20}H_{16}ClFO_3Na$ [M + Na]$^+$: 381.0670, Found: 381.0666.

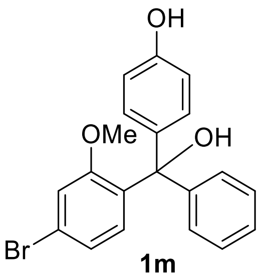

1m

4-((4-Bromo-2-methoxyphenyl)(hydroxy)(phenyl)methyl)phenol (1m) was prepared according to the General Procedure A as a yellow solid (chromatography eluent: hexanes/EtOAc = 15:1 → 10:1) in 60% yield (693.0 mg).

^{1}H NMR (400 MHz, $CDCl_3$) δ 7.33–7.17 (m, 6H), 7.03 (d, J = 8.7 Hz, 2H), 6.86 (d, J = 8.7 Hz, 1H), 6.71 (d, J = 8.7 Hz, 2H), 6.50 (d, J = 2.6 Hz, 1H), 5.23 (s, 1H), 3.62 (s, 3H) ppm.

^{13}C NMR (101 MHz, $CDCl_3$) δ 155.9, 154.9, 146.0, 137.7, 137.2, 130.1, 129.1, 128.6, 127.8, 127.5, 127.2, 125.7, 114.7, 113.3, 81.7, 56.1 ppm.

HRMS (ES+) Calcd for $C_{20}H_{18}BrO_3$ $[M + H]^+$: 385.0439, Found: 385.0436.

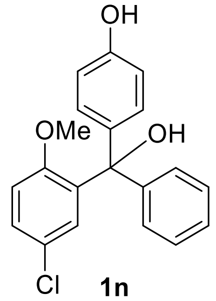

1n

4-((5-Chloro-2-methoxyphenyl)(hydroxy)(phenyl)methyl)phenol (1n) was prepared according to the General Procedure A as a yellow solid (chromatography eluent: hexanes/EtOAc = 15:1 → 10:1) in 47% yield (481.0 mg).

^{1}H NMR (400 MHz, $CDCl_3$) δ 7.33–7.16 (m, 5H), 7.06 (d, J = 1.9 Hz, 1H), 7.02–6.98 (m, 2H), 6.95 (dd, J_1 = 8.3 Hz, J_2 = 1.9 Hz, 1H), 6.74–6.65 (m, 2H), 6.38 (d, J = 8.3 Hz, 1H), 5.12 (s, 2H), 3.64 (s, 3H) ppm.

^{13}C NMR (101 MHz, $CDCl_3$) δ 157.8, 154.9, 146.1, 137.8, 134.5, 131.4, 129.1, 127.7, 127.5, 127.2, 123.4, 122.1, 115.5, 114.6, 81.7, 56.1 ppm.

HRMS (ES+) Calcd for $C_{20}H_{17}ClO_3$ $[M + H]^+$: 341.0944, Found: 341.0947.

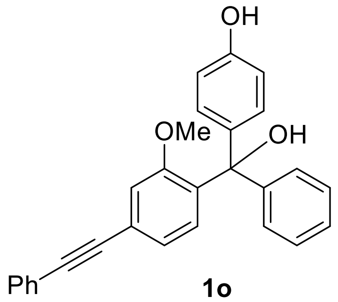

1o

4-(Hydroxy(2-methoxyphenyl)(4-(phenylethynyl)phenyl)methyl)phenol (1o) was prepared according to the General Procedure A as a yellow solid (chromatography eluent: hexanes/EtOAc = 15:1 → 10:1) in 58% yield (573.0 mg).

^{1}H NMR (400 MHz, $CDCl_3$) δ 7.54–7.51 (m, 2H), 7.38–7.19 (m, 8H), 7.10 (d, J = 1.5 Hz, 1H), 7.08–7.03 (m, 2H), 7.00 (dd, J_1 = 8.0 Hz, J_2 = 1.5 Hz, 1H), 6.72 (d, J = 8.7 Hz, 2H), 6.51 (d, J = 7.9 Hz, 1H), 5.26 (s, 1H), 3.68 (s, 3H) ppm.

^{13}C NMR (101 MHz, $CDCl_3$) δ 157.0, 154.8, 146.4, 138.2, 135.8, 131.6, 130.2, 129.2, 128.4, 128.4, 127.7, 127.6, 127.1, 123.9, 123.7, 122.9, 114.9, 114.6, 89.8, 88.8, 81.9, 55.9 ppm.

HRMS (ES+) Calcd for $C_{28}H_{22}O_3Na$ $[M + Na]^+$: 429.1467, Found: 429.1472.

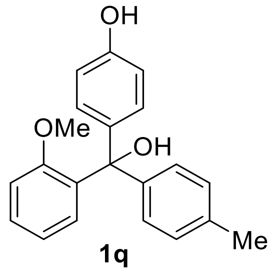

1q

4-(Hydroxy(2-methoxyphenyl)(p-tolyl)methyl)phenol (1q) was prepared according to the General Procedure A as a yellow solid (chromatography eluent: hexanes/EtOAc = 15:1 → 10:1) in 54% yield (519.0 mg).

^{1}H NMR (400 MHz, DMSO-d_6) δ 9.30 (s, 1H), 7.26 (td, J_1 = 1.8 Hz, J_2 = 7.7 Hz, 1H), 7.07–7.00 (m, 5H), 6.93 (d, J = 8.7 Hz, 2H), 6.84 (td, J_1 = 0.8 Hz, J_2 = 7.5 Hz, 1H), 6.77 (dd, J_1 = 1.8 Hz, J_2 = 7.7 Hz, 1H), 6.66 (d, J = 8.7 Hz, 2H), 5.46 (s, 1H), 3.52 (s, 3H), 2.27 (s, 3H) ppm.

^{13}C NMR (101 MHz, DMSO-d_6) δ 156.9, 155.9, 144.5, 137.3, 135.5, 135.4, 128.7, 128.7 (2

C), 127.9, 127.4, 119.9, 114.1, 112.3, 80.1, 55.5, 20.6 ppm.

HRMS (CI+) Calcd for $C_{21}H_{20}O_3$ $[M]^+$: 320.1412, Found: 320.1400.

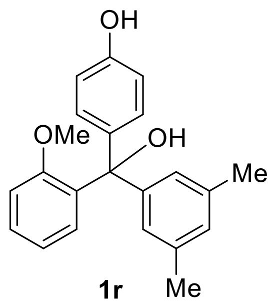

1r

4-((3,5-Dimethylphenyl)(hydroxy)(2-methoxyphenyl)methyl)phenol (1r) was prepared according to the General Procedure A as a yellow solid (chromatography eluent: hexanes/EtOAc = 15:1 → 10:1) in 70% yield (702.0 mg).

^{1}H NMR (300 MHz, DMSO-d_6) δ 9.29 (s, 1H), 7.26 (td, J_1 = 7.7 Hz, J_2 = 1.8 Hz, 1H), 7.01 (d, J = 7.2 Hz, 1H), 6.94 (d, J = 8.7 Hz, 2H), 6.88–6.80 (m, 2H), 6.79–6.70 (m, 3H), 6.66 (d, J = 8.7 Hz, 2H), 5.44 (s, 1H), 3.53 (s, 3H), 2.18 (s, 6H) ppm.

^{13}C NMR (75 MHz, DMSO-d_6) δ 157.0, 155.9, 147.4, 137.3, 136.0, 135.5, 128.8, 128.7, 128.7, 127.9, 125.3, 120.0, 114.1, 112.4, 80.2, 55.5, 21.2 ppm.

HRMS (ES+) Calcd for $C_{22}H_{23}O_3$ $[M + H]^+$: 334.1647, Found: 335.1645.

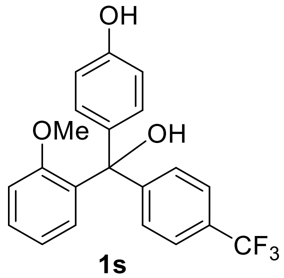

1s

4-(Hydroxy(2-methoxyphenyl)(4-(trifluoromethyl)phenyl)methyl)phenol (1s) was prepared according to the General Procedure A as a yellow solid (chromatography eluent: hexanes/EtOAc = 15:1 → 10:1) in 47% yield (528.0 mg).

^{1}H NMR (400 MHz, DMSO-d_6) δ 9.37 (s, 1H), 7.63 (d, J = 8.3 Hz, 2H), 7.38 (d, J = 8.2 Hz, 2H), 7.30 (td, J_1 = 1.4 Hz, J_2 = 7.6 Hz, 1H), 7.01–6.94 (m, 4H), 6.90 (t, J = 7.5 Hz, 1H), 6.68

(d, *J* = 8.6 Hz, 2H), 5.83 (s, 1H), 3.46 (s, 3H) ppm.

^{13}C NMR (75 MHz, DMSO-*d_6*) *δ* 156.6, 156.2, 152.3, 136.1, 134.7, 129.1, 128.8, 128.4, 128.1, 126.9 (q, *J* = 31.7 Hz), 124.5 (q, *J* = 272.0 Hz), 124.2 (q, *J* = 3.4 Hz), 120.1, 114.3, 112.4, 79.5, 55.3 ppm.

^{19}F NMR (282 MHz, DMSO-*d_6*) *δ* -60.7 ppm.

HRMS (ES+) Calcd for $C_{21}H_{17}F_3O_3Na$ $[M + Na]^+$: 397.1027, Found: 397.1018.

III. Catalytic Asymmetric Synthesis of Triarylmethanes

General Procedure B.

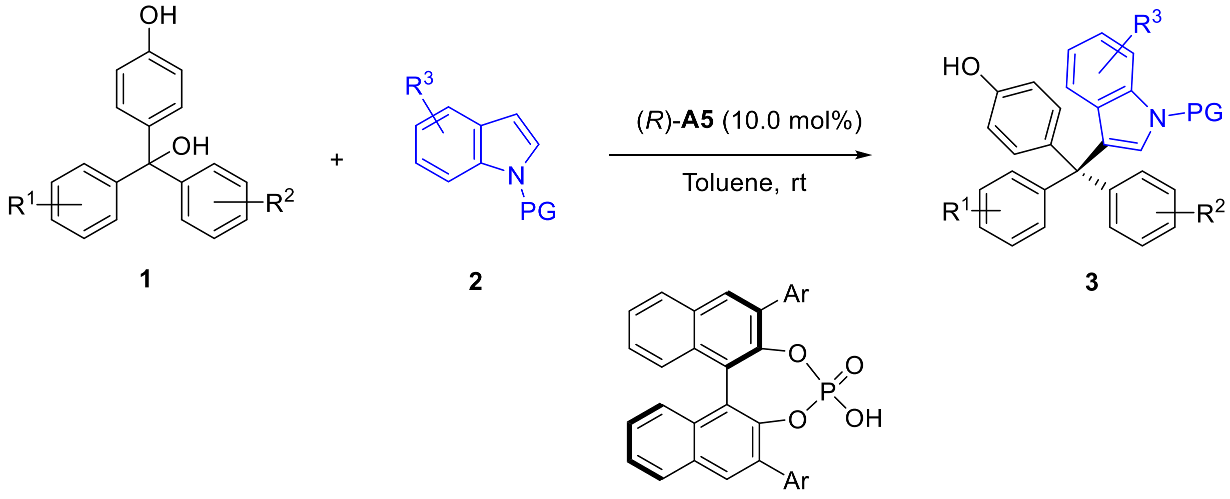

(*R*)-**A5** Ar =2,6-^{i}Pr$_2$-4-Ad-C$_6$H$_2$

At room temperature, to an oven-dried 10-mL vial charged with a solution of the tertiary alcohol **1** (0.3 mmol, 1.0 equiv) and **CPA** (*R*)-**A5** (28 mg, 0.03 mmol, 10.0 mol%) in toluene (1.5 mL), *N*-protected-indole **2** (0.45 mmol, 1.5 equiv) was added in one portion. The reaction mixture was stirred at room temperature for 1 - 7 days. Upon reaction completing, Et_3N (0.2 mL) was added and the mixture was stirred for 10 min, then concentrated under reduced pressure and further purified by silica gel flash chromatography to afford the desired product **3**.

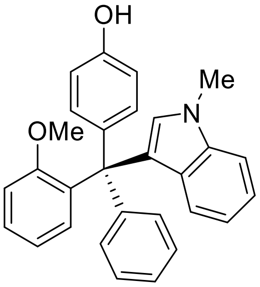

3aa

(*S*)-4-((2-Methoxyphenyl)(1-methyl-1*H*-indol-3-yl)(phenyl)methyl)phenol (3aa) was prepared according to the General Procedure B as off-white solid (chromatography eluent: hexanes/DCM = 5:2 → 5:4) in 98% yield (123.0 mg, 92% ee). The reaction time was 3 days.

$[\alpha]_D^{25}$: +2.50 (c = 1.0, $CHCl_3$). HPLC analysis of the product: Daicel CHIRALPAK IC column; 2% *i*-PrOH in hexanes, 0.4 mL/min; retention times: 39.2 min (major), 43.6 min (minor).

^{1}H NMR (400 MHz, $CDCl_3$) δ 7.32–7.06 (m, 11H), 6.92–6.81 (m, 2H), 6.76 (t, J = 1.2 Hz, 1H), 6.68–6.59 (m, 4H), 4.84 (s, 1H), 3.69 (s, 3H), 3.14 (s, 3H) ppm.

^{13}C NMR (101 MHz, $CDCl_3$) δ 158.3, 153.0, 146.1, 137.8, 137.5, 136.2, 131.5, 130.7, 130.0, 129.9, 128.2, 128.1, 126.9, 125.3, 122.4, 122.1, 120.9, 120.2, 118.5, 113.7, 113.4, 108.8, 57.3, 55.3, 32.7 ppm.

HRMS (ES+) Calcd for $C_{29}H_{25}NO_2Na$ $[M + Na]^+$: 442.1792, Found: 442.1783.

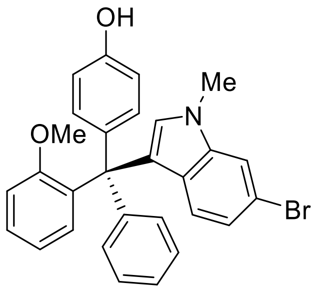

3ab

(*S*)-4-((6-Bromo-1-methyl-1*H*-indol-3-yl)(2-methoxyphenyl)(phenyl)methyl)phenol (3ab) was prepared according to the General Procedure B as yellow solid (chromatography eluent: hexanes/DCM = 5:2 → 5:4) in 99% yield (149.0 mg, 94% ee). The reaction time was 7 days.

$[\alpha]_D^{25}$: +1.98 (c = 1.0, $CHCl_3$). HPLC analysis of the product: Daicel CHIRALPAK IC column; 2% *i*-PrOH in hexanes, 0.4 mL/min; retention times: 37.2 min (major), 41.4 min (minor).

^{1}H NMR (300 MHz, $CDCl_3$) δ 7.34 (d, J = 1.8 Hz, 1H), 7.28–7.07 (m, 8H), 7.02 (d, J = 8.6 Hz, 2H), 6.89–6.73 (m, 3H), 6.65–6.52 (m, 3H), 6.46 (d, J = 8.5 Hz, 1H), 5.23 (s, 1H), 3.56 (s, 3H), 3.08 (s, 3H) ppm.

^{13}C NMR (75 MHz, $CDCl_3$) δ 158.2, 153.1, 145.8, 138.3, 137.5, 135.8, 131.4, 130.5, 130.4, 129.9, 128.3, 127.0, 125.4, 123.6, 122.3, 121.7, 120.3, 114.7, 113.8, 113.5, 111.9, 77.2, 57.1, 55.3, 32.7 ppm.

HRMS (ES+) Calcd for $C_{29}H_{24}BrNO_2Na$ [M + Na]$^+$: 520.0888, Found: 520.0887.

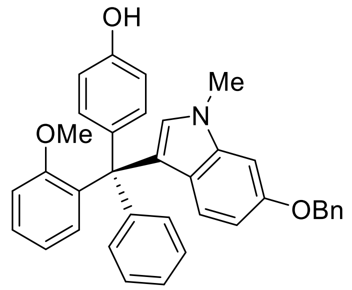

3ac

(*S*)-4-((6-(Benzyloxy)-1-methyl-1*H*-indol-3-yl)(2-methoxyphenyl)(phenyl)methyl) phenol (3ac) was prepared according to the General Procedure B as brown solid (chromatography eluent: hexanes/DCM = 5:2 → 5:4) in 99% yield (156.0 mg, 80% ee). The reaction time was 24 h.

$[\alpha]_D^{25}$: +3.24 (c = 1.0, $CHCl_3$). HPLC analysis of the product: Daicel CHIRALPAK IC column; 5% *i*-PrOH in hexanes, 1.0 mL/min; retention times: 10.9 min (major), 12.5 min (minor).

^{1}H NMR (300 MHz, $CDCl_3$) δ 7.46–7.25 (m, 6H), 7.24–7.11 (m, 6H), 7.06 (d, J = 8.7 Hz, 2H), 6.88–6.74 (m, 3H), 6.64 (d, J = 8.8 Hz, 2H), 6.53–6.42 (m, 3H), 5.04 (s, 2H), 3.61 (s, 3H), 3.12 (s, 3H) ppm.

^{13}C NMR (75 MHz, $CDCl_3$) δ 158.3, 154.9, 153.1, 146.2, 138.2, 137.8, 137.4, 136.2, 131.5, 130.7, 129.9, 129.1, 128.5, 128.2, 127.8, 127.6, 126.9, 125.3, 123.1, 122.9, 122.3, 120.3, 113.7, 113.4, 108.7, 93.6, 70.4, 57.3, 55.3, 32.7 ppm.

HRMS (ES+) Calcd for $C_{36}H_{31}NO_3Na$ [M + Na]$^+$: 548.2202, Found: 548.2210.

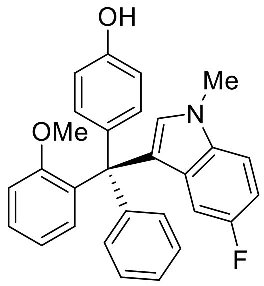

3ad

(*S*)-4-((5-Fluoro-1-methyl-1*H*-indol-3-yl)(2-methoxyphenyl)(phenyl)methyl)phenol (3ad) was prepared according to the General Procedure B as brown solid (chromatography eluent: hexanes/DCM = 5:2 → 5:4) in 99% yield (130.0 mg, 93% ee). The reaction time was 3 days.

$[\alpha]_D^{25}$: +1.76 (c = 0.5, $CHCl_3$). HPLC analysis of the product: Daicel CHIRALPAK AD-H column; 5% *i*-PrOH in hexanes, 1.0 mL/min; retention times: 14.5 min (minor), 15.4 min (major).

^{1}H NMR (300 MHz, $CDCl_3$) δ 7.33–7.27 (m, 1H), 7.23–7.09 (m, 7H), 7.05 (d, J = 8.7 Hz, 2H), 6.91–6.77 (m, 3H), 6.70–6.61 (m, 3H), 6.26 (dd, J_1 = 2.5, J_2 = 10.6 Hz, 1H), 3.69 (s, 3H), 3.13 (s, 3H) ppm.

^{13}C NMR (75 MHz, $CDCl_3$) δ 158.3, 156.9 (d, J = 231.8 Hz), 153.2, 145.8, 137.5, 135.8, 134.2, 131.5, 130.5, 129.9, 128.5 (d, J = 9.8 Hz), 128.3, 127.0, 125.4, 122.1 (d, J = 5.3 Hz), 120.3, 113.8, 113.4, 109.5 (d, J = 9.8 Hz), 109.3 (d, J = 7.5 Hz), 107.3, 106.9, 57.2, 55.3, 33.0 ppm.

^{19}F NMR (282 MHz, $CDCl_3$) δ -125.1 ppm.

HRMS (ES+) Calcd for $C_{29}H_{24}FNO_2Na$ $[M + Na]^+$: 460.1689, Found: 460.1682.

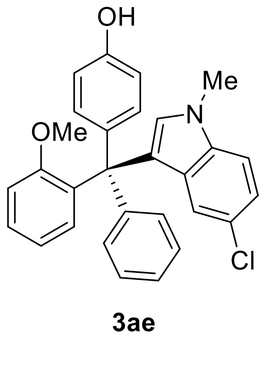

3ae

(*S*)-4-((5-Chloro-1-methyl-1*H*-indol-3-yl)(2-methoxyphenyl)(phenyl)methyl)phenol (3ae) was prepared according to the General Procedure B as orange solid (chromatography eluent: hexanes/DCM = 5:2 → 5:4) in 99% yield (136.0 mg, 90% ee). The reaction time was 7 days.

$[\alpha]_D^{25}$: +3.10 (c = 1.0, $CHCl_3$). HPLC analysis of the product: Daicel CHIRALPAK AD-H column; 5% *i*-PrOH in hexanes, 1.0 mL/min; retention times: 15.3 min (minor), 16.1 min (major).

^{1}H NMR (300 MHz, $CDCl_3$) δ 7.32–7.25 (m, 1H), 7.21–6.98 (m, 10H), 6.89–6.79 (m, 2H), 6.64 (d, J = 8.8 Hz, 2H), 6.62 (s, 1H), 6.55 (d, J = 2.0 Hz, 1H), 4.88 (s, 1H), 3.65 (s, 3H), 3.12 (s, 3H) ppm.

^{13}C NMR (75 MHz, $CDCl_3$) δ 158.2, 153.2, 145.8, 137.4, 135.9, 135.8, 131.5, 131.2, 130.5, 129.9, 129.1, 128.3, 127.0, 125.5, 124.3, 121.8, 121.5, 121.3, 120.4, 113.8, 113.5, 109.9, 57.2, 55.3, 32.9 ppm.

HRMS (ES+) Calcd for $C_{29}H_{24}ClNO_2Na$ $[M + Na]^+$: 476.1393, Found: 476.1390.

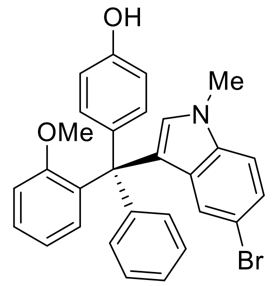

3af

(*S*)-4-((5-Bromo-1-methyl-1*H*-indol-3-yl)(2-methoxyphenyl)(phenyl)methyl)phenol (3af) was prepared according to the General Procedure B as yellow solid (chromatography eluent: hexanes/DCM = 5:2 → 5:4) in 99% yield (149.0 mg, 88% ee). The reaction time was 7 days.

$[\alpha]_D^{25}$: +10.5 (c = 0.5, $CHCl_3$). HPLC analysis of the product: Daicel CHIRALPAK AD-H column; 5% *i*-PrOH in hexanes, 1.0 mL/min; retention times: 15.7 min (minor), 16.6 min (major).

^{1}H NMR (300 MHz, $CDCl_3$) δ 7.33–7.27 (m, 1H), 7.23–6.98 (m, 10H), 6.90–6.81 (m, 2H), 6.70–6.64 (m, 2H), 6.63 (s, 1H), 6.58–6.54 (m, 1H), 4.74 (s, 1H), 3.68 (s, 3H), 3.13 (s, 3H) ppm.

^{13}**C NMR** (75 MHz, $CDCl_3$) δ 158.3, 153.2, 145.8, 137.5, 135.9, 135.8, 131.5, 131.2, 130.5, 129.9, 129.1, 128.3, 127.0, 125.5, 124.3, 121.9, 121.6, 121.4, 120.4, 113.8, 113.4, 109.9, 57.2, 55.3, 32.9 ppm.

HRMS (ES+) Calcd for $C_{29}H_{24}BrNO_2Na$ $[M + Na]^+$: 520.0888, Found: 520.0884.

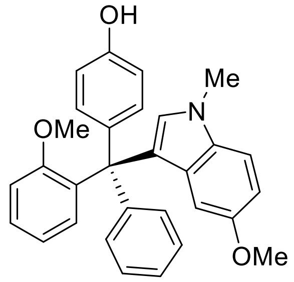

3ag

(*S*)-4-((5-Methoxy-1-methyl-1*H*-indol-3-yl)(2-methoxyphenyl) (phenyl)methyl) phenol (3ag) was prepared according to the General Procedure B as yellow solid (chromatography eluent: hexanes/DCM = 5:2 → 5:4) in 99% yield (133.0 mg, 89% ee). The reaction time was 12 h.

$[\alpha]_D^{25}$: +2.00 (c = 0.5, $CHCl_3$). HPLC analysis of the product: Daicel CHIRALPAK IC column; 5% *i*-PrOH in hexanes, 1.0 mL/min; retention times: 9.4 min (major), 10.5 min (minor).

^{1}**H NMR** (300 MHz, DMSO-d_6) δ 9.18 (s, 1H), 7.30–7.16 (m, 4H), 7.16–7.08 (m, 3H), 7.05 (dd, J_1 = 1.7, J_2 = 7.8 Hz, 1H), 6.96 (d, J = 8.1 Hz, 1H), 6.88 (d, J = 8.7 Hz, 2H), 6.82 (t, J = 7.5 Hz, 1H), 6.70–6.56 (m, 4H), 5.88 (d, J = 2.4 Hz, 1H), 3.66 (s, 3H), 3.36 (s, 3H), 3.09 (s, 3H) ppm.

^{13}**C NMR** (75 MHz, DMSO-d_6) δ 158.0, 154.8, 152.3, 145.8, 135.5, 135.3, 132.6, 130.9, 130.2, 129.8, 129.6, 128.3, 127.9, 126.8, 125.2, 120.8, 119.9, 113.7 (2C), 110.15, 110.12, 103.6, 56.8, 55.1, 54.8, 32.4 ppm.

HRMS (CI+) Calcd for $C_{30}H_{27}NO_3$ $[M]^+$:449.1991, Found: 449.1990.

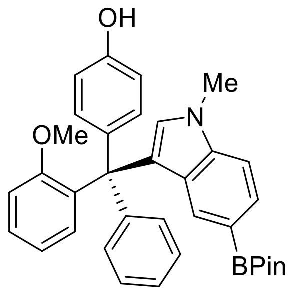

3ah

(*S*)-4-((2-Methoxyphenyl)(1-methyl-5-(4,4,5,5-tetramethyl-1,3,2-dioxaborolan-2-yl)-1*H*-indol-3-yl)(phenyl)methyl)phenol (3ah) was prepared according to the General Procedure B as yellow solid (chromatography eluent: hexanes/DCM = 5:2 → 5:4) in 43% yield (70.2 mg, 87% ee). The reaction time was 7 days.

$[\alpha]_D^{25}$: +4.32 (c = 0.5, $CHCl_3$). HPLC analysis of the product: Daicel CHIRALPAK AD-H column; 5% *i*-PrOH in hexanes, 1.0 mL/min; retention times: 10.6 min (minor), 13.1 min (major).

^{1}H NMR (300 MHz, $CDCl_3$) δ 7.51 (d, J = 8.3 Hz, 1H), 7.31–7.10 (m, 8H), 7.09–7.01 (m, 3H), 6.83 (d, J = 7.7 Hz, 2H) 6.60–6.57 (m, 3H), 3.64 (s, 3H), 3.08 (s, 3H), 1.21 (s, 12H) ppm.

^{13}C NMR (75 MHz, $CDCl_3$) δ 158.3, 153.2, 146.2, 139.5, 137.6, 136.5, 131.5, 130.8, 130.4, 130.1, 130.0, 128.0, 127.9, 127.1, 126.9, 125.2, 122.9, 120.4, 113.8, 113.3, 108.2, 83.1, 57.4, 55.3, 32.6, 24.67, 24.65 ppm.

HRMS (ES+) Calcd for $C_{35}H_{36}BNO_4Na$ $[M+Na]^+$: 568.2645, Found: 568.2645.

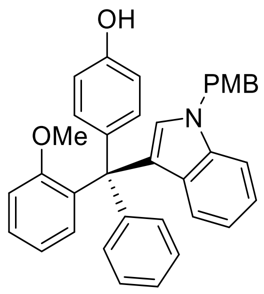

3ai

(*S*)-4-((1-(4-Methoxybenzyl)-1*H*-indol-3-yl)(2-methoxyphenyl)(phenyl)methyl) phenol (3ai) was prepared according to the General Procedure B as yellow solid

(chromatography eluent: hexanes/DCM = 5:2 → 5:4) in 38% yield (60.0 mg, 88% ee). The reaction time was 3 days.

$[\alpha]_D^{25}$: +0.72 (c = 0.5, $CHCl_3$). HPLC analysis of the product: Daicel CHIRALPAK AD-H column; 5% *i*-PrOH in hexanes, 1.0 mL/min; retention times: 21.3 min (major), 24.9 min (minor).

^{1}H NMR (300 MHz, $CDCl_3$) δ 7.30–7.12 (m, 8H), 7.08 (d, J = 8.7 Hz, 2H), 7.04–6.92 (m, 3H), 6.88–6.60 (m, 9H), 5.18 (s, 1H), 4.76 (s, 1H), 3.75 (s, 3H), 3.13 (s, 3H) ppm.

^{13}C NMR (75 MHz, $CDCl_3$) δ 158.8, 158.3, 153.1, 146.1, 137.9, 137.0, 135.9, 131.6, 130.7, 130.1, 130.0, 129.6, 128.5, 128.2, 127.5, 126.9, 125.3, 122.6, 122.5, 121.1, 120.2, 118.7, 114.0, 113.8, 113.1, 109.5, 57.4, 55.3, 55.1, 49.4 ppm.

HRMS (ES+) Calcd for $C_{36}H_{32}NO_3$ $[M + H]^+$: 526.2382, Found: 526.2373.

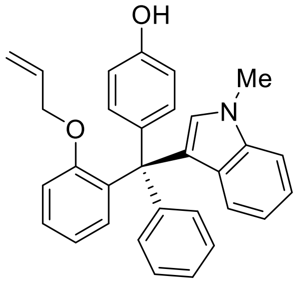

3ba

(*S*)-4-((2-(Allyloxy)phenyl)(1-methyl-1*H*-indol-3-yl)(phenyl)methyl)phenol (3ba) was prepared according to the General Procedure B as refous solid (chromatography eluent: hexanes/DCM = 5:2 → 5:4) in 99% yield (94.8 mg, 90% ee). The reaction time was 7 days.

$[\alpha]_D^{25}$: +2.34 (c = 1.0, $CHCl_3$). HPLC analysis of the product: Daicel CHIRALCEL OD-H column; 2% *i*-PrOH in hexanes, 1.0 mL/min; retention times: 26.2 min (major), 29.7 min (minor).

^{1}H NMR (300 MHz, $CDCl_3$) δ 7.24–7.05 (m, 11H), 6.83–6.58 (m, 7H), 5.37–5.25 (m, 1H), 4.87–4.72 (m, 3H), 3.92–3.83 (m, 2H), 3.63 (s, 3H) ppm.

^{13}**C NMR** (75 MHz, $CDCl_3$) δ 157.2, 153.0, 146.1, 137.8, 137.5, 135.9, 133.5, 131.7, 130.9, 130.13, 130.10, 128.2, 128.1, 126.9, 125.3, 122.5, 122.0, 120.9, 120.2, 118.5, 115.9, 113.68, 113.66, 108.8, 68.9, 57.4, 32.6 ppm.

HRMS (ES+) Calcd for $C_{31}H_{27}NO_2Na$ $[M + Na]^+$: 468.1939, Found: 468.1948.

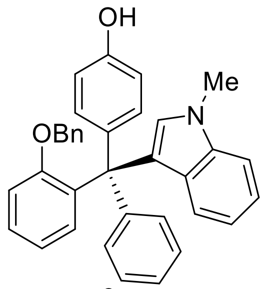

3ca

(*S*)-4-((2-(Benzyloxy)phenyl)(1-methyl-1*H*-indol-3-yl)(phenyl)methyl)phenol (3ca) was prepared according to the General Procedure B as brown solid (chromatography eluent: hexanes/DCM = 5:2 → 5:4) in 68% yield (97.0 mg, 83% ee). The reaction time was 7 days.

$[\alpha]_D^{25}$: -0.64 (c = 1.0, $CHCl_3$). HPLC analysis of the product: Daicel CHIRALCEL OD-H column; 5% *i*-PrOH in hexanes, 1.0 mL/min; retention times: 14.3 min (major), 17.0 min (minor).

^{1}**H NMR** (300 MHz, $CDCl_3$) δ 7.29–7.27 (m, 2H), 7.23–7.04 (m, 12H), 6.88–6.79 (m, 2H), 6.78–6.65 (m, 4H), 6.54–6.52 (m, 3H), 4.90 (s, 1H), 4.50 (d, J = 3.7 Hz, 2H), 3.52 (s, 3H) ppm.

^{13}**C NMR** (75 MHz, $CDCl_3$) δ 157.0, 153.0, 145.9, 137.7, 137.4, 137.2, 135.3, 131.6, 130.9, 130.2, 130.1, 128.1, 128.0, 127.8, 127.0, 126.9, 126.6, 125.2, 122.3, 121.5, 120.8, 120.1, 118.4, 113.7, 113.1, 108.8, 69.5, 57.4, 32.4 ppm.

HRMS (ES+) Calcd for $C_{35}H_{29}NO_2Na$ $[M + Na]^+$: 518.2096, Found: 518.2095.

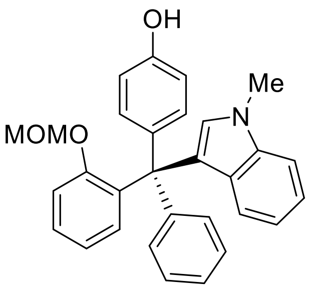

3da

(*S*)-4-((2-(Methoxymethoxy)phenyl)(1-methyl-1*H*-indol-3-yl)(phenyl)methyl) phenol (3da) was prepared according to the General Procedure B as grey solid (chromatography eluent: hexanes/DCM = 5:2 → 5:4) in 42% yield (57.0 mg, 90% ee). The reaction time was 7 days.

$[\alpha]_D^{25}$: +3.30 (c = 1.0, $CHCl_3$). HPLC analysis of the product: Daicel CHIRALPAK AD-H column; 2% *i*-PrOH in hexanes, 1.0 mL/min; retention times: 33.3 min (major), 35.7 min (minor).

^{1}H NMR (400 MHz, $CDCl_3$) δ 7.25–7.07 (m, 12H), 6.87 (t, J = 7.4, 1H), 6.74 (t, J = 7.5 Hz, 1H), δ 6.67–6.59 (m, 4H), 4.69 (s, 1H), 4.40 (s, 2H), 3.70 (s, 3H), 2.97 (s, 3H) ppm.

^{13}C NMR (101 MHz, $CDCl_3$) δ 156.2, 153.1, 146.1, 137.5, 135.9, 131.6, 130.8, 130.03, 129.98, 128.3, 128.1, 127.0, 125.4, 122.4, 122.2, 121.2, 121.0, 118.6, 115.2, 113.7, 108.8, 94.1, 57.4, 55.3, 32.7, 29.7 ppm.

HRMS (ES+) Calcd for $C_{30}H_{27}NO_3Na$ $[M + Na]^+$: 472.1889, Found: 472.1887.

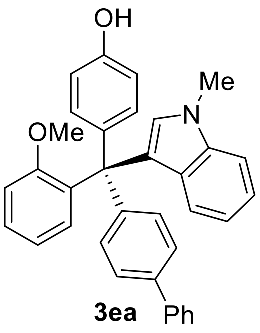

3ea

(*S*)-4-([1,1'-Biphenyl]-4-yl(2-methoxyphenyl)(1-methyl-1*H*-indol-3-yl)methyl) phenol (3ea) was prepared according to the General Procedure B as yellow solid

(chromatography eluent: hexanes/DCM = 5:2 → 5:4) in 90% yield (134.0 mg, 82% ee). The reaction time was 5 days.

$[\alpha]_D^{25}$: +10.08 (c = 0.5, $CHCl_3$). HPLC analysis of the product: Daicel CHIRALPAK IC column; 2% *i*-PrOH in hexanes, 1.0 mL/min; retention times: 19.2 min (major), 21.5 min (minor).

^{1}H NMR (400 MHz, $CDCl_3$) δ 7.61 – 7.59 (m, 2H), 7.46 (d, J = 8.3 Hz, 2H), 7.41 (t, J = 7.7 Hz, 2H), 7.34–7.27 (m, 5H), 7.24 (d, J = 1.6 Hz, 1H), 7.15–7.06 (m, 3H), 6.89 (dd, J_1 = 8.2 Hz, J_2 = 1.2 Hz, 1H), 6.85 (td, J = 7.6, 1.2 Hz, 1H), 6.76 (t, J = 7.5 Hz, 1H), 6.66 (dd, J_1 = 8.5 Hz, J_2 = 3.5 Hz, 3H), 6.63 (s, 1H), 4.69 (s, 1H), 3.70 (s, 3H), 3.15 (s, 3H) ppm.

^{13}C NMR (101 MHz, $CDCl_3$) δ 158.3, 153.1, 145.4, 140.8, 137.72, 137.66, 137.5, 136.1, 131.5, 130.7, 130.4, 130.0, 128.6, 128.2, 128.1, 126.8, 126.9, 125.4, 122.5, 122.0, 120.9, 120.3, 118.5, 113.8, 113.4, 108.8, 57.1, 55.3, 32.7 ppm.

HRMS (ES+) Calcd for $C_{35}H_{29}NO_2Na$ $[M + Na]^+$: 518.2096, Found: 518.2106.

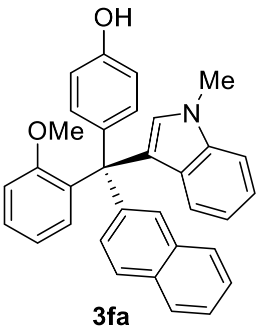

3fa

(*S*)-4-((2-Methoxyphenyl)(1-methyl-1*H*-indol-3-yl)(naphthalen-2-yl)methyl)phenol (3fa) was prepared according to the General Procedure B as light yellow solid (chromatography eluent: hexanes/DCM = 5:2 → 5:4) in 99% yield (139.0 mg, 82% ee). The reaction time was 7 days.

$[\alpha]_D^{25}$: +5.9 (c = 1.0, $CHCl_3$). HPLC analysis of the product: Daicel CHIRALPAK IC column; 2% *i*-PrOH in hexanes, 0.4 mL/min; retention times: 55.3 min (major), 62.4 min (minor).

^{1}H NMR (300 MHz, $CDCl_3$) δ 7.73–7.66 (m, 3H), 7.62 (d, J = 8.7 Hz, 1H), 7.40–7.34 (m, 2H), 7.30– 7.25 (m, 2H), 7.20–7.18 (m, 2H), 7.11–7.09 (m, 2H), 7.06–7.02 (m, 1H), 6.85–6.81 (m, 2H), 6.71–6.59 (m, 5H), 4.74 (s, 1H), 3.70 (s, 3H), 3.09 (s, 3H) ppm.

^{13}C NMR (75 MHz, $CDCl_3$) δ 158.4, 153.1, 143.6, 137.8, 137.5, 135.8, 132.9, 131.7 (2C), 130.8, 130.0, 129.5, 128.4, 128.3, 128.1, 127.7, 127.2, 125.9, 125.31, 125.27, 122.4, 121.8, 120.9, 120.3, 118.6, 113.8, 113.4, 108.8, 57.5, 55.3, 32.7 ppm.

HRMS (ES+) Calcd for $C_{33}H_{27}NO_2Na$ $[M + Na]^+$: 492.1939, Found: 492.1944.

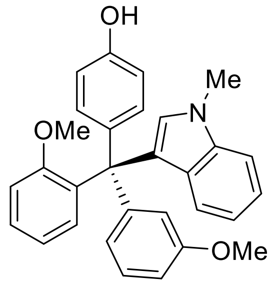

3ga

(*R*)-4-((2-Methoxyphenyl)(3-methoxyphenyl)(1-methyl-1*H*-indol-3-yl)methyl) phenol (3ga) was prepared according to the General Procedure B as grey solid (chromatography eluent: hexanes/DCM = 5:2 → 5:4) in 60% yield (81.0 mg, 87% ee). The reaction time was 7 days.

$[\alpha]_D^{25}$: +9.6 (c = 0.5, $CHCl_3$). HPLC analysis of the product: Daicel CHIRALPAK AD-H column; 5% *i*-PrOH in hexanes, 1.0 mL/min; retention times: 15.2 min (major), 17.6 min (minor).

^{1}H NMR (400 MHz, $CDCl_3$) δ 7.24–7.18 (m, 3H), 7.11–7.04 (m, 4H), 6.86–6.78 (m, 4H), 6.75–6.68 (m, 2H), 6.64–6.59 (m, 4H), 4.87 (s, 1H), 3.66 (s, 3H), 3.65 (s, 3H), 3.13 (s, 3H) ppm.

^{13}C NMR (101 MHz, $CDCl_3$) δ 158.6, 158.4, 153.1, 148.1, 137.6, 137.5, 136.1, 131.5, 130.7, 130.0, 128.2, 127.6, 123.0, 122.5, 121.9, 120.9, 120.3, 118.5, 116.6, 113.7 (2C), 113.4, 110.0, 108.8, 57.4, 55.3, 55.1, 32.7 ppm.

HRMS (CI+) Calcd for $C_{30}H_{27}NO_3$ $[M]^+$:449.1991, Found: 449.1990.

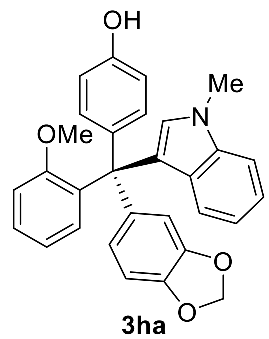

3ha

(*R*)-4-(Benzo[*d*][1,3]dioxol-5-yl(2-methoxyphenyl)(1-methyl-1*H*-indol-3-yl)methyl) phenol (3ha) was prepared according to the General Procedure B as grey solid (chromatography eluent: hexanes/DCM = 5:2 → 5:4) in 99% yield (137.0 mg, 76% ee). The reaction time was 7 days.

$[\alpha]_D^{25}$: +4.5 (c = 0.5, $CHCl_3$). HPLC analysis of the product: Daicel CHIRALPAK IC column; 5% *i*-PrOH in hexanes, 1.0 mL/min; retention times: 10.5 min (major), 12.3 min (minor).

^{1}H NMR (400 MHz, $CDCl_3$) δ 7.25–7.18 (m, 3H), 7.08–7.04 (m, 3H), 6.86–6.59 (m, 10H), 5.87 (dd, J_1 = 7.4 Hz, J_2 = 1.4 Hz, 2H), 4.87 (s, 1H), 3.67 (s, 3H), 3.17 (s, 3H) ppm.

^{13}C NMR (101 MHz, $CDCl_3$) δ 158.3, 153.1, 146.5, 144.9, 140.2, 138.1, 137.5, 136.1, 131.3, 130.6, 129.9, 128.2, 128.1, 123.2, 122.5, 122.1, 120.9, 120.2, 118.5, 113.7, 113.4, 111.1, 108.8, 106.6, 100.6, 57.1, 55.4, 32.7 ppm.

HRMS (ES+) Calcd for $C_{30}H_{26}NO_4$ $[M + H]^+$: 463.1784, Found: 463.1782.

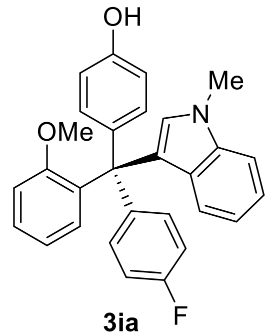

3ia

(*R*)-4-((4-Fluorophenyl)(2-methoxyphenyl)(1-methyl-1*H*-indol-3-yl)methyl)phenol (3ia) was prepared according to the General Procedure B as brown solid

(chromatography eluent: hexanes/DCM = 5:2 → 5:4) in 99% yield (131.0 mg, 93% ee). The reaction time was 2 days.

$[\alpha]_D^{25}$: +6.27 (c = 0.5, $CHCl_3$). HPLC analysis of the product: Daicel CHIRALPAK AD-H column; 2% *i*-PrOH in hexanes, 1.0 mL/min; retention times: 40.3 min (minor), 46.5 min (major).

^{1}H NMR (300 MHz, $CDCl_3$) δ 7.30 – 7.20 (m, 2H), 7.18–7.11 (m, 2H), 7.11–6.98 (m, 3H), 6.91–6.68 (m, 5H), 6.65–6.54 (m, 4H), 5.03 (s, 1H), 3.64 (s, 3H), 3.13 (s, 3H) ppm.

^{13}C NMR (75 MHz, $CDCl_3$) δ 160.7 (d, J = 242.3 Hz), 158.1, 153.1, 141.7 (d, J = 3.3 Hz), 137.7, 137.5, 135.9, 131.4 (d, J = 7.5 Hz), 131.3, 130.5, 129.9, 128.3, 128.0, 122.3, 122.0, 121.0, 120.3, 118.6, 113.8, 113.5 (d, J = 20.3 Hz), 113.3, 108.9, 56.8, 55.2, 32.6 ppm.

^{19}F NMR (282 MHz, $CDCl_3$) δ -118.4 ppm.

HRMS (ES+) Calcd for $C_{29}H_{24}FNO_2Na$ $[M + Na]^+$: 460.1689, Found: 460.1682.

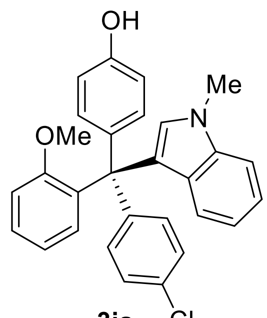

3ja

(*R*)-4-((4-Chlorophenyl)(2-methoxyphenyl)(1-methyl-1*H*-indol-3-yl)methyl)phenol (3ja) was prepared according to the General Procedure B as grey solid (chromatography eluent: hexanes/DCM = 5:2 → 5:4) in 61% yield (84.0 mg, 90% ee). The reaction time was 5 days.

$[\alpha]_D^{25}$: +4.5 (c = 0.5, $CHCl_3$). HPLC analysis of the product: Daicel CHIRALPAK IC column; 1.5% *i*-PrOH in hexanes, 0.5 mL/min; retention times: 29.7 min (major), 33.9 min (minor).

^{1}**H NMR** (300 MHz, $CDCl_3$) δ 7.29 (dd, J_1 = 1.3, J_2 = 7.7 Hz, 1H), 7.22–7.17 (m, 2H), 7.15 (s, 4H), 7.09 (t, J = 7.6 Hz, 1H), 7.03 (d, J = 8.7 Hz, 2H), 6.88–6.79 (m, 2H), 6.79–6.71 (m, 1H), 6.68–6.62 (m, 3H), 6.57 (s, 1H), 4.85 (s, 1H), 3.70 (s, 3H), 3.19 (s, 3H) ppm.

^{13}**C NMR** (75 MHz, $CDCl_3$) δ 158.1, 153.2, 144.7, 137.6, 137.4, 135.5, 131.37, 131.35, 130.9, 130.6, 129.9, 128.4, 127.9, 127.0, 122.3, 121.8, 121.1, 120.3, 118.7, 113.8, 113.2, 108.9, 56.9, 55.2, 32.7 ppm.

HRMS (ES+) Calcd for $C_{29}H_{24}ClNO_2Na$ $[M + Na]^+$: 476.1393, Found: 476.1387.

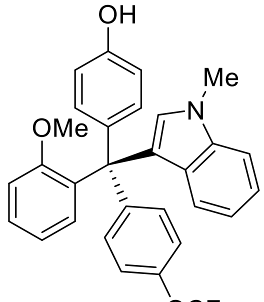

3ka

(*R*)-4-((2-Methoxyphenyl)(1-methyl-1*H*-indol-3-yl)(4-(trifluoromethoxy)phenyl) methyl)phenol (3ka) was prepared according to the General Procedure B as yellow solid (chromatography eluent: hexanes/DCM = 5:2 → 5:4) in 99% yield (151.0 mg, 94% ee). The reaction time was 2 days.

$[\alpha]_D^{25}$: +6.36 (c = 1.0, $CHCl_3$). HPLC analysis of the product: Daicel CHIRALPAK AD-H column; 2% *i*-PrOH in hexanes, 1.0 mL/min; retention times: 24.0 min (major), 26.2 min (minor).

^{1}**H NMR** (300 MHz, $CDCl_3$) δ 7.27-7.15 (m, 6H), 7.13–6.97 (m, 5H), 6.89–6.70 (m, 3H), 6.67–6.56 (m, 3H), 6.55 (s, 1H), 4.87 (s, 1H), 3.68 (s, 3H), 3.14 (s, 3H) ppm.

^{13}**C NMR** (75 MHz, $CDCl_3$) δ 158.0, 153.2, 146.8 (q, J = 1.5 Hz), 145.0, 137.6, 137.3, 135.7, 131.4, 131.1, 130.6, 129.9, 128.4, 127.9, 122.3, 121.7, 121.1, 120.5 (q, J = 255.0 Hz), 120.3, 119.2, 118.7, 113.9, 113.3, 108.9, 56.9, 55.1, 32.7 ppm.

^{19}**F NMR** (282 MHz, $CDCl_3$) δ -57.7 ppm.

HRMS (ES+) Calcd for $C_{30}H_{25}F_3NO_3$ $[M + H]^+$: 504.1787, Found: 504.1784.

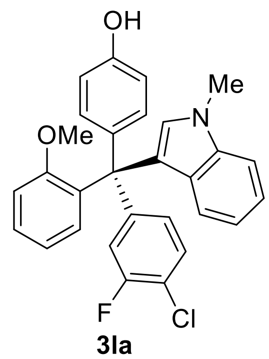

3la

(*R*)-4-((4-Chloro-3-fluorophenyl)(2-methoxyphenyl)(1-methyl-1*H*-indol-3-yl) methyl)phenol (3la) was prepared according to the General Procedure B as brown solid (chromatography eluent: hexanes/DCM = 5:2 → 5:4) in 99% yield (141.0 mg, 89% ee). The reaction time was 5 days.

$[\alpha]_D^{25}$: +6.38 (c = 1.0, $CHCl_3$). HPLC analysis of the product: Daicel CHIRALPAK AD-H column; 5% *i*-PrOH in hexanes, 1.0 mL/min; retention times: 11.2 min (minor), 12.4 min (major).

^{1}H NMR (300 MHz, $CDCl_3$) δ 7.34–7.27 (m, 1H), 7.25–7.00 (m, 7H), 6.96 (dd, J_1 = 1.8, J_2 = 8.7 Hz, 1H), 6.91–6.74 (m, 3H), 6.65 (t, J = 8.3 Hz, 3H), 6.55 (s, 1H), 4.87 (s, 1H), 3.71 (s, 3H), 3.23 (s, 3H) ppm.

^{13}C NMR (75 MHz, $CDCl_3$) δ 157.9, 157.2 (d, J = 243.8 Hz), 153.3, 147.6 (d, J = 6.0 Hz), 137.6, 136.8, 134.8, 131.3, 130.6, 129.8, 128.6 (d, J = 2.3 Hz), 127.8, 126.5 (d, J = 3.8 Hz), 122.2, 121.3, 121.2, 120.3, 118.8, 118.3, 118.0, 117.4 (d, J = 18.0 Hz), 113.9, 113.0, 109.0, 57.0, 55.1, 32.7 ppm.

^{19}F NMR (282 MHz, $CDCl_3$) δ -117.2 ppm.

HRMS (ES+) Calcd for $C_{29}H_{24}ClFNO_2$ $[M + H]^+$: 472.1480, Found: 472.1474.

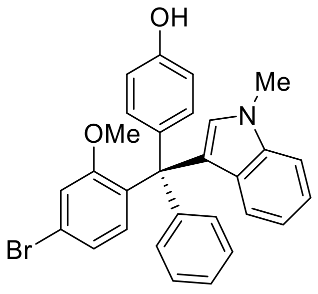

3ma

(*S*)-4-((4-Bromo-2-methoxyphenyl)(1-methyl-1*H*-indol-3-yl)(phenyl)methyl)phenol (3ma) was prepared according to the General Procedure B as brown solid (chromatography eluent: hexanes/DCM = 5:2 → 5:4) in 99% yield (148.0 mg, 91% ee). The reaction time was 5 days.

$[\alpha]_D^{25}$: -2.60 (c = 0.5, $CHCl_3$). HPLC analysis of the product: Daicel CHIRALPAK AD-H column; 5% *i*-PrOH in hexanes, 1.0 mL/min; retention times: 11.9 min (major), 12.7 min (minor).

^{1}H NMR (300 MHz, $CDCl_3$) δ 7.24–7.14 (m, 8H), 7.13–7.02 (m, 3H), 6.82–6.73 (m, 2H), 6.68–6.59 (m, 3H), 6.56 (s, 1H), 4.79 (s, 1H), 3.68 (s, 3H), 3.09 (s, 3H) ppm.

^{13}C NMR (75 MHz, $CDCl_3$) δ 157.0, 153.2, 145.5, 138.2, 137.5, 137.2, 131.4, 130.6, 130.0, 129.9, 127.9, 127.8, 127.0, 125.5, 125.4, 122.1, 121.2, 121.1, 118.7, 114.6, 113.9, 108.9, 57.3, 55.6, 32.7 ppm.

HRMS (ES+) Calcd for $C_{29}H_{25}BrNO_2$ $[M + H]^+$: 498.1069, Found: 498.1063.

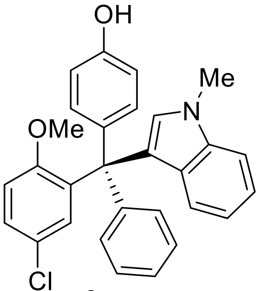

3na

(*S*)-4-((5-Chloro-2-methoxyphenyl)(1-methyl-1*H*-indol-3-yl)(phenyl)methyl)phenol (3na) was prepared according to the General Procedure B as white solid

(chromatography eluent: hexanes/DCM = 5:2 → 5:4) in 60% yield (81.0 mg, 86% ee). The reaction time was 5 days.

$[\alpha]_D^{25}$: +2.84 (c = 1.0, $CHCl_3$). HPLC analysis of the product: Daicel CHIRALPAK AD-H column; 5% *i*-PrOH in hexanes, 1.0 mL/min; retention times: 14.5 min (major), 16.4 min (minor).

^{1}H NMR (300 MHz, $CDCl_3$) δ 7.22–6.93 (m, 12H), 6.77 (t, J = 9.0 Hz, 1H), 6.69–6.55 (m, 4H), 4.89 (s, 1H), 3.66 (s, 3H), 3.13 (s, 3H) ppm.

^{13}C NMR (75 MHz, $CDCl_3$) δ 158.9, 153.1, 145.5, 137.5, 137.2, 135.2, 132.0, 131.4, 129.9, 129.8, 127.9, 127.0, 125.5, 123.2, 122.2, 121.7, 121.3, 121.1, 118.7, 116.5, 113.8, 108.9, 57.1, 55.5, 32.7 ppm.

HRMS (ES+) Calcd for $C_{29}H_{24}ClNO_2Na$ $[M + Na]^+$: 476.1393, Found: 476.1385.

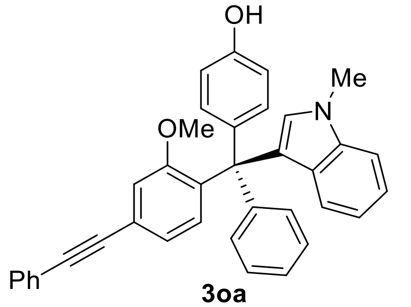

3oa

(*S*)-4-((2-Methoxy-4-(phenylethynyl)phenyl)(1-methyl-1*H*-indol-3-yl)(phenyl) methyl)phenol (3oa) was prepared according to the General Procedure B as yellow solid (chromatography eluent: hexanes/DCM = 5:2 → 5:4) in 99% yield (156.0 mg, 90% ee). The reaction time was 5 days.

$[\alpha]_D^{25}$: +3.30 (c = 1.0, $CHCl_3$). HPLC analysis of the product: Daicel CHIRALPAK AD-H column; 3% *i*-PrOH in hexanes, 1.0 mL/min; retention times: 44.3 min (minor), 49.1 min (major).

^{1}H NMR (300 MHz, $CDCl_3$) δ 7.53–7.50 (m, 2H), 7.39–7.31 (m, 3H), 7.25–6.99 (m, 12H), 6.77 (t, J = 7.5 Hz, 1H), 6.70–6.61 (m, 3H), 6.58 (s, 1H), 4.77 (s, 1H), 3.68 (s, 3H), 3.16 (s, 3H) ppm.

^{13}**C NMR** (75 MHz, $CDCl_3$) δ 158.0, 153.2, 145.7, 137.5, 137.4, 136.9, 131.6, 131.5, 130.8, 130.0, 129.9, 128.3, 128.2, 128.0, 127.0, 125.4, 123.8, 123.2, 122.8, 122.3, 121.8, 121.0, 118.6, 116.1, 113.8, 108.9, 89.30, 89.27, 57.4, 55.4, 32.7 ppm.

HRMS (ES+) Calcd for $C_{37}H_{29}NO_2Na$ $[M + Na]^+$: 542.2096, Found: 542.2100.

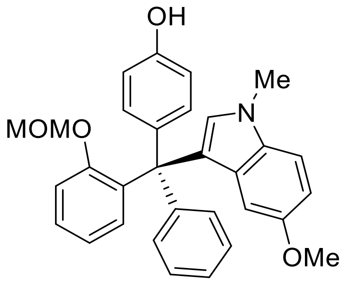

3dg

(*S*)-4-((5-Methoxy-1-methyl-1*H*-indol-3-yl)(2-(methoxymethoxy)phenyl)(phenyl) methyl)phenol (3dg) was prepared according to the General Procedure B as pink solid (chromatography eluent: hexanes/DCM = 5:2 → 5:4) in 90% yield (129.0 mg, 92% ee). The reaction time was 3 days.

$[\alpha]_D^{25}$: +6.44 (c = 1.0, $CHCl_3$). HPLC analysis of the product: Daicel CHIRALPAK AD-H column; 5% *i*-PrOH in hexanes, 1.0 mL/min; retention times: 13.8 min (major), 17.2 min (minor).

^{1}**H NMR** (300 MHz, $CDCl_3$) δ 7.29–7.06 (m, 11H), 6.93–6.84 (m, 1H), 6.74 (dd, J_1 = 2.5, J_2 = 8.8 Hz, 1H), 6.65 (d, J = 8.8 Hz, 2H), 6.58 (s, 1H), 6.03 (d, J = 2.4 Hz, 1H), 4.40 (s, 2H), 3.66 (s, 3H), 3.40 (s, 3H), 2.97 (s, 3H) ppm.

^{13}**C NMR** (75 MHz, $CDCl_3$) δ 156.2, 153.2, 152.8, 146.1, 137.6, 135.8, 132.9, 131.6, 130.8, 130.4, 130.1, 128.5, 128.3, 127.0, 125.4, 121.7, 121.2, 115.2, 113.7, 111.4, 109.5, 103.9, 94.1, 57.4, 55.5, 55.3, 32.8 ppm.

HRMS (ES+) Calcd for $C_{31}H_{29}NO_4Na$ $[M + Na]^+$: 502.1994, Found: 502.1997.

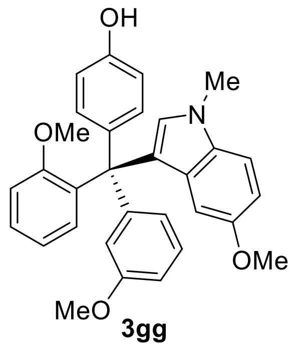

3gg

(*R*)-4-((5-Methoxy-1-methyl-1*H*-indol-3-yl)(2-methoxyphenyl)(3-methoxyphenyl) methyl)phenol (3gg) was prepared according to the General Procedure B as orange solid (chromatography eluent: hexanes/DCM = 5:2 → 5:4) in 79% yield (113.0 mg, 83% ee). The reaction time was 7 days.

$[\alpha]_D^{25}$: +15.2 (c = 1.0, $CHCl_3$). HPLC analysis of the product: Daicel CHIRALPAK AD-H column; 5% *i*-PrOH in hexanes, 1.0 mL/min; retention times: 21.0 min (major), 26.4 min (minor).

^{1}H NMR (300 MHz, $CDCl_3$) δ 7.27–7.02 (m, 6H), 6.85 (m, 4H), 6.77–6.69 (m, 2H), 6.65–6.57 (m, 3H), 6.04 (d, J = 2.5 Hz, 1H), 3.65 (s, 3H), 3.63 (s, 3H), 3.39 (s, 3H), 3.12 (s, 3H) ppm.

^{13}C NMR (75 MHz, $CDCl_3$) δ 158.6, 158.4, 153.2, 152.8, 148.2, 137.5, 136.0, 132.9, 131.6, 130.7, 130.4, 128.5, 128.2, 127.6, 122.9, 121.5, 120.3, 116.6, 113.7, 113.4, 111.2, 110.0, 109.4, 104.0, 57.3, 55.5, 55.4, 55.1, 32.8 ppm.

HRMS (ES+) Calcd for $C_{31}H_{29}NO_4Na$ $[M + Na]^+$: 502.1994, Found: 502.1997.

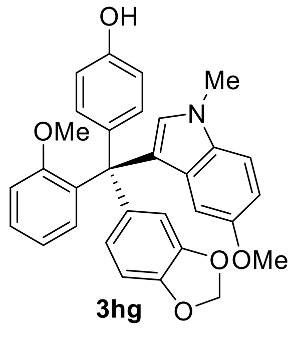

3hg

(*R*)-4-(Benzo[*d*][1,3]dioxol-5-yl(5-methoxy-1-methyl-1*H*-indol-3-yl)(2-methoxyphenyl)methyl)phenol (3hg) was prepared according to the General Procedure B as brown solid (chromatography eluent: hexanes/DCM = 5:2 → 5:4) in 93% yield (137.0 mg, 77% ee). The reaction time was 7 days.

$[\alpha]_D^{25}$: +4.2 (c = 1.0, $CHCl_3$). HPLC analysis of the product: Daicel CHIRALPAK IC column; 5% *i*-PrOH in hexanes, 1.0 mL/min; retention times: 16.4 min (major), 22.8 min (minor).

^{1}H NMR (300 MHz, $CDCl_3$) δ 7.28–7.16 (m, 2H), 7.13–6.99 (m, 3H), 6.90–6.56 (m, 9H), 6.05 (d, J = 2.4 Hz, 1H), 5.88 (q, J = 3.0 Hz, 2H), 3.65 (s, 3H), 3.42 (s, 3H), 3.17 (s, 3H) ppm.

^{13}C NMR (75 MHz, $CDCl_3$) δ 158.3, 153.2, 152.8, 146.5, 144.9, 140.3, 138.0, 136.1, 132.9, 131.4, 130.6, 130.3, 128.5, 128.2, 123.2, 121.7, 120.3, 113.7, 113.4, 111.2, 111.2, 109.5, 106.6, 104.0, 100.6, 57.1, 55.5, 55.4, 32.9 ppm.

HRMS (ES+) Calcd for $C_{31}H_{28}NO_5$ $[M+H]^+$: 494.1967, Found: 494.1964.

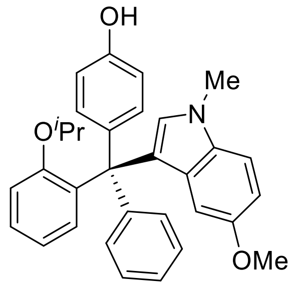

3pg

(*S*)-4-((2-Isopropoxyphenyl)(5-methoxy-1-methyl-1*H*-indol-3-yl)(phenyl)methyl) phenol (3pg) was prepared according to the General Procedure B as rufous solid (chromatography eluent: hexanes/DCM = 5:2 → 5:4) in 99% yield (143.0 mg, 84% ee). The reaction time was 24 h.

$[\alpha]_D^{25}$: +5.82 (c = 1.0, $CHCl_3$). HPLC analysis of the product: Daicel CHIRALPAK AD-H column; 5% *i*-PrOH in hexanes, 1.0 mL/min; retention times: 11.6 min (major), 13.5 min (minor).

^{1}**H NMR** (300 MHz, $CDCl_3$) δ 7.24–7.05 (m, 10H), 6.82–6.70 (m, 3H), 6.64 (d, J = 8.8 Hz, 2H), 6.55 (s, 1H), 6.02 (d, J = 2.5 Hz, 1H), 4.90 (s, 1H), 4.28 (p, J = 6.0 Hz, 1H), 3.64 (s, 3H), 3.38 (s, 3H), 0.69 (dd, J_1 = 6.0, J_2 = 20.1 Hz, 6H) ppm.

^{13}**C NMR** (75 MHz, $CDCl_3$) δ 155.5, 153.1, 152.7, 146.1, 137.7, 134.9, 132.8, 131.9, 131.2, 130.7, 130.3, 128.7, 127.8, 126.7, 125.1, 121.9, 118.7, 113.5, 111.4, 111.2, 109.3, 104.0, 67.5, 57.4, 55.5, 32.8, 20.9, 20.8 ppm.

HRMS (ES+) Calcd for $C_{32}H_{31}NO_3Na$ $[M + Na]^+$: 500.2202, Found: 500.2201.

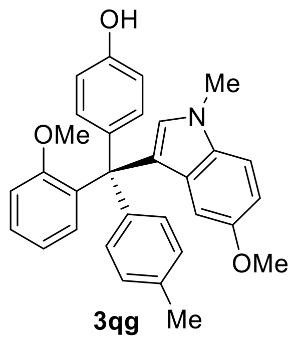

3qg

(*S*)-4-((5-Methoxy-1-methyl-1*H*-indol-3-yl)(2-methoxyphenyl)(p-tolyl)methyl) phenol (3qg) was prepared according to the General Procedure B as pink solid (chromatography eluent: hexanes/DCM = 5:2 → 5:4) in 99% yield (139.0 mg, 84% ee). The reaction time was 5 days.

$[\alpha]_D^{25}$: +2.00 (c = 1.0, $CHCl_3$). HPLC analysis of the product: Daicel CHIRALPAK AD-H column; 5% *i*-PrOH in hexanes, 1.0 mL/min; retention times: 14.2 min (minor), 17.4 min (major).

^{1}**H NMR** (300 MHz, $CDCl_3$) δ 7.27–7.15 (m, 2H), 7.13–6.94 (m, 7H), 6.88–6.76 (m, 2H), 6.72 (dd, J_1 = 2.5, J_1 = 8.8 Hz, 1H), 6.59 (d, J = 9.2 Hz, 3H), 6.02 (d, J = 2.4 Hz, 1H), 3.62 (s, 3H), 3.38 (s, 3H), 3.10 (s, 3H), 2.28 (s, 3H) ppm.

^{13}**C NMR** (75 MHz, $CDCl_3$) δ 158.4, 153.1, 152.6, 143.0, 138.1, 136.2, 134.6, 132.9, 131.4, 130.6, 130.4, 130.0, 128.5, 128.1, 127.6, 121.8, 120.2, 113.7, 113.5, 111.2, 109.4, 104.0, 56.9, 55.5, 55.4, 32.8, 20.9 ppm.

HRMS (ES+) Calcd for $C_{31}H_{29}NO_3Na$ $[M + Na]^+$: 486.2045, Found: 486.2046.

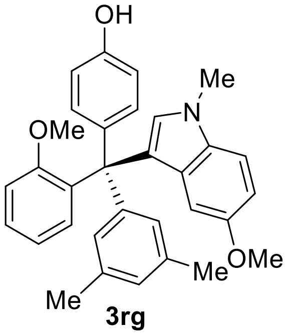

3rg

(*R*)-4-((3,5-Dimethylphenyl)(5-methoxy-1-methyl-1*H*-indol-3-yl)(2-methoxyphenyl) methyl)phenol (3rg) was prepared according to the General Procedure B as rufous solid (chromatography eluent: hexanes/DCM = 5:2 → 5:4) in 99% yield (143.0 mg, 82% ee). The reaction time was 5 days.

$[\alpha]_D^{25}$: +16.95 (*c* = 1.0, $CHCl_3$). HPLC analysis of the product: Daicel CHIRALPAK AD-H column; 5% *i*-PrOH in hexanes, 1.0 mL/min; retention times: 10.5 min (major), 14.3 min (minor).

^{1}H NMR (300 MHz, $CDCl_3$) δ 7.30–7.22 (m, 2H), 7.19 (dd, J_1 = 1.7, J_2 = 7.8 Hz, 1H), 7.12–7.02 (m, 3H), 6.90–6.78 (m, 5H), 6.73 (dd, J_1 = 2.5, J_2 = 8.8 Hz, 1H), 6.67–6.60 (d, *J* = 8.8, 2H), 6.58 (s, 1H), 6.03 (d, *J* = 2.4 Hz, 1H), 3.65 (s, 3H), 3.39 (s, 3H), 3.11 (s, 3H), 2.19 (s, 6H) ppm.

^{13}C NMR (75 MHz, $CDCl_3$) δ 158.6, 153.1, 152.7, 146.2, 137.9, 136.3, 136.0, 132.9, 131.7, 130.7, 130.4, 128.6, 128.0, 127.8, 126.9, 121.7, 120.3, 113.6 (2C), 111.2, 109.4, 104.0, 57.2, 55.5, 55.4, 32.8, 21.6 ppm.

HRMS (ES+) Calcd for $C_{32}H_{32}NO_3$ $[M + H]^+$: 478.2382, Found: 478.2372.

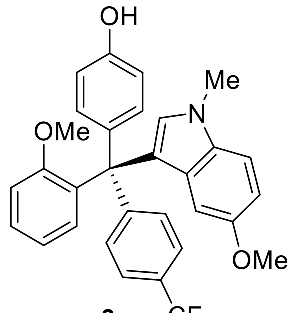

3sg

(*S*)-4-((5-Methoxy-1-methyl-1*H*-indol-3-yl)(2-methoxyphenyl)(4-(trifluoromethyl) phenyl)methyl)phenol (3sg) was prepared according to the General Procedure B as yellow solid (chromatography eluent: hexanes/DCM = 5:2 → 5:4) in 78% yield (120.0 mg, 91% ee). The reaction time was 7 days.

$[\alpha]_D^{25}$: 2.07 (c = 1.0, $CHCl_3$). HPLC analysis of the product: Daicel CHIRALPAK IC column; 3% *i*-PrOH in hexanes, 1.0 mL/min; retention times: 10.0 min (major), 11.7 min (minor).

^{1}H NMR (300 MHz, $CDCl_3$) δ 7.50–7.33 (m, 4H), 7.33–7.19 (m, 2H), 7.12 (d, J = 8.8 Hz, 1H), 7.08–6.99 (m, 2H), 6.91–6.81 (m, 2H), 6.76 (dd, J_1 = 8.8 Hz, J_2 = 2.5 Hz, 1H), 6.71–6.63 (m, 2H), 6.52 (s, 1H), 5.98 (d, J = 2.4 Hz, 1H), 4.90 (s, 1H), 3.66 (s, 3H), 3.39 (s, 3H), 3.14 (s, 3H) ppm.

^{13}C NMR (75 MHz, $CDCl_3$) δ 158.0, 153.4, 153.0, 150.5, 137.0, 135.1, 132.9, 131.5, 130.7, 130.4, 130.1, 128.5, 128.2, 127.4 (q, J = 32.3 Hz), 124.4 (q, J = 270.0 Hz), 123.8 (q, J = 3.8 Hz), 121.0, 120.4, 113.9, 113.1, 111.5, 109.7, 103.7, 57.3, 55.5, 55.1, 32.9 ppm.

^{19}F NMR (282 MHz, $CDCl_3$) δ -62.1 ppm.

HRMS (ES+) Calcd for $C_{31}H_{26}F_3NO_3Na$ $[M + Na]^+$: 540.1762, Found: 540.1765.

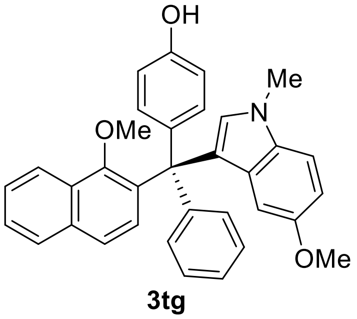

3tg

(*S*)-4-((5-Methoxy-1-methyl-1*H*-indol-3-yl)(1-methoxynaphthalen-2-yl)(phenyl)methyl)phenol (3tg) was prepared according to the General Procedure B as yellow solid (chromatography eluent: hexanes/DCM = 5:2 → 5:4) in 88% yield (132.0 mg, 86% ee). The reaction time was 36 h.

$[\alpha]_D^{25}$: +6.88 (c = 1.0, $CHCl_3$). HPLC analysis of the product: Daicel CHIRALPAK AD-H column; 5% *i*-PrOH in hexanes, 1.0 mL/min; retention times: 20.4 min (major), 29.0 min (minor).

^{1}H NMR (300 MHz, $CDCl_3$) δ 8.00–7.94 (m, 1H), 7.84–7.76 (m, 1H), 7.48–7.26 (m, 6H), 7.24–7.07 (m, 5H), 7.05 (d, J = 8.8 Hz, 1H), 6.71 (dd, J_1 = 2.5, J_2 = 8.8 Hz, 1H), 6.64 (s, 1H), 6.57 (d, J = 8.7 Hz, 2H), 6.00 (d, J = 2.5 Hz, 1H), 3.59 (s, 3H), 3.32 (s, 3H), 2.67 (s, 3H) ppm.

^{13}C NMR (75 MHz, $CDCl_3$) δ 154.5, 153.5, 152.7, 146.2, 137.4, 135.4, 134.7, 133.0, 131.8, 130.6, 130.5, 128.7, 128.5, 128.2, 128.0, 127.2, 125.7, 125.6, 125.2, 123.1, 122.5, 121.7, 114.0, 111.4, 109.6, 103.9, 60.0, 57.7, 55.4, 32.8 ppm.

HRMS (ES+) Calcd for $C_{34}H_{30}NO_3$ $[M+H]^+$: 500.2226, Found: 500.2215.

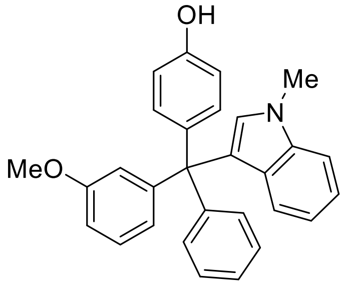

4da

4-((3-Methoxyphenyl)(1-methyl-1*H*-indol-3-yl)(phenyl)methyl)phenol (4da) was prepared according to the General Procedure B as grey solid (chromatography eluent: hexanes/DCM = 5:2 → 5:4) in 96% yield (120.8 mg, 0% ee). The reaction time was 3 days.

^{1}H NMR (400 MHz, $CDCl_3$) δ 7.26–7.16 (m, 6H), 7.14–7.06 (m, 3H), 6.81–6.65 (m, 8H), 4.94 (d, J = 13.9 Hz, 1H), 3.70 (s, 3H), 3.65 (s, 3H) ppm.

^{13}**C NMR** (101 MHz, $CDCl_3$) δ 158.7, 153.6, 148.7, 146.7, 138.7, 137.6, 131.9, 130.6, 130.0, 128.2, 128.0, 127.3, 125.9, 123.7, 122.9, 122.2, 121.2, 118.6, 117.2, 114.1, 110.5, 108.9, 58.7, 55.1, 32.7 ppm.

HRMS (ES+) Calcd for $C_{29}H_{25}NO_2Na$ $[M + Na]^+$: 442.1792, Found: 442.1783.

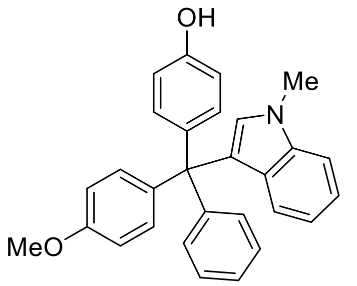

4ea

4-((4-Methoxyphenyl)(1-methyl-1*H*-indol-3-yl)(phenyl)methyl)phenol (4ea) was prepared according to the General Procedure B as yellow solid (chromatography eluent: hexanes/DCM = 5:2 → 5:4) in 98% yield (123.3 mg, 14% ee). The reaction time was 3 days.

$[\alpha]_D^{25}$: +1.2 (c = 1.0, $CHCl_3$). HPLC analysis of the product: Daicel CHIRALPAK IC column; 2% *i*-PrOH in hexanes, 0.4 mL/min; retention times: 53.6 min (major), 58.5 min (minor).

^{1}**H NMR** (400 MHz, $CDCl_3$) δ 7.26–7.10 (m, 9H), 7.06 (d, J = 8.7 Hz, 2H), 6.77 (q, J = 7.4 Hz, 3H), 6.67–6.62 (m, 4H), 4.97 (s, 1H), 3.77 (s, 3H), 3.69 (s, 3H) ppm.

^{13}**C NMR** (101 MHz, $CDCl_3$) δ 157.4, 153.5, 147.1, 139.2, 139.1, 137.7, 131.8, 131.6, 130.6, 130.0, 128.2, 127.3, 125.8, 122.9, 122.7, 121.1, 118.6, 114.1, 112.5, 108.9, 58.0, 55.1, 32.7 ppm.

HRMS (ES+) Calcd for $C_{29}H_{25}NO_2Na$ $[M + Na]^+$: 442.1792, Found: 442.1783.

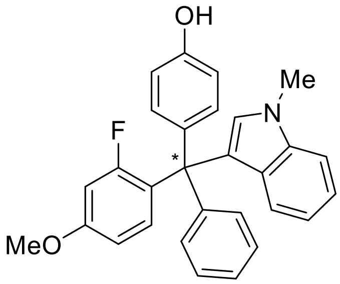

4fa

4-((2-Fluoro-4-methoxyphenyl)(1-methyl-1*H*-indol-3-yl)(phenyl)methyl)phenol (4fa): was prepared according to the General Procedure B as yellow solid (chromatography eluent: hexanes/DCM = 5:2 → 5:4) in 97% yield (131.0 mg, 81% ee). The reaction time was 2 days.

$[\alpha]_D^{25}$: -0.92 (*c* = 1.0, $CHCl_3$). HPLC analysis of the product: Daicel CHIRALPAK AD-H column; 10% *i*-PrOH in hexanes, 1.0 mL/min; retention times: 11.3 min (minor), 12.2 min (major).

^{1}H NMR (300 MHz, $CDCl_3$) δ 7.24–7.05 (m, 10H), 6.75 (t, *J* = 7.8 Hz, 1H), 6.64–6.51 (m, 6H), 5.00 (s, 1H), 3.73 (s, 3H), 3.65 (s, 3H) ppm.

^{13}C NMR (75 MHz, $CDCl_3$) δ 161.5 (d, *J* = 247.9 Hz), 159.76 (d, *J* = 11.3 Hz), 153.5, 145.5, 137.6, 137.4, 131.4 (d, *J* = 5.3 Hz), 131.2, 129.8, 127.8, 127.4, 126.7 (d, *J* = 12.0 Hz), 125.9, 122.2, 121.1, 121.0, 118.7, 114.2, 109.0, 108.4 (d, *J* = 2.3 Hz), 102.6 (d, *J* = 26.3 Hz), 102.4, 56.0, 55.4, 32.7 ppm.

HRMS (ES+) Calcd for $C_{29}H_{27}NO_2FNa$ $[M + Na]^+$: 460.1689, Found: 460.1682.

^{19}F NMR (282 MHz, $CDCl_3$) δ -99.0 ppm.

IV. Product Derivatizations

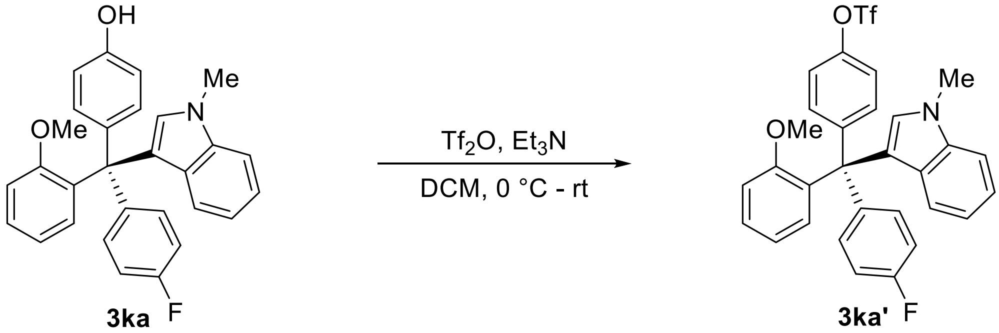

(*R*)-4-((4-Fluorophenyl)(2-methoxyphenyl)(1-methyl-1*H*-indol-3-yl)methyl)phenyl trifluoromethanesulfonate (3ka′). At 0 °C, a solution of **3ka** (437.5 mg, 1.0 mmol, 1.0 equiv.) and Et_3N (121.4 mg, 1.2 mmol, 1.2 equiv.) in DCM (5 mL) was stirred and treated dropwise with Tf_2O (338.6 mg, 1.2 mmol, 1.2 equiv.), and the mixture was allowed to warm to room temperature and stirred for 1 h. Then the reaction was quenched with water and extracted with DCM, the combined organic layer was dried over anhydrous Na_2SO_4 and concentrated under reduced pressure. The residue was further purified by silica gel flash chromatography to afford the desired product **3ka′** as white solid in 73.4% yield (418.2 mg).

^{1}H NMR (400 MHz, $CDCl_3$) δ 7.31–7.09 (m, 10H), 6.91–6.86 (m, 3H), 6.83 (td, J_1 = 7.2 Hz, J_2 = 1.0 Hz, 1H), 6.76 (td, J_1 = 7.6 Hz, J_2 = 0.6 Hz, 1H), 6.53 (t, J = 8.1 Hz, 2H), 3.71 (s, 3H), 3.16 (s, 3H) ppm.

^{13}C NMR (101 MHz, $CDCl_3$) δ 160.9 (d, J = 246.4 Hz), 157.7, 147.3, 146.7, 140.2, 137.6, 134.9, 131.6 (d, J = 8.1 Hz), 131.5, 130.6, 129.9, 128.8, 127.7, 122.1, 121.4, 121.3, 120.4, 119.7, 118.9, 117.1, 113.9 (d, J = 21.2 Hz), 113.1, 109.1, 57.1, 54.9, 32.8 ppm.

^{19}F NMR (282 MHz, $CDCl_3$) δ -72.8, -117.5 ppm.

HRMS (ES+) Calcd for $C_{30}H_{24}NO_4SF_4$ $[M+H]^+$: 570.1362, Found: 570.1362.

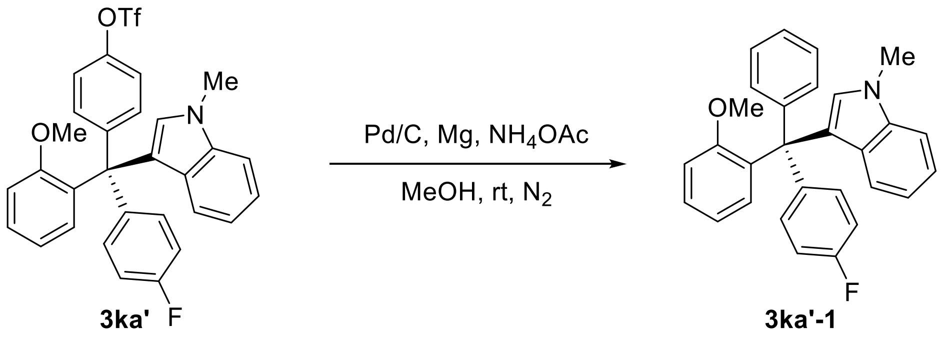

(*R*)-3-((4-Fluorophenyl)(2-methoxyphenyl)(phenyl)methyl)-1-methyl-1*H*-indole (3ka′-1). Under N_2, an oven-dried bottom equipped with magnetic stir bar was charged with triflate **3ka′** (114 mg, 0.2 mmol, 1.0 equiv.), Pd/C (12 mg, 10 wt%), Mg (5.6 mg, 0.24 mmol, 1.2 equiv.), NH_4OAc (18.6 mg, 0.24 mmol, 1.2 equiv.) and MeOH (2 mL). The reaction mixture was stirred at room temperature for 16 h, then the mixture was filtered through a pad of celite and the filtrate was evaporated under reduced pressure, the residue was further purified by silica gel chromatography (eluent: hexanes/EtOAc = 20:1) to afford the desired product **3ka′-1** as light yellow solid (83.5 mg, 99% yield, 93% ee).

$[\alpha]_D^{25}$: +6.0 (c = 0.5, $CHCl_3$). HPLC analysis of the product: Daicel CHIRALPAK AD-H column; 1% *i*-PrOH in hexanes, 1.0 mL/min; retention times: 6.0 min (major), 6.4 min (minor).

^{1}H NMR (400 MHz, $CDCl_3$) δ 7.32–7.24 (m, 5H), 7.20 (d, J = 7.7 Hz, 1H), 7.15–7.09 (m, 5H), 6.91–6.87 (m, 3H), 6.83 (t, J = 7.5 Hz, 1H), 6.77 (t, J = 7.6 Hz, 1H), 6.53 (t, J = 8.1 Hz, 2H), 3.72 (s, 3H), 3.16 (s, 3H) ppm.

^{13}C NMR (101 MHz, $CDCl_3$) δ 160.9 (d, J = 245.9 Hz), 157.7, 147.3, 146.7, 137.6, 134.9, 131.6 (d, J = 7.7 Hz), 131.5, 130.6, 129.9, 128.8, 127.7, 122.1, 121.4, 121.3, 120.4, 119.7, 118.9, 114.0, 113.8, 113.1, 109.1, 57.1, 54.9, 32.8 ppm.

^{19}F NMR (282 MHz, $CDCl_3$) δ -118.3 ppm.

HRMS (ES+) Calcd for $C_{29}H_{24}NOF$ $[M+H]^+$: 422.1920, Found: 422.1926.

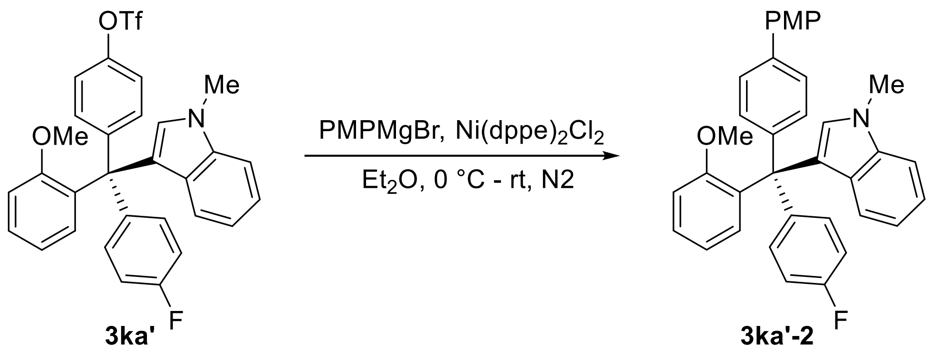

(*R*)-3-((4-Fluorophenyl)(4'-methoxy-[1,1'-biphenyl]-4-yl)(2-methoxyphenyl)methyl) -1-methyl-1*H*-indole (3ka′-2). Under N_2, an oven-dried bottom equipped with magnetic stir bar was charged with triflate **3ka′** (114 mg, 0.2 mmol, 1.0 equiv.), $Ni(dppe)_2Cl_2$ (5.2 mg, 5 mol%)and Et_2O (2 mL), then PMPMgBr (0.5 mmol, 5 equiv.) was added dropwise to the mixture at 0 °C. Upon completing, the resulting mixture was allowed to stirred at room temperature for 12 h, then the reaction was quenched with water and extracted with EtOAc, the combined organic layer was dried and evaporated under reduced pressure, further purified by silica gel chromatography (eluent: hexanes/EtOAc = 20:1) to afford the desired product **3ka′-2** as white solid in 70% yield (73.9 mg) and 89% ee.

$[\alpha]_D^{25}$: -15.6 (c = 1.0, $CHCl_3$). HPLC analysis of the product: Daicel CHIRALPAK AD-H column; 2% *i*-PrOH in hexanes, 1.0 mL/min; retention times: 7.1 min (minor), 7.6 min (major).

^{1}H NMR (300 MHz, $CDCl_3$) δ 7.57–7.54 (m, 2H), 7.45–7.42 (m, 2H), 7.31–7.22 (m, 7H), 7.12 (td, J_1 = 10.2 Hz, J_2 = 1.1 Hz, 1H), 6.99–6.86 (m, 6H), 6.78–6.73 (m, 1H), 6.65–6.61 (m, 2H), 3.82 (s, 3H), 3.70 (s, 3H), 3.16 (s, 3H) ppm.

^{13}C NMR (75 MHz, $CDCl_3$) δ 160.8 (d, J = 244.2 Hz), 158.9, 158.2, 144.2, 141.3 (d, J = 2.9 Hz), 137.6, 137.5, 135.7, 133.3, 131.8, 131.7 (d, J = 7.5 Hz), 130.4, 130.3 (d, J = 53.2 Hz), 128.4, 127.8, 127.0, 125.1, 122.4, 121.8, 121.1, 120.3, 118.7, 114.1, 113.6 (d, J = 20.7 Hz), 113.3, 108.9, 57.2, 55.3, 55.2, 32.7 ppm.

^{19}F NMR (376 MHz, $CDCl_3$) δ -118.2 ppm.

HRMS (ES+) Calcd for $C_{36}H_{30}NO_2Na$ $[M + Na]^+$: 550.2158, Found: 550.2152.

V. Mechanistic Experiments

Monitoring the ee values during the reaction process.

1a + **2a** $\xrightarrow[\text{Toluene (0.2 M), rt}]{(R)\text{-}\mathbf{A5}\ (10\ \text{mol\%})}$ **3aa**

At room temperature, to an oven-dried 10-mL vial charged with a solution of the tertiary alcohol **1a** (0.6 mmol) and **CPA** (*R*)-**A5** (56 mg, 0.06 mmol, 10 mol%) in toluene (3.0 mL), *N*-protected-indole **2a** (0.9 mmol) was added in one portion. The reaction mixture was stirred at the same temperature. Upon the corresponding reaction time, 100 μL reaction solution was extracted by syringe and injected to Et_3N (0.1 mL), then the resulting solution was passed through a short column of silica gel and concentrated under reduced pressure, the crude was analyzed by chiral HPLC for ee values of **1a** and **3aa**.

Time (h)	Ee of **1a**(%)	Ee of **3aa**(%)
1	0	87
12	0	86
24	0	88
36	0	90
48	0	92
60	0	92

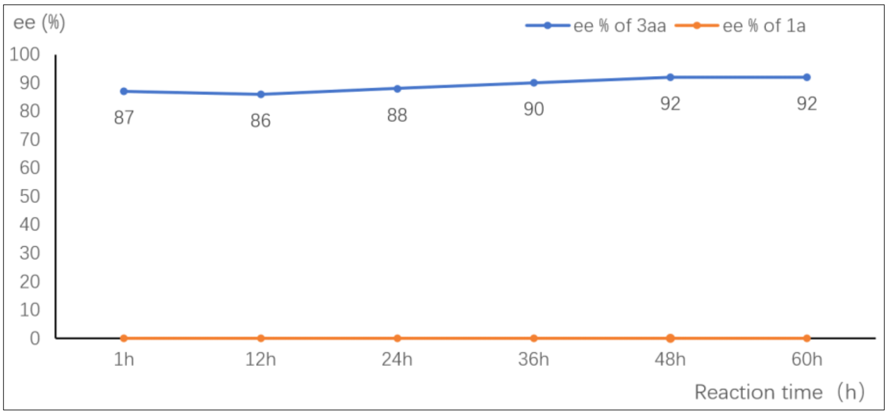

Figure S1. Original data and ee value-time plot of **2a** and **3aa**

VI. DFT Calculations

Computational details

All structures were optimized and characterized in toluene with the SMD[3] solvent model (SCRF = SMD) at B3LYP[4]-D3(BJ)[5]/6-31G(d) level. Harmonic frequency analysis calculations at the same level were performed to verify the optimized transition states (TSs, having unique one imaginary frequency). The energies were further improved by M06-2X[6]/6-311+G(d,p) //B3LYP-D3(BJ)/ 6-31G(d) single-point calculations with solvent effects accounted by the SMD solvent model, using the experimental solvent (toluene). All DFT calculations were carried out using Gaussian 09 program.[7] Computed structures are illustrated using the CYLview.[8]

Cartesian Coordinates in Å, SCF Energies and Free Energies (in a.u.) at 298.15 K and 1 atm for the Optimized Structures [BSI= 6-31G(d), BSII=6-311+G(d,p)

TS-*S*

B3LYP-D3/BSI SCF energy in toluene: -3907.622981 a.u.
M06-2X/BSII SCF energy in toluene: -3906.886197 a.u.
M06-2X/BSII free energy in toluene: -3905.573469 a.u.

P	1.49769800	0.22710400	-0.91362600
O	2.73025800	1.33428700	-1.00499200
O	2.15883000	-0.79517100	0.22055100
O	1.47904000	-0.41438500	-2.27747000
O	0.27475000	0.79909300	-0.26447500
C	-4.20590400	-0.91476600	0.57743300
C	-3.63686000	-1.29748200	1.93951300
C	-4.04231100	-2.53545000	2.46996400
C	-2.69870100	-0.54867700	2.66082100
C	-3.53985100	-2.99578200	3.68721300
C	-2.21153600	-0.99867100	3.88856800
C	-2.63011900	-2.22271000	4.41043300
C	-3.36731300	-1.29561900	-0.58585000
C	-3.75845600	-0.99559200	-1.91232300
C	-2.05462700	-1.77263400	-0.39133700
C	-2.85159800	-1.05060100	-2.96149000
C	-1.14637700	-1.83583700	-1.43215900
C	-1.50687500	-1.37246400	-2.71106500
C	-5.68396300	-1.17651300	0.51438100
C	-6.22585700	-2.06965400	-0.42526700
C	-6.56595300	-0.66849400	1.50852000
C	-7.57404200	-2.42667600	-0.41904900
C	-7.91881500	-1.02167400	1.51551500
C	-8.42083800	-1.89553100	0.54991400
O	-0.59815600	-1.18594200	-3.68398200
C	-4.07234800	1.18285900	0.53240100
C	-5.00660000	1.71257900	-0.46979500

C	-4.24347100	2.09328100	-1.59198400
C	-1.78103200	2.16781100	-2.19225600
H	-3.86756100	-3.95904700	4.06933700
H	-5.56734000	-2.53135300	-1.14975200
H	-7.94861200	-3.12822900	-1.15810800
H	-8.58332800	-0.62150300	2.27178600
H	-9.47348400	-2.16400900	0.57024700
H	0.26608400	-0.94631400	-3.22785000
H	-1.86066300	3.19649400	-2.54973300
H	-0.84097900	2.01140700	-1.66630200
H	-1.84903800	1.47262800	-3.03321700
H	-4.76055100	-0.63364300	-2.11153900
H	-3.14158300	-0.74760100	-3.96324800
H	-1.72317900	-2.06846900	0.59579600
H	-4.25455500	1.30353400	1.58976600
C	3.43224000	-1.28068700	0.02466900
C	4.52700000	-0.44370900	0.21677900
C	5.83517800	-0.96238700	-0.07774100
C	5.97958000	-2.35743000	-0.37342900
C	4.82645600	-3.18216900	-0.42771400
C	3.55528900	-2.66762700	-0.28511300
H	6.90349500	0.91887900	0.06095600
C	7.00010600	-0.14628900	-0.11668600
C	7.27405500	-2.88608500	-0.62990400
H	4.95230600	-4.24259700	-0.62433100
C	8.38409800	-2.07183800	-0.63354800
C	8.23898400	-0.68528500	-0.38816700
H	7.36716800	-3.94872400	-0.84217900
H	9.36747500	-2.48579300	-0.84000400
H	9.11160100	-0.03825700	-0.42041100
C	4.31202000	0.93789300	0.73752200
C	4.99156700	1.42580100	1.91022200
C	3.36970600	1.76231900	0.13497200

C	5.84664800	0.61901700	2.71277200
C	4.78048200	2.78465100	2.31988400
C	3.06474600	3.07339100	0.60400900
C	6.49805800	1.13830200	3.81081500
H	5.98104700	-0.42528500	2.45986000
C	5.48164900	3.29542700	3.44633200
C	3.81951600	3.57701800	1.64161400
C	6.33142000	2.49533100	4.17558400
H	7.14195900	0.49568300	4.40551800
H	5.31669400	4.33222000	3.73113900
H	3.62428000	4.58492600	1.99956200
H	6.85774300	2.89505900	5.03830700
C	1.88471000	3.81804900	0.06508100
C	1.85717200	4.28474200	-1.26975500
C	0.76395800	4.02617700	0.90497800
C	0.71828300	4.95161800	-1.72963500
C	-0.35017200	4.70690400	0.39485600
C	-0.39750400	5.17794300	-0.91730800
H	0.69803900	5.31579700	-2.75479100
H	-1.20439300	4.86077400	1.04679100
C	2.33632800	-3.52356200	-0.43984000
C	1.48798900	-3.76412600	0.66923100
C	2.02791200	-4.10186100	-1.69489500
C	0.35883800	-4.57081400	0.50116300
C	0.89235700	-4.91704200	-1.80234700
C	0.04278600	-5.16447400	-0.72417600
H	-0.28832900	-4.74621700	1.35620800
H	0.65614500	-5.36592700	-2.76375600
C	0.69348800	3.52670600	2.34952400
H	1.62902400	3.01779300	2.58731100
C	3.04466300	4.12288600	-2.21420800
H	3.81021100	3.53379900	-1.70341500
C	-1.58387100	5.95823500	-1.47218300

H	-1.67781000	5.68814900	-2.53467800
C	1.77452600	-3.20582400	2.06146600
H	2.67380800	-2.58729400	2.01246700
C	2.88905800	-3.88080400	-2.93819900
H	3.57567900	-3.05563300	-2.73311700
C	-1.18644400	-6.04919400	-0.87886900
H	-1.18864300	-6.42845400	-1.90977400
H	-0.13721600	-2.18470600	-1.25476900
H	-4.75218400	-3.14564600	1.92138700
H	-1.48873400	-0.39163800	4.42591900
H	-2.24313800	-2.57521600	5.36248600
H	-2.33132500	0.39542400	2.28408600
O	-6.02531900	0.18640100	2.41747500
C	-6.78599500	0.57195400	3.55930900
H	-7.12732400	-0.30495300	4.12253500
H	-6.10492400	1.15753400	4.17982700
H	-7.64683700	1.19454700	3.28388200
C	2.66641600	3.36233900	-3.49690700
H	3.55563400	3.20702700	-4.12044500
H	2.24518800	2.38114800	-3.26129400
H	1.93725600	3.91972200	-4.09831800
C	3.67184900	5.49119500	-2.54155800
H	2.96695900	6.13857300	-3.07804000
H	3.97749400	6.01542500	-1.62814900
H	4.55904900	5.36597900	-3.17481200
C	-1.29995900	7.47223100	-1.40912900
H	-0.37605900	7.72521300	-1.94129700
H	-2.12109800	8.04649800	-1.85665400
H	-1.18449500	7.79809500	-0.36779600
C	-2.92230400	5.63604600	-0.79167700
H	-3.11424100	4.55894100	-0.75629300
H	-2.95167000	6.01368900	0.23736600
H	-3.74938800	6.10812800	-1.33457000

C	0.62733100	-2.31218600	2.56089800
H	0.88384400	-1.86950600	3.53170600
H	0.43198000	-1.49828200	1.85807000
H	-0.29694500	-2.88380800	2.69801600
C	2.06739500	-4.33969500	3.06239100
H	2.32384500	-3.92600700	4.04606600
H	1.19845100	-4.99634300	3.19301900
H	2.90712400	-4.95975300	2.72655800
C	-2.49029900	-5.25550300	-0.67561100
H	-3.36392400	-5.90548500	-0.81500400
H	-2.54481900	-4.83348500	0.33525000
H	-2.56250200	-4.42421800	-1.38327000
C	-1.13340600	-7.26465600	0.06447000
H	-1.16131100	-6.95305200	1.11584700
H	-1.99008800	-7.92792600	-0.10903400
H	-0.21617300	-7.84556300	-0.08611300
C	2.06274600	-3.47671900	-4.17055600
H	1.41830800	-2.62409700	-3.95165100
H	2.73196500	-3.19881800	-4.99417700
H	1.42805900	-4.29623200	-4.52935400
C	3.72844000	-5.13410700	-3.25278100
H	3.07937500	-5.98946700	-3.47991600
H	4.37253300	-4.95980600	-4.12377900
H	4.36811600	-5.42159900	-2.41123100
C	0.54826700	4.69390700	3.34200200
H	0.56447800	4.32435200	4.37504300
H	1.36463700	5.41752800	3.22948400
H	-0.39522100	5.23470700	3.19775800
C	-0.42380100	2.48495500	2.54070600
H	-0.44631200	2.14093300	3.58302000
H	-1.41284000	2.90066300	2.30726800
H	-0.23946000	1.62571200	1.89066100
C	-2.77004000	1.43896400	-0.03263300

H	-1.78308200	1.20778300	0.34643900
N	-2.88037300	1.90052200	-1.26880500
C	-4.79409300	2.58637600	-2.76798700
C	-6.18578300	2.70953600	-2.80555500
C	-6.97046300	2.35835900	-1.69648700
C	-6.39418200	1.86120900	-0.52387600
H	-4.17552700	2.86215000	-3.61560700
H	-6.66341200	3.08986800	-3.70360100
H	-8.04905500	2.47591400	-1.74972500
H	-7.01505600	1.60012300	0.32426700

TS-*R*
B3LYP-D3/BSI SCF energy in toluene: -3907.620941 a.u.
M06-2X/BSII SCF energy in toluene: -3906.885508 a.u.
M06-2X/BSII free energy in toluene: -3905.572188 a.u.

P	1.29770500	0.17357000	-0.94295600
O	2.83602800	0.78171000	-0.80482300
O	1.44871800	-1.18321100	0.02571600
O	1.17603000	-0.21982700	-2.39155200
O	0.29637800	1.05101900	-0.24946200
C	-4.38992000	-0.32818800	0.88983100
C	-3.88389800	-1.04380200	2.14690700
C	-4.68759600	-2.08685000	2.64806500
C	-2.65904600	-0.80491700	2.82752700
C	-4.35183500	-2.82418000	3.78122800
C	-2.35013100	-1.50352000	4.00357600
C	-3.18857600	-2.50895600	4.47781100
C	-3.70506300	-0.64761500	-0.38927700
C	-4.11577400	-0.06211500	-1.60958200
C	-2.48186300	-1.34199500	-0.38746400
C	-3.29249300	-0.07047100	-2.72760400
C	-1.64576000	-1.34023000	-1.48940200
C	-2.00251000	-0.62201100	-2.64502800
C	-5.89863500	-0.26908300	0.90248700

C	-6.67765200	-0.87106400	-0.09811200
C	-6.56603300	0.25994500	2.02430300
C	-8.07148000	-0.90825900	0.00283000
C	-7.95223300	0.22445000	2.12696300
C	-8.71549500	-0.35598000	1.10841700
O	-1.14041400	-0.41518100	-3.65659300
C	-3.89201000	1.69443800	1.13183300
C	-4.82385700	2.61865200	0.45104400
C	-4.16614100	3.10770800	-0.69773000
C	-1.85472000	2.86474100	-1.72589900
H	-5.00504800	-3.62368800	4.11743200
H	-6.19778700	-1.35197400	-0.94176500
H	-8.64865600	-1.38640300	-0.78385800
H	-8.43780900	0.64456100	3.00366000
H	-9.79880200	-0.38764800	1.18581300
H	-0.21570800	-0.40272100	-3.26535300
H	-0.93661400	2.33020500	-1.47807100
H	-2.23702100	2.52490100	-2.69142700
H	-1.66787900	3.94193400	-1.75717700
H	-5.04921700	0.48770700	-1.65790200
H	-3.58988000	0.43433200	-3.64199000
H	-2.15471000	-1.85679000	0.50482000
H	-3.82749800	1.65292500	2.21185900
C	2.60072300	-1.94367200	-0.00270300
C	3.76458000	-1.44847500	0.57664700
C	4.95932900	-2.24833800	0.51941500
C	4.87727200	-3.58361800	0.00772500
C	3.63811600	-4.05952600	-0.48953000
C	2.51741900	-3.25958700	-0.54948100
H	6.33491500	-0.74824800	1.27233200
C	6.23677000	-1.77094000	0.92851000
C	6.04071300	-4.40008700	-0.01537300
H	3.57666600	-5.08461100	-0.84372300

C	7.25383300	-3.91564200	0.41785900
C	7.35038200	-2.58117300	0.87958600
H	5.95404900	-5.41480800	-0.39719400
H	8.13869400	-4.54581900	0.39043700
H	8.31410000	-2.18861400	1.19317300
C	3.77348200	-0.09458800	1.20000400
C	4.21744400	0.12990100	2.54825700
C	3.34041700	0.99585400	0.45834600
C	4.50179500	-0.92699900	3.45733200
C	4.35478600	1.47830600	3.01559400
C	3.45306300	2.34344800	0.91084200
C	4.93085900	-0.66583800	4.74069900
H	4.36906500	-1.95249900	3.13170400
C	4.81599100	1.71357300	4.33978700
C	3.99946100	2.55305600	2.16050800
C	5.10329100	0.66684900	5.18651000
H	5.13577900	-1.49101300	5.41769200
H	4.92533500	2.74248800	4.67514700
H	4.12311500	3.57019900	2.52223700
H	5.44933200	0.85930100	6.19854900
C	2.96576400	3.47679900	0.06588300
C	3.55485600	3.74101100	-1.19307200
C	1.89989200	4.29138500	0.52496100
C	3.05870700	4.79696800	-1.96638600
C	1.44887600	5.33824200	-0.28545900
C	2.00505900	5.60465100	-1.53871500
H	3.50923900	4.99749200	-2.93560700
H	0.62818500	5.95708900	0.06771200
C	1.22839400	-3.78834200	-1.10102100
C	0.27038200	-4.32547100	-0.21335100
C	0.97479800	-3.76273900	-2.49247400
C	-0.94631100	-4.78793900	-0.72513800
C	-0.25105100	-4.25826000	-2.95510300

C	-1.23055200	-4.75726400	-2.09236600
H	-1.69375600	-5.17211800	-0.03620500
H	-0.46687800	-4.23083800	-4.01904600
C	1.18899800	4.06463500	1.85893100
H	1.57079900	3.14097100	2.29926000
C	4.74299200	2.94853700	-1.73447400
H	4.97671100	2.14544600	-1.03165600
C	1.46724200	6.72452100	-2.41740600
H	2.08395100	6.75561300	-3.32566500
C	0.52344900	-4.39892300	1.28956200
H	1.55887900	-4.09847300	1.47430100
C	2.01085700	-3.22306500	-3.47781900
H	2.56060800	-2.42760400	-2.96500300
C	-2.57565600	-5.22935400	-2.62722300
H	-2.53668800	-5.15681300	-3.72255800
H	-0.69389100	-1.85126500	-1.44814300
H	-5.60343100	-2.33480300	2.12611900
H	-1.43553500	-1.27662500	4.53702600
H	-2.91972400	-3.04800200	5.38210600
C	4.42797600	2.29042700	-3.08970000
H	5.26699200	1.65841400	-3.40693100
H	3.53589000	1.66219600	-3.02133000
H	4.26582800	3.04002700	-3.87437300
C	5.99749800	3.83806900	-1.82616000
H	5.85323400	4.66553200	-2.53156700
H	6.24968800	4.27108900	-0.85065900
H	6.85944500	3.25285900	-2.17067700
C	1.57667700	8.09810800	-1.73236000
H	2.61196700	8.31818500	-1.44788200
H	1.23033900	8.89541100	-2.40173800
H	0.96564500	8.13992500	-0.82237400
C	0.01662700	6.44282900	-2.85173600
H	-0.05914700	5.48031700	-3.37091700

H	-0.65529300	6.41423300	-1.98435000
H	-0.34823400	7.22517300	-3.52922100
C	-0.37708700	-3.41298200	2.04968800
H	-0.13591300	-3.41106600	3.11958600
H	-0.24042800	-2.39942500	1.66257800
H	-1.43625600	-3.67828000	1.95300100
C	0.36545100	-5.82931000	1.83386200
H	0.62379000	-5.86347600	2.89975900
H	-0.66480900	-6.19120700	1.73313100
H	1.01956800	-6.53148900	1.30378500
C	-3.72271400	-4.32150500	-2.14365200
H	-4.68283300	-4.66117300	-2.55344700
H	-3.80209700	-4.33314600	-1.04970900
H	-3.56382100	-3.28350100	-2.45237800
C	-2.85055600	-6.70050400	-2.26795400
H	-2.93260500	-6.83746400	-1.18266200
H	-3.79203300	-7.04096900	-2.71692600
H	-2.04659700	-7.35408500	-2.62604400
C	1.39707700	-2.60288900	-4.74312400
H	0.57637800	-1.92385300	-4.50834600
H	2.16272700	-2.03690100	-5.28614900
H	1.01577600	-3.37077400	-5.42863500
C	3.00913000	-4.32329200	-3.89071600
H	2.48611000	-5.15075800	-4.38767300
H	3.75096800	-3.92314400	-4.59360700
H	3.54922700	-4.73407700	-3.03408800
C	1.47037900	5.20398400	2.85362900
H	0.97965800	5.00880100	3.81585200
H	2.54461500	5.32075700	3.03907200
H	1.09535000	6.16326000	2.47572800
C	-0.32109300	3.86032200	1.65585800
H	-0.80472700	3.56858000	2.59687700
H	-0.81962700	4.76894200	1.29686000

H	-0.47352800	3.06771500	0.92364900
C	-2.66861600	1.82949400	0.36975500
H	-1.72809300	1.31582100	0.49493000
N	-2.85132200	2.58624600	-0.69458400
C	-4.74254800	3.97632800	-1.61358600
C	-6.05166900	4.39093400	-1.35015300
C	-6.72142700	3.95349500	-0.19992100
C	-6.11813600	3.07651100	0.70816000
H	-4.19999500	4.32524700	-2.48610100
H	-6.54663800	5.06914600	-2.03879300
H	-7.73128600	4.30336300	-0.00584100
H	-6.65626300	2.76167700	1.59257600
H	-5.98228400	0.69759800	2.82857700
O	-1.81516100	0.11474800	2.29943500
C	-0.51542200	0.32462400	2.86367700
H	0.02386800	-0.61858000	2.98845300
H	0.02059000	0.93286400	2.13608500
H	-0.58845400	0.84172200	3.82863300

VII. Determination of the Absolute Stereochemistry

The absolute stereochemistry of product **3qg** was unambiguously determined by X-ray diffraction. The X-ray data have been deposited at the Cambridge Crystallographic Data Center (CCDC 2226390). The stereochemistry of other products was assumed by analogy.

The X-ray data were collected by SuperNova, Dual, Cu at zero, Atlas diffractometer. A suitable crystal of **3qg** was obtained from petroleum ether and dichloromethane by vapor deposition. The crystal was kept at 100.01(10) K during data collection. Using Olex2,1 the structure was solved with the SHELXT2 structure solution program using Intrinsic Phasing and refined with the SHELXL refinement package using Least Squares minimization.

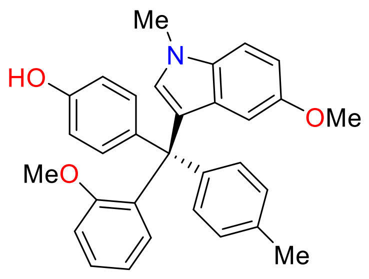

3qg CCDC 2226390

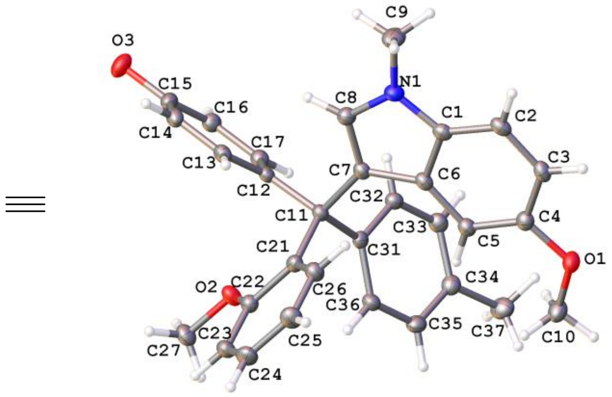

Table S1. Crystal data and structure refinement for 3qg.

Identification code	**3qg**
Empirical formula	$C_{32}H_{33}NO_4$
Formula weight	495.59
Temperature/K	100.01(10)
Crystal system	monoclinic
Space group	$P2_1$
a/Å	13.02736(13)
b/Å	7.62610(7)
c/Å	13.31312(13)
α/°	90

β/°	97.9813(9)
γ/°	90
Volume/Å^3	1309.82(2)
Z	2
ρ_{calc}g/cm^3	1.257
μ/mm^{-1}	0.655
F(000)	528.0
Crystal size/mm^3	0.2 × 0.18 × 0.18
Radiation	CuKα (λ = 1.54184)
2Θ range for data collection/°	6.704 to 153.748
Index ranges	-14 ≤ h ≤ 16, -9 ≤ k ≤ 9, -16 ≤ l ≤ 13
Reflections collected	8645
Independent reflections	5291 [R_{int} = 0.0181, R_{sigma} = 0.0226]
Data/restraints/parameters	5291/1/342
Goodness-of-fit on F^2	1.032
Final R indexes [I>=2σ (I)]	R_1 = 0.0247, wR_2 = 0.0647
Final R indexes [all data]	R_1 = 0.0256, wR_2 = 0.0652
Largest diff. peak/hole / e Å^{-3}	0.18/-0.12
Flack parameter	0.01(6)

Table S2. Fractional Atomic Coordinates (×10^4) and Equivalent Isotropic Displacement Parameters (Å^2×10^3) for 3qg. U_{eq} is defined as 1/3 of the trace of the orthogonalised U_{IJ} tensor.

Atom	*x*	*y*	*z*	U(eq)
O(1)	3162.7(8)	972.6(16)	2076.8(9)	22.3(2)
O(2)	8585.2(9)	4059.0(15)	4674.8(8)	21.5(2)
O(3)	11191.1(9)	6004.7(18)	1769.9(10)	29.0(3)
N(1)	6773.2(11)	430.7(19)	412.6(10)	19.8(3)
C(1)	5823.3(12)	389(2)	749.6(11)	17.6(3)
C(2)	4896.6(12)	-372(2)	300.8(11)	20.1(3)
C(3)	4030.4(12)	-145(2)	773.4(12)	20.1(3)
C(4)	4093.6(12)	808(2)	1687.9(12)	18.9(3)
C(5)	5011.1(12)	1537(2)	2150.6(11)	17.8(3)
C(6)	5904.3(12)	1342(2)	1668.8(11)	16.6(3)
C(7)	6959.9(12)	1968(2)	1881.6(11)	16.4(3)
C(8)	7442.4(12)	1376(2)	1090.7(12)	19.0(3)
C(9)	7023.8(14)	-465(3)	-485.8(13)	29.7(4)
C(10)	3161.8(13)	2023(2)	2967.5(13)	24.7(3)
C(11)	7453.5(11)	2991(2)	2821.3(11)	15.7(3)
C(12)	8449.5(12)	3896(2)	2565.7(11)	16.5(3)
C(13)	9312.1(12)	2855(2)	2452.0(11)	19.5(3)

Table S2. Fractional Atomic Coordinates (×10⁴) and Equivalent Isotropic Displacement Parameters (Å²×10³) for 3qg. U_{eq} is defined as 1/3 of the trace of the orthogonalised U_{IJ} tensor.

Atom	*x*	*y*	*z*	U(eq)
C(14)	10214.1(12)	3571(2)	2183.1(12)	21.3(3)
C(15)	10285.5(12)	5373(2)	2032.7(11)	21.1(3)
C(16)	9447.1(12)	6436(2)	2165.8(12)	20.7(3)
C(17)	8544.9(12)	5703(2)	2428.6(11)	18.7(3)
C(21)	7756.3(11)	1716(2)	3716.6(11)	17.2(3)
C(22)	8337.3(12)	2317(2)	4629.8(12)	18.4(3)
C(23)	8620.4(12)	1172(2)	5432.8(12)	21.2(3)
C(24)	8324.2(13)	-582(2)	5350.5(13)	24.5(3)
C(25)	7749.0(14)	-1201(2)	4470.4(13)	24.4(3)
C(26)	7473.0(12)	-45(2)	3663.6(12)	20.8(3)
C(27)	9178.8(13)	4692(2)	5583.2(12)	24.4(3)
C(31)	6622.5(11)	4303(2)	3070.0(11)	15.9(3)
C(32)	6149.7(12)	5418(2)	2308.4(11)	17.7(3)
C(33)	5285.6(12)	6407(2)	2440.7(12)	20.5(3)
C(34)	4846.9(12)	6311(2)	3338.1(12)	20.6(3)
C(35)	5335.8(13)	5252(2)	4111.0(12)	21.1(3)
C(36)	6213.5(12)	4275(2)	3984.2(11)	18.6(3)
C(37)	3860.3(14)	7281(2)	3451.2(14)	27.7(4)
O(1S)	1244.8(9)	-501.3(19)	1447.0(9)	29.1(3)
C(1S)	859.2(16)	17(3)	433.7(15)	36.8(4)

Table S3. Anisotropic Displacement Parameters (Å²×10³) for 3qg. The Anisotropic displacement factor exponent takes the form: $-2\pi^2[h^2a^{*2}U_{11}+2hka^*b^*U_{12}+\ldots]$.

Atom	U_{11}	U_{22}	U_{33}	U_{23}	U_{13}	U_{12}
O(1)	16.1(5)	26.4(6)	24.3(6)	-5.5(5)	1.9(4)	-2.5(4)
O(2)	25.0(6)	20.5(6)	17.4(5)	0.0(4)	-3.4(4)	-3.4(4)
O(3)	19.3(6)	34.1(7)	35.6(7)	-2.6(6)	10.5(5)	-5.4(5)
N(1)	22.4(7)	21.9(6)	15.3(6)	-2.1(5)	3.3(5)	-1.4(5)
C(1)	21.5(7)	16.5(7)	14.6(6)	1.5(6)	1.4(5)	0.5(6)
C(2)	26.2(8)	18.2(7)	14.6(7)	-0.2(6)	-1.7(6)	-0.6(6)
C(3)	19.2(7)	19.0(7)	20.2(7)	-0.3(6)	-3.7(6)	-2.2(6)
C(4)	18.1(7)	18.4(7)	19.7(7)	1.4(6)	1.2(6)	0.6(6)
C(5)	18.9(7)	17.7(7)	16.3(7)	-0.5(6)	0.3(5)	-0.6(6)
C(6)	18.8(7)	15.1(7)	14.8(6)	0.6(6)	-1.2(5)	-0.1(6)
C(7)	17.2(7)	16.1(7)	15.3(7)	1.2(5)	0.1(5)	-0.3(5)
C(8)	19.5(7)	19.9(7)	17.8(7)	-0.3(6)	2.8(5)	-1.6(6)
C(9)	29.5(8)	37.9(10)	23.0(8)	-12.4(8)	8.8(7)	-5.4(8)
C(10)	19.0(7)	28.1(8)	27.4(8)	-6.7(7)	5.0(6)	-0.8(6)
C(11)	15.8(6)	17.0(7)	13.7(7)	0.0(6)	0.0(5)	-0.7(6)

Table S3. Anisotropic Displacement Parameters (Å^2×10^3) for 3qg. The Anisotropic displacement factor exponent takes the form: -2π^2[h^{2}a*^{2}U$_{11}$+2hka*b*U$_{12}$+…].

Atom	U_{11}	U_{22}	U_{33}	U_{23}	U_{13}	U_{12}
C(12)	15.5(7)	21.5(7)	11.8(6)	-0.3(5)	-0.5(5)	-1.4(6)
C(13)	19.2(7)	21.6(7)	16.6(7)	0.2(6)	-1.3(6)	0.9(6)
C(14)	16.8(7)	28.3(8)	18.4(7)	-3.5(6)	0.5(6)	2.9(6)
C(15)	16.7(7)	30.3(8)	16.3(7)	-3.0(6)	2.7(6)	-4.3(6)
C(16)	21.2(7)	21.8(8)	18.9(7)	-1.2(6)	2.3(6)	-3.7(6)
C(17)	18.6(7)	20.7(8)	16.6(7)	-2.2(6)	1.8(6)	-0.6(6)
C(21)	15.1(6)	20.0(7)	16.3(7)	2.2(6)	1.7(5)	0.6(5)
C(22)	16.7(7)	20.7(7)	17.6(7)	0.1(6)	1.8(5)	-0.2(6)
C(23)	20.6(7)	27.5(8)	15.0(7)	1.3(6)	0.2(6)	-0.6(6)
C(24)	26.0(8)	27.4(8)	19.7(8)	8.6(6)	1.9(6)	1.4(7)
C(25)	28.1(8)	20.2(8)	24.6(8)	4.5(6)	2.1(7)	-2.7(6)
C(26)	21.3(7)	21.3(8)	19.1(7)	1.4(6)	0.7(6)	-1.2(6)
C(27)	24.7(8)	26.4(8)	20.5(8)	-3.8(6)	-3.1(6)	-2.2(7)
C(31)	14.3(6)	16.4(7)	16.6(7)	-1.6(6)	1.0(5)	-3.5(5)
C(32)	19.1(7)	18.5(7)	15.5(7)	-0.2(6)	2.3(5)	-2.2(6)
C(33)	21.8(7)	18.9(7)	20.3(7)	1.9(6)	1.0(6)	0.1(6)
C(34)	19.9(7)	20.0(7)	22.1(8)	-4.1(6)	3.1(6)	-0.1(6)
C(35)	22.8(8)	24.5(8)	16.4(7)	-3.4(6)	4.6(6)	-2.2(6)
C(36)	19.8(7)	21.4(7)	14.2(7)	-0.6(6)	0.5(5)	-1.1(6)
C(37)	28.2(9)	28.7(9)	27.3(9)	-1.2(7)	7.4(7)	6.6(7)
O(1S)	22.3(6)	36.0(7)	28.0(6)	0.9(5)	0.6(5)	-8.7(5)
C(1S)	35.8(10)	45.1(12)	27.3(9)	-3.6(8)	-3.6(7)	1.2(9)

Table S4. Bond Lengths for 3qg.

Atom	Atom	Length/Å	Atom	Atom	Length/Å
O(1)	C(4)	1.3888(19)	C(12)	C(13)	1.401(2)
O(1)	C(10)	1.431(2)	C(12)	C(17)	1.398(2)
O(2)	C(22)	1.3668(19)	C(13)	C(14)	1.387(2)
O(2)	C(27)	1.4262(18)	C(14)	C(15)	1.394(2)
O(3)	C(15)	1.3642(19)	C(15)	C(16)	1.391(2)
N(1)	C(1)	1.374(2)	C(16)	C(17)	1.389(2)
N(1)	C(8)	1.370(2)	C(21)	C(22)	1.416(2)
N(1)	C(9)	1.453(2)	C(21)	C(26)	1.391(2)
C(1)	C(2)	1.397(2)	C(22)	C(23)	1.390(2)
C(1)	C(6)	1.414(2)	C(23)	C(24)	1.392(2)
C(2)	C(3)	1.377(2)	C(24)	C(25)	1.383(2)
C(3)	C(4)	1.411(2)	C(25)	C(26)	1.398(2)
C(4)	C(5)	1.383(2)	C(31)	C(32)	1.399(2)
C(5)	C(6)	1.412(2)	C(31)	C(36)	1.395(2)
C(6)	C(7)	1.446(2)	C(32)	C(33)	1.387(2)

Table S4. Bond Lengths for 3qg.

Atom	Atom	Length/Å	Atom	Atom	Length/Å
C(7)	C(8)	1.375(2)	C(33)	C(34)	1.396(2)
C(7)	C(11)	1.538(2)	C(34)	C(35)	1.391(2)
C(11)	C(12)	1.549(2)	C(34)	C(37)	1.509(2)
C(11)	C(21)	1.546(2)	C(35)	C(36)	1.395(2)
C(11)	C(31)	1.544(2)	O(1S)	C(1S)	1.428(2)

Table S5. Bond Angles for 3qg.

Atom	Atom	Atom	Angle/˚	Atom	Atom	Atom	Angle/˚
C(4)	O(1)	C(10)	117.43(12)	C(17)	C(12)	C(13)	117.21(14)
C(22)	O(2)	C(27)	117.60(13)	C(14)	C(13)	C(12)	121.73(15)
C(1)	N(1)	C(9)	124.98(14)	C(13)	C(14)	C(15)	120.06(15)
C(8)	N(1)	C(1)	108.46(13)	O(3)	C(15)	C(14)	117.56(16)
C(8)	N(1)	C(9)	126.48(14)	O(3)	C(15)	C(16)	123.29(16)
N(1)	C(1)	C(2)	129.34(14)	C(16)	C(15)	C(14)	119.14(15)
N(1)	C(1)	C(6)	107.96(13)	C(17)	C(16)	C(15)	120.28(15)
C(2)	C(1)	C(6)	122.67(14)	C(16)	C(17)	C(12)	121.54(15)
C(3)	C(2)	C(1)	117.83(14)	C(22)	C(21)	C(11)	120.45(13)
C(2)	C(3)	C(4)	120.37(14)	C(26)	C(21)	C(11)	122.02(14)
O(1)	C(4)	C(3)	114.77(14)	C(26)	C(21)	C(22)	117.53(14)
C(5)	C(4)	O(1)	122.89(14)	O(2)	C(22)	C(21)	116.56(13)
C(5)	C(4)	C(3)	122.34(15)	O(2)	C(22)	C(23)	122.76(14)
C(4)	C(5)	C(6)	118.10(14)	C(23)	C(22)	C(21)	120.67(14)
C(1)	C(6)	C(7)	107.08(13)	C(22)	C(23)	C(24)	120.13(15)
C(5)	C(6)	C(1)	118.67(14)	C(25)	C(24)	C(23)	120.38(15)
C(5)	C(6)	C(7)	134.22(14)	C(24)	C(25)	C(26)	119.17(16)
C(6)	C(7)	C(11)	127.29(13)	C(21)	C(26)	C(25)	122.11(15)
C(8)	C(7)	C(6)	105.36(13)	C(32)	C(31)	C(11)	119.33(13)
C(8)	C(7)	C(11)	127.27(14)	C(36)	C(31)	C(11)	122.78(13)
N(1)	C(8)	C(7)	111.14(14)	C(36)	C(31)	C(32)	117.31(14)
C(7)	C(11)	C(12)	108.42(12)	C(33)	C(32)	C(31)	121.48(14)
C(7)	C(11)	C(21)	109.95(12)	C(32)	C(33)	C(34)	121.06(15)
C(7)	C(11)	C(31)	106.07(11)	C(33)	C(34)	C(37)	121.01(15)
C(21)	C(11)	C(12)	108.54(12)	C(35)	C(34)	C(33)	117.65(14)
C(31)	C(11)	C(12)	113.09(12)	C(35)	C(34)	C(37)	121.32(14)
C(31)	C(11)	C(21)	110.72(12)	C(34)	C(35)	C(36)	121.30(14)
C(13)	C(12)	C(11)	118.69(14)	C(35)	C(36)	C(31)	121.09(14)
C(17)	C(12)	C(11)	124.09(14)				

Table S6. Torsion Angles for 3qg.

A	B	C	D	Angle/°	A	B	C	D	Angle/°
O(1)	C(4)	C(5)	C(6)	-177.72(14)	C(11)	C(12)	C(17)	C(16)	-177.77(13)
O(2)	C(22)	C(23)	C(24)	178.49(15)	C(11)	C(21)	C(22)	O(2)	1.4(2)
O(3)	C(15)	C(16)	C(17)	179.64(14)	C(11)	C(21)	C(22)	C(23)	-179.34(14)
N(1)	C(1)	C(2)	C(3)	-176.59(16)	C(11)	C(21)	C(26)	C(25)	179.76(15)
N(1)	C(1)	C(6)	C(5)	177.97(14)	C(11)	C(31)	C(32)	C(33)	169.15(14)
N(1)	C(1)	C(6)	C(7)	-0.20(17)	C(11)	C(31)	C(36)	C(35)	-167.94(14)
C(1)	N(1)	C(8)	C(7)	0.18(19)	C(12)	C(11)	C(21)	C(22)	53.95(18)
C(1)	C(2)	C(3)	C(4)	-0.8(2)	C(12)	C(11)	C(21)	C(26)	-126.20(15)
C(1)	C(6)	C(7)	C(8)	0.30(17)	C(12)	C(11)	C(31)	C(32)	66.14(17)
C(1)	C(6)	C(7)	C(11)	-176.66(14)	C(12)	C(11)	C(31)	C(36)	-122.84(15)
C(2)	C(1)	C(6)	C(5)	-0.2(2)	C(12)	C(13)	C(14)	C(15)	0.9(2)
C(2)	C(1)	C(6)	C(7)	-178.41(14)	C(13)	C(12)	C(17)	C(16)	1.5(2)
C(2)	C(3)	C(4)	O(1)	178.75(14)	C(13)	C(14)	C(15)	O(3)	179.96(13)
C(2)	C(3)	C(4)	C(5)	-0.5(2)	C(13)	C(14)	C(15)	C(16)	0.6(2)
C(3)	C(4)	C(5)	C(6)	1.5(2)	C(14)	C(15)	C(16)	C(17)	-1.1(2)
C(4)	C(5)	C(6)	C(1)	-1.1(2)	C(15)	C(16)	C(17)	C(12)	0.0(2)
C(4)	C(5)	C(6)	C(7)	176.49(16)	C(17)	C(12)	C(13)	C(14)	-2.0(2)
C(5)	C(6)	C(7)	C(8)	-177.46(17)	C(21)	C(11)	C(12)	C(13)	48.76(17)
C(5)	C(6)	C(7)	C(11)	5.6(3)	C(21)	C(11)	C(12)	C(17)	-131.93(15)
C(6)	C(1)	C(2)	C(3)	1.2(2)	C(21)	C(11)	C(31)	C(32)	-171.80(13)
C(6)	C(7)	C(8)	N(1)	-0.30(18)	C(21)	C(11)	C(31)	C(36)	-0.79(19)
C(6)	C(7)	C(11)	C(12)	-161.47(14)	C(21)	C(22)	C(23)	C(24)	-0.7(2)
C(6)	C(7)	C(11)	C(21)	80.02(18)	C(22)	C(21)	C(26)	C(25)	-0.4(2)
C(6)	C(7)	C(11)	C(31)	-39.73(19)	C(22)	C(23)	C(24)	C(25)	0.1(2)
C(7)	C(11)	C(12)	C(13)	-70.63(16)	C(23)	C(24)	C(25)	C(26)	0.3(3)
C(7)	C(11)	C(12)	C(17)	108.67(16)	C(24)	C(25)	C(26)	C(21)	-0.1(3)
C(7)	C(11)	C(21)	C(22)	172.38(13)	C(26)	C(21)	C(22)	O(2)	-178.42(14)
C(7)	C(11)	C(21)	C(26)	-7.8(2)	C(26)	C(21)	C(22)	C(23)	0.8(2)
C(7)	C(11)	C(31)	C(32)	-52.56(17)	C(27)	O(2)	C(22)	C(21)	-179.41(13)
C(7)	C(11)	C(31)	C(36)	118.46(15)	C(27)	O(2)	C(22)	C(23)	1.4(2)
C(8)	N(1)	C(1)	C(2)	178.07(16)	C(31)	C(11)	C(12)	C(13)	172.04(13)
C(8)	N(1)	C(1)	C(6)	0.02(18)	C(31)	C(11)	C(12)	C(17)	-8.7(2)
C(8)	C(7)	C(11)	C(12)	22.2(2)	C(31)	C(11)	C(21)	C(22)	-70.74(17)
C(8)	C(7)	C(11)	C(21)	-96.30(18)	C(31)	C(11)	C(21)	C(26)	109.11(16)
C(8)	C(7)	C(11)	C(31)	143.95(15)	C(31)	C(32)	C(33)	C(34)	-0.8(2)
C(9)	N(1)	C(1)	C(2)	-4.9(3)	C(32)	C(31)	C(36)	C(35)	3.2(2)
C(9)	N(1)	C(1)	C(6)	177.04(15)	C(32)	C(33)	C(34)	C(35)	3.0(2)
C(9)	N(1)	C(8)	C(7)	-176.78(16)	C(32)	C(33)	C(34)	C(37)	-175.30(16)
C(10)	O(1)	C(4)	C(3)	-175.99(14)	C(33)	C(34)	C(35)	C(36)	-2.1(2)
C(10)	O(1)	C(4)	C(5)	3.2(2)	C(34)	C(35)	C(36)	C(31)	-1.1(2)
C(11)	C(7)	C(8)	N(1)	176.66(15)	C(36)	C(31)	C(32)	C(33)	-2.4(2)
C(11)	C(12)	C(13)	C(14)	177.36(14)	C(37)	C(34)	C(35)	C(36)	176.19(16)

Table S7. Hydrogen Atom Coordinates (Å×10[4]) and Isotropic Displacement Parameters (Å[2]×10[3]) for 3qg.

**Atom**	***x***	***y***	***z***	**U(eq)**
H(3)	11155	7101	1717	44
H(2)	4865	-1024	-311	24
H(3A)	3387	-634	482	24
H(5)	5040	2151	2775	21
H(8)	8148	1593	1024	23
H(9A)	7039	-1734	-367	44
H(9B)	7704	-75	-632	44
H(9C)	6497	-191	-1064	44
H(10A)	3363	3226	2826	37
H(10B)	3656	1537	3519	37
H(10C)	2466	2025	3168	37
H(13)	9278	1627	2562	23
H(14)	10784	2832	2101	26
H(16)	9492	7668	2077	25
H(17)	7980	6447	2517	22
H(23)	9017	1587	6039	25
H(24)	8518	-1359	5902	29
H(25)	7544	-2396	4415	29
H(26)	7079	-474	3060	25
H(27A)	9319	5945	5511	37
H(27B)	9836	4050	5709	37
H(27C)	8789	4517	6154	37
H(32)	6427	5499	1687	21
H(33)	4987	7163	1912	25
H(35)	5067	5194	4738	25
H(36)	6538	3578	4529	22
H(37A)	4030	8464	3711	42
H(37B)	3484	6649	3927	42
H(37C)	3427	7360	2790	42
H(1S)	1856	-142	1598	44
H(1SA)	1315	-441	-31	55
H(1SB)	839	1300	392	55
H(1SC)	159	-452	246	55

VIII. References

1 X. G. Li, M. Duan, Z. Q. Deng, Q. Z. Shao, M. Chen, G. Y. Zhu, K. N. Houk and J. Sun, *Nat. Catal.*, 2020, **3**, 1010-1019.

2 Z. Han, Y. Zang, C. Liu, W. Guo, H. Huang and J. Sun, *Chem. Commun.*, 2022, **58**, 7128-7131.

3 A. V. Marenich, C. J. Cramer, D. G. Truhlar, *J. Phys. Chem. B.* 2009, **113**, 6378-6396.

4 (a) L. Noodleman, T. Lovell, W.-G. Han, J. Li, F. Himo, *Chem. Rev.* 2004, **104**, 459-508; (b) L. Noodleman, *J. Chem. Phys.*1981, **74**, 5737; (c) L. Noodleman, D. A. Case, *Adv. Inorg. Chem.* 1992, **38**, 423-458.

5 S. Grimme, J. Antony, Ehrlich, S. H. Krieg, *J. Chem. Phys.* 2010, **132**, 154104.

6 (a) Y. Zhao, D. G. Truhlar, *J. Chem. Theory Comput.* 2009, **5**, 324-333; (b) Y. Zhao, D. G. Truhlar, *Theor. Chem. Acc.* 2008, **120**, 215-241; (c) Y. Zhao, D. G. Truhlar, *Acc. Chem. Res.* 2008, **41**, 157-167.

7 M. J. Frisch, G. W. Trucks, H. B. Schlegel, G. E. Scuseria, M. A. Robb, J. R. Cheeseman, G. Scalmani, V. Barone, B. Mennucci, G. A. Petersson, H. Nakatsuji, M. Caricato, X. Li, H. P. Hratchian, A. F. Izmaylov, J. Bloino, G. Zheng, J. L. Sonnenberg, M. Hada, M. Ehara, K. Toyota, R. Fukuda, J. Hasegawa, , M. Ishida, T.Nakajima, Y. Honda, O. Kitao, H. Nakai, T. Vreven, J. A. Montgomery, Jr.; J. E. Peralta, F. Ogliaro, M. Bearpark, J. J. Heyd, E. Brothers, K. N. Kudin, V. N. Staroverov, T. Keith, R. Kobayashi, J. Normand, K. Raghavachari, A. Rendell, J. C. Burant, S. S. Iyengar, J. Tomasi, M. Cossi, N. Rega, J. M. Millam, M. Klene, J. E. Knox, J. B. Cross, V. Bakken, C. Adamo, J. Jaramillo, R. Gomperts, R. E. Stratmann, O. Yazyev, A. J. Austin, R. Cammi, C. Pomelli, J. W. Ochterski, R. L. Martin, K. Morokuma, V. G. Zakrzewski, G. A. Voth, P. Salvador, J. J. Dannenberg, S. Dapprich, A. D. Daniels, O. Farkas, J. B. Foresman, J. V. Ortiz, J. Cioslowski, and D. J. Fox, Gaussian 09, Rev. A.01; Gaussian, Inc.: Wallingford, CT, 2010.

8 C. Y. Legault, *CYLView, version 1.0 b*; Université de Sherbrooke: Sherbrooke, Quebec, Canada, 2009. http://www.cylview.org.

1e

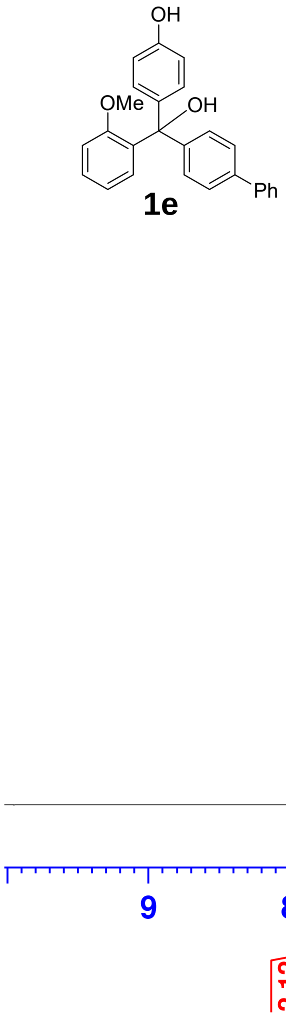

1e

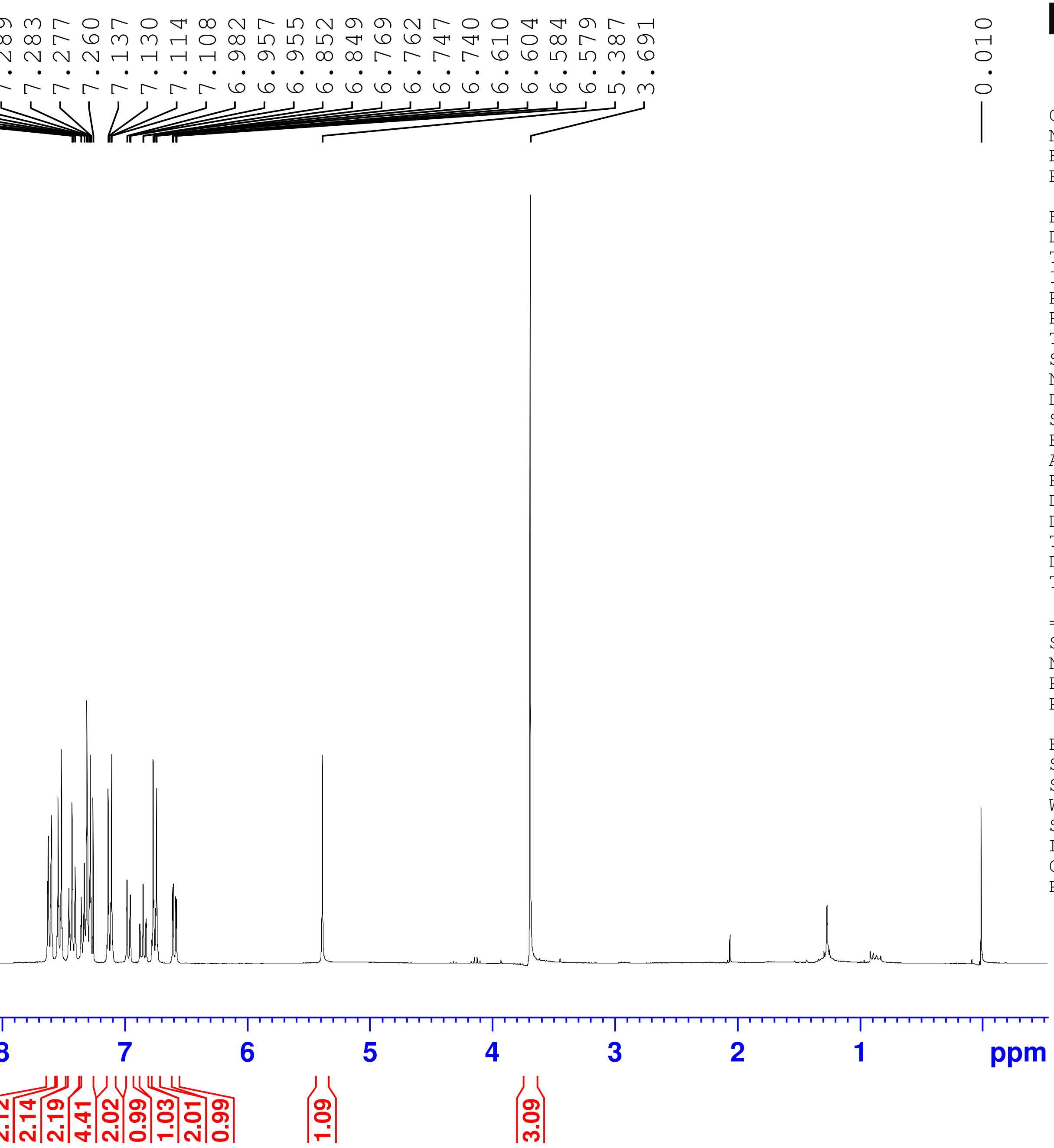

Current Data Parameters
NAME 0907sjw
EXPNO 5251
PROCNO 1

F2 - Acquisition Parameters
Date_ 20210907
Time 13.42
INSTRUM spect
PROBHD 5 mm PABBO BB-
PULPROG zg30
TD 65536
SOLVENT CDCl3
NS 16
DS 2
SWH 6009.615 Hz
FIDRES 0.091699 Hz
AQ 5.4525952 sec
RG 181
DW 83.200 usec
DE 6.50 usec
TE -59.1 K
D1 1.00000000 sec
TD0 1

======== CHANNEL f1 ========
SFO1 300.1318534 MHz
NUC1 1H
P1 10.00 usec
PLW1 14.00000000 W

F2 - Processing parameters
SI 65536
SF 300.1300071 MHz
WDW EM
SSB 0
LB 0.30 Hz
GB 0
PC 1.00

1e

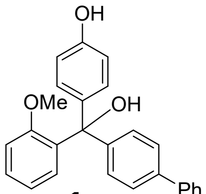

1e

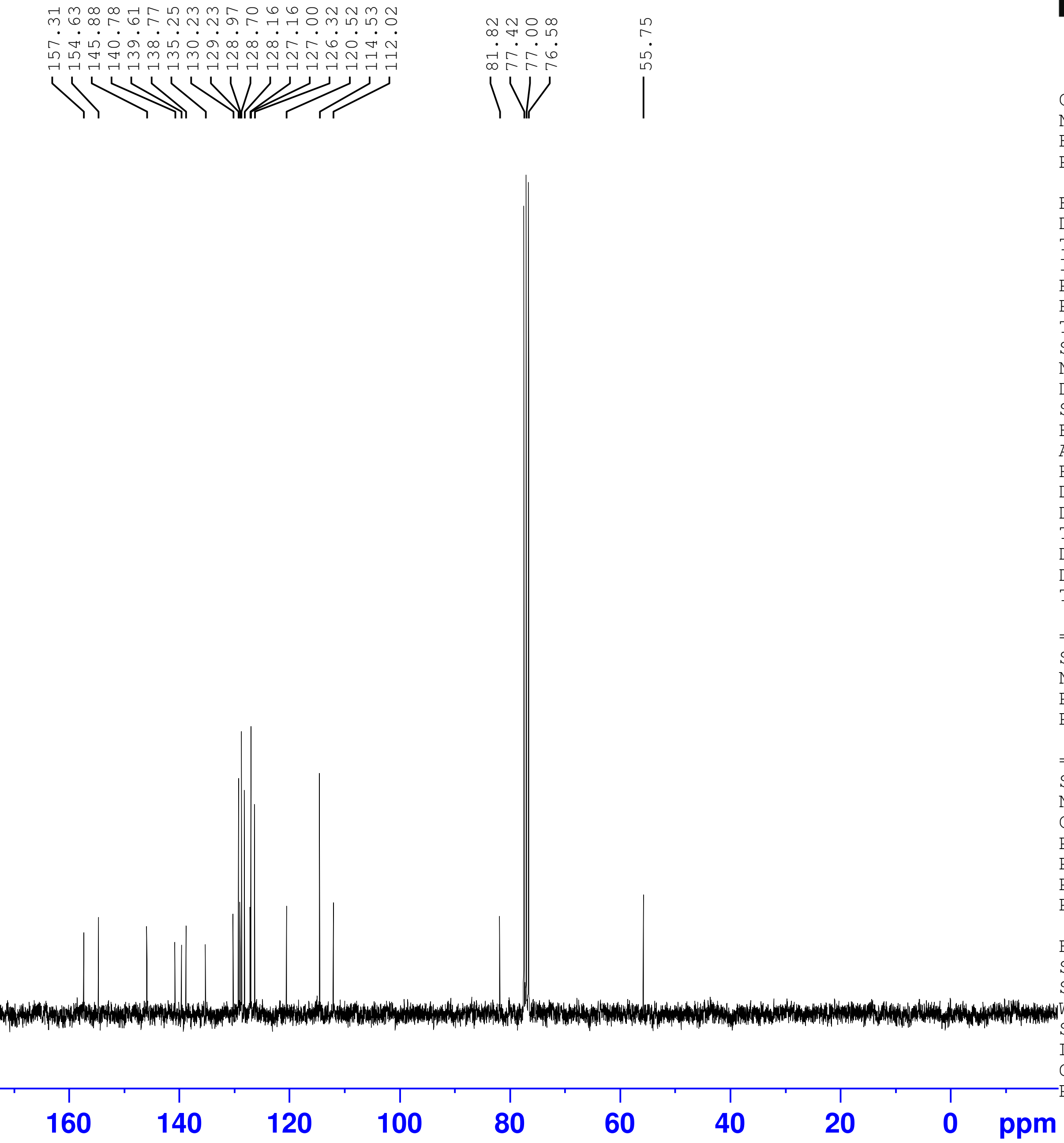


```
Current Data Parameters
NAME           1e-ZY-4-71B
EXPNO                 5264
PROCNO                   1

F2 - Acquisition Parameters
Date_             20210908
Time                 11.30
INSTRUM              spect
PROBHD   5 mm PABBO BB-
PULPROG            zgpg30
TD                   65536
SOLVENT              CDCl3
NS                     350
DS                       4
SWH              18028.846 Hz
FIDRES            0.275098 Hz
AQ               1.8175317 sec
RG                     203
DW                  27.733 usec
DE                    6.50 usec
TE                   -59.1 K
D1              2.00000000 sec
D11             0.03000000 sec
TD0                      1

======== CHANNEL f1 ========
SFO1            75.4752949 MHz
NUC1                   13C
P1                    9.50 usec
PLW1           34.20000076 W

======== CHANNEL f2 ========
SFO2           300.1312005 MHz
NUC2                    1H
CPDPRG[2           waltz16
PCPD2                90.00 usec
PLW2           14.00000000 W
PLW12           0.17284000 W
PLW13           0.14000000 W

F2 - Processing parameters
SI                   32768
SF              75.4677523 MHz
WDW                     EM
SSB                      0
LB                    1.00 Hz
GB                       0
PC                    1.40
```

1j

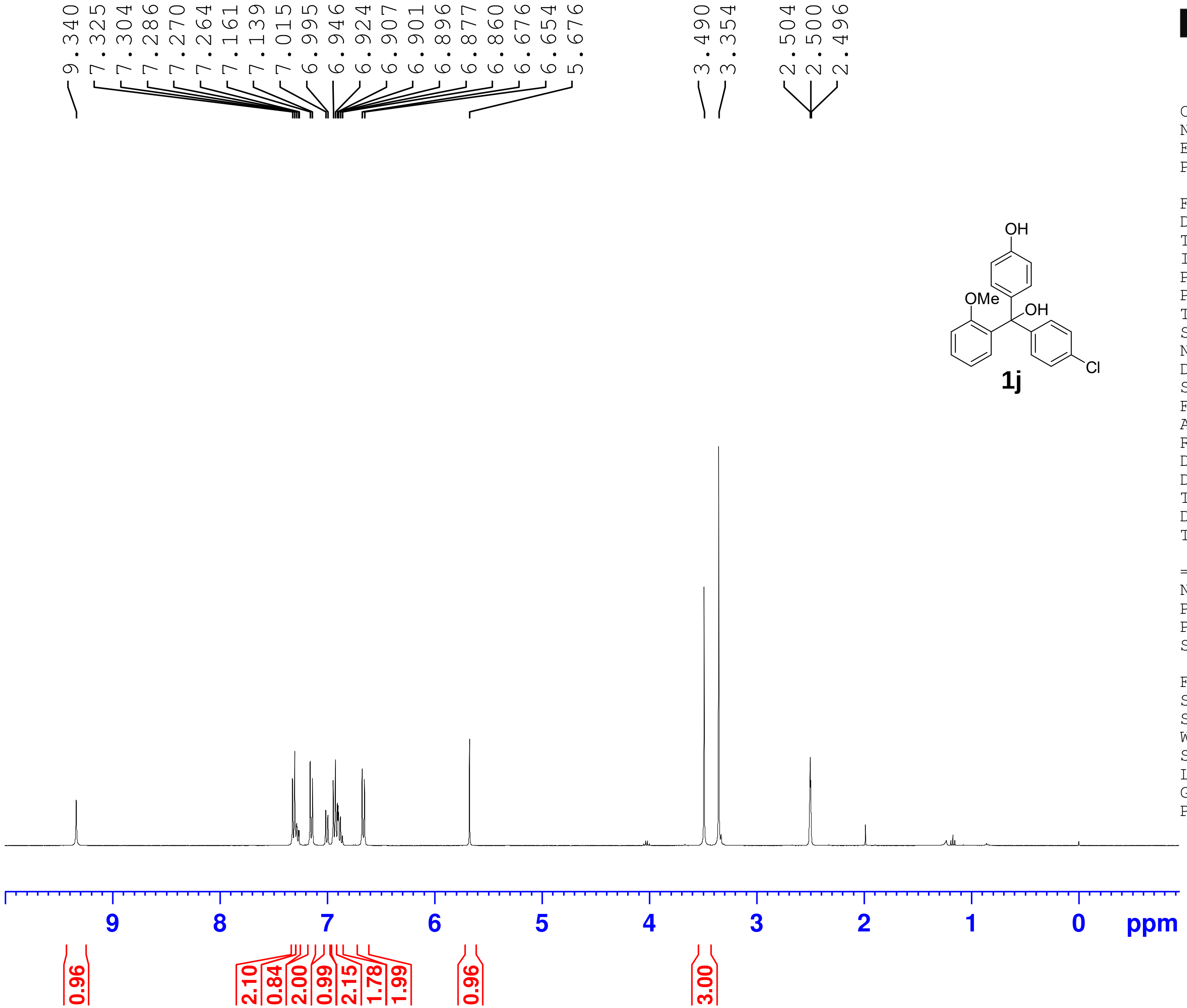

1j

Current Data Parameters
NAME 0720-400
EXPNO 39
PROCNO 1

F2 - Acquisition Parameters
Date_ 20220720
Time 17.01
INSTRUM spect
PROBHD 5 mm PABBO BB-
PULPROG zg30
TD 65536
SOLVENT DMSO
NS 8
DS 2
SWH 8223.685 Hz
FIDRES 0.125483 Hz
AQ 3.9845889 sec
RG 154.68
DW 60.800 usec
DE 6.50 usec
TE 294.4 K
D1 1.00000000 sec
TD0 1

======== CHANNEL f1 ========
NUC1 1H
P1 14.68 usec
PLW1 14.00000000 W
SFO1 400.1924713 MHz

F2 - Processing parameters
SI 65536
SF 400.1900123 MHz
WDW EM
SSB 0
LB 0.30 Hz
GB 0
PC 1.00

1j

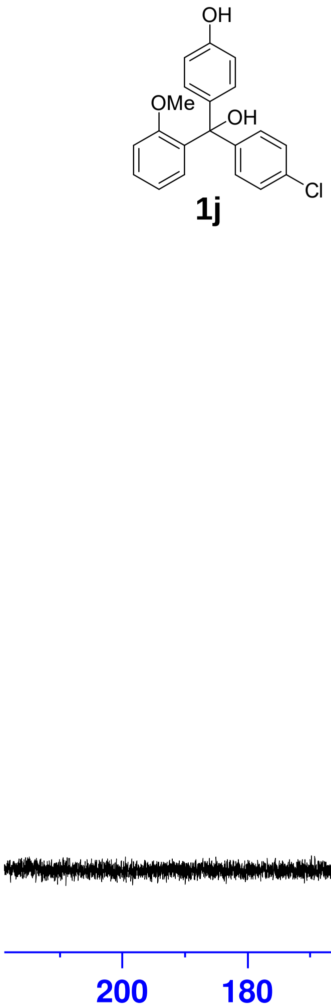

1j

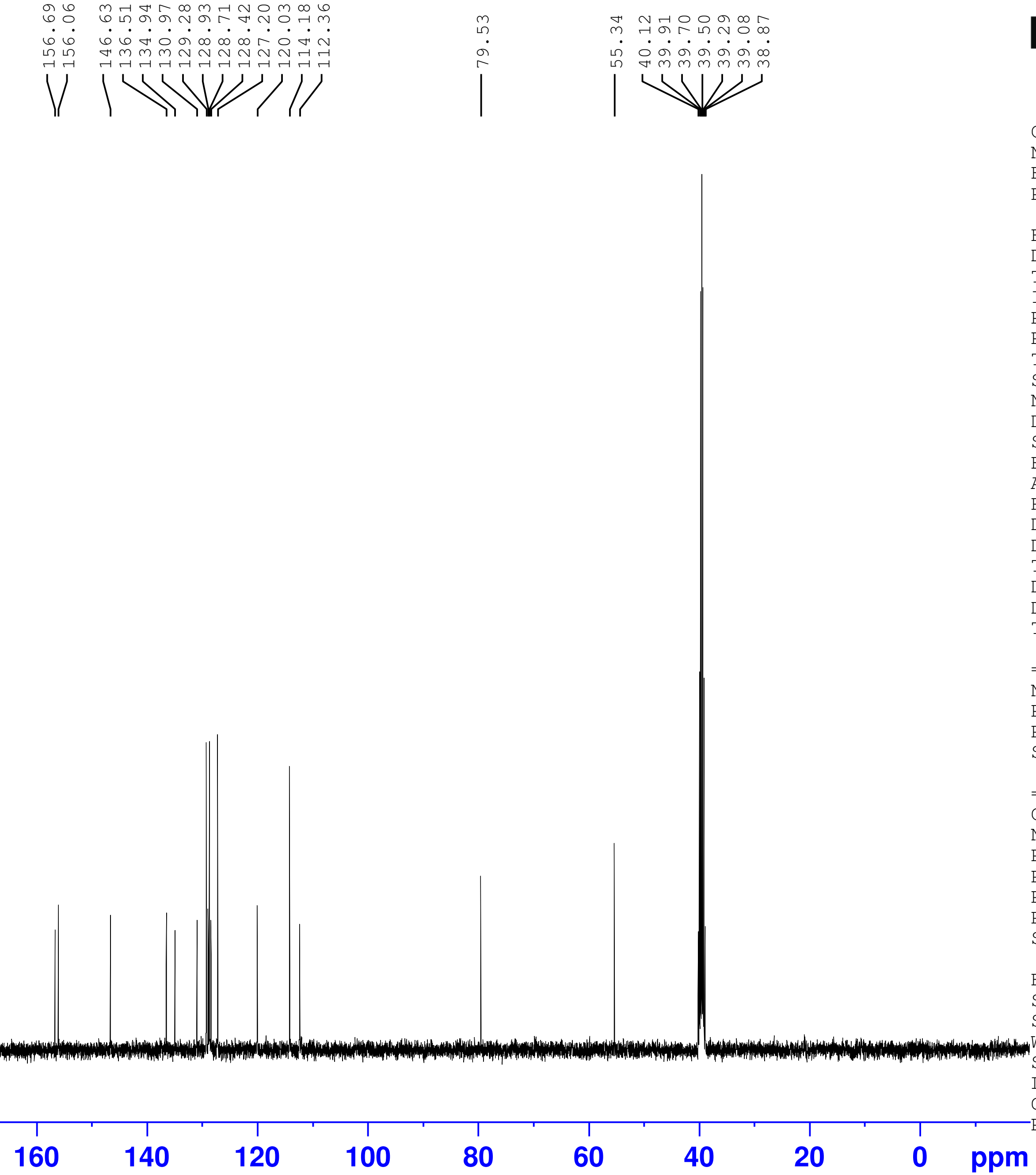

Current Data Parameters
NAME 1j-ZY-4-70A
EXPNO 1
PROCNO 1

F2 - Acquisition Parameters
Date_ 20220718
Time 15.42
INSTRUM spect
PROBHD 5 mm PABBO BB-
PULPROG zgpg30
TD 65536
SOLVENT DMSO
NS 50
DS 4
SWH 24038.461 Hz
FIDRES 0.366798 Hz
AQ 1.3631488 sec
RG 193.13
DW 20.800 usec
DE 6.50 usec
TE 295.1 K
D1 2.00000000 sec
D11 0.03000000 sec
TD0 1

======== CHANNEL f1 ========
NUC1 13C
P1 12.00 usec
PLW1 53.00000000 W
SFO1 100.6379178 MHz

======== CHANNEL f2 ========
CPDPRG[2 waltz16
NUC2 1H
PCPD2 90.00 usec
PLW2 14.00000000 W
PLW12 0.37246999 W
PLW13 0.30170000 W
SFO2 400.1916008 MHz

F2 - Processing parameters
SI 32768
SF 100.6279052 MHz
WDW EM
SSB 0
LB 1.00 Hz
GB 0
PC 1.40

1k

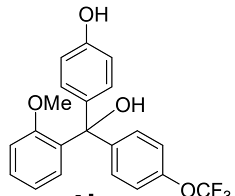

1k

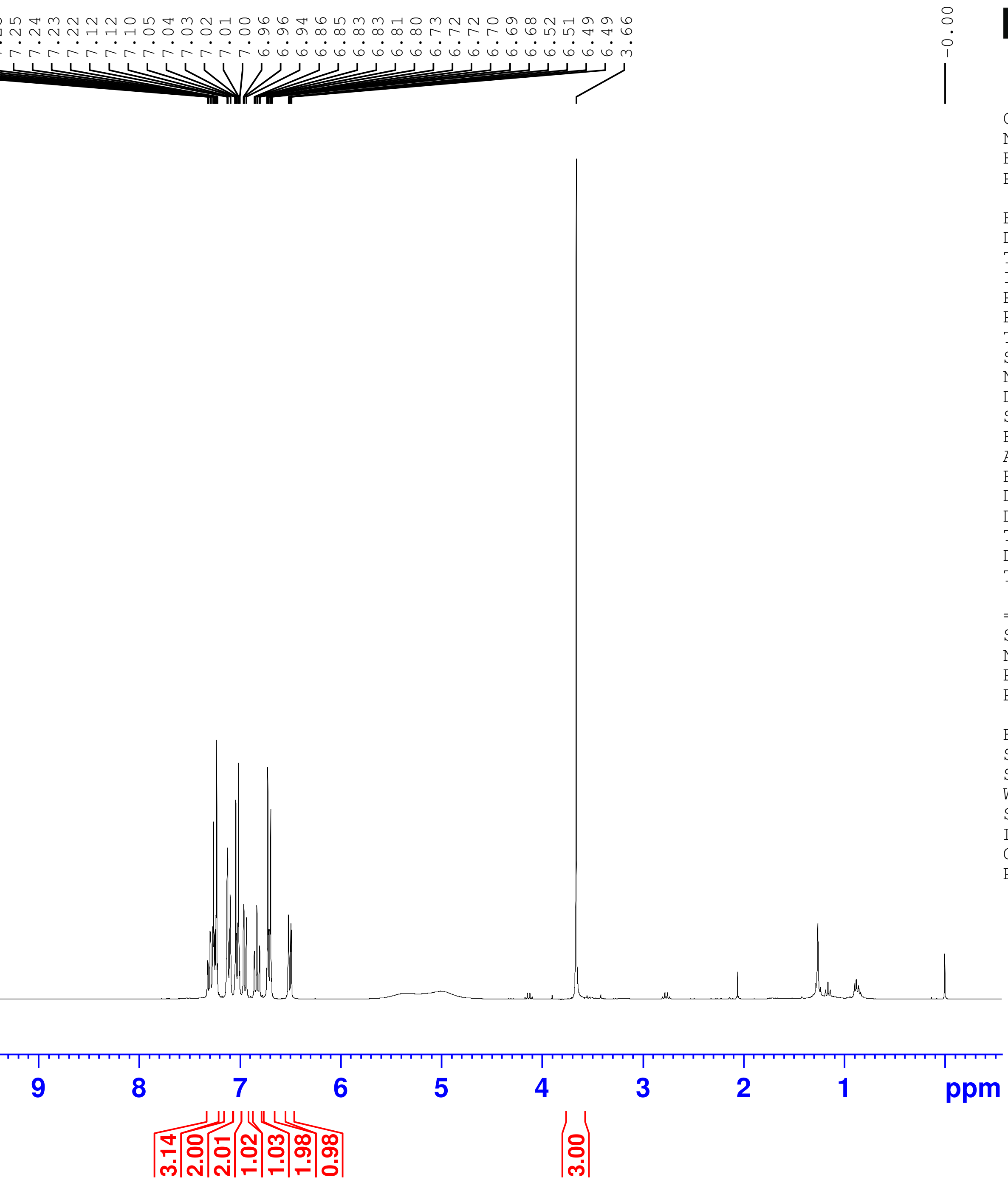

Current Data Parameters
NAME ZY-4-71A-h-fr
EXPNO 5248
PROCNO 1

F2 - Acquisition Parameters
Date_ 20210907
Time 13.34
INSTRUM spect
PROBHD 5 mm PABBO BB-
PULPROG zg30
TD 65536
SOLVENT CDCl3
NS 16
DS 2
SWH 6009.615 Hz
FIDRES 0.091699 Hz
AQ 5.4525952 sec
RG 90.5
DW 83.200 usec
DE 6.50 usec
TE -59.1 K
D1 1.00000000 sec
TD0 1

======== CHANNEL f1 ========
SFO1 300.1318534 MHz
NUC1 1H
P1 10.00 usec
PLW1 14.00000000 W

F2 - Processing parameters
SI 65536
SF 300.1300115 MHz
WDW EM
SSB 0
LB 0.30 Hz
GB 0
PC 1.00

1k

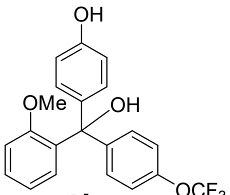

1k

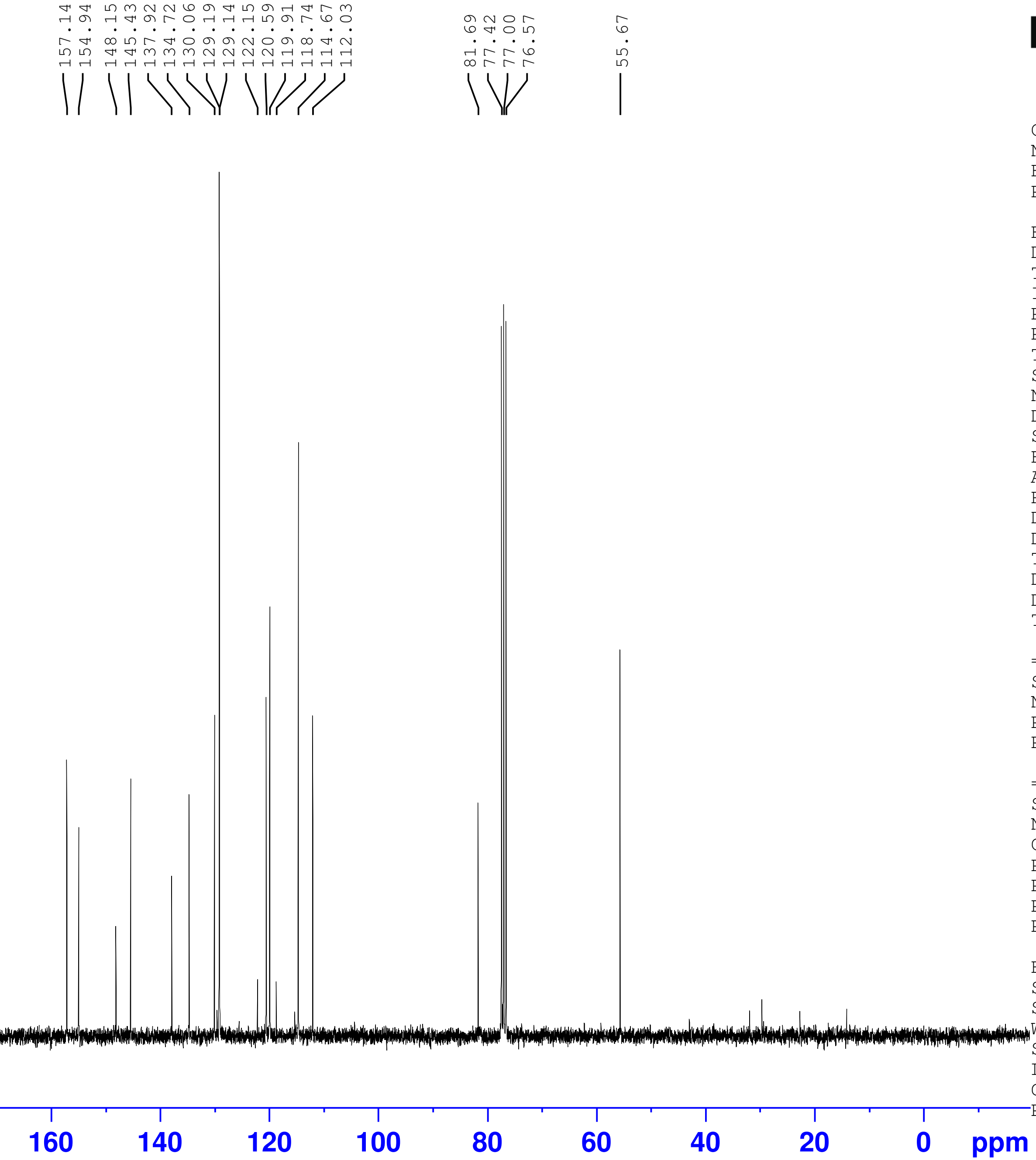

Current Data Parameters
NAME 1k-ZY-4-71A
EXPNO 5263
PROCNO 1

F2 - Acquisition Parameters
Date_ 20210908
Time 11.04
INSTRUM spect
PROBHD 5 mm PABBO BB-
PULPROG zgpg30
TD 65536
SOLVENT CDCl3
NS 350
DS 4
SWH 18028.846 Hz
FIDRES 0.275098 Hz
AQ 1.8175317 sec
RG 203
DW 27.733 usec
DE 6.50 usec
TE -59.1 K
D1 2.00000000 sec
D11 0.03000000 sec
TD0 1

======== CHANNEL f1 ========
SFO1 75.4752949 MHz
NUC1 13C
P1 9.50 usec
PLW1 34.20000076 W

======== CHANNEL f2 ========
SFO2 300.1312005 MHz
NUC2 1H
CPDPRG[2 waltz16
PCPD2 90.00 usec
PLW2 14.00000000 W
PLW12 0.17284000 W
PLW13 0.14000000 W

F2 - Processing parameters
SI 32768
SF 75.4677535 MHz
WDW EM
SSB 0
LB 1.00 Hz
GB 0
PC 1.40

1k

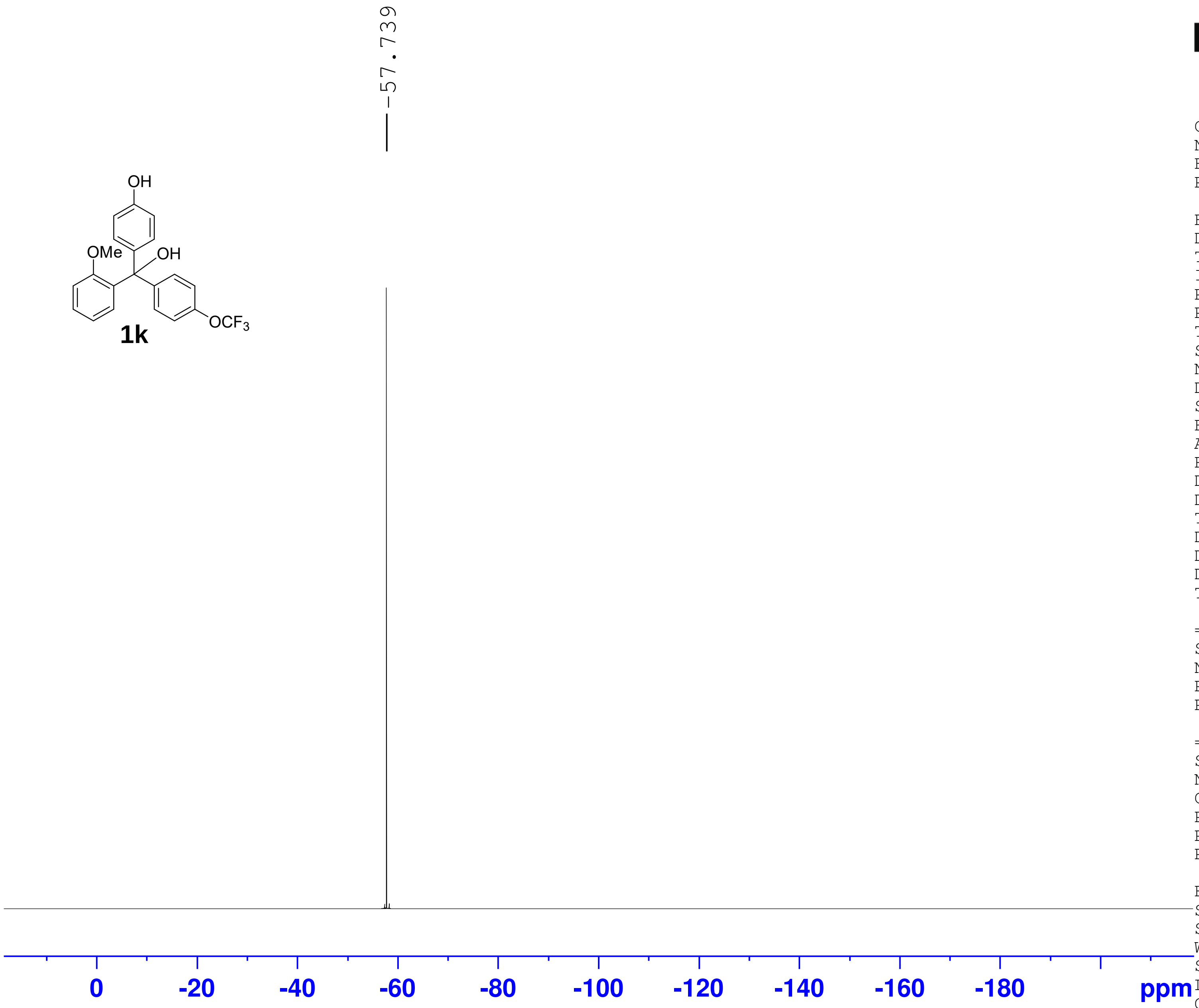

Current Data Parameters
NAME 0907sjw
EXPNO 5249
PROCNO 1

F2 - Acquisition Parameters
Date_ 20210907
Time 13.37
INSTRUM spect
PROBHD 5 mm PABBO BB-
PULPROG zgfhigqn.2
TD 131072
SOLVENT CDCl3
NS 16
DS 4
SWH 66964.289 Hz
FIDRES 0.510897 Hz
AQ 0.9786710 sec
RG 203
DW 7.467 usec
DE 6.50 usec
TE -59.1 K
D1 1.00000000 sec
D11 0.03000000 sec
D12 0.00002000 sec
TD0 1

======== CHANNEL f1 ========
SFO1 282.3761148 MHz
NUC1 19F
P1 14.50 usec
PLW1 10.39999962 W

======== CHANNEL f2 ========
SFO2 300.1312005 MHz
NUC2 1H
CPDPRG[2 waltz16
PCPD2 90.00 usec
PLW2 14.00000000 W
PLW12 0.17284000 W

F2 - Processing parameters
SI 65536
SF 282.4043552 MHz
WDW EM
SSB 0
LB 0.30 Hz
GB 0
PC 1.00

1l

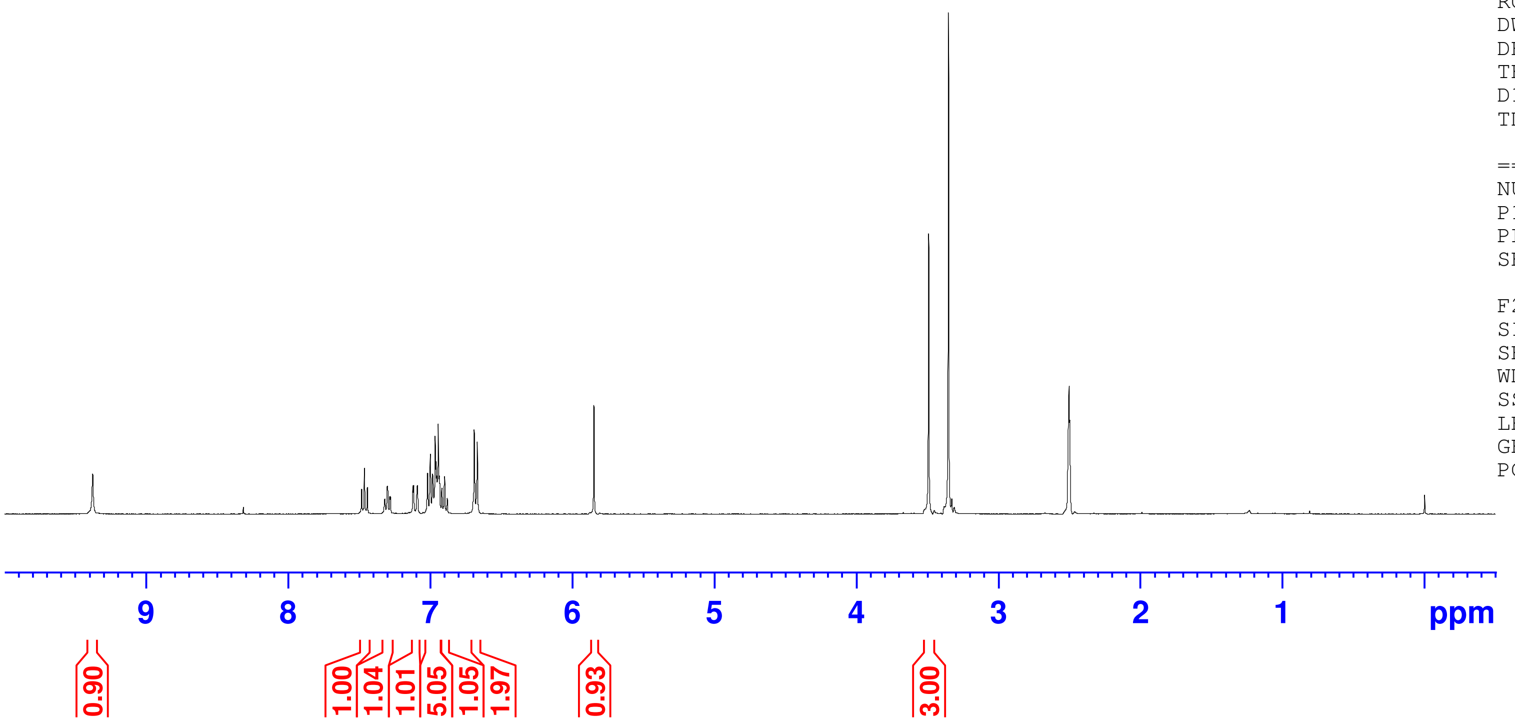

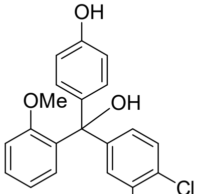

1l

Current Data Parameters

NAME	0729-400
EXPNO	110
PROCNO	1

F2 - Acquisition Parameters

Date_	20220729
Time	21.44
INSTRUM	spect
PROBHD	5 mm PABBO BB-
PULPROG	zg30
TD	65536
SOLVENT	DMSO
NS	8
DS	2
SWH	8223.685 Hz
FIDRES	0.125483 Hz
AQ	3.9845889 sec
RG	181.41
DW	60.800 usec
DE	6.50 usec
TE	294.7 K
D1	1.00000000 sec
TD0	1

======== CHANNEL f1 ========

NUC1	1H
P1	14.68 usec
PLW1	14.00000000 W
SFO1	400.1924713 MHz

F2 - Processing parameters

SI	65536
SF	400.1900124 MHz
WDW	EM
SSB	0
LB	0.30 Hz
GB	0
PC	1.00

1l

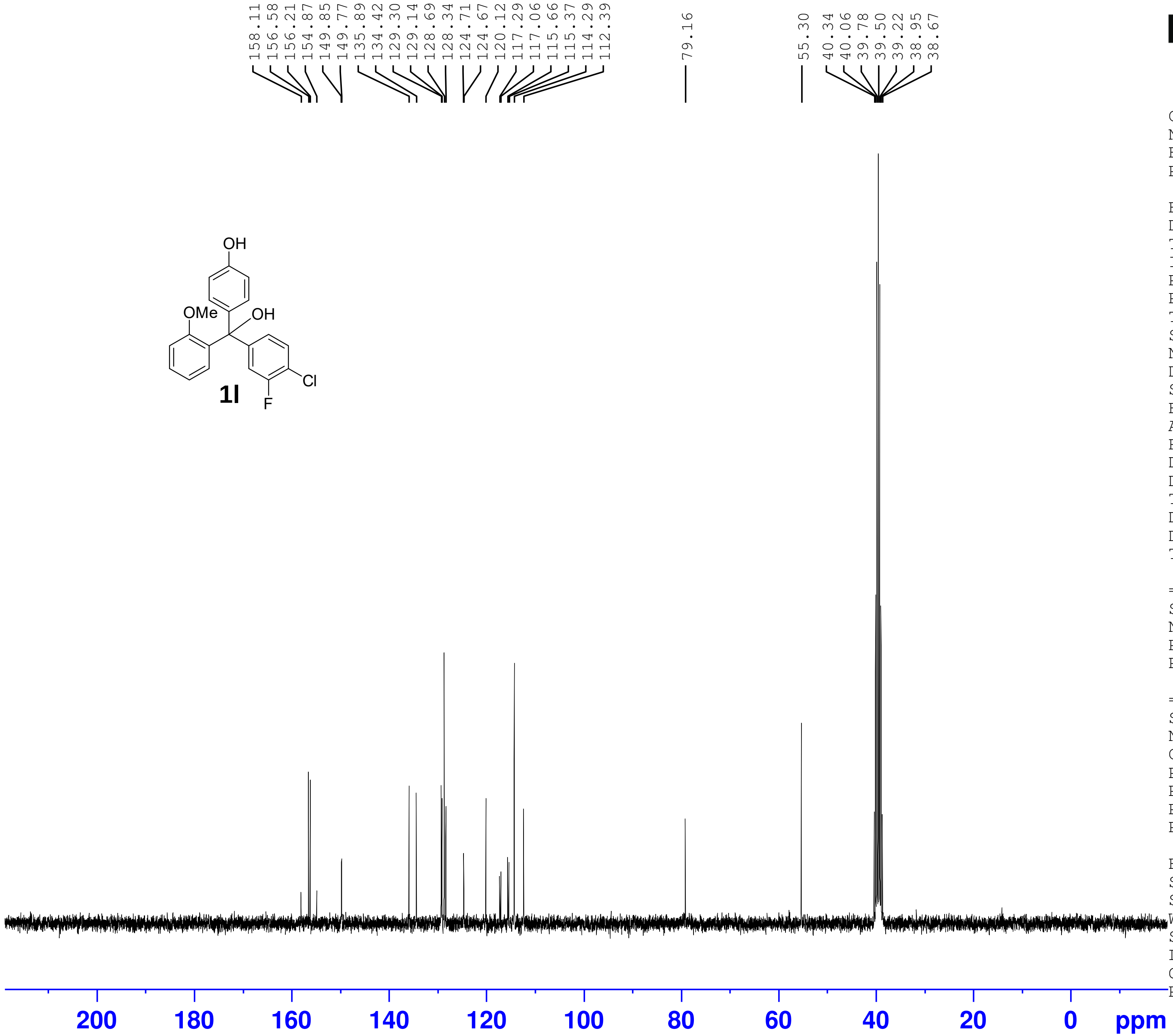


```
Current Data Parameters
NAME           1l-ZY-4-78B
EXPNO                 5462
PROCNO                   1

F2 - Acquisition Parameters
Date_             20210927
Time                 13.31
INSTRUM              spect
PROBHD    5 mm PABBO BB-
PULPROG            zgpg30
TD                   65536
SOLVENT               DMSO
NS                     138
DS                       4
SWH              18028.846 Hz
FIDRES            0.275098 Hz
AQ               1.8175317 sec
RG                     203
DW                  27.733 usec
DE                    6.50 usec
TE                   -59.1 K
D1              2.00000000 sec
D11             0.03000000 sec
TD0                      1

======== CHANNEL f1 ========
SFO1           75.4752949 MHz
NUC1                   13C
P1                    9.50 usec
PLW1           34.20000076 W

======== CHANNEL f2 ========
SFO2          300.1312005 MHz
NUC2                    1H
CPDPRG[2           waltz16
PCPD2                90.00 usec
PLW2           14.00000000 W
PLW12           0.17284000 W
PLW13           0.14000000 W

F2 - Processing parameters
SI                   32768
SF             75.4677831 MHz
WDW                     EM
SSB                      0
LB                    1.00 Hz
GB                       0
PC                    1.40
```

1l

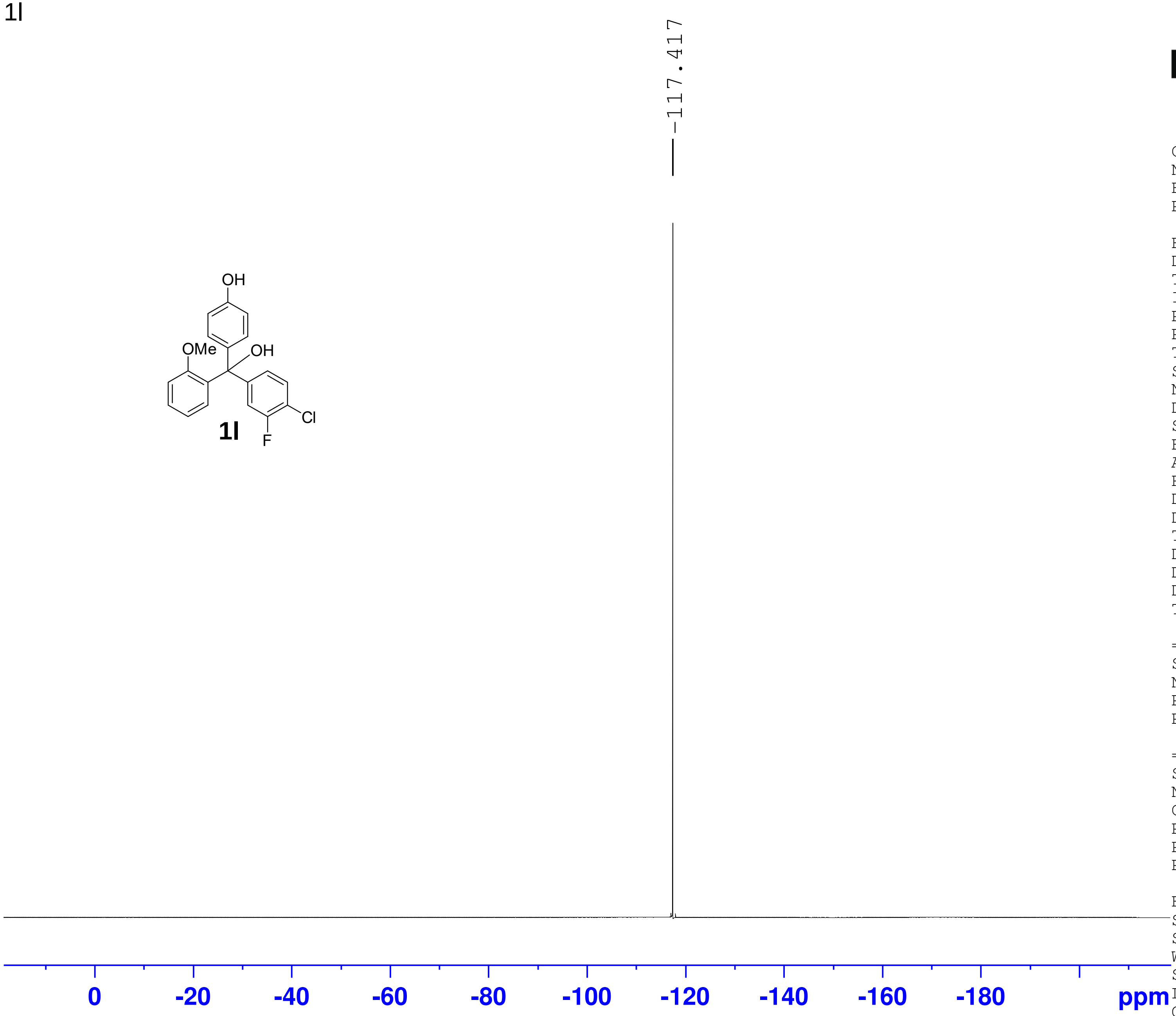

BRUKER

```
Current Data Parameters
NAME              0917sjw
EXPNO                5382
PROCNO                  1

F2 - Acquisition Parameters
Date_            20210917
Time                12.47
INSTRUM             spect
PROBHD    5 mm PABBO BB-
PULPROG        zgfhigqn.2
TD                 131072
SOLVENT              DMSO
NS                     16
DS                      4
SWH             66964.289 Hz
FIDRES           0.510897 Hz
AQ              0.9786710 sec
RG                    203
DW                  7.467 usec
DE                   6.50 usec
TE                  -59.1 K
D1             1.00000000 sec
D11            0.03000000 sec
D12            0.00002000 sec
TD0                     1

======== CHANNEL f1 ========
SFO1          282.3761148 MHz
NUC1                  19F
P1                  14.50 usec
PLW1          10.39999962 W

======== CHANNEL f2 ========
SFO2          300.1312005 MHz
NUC2                   1H
CPDPRG[2          waltz16
PCPD2               90.00 usec
PLW2          14.00000000 W
PLW12          0.17284000 W

F2 - Processing parameters
SI                  65536
SF            282.4043552 MHz
WDW                    EM
SSB                     0
LB                   0.30 Hz
GB                      0
PC                   1.00
```

1m

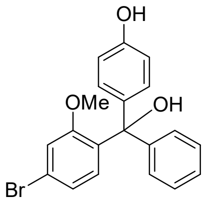

1m

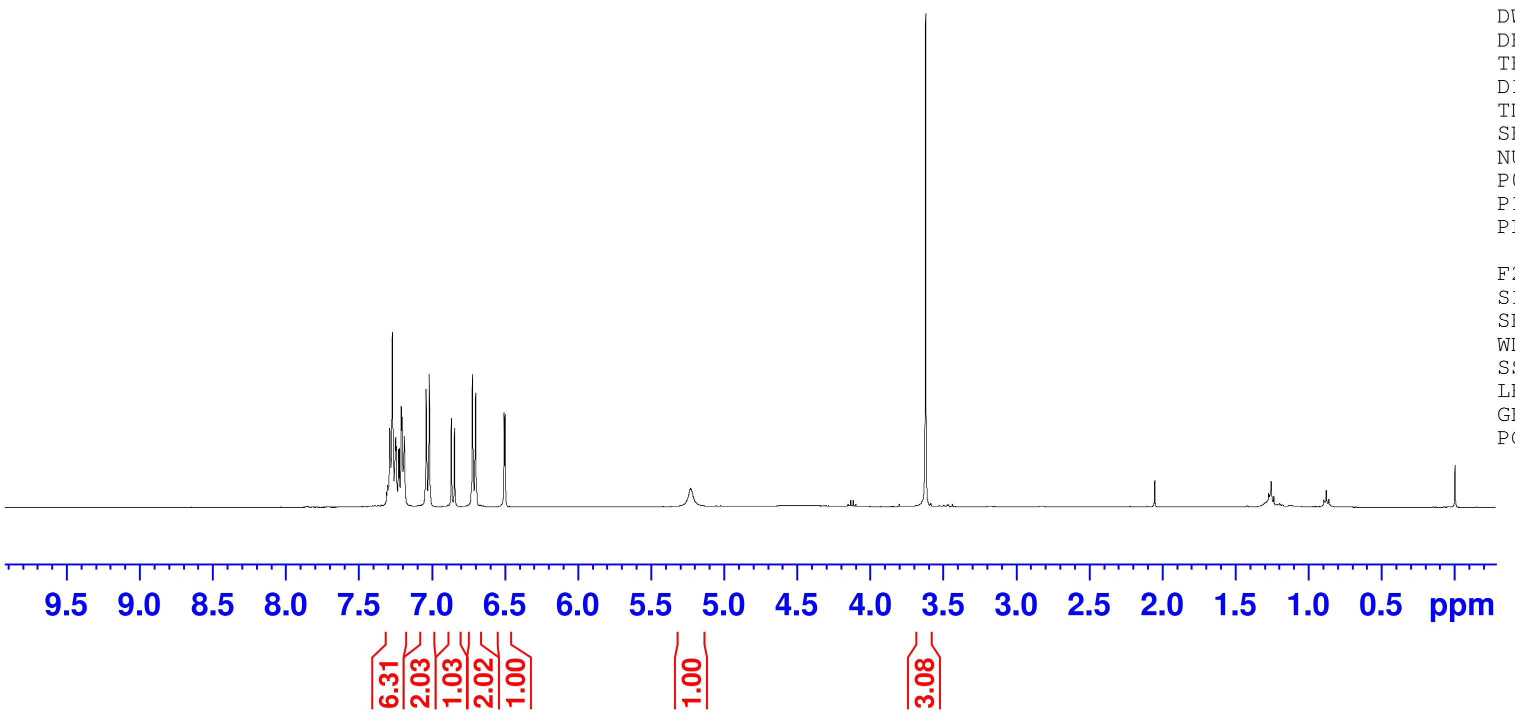

Current Data Parameters

NAME	0611HH
EXPNO	16
PROCNO	1

F2 - Acquisition Parameters

Date_	20220611
Time	4.44 h
INSTRUM	Avance
PROBHD	Z116098_0833 (
PULPROG	zg30
TD	65536
SOLVENT	CDCl3
NS	16
DS	2
SWH	8196.722 Hz
FIDRES	0.250144 Hz
AQ	3.9976959 sec
RG	101
DW	61.000 usec
DE	13.54 usec
TE	292.4 K
D1	1.00000000 sec
TD0	1
SFO1	400.1324708 MHz
NUC1	1H
P0	3.33 usec
P1	10.00 usec
PLW1	20.73200035 W

F2 - Processing parameters

SI	65536
SF	400.1300172 MHz
WDW	EM
SSB	0
LB	0.30 Hz
GB	0
PC	1.00

1m

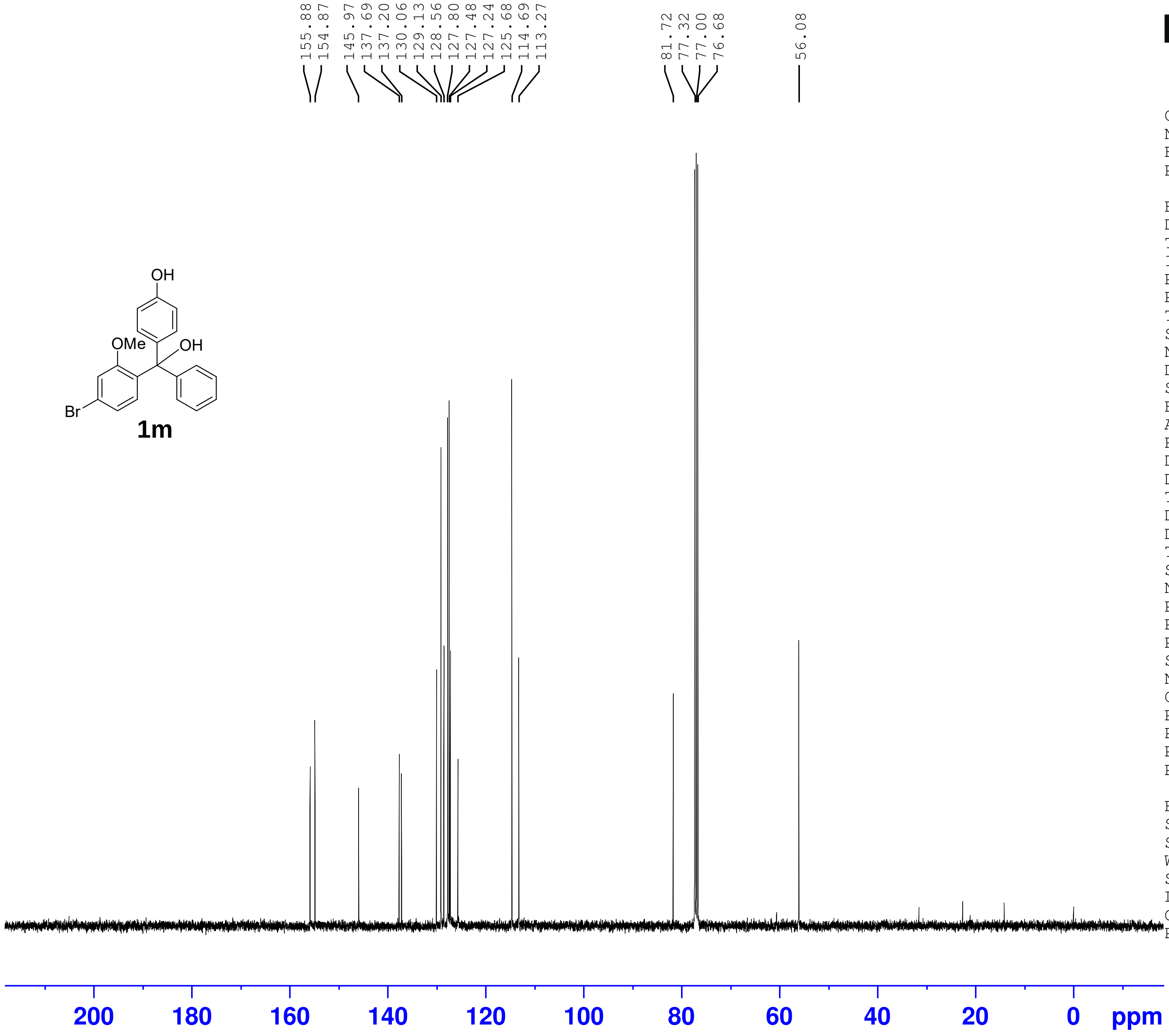

BRUKER

Current Data Parameters
NAME 1m-ZY-4-82
EXPNO 15
PROCNO 1

F2 - Acquisition Parameters
Date_ 20220611
Time 4.42 h
INSTRUM Avance
PROBHD Z116098_0833 (
PULPROG zgpg30
TD 65536
SOLVENT CDCl3
NS 400
DS 4
SWH 23809.523 Hz
FIDRES 0.726609 Hz
AQ 1.3762560 sec
RG 54.9866
DW 21.000 usec
DE 6.50 usec
TE 292.8 K
D1 2.00000000 sec
D11 0.03000000 sec
TD0 1
SFO1 100.6228298 MHz
NUC1 13C
P0 3.33 usec
P1 10.00 usec
PLW1 87.89900208 W
SFO2 400.1316005 MHz
NUC2 1H
CPDPRG[2 waltz65
PCPD2 90.00 usec
PLW2 20.73200035 W
PLW12 0.25595000 W
PLW13 0.12874000 W

F2 - Processing parameters
SI 32768
SF 100.6127766 MHz
WDW EM
SSB 0
LB 1.00 Hz
GB 0
PC 1.40

1n

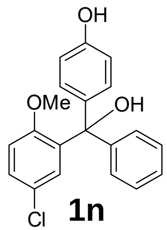

1n

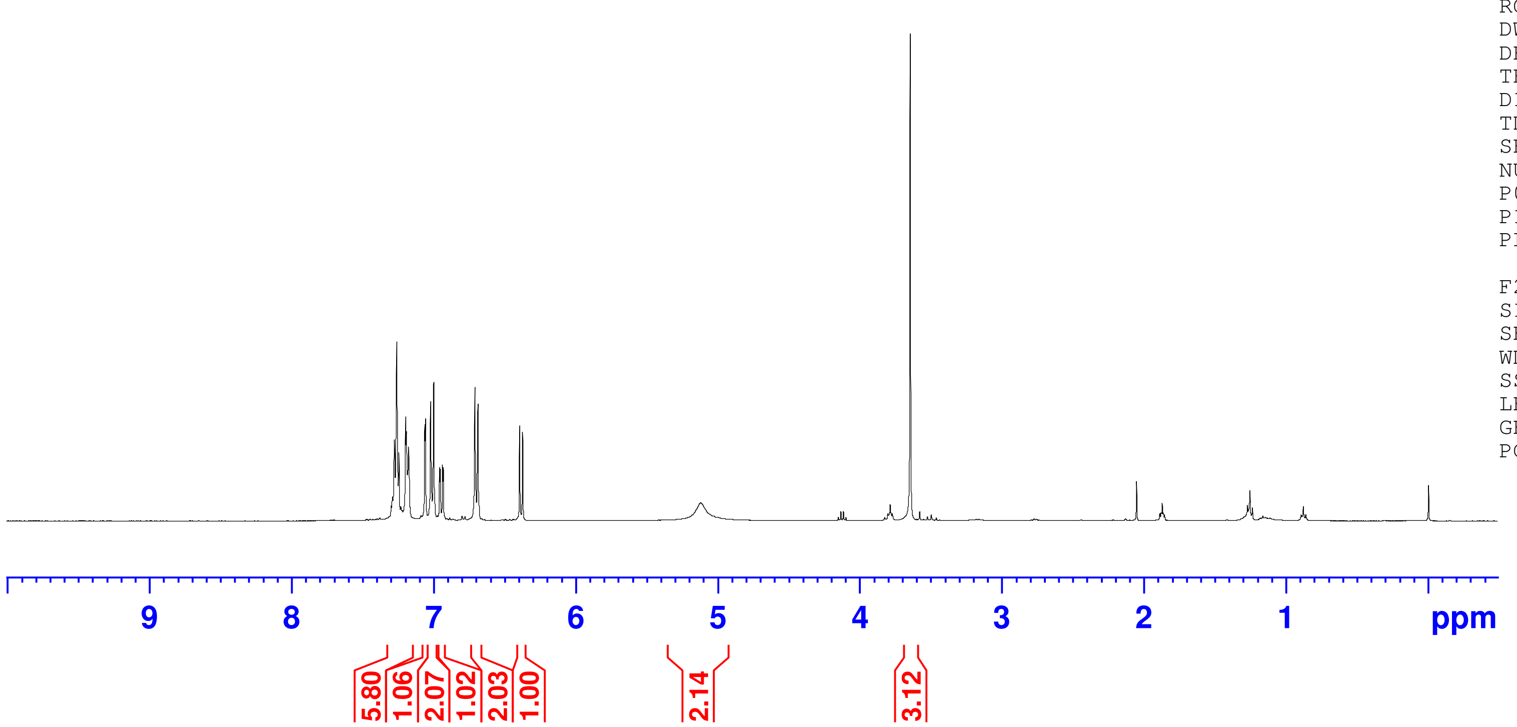

Current Data Parameters
NAME 0611HH
EXPNO 14
PROCNO 1

F2 - Acquisition Parameters
Date_ 20220611
Time 4.14 h
INSTRUM Avance
PROBHD Z116098_0833 (
PULPROG zg30
TD 65536
SOLVENT CDCl3
NS 16
DS 2
SWH 8196.722 Hz
FIDRES 0.250144 Hz
AQ 3.9976959 sec
RG 82.3452
DW 61.000 usec
DE 13.54 usec
TE 292.2 K
D1 1.00000000 sec
TD0 1
SFO1 400.1324708 MHz
NUC1 1H
P0 3.33 usec
P1 10.00 usec
PLW1 20.73200035 W

F2 - Processing parameters
SI 65536
SF 400.1300163 MHz
WDW EM
SSB 0
LB 0.30 Hz
GB 0
PC 1.00

1n

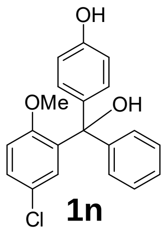

1n

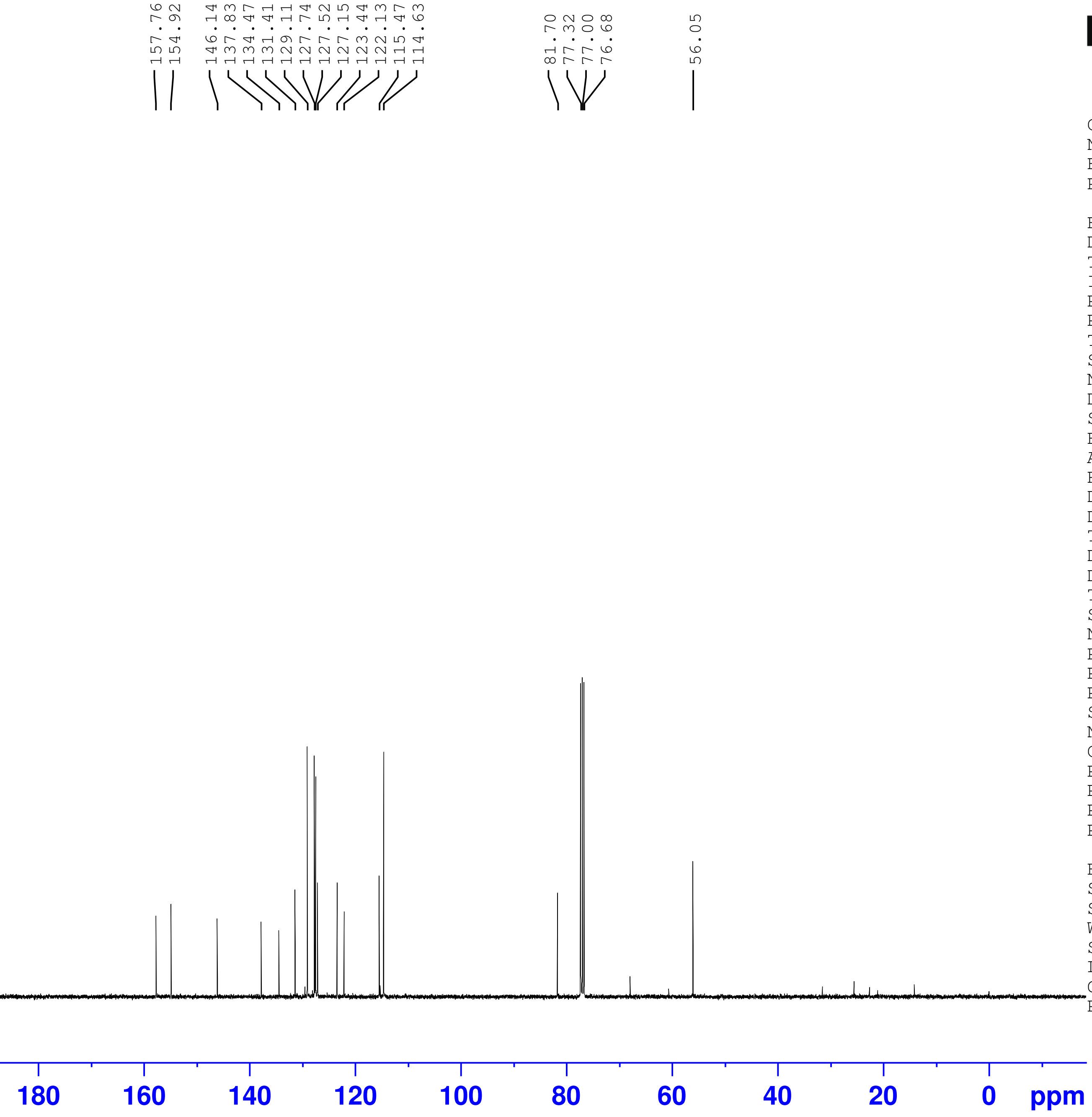

BRUKER

Current Data Parameters
NAME 1n-ZY-4-83
EXPNO 13
PROCNO 1

F2 - Acquisition Parameters
Date_ 20220611
Time 4.12 h
INSTRUM Avance
PROBHD Z116098_0833 (
PULPROG zgpg30
TD 65536
SOLVENT CDCl3
NS 400
DS 4
SWH 23809.523 Hz
FIDRES 0.726609 Hz
AQ 1.3762560 sec
RG 54.9866
DW 21.000 usec
DE 6.50 usec
TE 292.7 K
D1 2.00000000 sec
D11 0.03000000 sec
TD0 1
SFO1 100.6228298 MHz
NUC1 13C
P0 3.33 usec
P1 10.00 usec
PLW1 87.89900208 W
SFO2 400.1316005 MHz
NUC2 1H
CPDPRG[2 waltz65
PCPD2 90.00 usec
PLW2 20.73200035 W
PLW12 0.25595000 W
PLW13 0.12874000 W

F2 - Processing parameters
SI 32768
SF 100.6127772 MHz
WDW EM
SSB 0
LB 1.00 Hz
GB 0
PC 1.40

1o

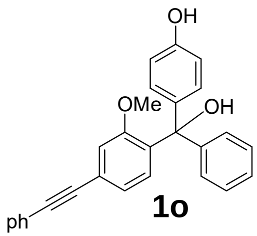

1o

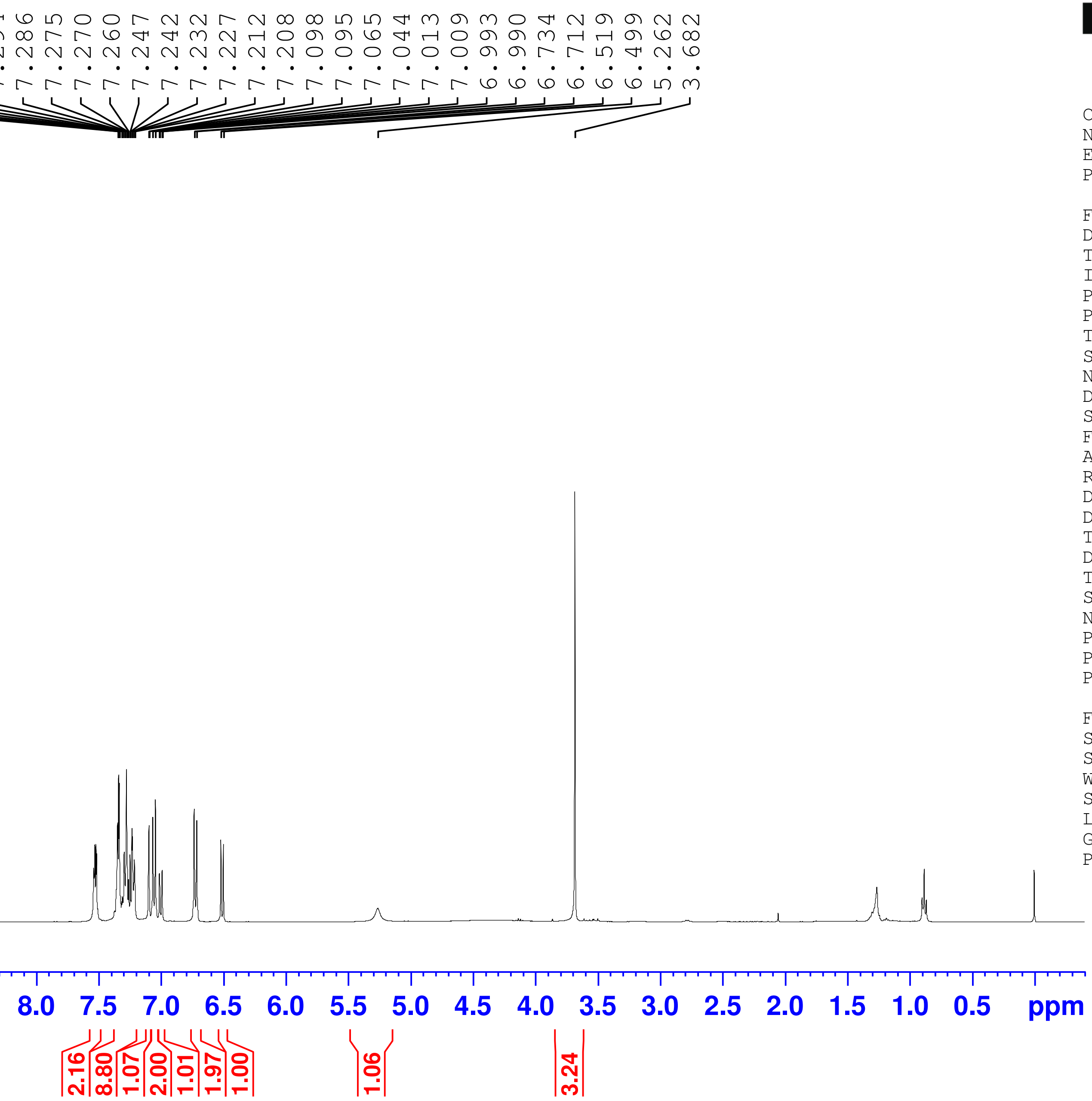

BRUKER

```
Current Data Parameters
NAME                0611HH
EXPNO                   12
PROCNO                   1

F2 - Acquisition Parameters
Date_             20220611
Time                  3.45 h
INSTRUM             Avance
PROBHD    Z116098_0833 (
PULPROG               zg30
TD                   65536
SOLVENT              CDCl3
NS                      16
DS                       2
SWH               8196.722 Hz
FIDRES            0.250144 Hz
AQ               3.9976959 sec
RG                 91.9118
DW                  61.000 usec
DE                   13.54 usec
TE                   292.4 K
D1              1.00000000 sec
TD0                      1
SFO1           400.1324708 MHz
NUC1                    1H
P0                    3.33 usec
P1                   10.00 usec
PLW1           20.73200035 W

F2 - Processing parameters
SI                   65536
SF             400.1300153 MHz
WDW                     EM
SSB                      0
LB                    0.30 Hz
GB                       0
PC                    1.00
```

1o

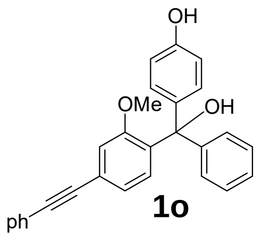

1o

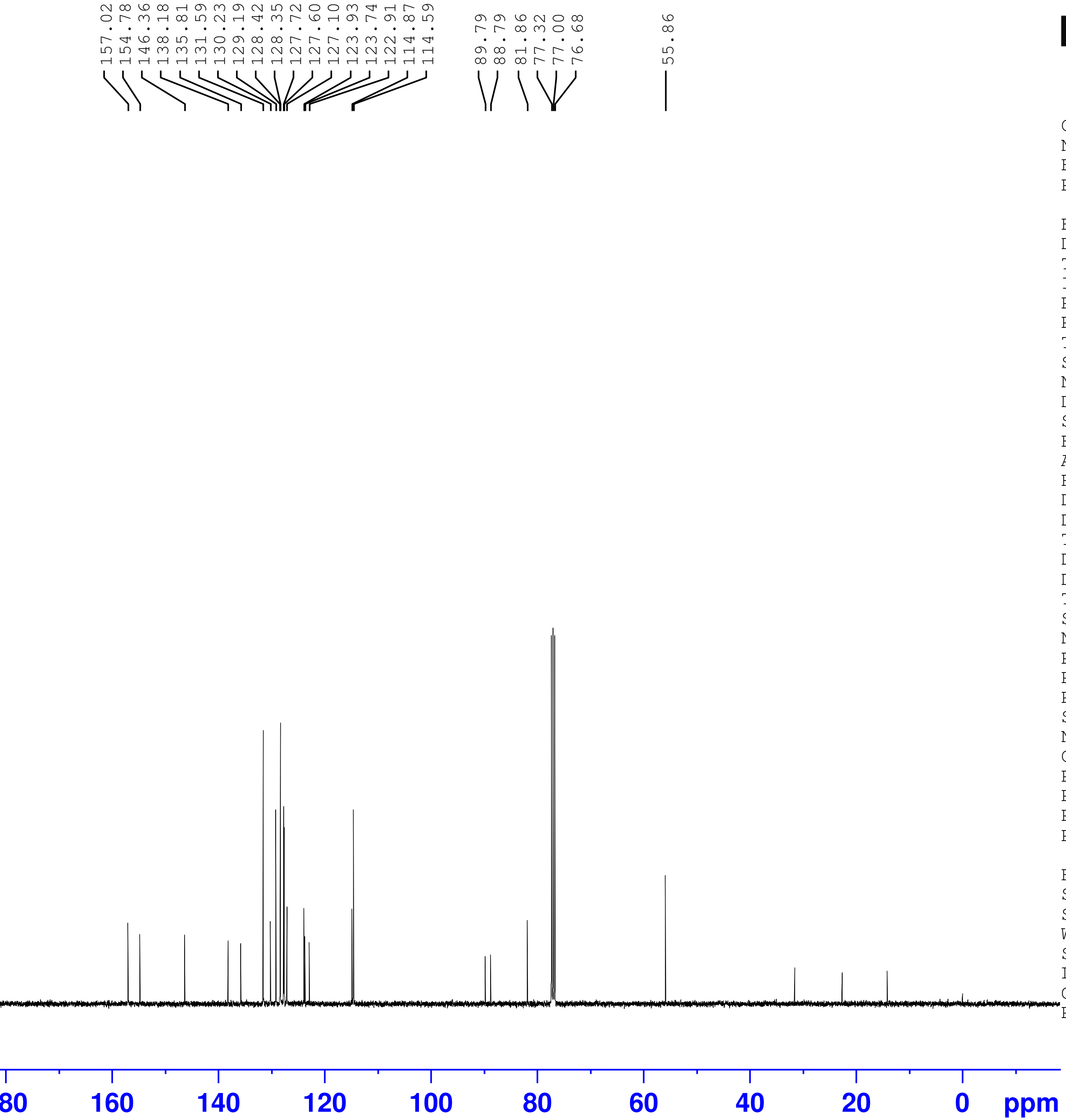

Current Data Parameters
NAME 1o-ZY-4-84
EXPNO 11
PROCNO 1

F2 - Acquisition Parameters
Date_ 20220611
Time 3.43 h
INSTRUM Avance
PROBHD Z116098_0833 (
PULPROG zgpg30
TD 65536
SOLVENT CDCl3
NS 400
DS 4
SWH 23809.523 Hz
FIDRES 0.726609 Hz
AQ 1.3762560 sec
RG 53.2129
DW 21.000 usec
DE 6.50 usec
TE 292.9 K
D1 2.00000000 sec
D11 0.03000000 sec
TD0 1
SFO1 100.6228298 MHz
NUC1 13C
P0 3.33 usec
P1 10.00 usec
PLW1 87.89900208 W
SFO2 400.1316005 MHz
NUC2 1H
CPDPRG[2 waltz65
PCPD2 90.00 usec
PLW2 20.73200035 W
PLW12 0.25595000 W
PLW13 0.12874000 W

F2 - Processing parameters
SI 32768
SF 100.6127763 MHz
WDW EM
SSB 0
LB 1.00 Hz
GB 0
PC 1.40

1q

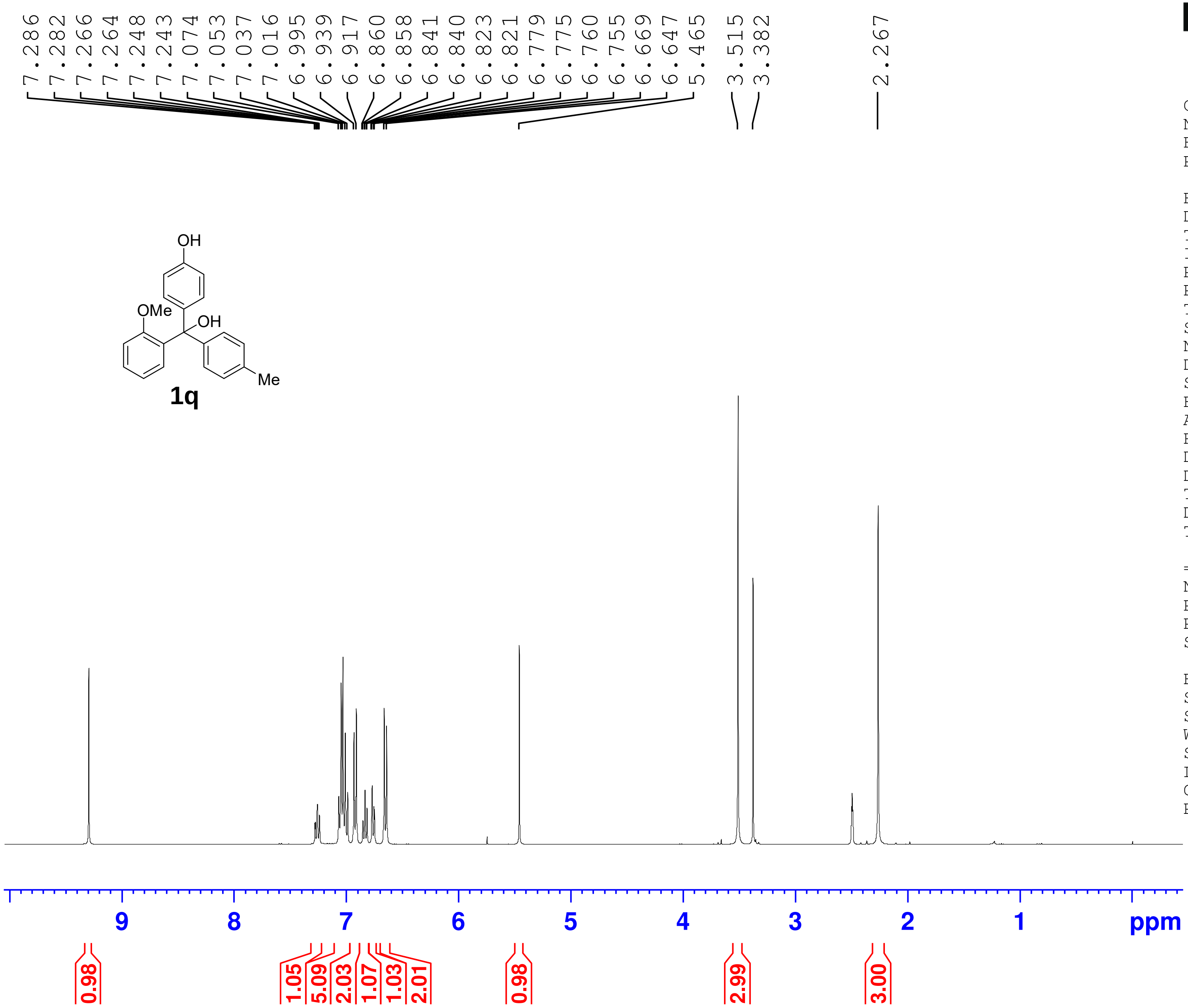

Current Data Parameters

NAME	0718-400
EXPNO	15
PROCNO	1

F2 - Acquisition Parameters

Date_	20220718
Time	15.33
INSTRUM	spect
PROBHD	5 mm PABBO BB-
PULPROG	zg30
TD	65536
SOLVENT	DMSO
NS	6
DS	0
SWH	8223.685 Hz
FIDRES	0.125483 Hz
AQ	3.9845889 sec
RG	75.43
DW	60.800 usec
DE	6.50 usec
TE	294.6 K
D1	1.00000000 sec
TD0	1

======== CHANNEL f1 ========

NUC1	1H
P1	14.68 usec
PLW1	14.00000000 W
SFO1	400.1924713 MHz

F2 - Processing parameters

SI	65536
SF	400.1900135 MHz
WDW	EM
SSB	0
LB	0.30 Hz
GB	0
PC	1.00

1q

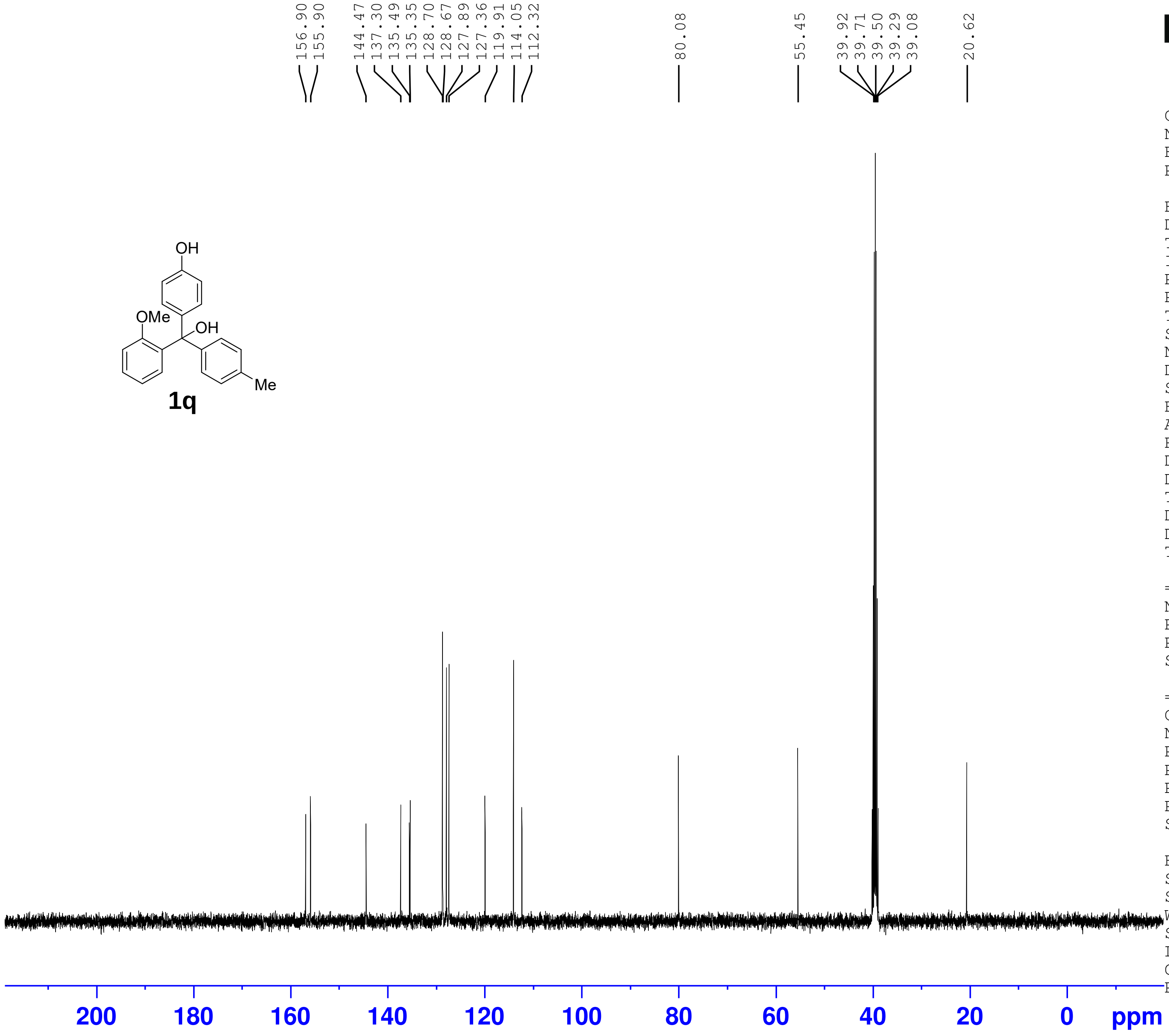

BRUKER

```
Current Data Parameters
NAME          1q-ZY-4-69A
EXPNO                   1
PROCNO                  1

F2 - Acquisition Parameters
Date_            20220718
Time                15.35
INSTRUM             spect
PROBHD   5 mm PABBO BB-
PULPROG           zgpg30
TD                  65536
SOLVENT              DMSO
NS                     50
DS                      4
SWH             24038.461 Hz
FIDRES           0.366798 Hz
AQ              1.3631488 sec
RG                 193.13
DW                 20.800 usec
DE                   6.50 usec
TE                  294.9 K
D1             2.00000000 sec
D11            0.03000000 sec
TD0                     1

======== CHANNEL f1 ========
NUC1                  13C
P1                  12.00 usec
PLW1          53.00000000 W
SFO1          100.6379178 MHz

======== CHANNEL f2 ========
CPDPRG[2          waltz16
NUC2                   1H
PCPD2               90.00 usec
PLW2          14.00000000 W
PLW12          0.37246999 W
PLW13          0.30170000 W
SFO2          400.1916008 MHz

F2 - Processing parameters
SI                  32768
SF            100.6279048 MHz
WDW                    EM
SSB                     0
LB                   1.00 Hz
GB                      0
PC                   1.40
```

1r

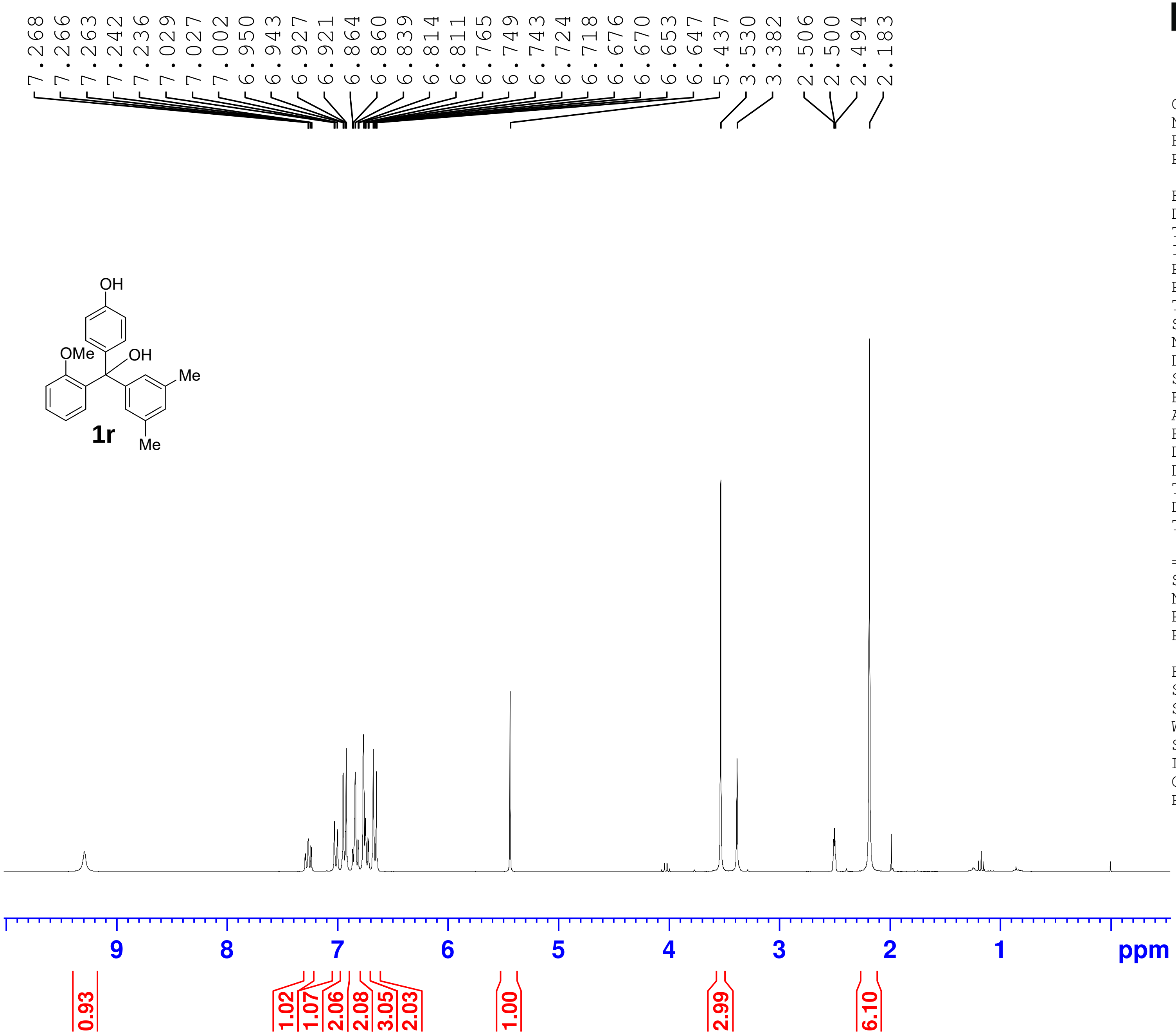

BRUKER

Current Data Parameters
NAME 0917sjw
EXPNO 5380
PROCNO 1

F2 - Acquisition Parameters
Date_ 20210917
Time 12.40
INSTRUM spect
PROBHD 5 mm PABBO BB-
PULPROG zg30
TD 65536
SOLVENT DMSO
NS 16
DS 2
SWH 6009.615 Hz
FIDRES 0.091699 Hz
AQ 5.4525952 sec
RG 80.6
DW 83.200 usec
DE 6.50 usec
TE -59.1 K
D1 1.00000000 sec
TD0 1

======== CHANNEL f1 ========
SFO1 300.1318534 MHz
NUC1 1H
P1 10.00 usec
PLW1 14.00000000 W

F2 - Processing parameters
SI 65536
SF 300.1300023 MHz
WDW EM
SSB 0
LB 0.30 Hz
GB 0
PC 1.00

1r

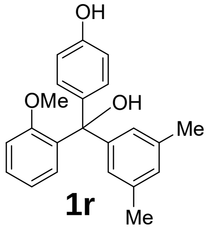

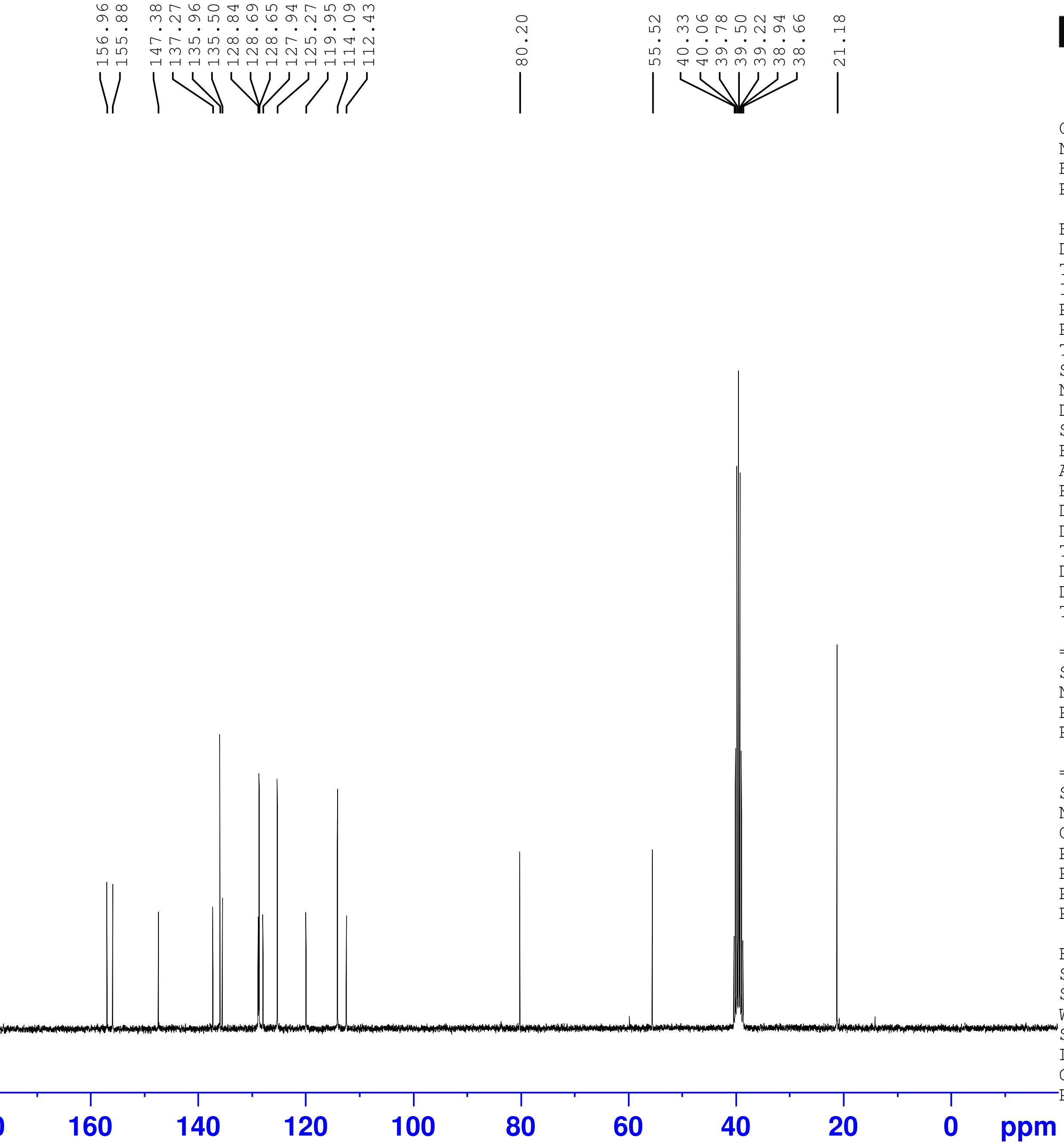

Current Data Parameters
NAME 1r-ZY-4-78A
EXPNO 5461
PROCNO 1

F2 - Acquisition Parameters
Date_ 20210927
Time 13.25
INSTRUM spect
PROBHD 5 mm PABBO BB-
PULPROG zgpg30
TD 65536
SOLVENT DMSO
NS 500
DS 4
SWH 18028.846 Hz
FIDRES 0.275098 Hz
AQ 1.8175317 sec
RG 203
DW 27.733 usec
DE 6.50 usec
TE -59.1 K
D1 2.00000000 sec
D11 0.03000000 sec
TD0 1

======== CHANNEL f1 ========
SFO1 75.4752949 MHz
NUC1 13C
P1 9.50 usec
PLW1 34.20000076 W

======== CHANNEL f2 ========
SFO2 300.1312005 MHz
NUC2 1H
CPDPRG[2 waltz16
PCPD2 90.00 usec
PLW2 14.00000000 W
PLW12 0.17284000 W
PLW13 0.14000000 W

F2 - Processing parameters
SI 32768
SF 75.4677836 MHz
WDW EM
SSB 0
LB 1.00 Hz
GB 0
PC 1.40

1s

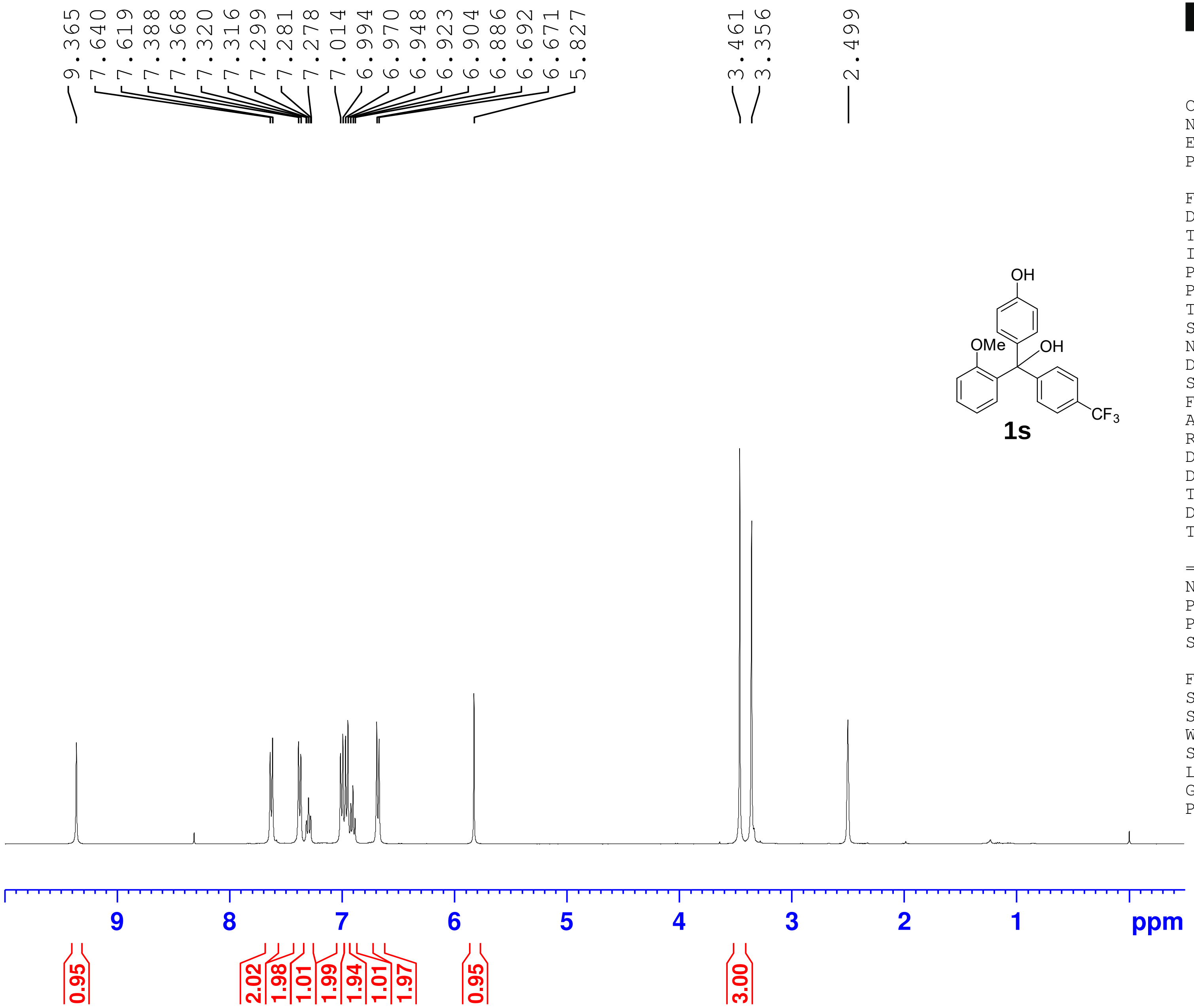

1s

```
Current Data Parameters
NAME              0729-400
EXPNO                  109
PROCNO                   1

F2 - Acquisition Parameters
Date_             20220729
Time                 21.40
INSTRUM              spect
PROBHD   5 mm PABBO BB-
PULPROG               zg30
TD                   65536
SOLVENT               DMSO
NS                       8
DS                       2
SWH               8223.685 Hz
FIDRES            0.125483 Hz
AQ               3.9845889 sec
RG                  140.02
DW                  60.800 usec
DE                    6.50 usec
TE                   294.7 K
D1              1.00000000 sec
TD0                      1

======== CHANNEL f1 ========
NUC1                    1H
P1                   14.68 usec
PLW1           14.00000000 W
SFO1           400.1924713 MHz

F2 - Processing parameters
SI                   65536
SF             400.1900133 MHz
WDW                     EM
SSB                      0
LB                    0.30 Hz
GB                       0
PC                    1.00
```

1s

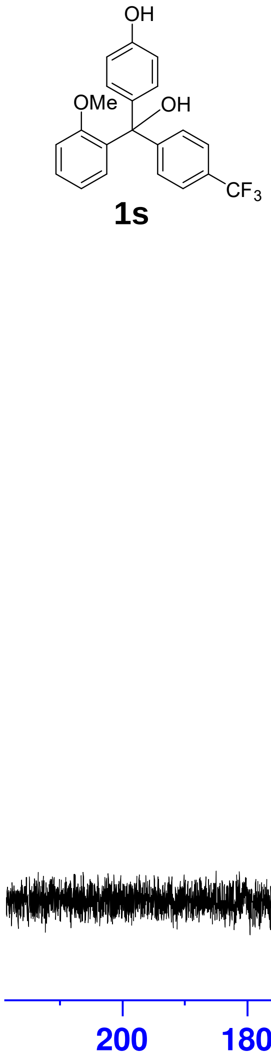

1s

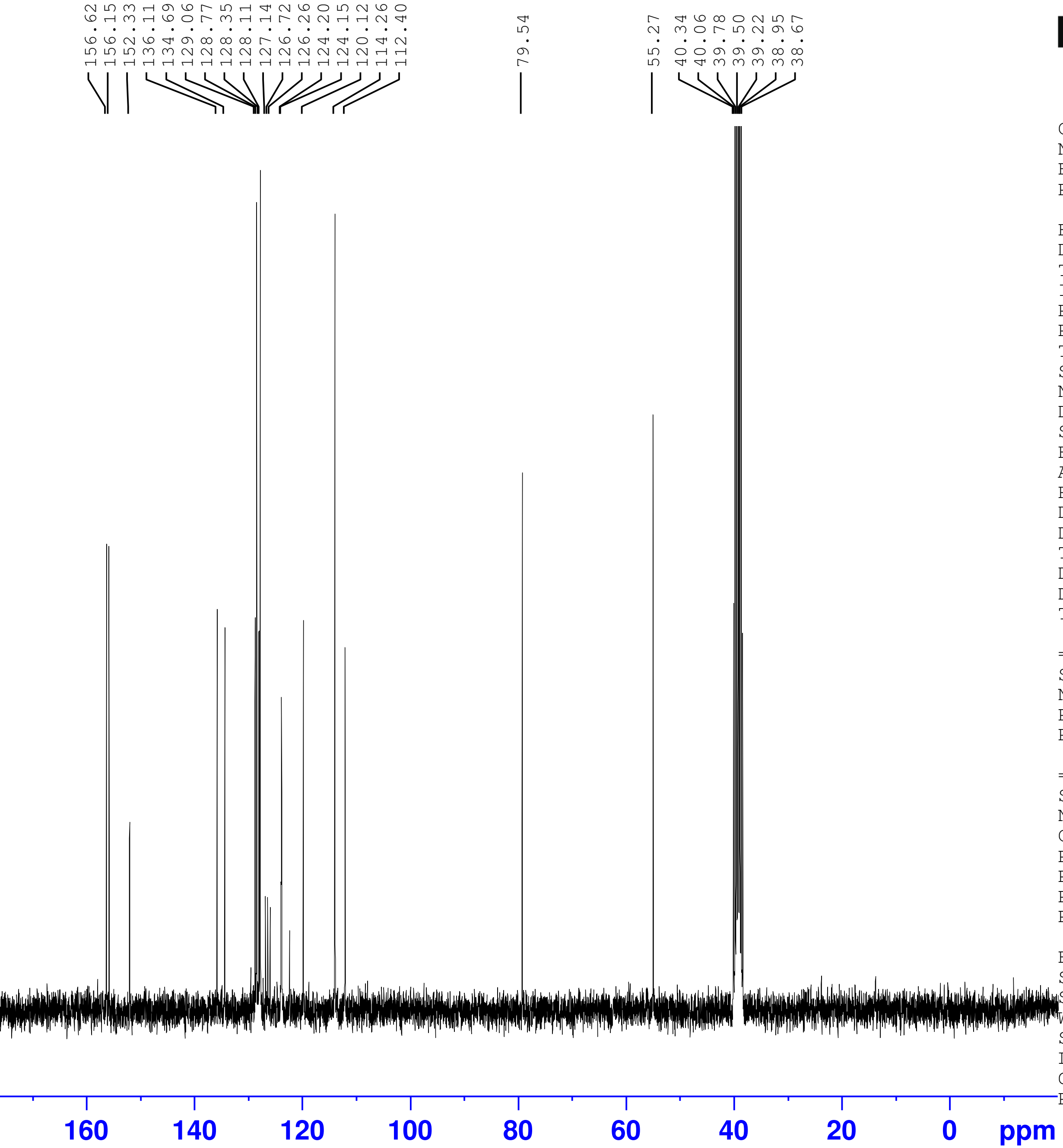


```
Current Data Parameters
NAME          1s-ZY-4-70B
EXPNO                5299
PROCNO                  1

F2 - Acquisition Parameters
Date_            20210909
Time                13.28
INSTRUM             spect
PROBHD   5 mm PABBO BB-
PULPROG           zgpg30
TD                  65536
SOLVENT              DMSO
NS                    300
DS                      4
SWH             18028.846 Hz
FIDRES           0.275098 Hz
AQ              1.8175317 sec
RG                    203
DW                 27.733 usec
DE                   6.50 usec
TE                  -59.1 K
D1             2.00000000 sec
D11            0.03000000 sec
TD0                     1

======== CHANNEL f1 ========
SFO1          75.4752949 MHz
NUC1                  13C
P1                   9.50 usec
PLW1          34.20000076 W

======== CHANNEL f2 ========
SFO2         300.1312005 MHz
NUC2                   1H
CPDPRG[2          waltz16
PCPD2               90.00 usec
PLW2          14.00000000 W
PLW12          0.17284000 W
PLW13          0.14000000 W

F2 - Processing parameters
SI                  32768
SF             75.4677830 MHz
WDW                    EM
SSB                     0
LB                   1.00 Hz
GB                      0
PC                   1.40
```

1s

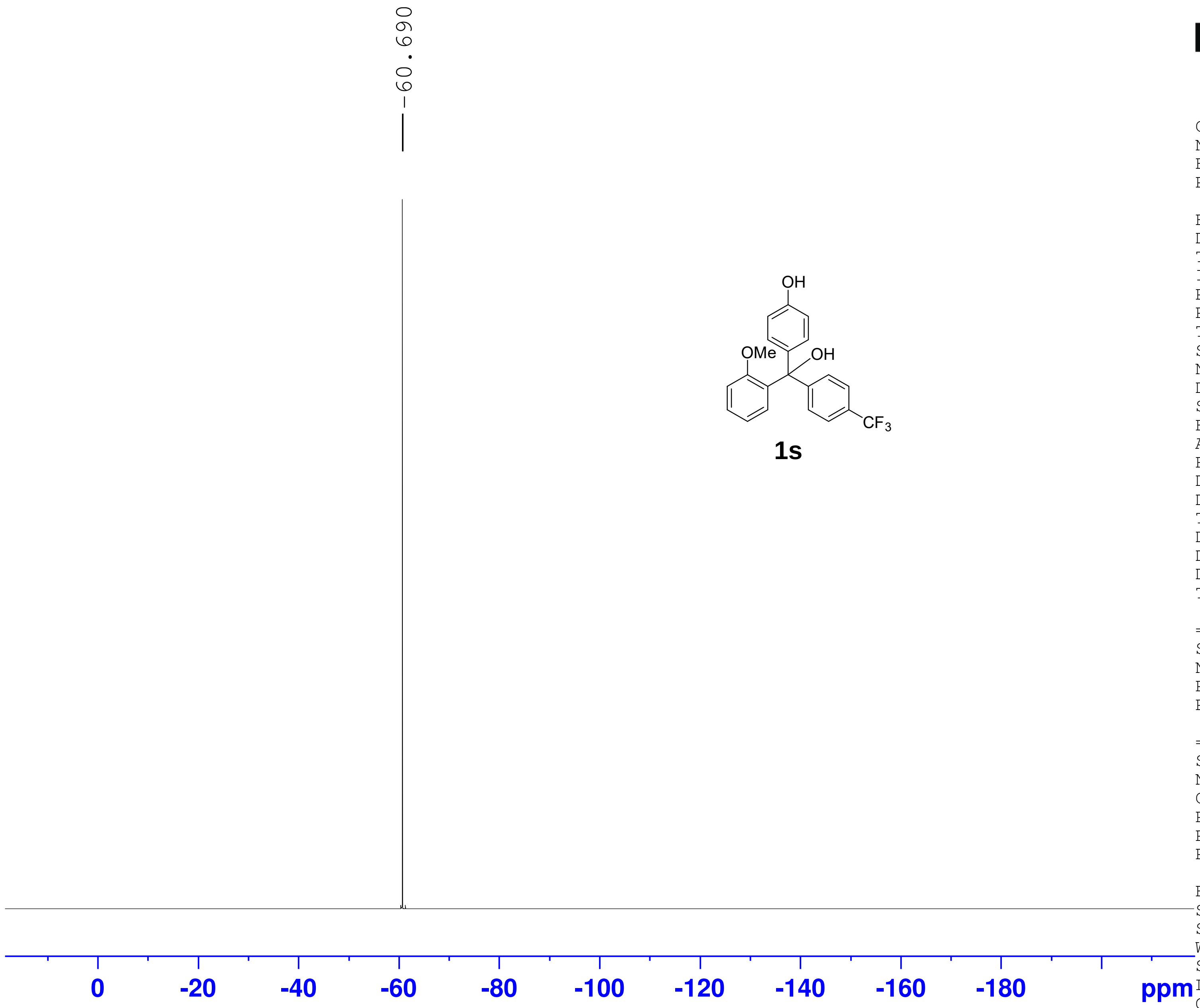


```
Current Data Parameters
NAME               0909sjw
EXPNO                 5300
PROCNO                   1

F2 - Acquisition Parameters
Date_             20210909
Time                 13.31
INSTRUM              spect
PROBHD     5 mm PABBO BB-
PULPROG         zgfhigqn.2
TD                  131072
SOLVENT               DMSO
NS                      16
DS                       4
SWH             66964.289 Hz
FIDRES           0.510897 Hz
AQ              0.9786710 sec
RG                     203
DW                   7.467 usec
DE                    6.50 usec
TE                   -59.1 K
D1              1.00000000 sec
D11             0.03000000 sec
D12             0.00002000 sec
TD0                      1

======== CHANNEL f1 ========
SFO1           282.3761148 MHz
NUC1                   19F
P1                   14.50 usec
PLW1           10.39999962 W

======== CHANNEL f2 ========
SFO2           300.1312005 MHz
NUC2                    1H
CPDPRG[2           waltz16
PCPD2                90.00 usec
PLW2           14.00000000 W
PLW12           0.17284000 W

F2 - Processing parameters
SI                   65536
SF             282.4043552 MHz
WDW                     EM
SSB                      0
LB                    0.30 Hz
GB                       0
PC                    1.00
```

3aa

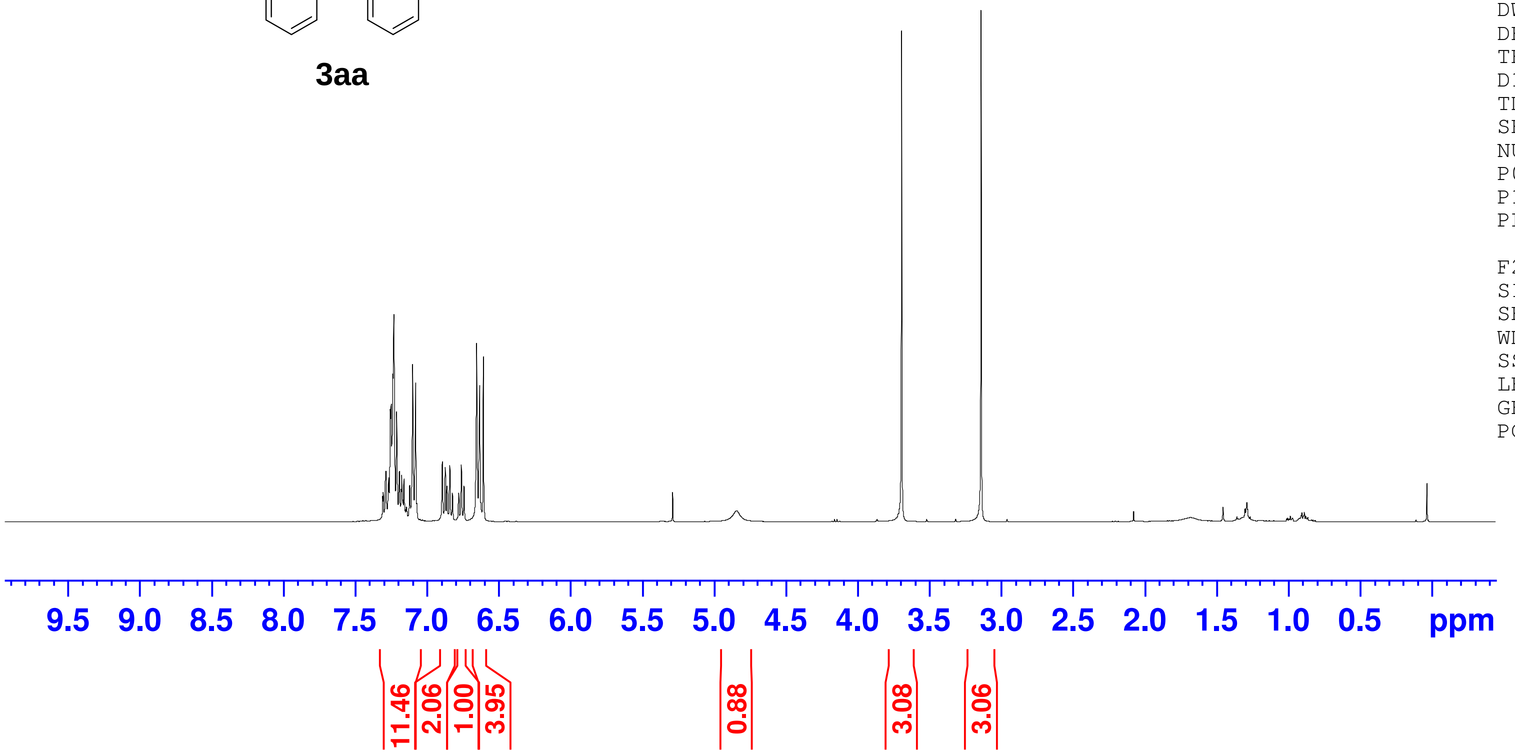

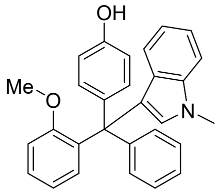

3aa

BRUKER

Current Data Parameters
NAME 0607HH
EXPNO 4
PROCNO 1

F2 - Acquisition Parameters
Date_ 20220608
Time 0.46 h
INSTRUM Avance
PROBHD Z116098_0833 (
PULPROG zg30
TD 65536
SOLVENT CDCl3
NS 16
DS 2
SWH 8196.722 Hz
FIDRES 0.250144 Hz
AQ 3.9976959 sec
RG 60.6061
DW 61.000 usec
DE 13.54 usec
TE 292.0 K
D1 1.00000000 sec
TD0 1
SFO1 400.1324708 MHz
NUC1 1H
P0 3.33 usec
P1 10.00 usec
PLW1 20.73200035 W

F2 - Processing parameters
SI 65536
SF 400.1300112 MHz
WDW EM
SSB 0
LB 0.30 Hz
GB 0
PC 1.00

3aa

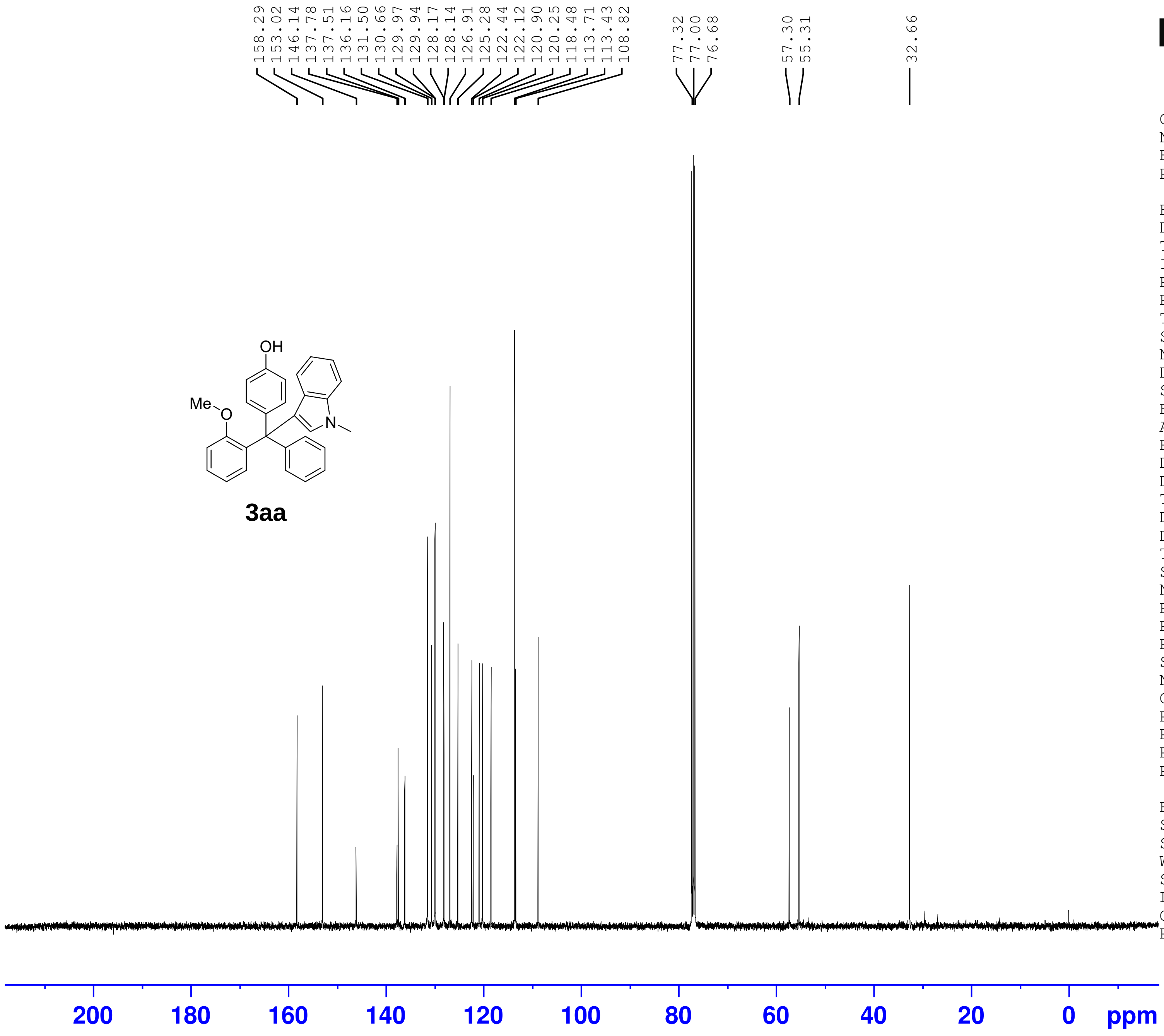

Current Data Parameters
NAME 3aa-ZY-4-1
EXPNO 5
PROCNO 1

F2 - Acquisition Parameters
Date_ 20220608
Time 1.46 h
INSTRUM Avance
PROBHD Z116098_0833 (
PULPROG zgpg30
TD 65536
SOLVENT CDCl3
NS 1024
DS 4
SWH 23809.523 Hz
FIDRES 0.726609 Hz
AQ 1.3762560 sec
RG 51.3764
DW 21.000 usec
DE 6.50 usec
TE 292.6 K
D1 2.00000000 sec
D11 0.03000000 sec
TD0 1
SFO1 100.6228298 MHz
NUC1 13C
P0 3.33 usec
P1 10.00 usec
PLW1 87.89900208 W
SFO2 400.1316005 MHz
NUC2 1H
CPDPRG[2 waltz65
PCPD2 90.00 usec
PLW2 20.73200035 W
PLW12 0.25595000 W
PLW13 0.12874000 W

F2 - Processing parameters
SI 32768
SF 100.6127784 MHz
WDW EM
SSB 0
LB 1.00 Hz
GB 0
PC 1.40

3ab

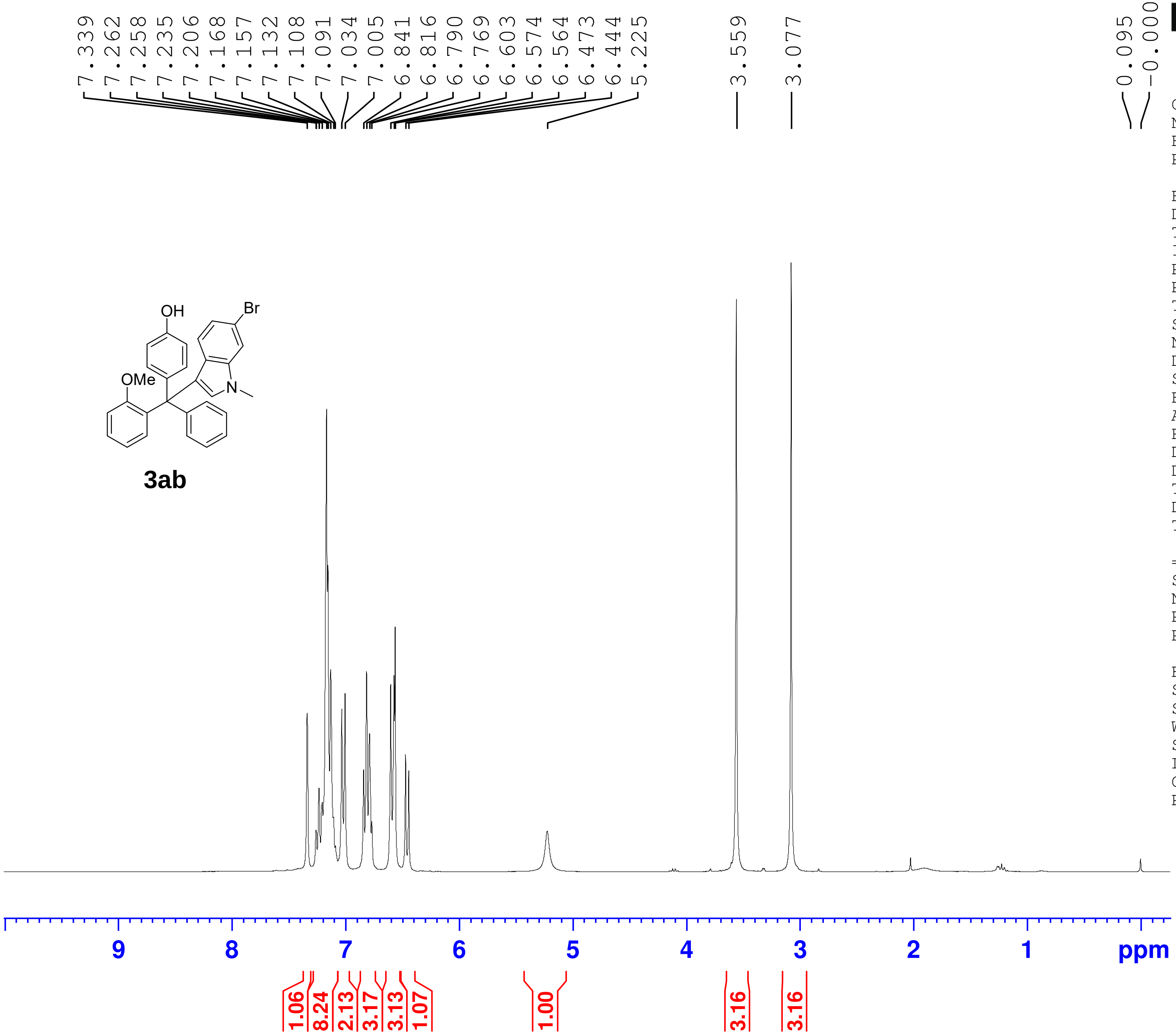

Current Data Parameters
NAME 0929sjw
EXPNO 5465
PROCNO 1

F2 - Acquisition Parameters
Date_ 20210929
Time 10.11
INSTRUM spect
PROBHD 5 mm PABBO BB-
PULPROG zg30
TD 65536
SOLVENT CDCl3
NS 16
DS 2
SWH 6009.615 Hz
FIDRES 0.091699 Hz
AQ 5.4525952 sec
RG 32
DW 83.200 usec
DE 6.50 usec
TE -59.1 K
D1 1.00000000 sec
TD0 1

======== CHANNEL f1 ========
SFO1 300.1318534 MHz
NUC1 1H
P1 10.00 usec
PLW1 14.00000000 W

F2 - Processing parameters
SI 65536
SF 300.1300344 MHz
WDW EM
SSB 0
LB 0.30 Hz
GB 0
PC 1.00

3ab

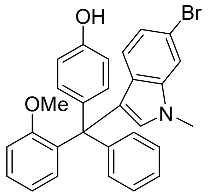

3ab

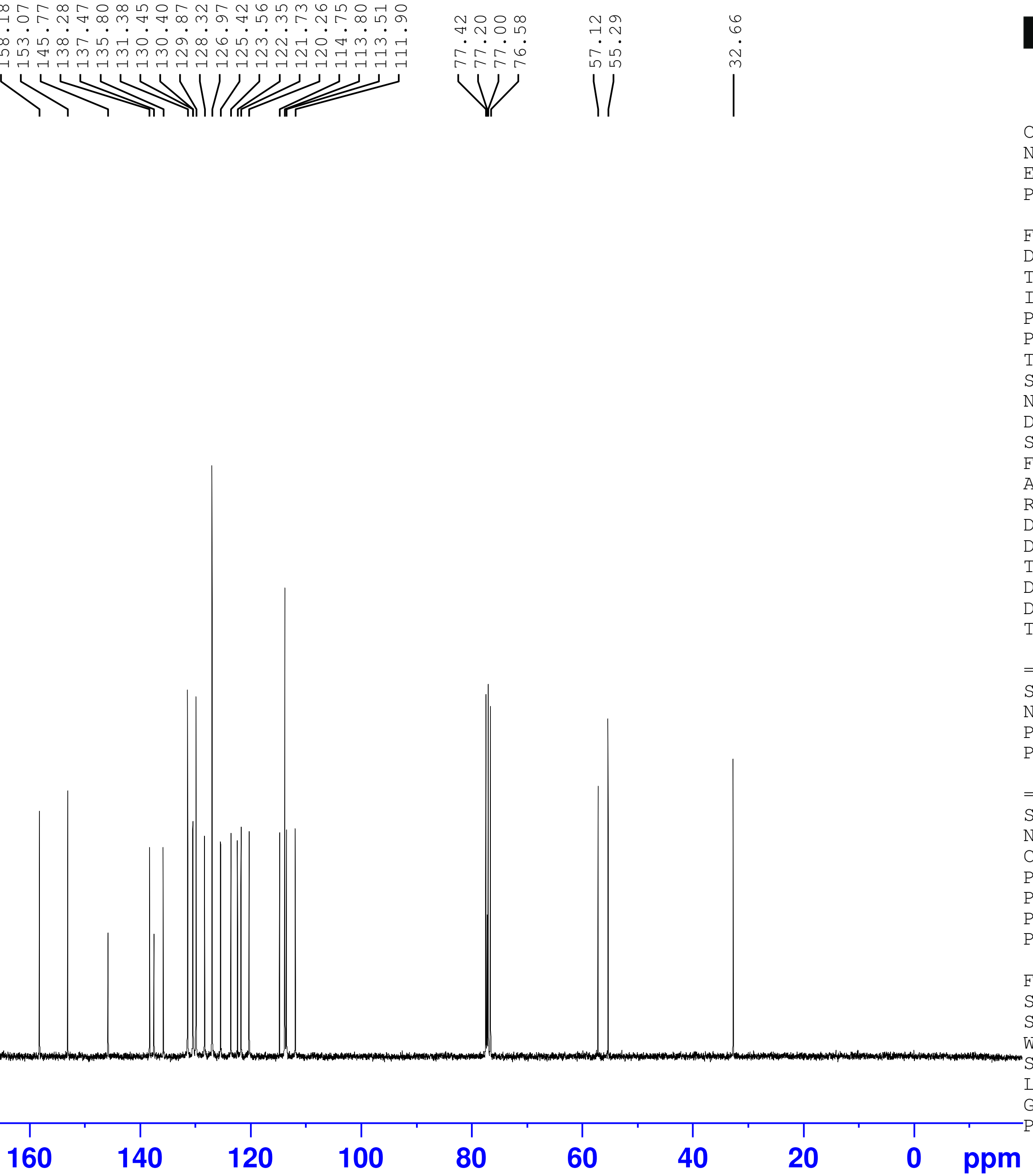

BRUKER

Current Data Parameters
NAME 3ab-ZY-4-64B
EXPNO 5496
PROCNO 1

F2 - Acquisition Parameters
Date_ 20210930
Time 12.04
INSTRUM spect
PROBHD 5 mm PABBO BB-
PULPROG zgpg30
TD 65536
SOLVENT CDCl3
NS 500
DS 4
SWH 18028.846 Hz
FIDRES 0.275098 Hz
AQ 1.8175317 sec
RG 203
DW 27.733 usec
DE 6.50 usec
TE -59.1 K
D1 2.00000000 sec
D11 0.03000000 sec
TD0 1

======== CHANNEL f1 ========
SFO1 75.4752949 MHz
NUC1 13C
P1 9.50 usec
PLW1 34.20000076 W

======== CHANNEL f2 ========
SFO2 300.1312005 MHz
NUC2 1H
CPDPRG[2 waltz16
PCPD2 90.00 usec
PLW2 14.00000000 W
PLW12 0.17284000 W
PLW13 0.14000000 W

F2 - Processing parameters
SI 32768
SF 75.4677642 MHz
WDW EM
SSB 0
LB 1.00 Hz
GB 0
PC 1.40

3ac

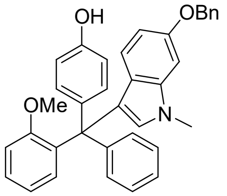

3ac

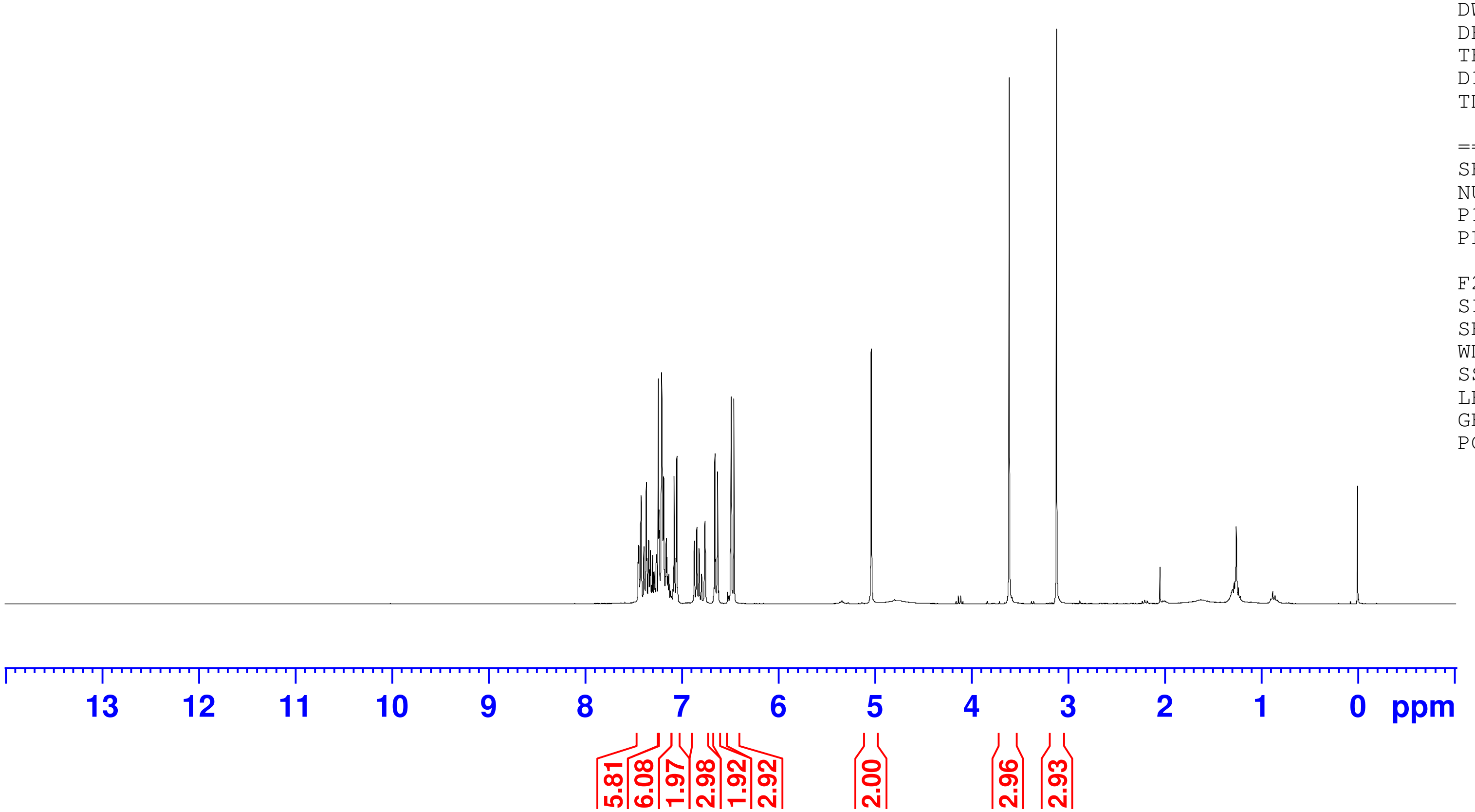

Current Data Parameters

NAME	ZY-4-64G-h-fr
EXPNO	5413
PROCNO	1

F2 - Acquisition Parameters

Date_	20210924
Time	10.18
INSTRUM	spect
PROBHD	5 mm PABBO BB-
PULPROG	zg30
TD	65536
SOLVENT	CDCl3
NS	16
DS	2
SWH	6009.615 Hz
FIDRES	0.091699 Hz
AQ	5.4525952 sec
RG	144
DW	83.200 usec
DE	6.50 usec
TE	-59.1 K
D1	1.00000000 sec
TD0	1

======== CHANNEL f1 ========

SFO1	300.1318534 MHz
NUC1	1H
P1	10.00 usec
PLW1	14.00000000 W

F2 - Processing parameters

SI	65536
SF	300.1300127 MHz
WDW	EM
SSB	0
LB	0.30 Hz
GB	0
PC	1.00

3ac

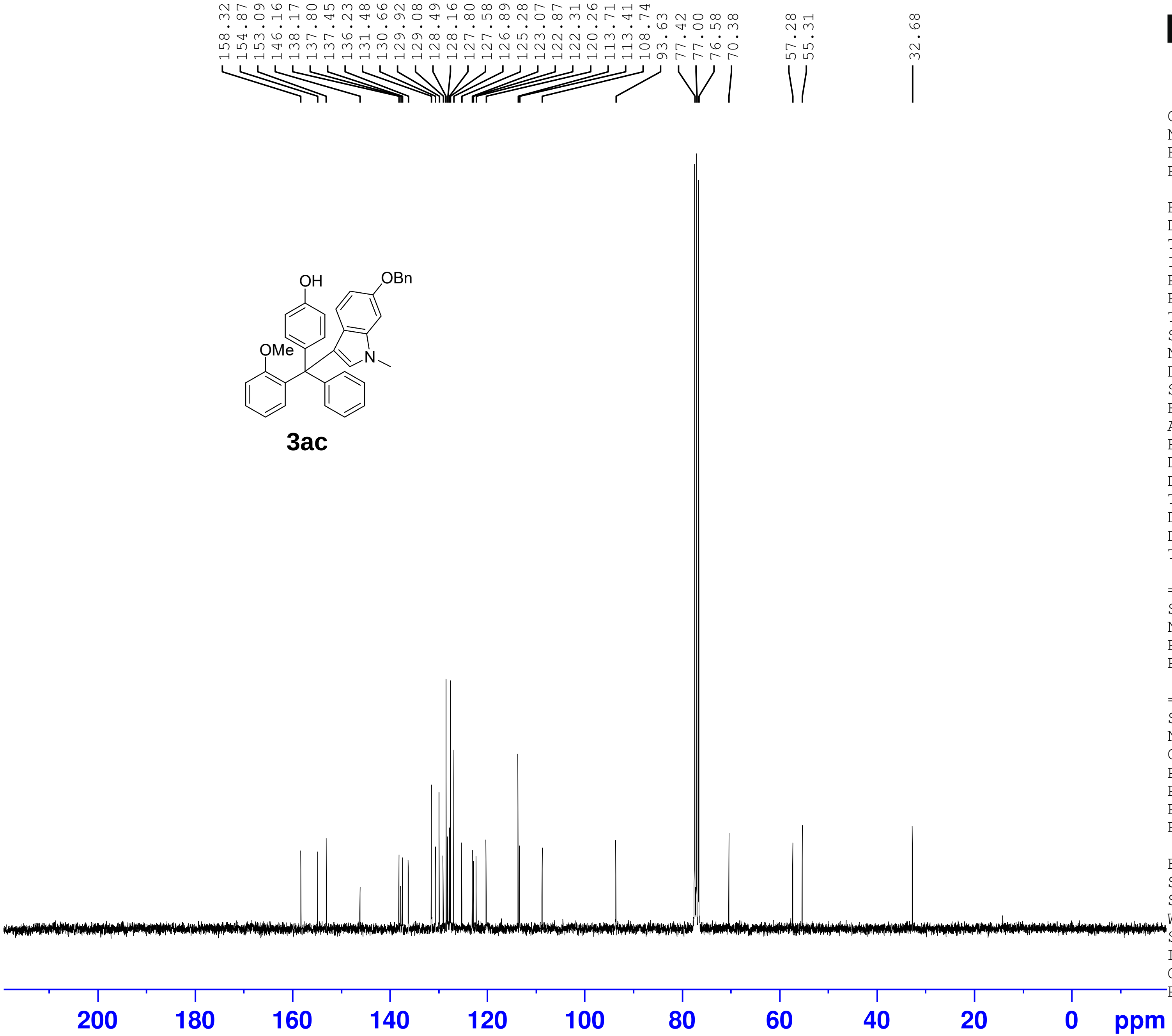

3ac

Current Data Parameters
NAME 3ac-ZY-4-64G
EXPNO 5338
PROCNO 1

F2 - Acquisition Parameters
Date_ 20210915
Time 11.47
INSTRUM spect
PROBHD 5 mm PABBO BB-
PULPROG zgpg30
TD 65536
SOLVENT CDCl3
NS 800
DS 4
SWH 18028.846 Hz
FIDRES 0.275098 Hz
AQ 1.8175317 sec
RG 203
DW 27.733 usec
DE 6.50 usec
TE -59.1 K
D1 2.00000000 sec
D11 0.03000000 sec
TD0 1

======== CHANNEL f1 ========
SFO1 75.4752949 MHz
NUC1 13C
P1 9.50 usec
PLW1 34.20000076 W

======== CHANNEL f2 ========
SFO2 300.1312005 MHz
NUC2 1H
CPDPRG[2 waltz16
PCPD2 90.00 usec
PLW2 14.00000000 W
PLW12 0.17284000 W
PLW13 0.14000000 W

F2 - Processing parameters
SI 32768
SF 75.4677531 MHz
WDW EM
SSB 0
LB 1.00 Hz
GB 0
PC 1.40

3ad

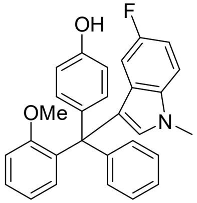

3ad

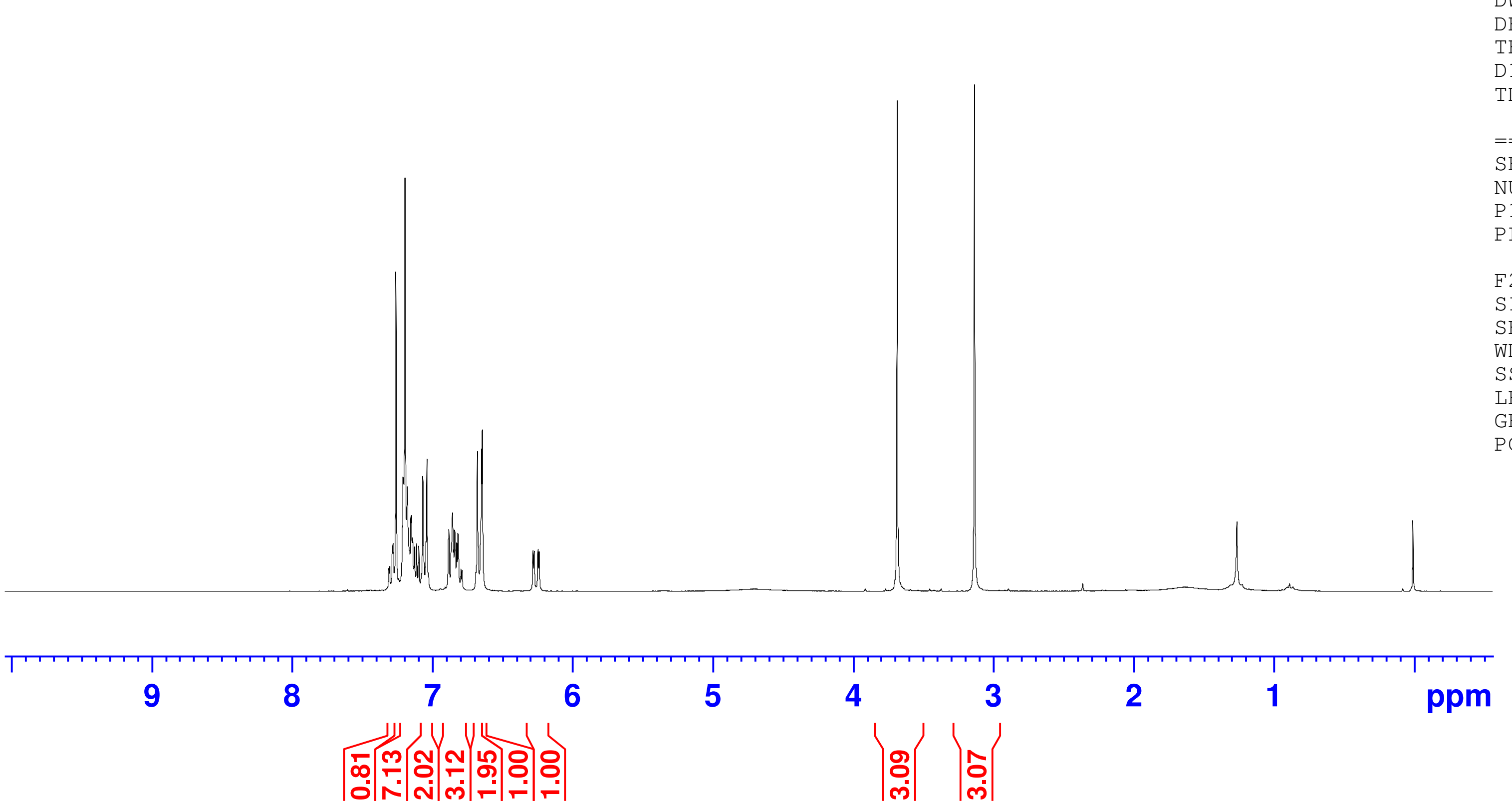

BRUKER

Current Data Parameters

NAME	ZY-4-64C-h-fr
EXPNO	5542
PROCNO	1

F2 - Acquisition Parameters

Date_	20211019
Time	9.45
INSTRUM	spect
PROBHD	5 mm PABBO BB-
PULPROG	zg30
TD	65536
SOLVENT	CDCl3
NS	16
DS	2
SWH	6009.615 Hz
FIDRES	0.091699 Hz
AQ	5.4525952 sec
RG	203
DW	83.200 usec
DE	6.50 usec
TE	-59.1 K
D1	1.00000000 sec
TD0	1

======== CHANNEL f1 ========

SFO1	300.1318534 MHz
NUC1	1H
P1	10.00 usec
PLW1	14.00000000 W

F2 - Processing parameters

SI	65536
SF	300.1300072 MHz
WDW	EM
SSB	0
LB	0.30 Hz
GB	0
PC	1.00

3ad

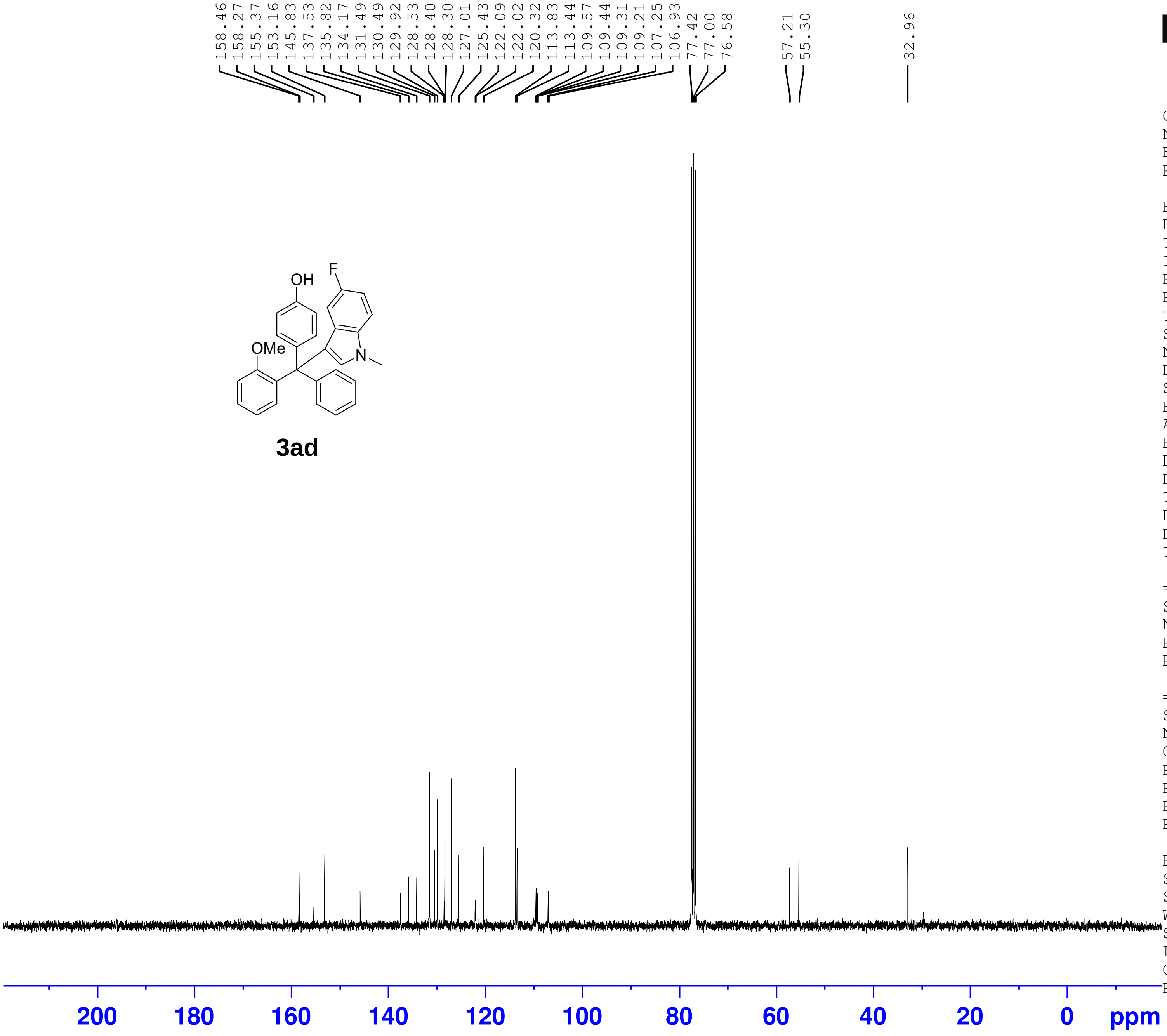

3ad

BRUKER

```
Current Data Parameters
NAME          3ad-ZY-4-64C
EXPNO                 5543
PROCNO                   1

F2 - Acquisition Parameters
Date_             20211019
Time                 10.53
INSTRUM              spect
PROBHD     5 mm PABBO BB-
PULPROG            zgpg30
TD                   65536
SOLVENT              CDCl3
NS                    1024
DS                       4
SWH              18028.846 Hz
FIDRES            0.275098 Hz
AQ               1.8175317 sec
RG                     203
DW                  27.733 usec
DE                    6.50 usec
TE                   -59.1 K
D1              2.00000000 sec
D11             0.03000000 sec
TD0                      1

======== CHANNEL f1 ========
SFO1            75.4752949 MHz
NUC1                   13C
P1                    9.50 usec
PLW1           34.20000076 W

======== CHANNEL f2 ========
SFO2           300.1312005 MHz
NUC2                    1H
CPDPRG[2           waltz16
PCPD2                90.00 usec
PLW2           14.00000000 W
PLW12           0.17284000 W
PLW13           0.14000000 W

F2 - Processing parameters
SI                   32768
SF              75.4677522 MHz
WDW                     EM
SSB                      0
LB                    1.00 Hz
GB                       0
PC                    1.40
```

3ad

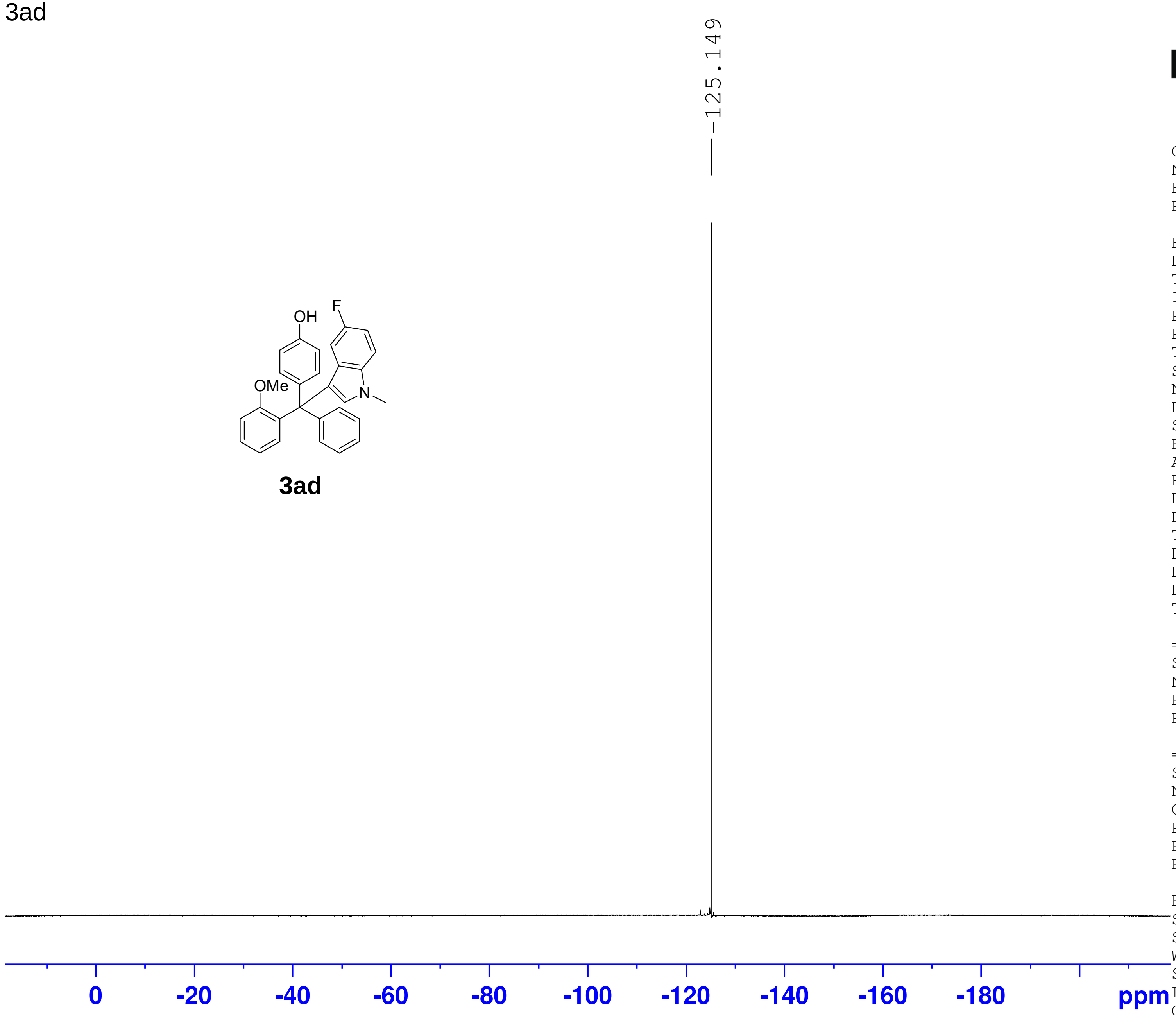

BRUKER

Current Data Parameters
NAME 211010sjw
EXPNO 5511
PROCNO 1

F2 - Acquisition Parameters
Date_ 20211010
Time 9.52
INSTRUM spect
PROBHD 5 mm PABBO BB-
PULPROG zgfhigqn.2
TD 131072
SOLVENT CDCl3
NS 16
DS 4
SWH 66964.289 Hz
FIDRES 0.510897 Hz
AQ 0.9786710 sec
RG 203
DW 7.467 usec
DE 6.50 usec
TE -59.1 K
D1 1.00000000 sec
D11 0.03000000 sec
D12 0.00002000 sec
TD0 1

======== CHANNEL f1 ========
SFO1 282.3761148 MHz
NUC1 19F
P1 14.50 usec
PLW1 10.39999962 W

======== CHANNEL f2 ========
SFO2 300.1312005 MHz
NUC2 1H
CPDPRG[2 waltz16
PCPD2 90.00 usec
PLW2 14.00000000 W
PLW12 0.17284000 W

F2 - Processing parameters
SI 65536
SF 282.4043552 MHz
WDW EM
SSB 0
LB 0.30 Hz
GB 0
PC 1.00

3ae

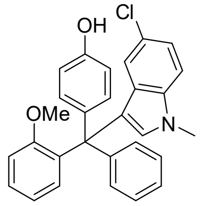

3ae

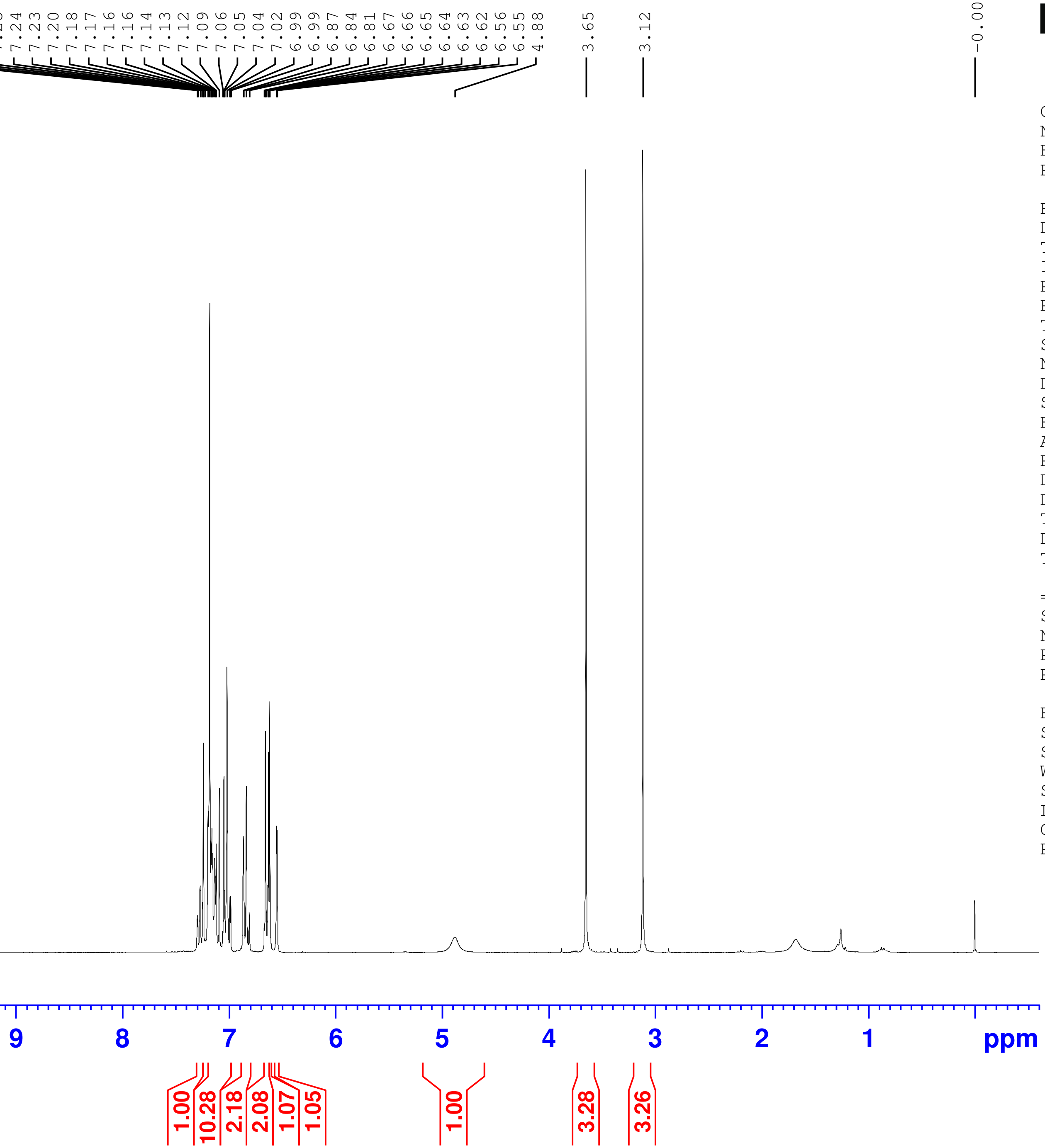

Current Data Parameters
NAME ZY-4-64D-h-fr
EXPNO 5340
PROCNO 1

F2 - Acquisition Parameters
Date_ 20210914
Time 13.44
INSTRUM spect
PROBHD 5 mm PABBO BB-
PULPROG zg30
TD 65536
SOLVENT CDCl3
NS 16
DS 2
SWH 6009.615 Hz
FIDRES 0.091699 Hz
AQ 5.4525952 sec
RG 128
DW 83.200 usec
DE 6.50 usec
TE -59.1 K
D1 1.00000000 sec
TD0 1

======== CHANNEL f1 ========
SFO1 300.1318534 MHz
NUC1 1H
P1 10.00 usec
PLW1 14.00000000 W

F2 - Processing parameters
SI 65536
SF 300.1300129 MHz
WDW EM
SSB 0
LB 0.30 Hz
GB 0
PC 1.00

3ae

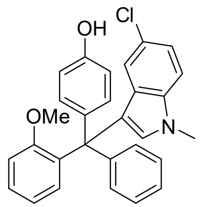

3ae

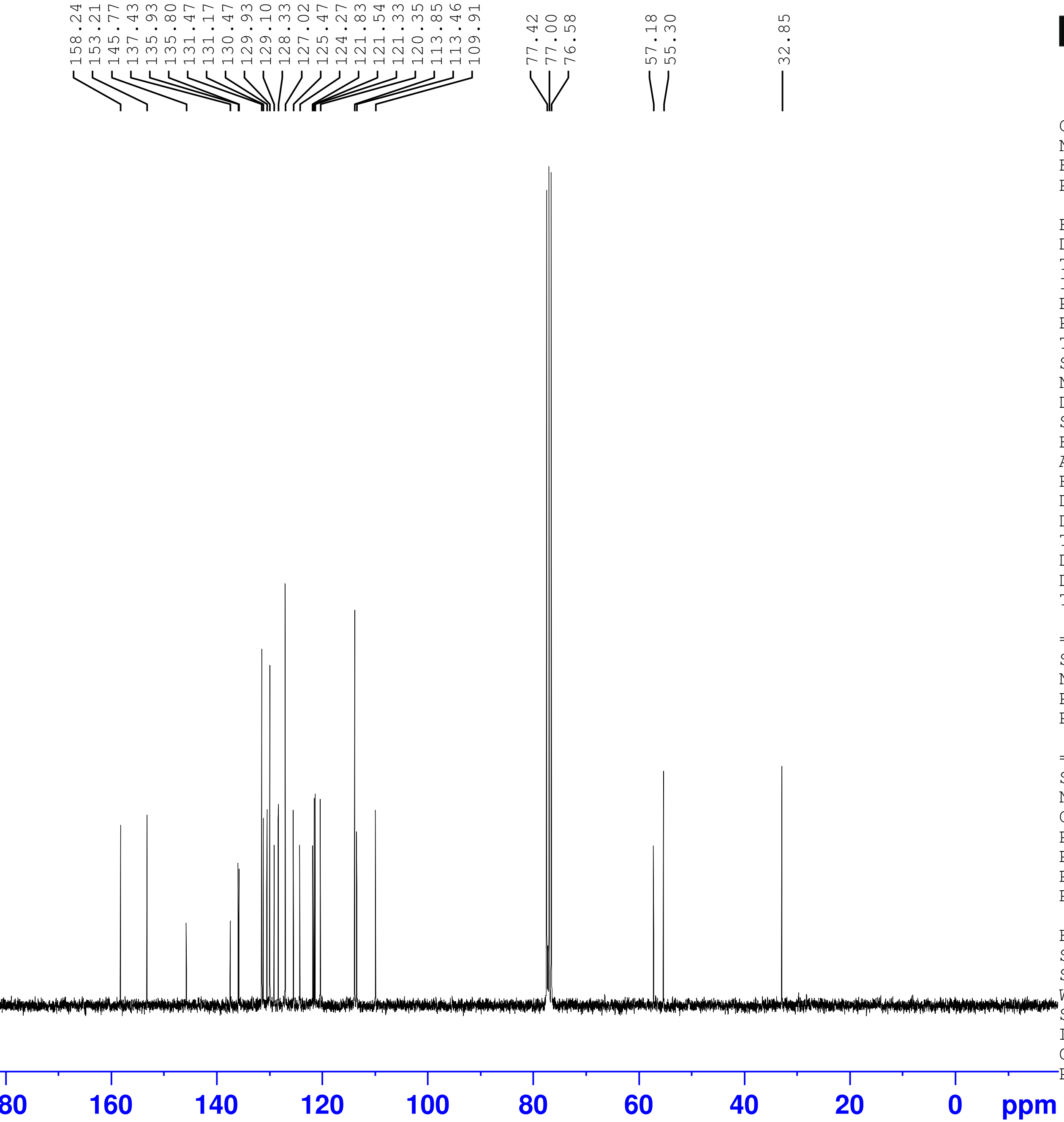

Current Data Parameters
NAME 3ae-ZY-4-64D
EXPNO 5337
PROCNO 1

F2 - Acquisition Parameters
Date_ 20210915
Time 10.51
INSTRUM spect
PROBHD 5 mm PABBO BB-
PULPROG zgpg30
TD 65536
SOLVENT CDCl3
NS 800
DS 4
SWH 18028.846 Hz
FIDRES 0.275098 Hz
AQ 1.8175317 sec
RG 203
DW 27.733 usec
DE 6.50 usec
TE -59.1 K
D1 2.00000000 sec
D11 0.03000000 sec
TD0 1

======== CHANNEL f1 ========
SFO1 75.4752949 MHz
NUC1 13C
P1 9.50 usec
PLW1 34.20000076 W

======== CHANNEL f2 ========
SFO2 300.1312005 MHz
NUC2 1H
CPDPRG[2 waltz16
PCPD2 90.00 usec
PLW2 14.00000000 W
PLW12 0.17284000 W
PLW13 0.14000000 W

F2 - Processing parameters
SI 32768
SF 75.4677539 MHz
WDW EM
SSB 0
LB 1.00 Hz
GB 0
PC 1.40

3af

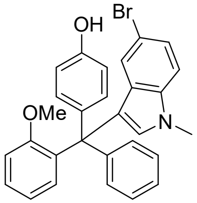

3af

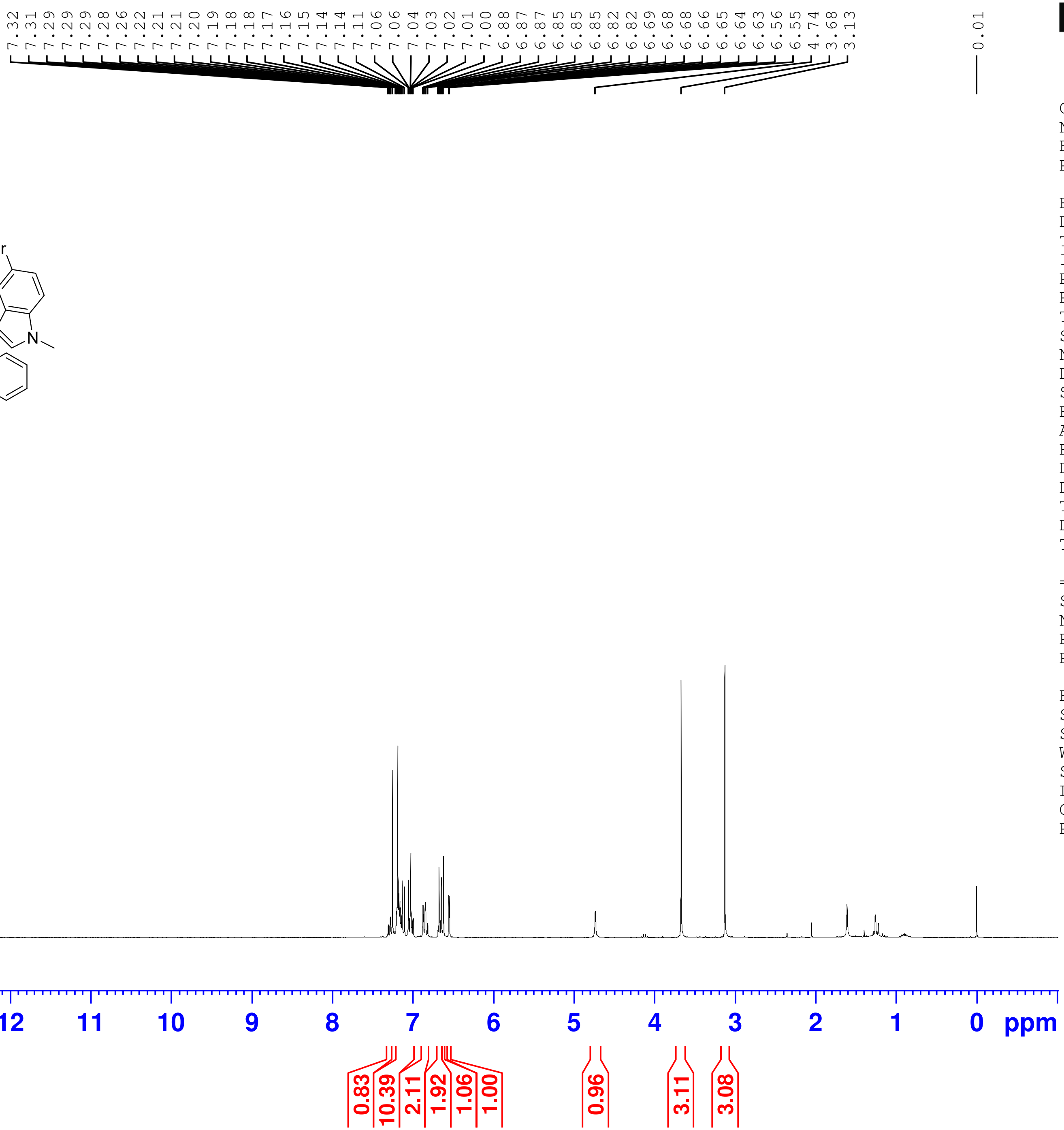

Current Data Parameters
NAME ZY-4-64E-h-fr
EXPNO 5617
PROCNO 1

F2 - Acquisition Parameters
Date_ 20211117
Time 10.35
INSTRUM spect
PROBHD 5 mm PABBO BB-
PULPROG zg30
TD 65536
SOLVENT CDCl3
NS 16
DS 2
SWH 6009.615 Hz
FIDRES 0.091699 Hz
AQ 5.4525952 sec
RG 181
DW 83.200 usec
DE 6.50 usec
TE -59.1 K
D1 1.00000000 sec
TD0 1

======== CHANNEL f1 ========
SFO1 300.1318534 MHz
NUC1 1H
P1 10.00 usec
PLW1 14.00000000 W

F2 - Processing parameters
SI 65536
SF 300.1300097 MHz
WDW EM
SSB 0
LB 0.30 Hz
GB 0
PC 1.00

3af

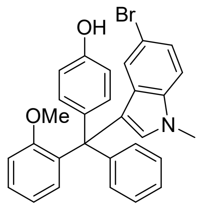

3af

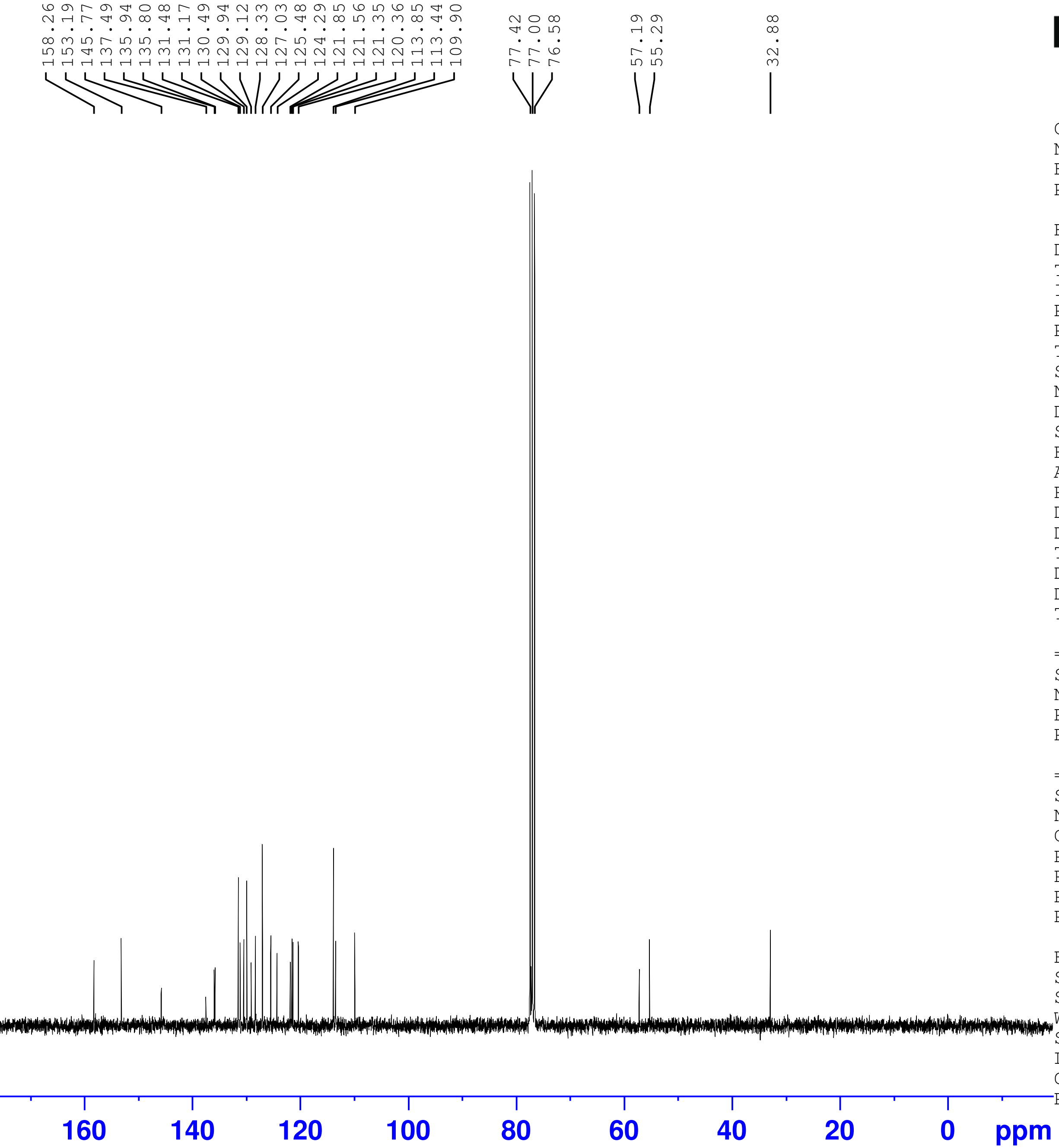

Current Data Parameters
NAME 3af-ZY-4-64E
EXPNO 5618
PROCNO 1

F2 - Acquisition Parameters
Date_ 20211117
Time 11.09
INSTRUM spect
PROBHD 5 mm PABBO BB-
PULPROG zgpg30
TD 65536
SOLVENT CDCl3
NS 500
DS 4
SWH 18028.846 Hz
FIDRES 0.275098 Hz
AQ 1.8175317 sec
RG 203
DW 27.733 usec
DE 6.50 usec
TE -59.1 K
D1 2.00000000 sec
D11 0.03000000 sec
TD0 1

======== CHANNEL f1 ========
SFO1 75.4752949 MHz
NUC1 13C
P1 9.50 usec
PLW1 34.20000076 W

======== CHANNEL f2 ========
SFO2 300.1312005 MHz
NUC2 1H
CPDPRG[2 waltz16
PCPD2 90.00 usec
PLW2 14.00000000 W
PLW12 0.17284000 W
PLW13 0.14000000 W

F2 - Processing parameters
SI 32768
SF 75.4677521 MHz
WDW EM
SSB 0
LB 1.00 Hz
GB 0
PC 1.40

3ag

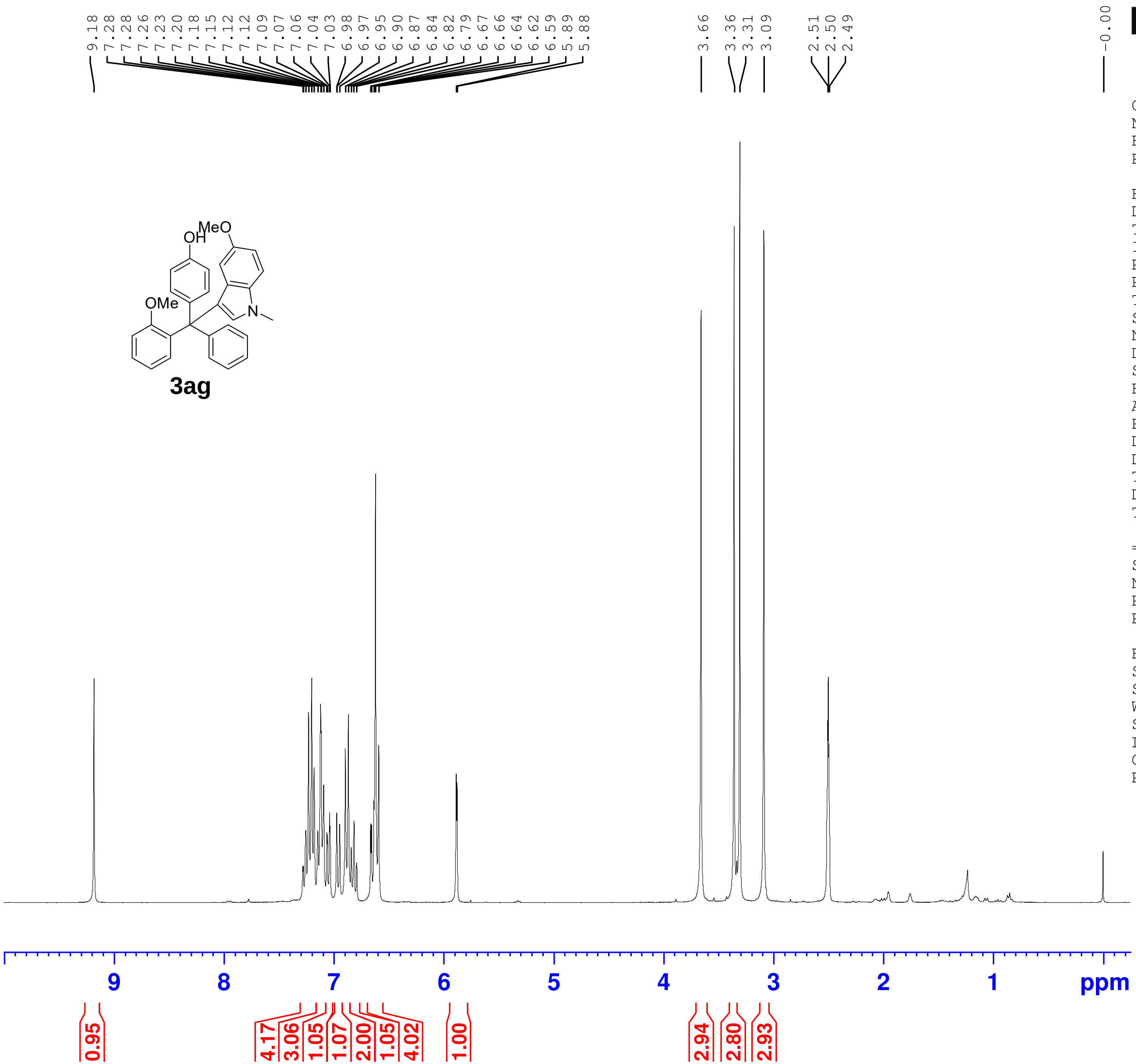

BRUKER

Current Data Parameters
NAME ZY-4-64H-h-fr
EXPNO 5301
PROCNO 1

F2 - Acquisition Parameters
Date_ 20210909
Time 13.35
INSTRUM spect
PROBHD 5 mm PABBO BB-
PULPROG zg30
TD 65536
SOLVENT DMSO
NS 16
DS 2
SWH 6009.615 Hz
FIDRES 0.091699 Hz
AQ 5.4525952 sec
RG 144
DW 83.200 usec
DE 6.50 usec
TE -59.1 K
D1 1.00000000 sec
TD0 1

======== CHANNEL f1 ========
SFO1 300.1318534 MHz
NUC1 1H
P1 10.00 usec
PLW1 14.00000000 W

F2 - Processing parameters
SI 65536
SF 300.1300013 MHz
WDW EM
SSB 0
LB 0.30 Hz
GB 0
PC 1.00

3ag

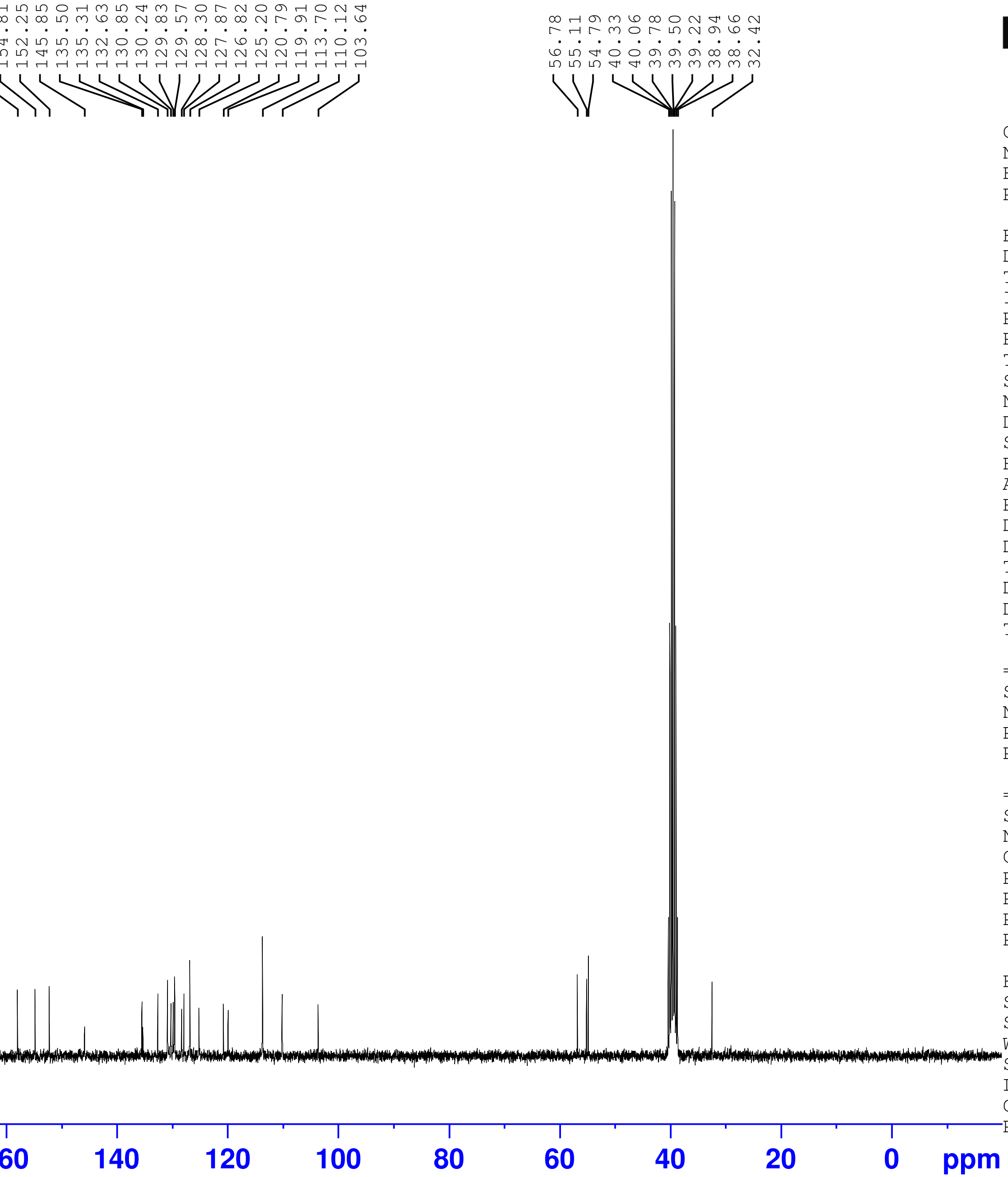

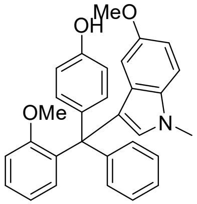

3ag

```
Current Data Parameters
NAME          3ag-ZY-4-64H
EXPNO                 5311
PROCNO                   1

F2 - Acquisition Parameters
Date_             20210910
Time                 13.51
INSTRUM              spect
PROBHD     5 mm PABBO BB-
PULPROG            zgpg30
TD                   65536
SOLVENT               DMSO
NS                     473
DS                       4
SWH              18028.846 Hz
FIDRES            0.275098 Hz
AQ               1.8175317 sec
RG                     203
DW                  27.733 usec
DE                    6.50 usec
TE                   -59.1 K
D1              2.00000000 sec
D11             0.03000000 sec
TD0                      1

======== CHANNEL f1 ========
SFO1            75.4752949 MHz
NUC1                   13C
P1                    9.50 usec
PLW1           34.20000076 W

======== CHANNEL f2 ========
SFO2           300.1312005 MHz
NUC2                    1H
CPDPRG[2           waltz16
PCPD2                90.00 usec
PLW2           14.00000000 W
PLW12           0.17284000 W
PLW13           0.14000000 W

F2 - Processing parameters
SI                   32768
SF              75.4677847 MHz
WDW                     EM
SSB                      0
LB                    1.00 Hz
GB                       0
PC                    1.40
```

3ah

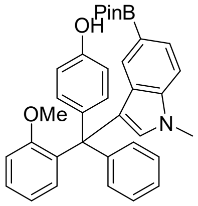

3ah

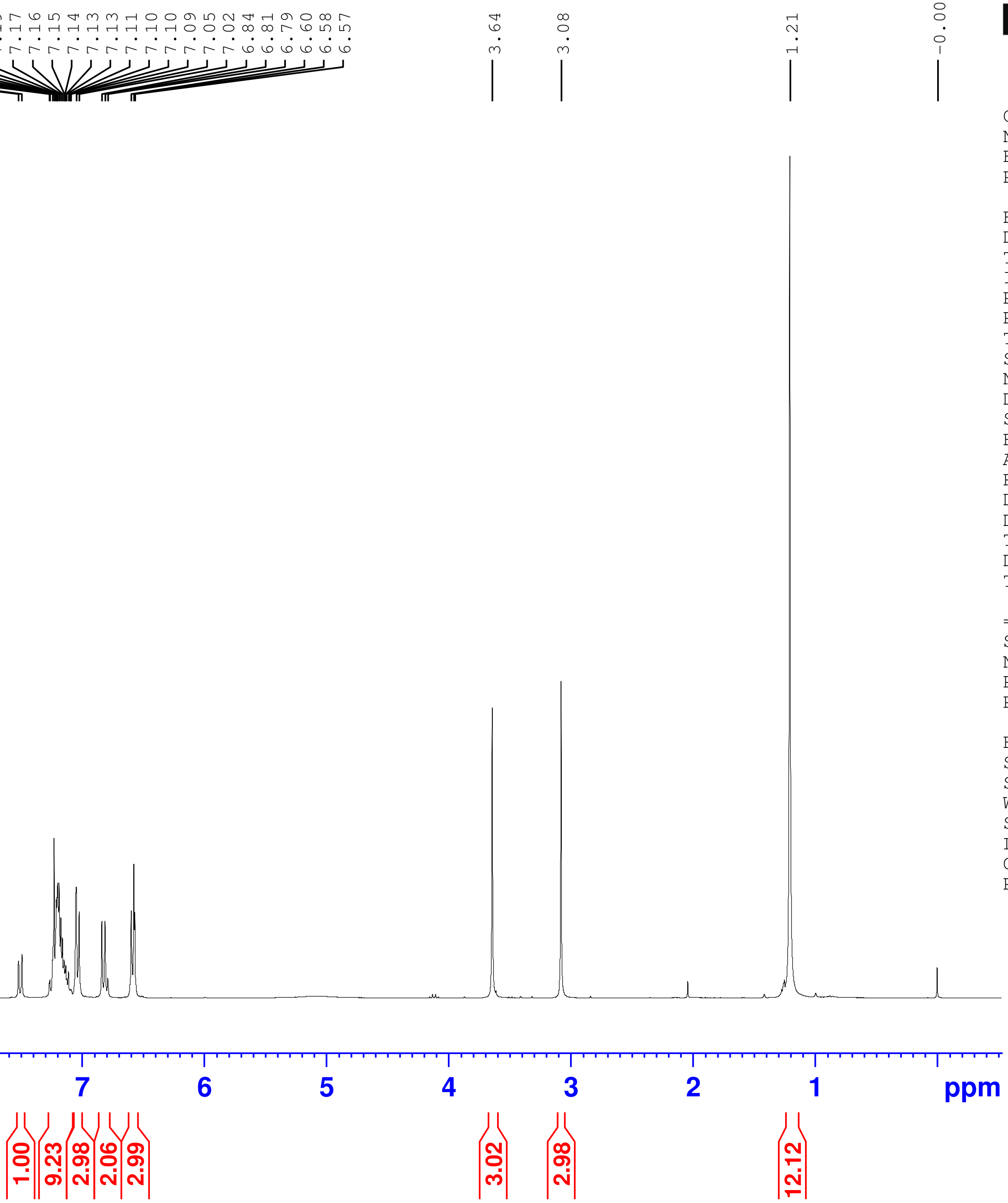

BRUKER

Current Data Parameters
NAME ZY-4-72-h-fr
EXPNO 5589
PROCNO 1

F2 - Acquisition Parameters
Date_ 20211029
Time 23.47
INSTRUM spect
PROBHD 5 mm PABBO BB-
PULPROG zg30
TD 65536
SOLVENT CDCl3
NS 16
DS 2
SWH 6009.615 Hz
FIDRES 0.091699 Hz
AQ 5.4525952 sec
RG 80.6
DW 83.200 usec
DE 6.50 usec
TE -59.1 K
D1 1.00000000 sec
TD0 1

======== CHANNEL f1 ========
SFO1 300.1318534 MHz
NUC1 1H
P1 10.00 usec
PLW1 14.00000000 W

F2 - Processing parameters
SI 65536
SF 300.1300161 MHz
WDW EM
SSB 0
LB 0.30 Hz
GB 0
PC 1.00

3ah

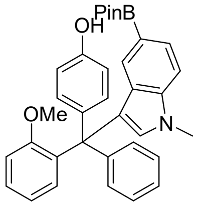

3ah

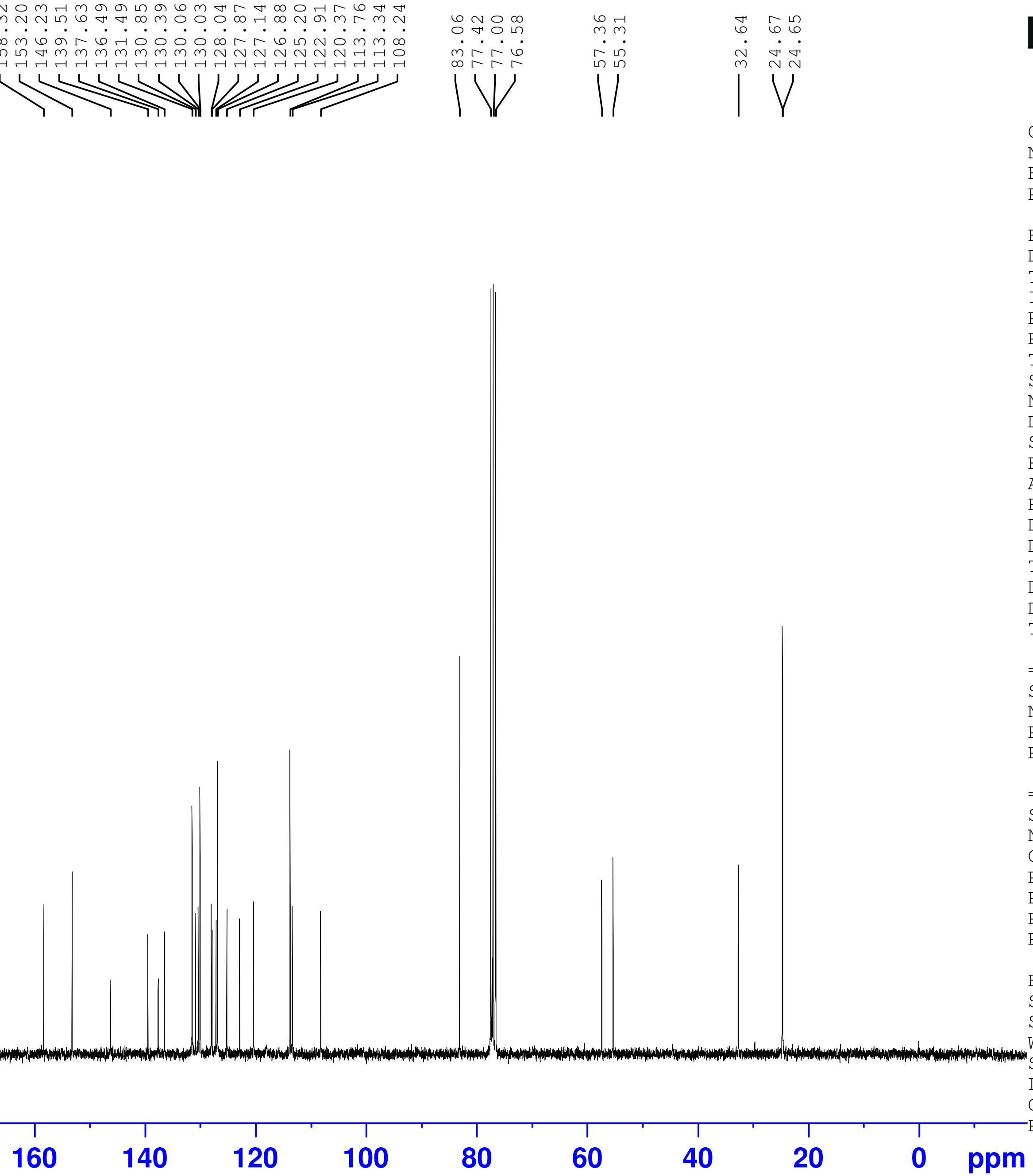

Current Data Parameters
NAME 3ah-ZY-4-72
EXPNO 5590
PROCNO 1

F2 - Acquisition Parameters
Date_ 20211030
Time 0.34
INSTRUM spect
PROBHD 5 mm PABBO BB-
PULPROG zgpg30
TD 65536
SOLVENT CDCl3
NS 700
DS 4
SWH 18028.846 Hz
FIDRES 0.275098 Hz
AQ 1.8175317 sec
RG 203
DW 27.733 usec
DE 6.50 usec
TE -59.1 K
D1 2.00000000 sec
D11 0.03000000 sec
TD0 1

======== CHANNEL f1 ========
SFO1 75.4752949 MHz
NUC1 13C
P1 9.50 usec
PLW1 34.20000076 W

======== CHANNEL f2 ========
SFO2 300.1312005 MHz
NUC2 1H
CPDPRG[2 waltz16
PCPD2 90.00 usec
PLW2 14.00000000 W
PLW12 0.17284000 W
PLW13 0.14000000 W

F2 - Processing parameters
SI 32768
SF 75.4677552 MHz
WDW EM
SSB 0
LB 1.00 Hz
GB 0
PC 1.40

3ai

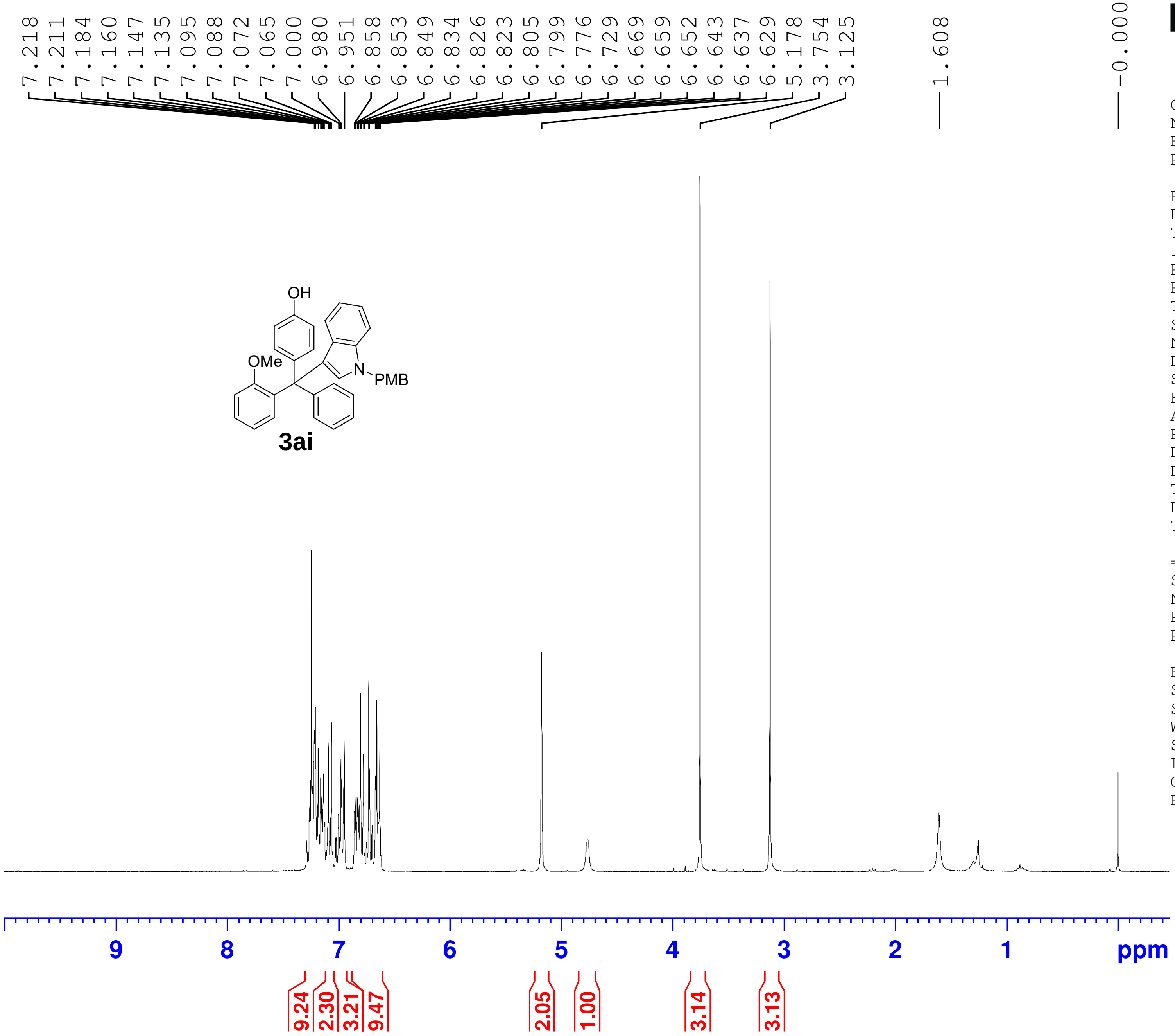

Current Data Parameters

NAME	0914sjw
EXPNO	5338
PROCNO	1

F2 - Acquisition Parameters

Date_	20210914
Time	12.39
INSTRUM	spect
PROBHD	5 mm PABBO BB-
PULPROG	zg30
TD	65536
SOLVENT	CDCl3
NS	16
DS	2
SWH	6009.615 Hz
FIDRES	0.091699 Hz
AQ	5.4525952 sec
RG	181
DW	83.200 usec
DE	6.50 usec
TE	-59.1 K
D1	1.00000000 sec
TD0	1

======== CHANNEL f1 ========

SFO1	300.1318534 MHz
NUC1	1H
P1	10.00 usec
PLW1	14.00000000 W

F2 - Processing parameters

SI	65536
SF	300.1300113 MHz
WDW	EM
SSB	0
LB	0.30 Hz
GB	0
PC	1.00

3ai

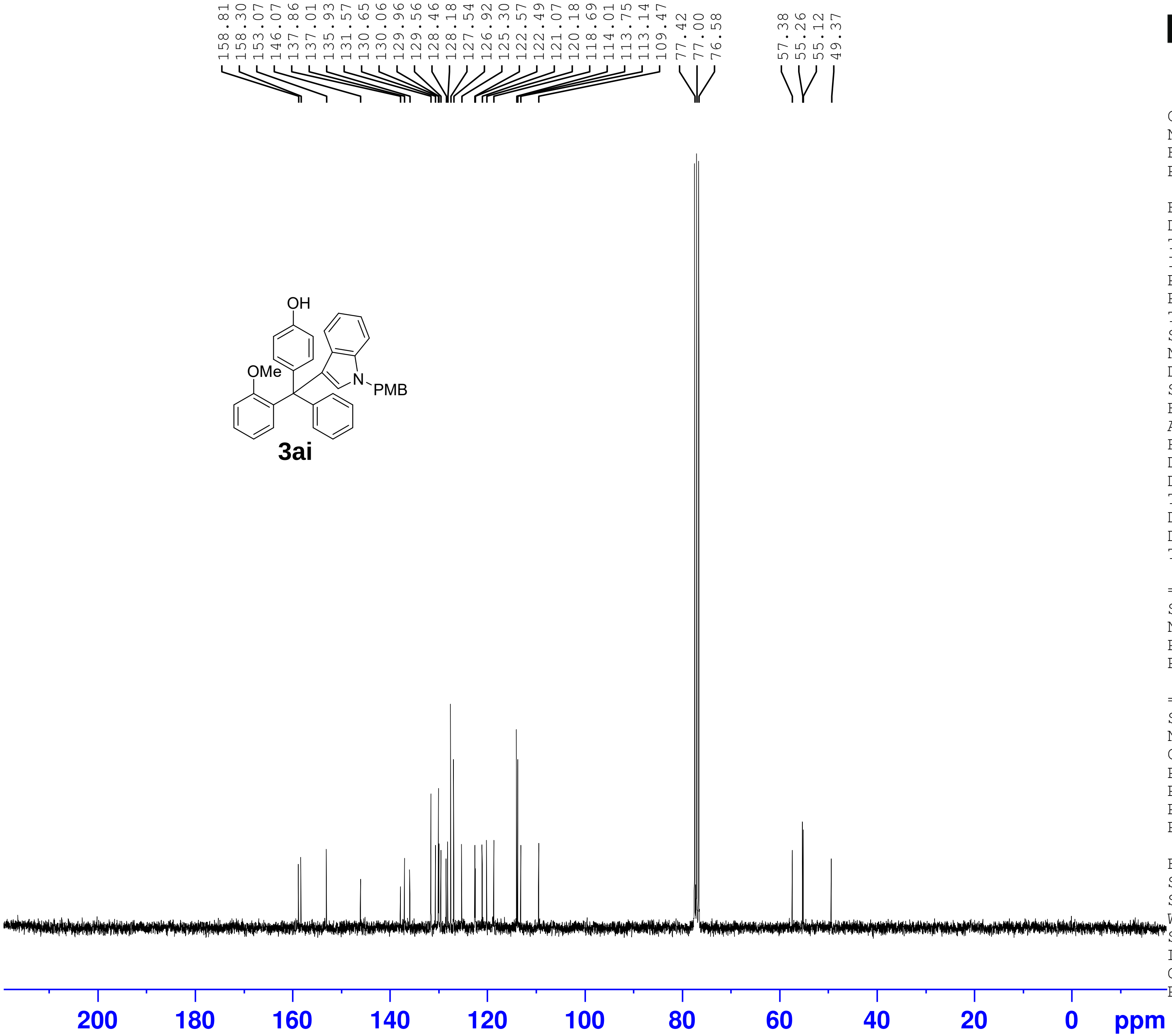

Current Data Parameters
NAME 3ai-ZY-4-64A
EXPNO 5339
PROCNO 1

F2 - Acquisition Parameters
Date_ 20210914
Time 13.39
INSTRUM spect
PROBHD 5 mm PABBO BB-
PULPROG zgpg30
TD 65536
SOLVENT CDCl3
NS 900
DS 4
SWH 18028.846 Hz
FIDRES 0.275098 Hz
AQ 1.8175317 sec
RG 203
DW 27.733 usec
DE 6.50 usec
TE -59.1 K
D1 2.00000000 sec
D11 0.03000000 sec
TD0 1

======== CHANNEL f1 ========
SFO1 75.4752949 MHz
NUC1 13C
P1 9.50 usec
PLW1 34.20000076 W

======== CHANNEL f2 ========
SFO2 300.1312005 MHz
NUC2 1H
CPDPRG[2 waltz16
PCPD2 90.00 usec
PLW2 14.00000000 W
PLW12 0.17284000 W
PLW13 0.14000000 W

F2 - Processing parameters
SI 32768
SF 75.4677526 MHz
WDW EM
SSB 0
LB 1.00 Hz
GB 0
PC 1.40

3ba

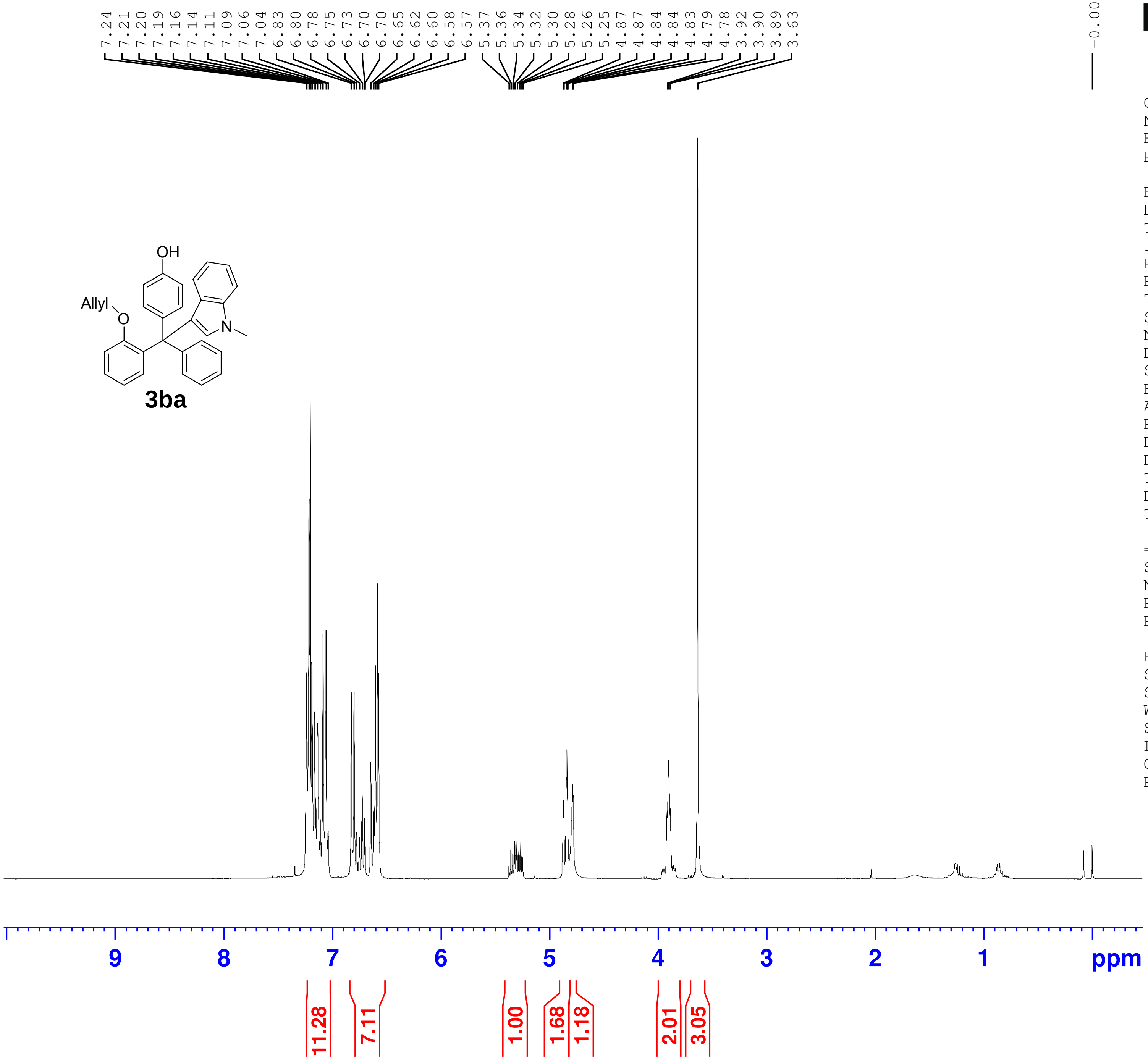

Current Data Parameters

NAME	ZY-4-11D-h-fr
EXPNO	5723
PROCNO	1

F2 - Acquisition Parameters

Date_	20211218
Time	9.11
INSTRUM	spect
PROBHD	5 mm PABBO BB-
PULPROG	zg30
TD	65536
SOLVENT	CDCl3
NS	16
DS	2
SWH	6009.615 Hz
FIDRES	0.091699 Hz
AQ	5.4525952 sec
RG	80.6
DW	83.200 usec
DE	6.50 usec
TE	-59.1 K
D1	1.00000000 sec
TD0	1

======== CHANNEL f1 ========

SFO1	300.1318534 MHz
NUC1	1H
P1	10.00 usec
PLW1	14.00000000 W

F2 - Processing parameters

SI	65536
SF	300.1300245 MHz
WDW	EM
SSB	0
LB	0.30 Hz
GB	0
PC	1.00

3ba

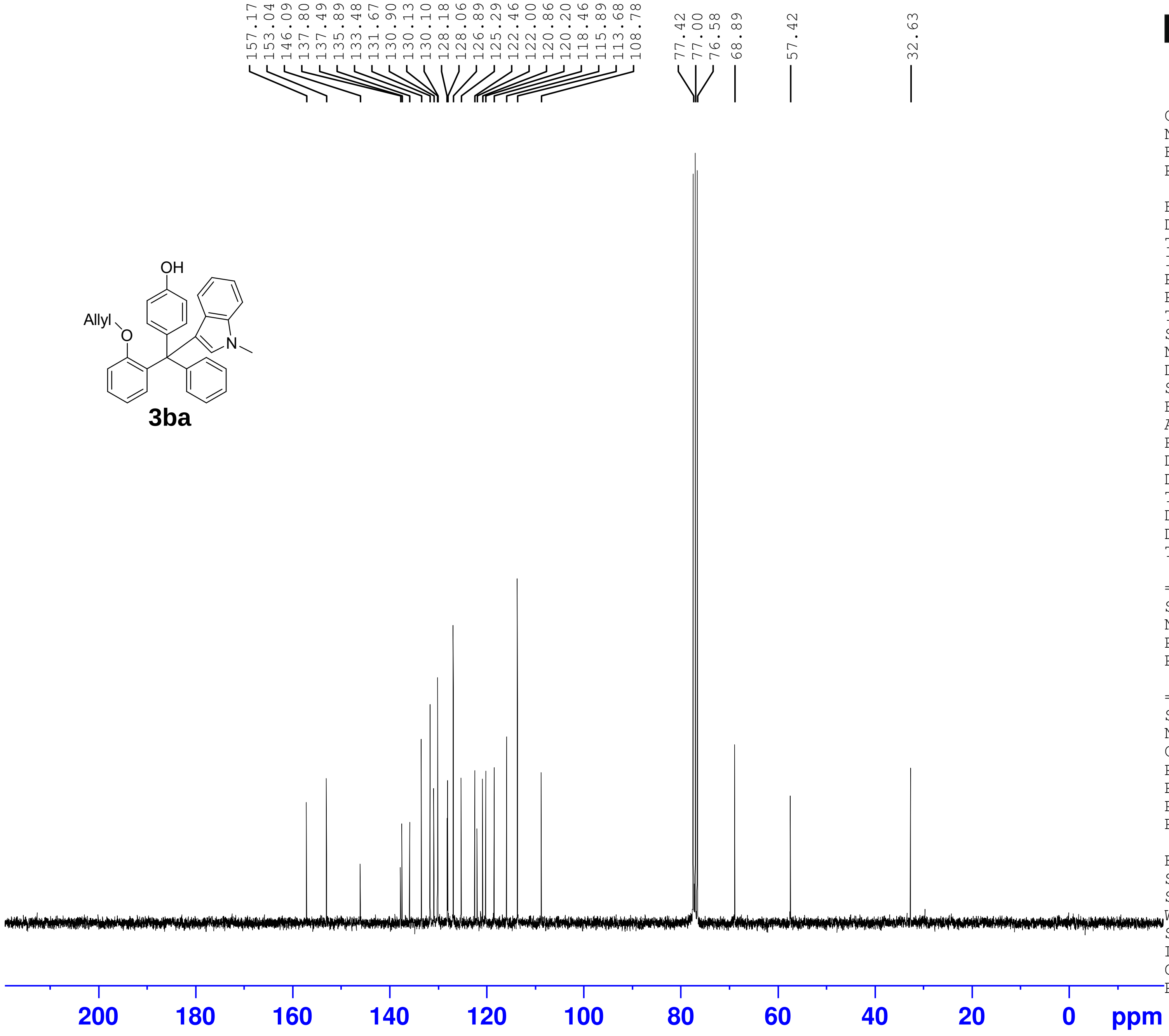

3ba

Current Data Parameters
NAME 3ca-ZY-4-11D
EXPNO 5513
PROCNO 1

F2 - Acquisition Parameters
Date_ 20211010
Time 11.17
INSTRUM spect
PROBHD 5 mm PABBO BB-
PULPROG zgpg30
TD 65536
SOLVENT CDCl3
NS 600
DS 4
SWH 18028.846 Hz
FIDRES 0.275098 Hz
AQ 1.8175317 sec
RG 203
DW 27.733 usec
DE 6.50 usec
TE -59.1 K
D1 2.00000000 sec
D11 0.03000000 sec
TD0 1

======== CHANNEL f1 ========
SFO1 75.4752949 MHz
NUC1 13C
P1 9.50 usec
PLW1 34.20000076 W

======== CHANNEL f2 ========
SFO2 300.1312005 MHz
NUC2 1H
CPDPRG[2 waltz16
PCPD2 90.00 usec
PLW2 14.00000000 W
PLW12 0.17284000 W
PLW13 0.14000000 W

F2 - Processing parameters
SI 32768
SF 75.4677539 MHz
WDW EM
SSB 0
LB 1.00 Hz
GB 0
PC 1.40

3ca

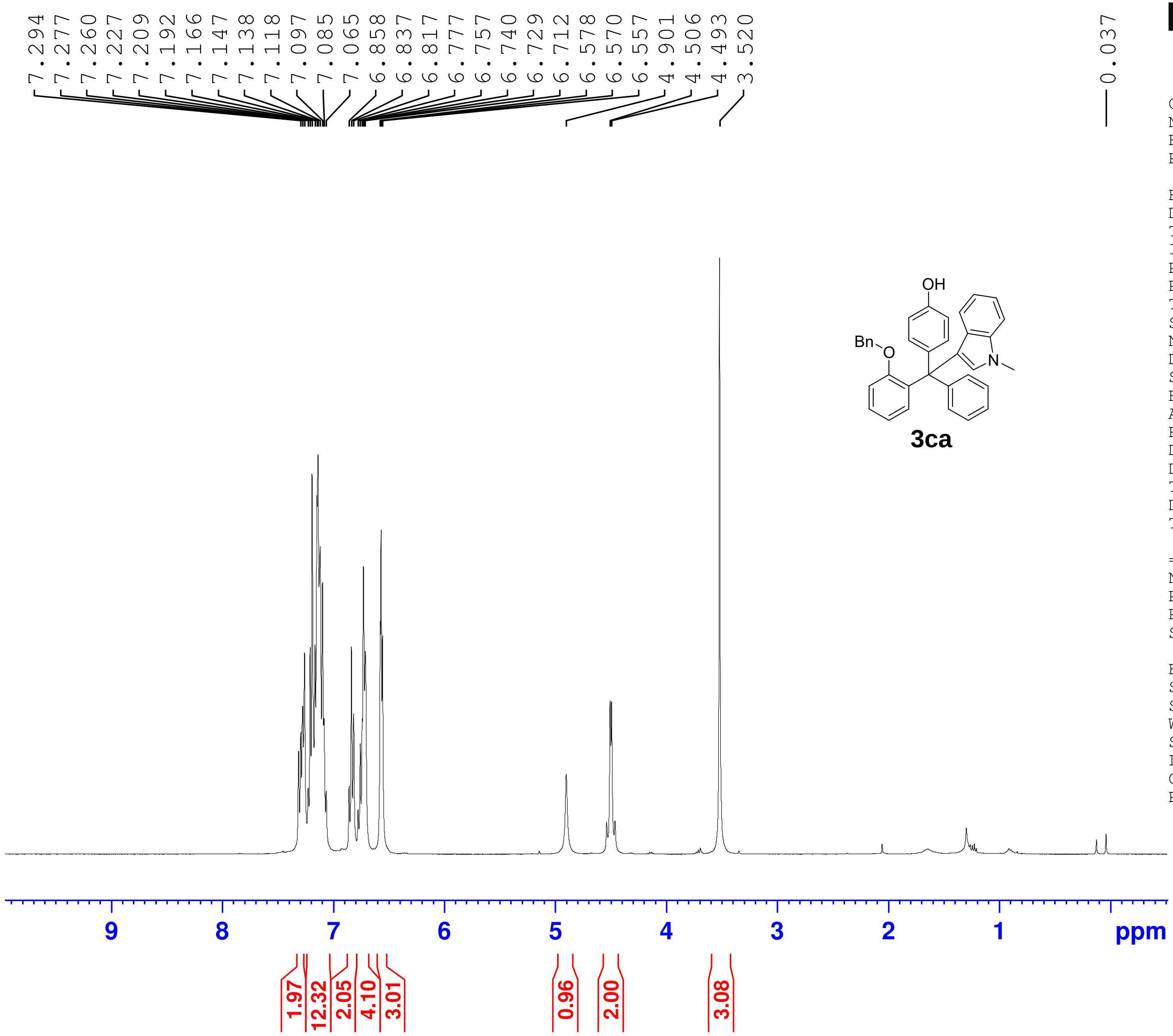

3ca

Current Data Parameters
NAME 211214-400
EXPNO 230
PROCNO 1

F2 - Acquisition Parameters
Date_ 20211214
Time 19.41
INSTRUM spect
PROBHD 5 mm PABBO BB-
PULPROG zg30
TD 65536
SOLVENT CDCl3
NS 8
DS 2
SWH 8223.685 Hz
FIDRES 0.125483 Hz
AQ 3.9845889 sec
RG 29.75
DW 60.800 usec
DE 6.50 usec
TE 292.4 K
D1 1.00000000 sec
TD0 1

======== CHANNEL f1 ========
NUC1 1H
P1 14.40 usec
PLW1 14.00000000 W
SFO1 400.1924713 MHz

F2 - Processing parameters
SI 65536
SF 400.1900442 MHz
WDW EM
SSB 0
LB 0.30 Hz
GB 0
PC 1.00

3ca

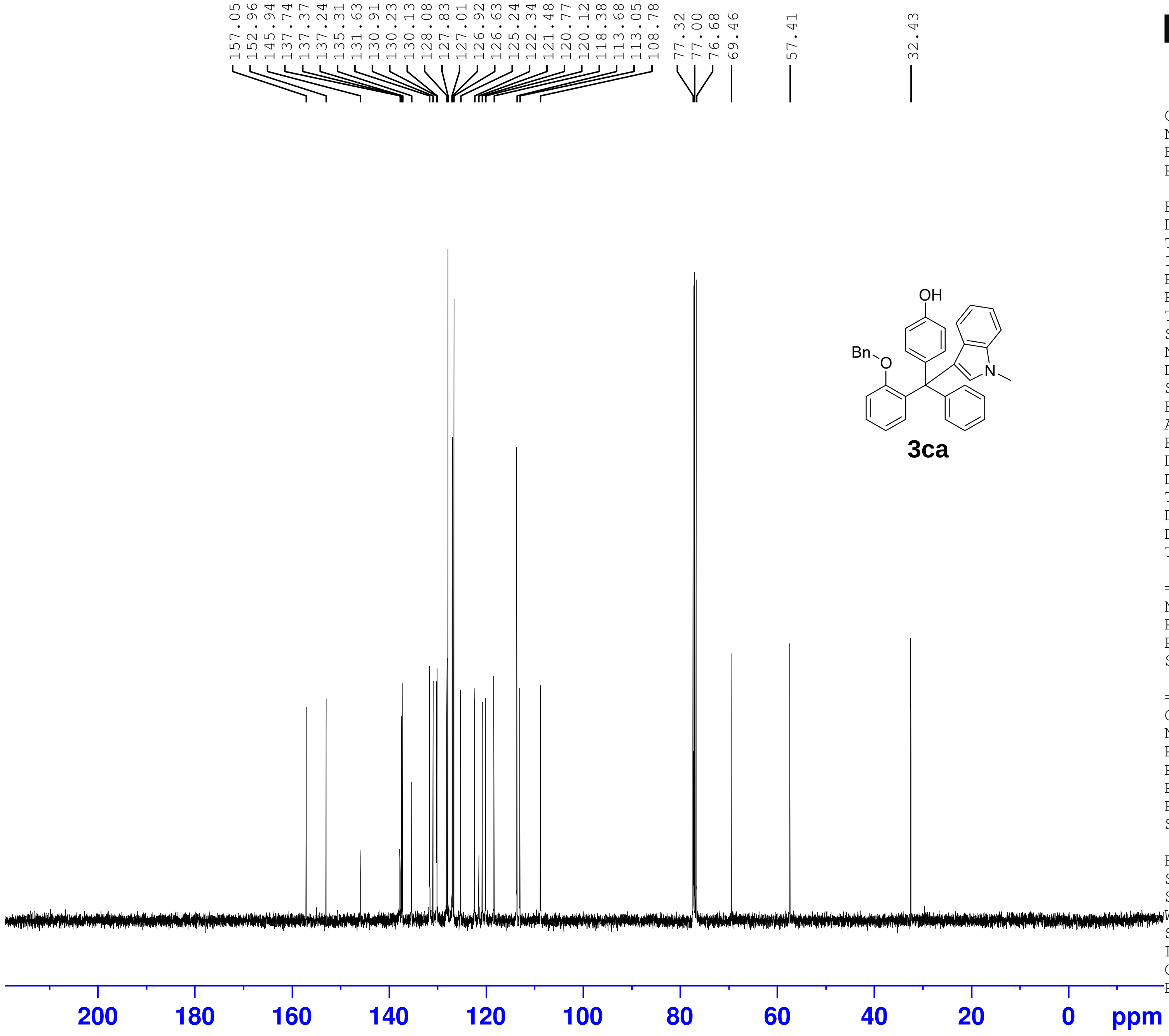

3ca

Current Data Parameters
NAME 3ba-ZY-4-11B
EXPNO 234
PROCNO 1

F2 - Acquisition Parameters
Date_ 20211214
Time 20.09
INSTRUM spect
PROBHD 5 mm PABBO BB-
PULPROG zgpg30
TD 65536
SOLVENT CDCl3
NS 112
DS 4
SWH 24038.461 Hz
FIDRES 0.366798 Hz
AQ 1.3631488 sec
RG 193.13
DW 20.800 usec
DE 6.50 usec
TE 293.2 K
D1 2.00000000 sec
D11 0.03000000 sec
TD0 1

======== CHANNEL f1 ========
NUC1 13C
P1 9.90 usec
PLW1 53.00000000 W
SFO1 100.6379178 MHz

======== CHANNEL f2 ========
CPDPRG[2 waltz16
NUC2 1H
PCPD2 90.00 usec
PLW2 14.00000000 W
PLW12 0.35839999 W
PLW13 0.29030001 W
SFO2 400.1916008 MHz

F2 - Processing parameters
SI 32768
SF 100.6278764 MHz
WDW EM
SSB 0
LB 1.00 Hz
GB 0
PC 1.40

3da

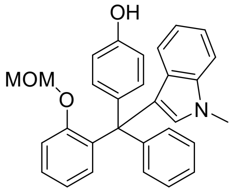

3da

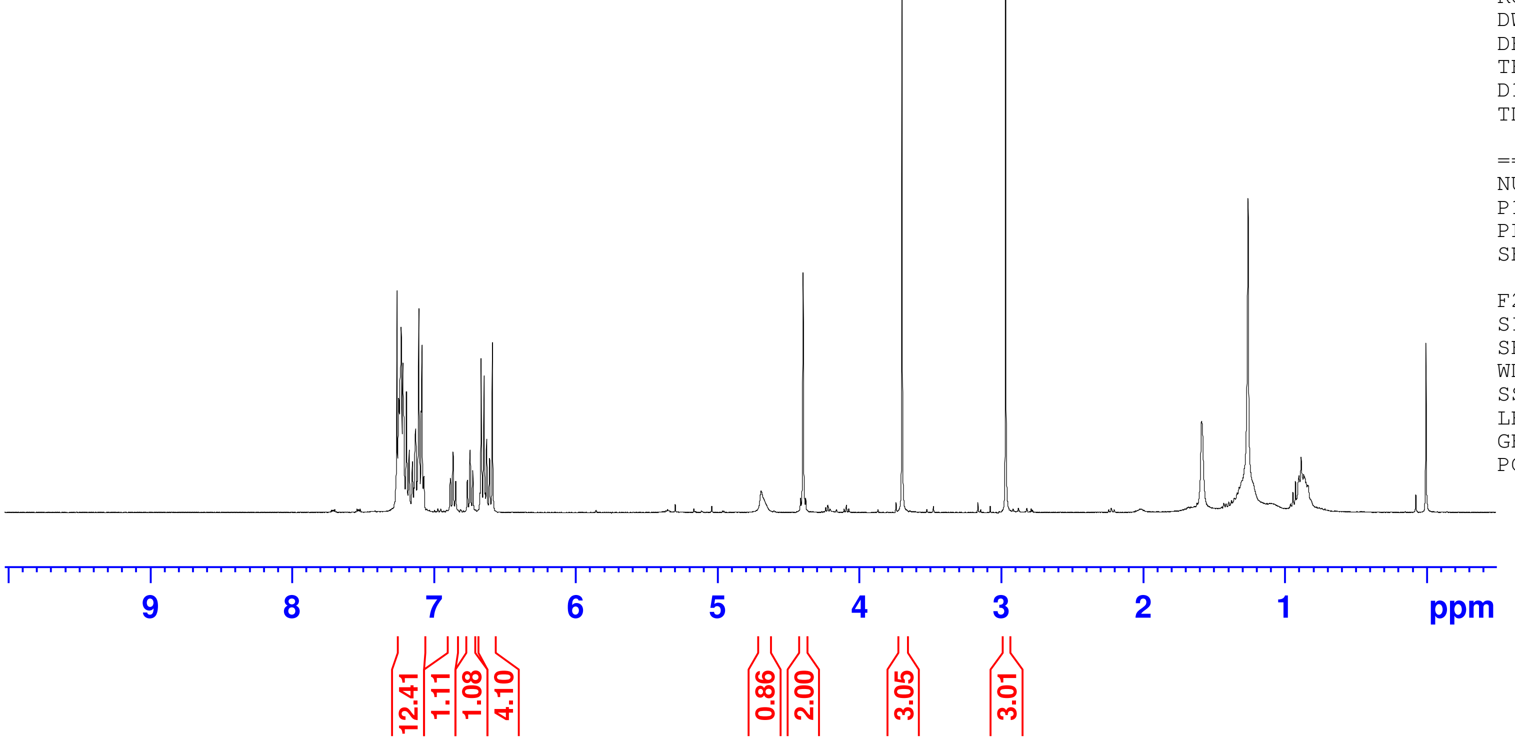

Current Data Parameters
NAME 0812-400
EXPNO 58
PROCNO 1

F2 - Acquisition Parameters
Date_ 20220812
Time 22.18
INSTRUM spect
PROBHD 5 mm PABBO BB-
PULPROG zg30
TD 65536
SOLVENT CDCl3
NS 8
DS 2
SWH 8223.685 Hz
FIDRES 0.125483 Hz
AQ 3.9845889 sec
RG 193.13
DW 60.800 usec
DE 6.50 usec
TE 294.6 K
D1 1.00000000 sec
TD0 1

======== CHANNEL f1 ========
NUC1 1H
P1 14.68 usec
PLW1 14.00000000 W
SFO1 400.1924713 MHz

F2 - Processing parameters
SI 65536
SF 400.1900165 MHz
WDW EM
SSB 0
LB 0.30 Hz
GB 0
PC 1.00

3da

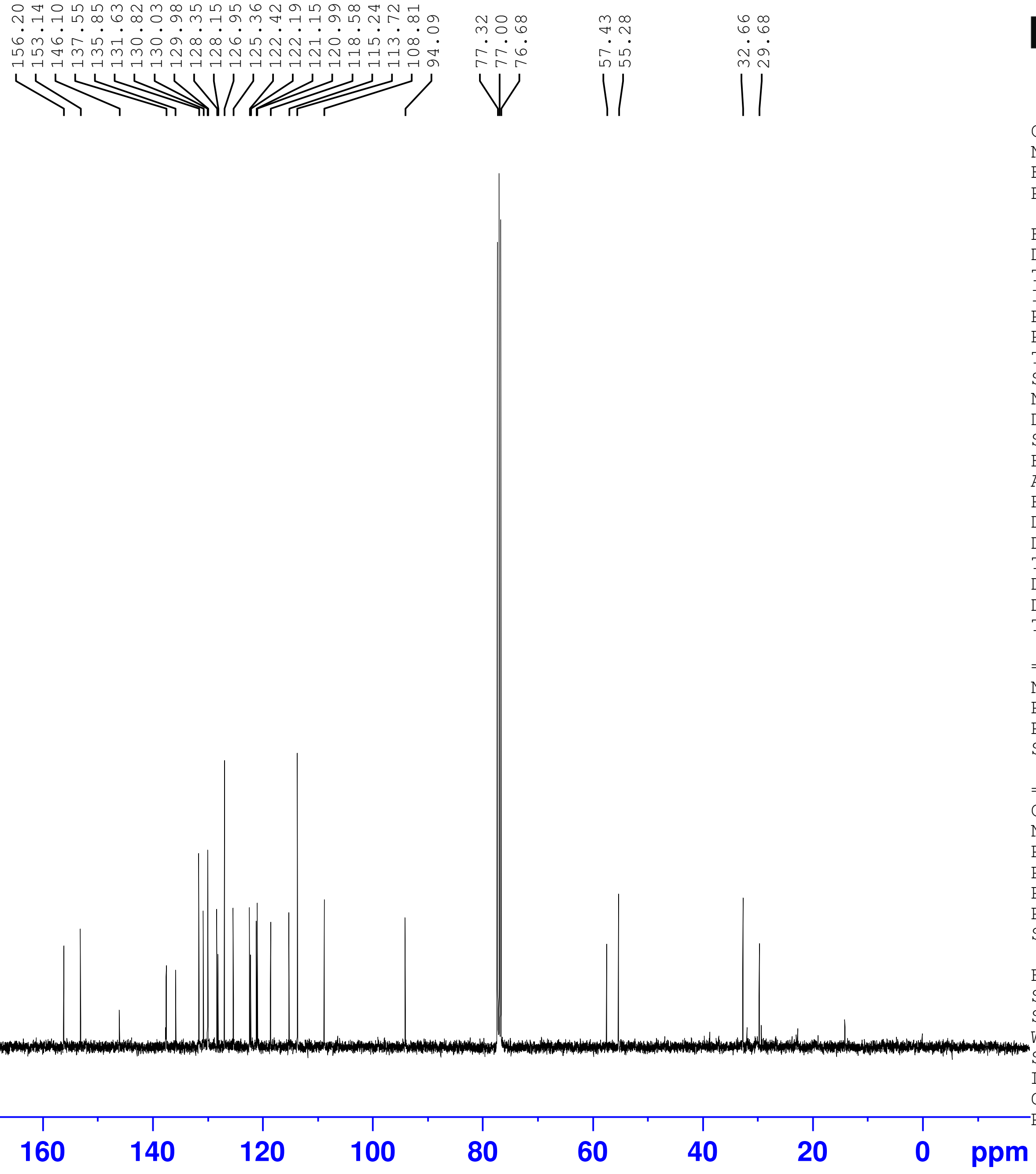

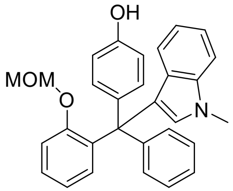

3da

Current Data Parameters
NAME 3da-ZB-1-50
EXPNO 1
PROCNO 1

F2 - Acquisition Parameters
Date_ 20220808
Time 22.41
INSTRUM spect
PROBHD 5 mm PABBO BB-
PULPROG zgpg30
TD 65536
SOLVENT CDCl3
NS 400
DS 4
SWH 24038.461 Hz
FIDRES 0.366798 Hz
AQ 1.3631488 sec
RG 193.13
DW 20.800 usec
DE 6.50 usec
TE 295.1 K
D1 2.00000000 sec
D11 0.03000000 sec
TD0 1

======== CHANNEL f1 ========
NUC1 13C
P1 12.00 usec
PLW1 53.00000000 W
SFO1 100.6379178 MHz

======== CHANNEL f2 ========
CPDPRG[2 waltz16
NUC2 1H
PCPD2 90.00 usec
PLW2 14.00000000 W
PLW12 0.37246999 W
PLW13 0.30170000 W
SFO2 400.1916008 MHz

F2 - Processing parameters
SI 32768
SF 100.6278628 MHz
WDW EM
SSB 0
LB 1.00 Hz
GB 0
PC 1.40

3ea

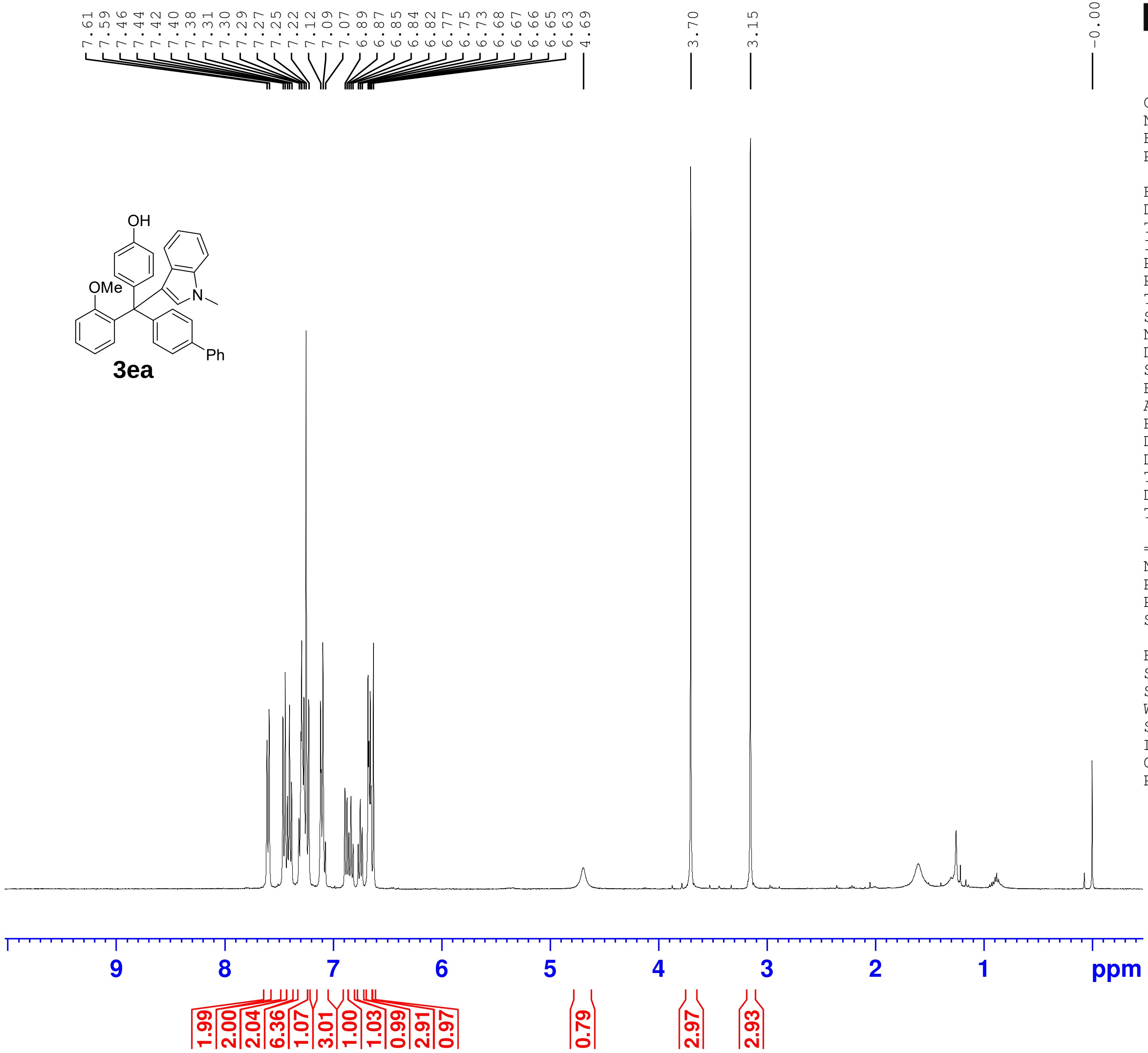

Current Data Parameters
NAME ZY-4-75F-h-fr
EXPNO 281
PROCNO 1

F2 - Acquisition Parameters
Date_ 20211223
Time 19.58
INSTRUM spect
PROBHD 5 mm PABBO BB-
PULPROG zg30
TD 65536
SOLVENT CDCl3
NS 8
DS 2
SWH 8223.685 Hz
FIDRES 0.125483 Hz
AQ 3.9845889 sec
RG 154.68
DW 60.800 usec
DE 6.50 usec
TE 292.4 K
D1 1.00000000 sec
TD0 1

======== CHANNEL f1 ========
NUC1 1H
P1 14.40 usec
PLW1 14.00000000 W
SFO1 400.1924713 MHz

F2 - Processing parameters
SI 65536
SF 400.1900203 MHz
WDW EM
SSB 0
LB 0.30 Hz
GB 0
PC 1.00

3ea

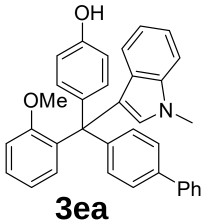

3ea

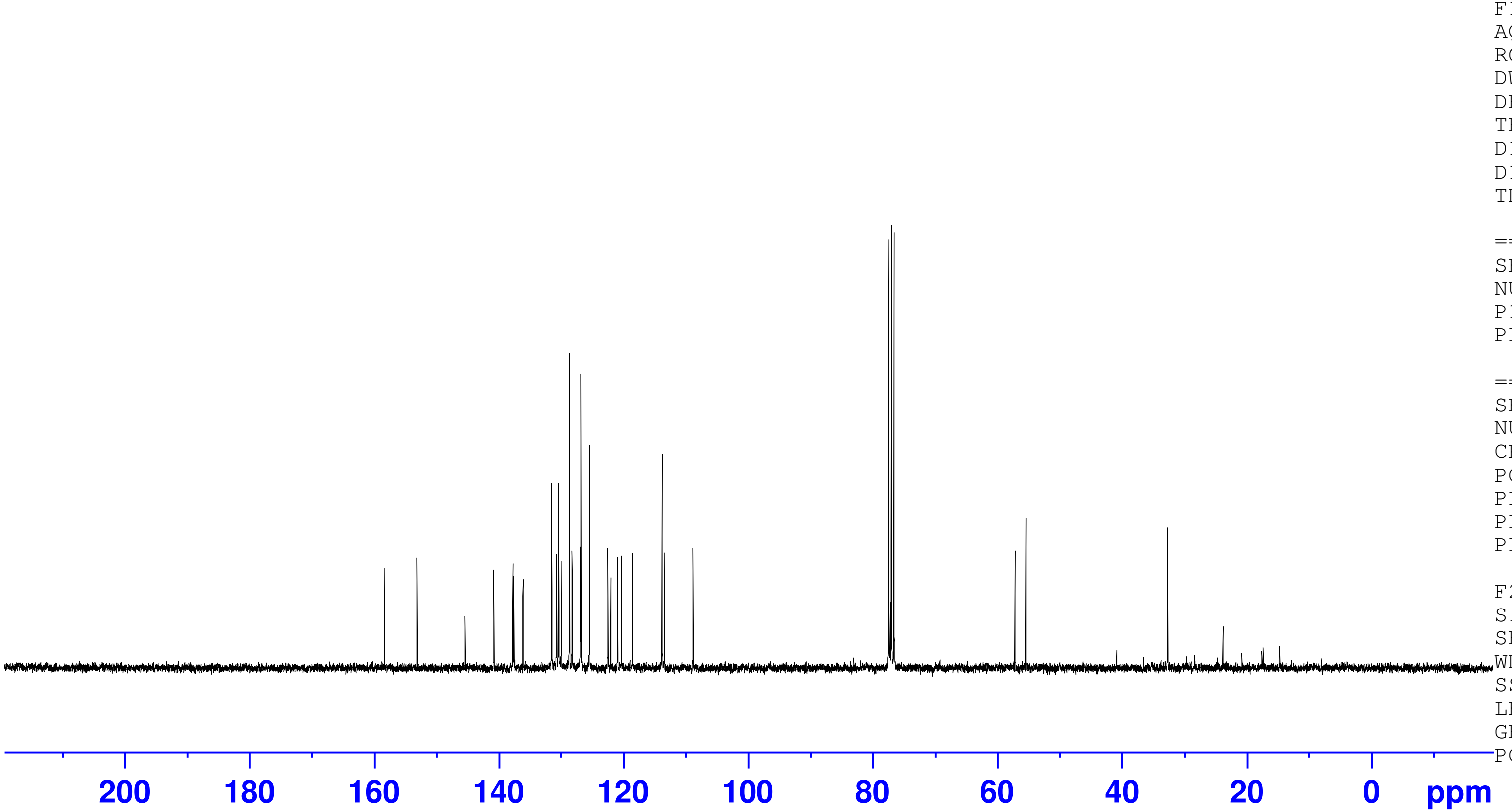

Current Data Parameters	
NAME	3ea-ZY-4-75F
EXPNO	5617
PROCNO	1
**F2 - Acquisition Parameters**	
Date_	20211118
Time	10.01
INSTRUM	spect
PROBHD	5 mm PABBO BB-
PULPROG	zgpg30
TD	65536
SOLVENT	CDCl3
NS	500
DS	4
SWH	18028.846 Hz
FIDRES	0.275098 Hz
AQ	1.8175317 sec
RG	203
DW	27.733 usec
DE	6.50 usec
TE	-59.1 K
D1	2.00000000 sec
D11	0.03000000 sec
TD0	1
======== CHANNEL f1 ========	
SFO1	75.4752949 MHz
NUC1	13C
P1	9.50 usec
PLW1	34.20000076 W
======== CHANNEL f2 ========	
SFO2	300.1312005 MHz
NUC2	1H
CPDPRG[2	waltz16
PCPD2	90.00 usec
PLW2	14.00000000 W
PLW12	0.17284000 W
PLW13	0.14000000 W
**F2 - Processing parameters**	
SI	32768
SF	75.4677568 MHz
WDW	EM
SSB	0
LB	1.00 Hz
GB	0
PC	1.40

3fa

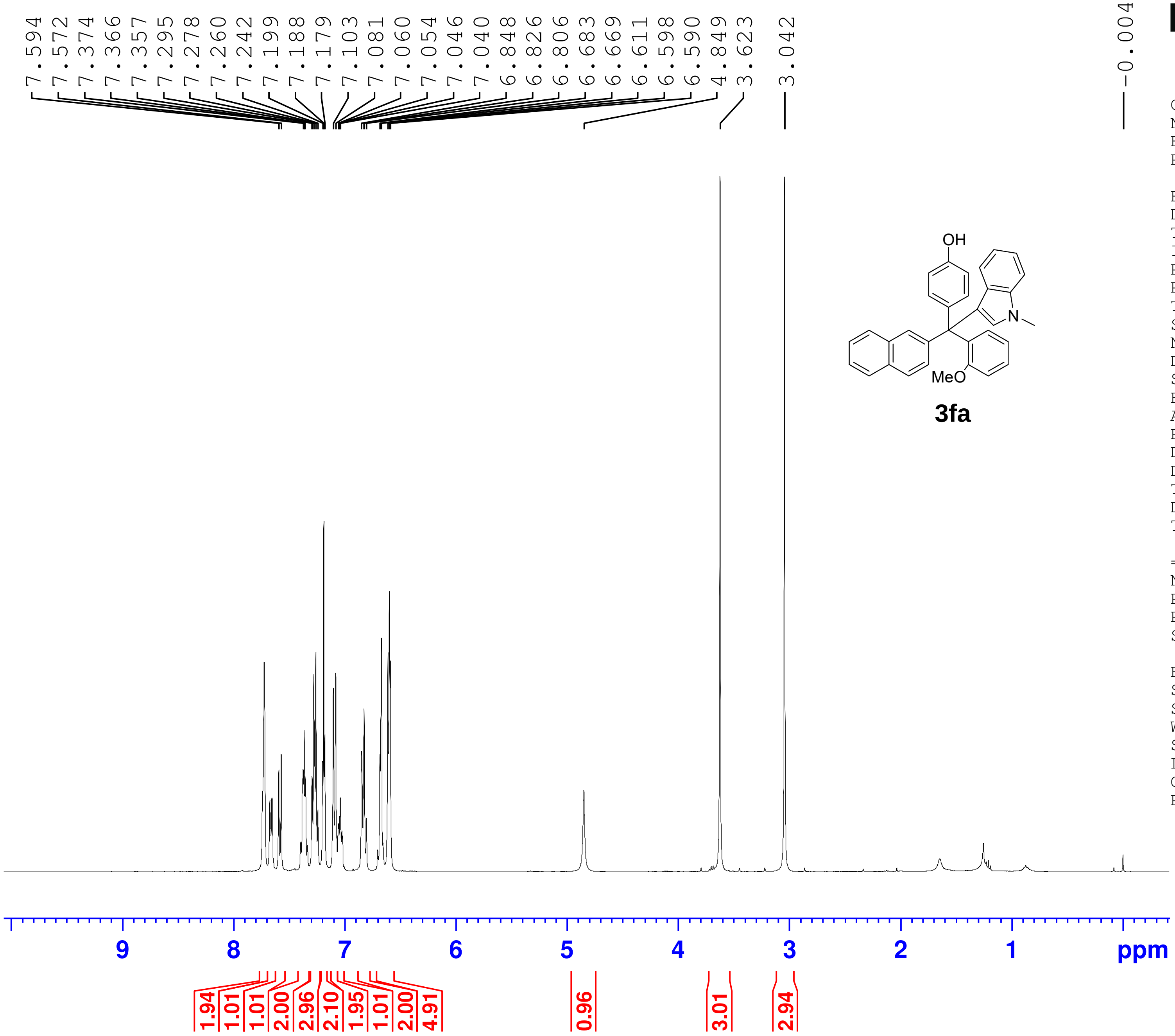

3fa

BRUKER

Current Data Parameters
NAME 211223-400
EXPNO 279
PROCNO 1

F2 - Acquisition Parameters
Date_ 20211223
Time 19.49
INSTRUM spect
PROBHD 5 mm PABBO BB-
PULPROG zg30
TD 65536
SOLVENT CDCl3
NS 6
DS 2
SWH 8223.685 Hz
FIDRES 0.125483 Hz
AQ 3.9845889 sec
RG 53.3
DW 60.800 usec
DE 6.50 usec
TE 292.3 K
D1 1.00000000 sec
TD0 1

======== CHANNEL f1 ========
NUC1 1H
P1 14.40 usec
PLW1 14.00000000 W
SFO1 400.1924713 MHz

F2 - Processing parameters
SI 65536
SF 400.1900447 MHz
WDW EM
SSB 0
LB 0.30 Hz
GB 0
PC 1.00

3fa

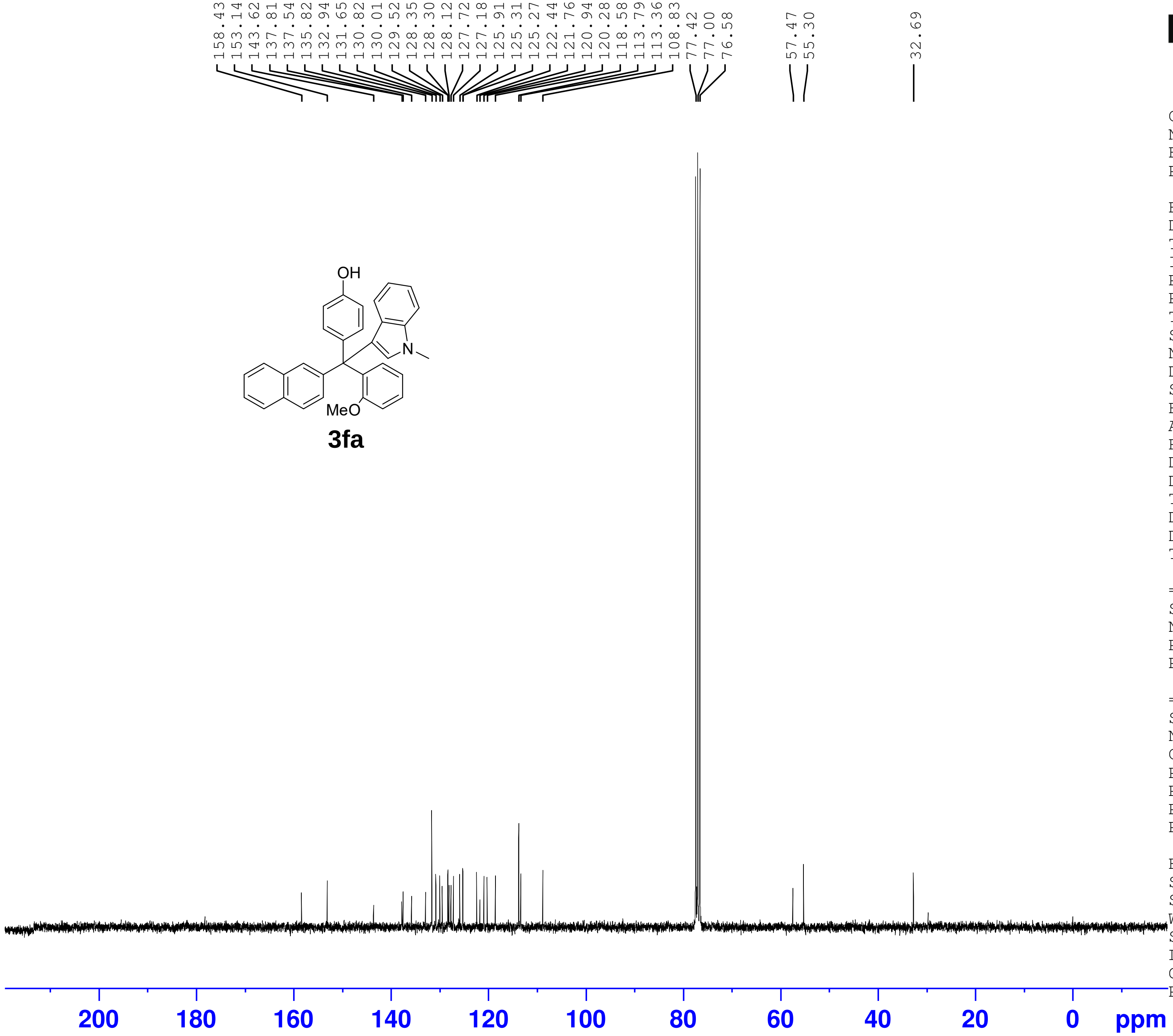

Current Data Parameters
NAME 3fa-ZY-4-11A
EXPNO 4252
PROCNO 1

F2 - Acquisition Parameters
Date_ 20210623
Time 11.59
INSTRUM spect
PROBHD 5 mm PABBO BB-
PULPROG zgpg30
TD 65536
SOLVENT CDCl3
NS 1024
DS 4
SWH 18028.846 Hz
FIDRES 0.275098 Hz
AQ 1.8175317 sec
RG 203
DW 27.733 usec
DE 6.50 usec
TE 296.2 K
D1 2.00000000 sec
D11 0.03000000 sec
TD0 1

======== CHANNEL f1 ========
SFO1 75.4752949 MHz
NUC1 13C
P1 9.50 usec
PLW1 34.20000076 W

======== CHANNEL f2 ========
SFO2 300.1312005 MHz
NUC2 1H
CPDPRG[2 waltz16
PCPD2 90.00 usec
PLW2 14.00000000 W
PLW12 0.17284000 W
PLW13 0.14000000 W

F2 - Processing parameters
SI 32768
SF 75.4677518 MHz
WDW EM
SSB 0
LB 1.00 Hz
GB 0
PC 1.40

3ga

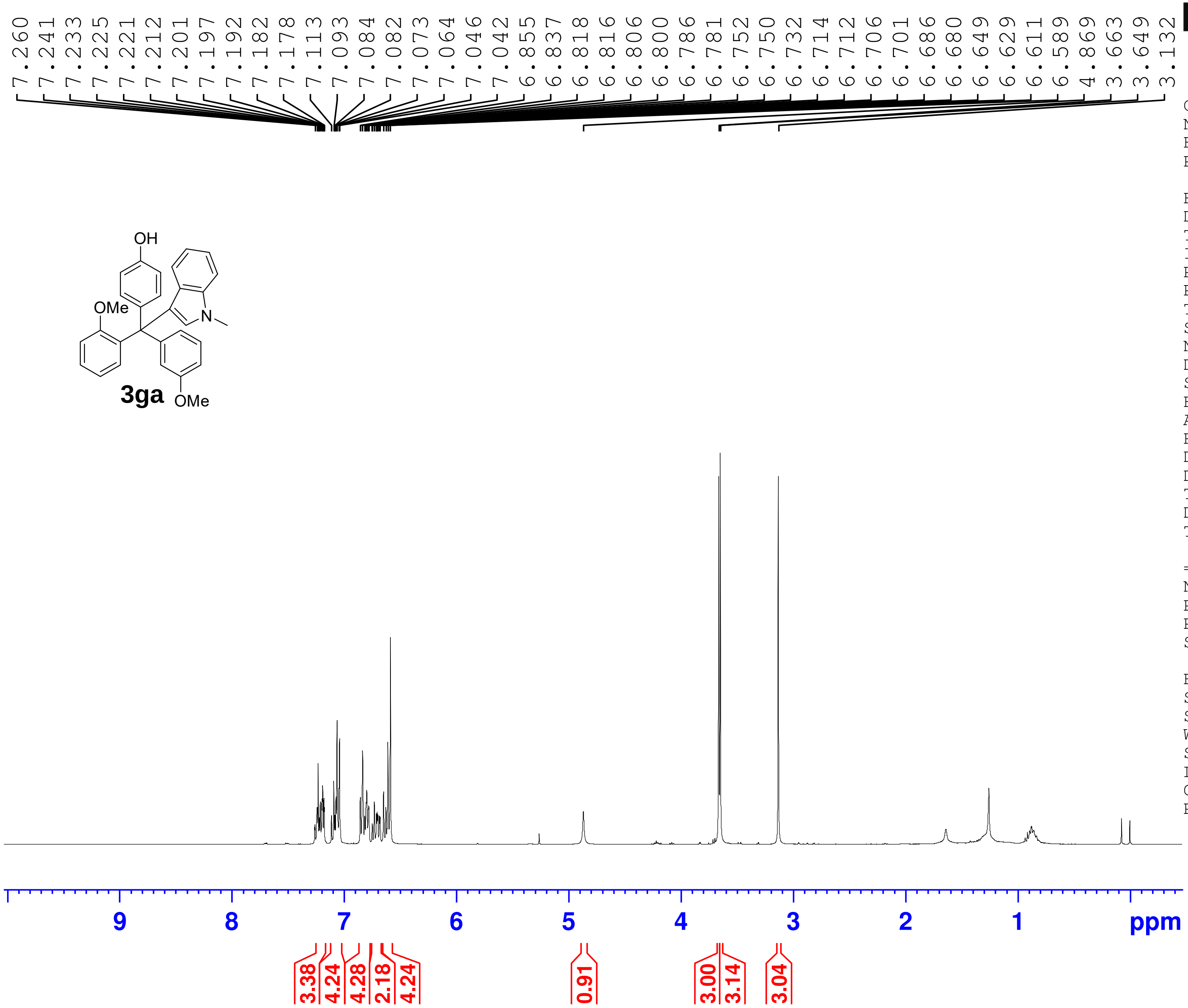

BRUKER

Current Data Parameters
NAME 0812-400
EXPNO 69
PROCNO 1

F2 - Acquisition Parameters
Date_ 20220812
Time 23.29
INSTRUM spect
PROBHD 5 mm PABBO BB-
PULPROG zg30
TD 65536
SOLVENT CDCl3
NS 8
DS 2
SWH 8223.685 Hz
FIDRES 0.125483 Hz
AQ 3.9845889 sec
RG 90.23
DW 60.800 usec
DE 6.50 usec
TE 294.7 K
D1 1.00000000 sec
TD0 1

======== CHANNEL f1 ========
NUC1 1H
P1 14.68 usec
PLW1 14.00000000 W
SFO1 400.1924713 MHz

F2 - Processing parameters
SI 65536
SF 400.1900270 MHz
WDW EM
SSB 0
LB 0.30 Hz
GB 0
PC 1.00

3ga

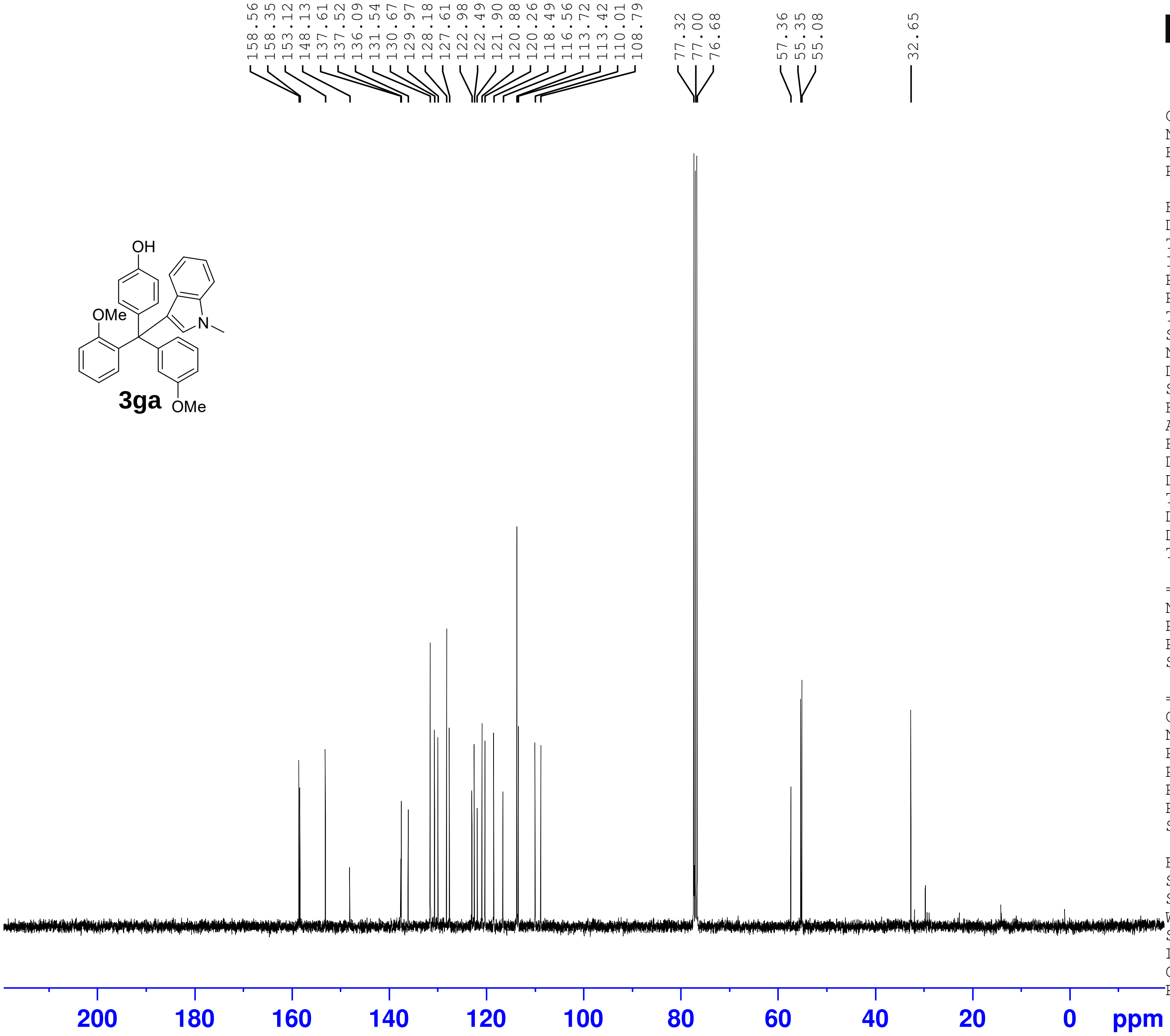

Current Data Parameters
NAME 3ga-ZB-1-77
EXPNO 1
PROCNO 1

F2 - Acquisition Parameters
Date_ 20220812
Time 23.47
INSTRUM spect
PROBHD 5 mm PABBO BB-
PULPROG zgpg30
TD 65536
SOLVENT CDCl3
NS 300
DS 4
SWH 24038.461 Hz
FIDRES 0.366798 Hz
AQ 1.3631488 sec
RG 193.13
DW 20.800 usec
DE 6.50 usec
TE 295.4 K
D1 2.00000000 sec
D11 0.03000000 sec
TD0 1

======== CHANNEL f1 ========
NUC1 13C
P1 12.00 usec
PLW1 53.00000000 W
SFO1 100.6379178 MHz

======== CHANNEL f2 ========
CPDPRG[2 waltz16
NUC2 1H
PCPD2 90.00 usec
PLW2 14.00000000 W
PLW12 0.37246999 W
PLW13 0.30170000 W
SFO2 400.1916008 MHz

F2 - Processing parameters
SI 32768
SF 100.6278644 MHz
WDW EM
SSB 0
LB 1.00 Hz
GB 0
PC 1.40

3ha

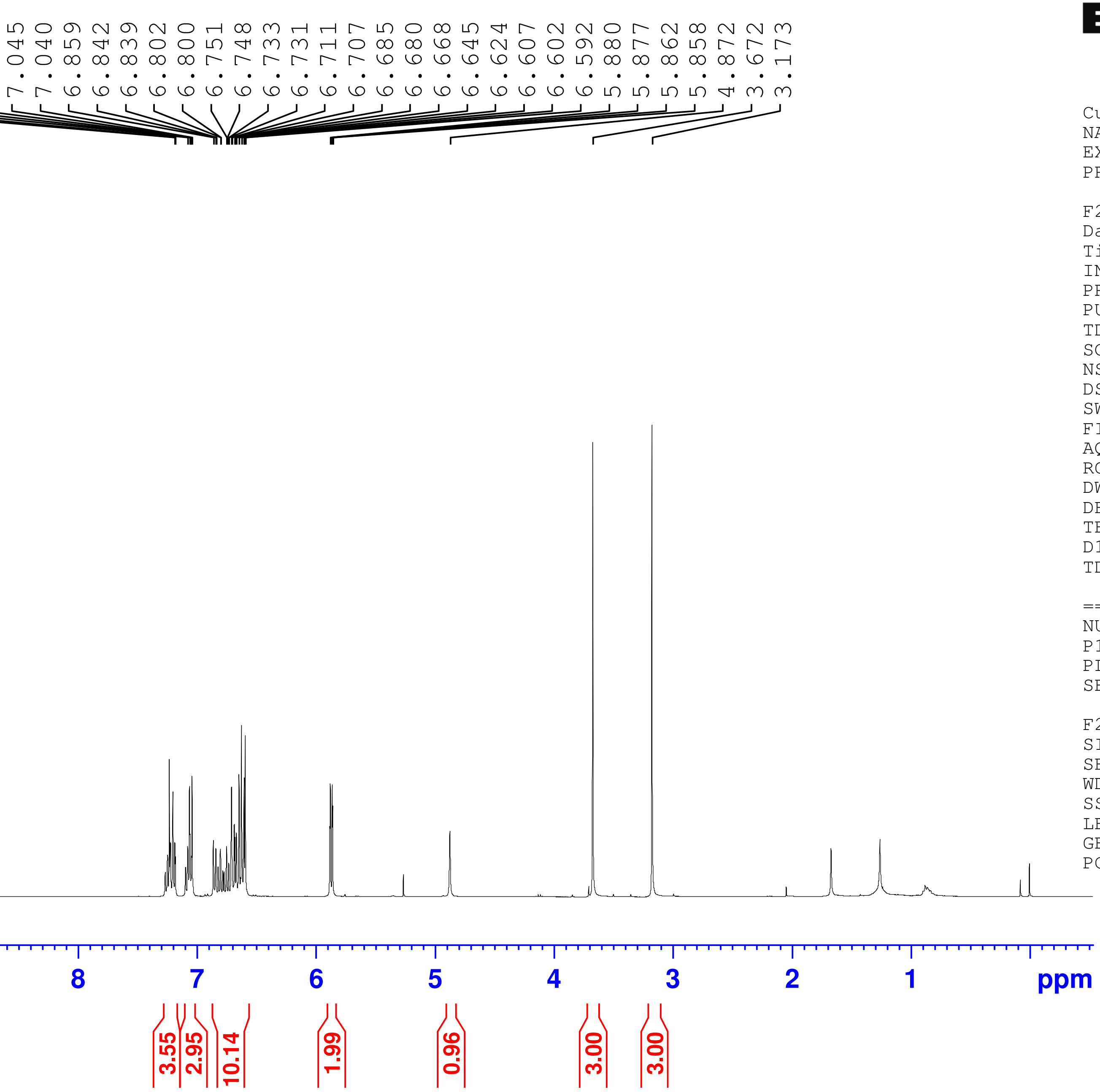

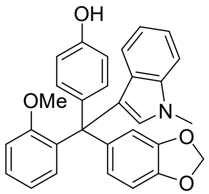

3ha

Current Data Parameters
NAME 0812-400
EXPNO 61
PROCNO 1

F2 - Acquisition Parameters
Date_ 20220812
Time 22.31
INSTRUM spect
PROBHD 5 mm PABBO BB-
PULPROG zg30
TD 65536
SOLVENT CDCl3
NS 8
DS 2
SWH 8223.685 Hz
FIDRES 0.125483 Hz
AQ 3.9845889 sec
RG 90.23
DW 60.800 usec
DE 6.50 usec
TE 294.6 K
D1 1.00000000 sec
TD0 1

======== CHANNEL f1 ========
NUC1 1H
P1 14.68 usec
PLW1 14.00000000 W
SFO1 400.1924713 MHz

F2 - Processing parameters
SI 65536
SF 400.1900277 MHz
WDW EM
SSB 0
LB 0.30 Hz
GB 0
PC 1.00

3ha

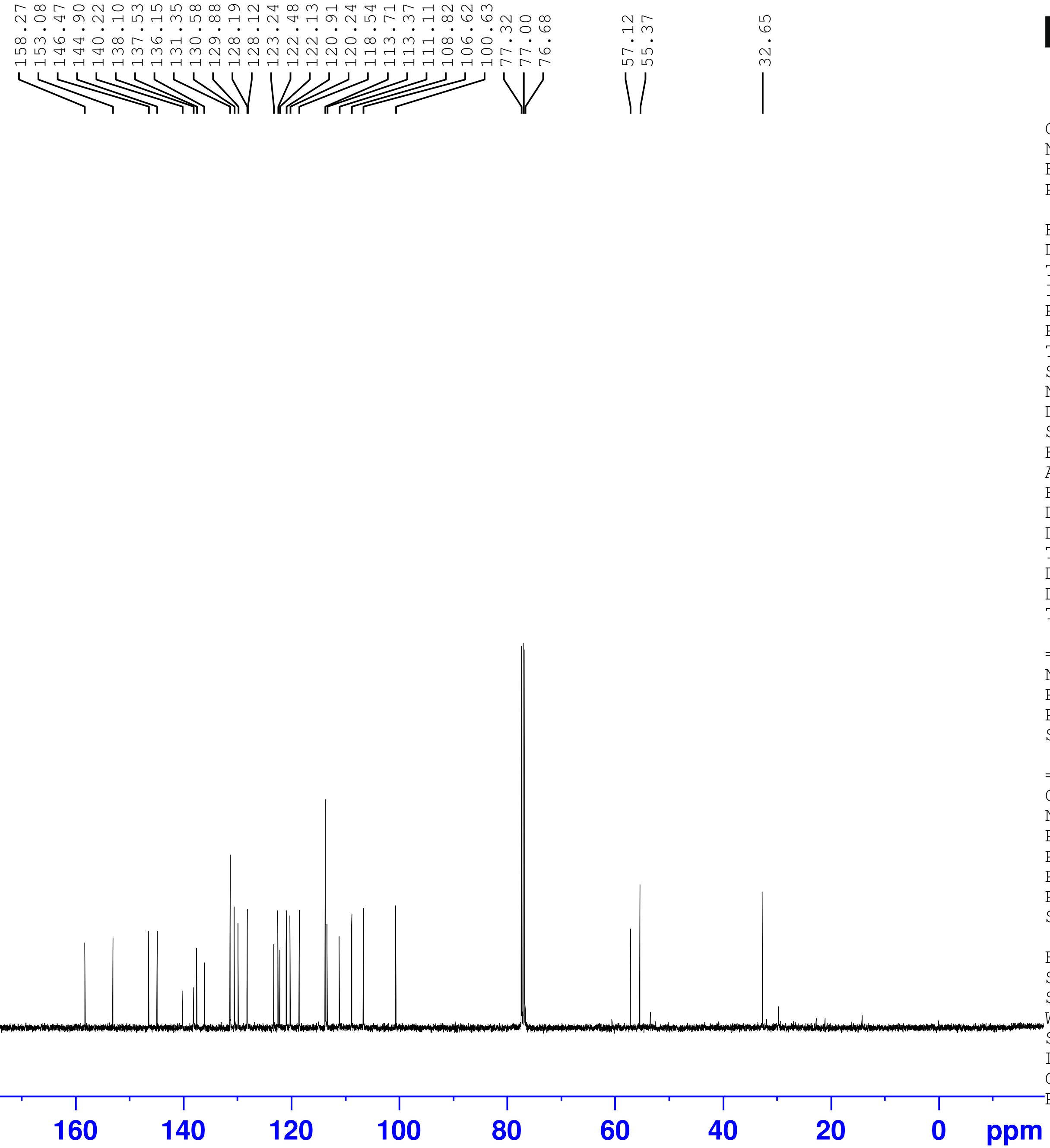

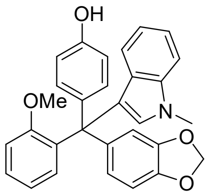

3ha

Current Data Parameters
NAME 0811-400-2
EXPNO 35
PROCNO 1

F2 - Acquisition Parameters
Date_ 20220812
Time 0.58
INSTRUM spect
PROBHD 5 mm PABBO BB-
PULPROG zgpg30
TD 65536
SOLVENT CDCl3
NS 300
DS 4
SWH 24038.461 Hz
FIDRES 0.366798 Hz
AQ 1.3631488 sec
RG 193.13
DW 20.800 usec
DE 6.50 usec
TE 295.0 K
D1 2.00000000 sec
D11 0.03000000 sec
TD0 1

======== CHANNEL f1 ========
NUC1 13C
P1 12.00 usec
PLW1 53.00000000 W
SFO1 100.6379178 MHz

======== CHANNEL f2 ========
CPDPRG[2 waltz16
NUC2 1H
PCPD2 90.00 usec
PLW2 14.00000000 W
PLW12 0.37246999 W
PLW13 0.30170000 W
SFO2 400.1916008 MHz

F2 - Processing parameters
SI 32768
SF 100.6278652 MHz
WDW EM
SSB 0
LB 1.00 Hz
GB 0
PC 1.40

3ia

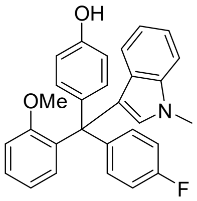

3ia

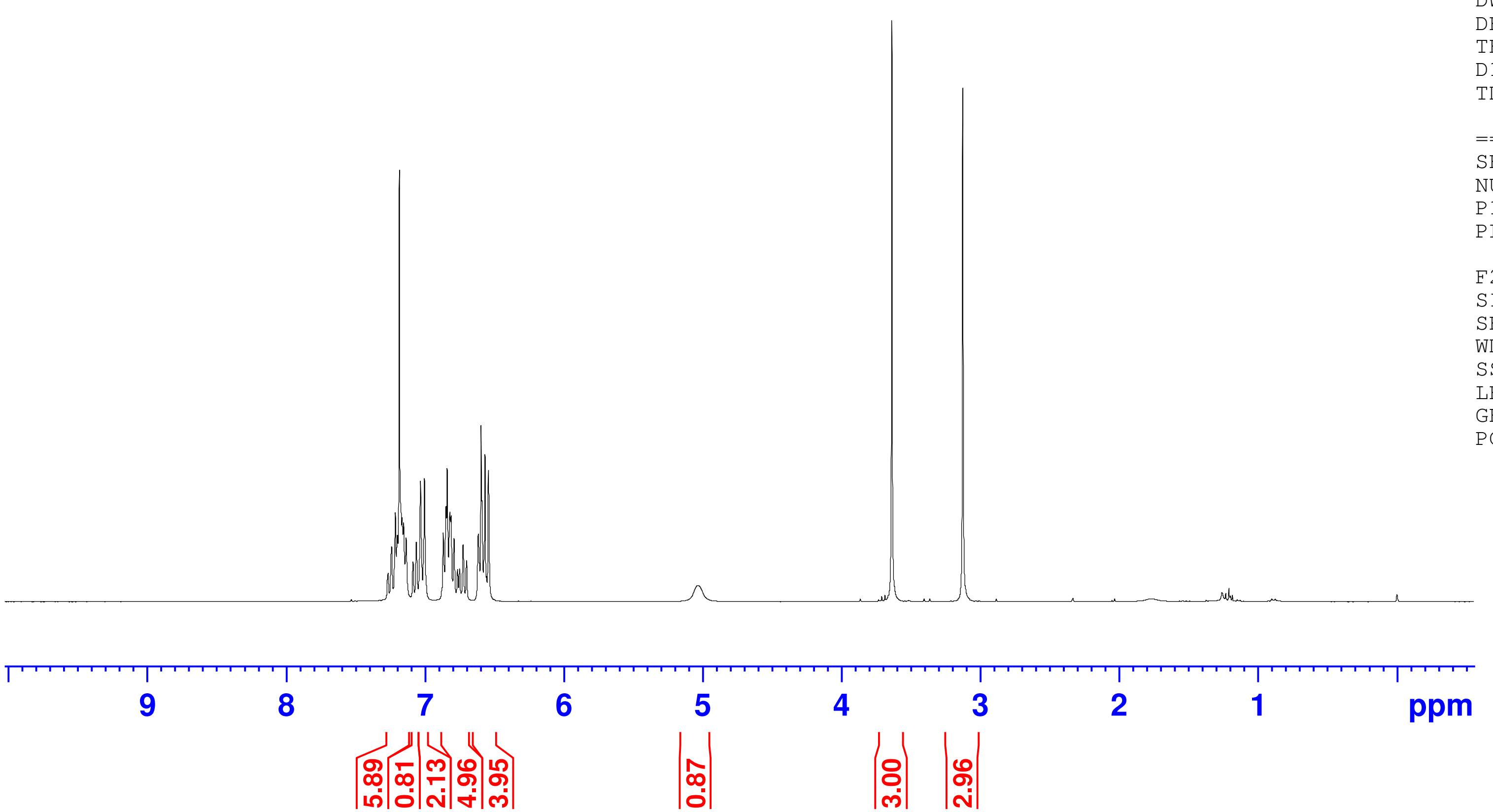

Current Data Parameters
NAME ZY-4-75B-h-fr
EXPNO 5623
PROCNO 1

F2 - Acquisition Parameters
Date_ 20211117
Time 12.31
INSTRUM spect
PROBHD 5 mm PABBO BB-
PULPROG zg30
TD 65536
SOLVENT CDCl3
NS 16
DS 2
SWH 6009.615 Hz
FIDRES 0.091699 Hz
AQ 5.4525952 sec
RG 57
DW 83.200 usec
DE 6.50 usec
TE -59.1 K
D1 1.00000000 sec
TD0 1

======== CHANNEL f1 ========
SFO1 300.1318534 MHz
NUC1 1H
P1 10.00 usec
PLW1 14.00000000 W

F2 - Processing parameters
SI 65536
SF 300.1300298 MHz
WDW EM
SSB 0
LB 0.30 Hz
GB 0
PC 1.00

3ia

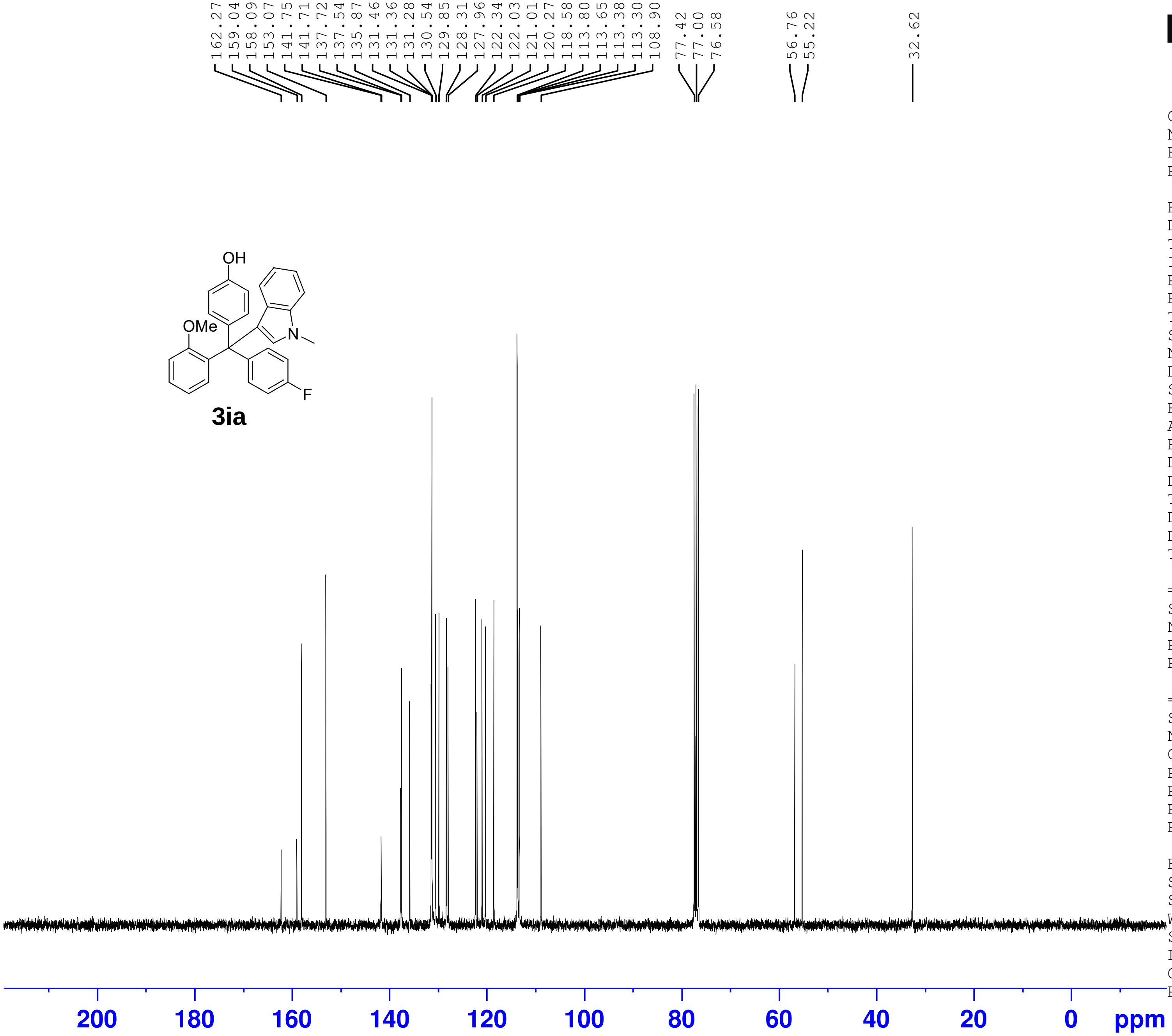

3ia

BRUKER

```
Current Data Parameters
NAME          3ia-ZY-4-75B
EXPNO                 5624
PROCNO                   1

F2 - Acquisition Parameters
Date_             20211117
Time                 13.05
INSTRUM              spect
PROBHD     5 mm PABBO BB-
PULPROG            zgpg30
TD                   65536
SOLVENT              CDCl3
NS                     500
DS                       4
SWH             18028.846 Hz
FIDRES           0.275098 Hz
AQ              1.8175317 sec
RG                     203
DW                  27.733 usec
DE                    6.50 usec
TE                   -59.1 K
D1              2.00000000 sec
D11             0.03000000 sec
TD0                      1

======== CHANNEL f1 ========
SFO1           75.4752949 MHz
NUC1                   13C
P1                    9.50 usec
PLW1           34.20000076 W

======== CHANNEL f2 ========
SFO2          300.1312005 MHz
NUC2                    1H
CPDPRG[2           waltz16
PCPD2                90.00 usec
PLW2           14.00000000 W
PLW12           0.17284000 W
PLW13           0.14000000 W

F2 - Processing parameters
SI                   32768
SF             75.4677597 MHz
WDW                     EM
SSB                      0
LB                    1.00 Hz
GB                       0
PC                    1.40
```

3ia

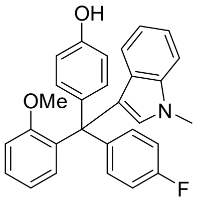

3ia

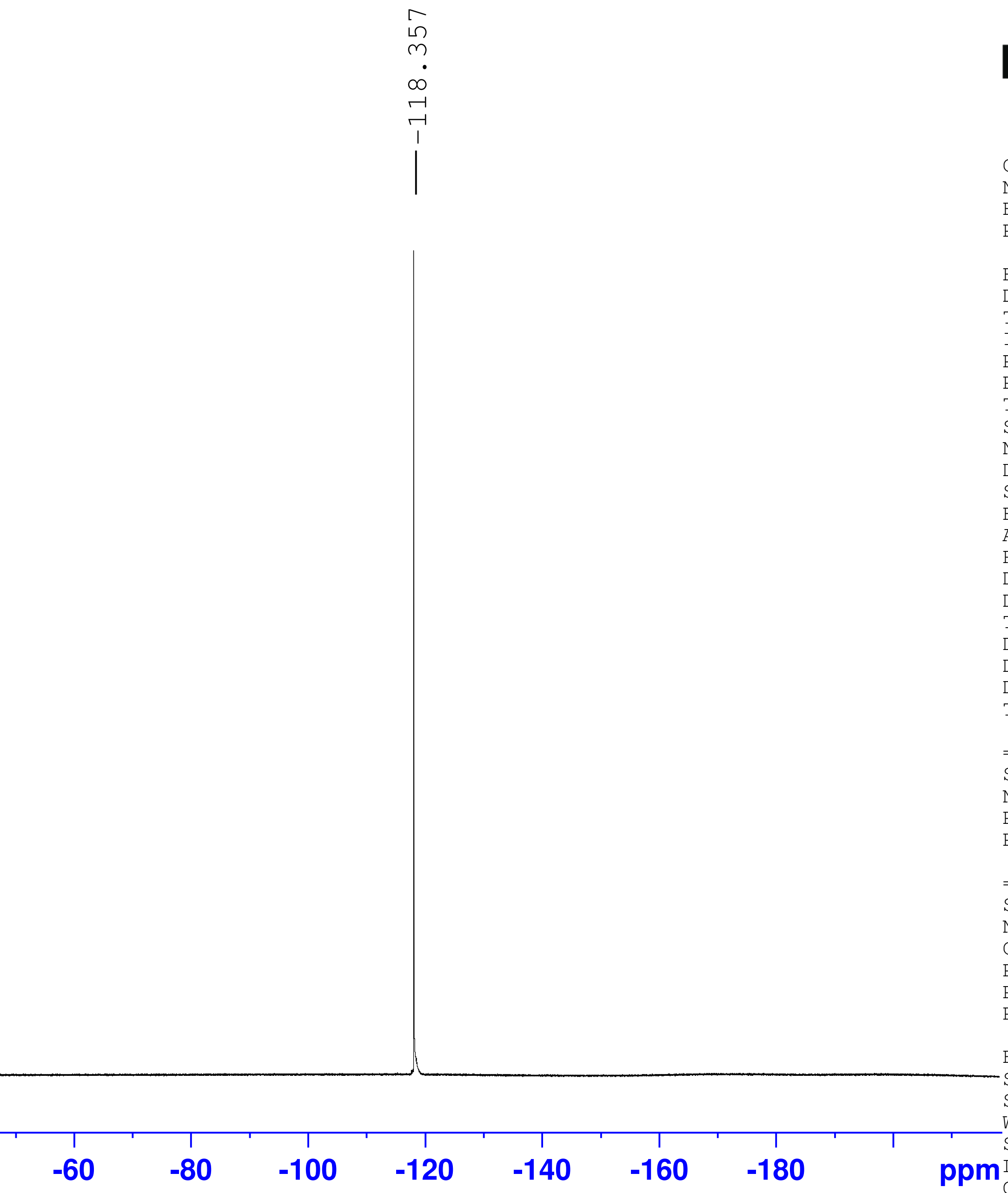

Current Data Parameters
NAME 0927sjw
EXPNO 5451
PROCNO 1

F2 - Acquisition Parameters
Date_ 20210927
Time 11.06
INSTRUM spect
PROBHD 5 mm PABBO BB-
PULPROG zgfhigqn.2
TD 131072
SOLVENT CDCl3
NS 16
DS 4
SWH 66964.289 Hz
FIDRES 0.510897 Hz
AQ 0.9786710 sec
RG 203
DW 7.467 usec
DE 6.50 usec
TE -59.1 K
D1 1.00000000 sec
D11 0.03000000 sec
D12 0.00002000 sec
TD0 1

======== CHANNEL f1 ========
SFO1 282.3761148 MHz
NUC1 19F
P1 14.50 usec
PLW1 10.39999962 W

======== CHANNEL f2 ========
SFO2 300.1312005 MHz
NUC2 1H
CPDPRG[2 waltz16
PCPD2 90.00 usec
PLW2 14.00000000 W
PLW12 0.17284000 W

F2 - Processing parameters
SI 65536
SF 282.4043552 MHz
WDW EM
SSB 0
LB 0.30 Hz
GB 0
PC 1.00

3ja

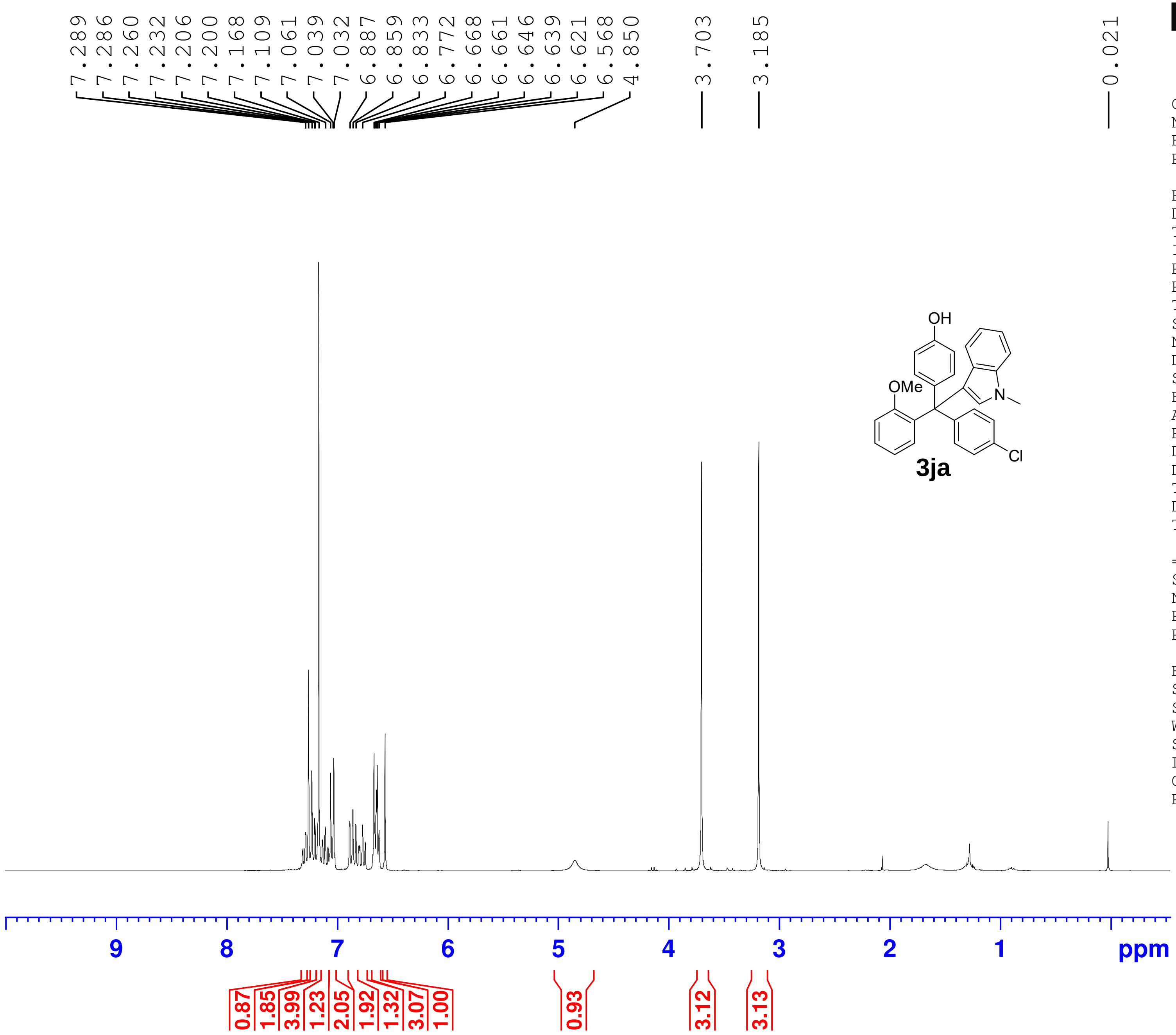

BRUKER

Current Data Parameters
NAME 20211030
EXPNO 5493
PROCNO 1

F2 - Acquisition Parameters
Date_ 20210930
Time 10.19
INSTRUM spect
PROBHD 5 mm PABBO BB-
PULPROG zg30
TD 65536
SOLVENT CDCl3
NS 16
DS 2
SWH 6009.615 Hz
FIDRES 0.091699 Hz
AQ 5.4525952 sec
RG 128
DW 83.200 usec
DE 6.50 usec
TE -59.1 K
D1 1.00000000 sec
TD0 1

======== CHANNEL f1 ========
SFO1 300.1318534 MHz
NUC1 1H
P1 10.00 usec
PLW1 14.00000000 W

F2 - Processing parameters
SI 65536
SF 300.1300072 MHz
WDW EM
SSB 0
LB 0.30 Hz
GB 0
PC 1.00

3ja

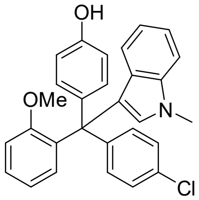

3ja

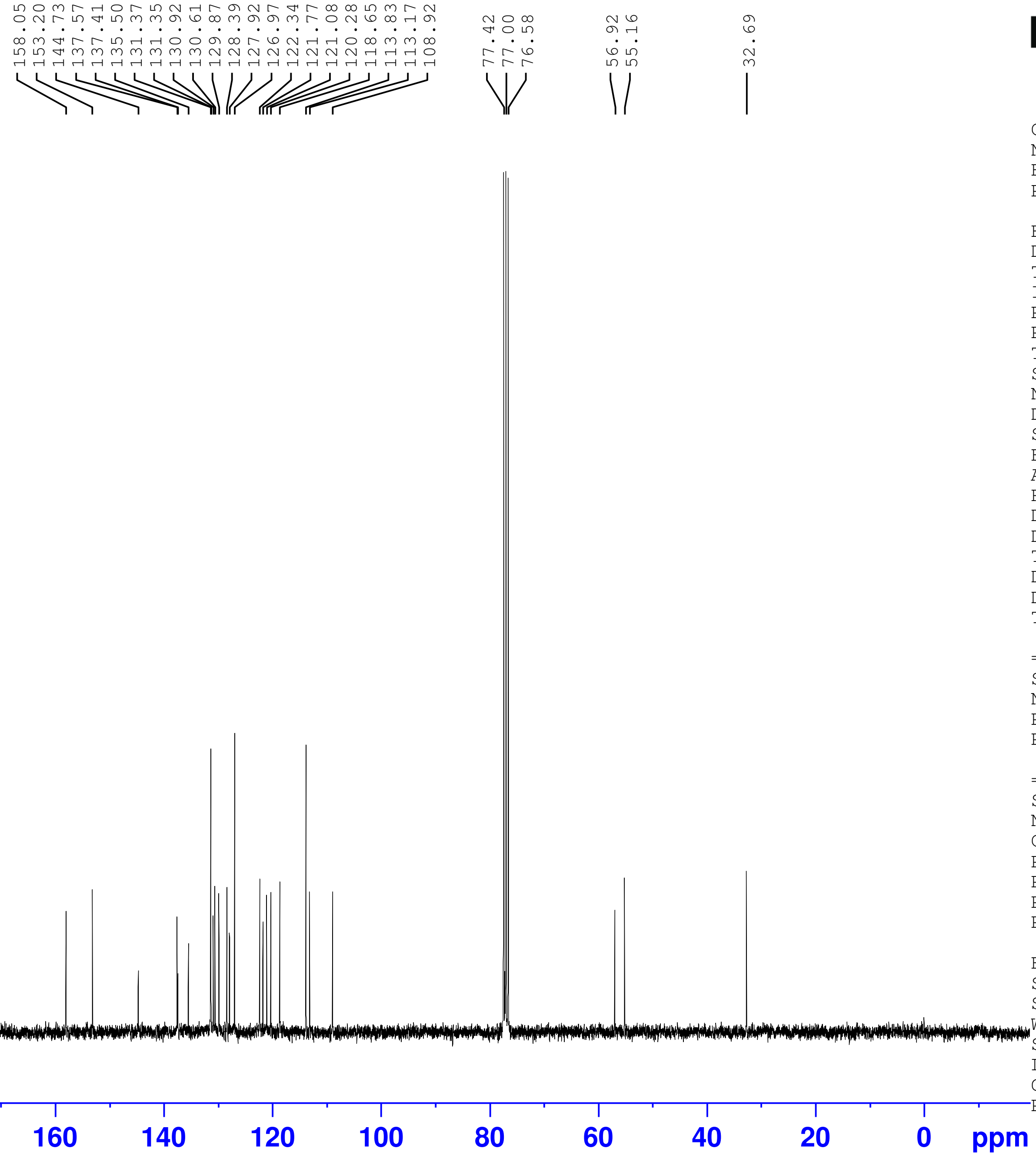

Current Data Parameters
NAME 3ja-ZY-4-75C
EXPNO 5494
PROCNO 1

F2 - Acquisition Parameters
Date_ 20210930
Time 10.52
INSTRUM spect
PROBHD 5 mm PABBO BB-
PULPROG zgpg30
TD 65536
SOLVENT CDCl3
NS 500
DS 4
SWH 18028.846 Hz
FIDRES 0.275098 Hz
AQ 1.8175317 sec
RG 203
DW 27.733 usec
DE 6.50 usec
TE -59.1 K
D1 2.00000000 sec
D11 0.03000000 sec
TD0 1

======== CHANNEL f1 ========
SFO1 75.4752949 MHz
NUC1 13C
P1 9.50 usec
PLW1 34.20000076 W

======== CHANNEL f2 ========
SFO2 300.1312005 MHz
NUC2 1H
CPDPRG[2 waltz16
PCPD2 90.00 usec
PLW2 14.00000000 W
PLW12 0.17284000 W
PLW13 0.14000000 W

F2 - Processing parameters
SI 32768
SF 75.4677536 MHz
WDW EM
SSB 0
LB 1.00 Hz
GB 0
PC 1.40

3ka

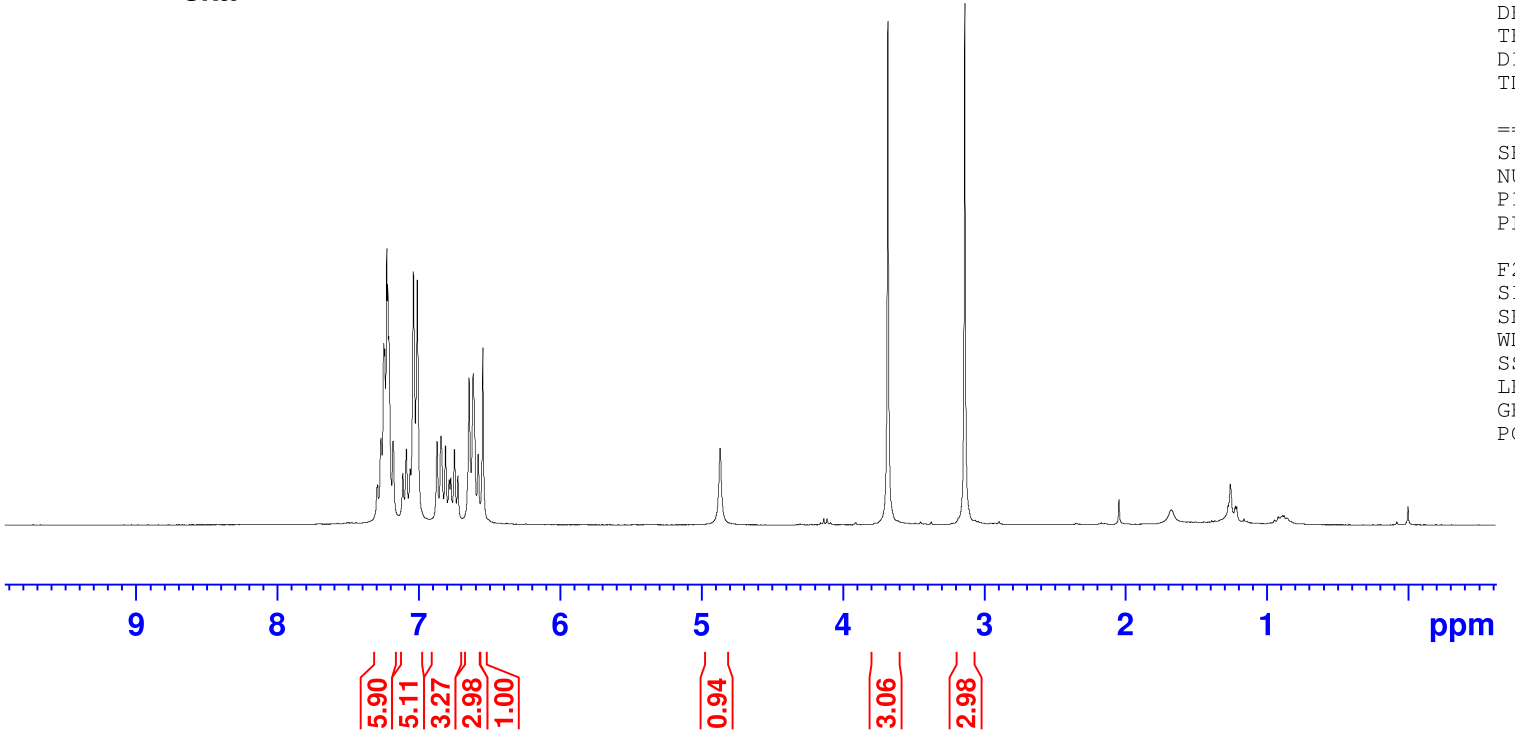

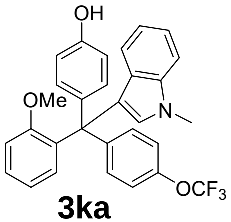

3ka

Current Data Parameters
NAME 211223sjw
EXPNO 5730
PROCNO 1

F2 - Acquisition Parameters
Date_ 20211223
Time 9.07
INSTRUM spect
PROBHD 5 mm PABBO BB-
PULPROG zg30
TD 65536
SOLVENT CDCl3
NS 16
DS 2
SWH 6009.615 Hz
FIDRES 0.091699 Hz
AQ 5.4525952 sec
RG 101
DW 83.200 usec
DE 6.50 usec
TE -59.1 K
D1 1.00000000 sec
TD0 1

======== CHANNEL f1 ========
SFO1 300.1318534 MHz
NUC1 1H
P1 10.00 usec
PLW1 14.00000000 W

F2 - Processing parameters
SI 65536
SF 300.1300169 MHz
WDW EM
SSB 0
LB 0.30 Hz
GB 0
PC 1.00

3ka

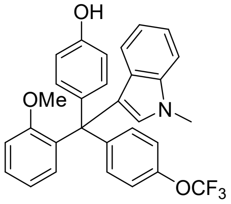

3ka

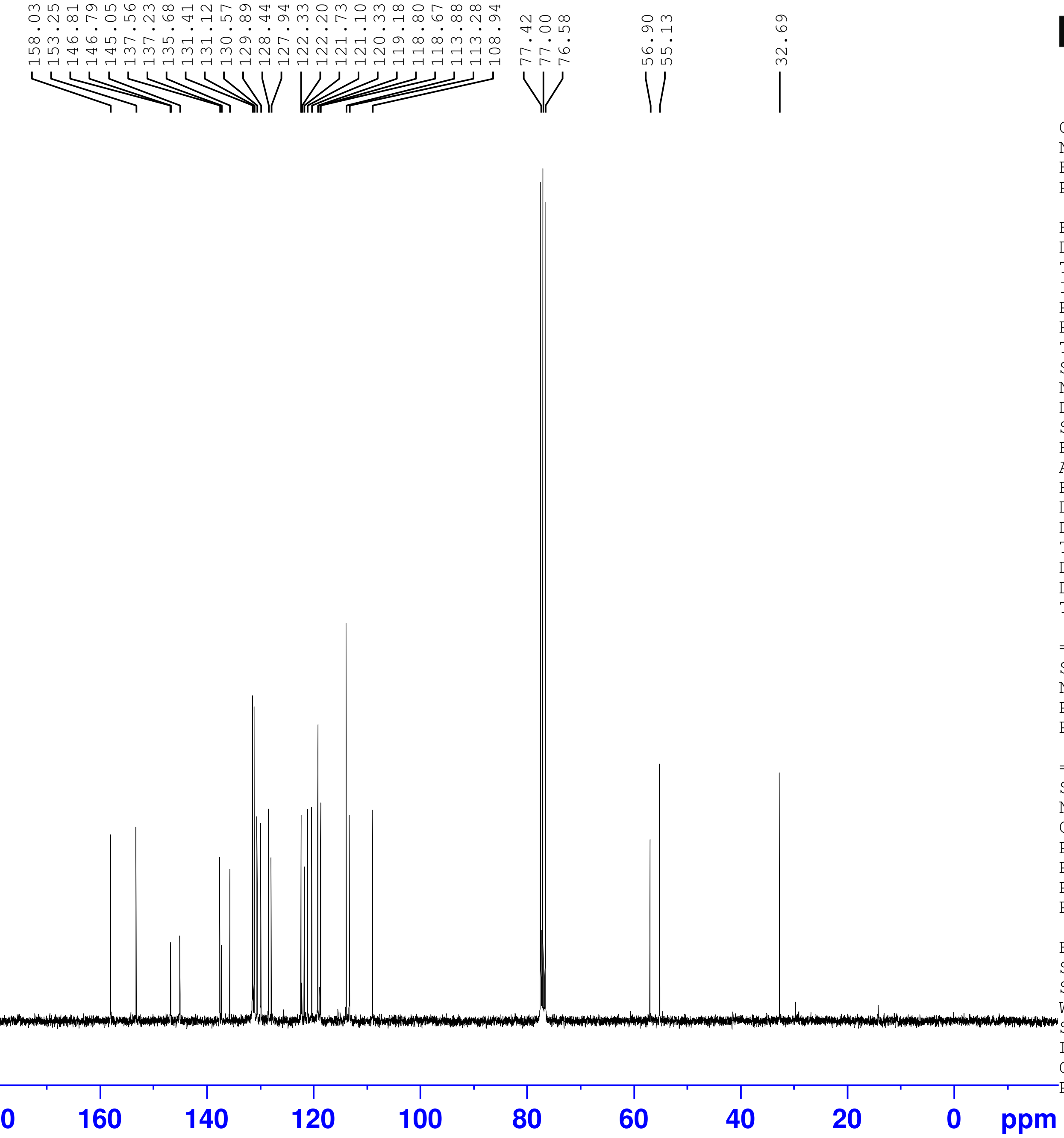


```
Current Data Parameters
NAME           3ka-ZY-4-75E
EXPNO                  5739
PROCNO                    1

F2 - Acquisition Parameters
Date_              20211224
Time                  10.31
INSTRUM               spect
PROBHD   5 mm PABBO BB-
PULPROG             zgpg30
TD                   65536
SOLVENT              CDCl3
NS                    1024
DS                       4
SWH              18028.846 Hz
FIDRES            0.275098 Hz
AQ               1.8175317 sec
RG                     203
DW                  27.733 usec
DE                    6.50 usec
TE                   -59.1 K
D1              2.00000000 sec
D11             0.03000000 sec
TD0                      1

======== CHANNEL f1 ========
SFO1            75.4752949 MHz
NUC1                   13C
P1                    9.50 usec
PLW1           34.20000076 W

======== CHANNEL f2 ========
SFO2           300.1312005 MHz
NUC2                    1H
CPDPRG[2           waltz16
PCPD2                90.00 usec
PLW2           14.00000000 W
PLW12           0.17284000 W
PLW13           0.14000000 W

F2 - Processing parameters
SI                   32768
SF              75.4677548 MHz
WDW                     EM
SSB                      0
LB                    1.00 Hz
GB                       0
PC                    1.40
```

3ka

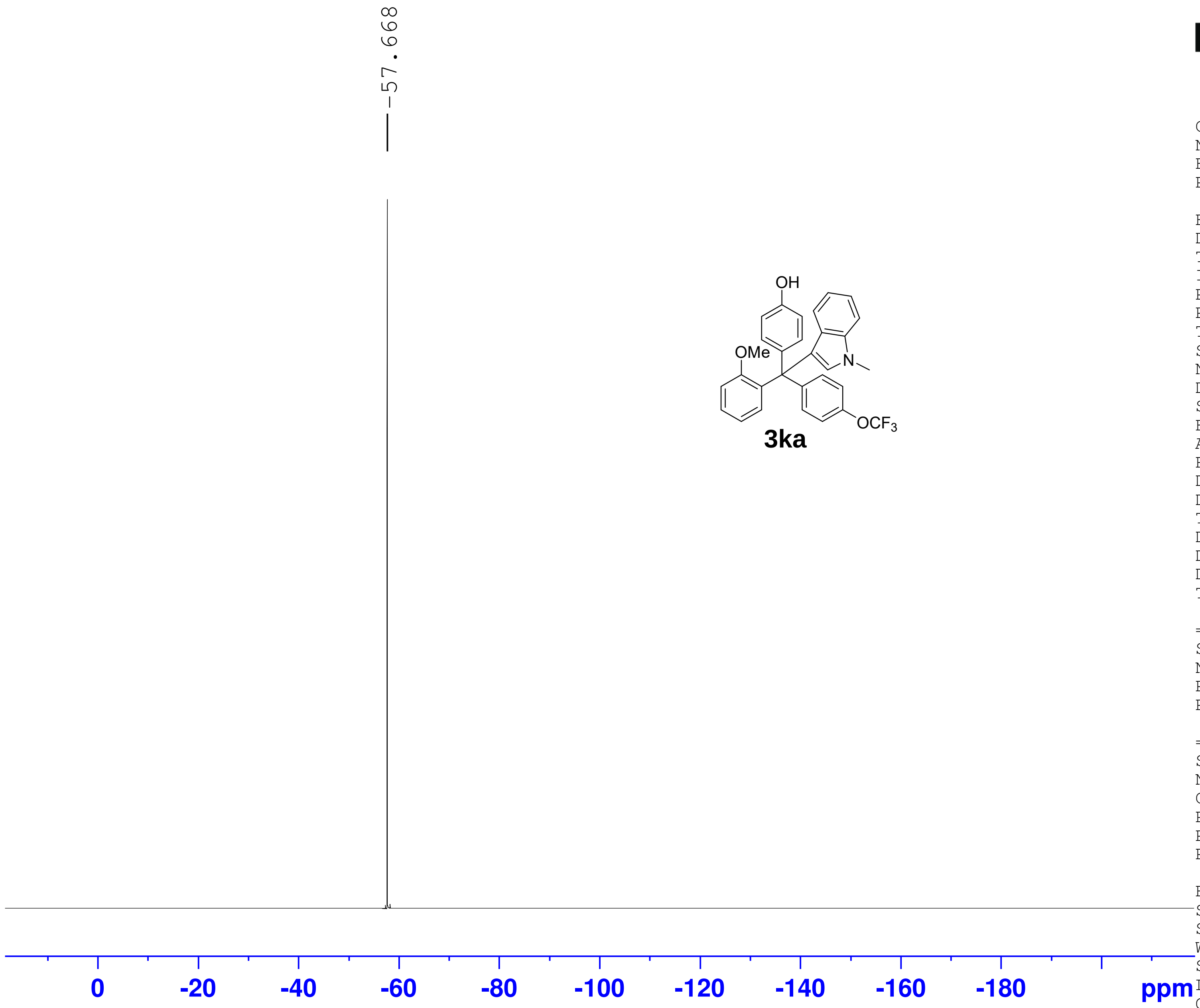

Current Data Parameters
NAME 211124sjw (2)
EXPNO 5628
PROCNO 1

F2 - Acquisition Parameters
Date_ 20211124
Time 9.09
INSTRUM spect
PROBHD 5 mm PABBO BB-
PULPROG zgfhigqn.2
TD 131072
SOLVENT CDCl3
NS 16
DS 4
SWH 66964.289 Hz
FIDRES 0.510897 Hz
AQ 0.9786710 sec
RG 203
DW 7.467 usec
DE 6.50 usec
TE -59.1 K
D1 1.00000000 sec
D11 0.03000000 sec
D12 0.00002000 sec
TD0 1

======== CHANNEL f1 ========
SFO1 282.3761148 MHz
NUC1 19F
P1 14.50 usec
PLW1 10.39999962 W

======== CHANNEL f2 ========
SFO2 300.1312005 MHz
NUC2 1H
CPDPRG[2 waltz16
PCPD2 90.00 usec
PLW2 14.00000000 W
PLW12 0.17284000 W

F2 - Processing parameters
SI 65536
SF 282.4043552 MHz
WDW EM
SSB 0
LB 0.30 Hz
GB 0
PC 1.00

3la

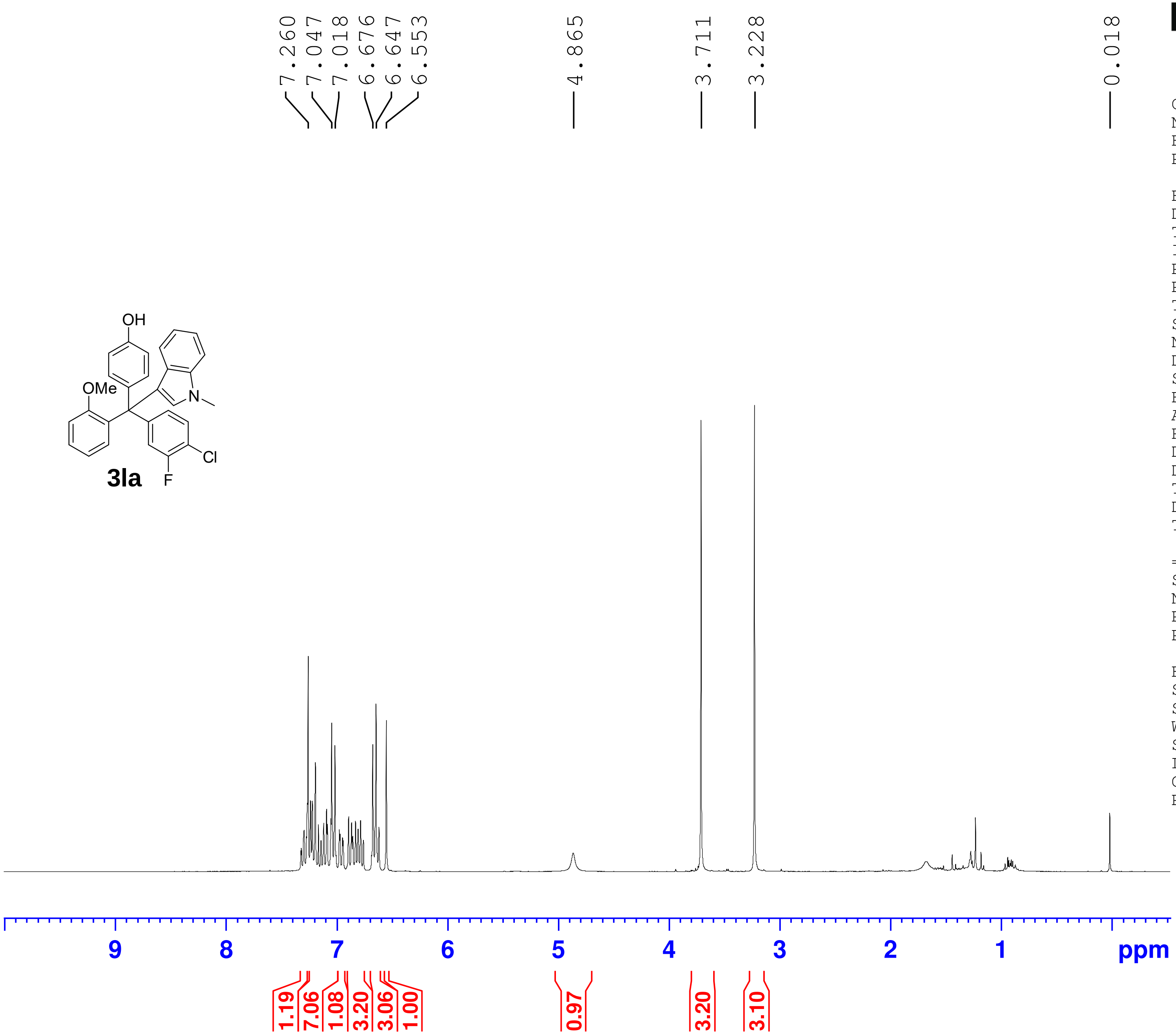

3la

```
Current Data Parameters
NAME             211015sjw
EXPNO                 5528
PROCNO                   1

F2 - Acquisition Parameters
Date_             20211015
Time                 10.02
INSTRUM              spect
PROBHD     5 mm PABBO BB-
PULPROG               zg30
TD                   65536
SOLVENT              CDCl3
NS                      16
DS                       2
SWH               6009.615 Hz
FIDRES            0.091699 Hz
AQ               5.4525952 sec
RG                     128
DW                  83.200 usec
DE                    6.50 usec
TE                   -59.1 K
D1              1.00000000 sec
TD0                      1

======== CHANNEL f1 ========
SFO1           300.1318534 MHz
NUC1                    1H
P1                   10.00 usec
PLW1           14.00000000 W

F2 - Processing parameters
SI                   65536
SF             300.1300073 MHz
WDW                     EM
SSB                      0
LB                    0.30 Hz
GB                       0
PC                    1.00
```

3la

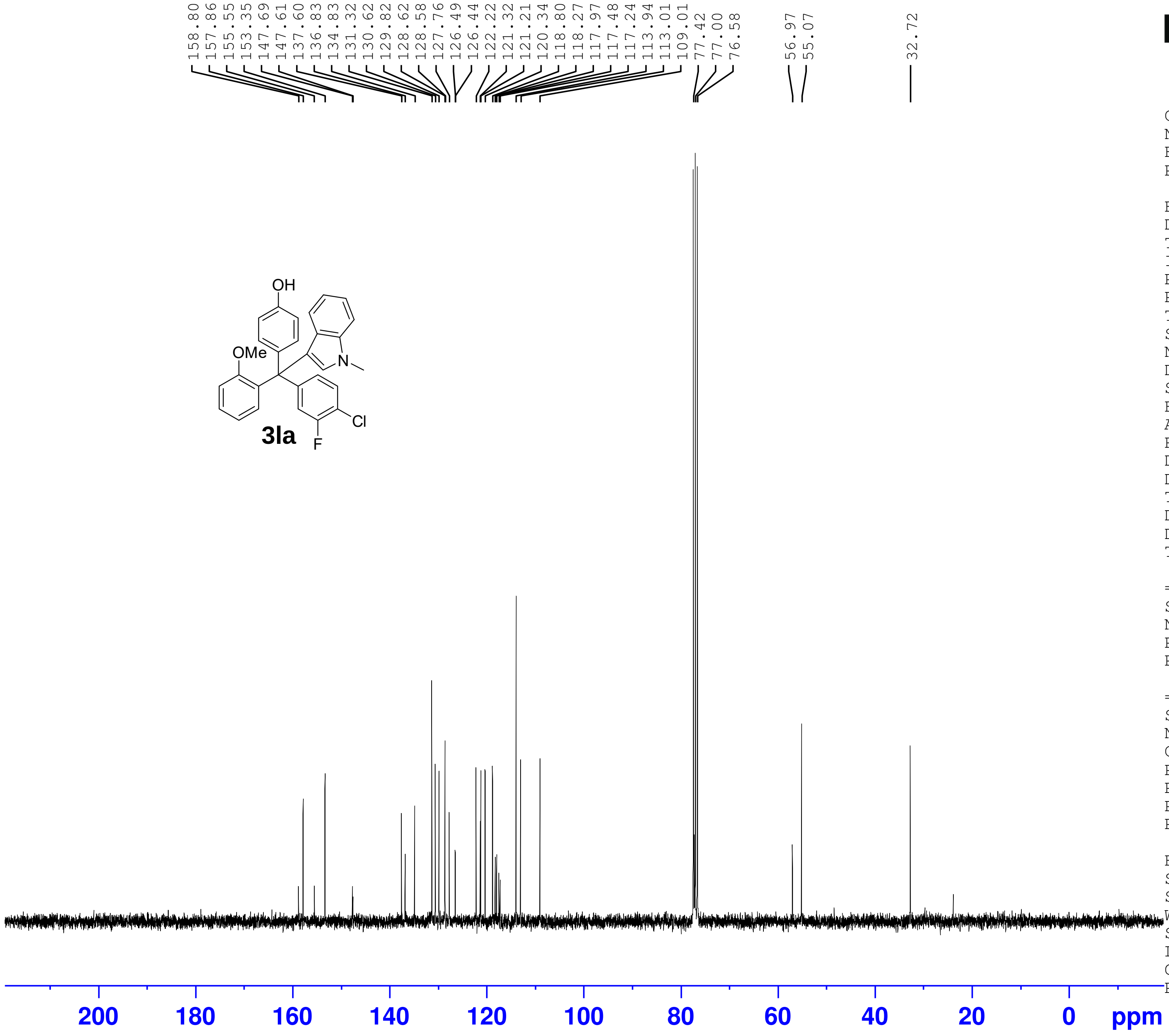

BRUKER

Current Data Parameters
NAME 3la-ZY-4-93C
EXPNO 5537
PROCNO 1

F2 - Acquisition Parameters
Date_ 20211016
Time 13.05
INSTRUM spect
PROBHD 5 mm PABBO BB-
PULPROG zgpg30
TD 65536
SOLVENT CDCl3
NS 500
DS 4
SWH 18028.846 Hz
FIDRES 0.275098 Hz
AQ 1.8175317 sec
RG 203
DW 27.733 usec
DE 6.50 usec
TE -59.1 K
D1 2.00000000 sec
D11 0.03000000 sec
TD0 1

======== CHANNEL f1 ========
SFO1 75.4752949 MHz
NUC1 13C
P1 9.50 usec
PLW1 34.20000076 W

======== CHANNEL f2 ========
SFO2 300.1312005 MHz
NUC2 1H
CPDPRG[2 waltz16
PCPD2 90.00 usec
PLW2 14.00000000 W
PLW12 0.17284000 W
PLW13 0.14000000 W

F2 - Processing parameters
SI 32768
SF 75.4677533 MHz
WDW EM
SSB 0
LB 1.00 Hz
GB 0
PC 1.40

3la

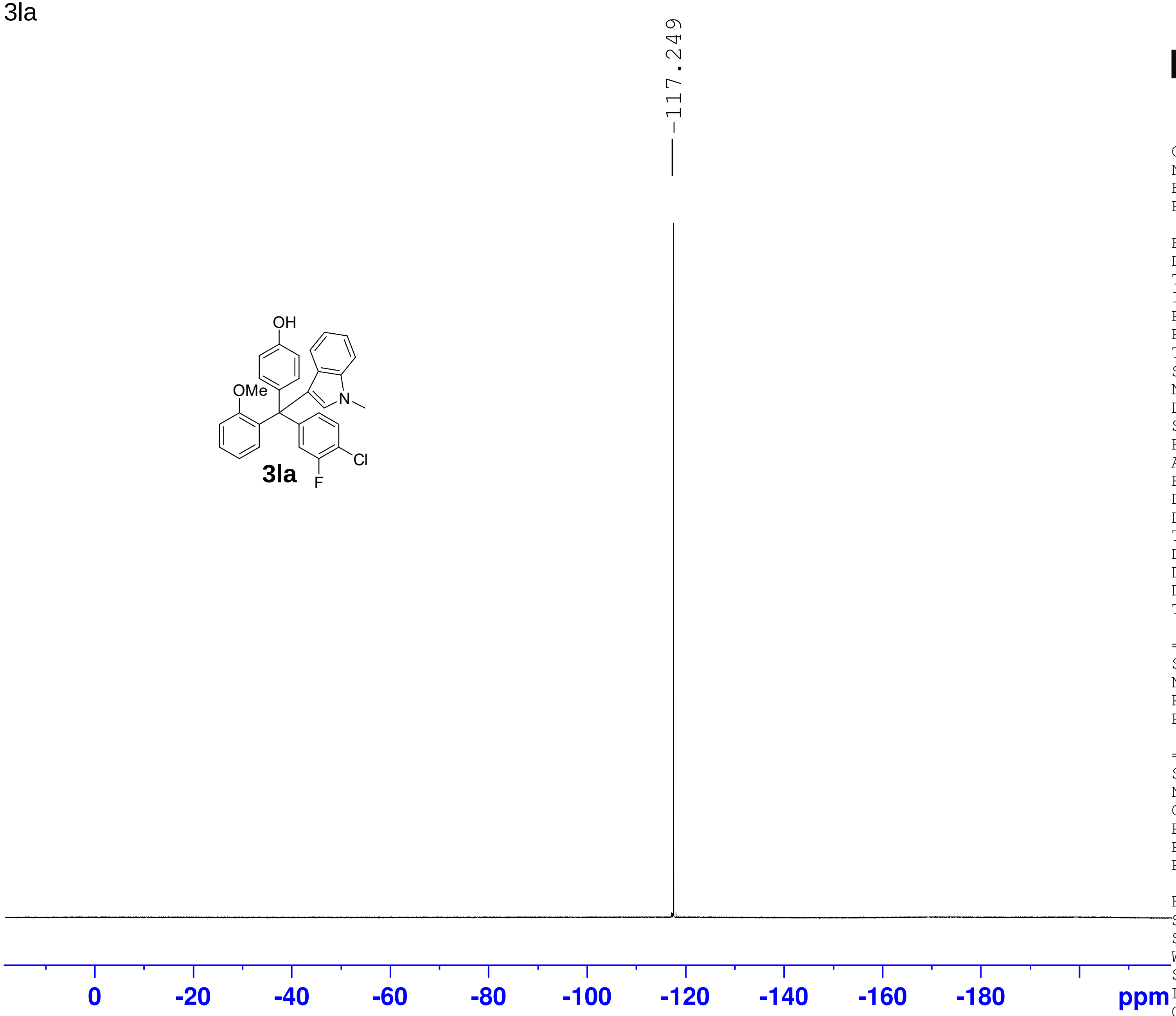

Current Data Parameters

NAME	211019sjw	
EXPNO	5541	
PROCNO	1	

F2 - Acquisition Parameters

Date_	20211019	
Time	9.40	
INSTRUM	spect	
PROBHD	5 mm PABBO BB-	
PULPROG	zgfhigqn.2	
TD	131072	
SOLVENT	CDCl3	
NS	16	
DS	4	
SWH	66964.289	Hz
FIDRES	0.510897	Hz
AQ	0.9786710	sec
RG	203	
DW	7.467	usec
DE	6.50	usec
TE	-59.1	K
D1	1.00000000	sec
D11	0.03000000	sec
D12	0.00002000	sec
TD0	1	

======== CHANNEL f1 ========

SFO1	282.3761148	MHz
NUC1	19F	
P1	14.50	usec
PLW1	10.39999962	W

======== CHANNEL f2 ========

SFO2	300.1312005	MHz
NUC2	1H	
CPDPRG[2	waltz16	
PCPD2	90.00	usec
PLW2	14.00000000	W
PLW12	0.17284000	W

F2 - Processing parameters

SI	65536	
SF	282.4043552	MHz
WDW	EM	
SSB	0	
LB	0.30	Hz
GB	0	
PC	1.00	

3ma

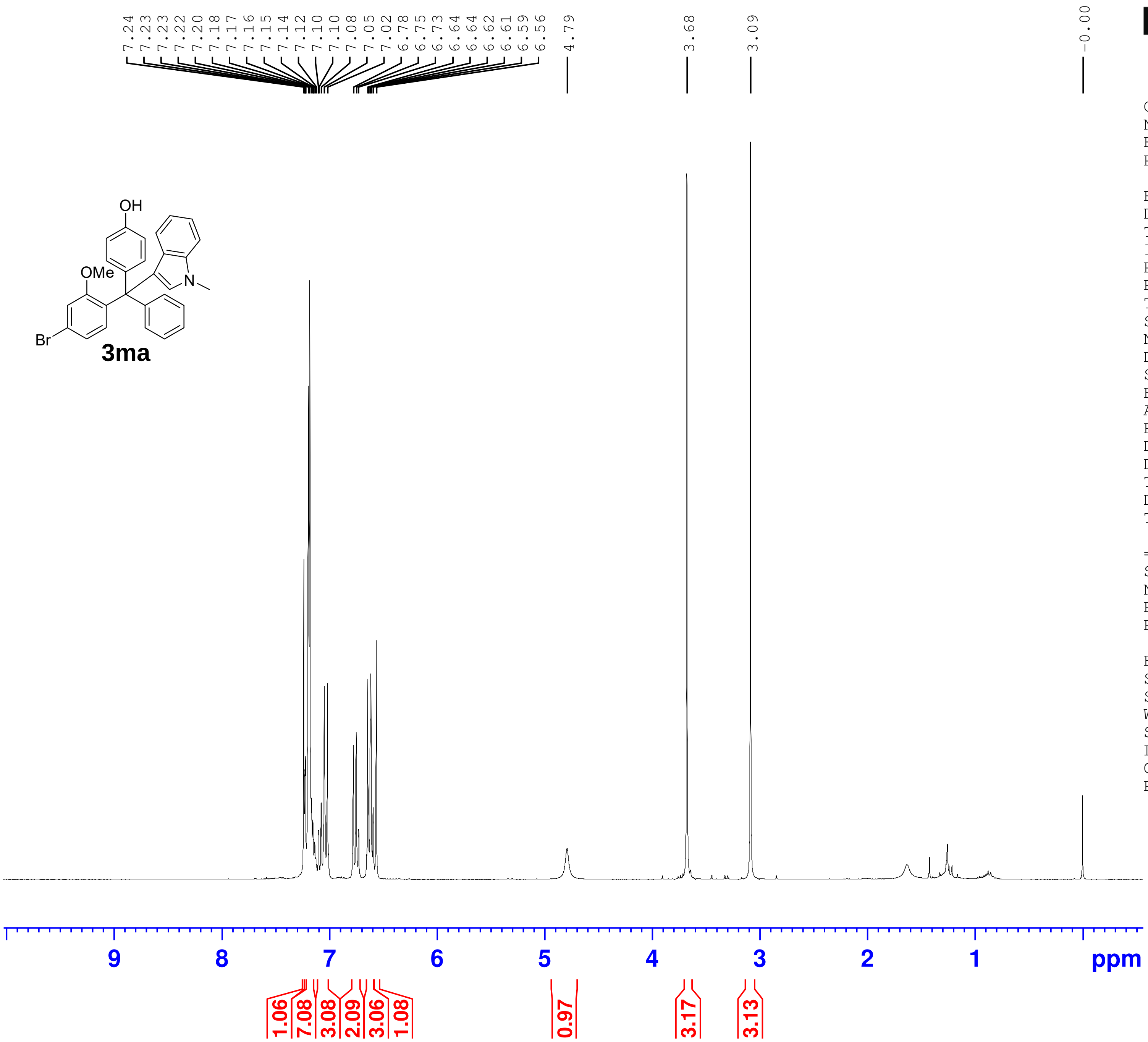

Current Data Parameters

NAME	ZY-4-93D-h-fr
EXPNO	5529
PROCNO	1

F2 - Acquisition Parameters

Date_	20211015
Time	10.06
INSTRUM	spect
PROBHD	5 mm PABBO BB-
PULPROG	zg30
TD	65536
SOLVENT	CDCl3
NS	16
DS	2
SWH	6009.615 Hz
FIDRES	0.091699 Hz
AQ	5.4525952 sec
RG	128
DW	83.200 usec
DE	6.50 usec
TE	-59.1 K
D1	1.00000000 sec
TD0	1

======== CHANNEL f1 ========

SFO1	300.1318534 MHz
NUC1	1H
P1	10.00 usec
PLW1	14.00000000 W

F2 - Processing parameters

SI	65536
SF	300.1300136 MHz
WDW	EM
SSB	0
LB	0.30 Hz
GB	0
PC	1.00

3ma

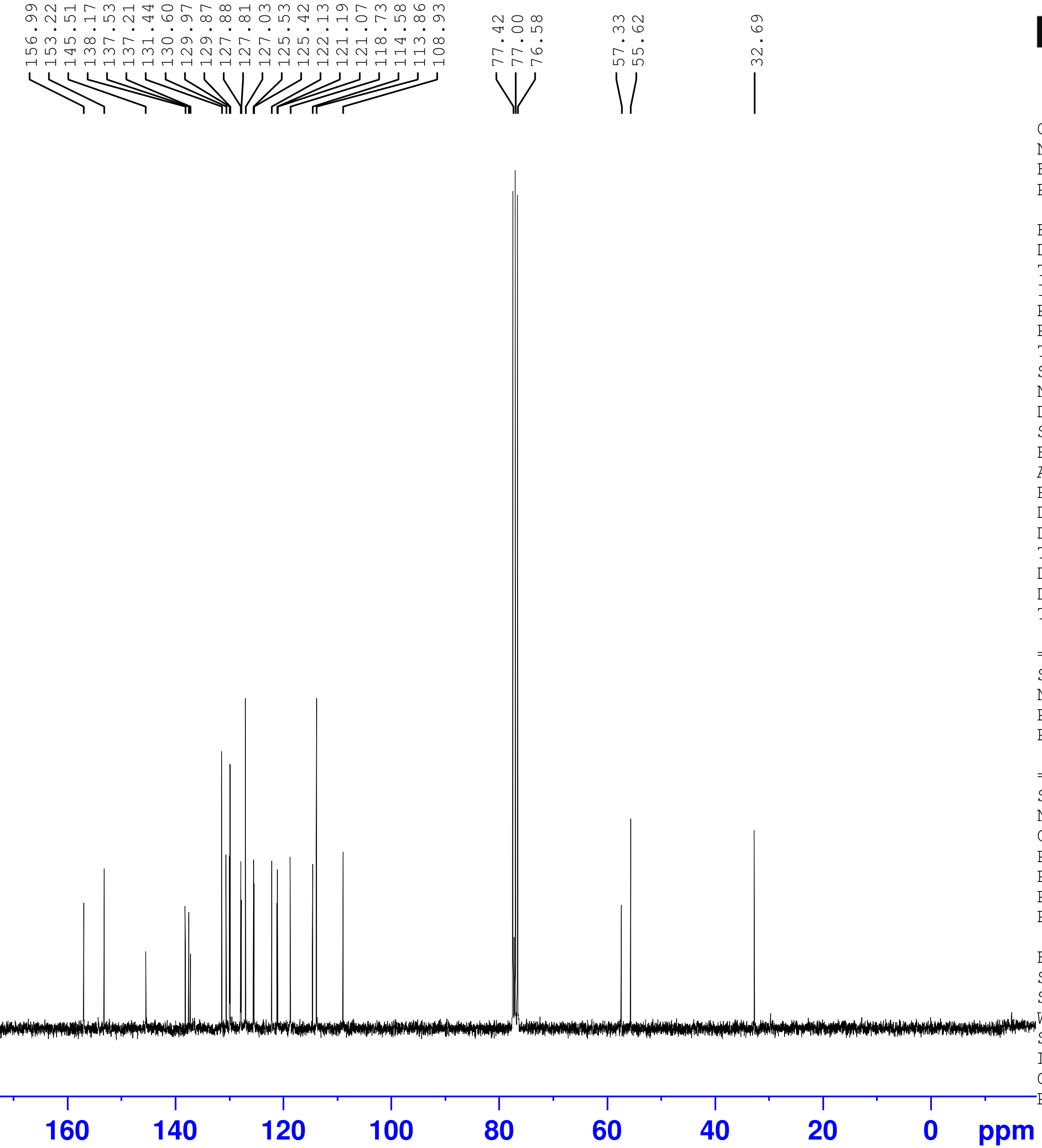

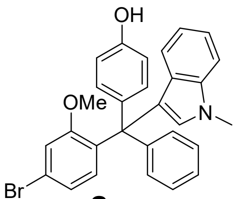

3ma

Current Data Parameters
NAME 3ma-ZY-4-93D
EXPNO 5538
PROCNO 1

F2 - Acquisition Parameters
Date_ 20211016
Time 13.41
INSTRUM spect
PROBHD 5 mm PABBO BB-
PULPROG zgpg30
TD 65536
SOLVENT CDCl3
NS 500
DS 4
SWH 18028.846 Hz
FIDRES 0.275098 Hz
AQ 1.8175317 sec
RG 203
DW 27.733 usec
DE 6.50 usec
TE -59.1 K
D1 2.00000000 sec
D11 0.03000000 sec
TD0 1

======== CHANNEL f1 ========
SFO1 75.4752949 MHz
NUC1 13C
P1 9.50 usec
PLW1 34.20000076 W

======== CHANNEL f2 ========
SFO2 300.1312005 MHz
NUC2 1H
CPDPRG[2 waltz16
PCPD2 90.00 usec
PLW2 14.00000000 W
PLW12 0.17284000 W
PLW13 0.14000000 W

F2 - Processing parameters
SI 32768
SF 75.4677539 MHz
WDW EM
SSB 0
LB 1.00 Hz
GB 0
PC 1.40

3na

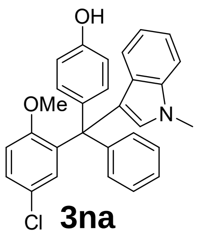

3na

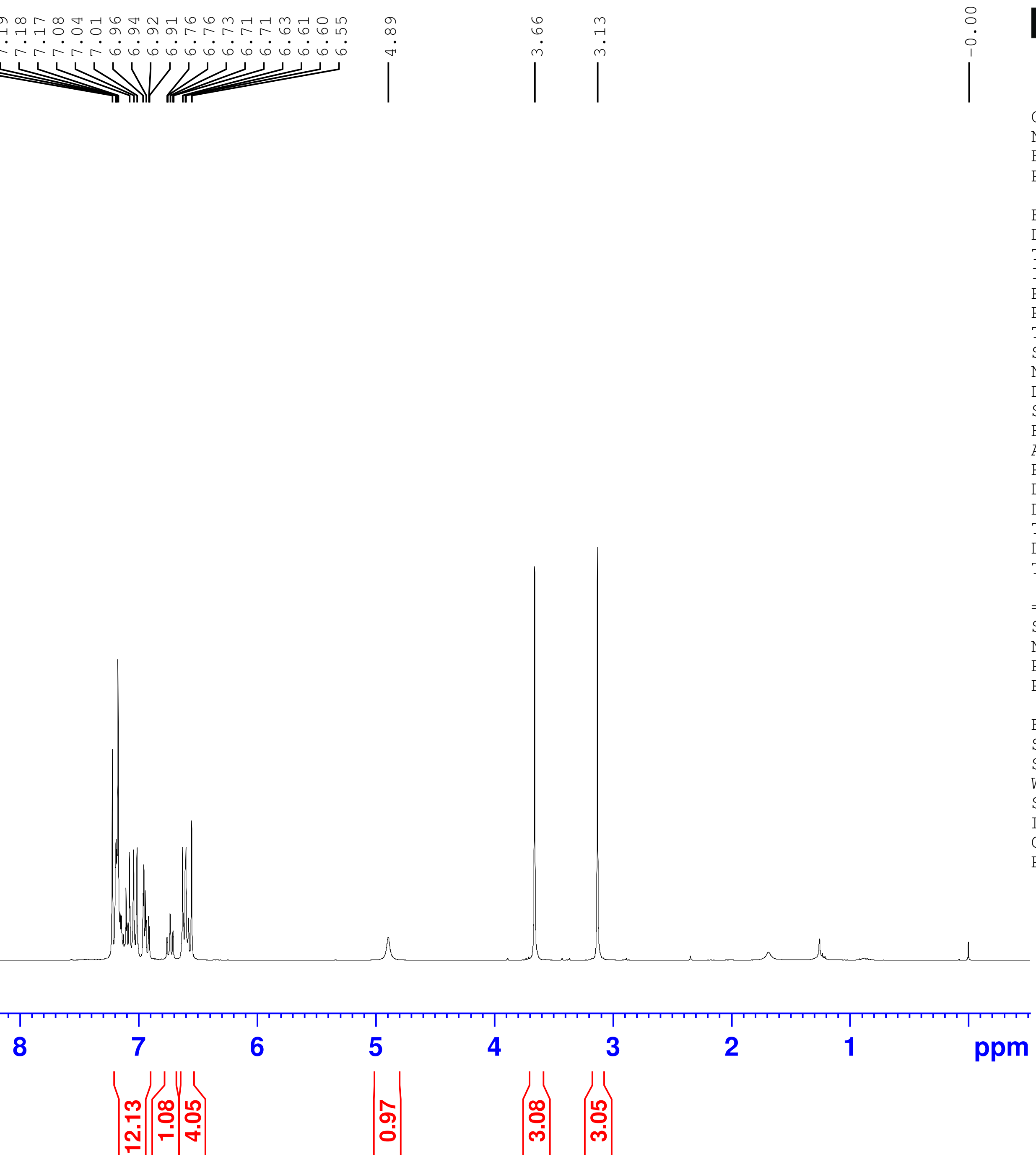


```
Current Data Parameters
NAME          ZY-4-93E-h-fr
EXPNO                  5618
PROCNO                    1

F2 - Acquisition Parameters
Date_              20211118
Time                  10.06
INSTRUM               spect
PROBHD     5 mm PABBO BB-
PULPROG                zg30
TD                    65536
SOLVENT               CDCl3
NS                       16
DS                        2
SWH                6009.615 Hz
FIDRES             0.091699 Hz
AQ                5.4525952 sec
RG                     90.5
DW                   83.200 usec
DE                     6.50 usec
TE                    -59.1 K
D1               1.00000000 sec
TD0                       1

======== CHANNEL f1 ========
SFO1            300.1318534 MHz
NUC1                     1H
P1                    10.00 usec
PLW1            14.00000000 W

F2 - Processing parameters
SI                    65536
SF              300.1300191 MHz
WDW                      EM
SSB                       0
LB                     0.30 Hz
GB                        0
PC                     1.00
```

3na

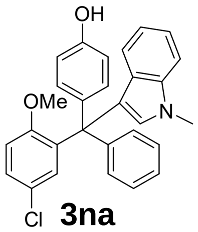

3na

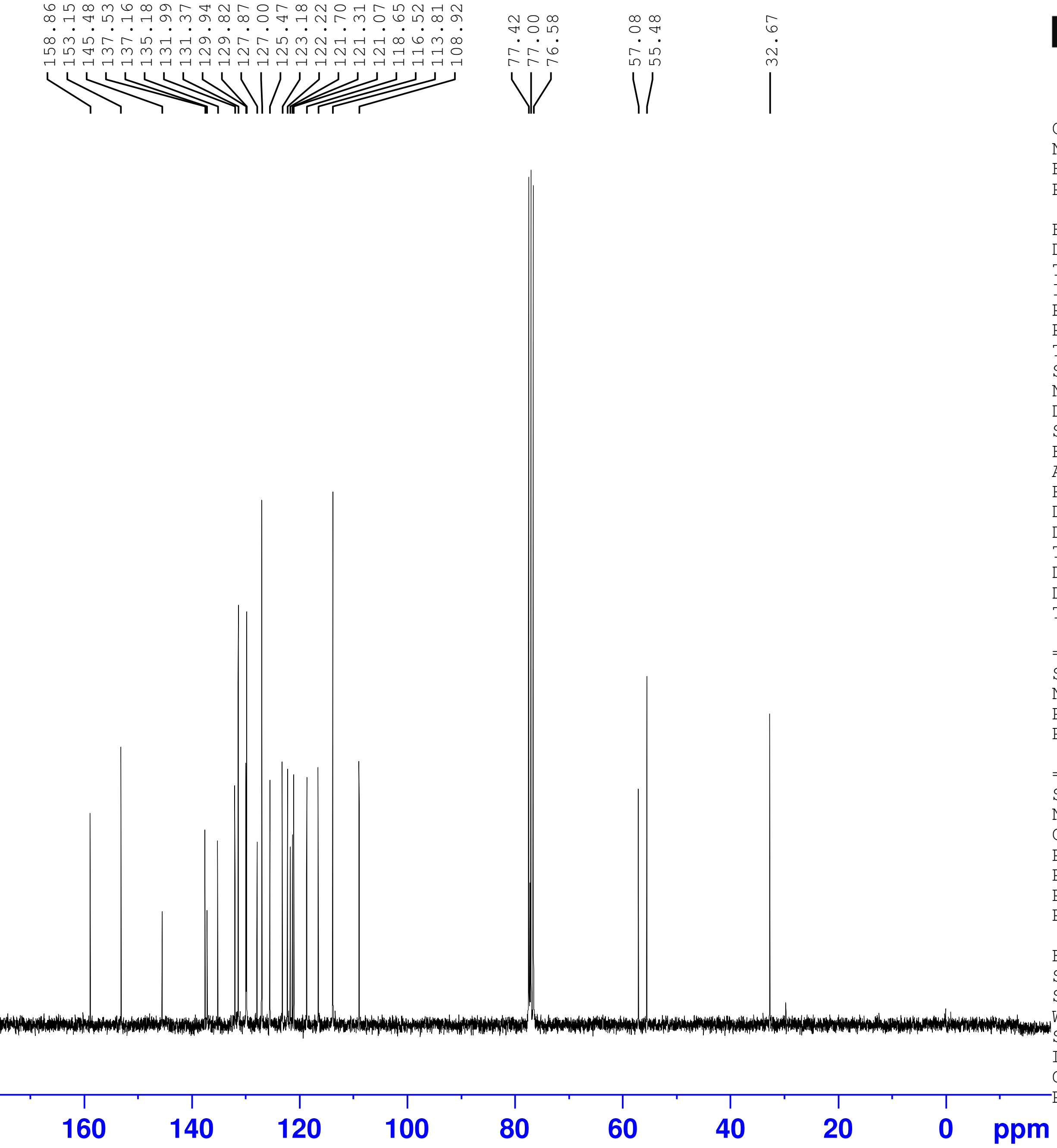

BRUKER

Current Data Parameters
NAME 3na-ZY-4-93E
EXPNO 5619
PROCNO 1

F2 - Acquisition Parameters
Date_ 20211118
Time 10.39
INSTRUM spect
PROBHD 5 mm PABBO BB-
PULPROG zgpg30
TD 65536
SOLVENT CDCl3
NS 500
DS 4
SWH 18028.846 Hz
FIDRES 0.275098 Hz
AQ 1.8175317 sec
RG 203
DW 27.733 usec
DE 6.50 usec
TE -59.1 K
D1 2.00000000 sec
D11 0.03000000 sec
TD0 1

======== CHANNEL f1 ========
SFO1 75.4752949 MHz
NUC1 13C
P1 9.50 usec
PLW1 34.20000076 W

======== CHANNEL f2 ========
SFO2 300.1312005 MHz
NUC2 1H
CPDPRG[2 waltz16
PCPD2 90.00 usec
PLW2 14.00000000 W
PLW12 0.17284000 W
PLW13 0.14000000 W

F2 - Processing parameters
SI 32768
SF 75.4677563 MHz
WDW EM
SSB 0
LB 1.00 Hz
GB 0
PC 1.40

3oa

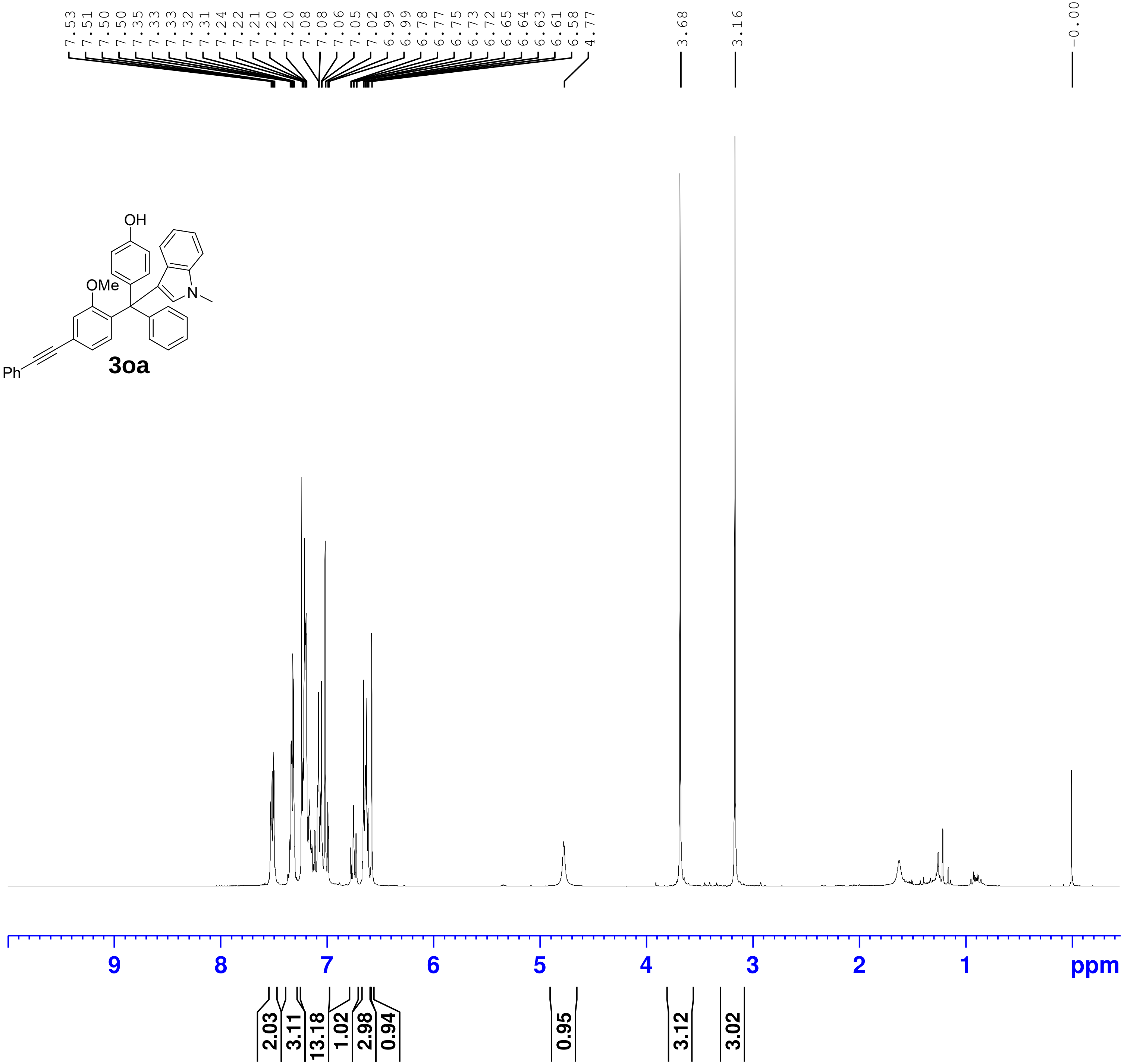

3oa

BRUKER

Current Data Parameters
NAME ZY-4-93F-h-fr
EXPNO 5531
PROCNO 1

F2 - Acquisition Parameters
Date_ 20211015
Time 10.16
INSTRUM spect
PROBHD 5 mm PABBO BB-
PULPROG zg30
TD 65536
SOLVENT CDCl3
NS 16
DS 2
SWH 6009.615 Hz
FIDRES 0.091699 Hz
AQ 5.4525952 sec
RG 128
DW 83.200 usec
DE 6.50 usec
TE -59.1 K
D1 1.00000000 sec
TD0 1

======== CHANNEL f1 ========
SFO1 300.1318534 MHz
NUC1 1H
P1 10.00 usec
PLW1 14.00000000 W

F2 - Processing parameters
SI 65536
SF 300.1300146 MHz
WDW EM
SSB 0
LB 0.30 Hz
GB 0
PC 1.00

3oa

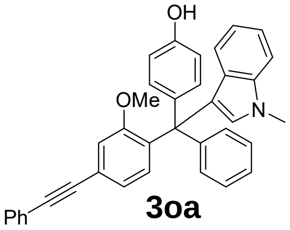

3oa

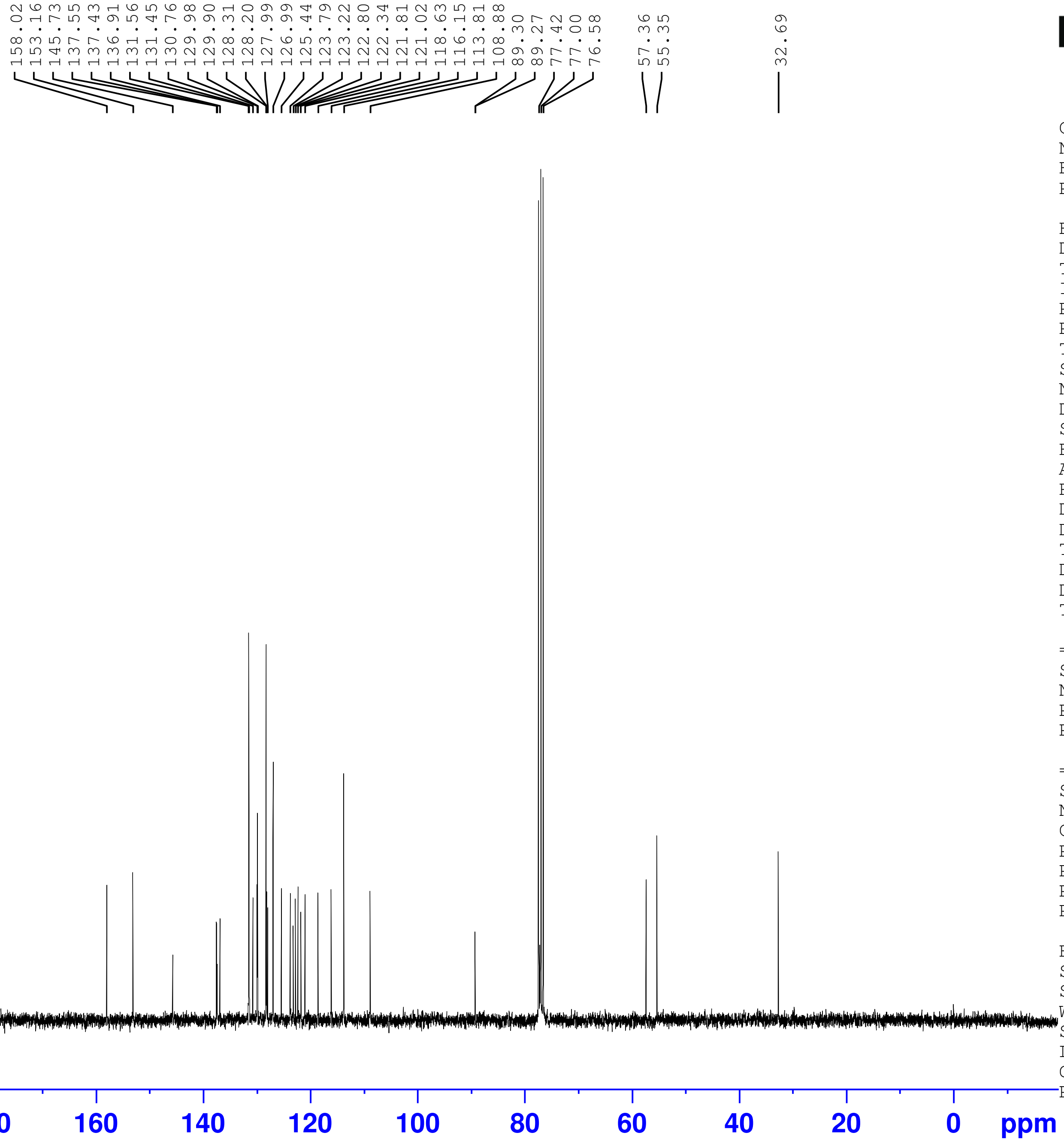

BRUKER

Current Data Parameters
NAME 3oa-ZY-4-93F
EXPNO 5539
PROCNO 1

F2 - Acquisition Parameters
Date_ 20211016
Time 14.16
INSTRUM spect
PROBHD 5 mm PABBO BB-
PULPROG zgpg30
TD 65536
SOLVENT CDCl3
NS 500
DS 4
SWH 18028.846 Hz
FIDRES 0.275098 Hz
AQ 1.8175317 sec
RG 203
DW 27.733 usec
DE 6.50 usec
TE -59.1 K
D1 2.00000000 sec
D11 0.03000000 sec
TD0 1

======== CHANNEL f1 ========
SFO1 75.4752949 MHz
NUC1 13C
P1 9.50 usec
PLW1 34.20000076 W

======== CHANNEL f2 ========
SFO2 300.1312005 MHz
NUC2 1H
CPDPRG[2 waltz16
PCPD2 90.00 usec
PLW2 14.00000000 W
PLW12 0.17284000 W
PLW13 0.14000000 W

F2 - Processing parameters
SI 32768
SF 75.4677540 MHz
WDW EM
SSB 0
LB 1.00 Hz
GB 0
PC 1.40

3dg

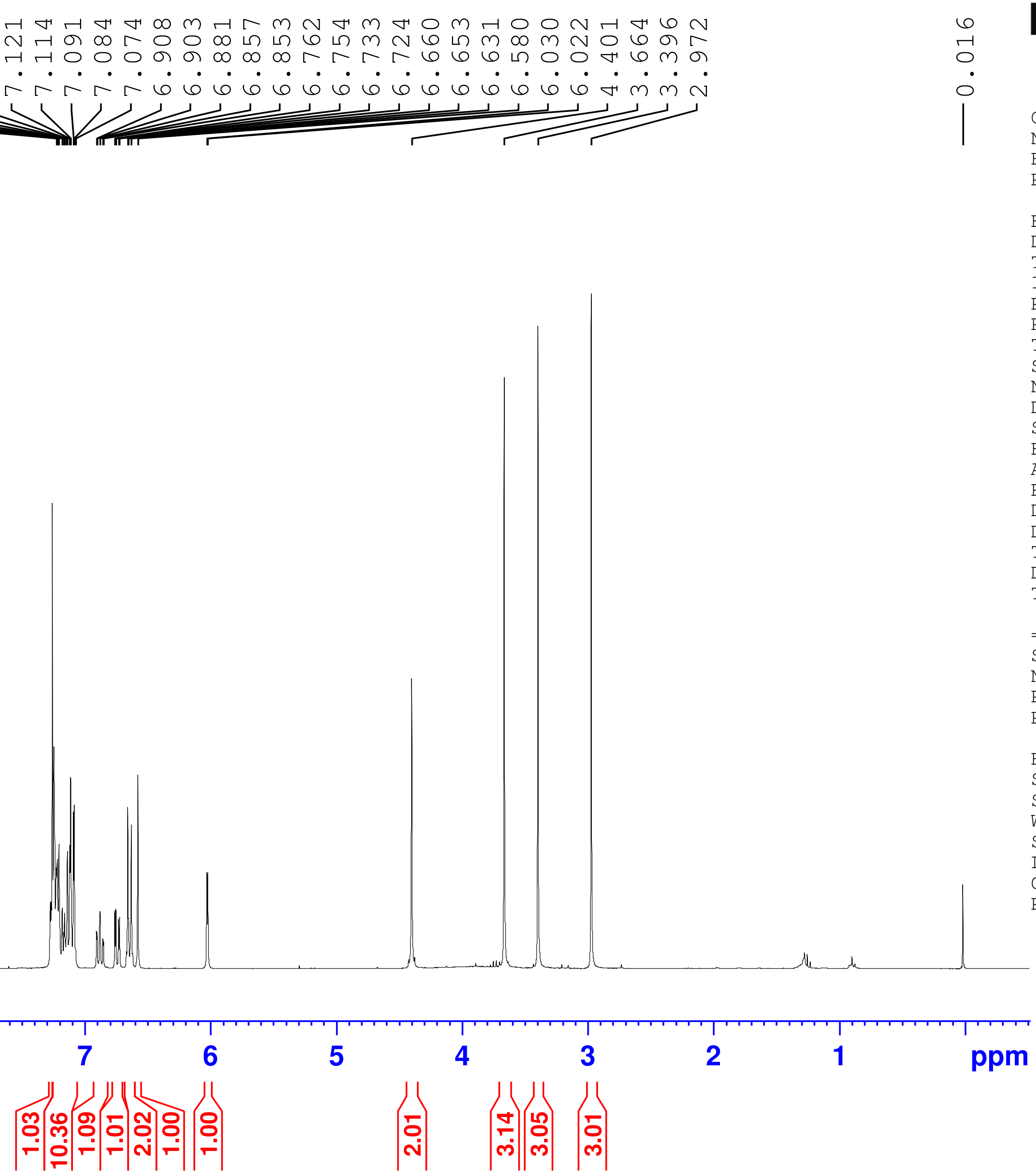

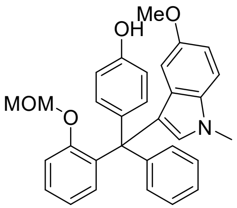

3dg

```
Current Data Parameters
NAME              211020sjw
EXPNO                  5541
PROCNO                    1

F2 - Acquisition Parameters
Date_              20211020
Time                   9.28
INSTRUM               spect
PROBHD    5 mm PABBO BB-
PULPROG                zg30
TD                    65536
SOLVENT               CDCl3
NS                       16
DS                        2
SWH                6009.615 Hz
FIDRES             0.091699 Hz
AQ                5.4525952 sec
RG                      114
DW                   83.200 usec
DE                     6.50 usec
TE                    -59.1 K
D1               1.00000000 sec
TD0                       1

======== CHANNEL f1 ========
SFO1            300.1318534 MHz
NUC1                     1H
P1                    10.00 usec
PLW1            14.00000000 W

F2 - Processing parameters
SI                    65536
SF              300.1300075 MHz
WDW                      EM
SSB                       0
LB                     0.30 Hz
GB                        0
PC                     1.00
```

3dg

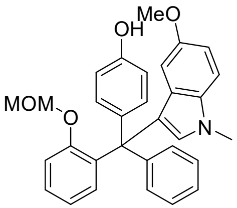

3dg

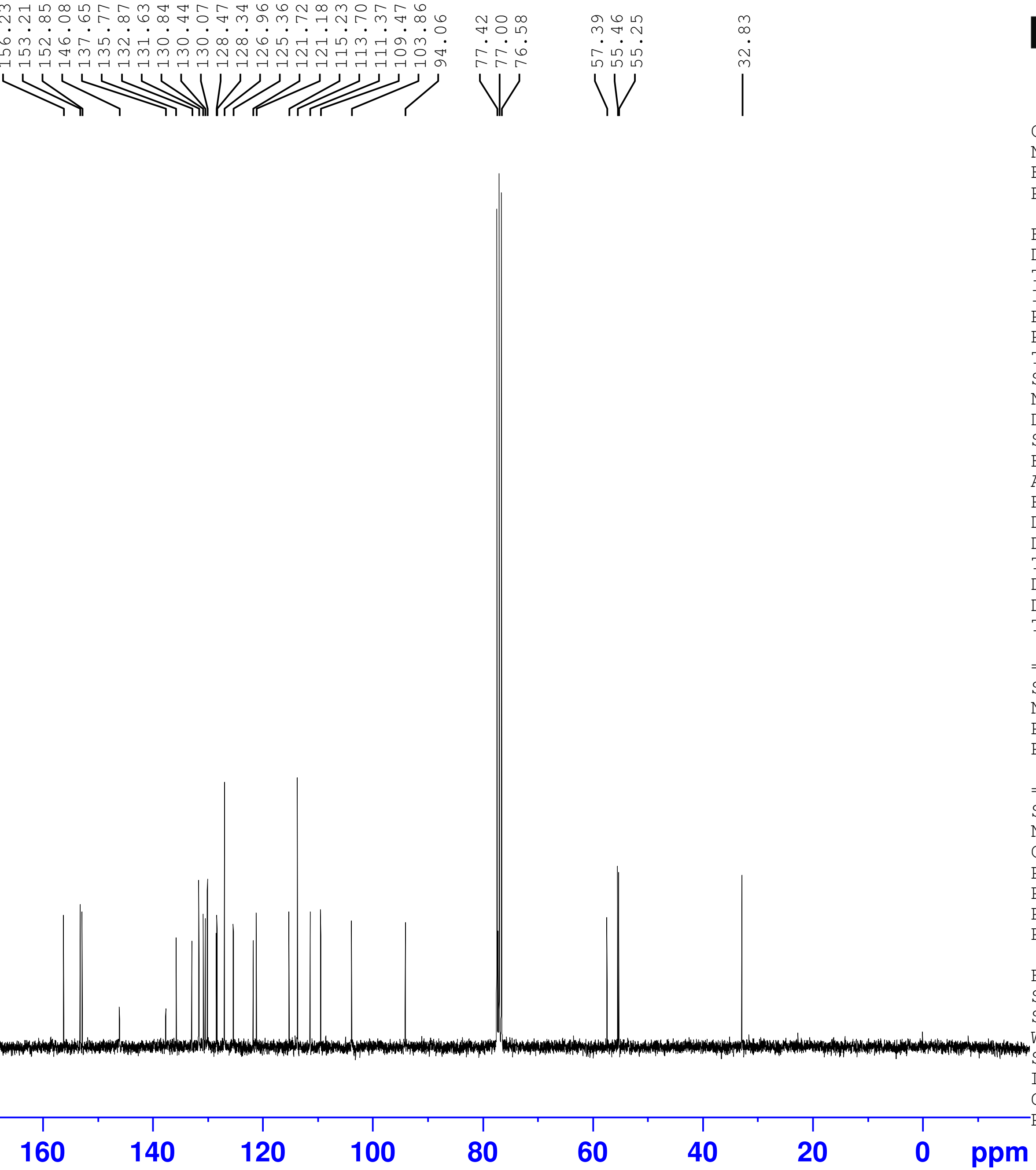

Current Data Parameters
NAME 3dg-ZY-4-86C
EXPNO 5562
PROCNO 1

F2 - Acquisition Parameters
Date_ 20211022
Time 12.10
INSTRUM spect
PROBHD 5 mm PABBO BB-
PULPROG zgpg30
TD 65536
SOLVENT CDCl3
NS 500
DS 4
SWH 18028.846 Hz
FIDRES 0.275098 Hz
AQ 1.8175317 sec
RG 203
DW 27.733 usec
DE 6.50 usec
TE -59.1 K
D1 2.00000000 sec
D11 0.03000000 sec
TD0 1

======== CHANNEL f1 ========
SFO1 75.4752949 MHz
NUC1 13C
P1 9.50 usec
PLW1 34.20000076 W

======== CHANNEL f2 ========
SFO2 300.1312005 MHz
NUC2 1H
CPDPRG[2 waltz16
PCPD2 90.00 usec
PLW2 14.00000000 W
PLW12 0.17284000 W
PLW13 0.14000000 W

F2 - Processing parameters
SI 32768
SF 75.4677535 MHz
WDW EM
SSB 0
LB 1.00 Hz
GB 0
PC 1.40

3gg

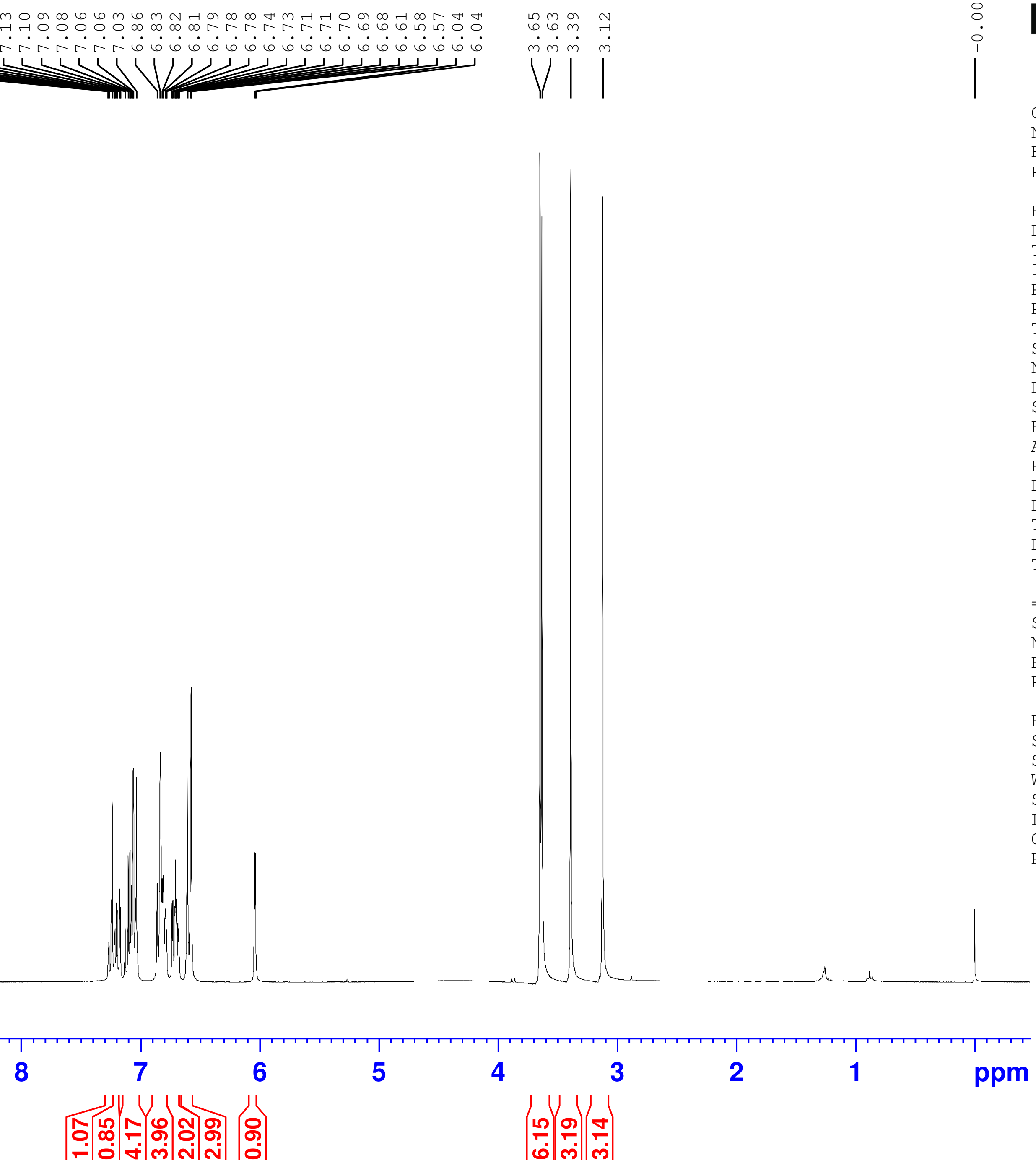

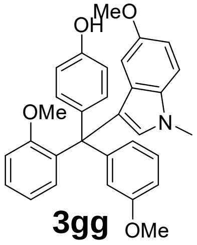

3gg

BRUKER

Current Data Parameters
NAME ZY-4-86E-h-fr
EXPNO 5543
PROCNO 1

F2 - Acquisition Parameters
Date_ 20211020
Time 9.37
INSTRUM spect
PROBHD 5 mm PABBO BB-
PULPROG zg30
TD 65536
SOLVENT CDCl3
NS 16
DS 2
SWH 6009.615 Hz
FIDRES 0.091699 Hz
AQ 5.4525952 sec
RG 101
DW 83.200 usec
DE 6.50 usec
TE -59.1 K
D1 1.00000000 sec
TD0 1

======== CHANNEL f1 ========
SFO1 300.1318534 MHz
NUC1 1H
P1 10.00 usec
PLW1 14.00000000 W

F2 - Processing parameters
SI 65536
SF 300.1300142 MHz
WDW EM
SSB 0
LB 0.30 Hz
GB 0
PC 1.00

3gg

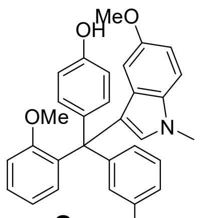

3gg

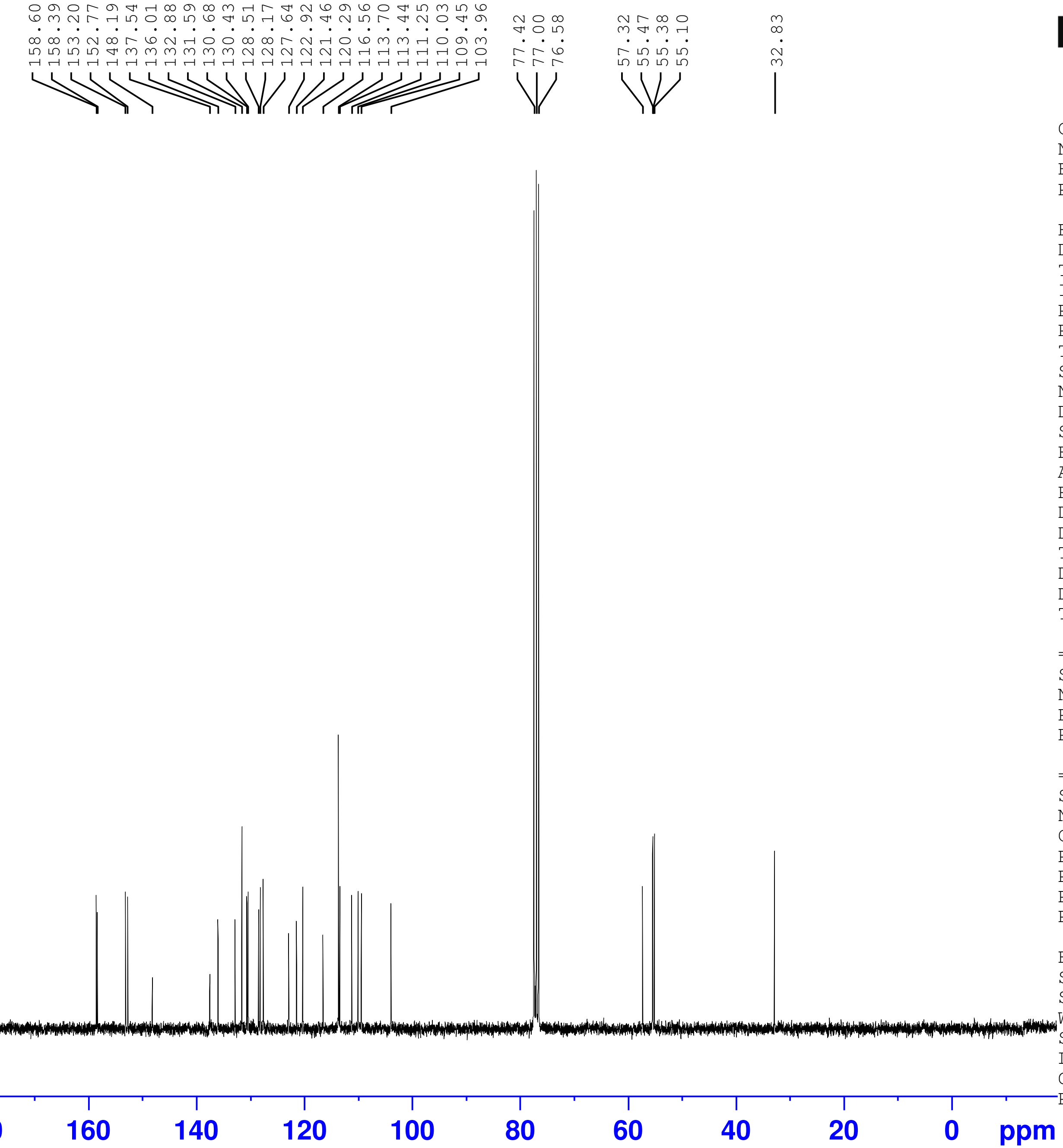

BRUKER

```
Current Data Parameters
NAME         3gg-ZY-4-86E
EXPNO                5564
PROCNO                  1

F2 - Acquisition Parameters
Date_            20211022
Time                13.22
INSTRUM             spect
PROBHD   5 mm PABBO BB-
PULPROG           zgpg30
TD                  65536
SOLVENT             CDCl3
NS                    500
DS                      4
SWH            18028.846 Hz
FIDRES          0.275098 Hz
AQ             1.8175317 sec
RG                    203
DW                 27.733 usec
DE                   6.50 usec
TE                  -59.1 K
D1             2.00000000 sec
D11            0.03000000 sec
TD0                     1

======== CHANNEL f1 ========
SFO1           75.4752949 MHz
NUC1                  13C
P1                   9.50 usec
PLW1          34.20000076 W

======== CHANNEL f2 ========
SFO2          300.1312005 MHz
NUC2                   1H
CPDPRG[2          waltz16
PCPD2               90.00 usec
PLW2          14.00000000 W
PLW12          0.17284000 W
PLW13          0.14000000 W

F2 - Processing parameters
SI                  32768
SF             75.4677539 MHz
WDW                    EM
SSB                     0
LB                   1.00 Hz
GB                      0
PC                   1.40
```

3hg

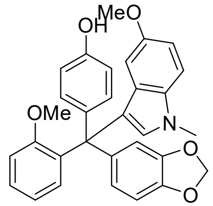

3hg

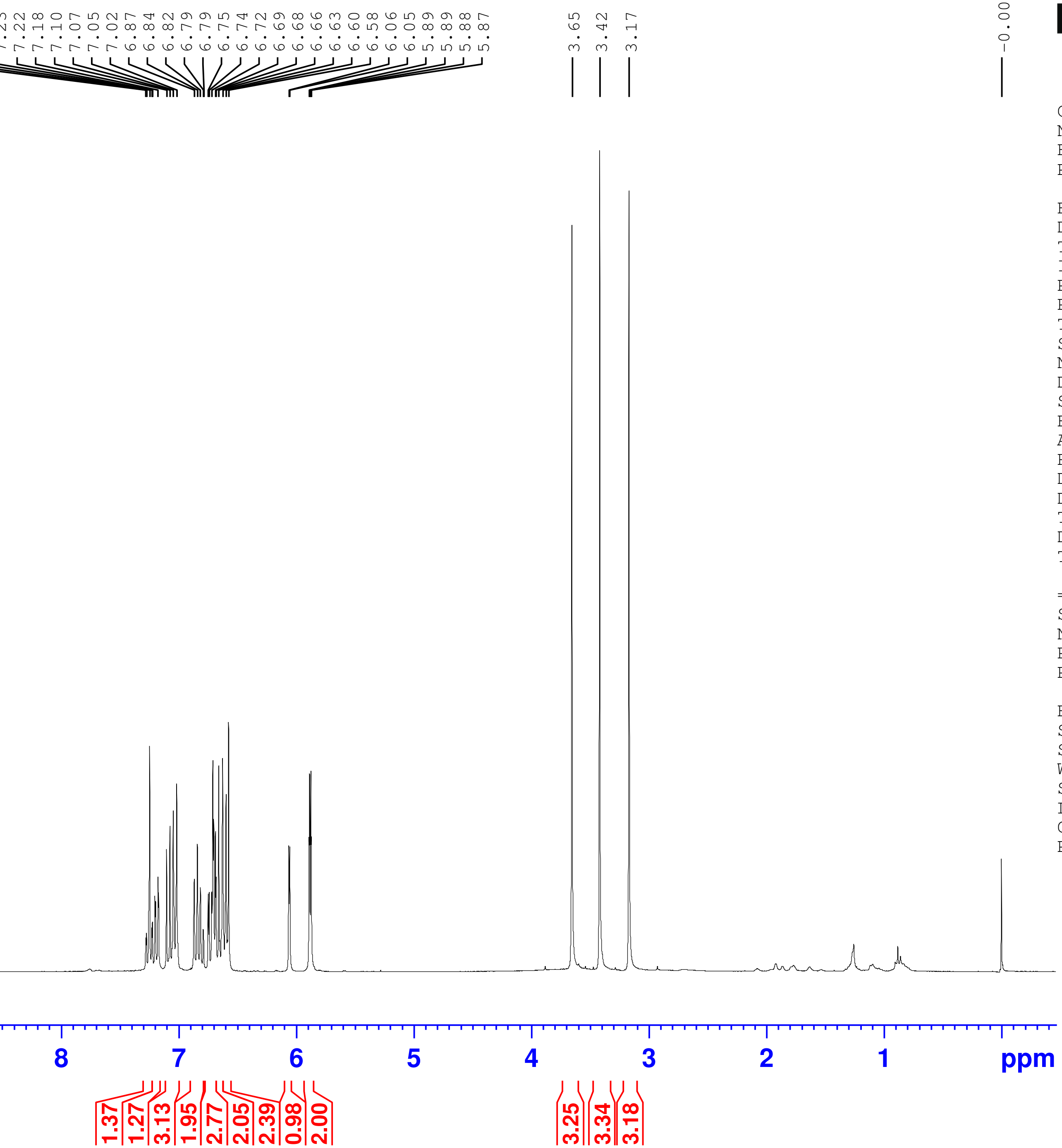


```
Current Data Parameters
NAME        ZY-4-86F-h-fr
EXPNO                5544
PROCNO                  1

F2 - Acquisition Parameters
Date_            20211020
Time                 9.42
INSTRUM             spect
PROBHD    5 mm PABBO BB-
PULPROG              zg30
TD                  65536
SOLVENT             CDCl3
NS                     16
DS                      2
SWH              6009.615 Hz
FIDRES           0.091699 Hz
AQ              5.4525952 sec
RG                    144
DW                 83.200 usec
DE                   6.50 usec
TE                  -59.1 K
D1             1.00000000 sec
TD0                     1

======== CHANNEL f1 ========
SFO1          300.1318534 MHz
NUC1                   1H
P1                  10.00 usec
PLW1          14.00000000 W

F2 - Processing parameters
SI                  65536
SF            300.1300113 MHz
WDW                    EM
SSB                     0
LB                   0.30 Hz
GB                      0
PC                   1.00
```

3hg

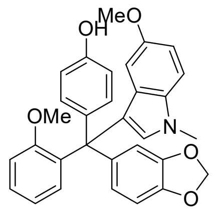

3hg

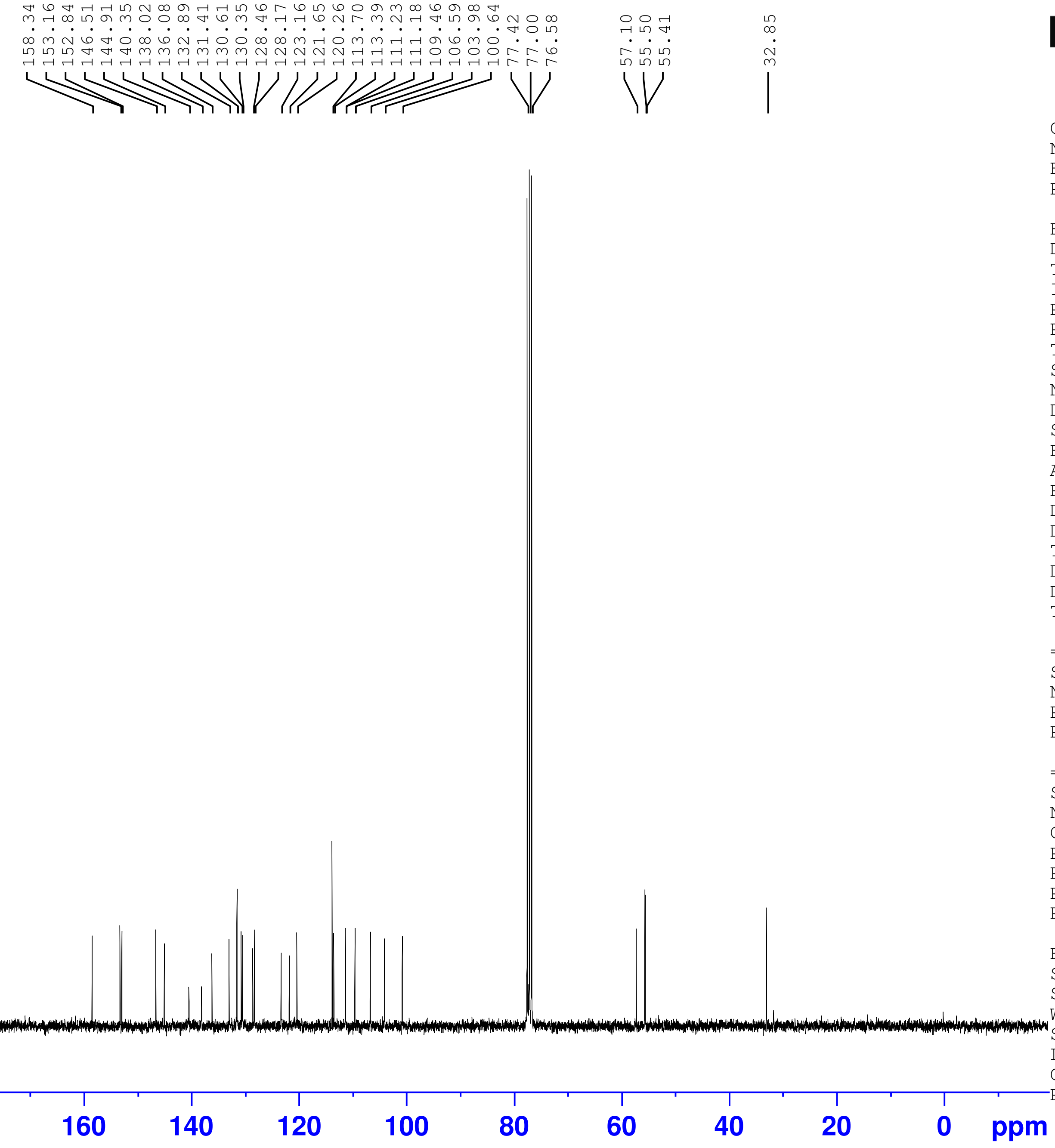

BRUKER

Current Data Parameters
NAME 3hg-ZY-4-86F
EXPNO 5555
PROCNO 1

F2 - Acquisition Parameters
Date_ 20211023
Time 10.01
INSTRUM spect
PROBHD 5 mm PABBO BB-
PULPROG zgpg30
TD 65536
SOLVENT CDCl3
NS 700
DS 4
SWH 18028.846 Hz
FIDRES 0.275098 Hz
AQ 1.8175317 sec
RG 203
DW 27.733 usec
DE 6.50 usec
TE -59.1 K
D1 2.00000000 sec
D11 0.03000000 sec
TD0 1

======== CHANNEL f1 ========
SFO1 75.4752949 MHz
NUC1 13C
P1 9.50 usec
PLW1 34.20000076 W

======== CHANNEL f2 ========
SFO2 300.1312005 MHz
NUC2 1H
CPDPRG[2 waltz16
PCPD2 90.00 usec
PLW2 14.00000000 W
PLW12 0.17284000 W
PLW13 0.14000000 W

F2 - Processing parameters
SI 32768
SF 75.4677529 MHz
WDW EM
SSB 0
LB 1.00 Hz
GB 0
PC 1.40

3pg

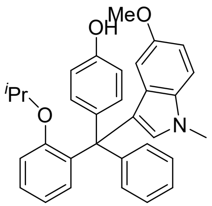

3pg

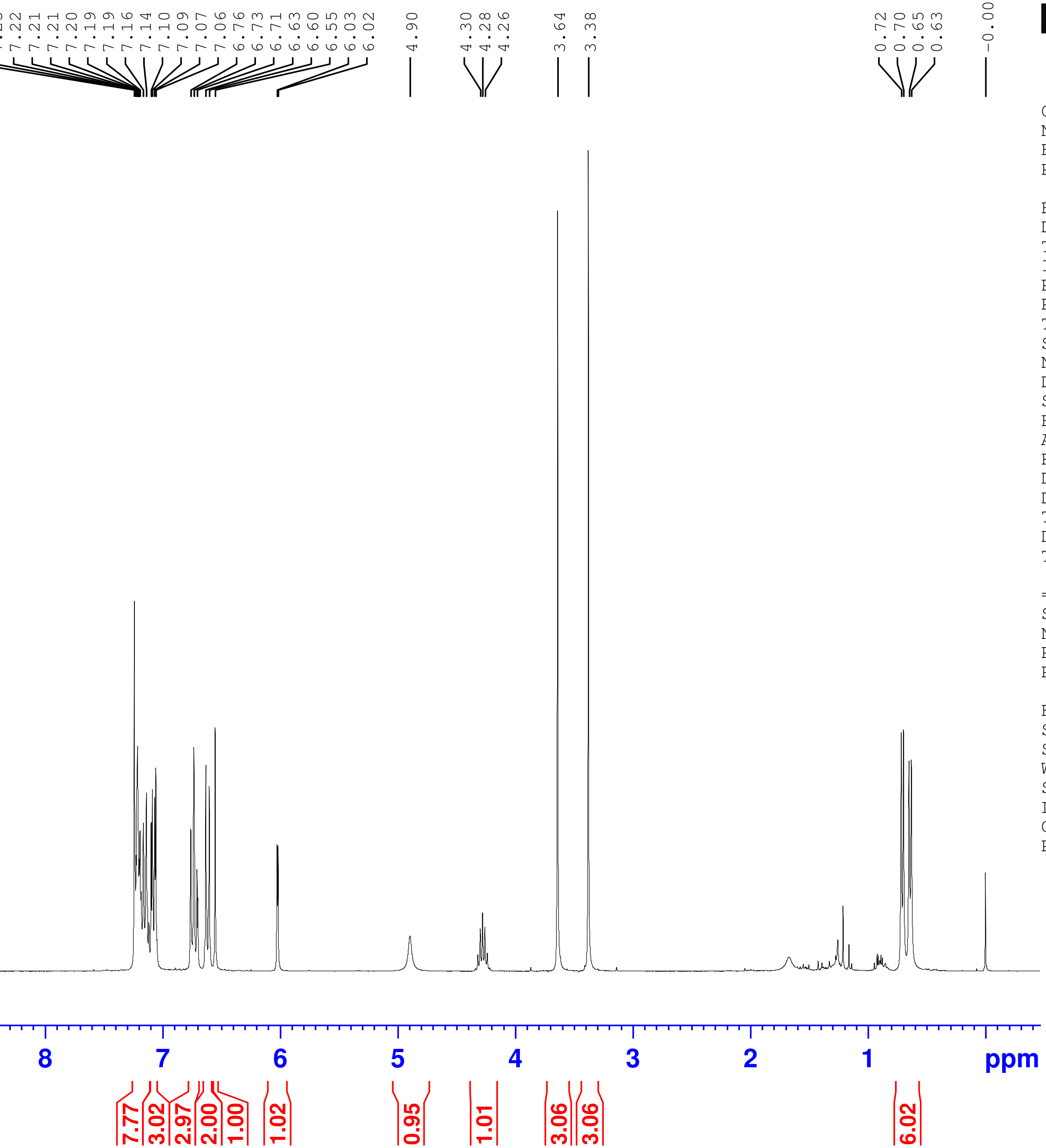

Current Data Parameters
NAME ZY-4-93B-h-fr
EXPNO 5527
PROCNO 1

F2 - Acquisition Parameters
Date_ 20211015
Time 9.57
INSTRUM spect
PROBHD 5 mm PABBO BB-
PULPROG zg30
TD 65536
SOLVENT CDCl3
NS 16
DS 2
SWH 6009.615 Hz
FIDRES 0.091699 Hz
AQ 5.4525952 sec
RG 101
DW 83.200 usec
DE 6.50 usec
TE -59.1 K
D1 1.00000000 sec
TD0 1

======== CHANNEL f1 ========
SFO1 300.1318534 MHz
NUC1 1H
P1 10.00 usec
PLW1 14.00000000 W

F2 - Processing parameters
SI 65536
SF 300.1300132 MHz
WDW EM
SSB 0
LB 0.30 Hz
GB 0
PC 1.00

3pg

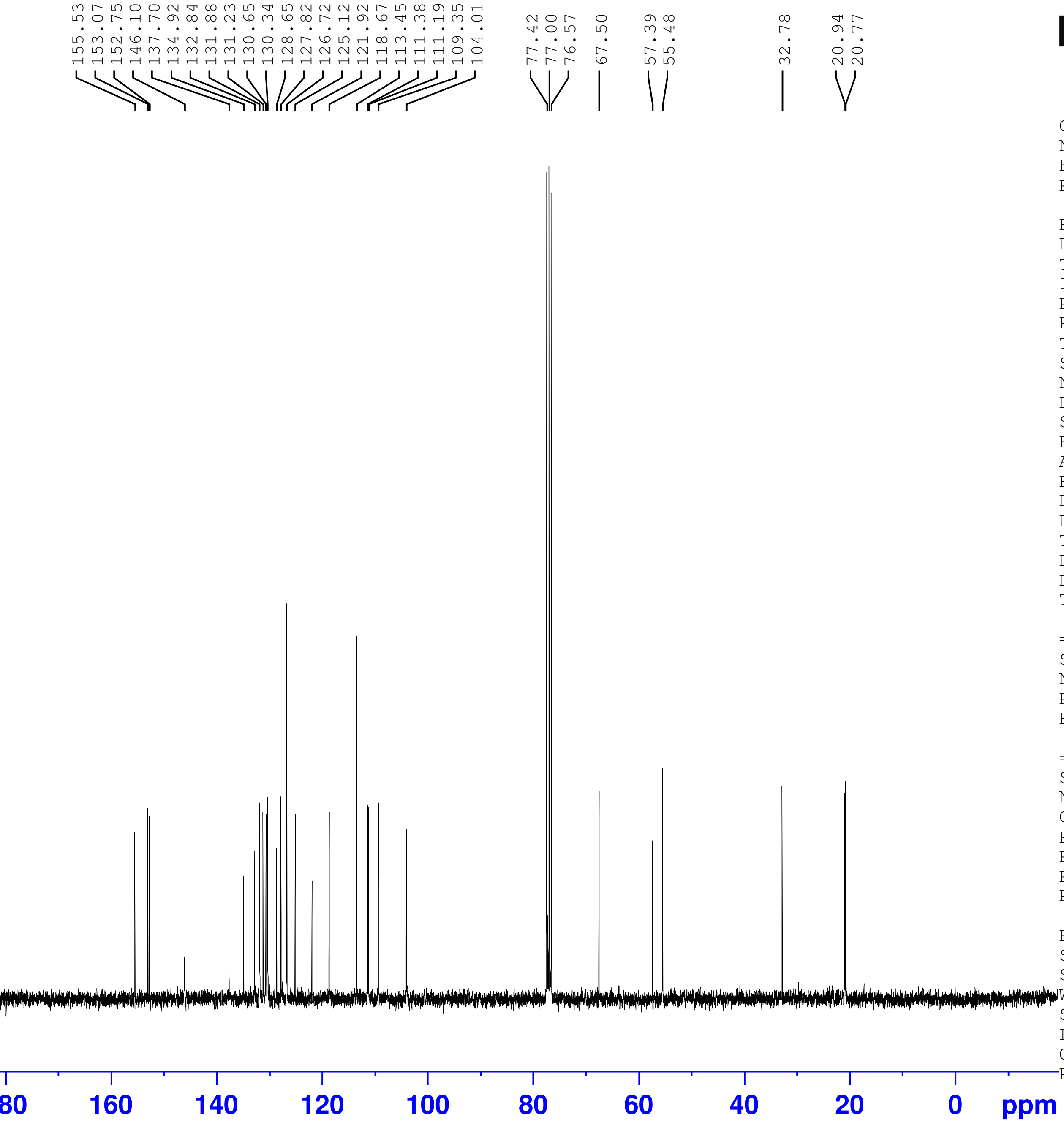

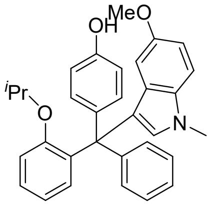

3pg

BRUKER

```
Current Data Parameters
NAME         3qg-ZY-4-93B
EXPNO                5536
PROCNO                  1

F2 - Acquisition Parameters
Date_            20211016
Time                12.30
INSTRUM             spect
PROBHD   5 mm PABBO BB-
PULPROG           zgpg30
TD                  65536
SOLVENT             CDCl3
NS                    500
DS                      4
SWH            18028.846 Hz
FIDRES          0.275098 Hz
AQ             1.8175317 sec
RG                    203
DW                 27.733 usec
DE                   6.50 usec
TE                  -59.1 K
D1            2.00000000 sec
D11           0.03000000 sec
TD0                     1

======== CHANNEL f1 ========
SFO1          75.4752949 MHz
NUC1                  13C
P1                   9.50 usec
PLW1         34.20000076 W

======== CHANNEL f2 ========
SFO2         300.1312005 MHz
NUC2                   1H
CPDPRG[2          waltz16
PCPD2               90.00 usec
PLW2         14.00000000 W
PLW12         0.17284000 W
PLW13         0.14000000 W

F2 - Processing parameters
SI                  32768
SF            75.4677539 MHz
WDW                    EM
SSB                     0
LB                   1.00 Hz
GB                      0
PC                   1.40
```

3qg

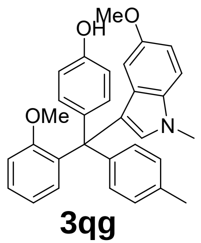

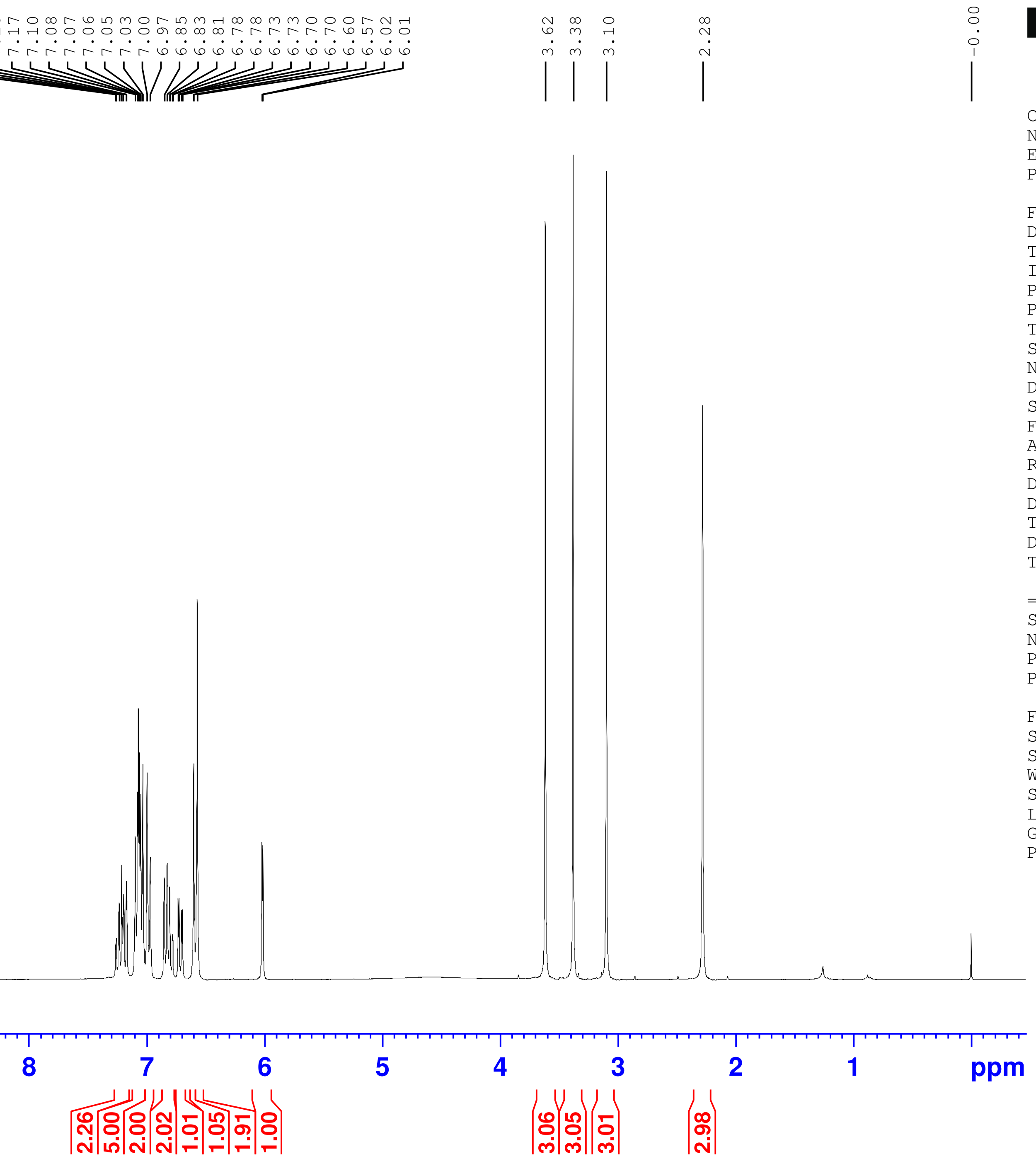

Current Data Parameters
NAME ZY-4-86A-h-fr
EXPNO 5540
PROCNO 1

F2 - Acquisition Parameters
Date_ 20211020
Time 9.22
INSTRUM spect
PROBHD 5 mm PABBO BB-
PULPROG zg30
TD 65536
SOLVENT CDCl3
NS 16
DS 2
SWH 6009.615 Hz
FIDRES 0.091699 Hz
AQ 5.4525952 sec
RG 71.8
DW 83.200 usec
DE 6.50 usec
TE -59.1 K
D1 1.00000000 sec
TD0 1

======== CHANNEL f1 ========
SFO1 300.1318534 MHz
NUC1 1H
P1 10.00 usec
PLW1 14.00000000 W

F2 - Processing parameters
SI 65536
SF 300.1300212 MHz
WDW EM
SSB 0
LB 0.30 Hz
GB 0
PC 1.00

3qg

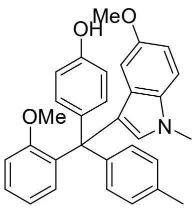

3qg

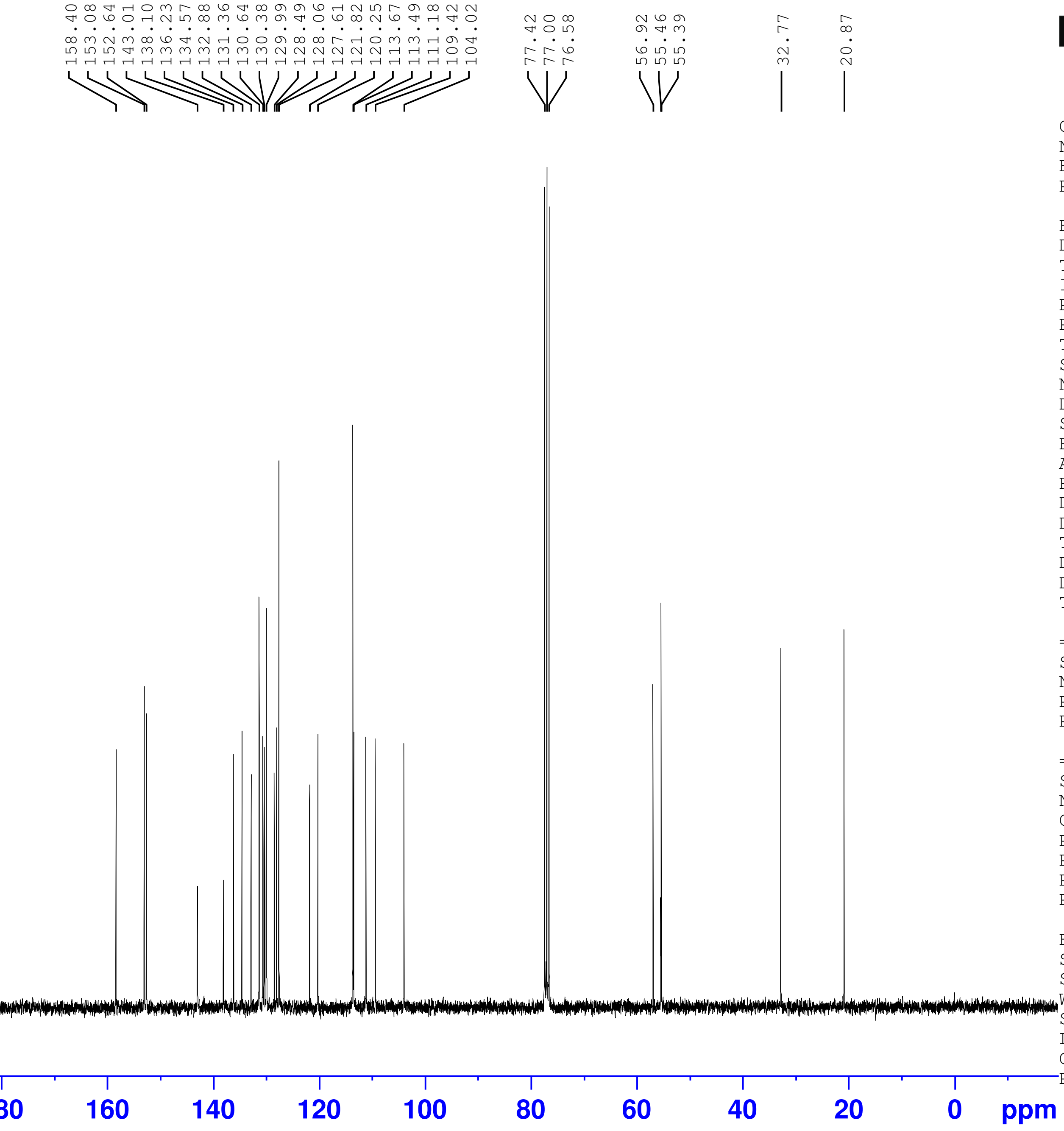

BRUKER

Current Data Parameters
NAME 3rg-ZY-4-86A
EXPNO 5561
PROCNO 1

F2 - Acquisition Parameters
Date_ 20211022
Time 11.34
INSTRUM spect
PROBHD 5 mm PABBO BB-
PULPROG zgpg30
TD 65536
SOLVENT CDCl3
NS 500
DS 4
SWH 18028.846 Hz
FIDRES 0.275098 Hz
AQ 1.8175317 sec
RG 203
DW 27.733 usec
DE 6.50 usec
TE -59.1 K
D1 2.00000000 sec
D11 0.03000000 sec
TD0 1

======== CHANNEL f1 ========
SFO1 75.4752949 MHz
NUC1 13C
P1 9.50 usec
PLW1 34.20000076 W

======== CHANNEL f2 ========
SFO2 300.1312005 MHz
NUC2 1H
CPDPRG[2 waltz16
PCPD2 90.00 usec
PLW2 14.00000000 W
PLW12 0.17284000 W
PLW13 0.14000000 W

F2 - Processing parameters
SI 32768
SF 75.4677576 MHz
WDW EM
SSB 0
LB 1.00 Hz
GB 0
PC 1.40

3rg

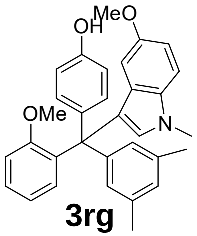

3rg

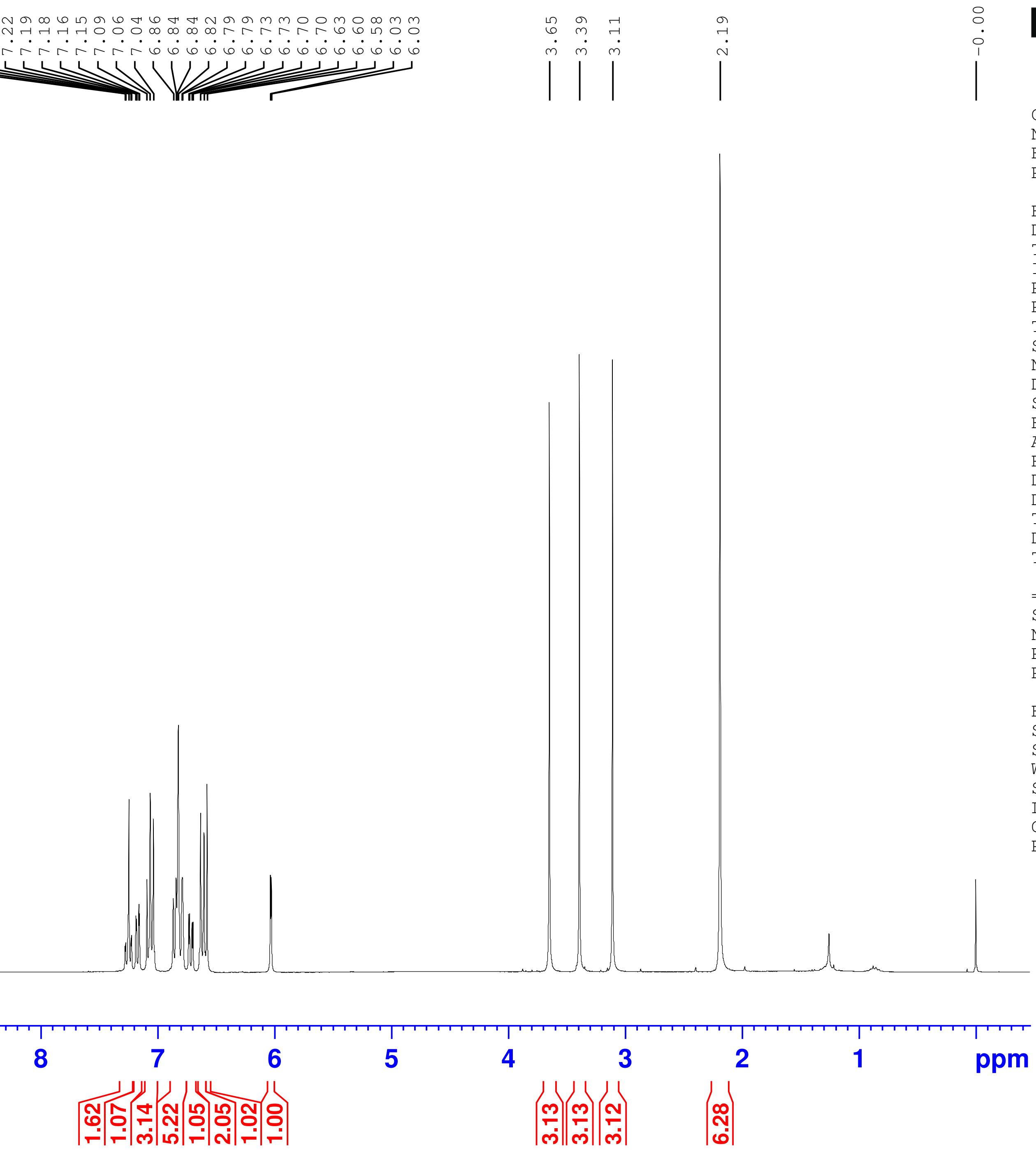

Current Data Parameters
NAME ZY-4-93A-h-fr
EXPNO 5526
PROCNO 1

F2 - Acquisition Parameters
Date_ 20211015
Time 9.52
INSTRUM spect
PROBHD 5 mm PABBO BB-
PULPROG zg30
TD 65536
SOLVENT CDCl3
NS 16
DS 2
SWH 6009.615 Hz
FIDRES 0.091699 Hz
AQ 5.4525952 sec
RG 128
DW 83.200 usec
DE 6.50 usec
TE -59.1 K
D1 1.00000000 sec
TD0 1

======== CHANNEL f1 ========
SFO1 300.1318534 MHz
NUC1 1H
P1 10.00 usec
PLW1 14.00000000 W

F2 - Processing parameters
SI 65536
SF 300.1300119 MHz
WDW EM
SSB 0
LB 0.30 Hz
GB 0
PC 1.00

3rg

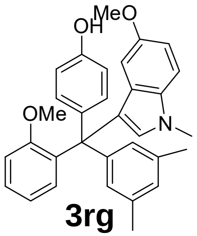

3rg

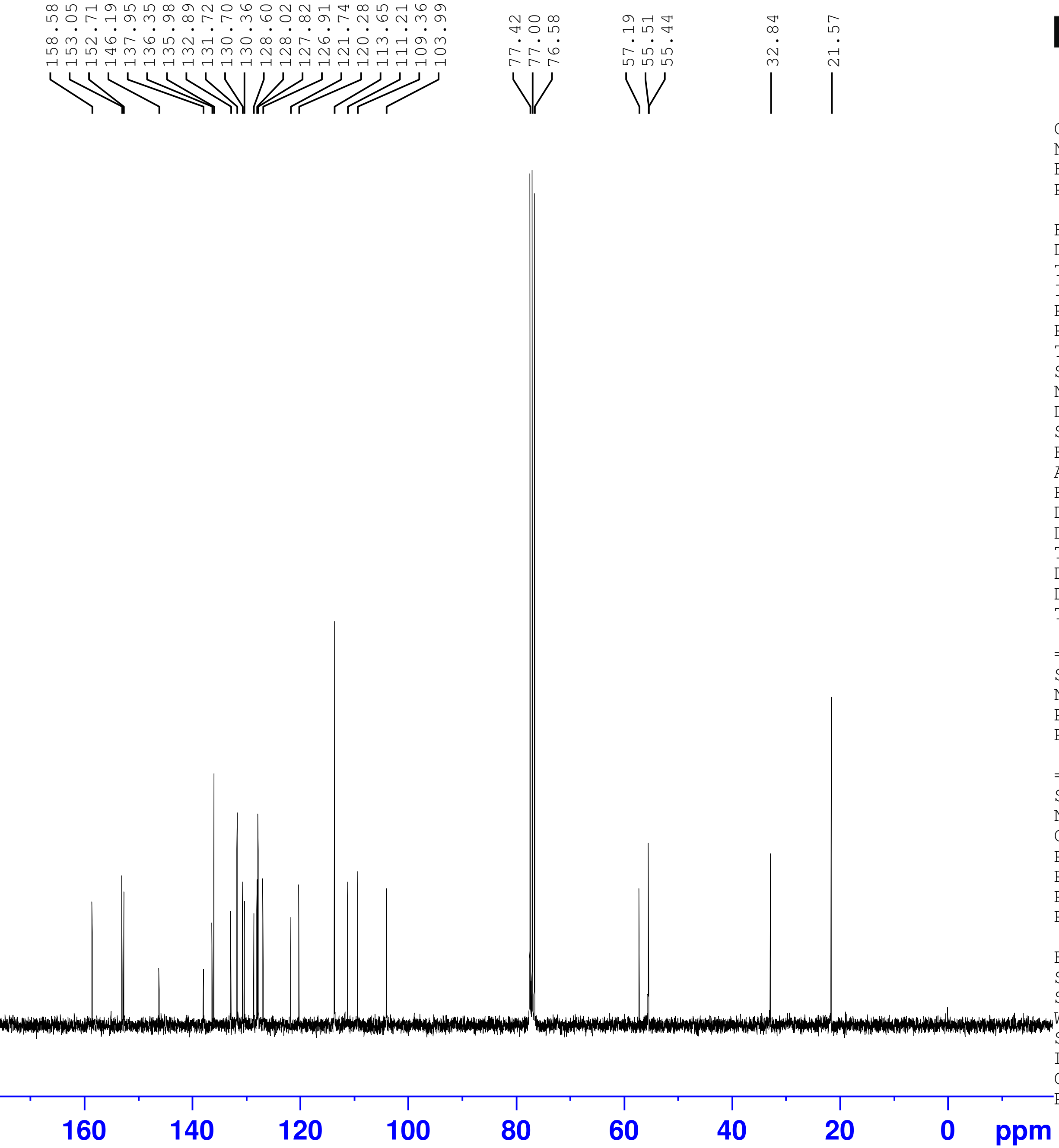

Current Data Parameters
NAME 3tg-ZY-4-93A
EXPNO 5535
PROCNO 1

F2 - Acquisition Parameters
Date_ 20211016
Time 11.55
INSTRUM spect
PROBHD 5 mm PABBO BB-
PULPROG zgpg30
TD 65536
SOLVENT CDCl3
NS 500
DS 4
SWH 18028.846 Hz
FIDRES 0.275098 Hz
AQ 1.8175317 sec
RG 203
DW 27.733 usec
DE 6.50 usec
TE -59.1 K
D1 2.00000000 sec
D11 0.03000000 sec
TD0 1

======== CHANNEL f1 ========
SFO1 75.4752949 MHz
NUC1 13C
P1 9.50 usec
PLW1 34.20000076 W

======== CHANNEL f2 ========
SFO2 300.1312005 MHz
NUC2 1H
CPDPRG[2 waltz16
PCPD2 90.00 usec
PLW2 14.00000000 W
PLW12 0.17284000 W
PLW13 0.14000000 W

F2 - Processing parameters
SI 32768
SF 75.4677532 MHz
WDW EM
SSB 0
LB 1.00 Hz
GB 0
PC 1.40

3sg

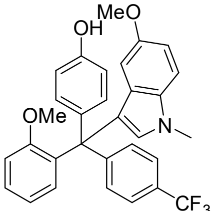

3sg

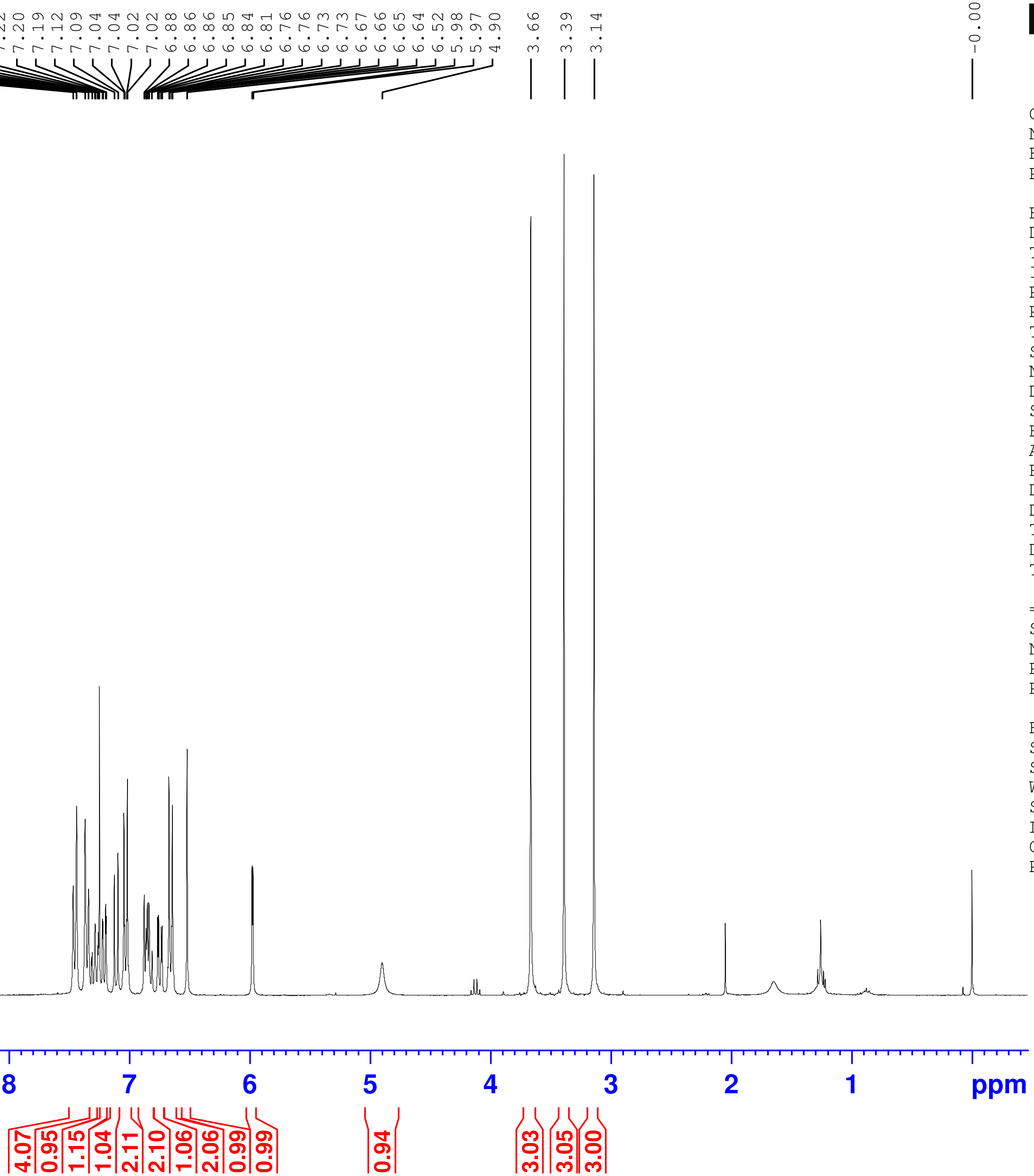

BRUKER

Current Data Parameters
NAME ZY-4-86B-h-fr
EXPNO 5618
PROCNO 1

F2 - Acquisition Parameters
Date_ 20211116
Time 10.35
INSTRUM spect
PROBHD 5 mm PABBO BB-
PULPROG zg30
TD 65536
SOLVENT CDCl3
NS 16
DS 2
SWH 6009.615 Hz
FIDRES 0.091699 Hz
AQ 5.4525952 sec
RG 161
DW 83.200 usec
DE 6.50 usec
TE -59.1 K
D1 1.00000000 sec
TD0 1

======== CHANNEL f1 ========
SFO1 300.1318534 MHz
NUC1 1H
P1 10.00 usec
PLW1 14.00000000 W

F2 - Processing parameters
SI 65536
SF 300.1300108 MHz
WDW EM
SSB 0
LB 0.30 Hz
GB 0
PC 1.00

3sg

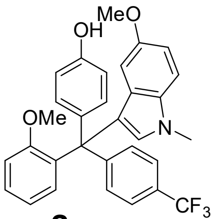

3sg

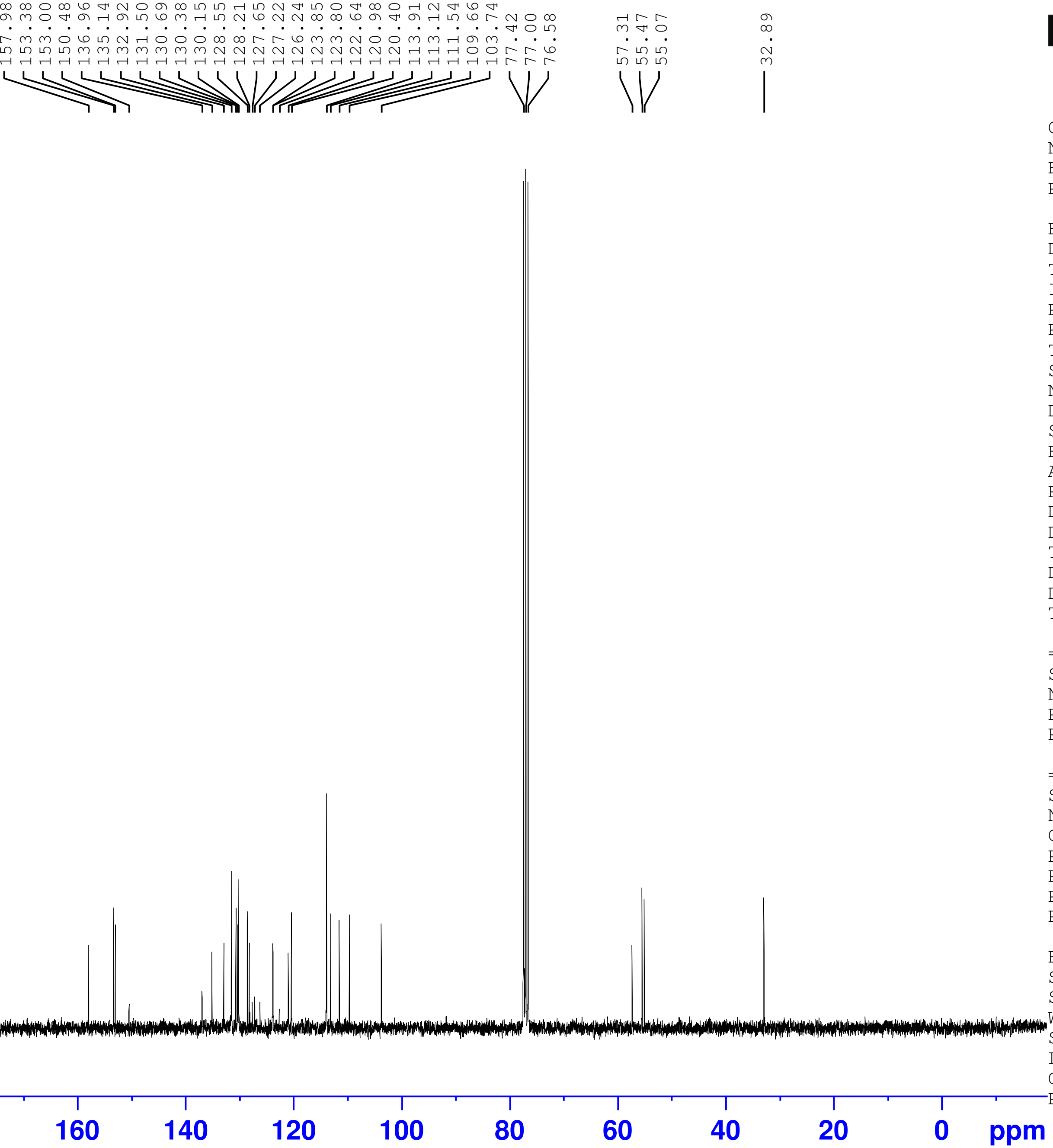

Current Data Parameters
NAME 3sg-ZY-4-86B
EXPNO 5619
PROCNO 1

F2 - Acquisition Parameters
Date_ 20211116
Time 11.09
INSTRUM spect
PROBHD 5 mm PABBO BB-
PULPROG zgpg30
TD 65536
SOLVENT CDCl3
NS 500
DS 4
SWH 18028.846 Hz
FIDRES 0.275098 Hz
AQ 1.8175317 sec
RG 203
DW 27.733 usec
DE 6.50 usec
TE -59.1 K
D1 2.00000000 sec
D11 0.03000000 sec
TD0 1

======== CHANNEL f1 ========
SFO1 75.4752949 MHz
NUC1 13C
P1 9.50 usec
PLW1 34.20000076 W

======== CHANNEL f2 ========
SFO2 300.1312005 MHz
NUC2 1H
CPDPRG[2 waltz16
PCPD2 90.00 usec
PLW2 14.00000000 W
PLW12 0.17284000 W
PLW13 0.14000000 W

F2 - Processing parameters
SI 32768
SF 75.4677524 MHz
WDW EM
SSB 0
LB 1.00 Hz
GB 0
PC 1.40

3sg

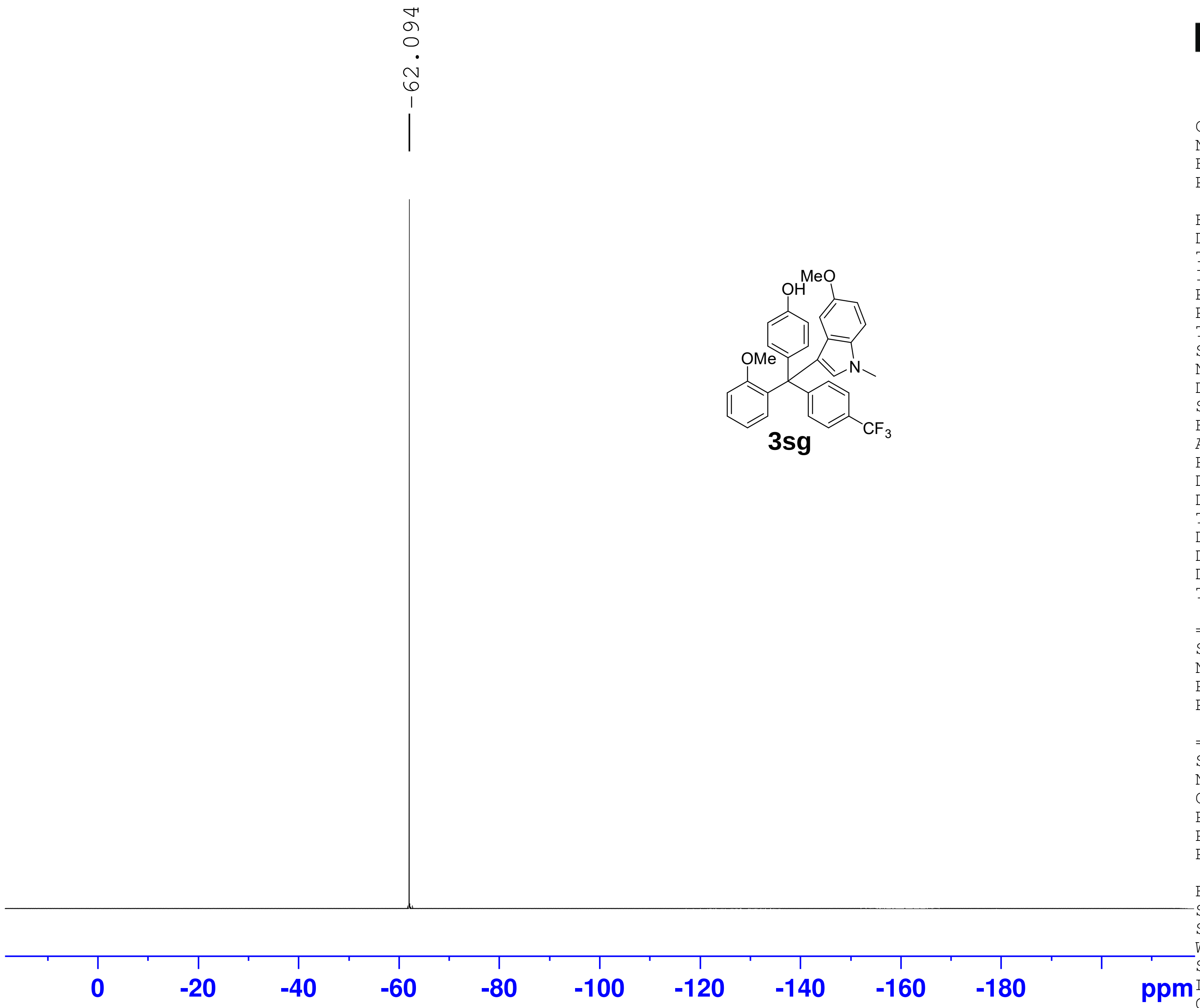

3sg

Current Data Parameters
NAME 211029sjw
EXPNO 5574
PROCNO 1

F2 - Acquisition Parameters
Date_ 20211029
Time 9.32
INSTRUM spect
PROBHD 5 mm PABBO BB-
PULPROG zgfhigqn.2
TD 131072
SOLVENT CDCl3
NS 16
DS 4
SWH 66964.289 Hz
FIDRES 0.510897 Hz
AQ 0.9786710 sec
RG 203
DW 7.467 usec
DE 6.50 usec
TE -59.1 K
D1 1.00000000 sec
D11 0.03000000 sec
D12 0.00002000 sec
TD0 1

======== CHANNEL f1 ========
SFO1 282.3761148 MHz
NUC1 19F
P1 14.50 usec
PLW1 10.39999962 W

======== CHANNEL f2 ========
SFO2 300.1312005 MHz
NUC2 1H
CPDPRG[2 waltz16
PCPD2 90.00 usec
PLW2 14.00000000 W
PLW12 0.17284000 W

F2 - Processing parameters
SI 65536
SF 282.4043552 MHz
WDW EM
SSB 0
LB 0.30 Hz
GB 0
PC 1.00

3tg

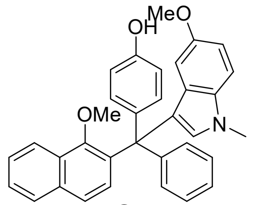

3tg

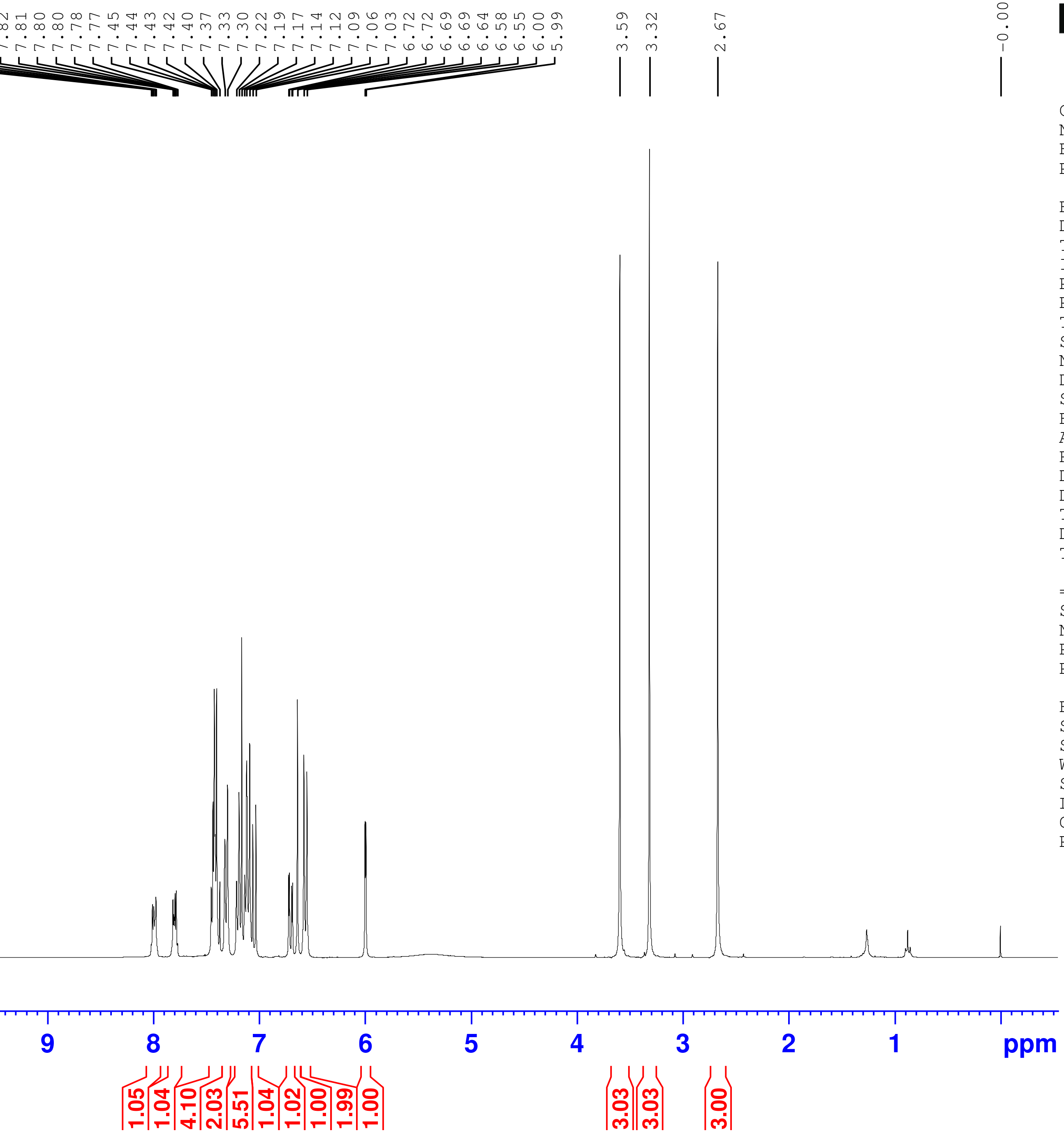

Current Data Parameters
NAME ZY-4-86D-h-fr
EXPNO 5542
PROCNO 1

F2 - Acquisition Parameters
Date_ 20211020
Time 9.33
INSTRUM spect
PROBHD 5 mm PABBO BB-
PULPROG zg30
TD 65536
SOLVENT CDCl3
NS 16
DS 2
SWH 6009.615 Hz
FIDRES 0.091699 Hz
AQ 5.4525952 sec
RG 32
DW 83.200 usec
DE 6.50 usec
TE -59.1 K
D1 1.00000000 sec
TD0 1

======== CHANNEL f1 ========
SFO1 300.1318534 MHz
NUC1 1H
P1 10.00 usec
PLW1 14.00000000 W

F2 - Processing parameters
SI 65536
SF 300.1300352 MHz
WDW EM
SSB 0
LB 0.30 Hz
GB 0
PC 1.00

3tg

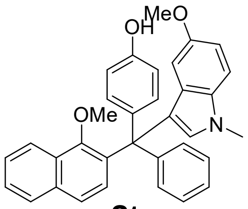

3tg

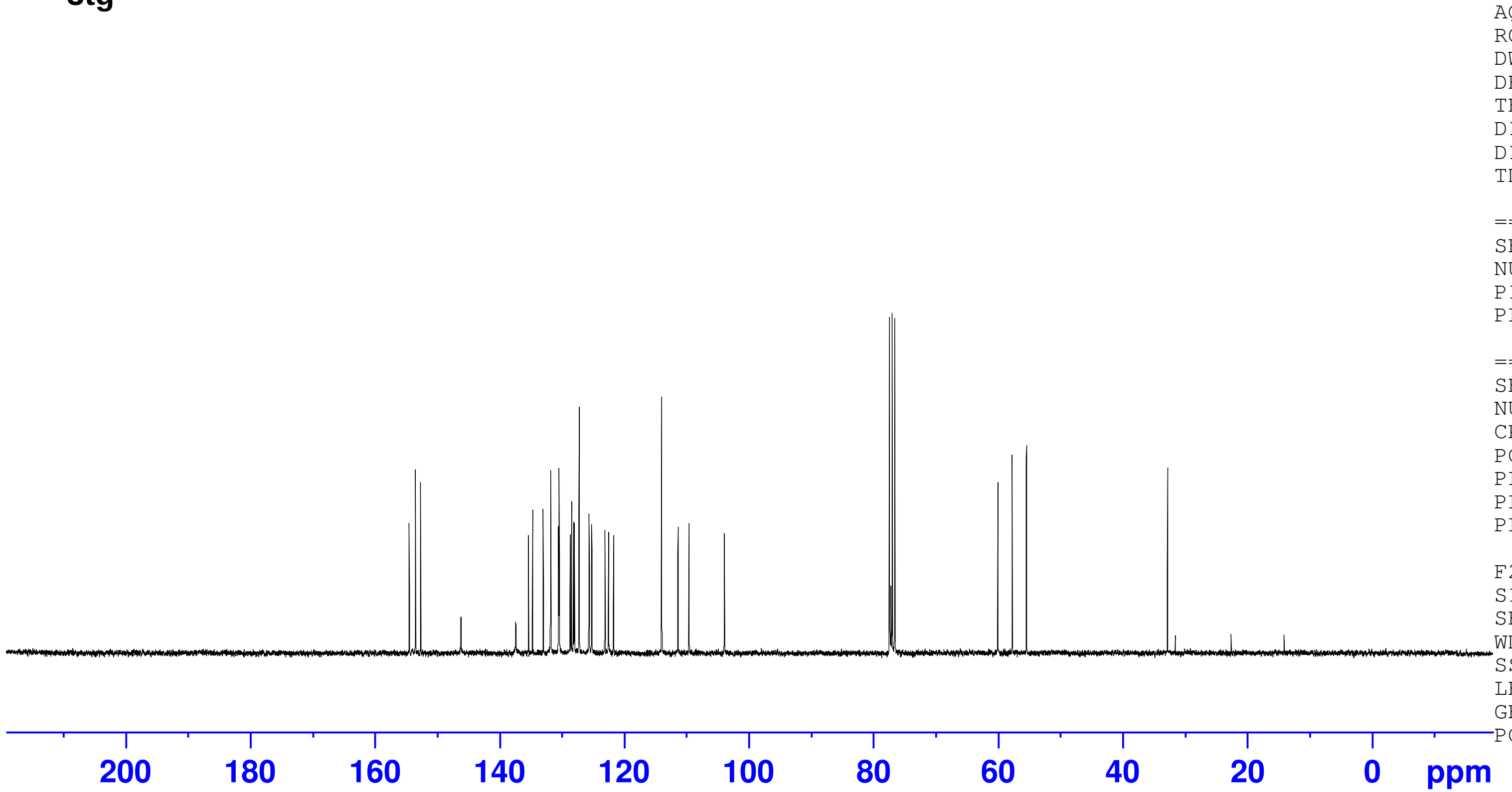

Current Data Parameters
NAME 3pg-ZY-4-86D
EXPNO 5563
PROCNO 1

F2 - Acquisition Parameters
Date_ 20211022
Time 12.45
INSTRUM spect
PROBHD 5 mm PABBO BB-
PULPROG zgpg30
TD 65536
SOLVENT CDCl3
NS 500
DS 4
SWH 18028.846 Hz
FIDRES 0.275098 Hz
AQ 1.8175317 sec
RG 203
DW 27.733 usec
DE 6.50 usec
TE -59.1 K
D1 2.00000000 sec
D11 0.03000000 sec
TD0 1

======== CHANNEL f1 ========
SFO1 75.4752949 MHz
NUC1 13C
P1 9.50 usec
PLW1 34.20000076 W

======== CHANNEL f2 ========
SFO2 300.1312005 MHz
NUC2 1H
CPDPRG[2 waltz16
PCPD2 90.00 usec
PLW2 14.00000000 W
PLW12 0.17284000 W
PLW13 0.14000000 W

F2 - Processing parameters
SI 32768
SF 75.4677624 MHz
WDW EM
SSB 0
LB 1.00 Hz
GB 0
PC 1.40

4da

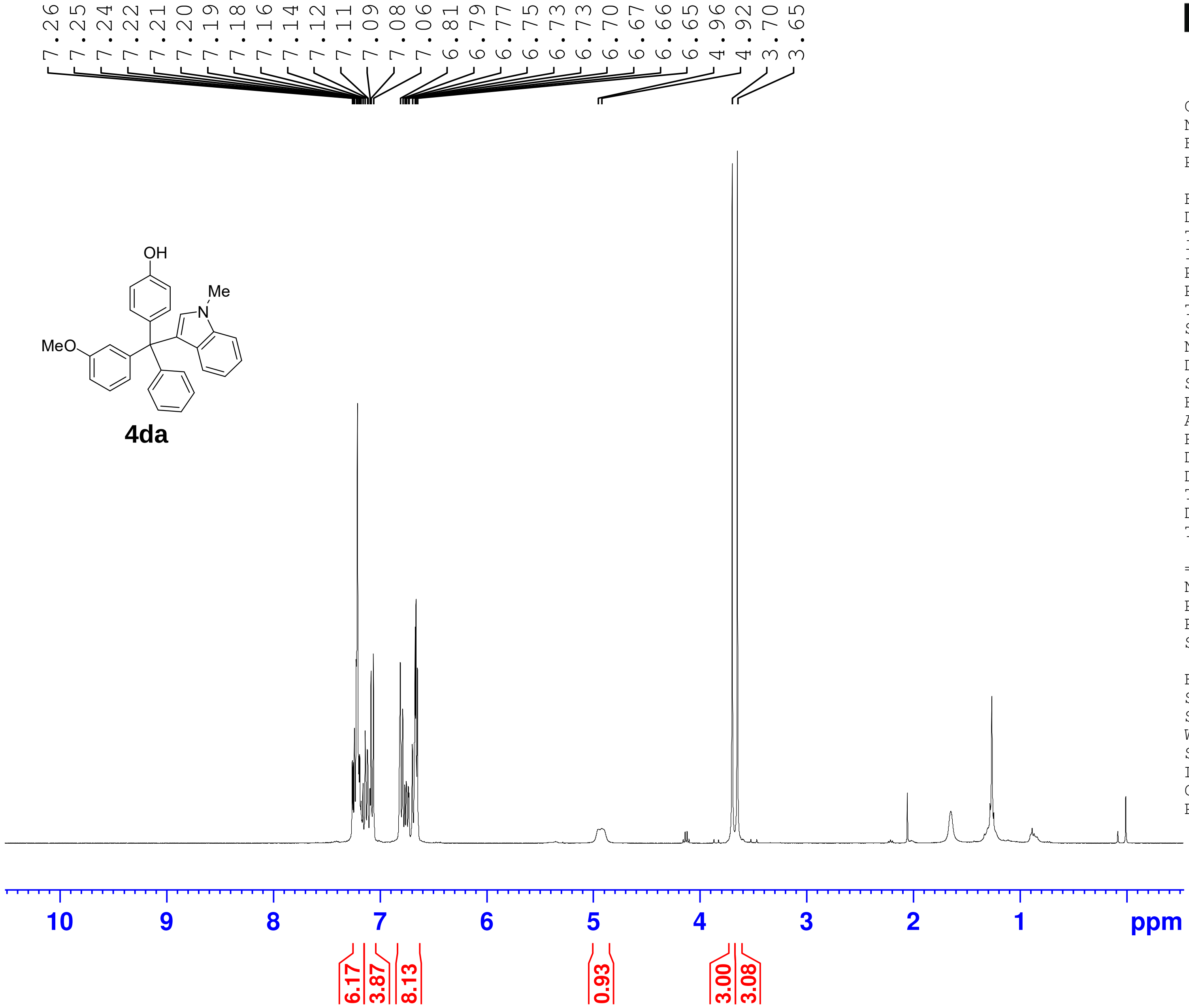

4da

Current Data Parameters
NAME 0808-400
EXPNO 188
PROCNO 1

F2 - Acquisition Parameters
Date_ 20220808
Time 21.38
INSTRUM spect
PROBHD 5 mm PABBO BB-
PULPROG zg30
TD 65536
SOLVENT CDCl3
NS 8
DS 0
SWH 8223.685 Hz
FIDRES 0.125483 Hz
AQ 3.9845889 sec
RG 125.76
DW 60.800 usec
DE 6.50 usec
TE 294.2 K
D1 1.00000000 sec
TD0 1

======== CHANNEL f1 ========
NUC1 1H
P1 14.68 usec
PLW1 14.00000000 W
SFO1 400.1924713 MHz

F2 - Processing parameters
SI 65536
SF 400.1900204 MHz
WDW EM
SSB 0
LB 0.30 Hz
GB 0
PC 1.00

4da

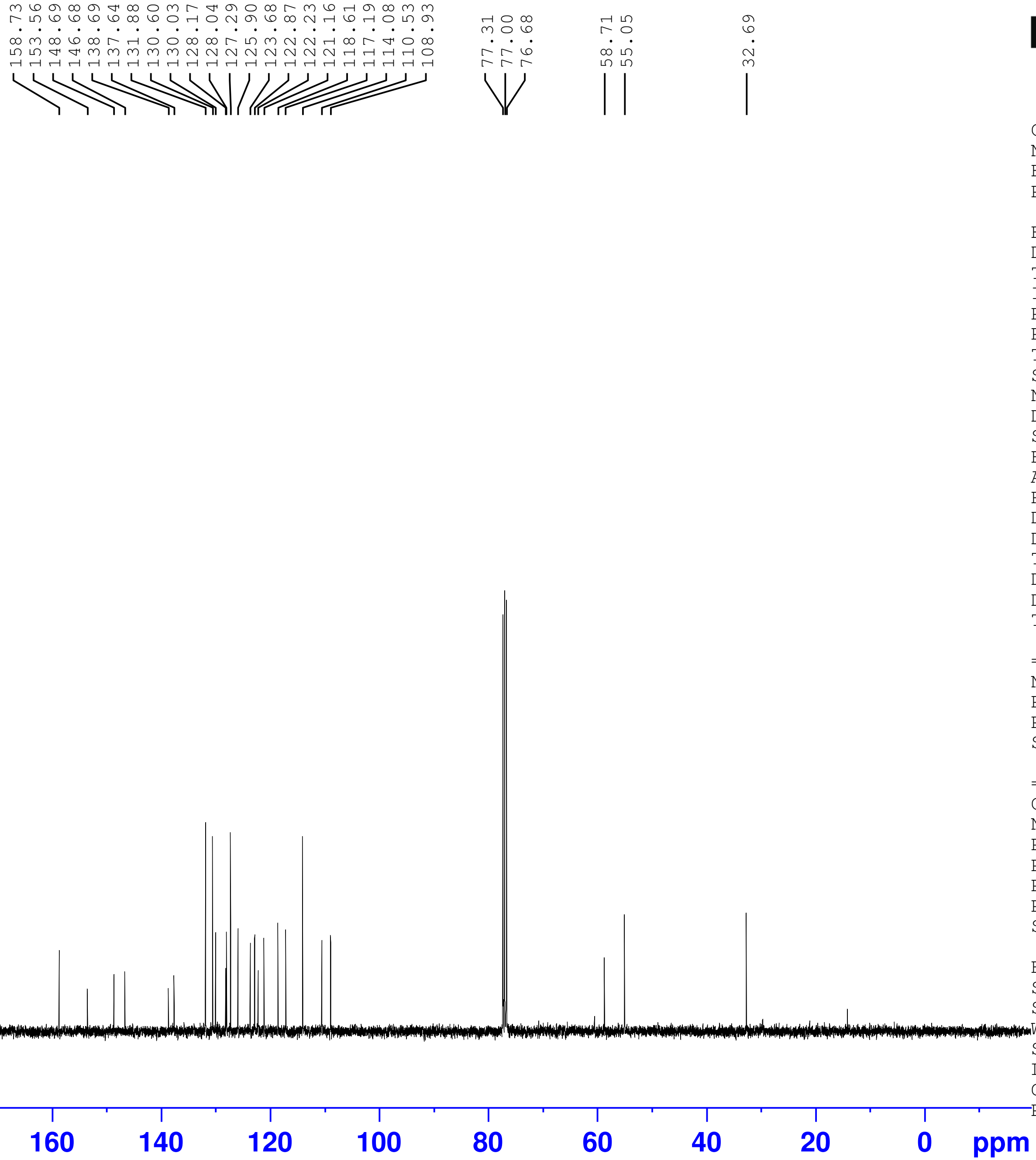

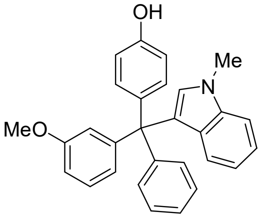

4da

Current Data Parameters
NAME 0729-400(2)
EXPNO 115
PROCNO 1

F2 - Acquisition Parameters
Date_ 20220729
Time 22.10
INSTRUM spect
PROBHD 5 mm PABBO BB-
PULPROG zgpg30
TD 65536
SOLVENT CDCl3
NS 140
DS 4
SWH 24038.461 Hz
FIDRES 0.366798 Hz
AQ 1.3631488 sec
RG 193.13
DW 20.800 usec
DE 6.50 usec
TE 295.4 K
D1 2.00000000 sec
D11 0.03000000 sec
TD0 1

======== CHANNEL f1 ========
NUC1 13C
P1 12.00 usec
PLW1 53.00000000 W
SFO1 100.6379178 MHz

======== CHANNEL f2 ========
CPDPRG[2 waltz16
NUC2 1H
PCPD2 90.00 usec
PLW2 14.00000000 W
PLW12 0.37246999 W
PLW13 0.30170000 W
SFO2 400.1916008 MHz

F2 - Processing parameters
SI 32768
SF 100.6278643 MHz
WDW EM
SSB 0
LB 1.00 Hz
GB 0
PC 1.40

4ea

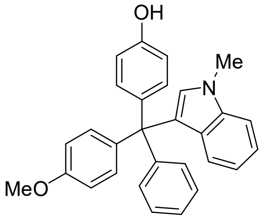

4ea

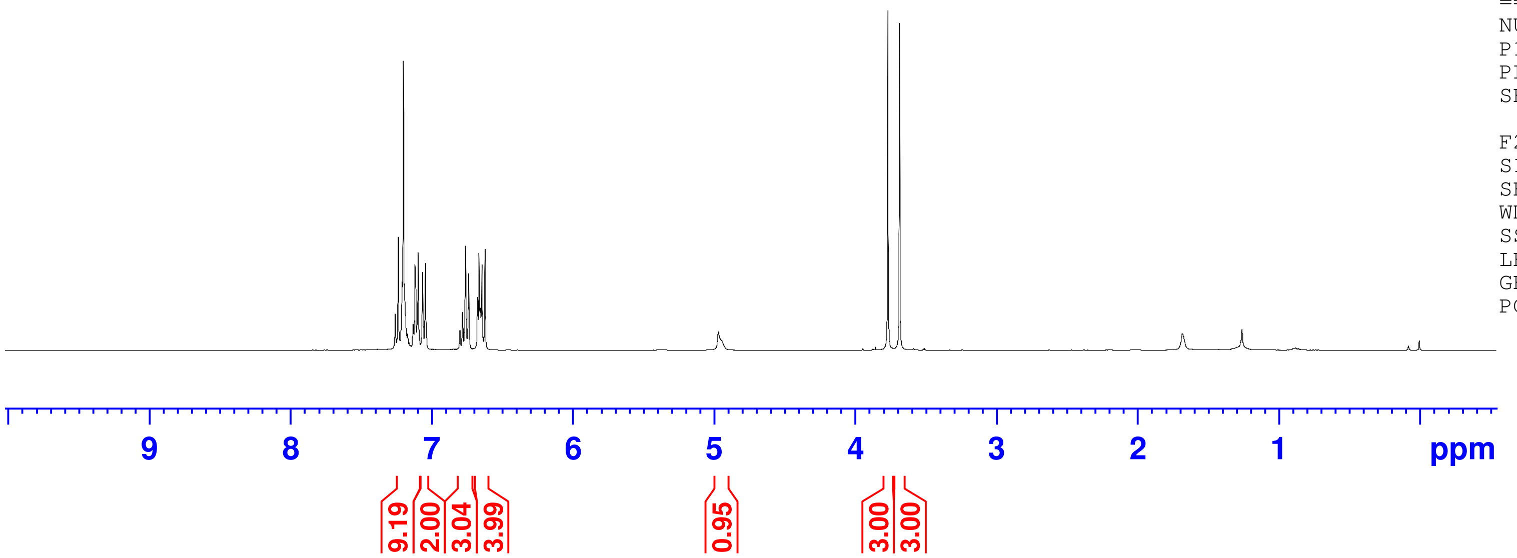

BRUKER

Current Data Parameters
NAME 0729-400(2)
EXPNO 116
PROCNO 1

F2 - Acquisition Parameters
Date_ 20220729
Time 22.19
INSTRUM spect
PROBHD 5 mm PABBO BB-
PULPROG zg30
TD 65536
SOLVENT CDCl3
NS 24
DS 0
SWH 8223.685 Hz
FIDRES 0.125483 Hz
AQ 3.9845889 sec
RG 100.49
DW 60.800 usec
DE 6.50 usec
TE 294.8 K
D1 1.00000000 sec
TD0 1

======== CHANNEL f1 ========
NUC1 1H
P1 14.68 usec
PLW1 14.00000000 W
SFO1 400.1924713 MHz

F2 - Processing parameters
SI 65536
SF 400.1900260 MHz
WDW EM
SSB 0
LB 0.30 Hz
GB 0
PC 1.00

4ea

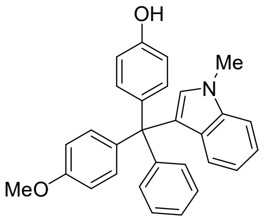

4ea

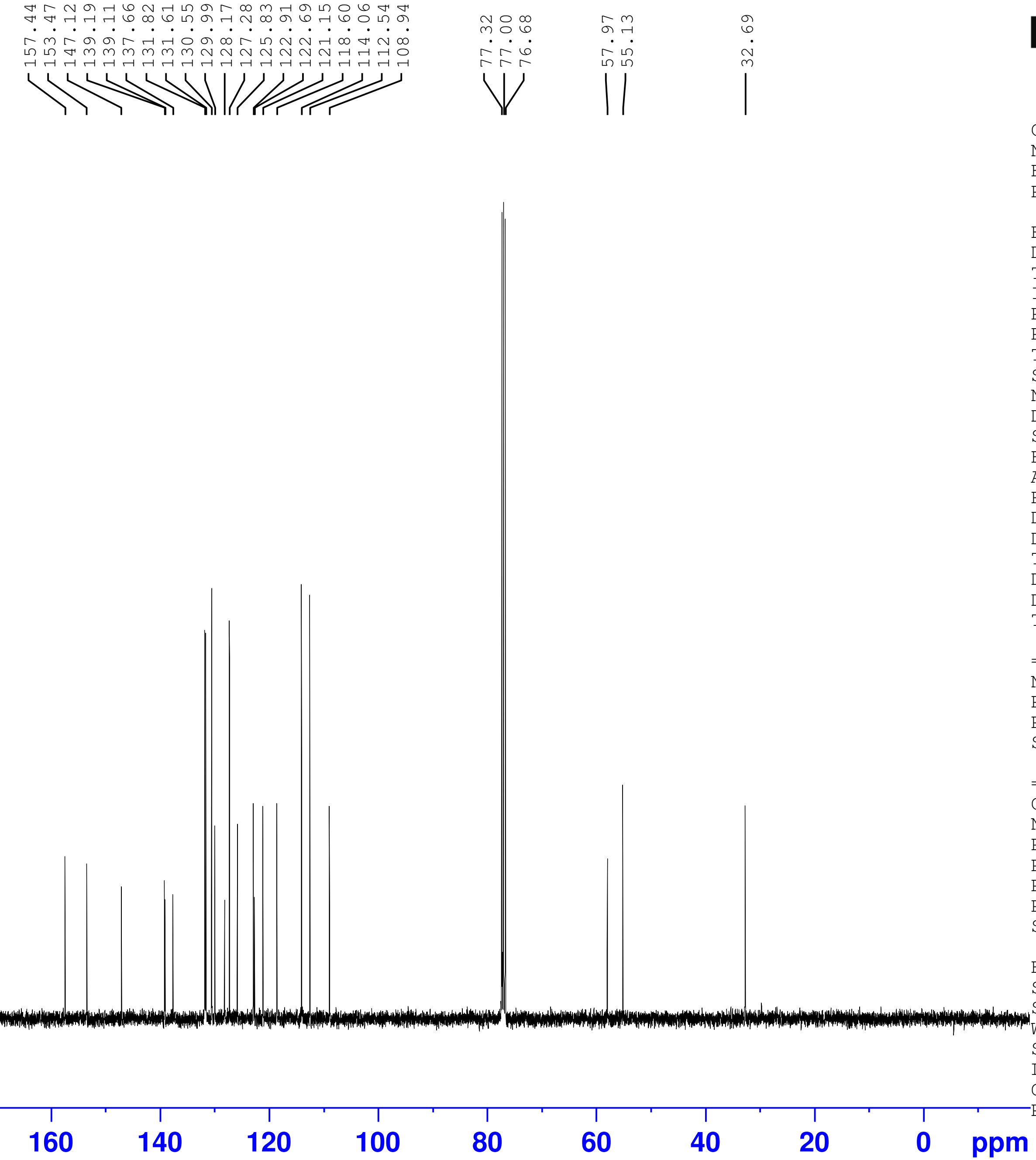

Current Data Parameters
NAME 0729-400(2)
EXPNO 117
PROCNO 1

F2 - Acquisition Parameters
Date_ 20220729
Time 22.32
INSTRUM spect
PROBHD 5 mm PABBO BB-
PULPROG zgpg30
TD 65536
SOLVENT CDCl3
NS 200
DS 4
SWH 24038.461 Hz
FIDRES 0.366798 Hz
AQ 1.3631488 sec
RG 193.13
DW 20.800 usec
DE 6.50 usec
TE 295.5 K
D1 2.00000000 sec
D11 0.03000000 sec
TD0 1

======== CHANNEL f1 ========
NUC1 13C
P1 12.00 usec
PLW1 53.00000000 W
SFO1 100.6379178 MHz

======== CHANNEL f2 ========
CPDPRG[2 waltz16
NUC2 1H
PCPD2 90.00 usec
PLW2 14.00000000 W
PLW12 0.37246999 W
PLW13 0.30170000 W
SFO2 400.1916008 MHz

F2 - Processing parameters
SI 32768
SF 100.6278644 MHz
WDW EM
SSB 0
LB 1.00 Hz
GB 0
PC 1.40

4fa

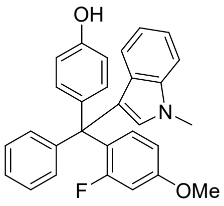

4fa

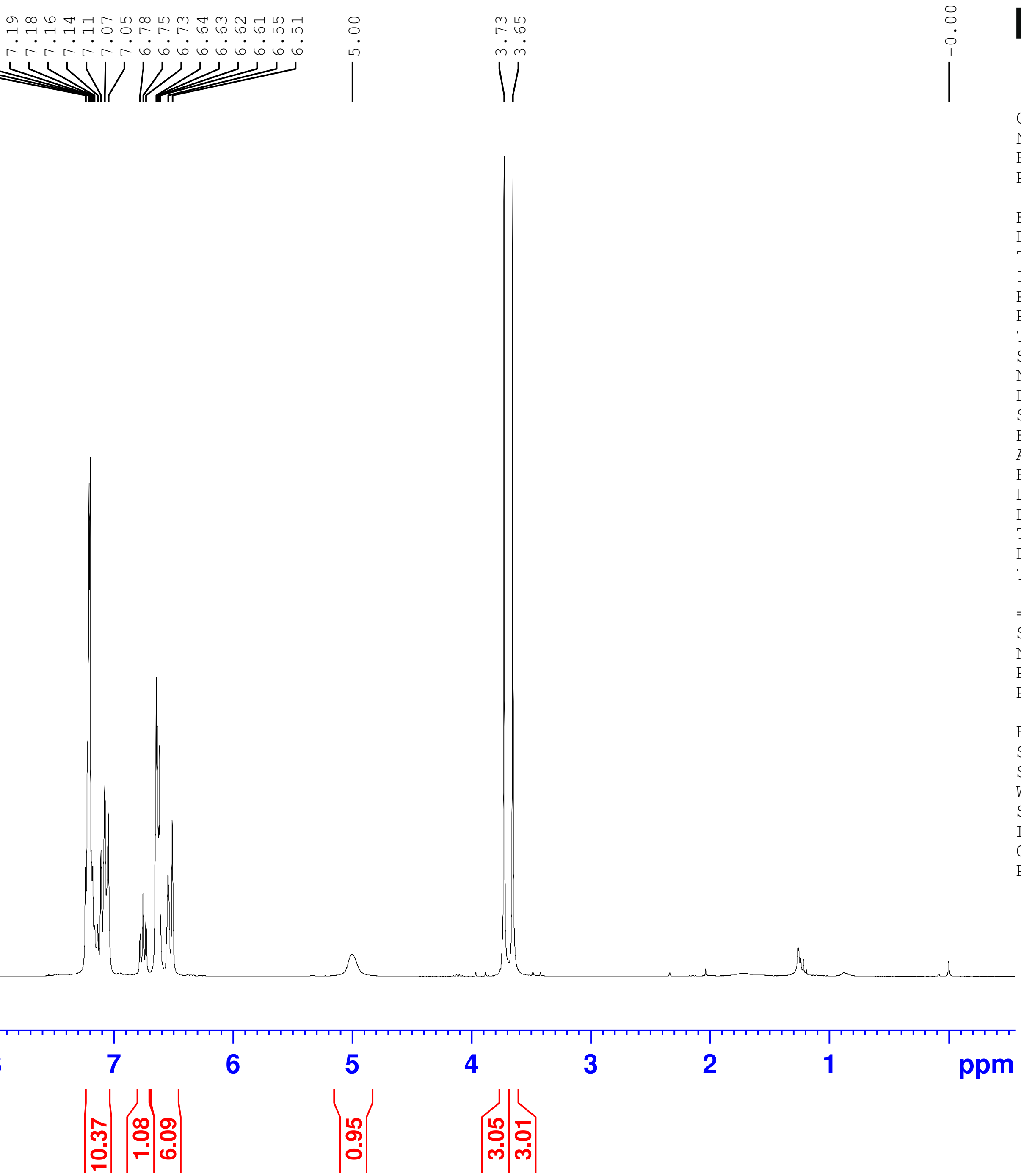


```
Current Data Parameters
NAME        ZY-4-56B-h-fr
EXPNO                5620
PROCNO                  1

F2 - Acquisition Parameters
Date_            20211116
Time                11.14
INSTRUM             spect
PROBHD    5 mm PABBO BB-
PULPROG              zg30
TD                  65536
SOLVENT             CDCl3
NS                     16
DS                      2
SWH              6009.615 Hz
FIDRES           0.091699 Hz
AQ              5.4525952 sec
RG                   71.8
DW                 83.200 usec
DE                   6.50 usec
TE                  -59.1 K
D1             1.00000000 sec
TD0                     1

======== CHANNEL f1 ========
SFO1          300.1318534 MHz
NUC1                   1H
P1                  10.00 usec
PLW1          14.00000000 W

F2 - Processing parameters
SI                  65536
SF            300.1300257 MHz
WDW                    EM
SSB                     0
LB                   0.30 Hz
GB                      0
PC                   1.00
```

4fa

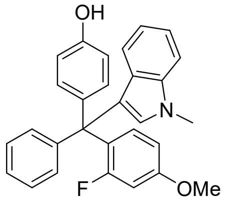

4fa

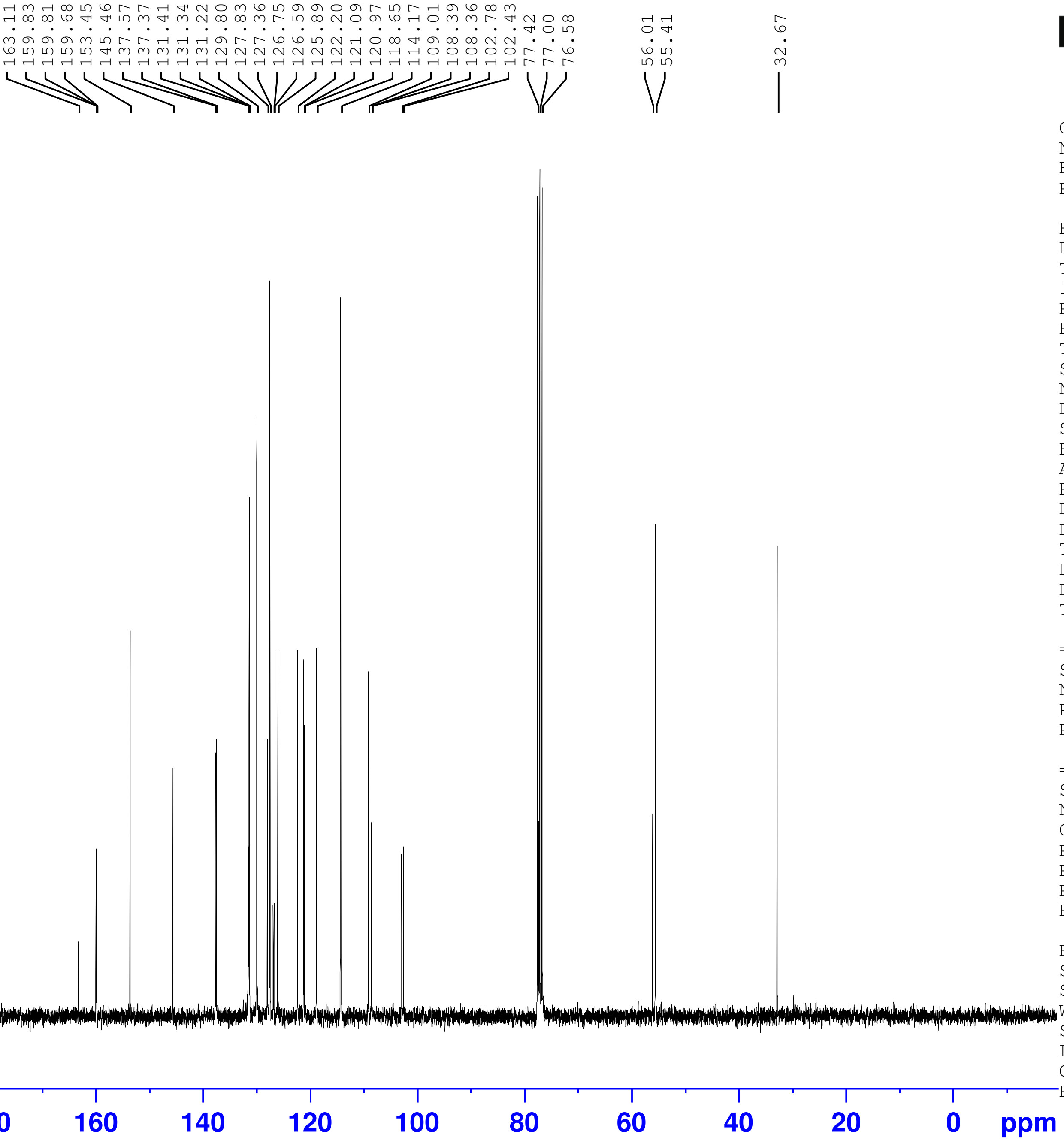


```
Current Data Parameters
NAME        ZY-4-56B-c-fr
EXPNO                5622
PROCNO                  1

F2 - Acquisition Parameters
Date_            20211116
Time                11.50
INSTRUM             spect
PROBHD   5 mm PABBO BB-
PULPROG           zgpg30
TD                  65536
SOLVENT             CDCl3
NS                    500
DS                      4
SWH             18028.846 Hz
FIDRES           0.275098 Hz
AQ              1.8175317 sec
RG                    203
DW                 27.733 usec
DE                   6.50 usec
TE                  -59.1 K
D1             2.00000000 sec
D11            0.03000000 sec
TD0                     1

======== CHANNEL f1 ========
SFO1          75.4752949 MHz
NUC1                  13C
P1                   9.50 usec
PLW1          34.20000076 W

======== CHANNEL f2 ========
SFO2         300.1312005 MHz
NUC2                   1H
CPDPRG[2          waltz16
PCPD2               90.00 usec
PLW2          14.00000000 W
PLW12          0.17284000 W
PLW13          0.14000000 W

F2 - Processing parameters
SI                  32768
SF            75.4677583 MHz
WDW                    EM
SSB                     0
LB                   1.00 Hz
GB                      0
PC                   1.40
```

4fa

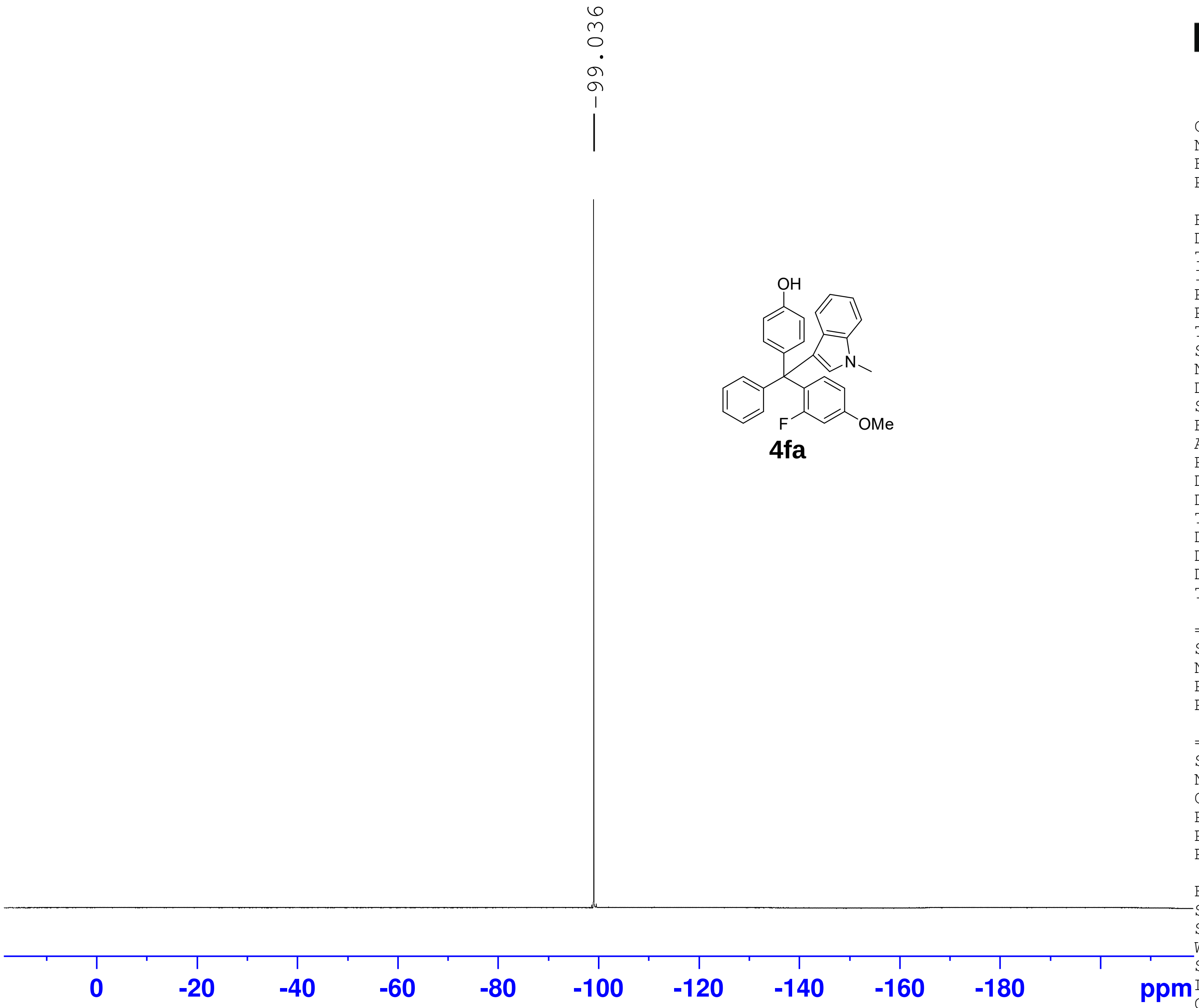

4fa

Current Data Parameters
NAME 211116sjw
EXPNO 5621
PROCNO 1

F2 - Acquisition Parameters
Date_ 20211116
Time 11.16
INSTRUM spect
PROBHD 5 mm PABBO BB-
PULPROG zgfhigqn.2
TD 131072
SOLVENT CDCl3
NS 16
DS 4
SWH 66964.289 Hz
FIDRES 0.510897 Hz
AQ 0.9786710 sec
RG 203
DW 7.467 usec
DE 6.50 usec
TE -59.1 K
D1 1.00000000 sec
D11 0.03000000 sec
D12 0.00002000 sec
TD0 1

======== CHANNEL f1 ========
SFO1 282.3761148 MHz
NUC1 19F
P1 14.50 usec
PLW1 10.39999962 W

======== CHANNEL f2 ========
SFO2 300.1312005 MHz
NUC2 1H
CPDPRG[2 waltz16
PCPD2 90.00 usec
PLW2 14.00000000 W
PLW12 0.17284000 W

F2 - Processing parameters
SI 65536
SF 282.4043552 MHz
WDW EM
SSB 0
LB 0.30 Hz
GB 0
PC 1.00

3ka'

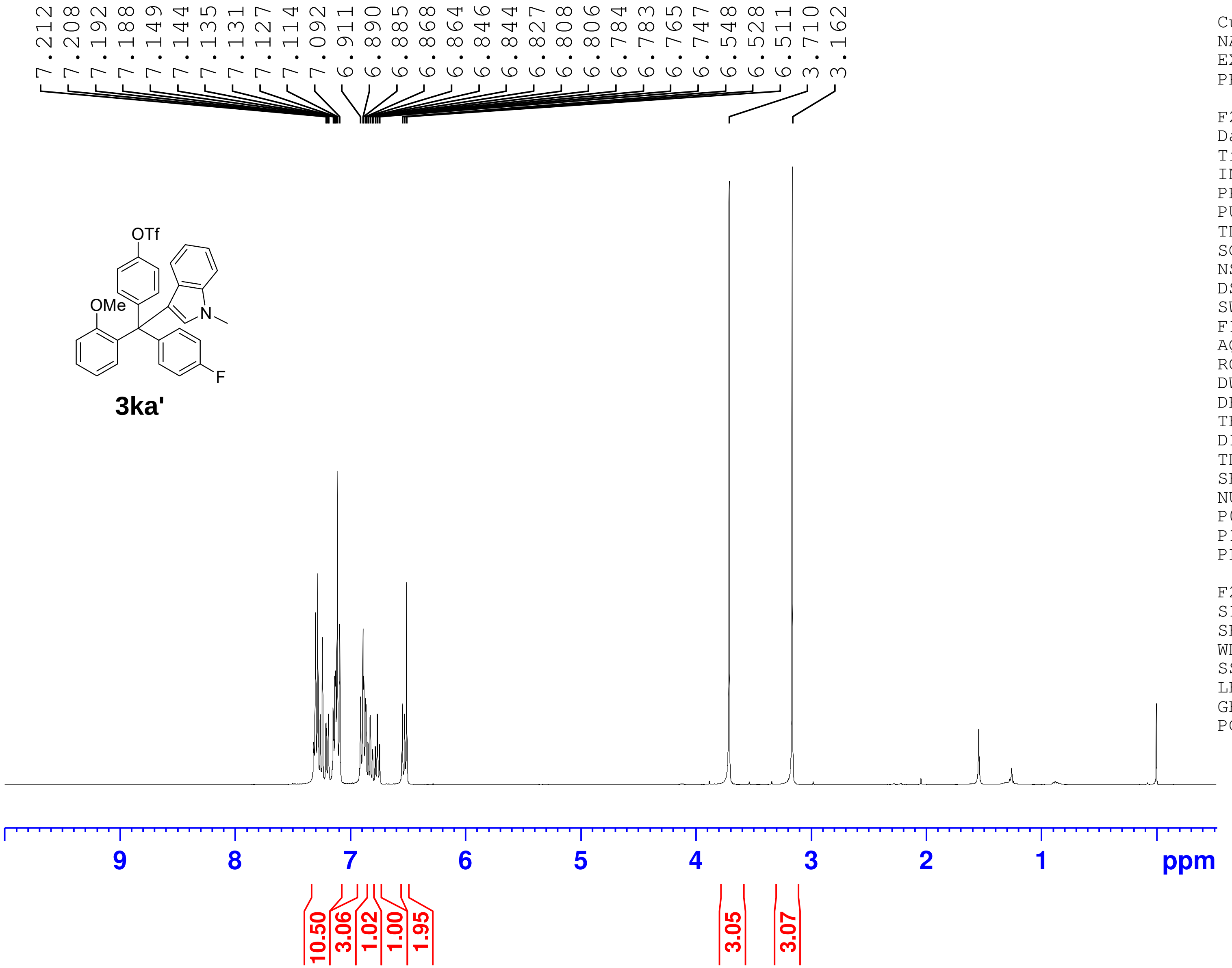

3ka'

Current Data Parameters
NAME ZY-5-41-h-fr
EXPNO 6
PROCNO 1

F2 - Acquisition Parameters
Date_ 20220608
Time 1.51 h
INSTRUM Avance
PROBHD Z116098_0833 (
PULPROG zg30
TD 65536
SOLVENT CDCl3
NS 16
DS 2
SWH 8196.722 Hz
FIDRES 0.250144 Hz
AQ 3.9976959 sec
RG 101
DW 61.000 usec
DE 13.54 usec
TE 292.1 K
D1 1.00000000 sec
TD0 1
SFO1 400.1324708 MHz
NUC1 1H
P0 3.33 usec
P1 10.00 usec
PLW1 20.73200035 W

F2 - Processing parameters
SI 65536
SF 400.1300170 MHz
WDW EM
SSB 0
LB 0.30 Hz
GB 0
PC 1.00

3ka'

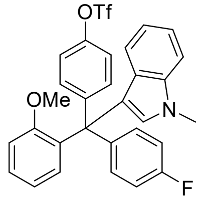

3ka'

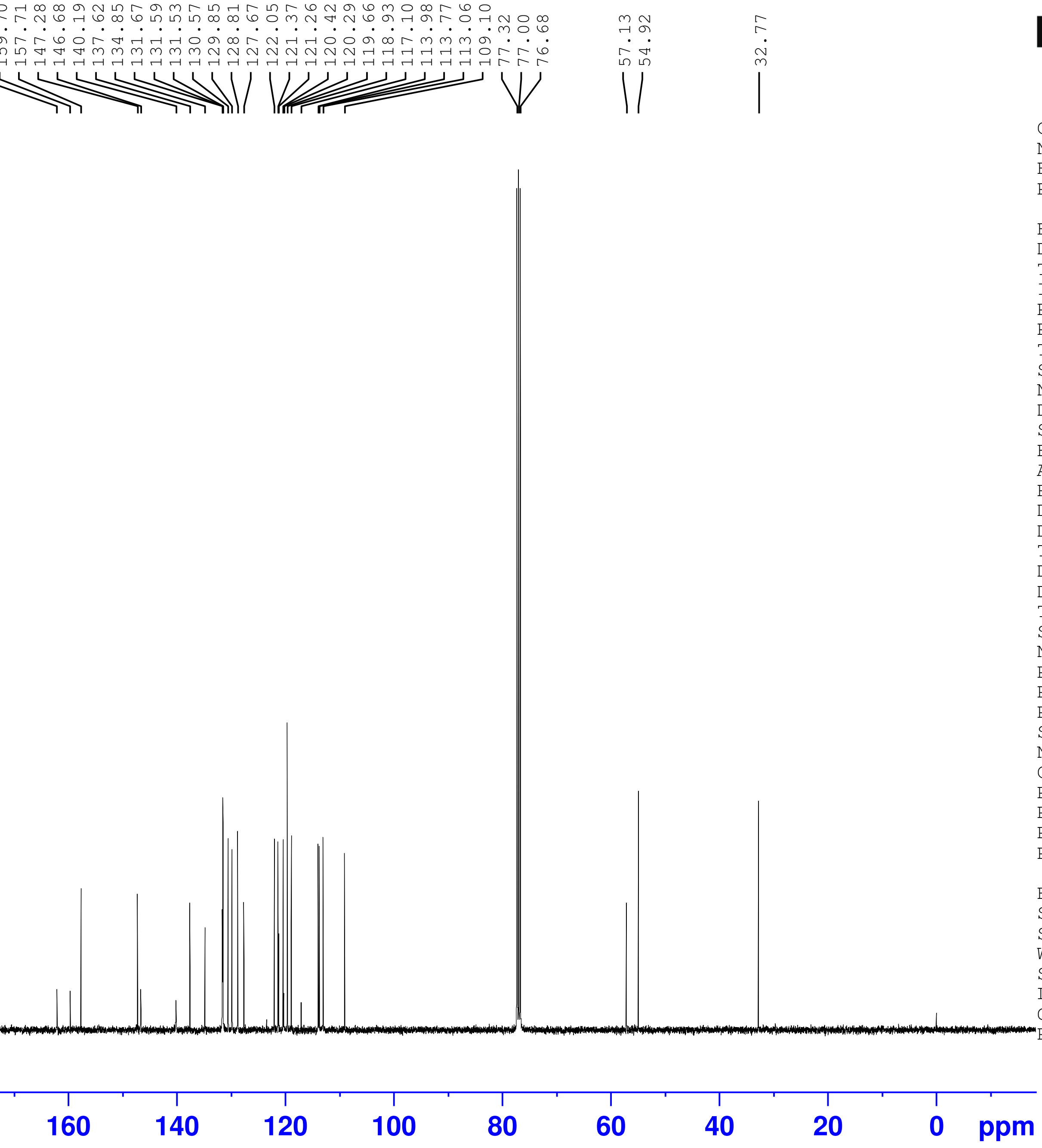

BRUKER

Current Data Parameters
NAME 3ka'-ZY-5-41OTf
EXPNO 7
PROCNO 1

F2 - Acquisition Parameters
Date_ 20220608
Time 2.51 h
INSTRUM Avance
PROBHD Z116098_0833 (
PULPROG zgpg30
TD 65536
SOLVENT CDCl3
NS 1024
DS 4
SWH 23809.523 Hz
FIDRES 0.726609 Hz
AQ 1.3762560 sec
RG 52.9819
DW 21.000 usec
DE 6.50 usec
TE 292.7 K
D1 2.00000000 sec
D11 0.03000000 sec
TD0 1
SFO1 100.6228298 MHz
NUC1 13C
P0 3.33 usec
P1 10.00 usec
PLW1 87.89900208 W
SFO2 400.1316005 MHz
NUC2 1H
CPDPRG[2 waltz65
PCPD2 90.00 usec
PLW2 20.73200035 W
PLW12 0.25595000 W
PLW13 0.12874000 W

F2 - Processing parameters
SI 32768
SF 100.6127748 MHz
WDW EM
SSB 0
LB 1.00 Hz
GB 0
PC 1.40

3ka'

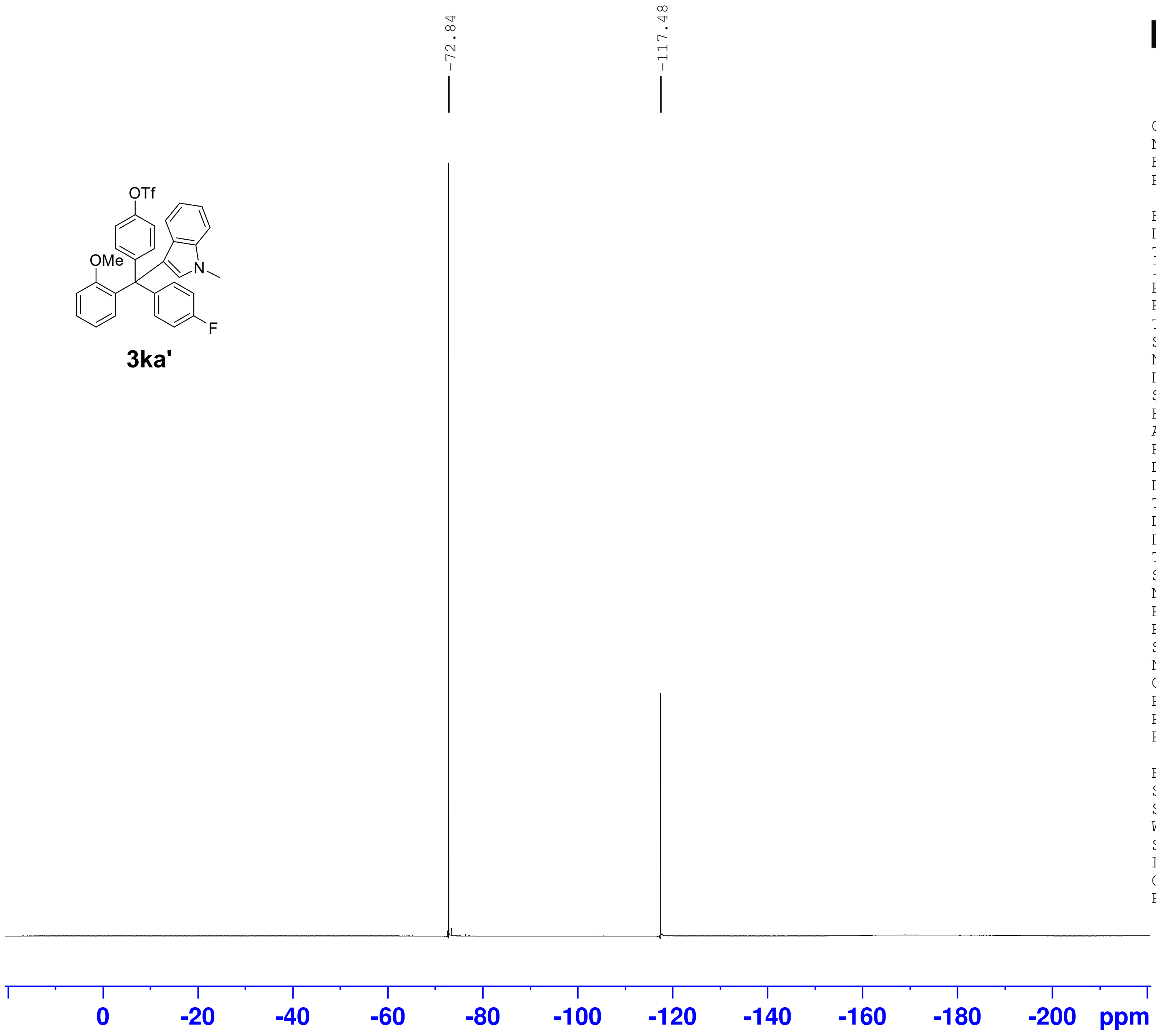

Current Data Parameters
NAME ZY-5-41-f-fr
EXPNO 8
PROCNO 1

F2 - Acquisition Parameters
Date_ 20220608
Time 2.53 h
INSTRUM Avance
PROBHD Z116098_0833 (
PULPROG zgig
TD 131072
SOLVENT CDCl3
NS 16
DS 4
SWH 90909.094 Hz
FIDRES 1.387163 Hz
AQ 0.7208960 sec
RG 101
DW 5.500 usec
DE 6.50 usec
TE 292.3 K
D1 1.00000000 sec
D11 0.03000000 sec
TD0 1
SFO1 376.4607164 MHz
NUC1 19F
P1 18.00 usec
PLW1 16.73100090 W
SFO2 400.1316005 MHz
NUC2 1H
CPDPRG[2 waltz16
PCPD2 90.00 usec
PLW2 20.73200035 W
PLW12 0.25595000 W

F2 - Processing parameters
SI 65536
SF 376.4983662 MHz
WDW EM
SSB 0
LB 0.30 Hz
GB 0
PC 1.00

3ka'-1

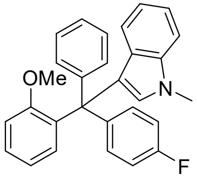

3ka'-1

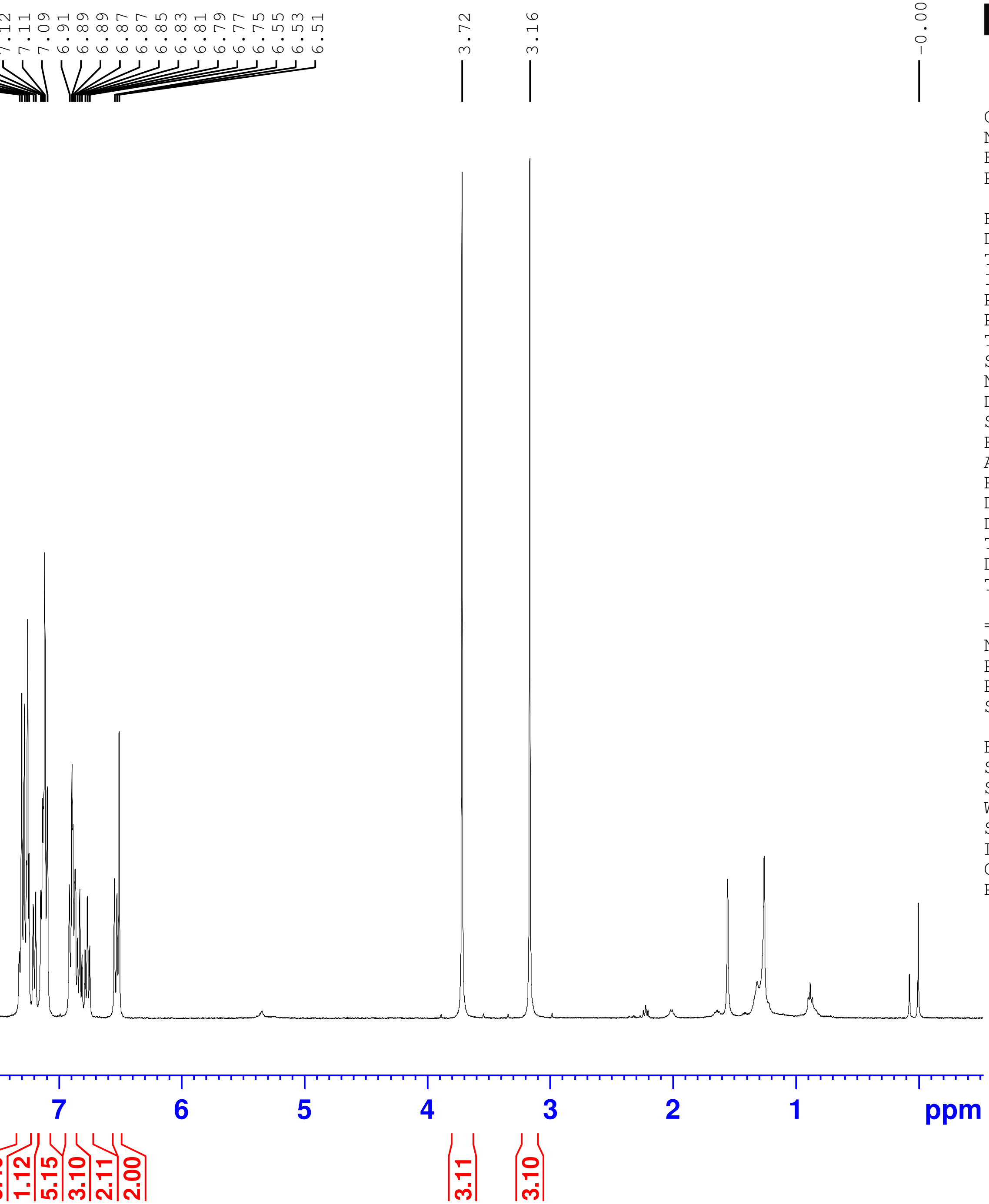

Current Data Parameters
NAME ZY-5-46-h-fr
EXPNO 416
PROCNO 1

F2 - Acquisition Parameters
Date_ 20220113
Time 21.10
INSTRUM spect
PROBHD 5 mm PABBO BB-
PULPROG zg30
TD 65536
SOLVENT CDCl3
NS 6
DS 2
SWH 8223.685 Hz
FIDRES 0.125483 Hz
AQ 3.9845889 sec
RG 181.41
DW 60.800 usec
DE 6.50 usec
TE 292.6 K
D1 1.00000000 sec
TD0 1

======== CHANNEL f1 ========
NUC1 1H
P1 14.40 usec
PLW1 14.00000000 W
SFO1 400.1924713 MHz

F2 - Processing parameters
SI 65536
SF 400.1900180 MHz
WDW EM
SSB 0
LB 0.30 Hz
GB 0
PC 1.00

3ka'-1

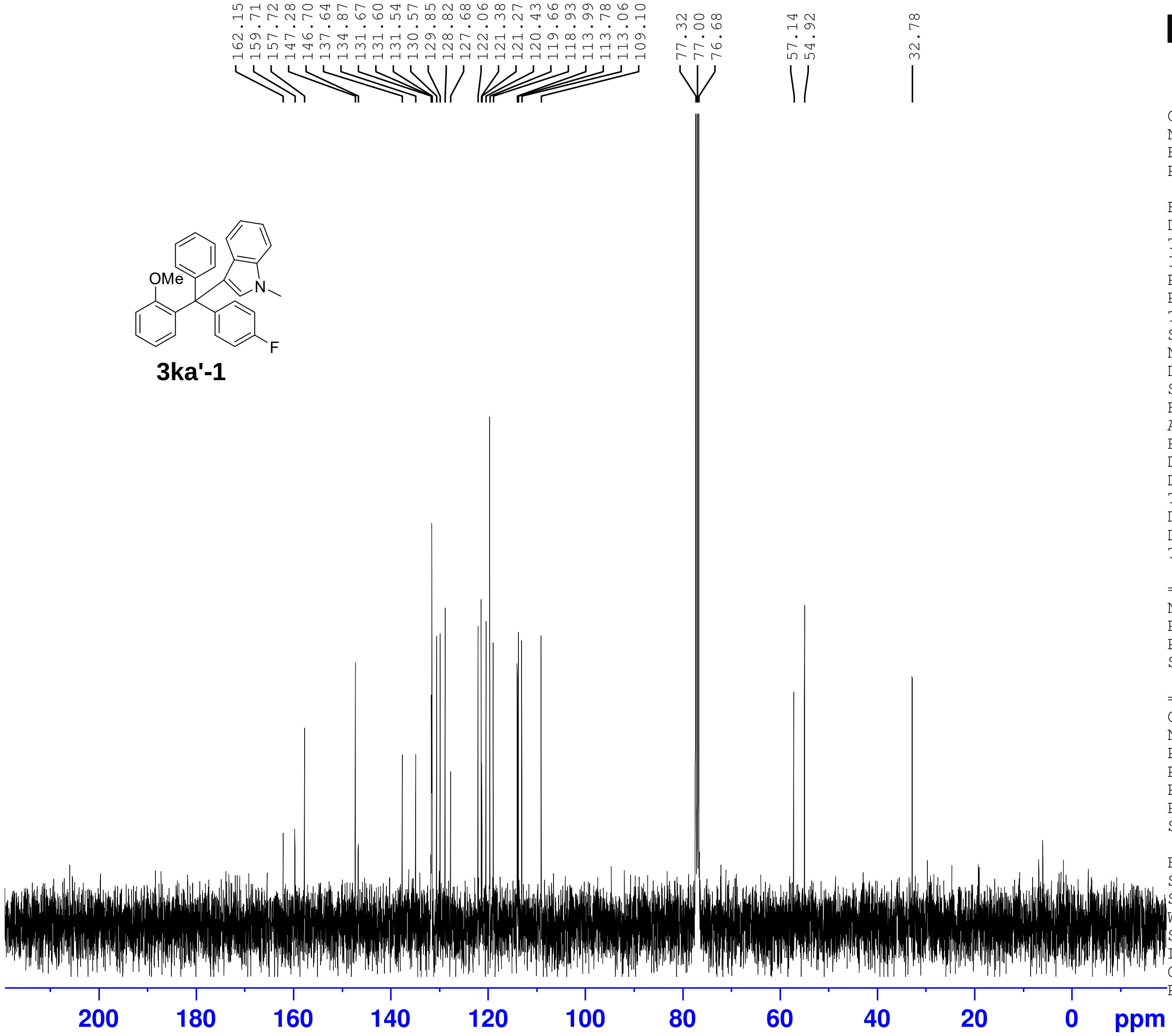

BRUKER

Current Data Parameters
NAME 3ka'-1-ZY-5-46-OTf
EXPNO 417
PROCNO 1

F2 - Acquisition Parameters
Date_ 20220113
Time 21.12
INSTRUM spect
PROBHD 5 mm PABBO BB-
PULPROG zgpg30
TD 65536
SOLVENT CDCl3
NS 190
DS 4
SWH 24038.461 Hz
FIDRES 0.366798 Hz
AQ 1.3631488 sec
RG 193.13
DW 20.800 usec
DE 6.50 usec
TE 292.9 K
D1 2.00000000 sec
D11 0.03000000 sec
TD0 1

======== CHANNEL f1 ========
NUC1 13C
P1 9.90 usec
PLW1 53.00000000 W
SFO1 100.6379178 MHz

======== CHANNEL f2 ========
CPDPRG[2 waltz16
NUC2 1H
PCPD2 90.00 usec
PLW2 14.00000000 W
PLW12 0.35839999 W
PLW13 0.29030001 W
SFO2 400.1916008 MHz

F2 - Processing parameters
SI 32768
SF 100.6278614 MHz
WDW EM
SSB 0
LB 1.00 Hz
GB 0
PC 1.40

3ka'-1

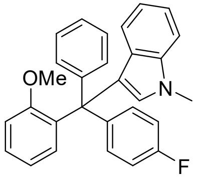

3ka'-1

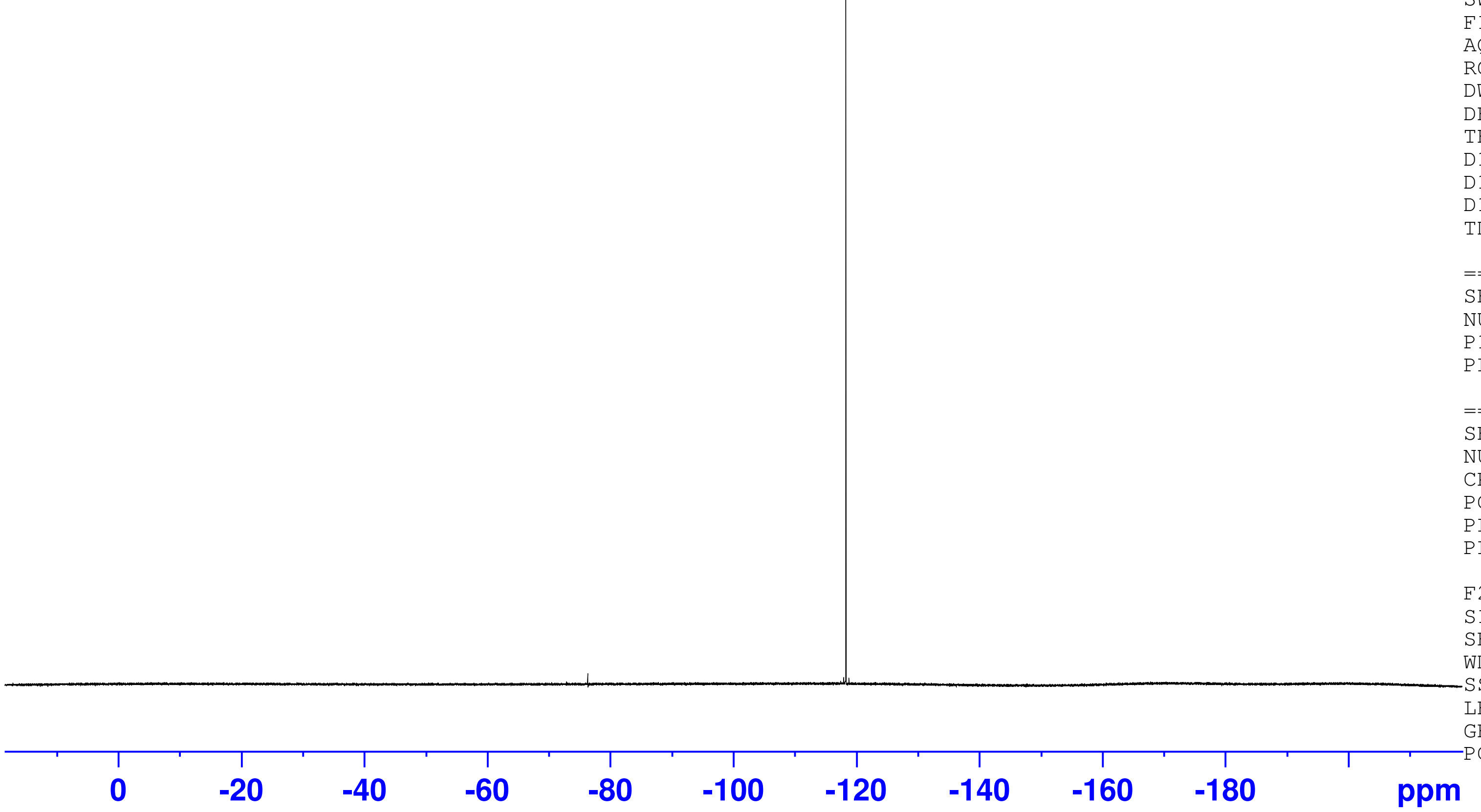

Current Data Parameters
NAME 211129sjw
EXPNO 5668
PROCNO 1

F2 - Acquisition Parameters
Date_ 20211129
Time 8.58
INSTRUM spect
PROBHD 5 mm PABBO BB-
PULPROG zgfhigqn.2
TD 131072
SOLVENT CDCl3
NS 16
DS 4
SWH 66964.289 Hz
FIDRES 0.510897 Hz
AQ 0.9786710 sec
RG 203
DW 7.467 usec
DE 6.50 usec
TE -59.1 K
D1 1.00000000 sec
D11 0.03000000 sec
D12 0.00002000 sec
TD0 1

======== CHANNEL f1 ========
SFO1 282.3761148 MHz
NUC1 19F
P1 14.50 usec
PLW1 10.39999962 W

======== CHANNEL f2 ========
SFO2 300.1312005 MHz
NUC2 1H
CPDPRG[2 waltz16
PCPD2 90.00 usec
PLW2 14.00000000 W
PLW12 0.17284000 W

F2 - Processing parameters
SI 65536
SF 282.4043552 MHz
WDW EM
SSB 0
LB 0.30 Hz
GB 0
PC 1.00

3ka'-2

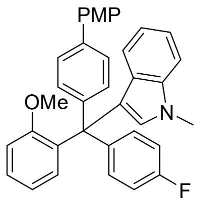

3ka'-2

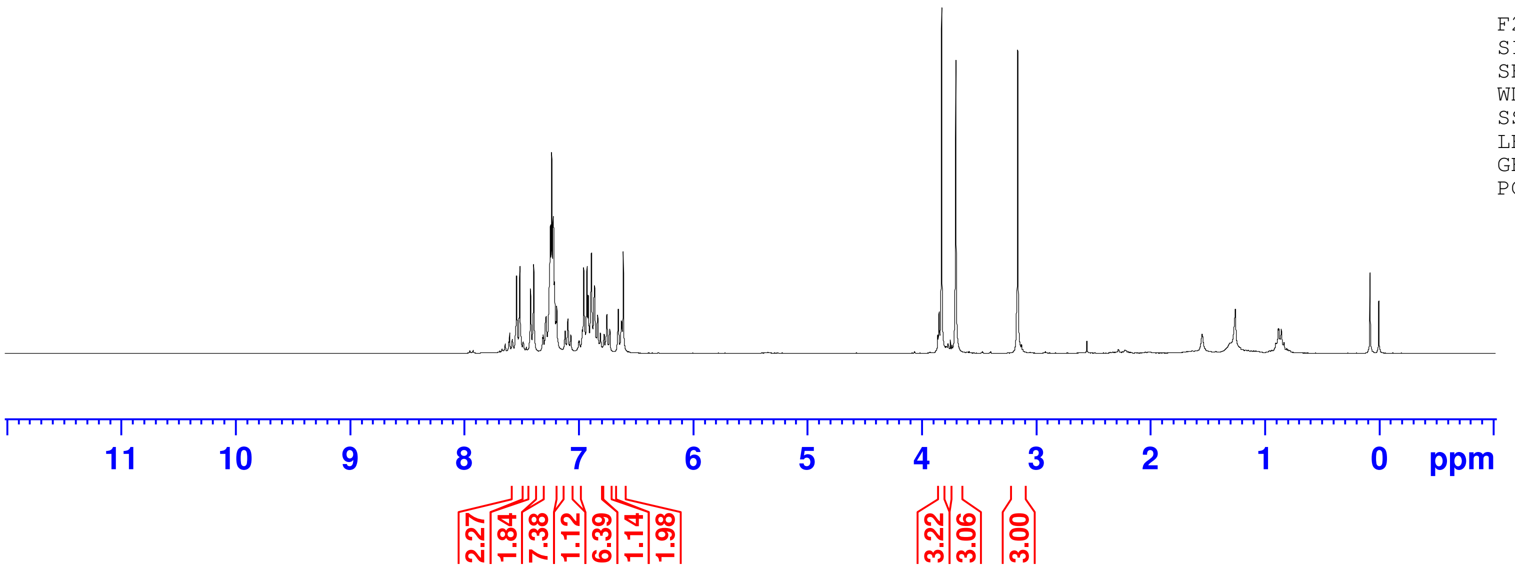

Current Data Parameters

NAME	ZY-5-42-h-fr
EXPNO	5690
PROCNO	1

F2 - Acquisition Parameters

Date_	20211207
Time	9.05
INSTRUM	spect
PROBHD	5 mm PABBO BB-
PULPROG	zg30
TD	65536
SOLVENT	CDCl3
NS	16
DS	2
SWH	6009.615 Hz
FIDRES	0.091699 Hz
AQ	5.4525952 sec
RG	128
DW	83.200 usec
DE	6.50 usec
TE	-59.1 K
D1	1.00000000 sec
TD0	1

======== CHANNEL f1 ========

SFO1	300.1318534 MHz
NUC1	1H
P1	10.00 usec
PLW1	14.00000000 W

F2 - Processing parameters

SI	65536
SF	300.1300145 MHz
WDW	EM
SSB	0
LB	0.30 Hz
GB	0
PC	1.00

3ka'-2

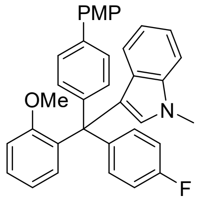

3ka'-2

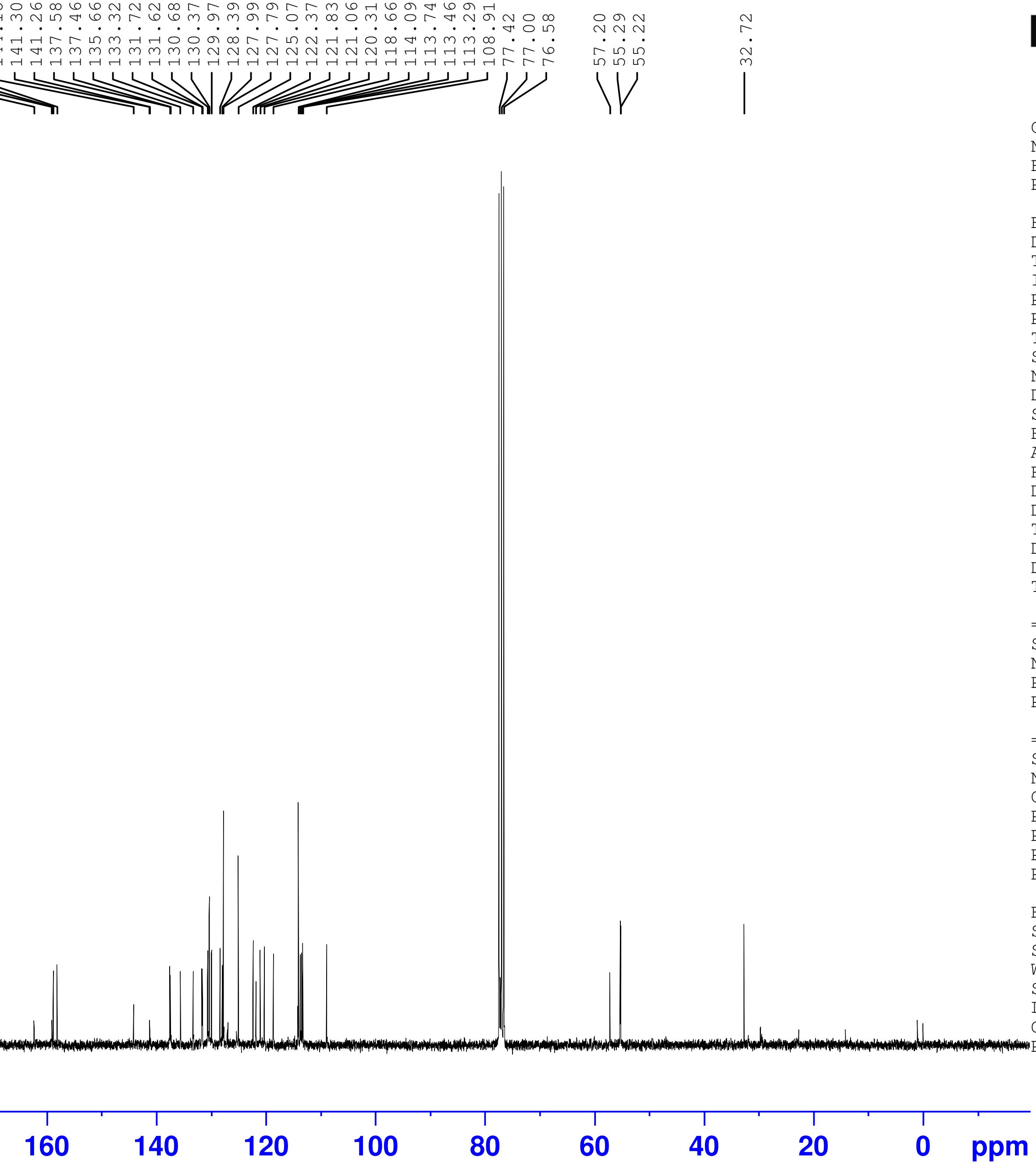

Current Data Parameters
NAME 3ka'-2-ZY-5-42-Kumada
EXPNO 5691
PROCNO 1

F2 - Acquisition Parameters
Date_ 20211207
Time 10.15
INSTRUM spect
PROBHD 5 mm PABBO BB-
PULPROG zgpg30
TD 65536
SOLVENT CDCl3
NS 1024
DS 4
SWH 18028.846 Hz
FIDRES 0.275098 Hz
AQ 1.8175317 sec
RG 203
DW 27.733 usec
DE 6.50 usec
TE -59.1 K
D1 2.00000000 sec
D11 0.03000000 sec
TD0 1

======== CHANNEL f1 ========
SFO1 75.4752949 MHz
NUC1 13C
P1 9.50 usec
PLW1 34.20000076 W

======== CHANNEL f2 ========
SFO2 300.1312005 MHz
NUC2 1H
CPDPRG[2 waltz16
PCPD2 90.00 usec
PLW2 14.00000000 W
PLW12 0.17284000 W
PLW13 0.14000000 W

F2 - Processing parameters
SI 32768
SF 75.4677538 MHz
WDW EM
SSB 0
LB 1.00 Hz
GB 0
PC 1.40

3ka'-2

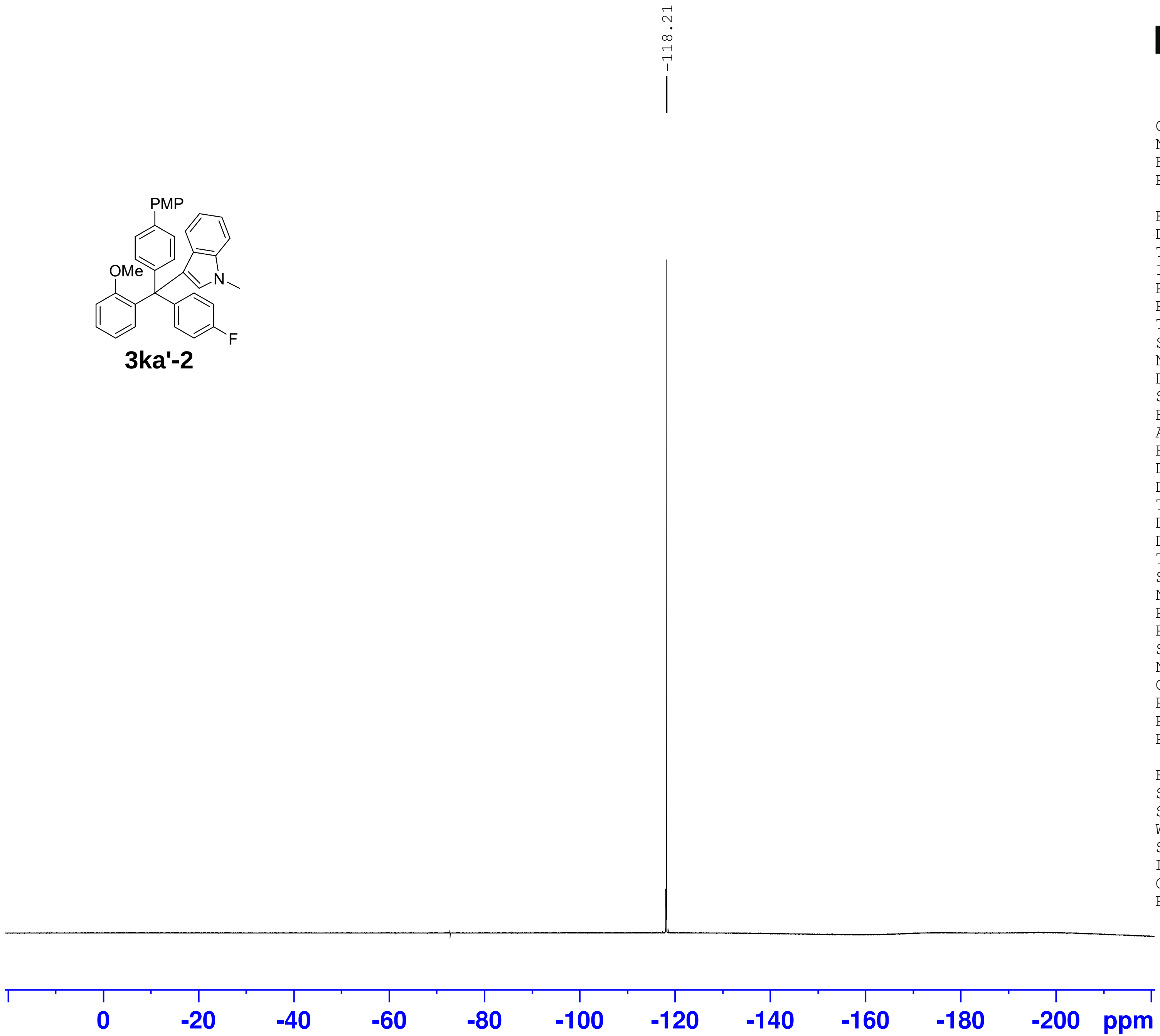


```
Current Data Parameters
NAME              0607HH
EXPNO                  1
PROCNO                 1

F2 - Acquisition Parameters
Date_           20220607
Time               21.19 h
INSTRUM           Avance
PROBHD   Z116098_0833 (
PULPROG             zgig
TD                131072
SOLVENT            CDCl3
NS                    16
DS                     4
SWH           90909.094 Hz
FIDRES         1.387163 Hz
AQ            0.7208960 sec
RG                   101
DW                 5.500 usec
DE                  6.50 usec
TE                 291.8 K
D1            1.00000000 sec
D11           0.03000000 sec
TD0                    1
SFO1         376.4607164 MHz
NUC1                 19F
P1                 18.00 usec
PLW1         16.73100090 W
SFO2         400.1316005 MHz
NUC2                  1H
CPDPRG[2         waltz16
PCPD2              90.00 usec
PLW2         20.73200035 W
PLW12         0.25595000 W

F2 - Processing parameters
SI                 65536
SF           376.4983662 MHz
WDW                   EM
SSB                    0
LB                  0.30 Hz
GB                     0
PC                  1.00
```

Sample Name: ZY-4-1-OP

HPLC Condition: IC, *n*-Hexane/*i*PrOH = 98:2, 0.4 mL/min

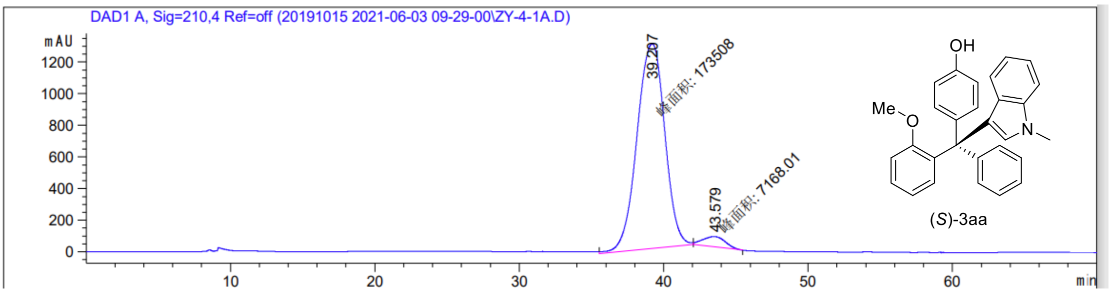

Peak	RetTime[min]	Type	Width[min]	Area[mAu*s]	Height[mAU]	Area[%]
1	39.207	MM	2.2311	1.73508e5	1296.14172	96.0327
2	43.579	MM	1.8022	7168.00879	66.29072	3.9673

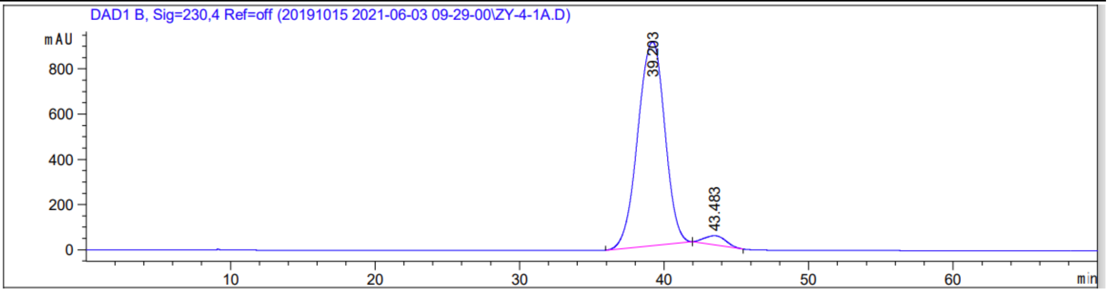

Peak	RetTime[min]	Type	Width[min]	Area[mAu*s]	Height[mAU]	Area[%]
1	39.203	BB	2.0480	1.16817e5	901.35608	96.5511
2	43.483	BB	1.3338	4172.75342	40.57460	3.4489

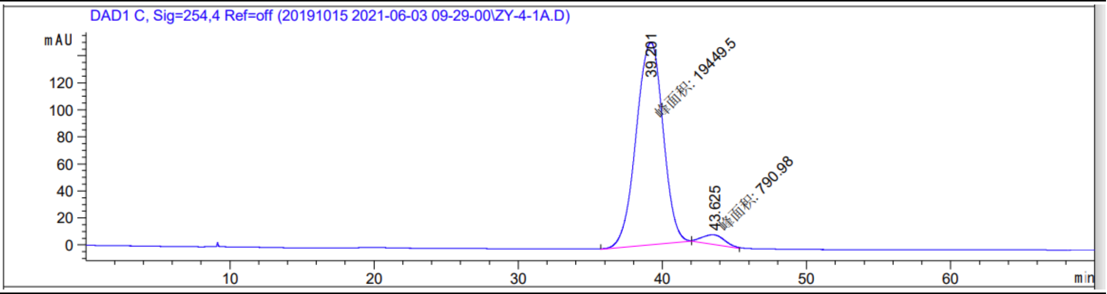

Peak	RetTime[min]	Type	Width[min]	Area[mAu*s]	Height[mAU]	Area[%]
1	39.201	MM	2.1637	1.94495e4	149.81966	96.0921
2	43.625	MM	1.8086	790.98035	7.28912	3.9079

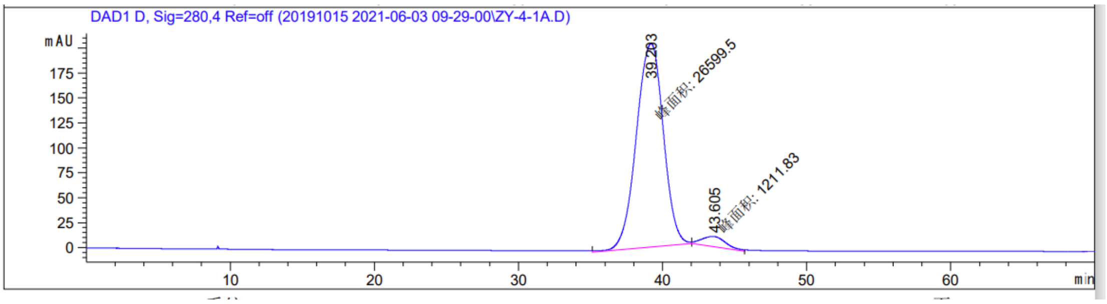

Peak	RetTime[min]	Type	Width[min]	Area[mAu*s]	Height[mAU]	Area[%]
1	39.203	MM	2.1737	2.65995e4	203.94975	95.6427
2	43.605	MM	1.9418	1211.82544	10.40147	4.3573

==

End of Report

Sample Name: ZY-4-1-Rac

HPLC Condition: IC, *n*-Hexane/*i*PrOH = 98:2, 0.4 mL/min

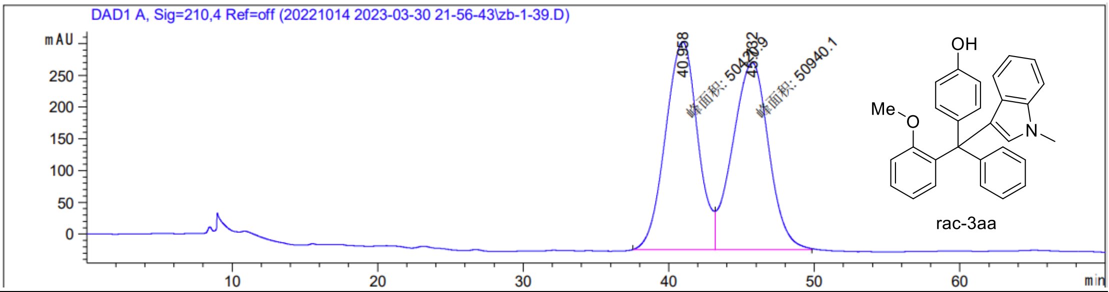

Peak	RetTime[min]	Type	Width[min]	Area[mAu*s]	Height[mAU]	Area[%]
1	40.958	MF	2.5684	5.04209e4	327.18265	49.7439
2	45.732	FM	2.8834	5.09401e4	294.44788	50.2561

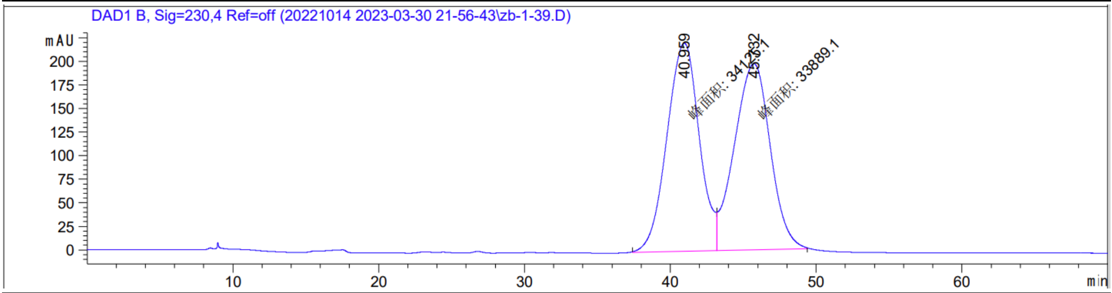

Peak	RetTime[min]	Type	Width[min]	Area[mAu*s]	Height[mAU]	Area[%]
1	40.959	MF	2.5744	3.41251e4	220.92221	50.1735
2	45.732	FM	2.8585	3.38891e4	197.59492	49.8265

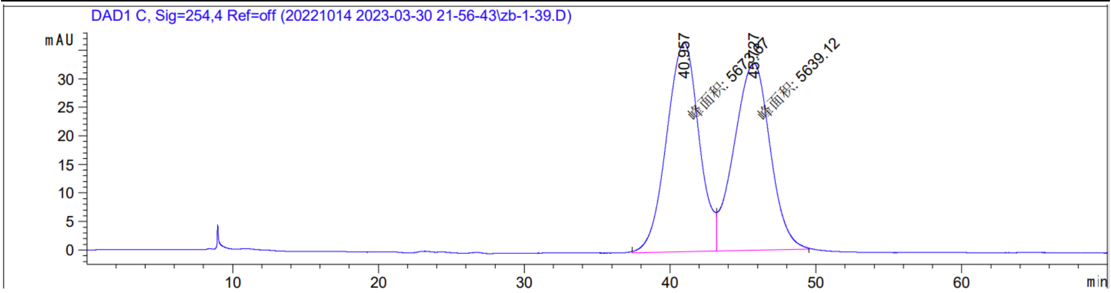

Peak	RetTime[min]	Type	Width[min]	Area[mAu*s]	Height[mAU]	Area[%]
1	40.957	MF	2.5842	5673.66846	36.59201	50.1527
2	45.727	FM	2.8712	5639.12256	32.73415	49.8473

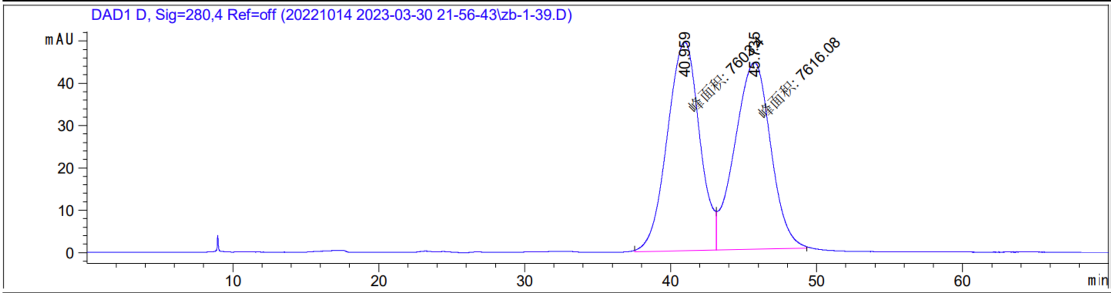

Peak	RetTime[min]	Type	Width[min]	Area[mAu*s]	Height[mAU]	Area[%]
1	40.959	MF	2.5662	7603.39795	49.38079	49.9583
2	45.735	FM	2.8743	7616.07617	44.16167	50.0417

==

End of Report

Sample Name: ZY-4-64B-OP

HPLC Condition: IC, *n*-Hexane/*i*PrOH = 98:2 0.4 mL/min

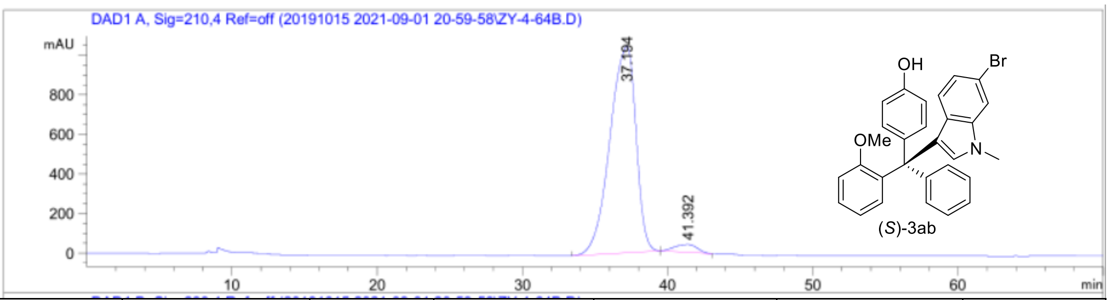

Peak	RetTime[min]	Type	Width[min]	Area[mAu*s]	Height[mAU]	Area[%]
1	37.194	BB	1.7533	1.30019e5	1042.30164	96.8915
2	41.392	BB	1.2611	4171.35645	39.02382	3.1085

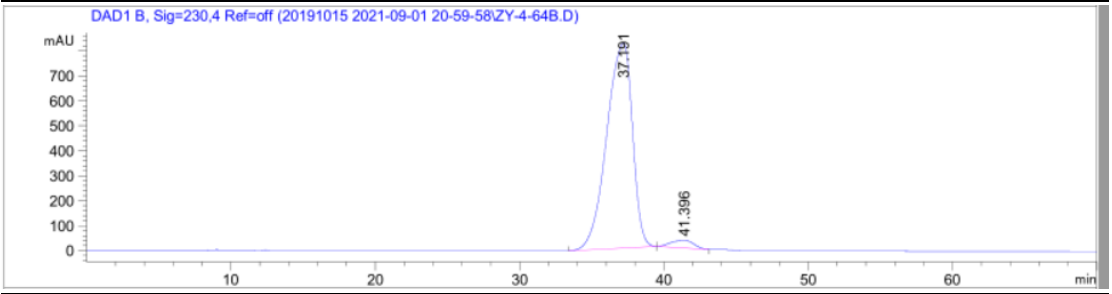

Peak	RetTime[min]	Type	Width[min]	Area[mAu*s]	Height[mAU]	Area[%]
1	37.191	BB	1.9635	1.01733e5	821.08508	96.8864
2	41.396	BB	1.3226	3269.40015	30.54935	3.1136

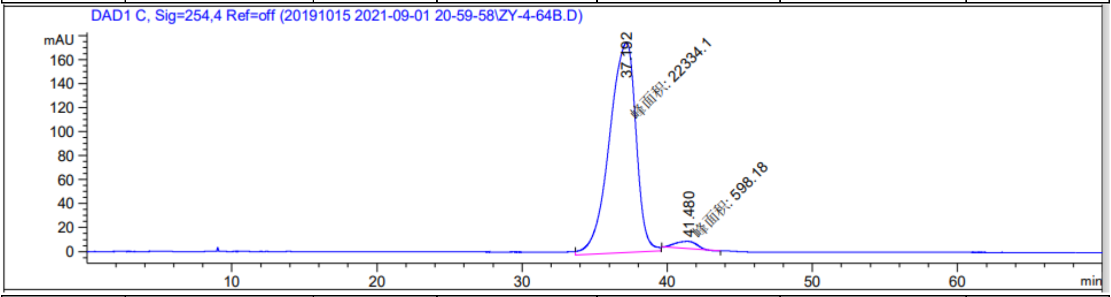

Peak	RetTime[min]	Type	Width[min]	Area[mAu*s]	Height[mAU]	Area[%]
1	37.192	MM	2.1263	2.23341e4	175.05919	97.3915
2	41.480	MM	1.6590	598.17963	6.00946	2.6085

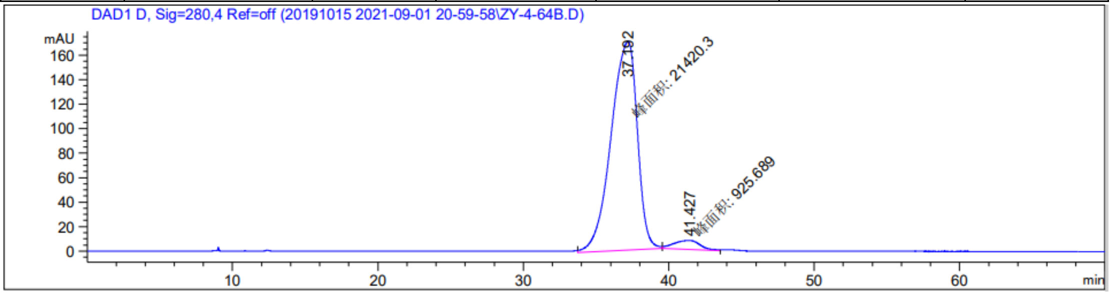

Peak	RetTime[min]	Type	Width[min]	Area[mAu*s]	Height[mAU]	Area[%]
1	37.192	MM	2.0948	2.14203e4	170.42166	95.8575
2	41.427	MM	2.0975	925.68915	7.35564	4.1425

==

End of Report

Sample Name: ZY-4-58B-Rac

HPLC Condition: IC, *n*-Hexane/*i*PrOH = 98:2, 0.4 mL/min

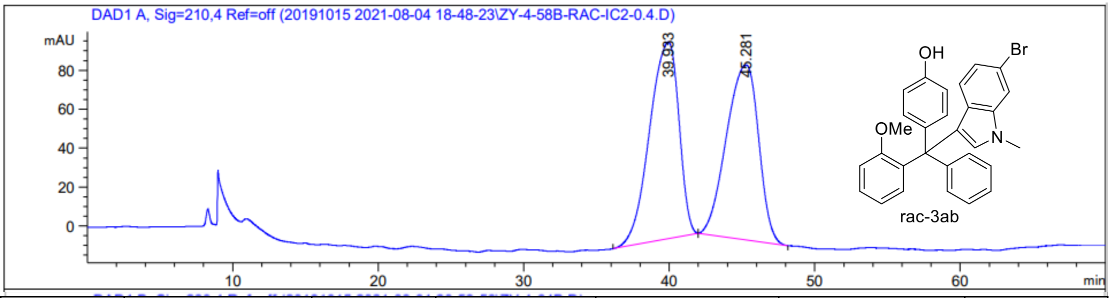

Peak	RetTime[min]	Type	Width[min]	Area[mAu*s]	Height[mAU]	Area[%]
1	39.933	BB	1.8766	1.41397e4	101.04619	50.6053
2	45.281	BB	1.8234	1.38015e4	89.98221	49.3947

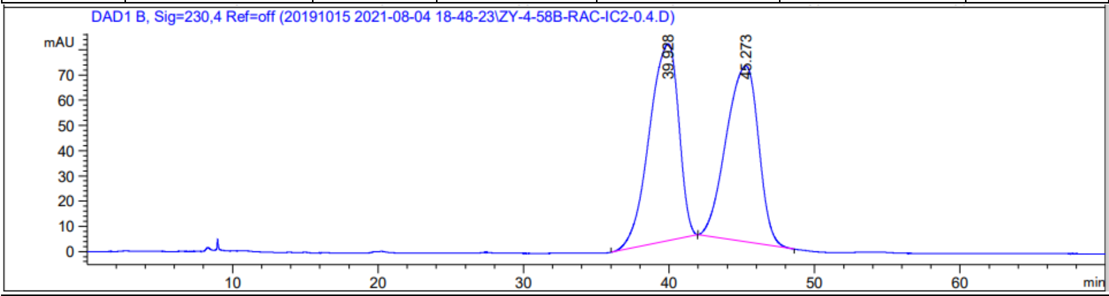

Peak	RetTime[min]	Type	Width[min]	Area[mAu*s]	Height[mAU]	Area[%]
1	39.928	BB	1.6893	1.09764e4	78.02338	50.5438
2	45.273	BB	2.0151	1.07403e4	69.72675	49.4562

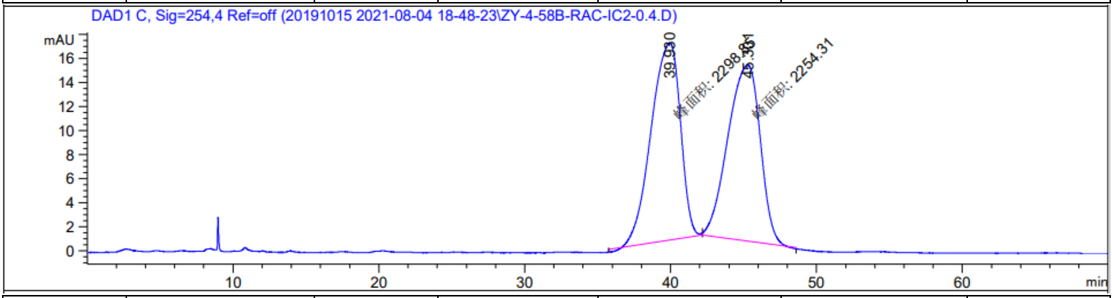

Peak	RetTime[min]	Type	Width[min]	Area[mAu*s]	Height[mAU]	Area[%]
1	39.930	MM	2.3328	2298.85034	16.42433	50.4891
2	45.301	MM	2.5637	2254.31396	14.65508	49.5109

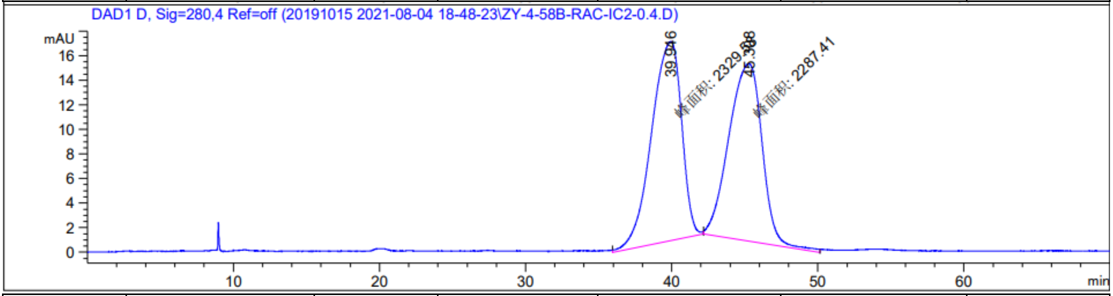

Peak	RetTime[min]	Type	Width[min]	Area[mAu*s]	Height[mAU]	Area[%]
1	39.946	MM	2.3935	2329.57959	16.22133	50.4567
2	45.308	MM	2.6355	2287.40967	14.46512	49.5433

==

End of Report

Sample Name: ZY-4-64G-OP

HPLC Condition: IC, *n*-Hexane/*i*PrOH = 95:5, 1.0 mL/min

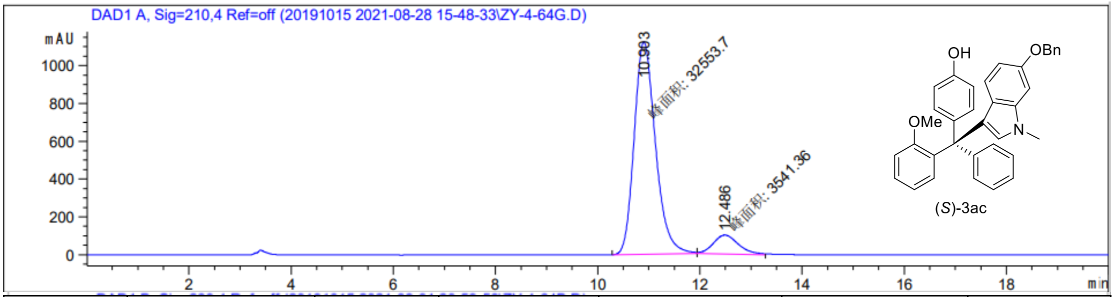

Peak	RetTime[min]	Type	Width[min]	Area[mAu*s]	Height[mAU]	Area[%]
1	10.903	MM	0.4831	3.25537e4	1123.04224	90.1888
2	12.486	MM	0.5844	3541.36279	101.00571	9.8112

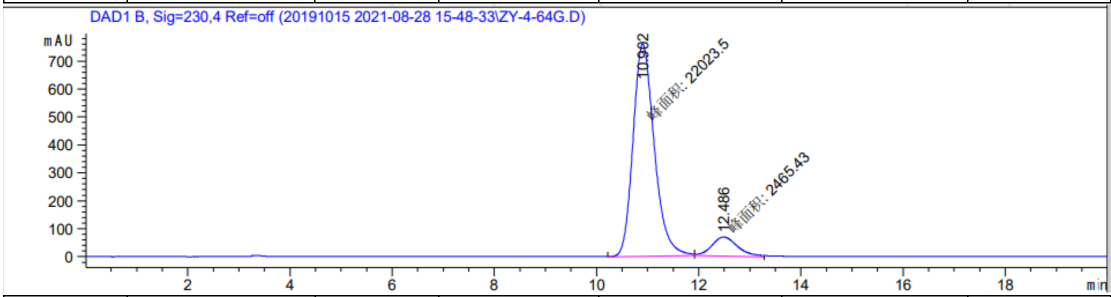

Peak	RetTime[min]	Type	Width[min]	Area[mAu*s]	Height[mAU]	Area[%]
1	10.902	MM	0.4824	2.20235e4	760.86499	89.9324
2	12.486	MM	0.5974	2465.43408	68.78637	10.0676

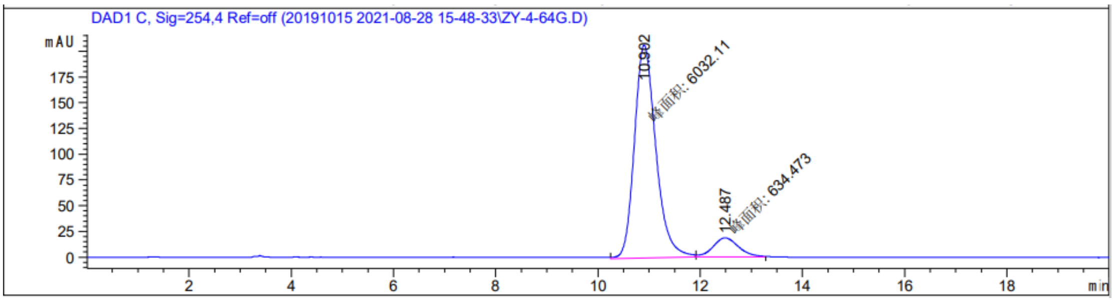

Peak	RetTime[min]	Type	Width[min]	Area[mAu*s]	Height[mAU]	Area[%]
1	10.902	MM	0.4859	6032.11475	206.91098	90.4828
2	12.487	MM	0.5766	634.47290	18.34086	9.5172

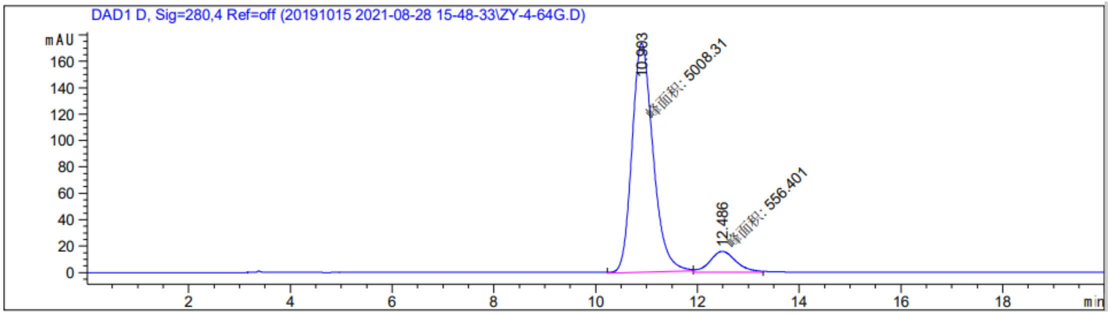

Peak	RetTime[min]	Type	Width[min]	Area[mAu*s]	Height[mAU]	Area[%]
1	10.903	MM	0.4812	5008.30859	173.46349	90.0013
2	12.486	MM	0.5916	556.40137	15.67381	9.9987

==

End of Report

Sample Name: ZY-4-64G-Rac

HPLC Condition: IC, *n*-Hexane/*i*PrOH = 95:5, 1.0 mL/min

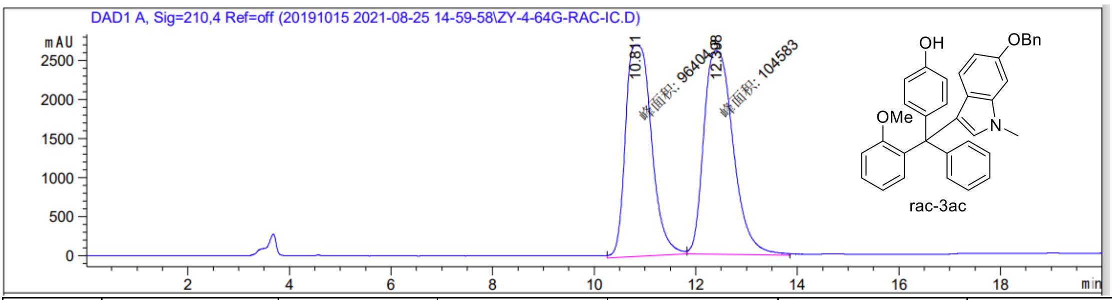

Peak	RetTime[min]	Type	Width[min]	Area[mAu*s]	Height[mAU]	Area[%]
1	10.811	MM	0.5927	9.64043e4	2710.94531	47.9654
2	12.398	MM	0.6697	1.04583e5	2602.57324	52.0346

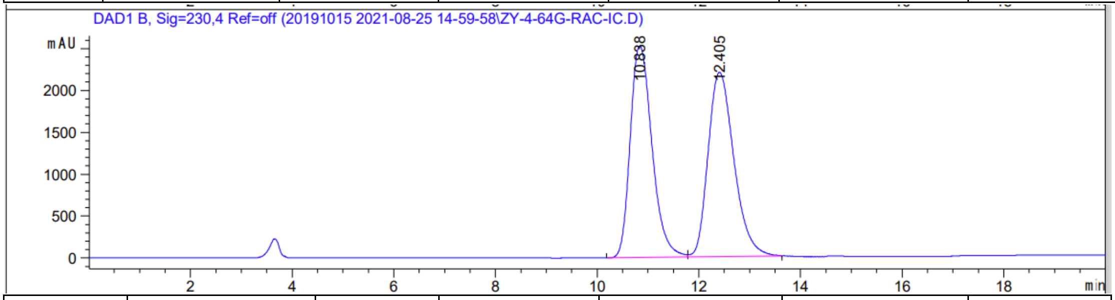

Peak	RetTime[min]	Type	Width[min]	Area[mAu*s]	Height[mAU]	Area[%]
1	10.838	BV	0.4625	7.54036e4	2524.41772	49.2230
2	12.405	VV R	0.5460	7.77843e4	2201.99829	50.7770

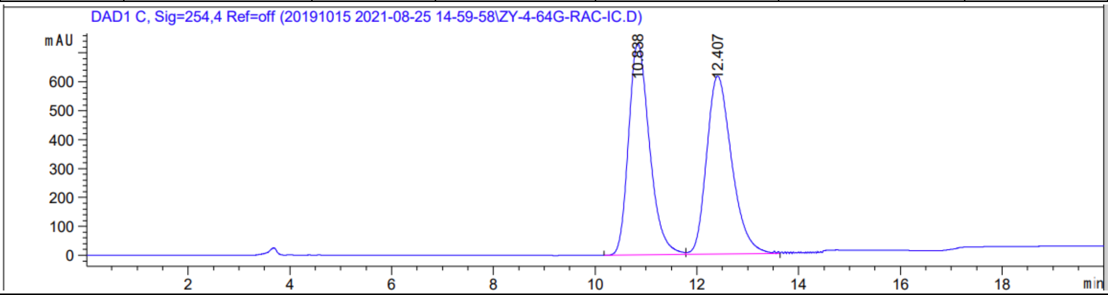

Peak	RetTime[min]	Type	Width[min]	Area[mAu*s]	Height[mAU]	Area[%]
1	10.838	BV	0.4409	2.09894e4	731.08252	49.8442 2
2	12.407	VV R	0.5256	2.11206e4	615.84363	50.1558

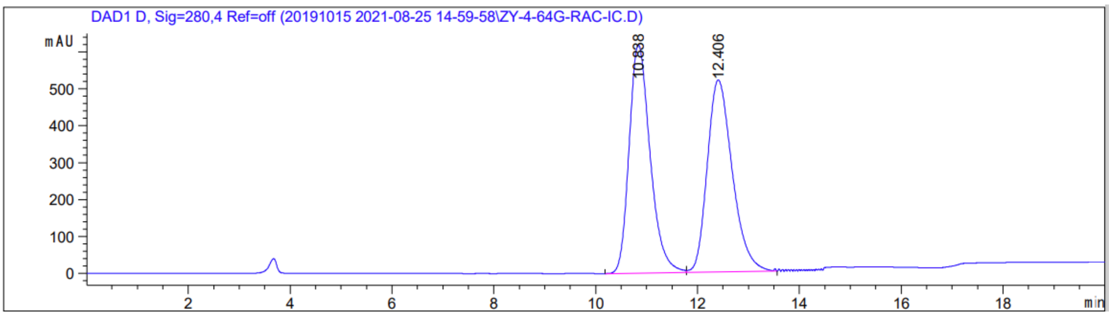

Peak	RetTime[min]	Type	Width[min]	Area[mAu*s]	Height[mAU]	Area[%]
1	10.838	BV	0.4400	1.77138e4	618.73187	49.8822
2	12.406	VV R	0.5246	1.77975e4	520.47125	50.1178

==

End of Report

Sample Name: ZY-4-64C-OP

HPLC Condition: AD-H, *n*-Hexane/*i*PrOH = 95:5, 1.0 mL/min

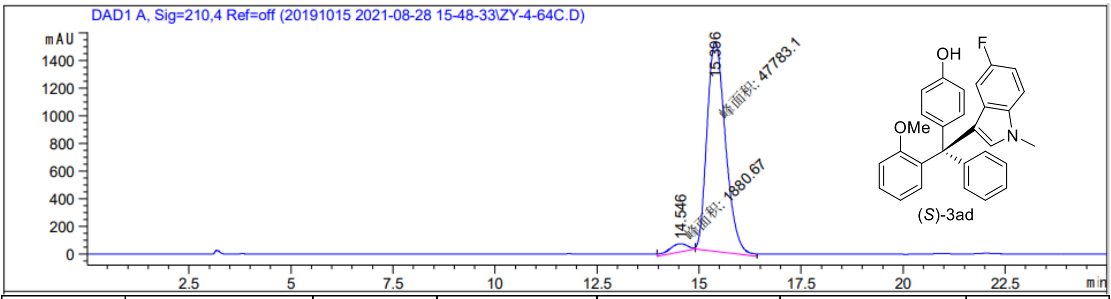

Peak	RetTime[min]	Type	Width[min]	Area[mAu*s]	Height[mAU]	Area[%]
1	14.546	MM	0.5313	1880.67407	59.00087	3.7868
2	15.396	MM	0.5268	4.77831e4	1511.83679	96.2132

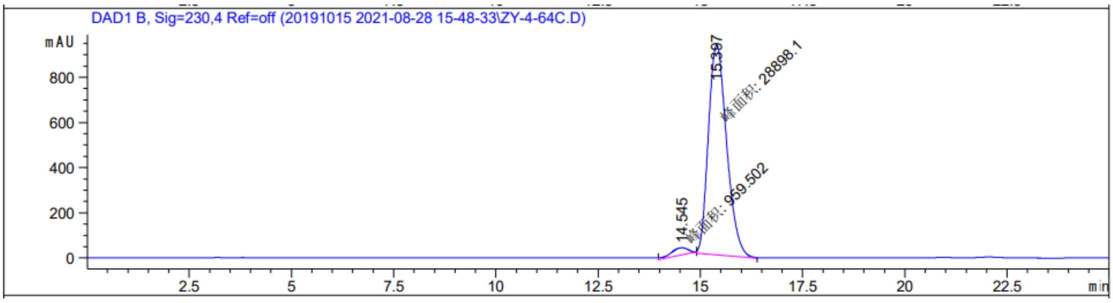

Peak	RetTime[min]	Type	Width[min]	Area[mAu*s]	Height[mAU]	Area[%]
1	14.545	MM	0.4988	959.50208	32.05903	3.2136
2	15.397	MM	0.5182	2.88981e4	929.38043	96.7864

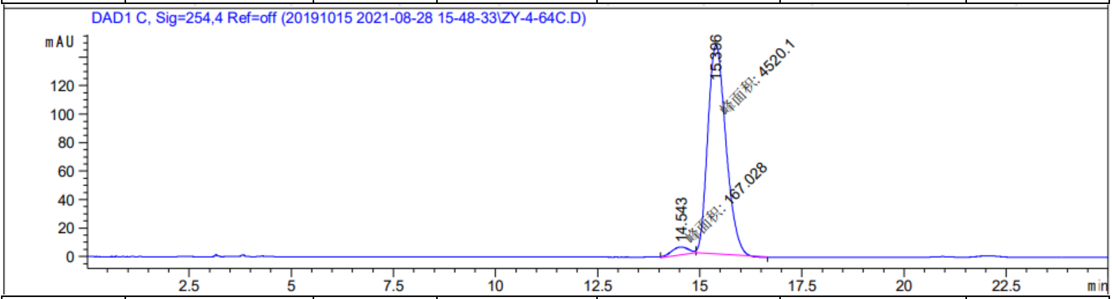

Peak	RetTime[min]	Type	Width[min]	Area[mAu*s]	Height[mAU]	Area[%]
1	14.543	MM	0.5001	167.02847	5.56616	3.5636
2	15.396	MM	0.5136	4520.09570	146.68657	96.4364

==

End of Report

Sample Name: ZY-4-58C-Rac

HPLC Condition: AD-H, *n*-Hexane/*i*PrOH = 95:5, 1.0 mL/min

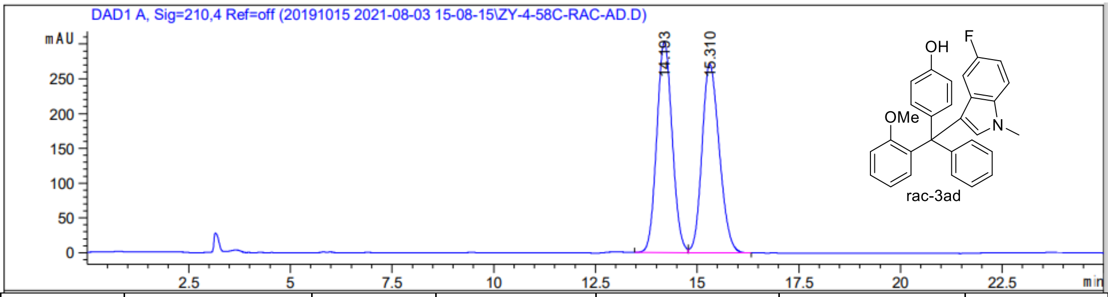

Peak	RetTime[min]	Type	Width[min]	Area[mAu*s]	Height[mAU]	Area[%]
1	14.193	BV	0.4122	8009.29004	303.13669	49.9610
2	15.310	VB	0.4608	8021.78174	271.44949	50.0390

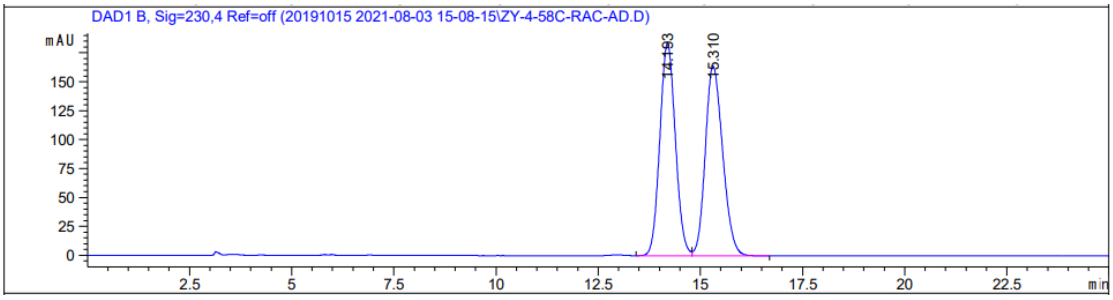

Peak	RetTime[min]	Type	Width[min]	Area[mAu*s]	Height[mAU]	Area[%]
1	14.193	BV	0.4129	4855.02344	183.37392	49.9444
2	15.310	VB	0.4620	4865.83594	164.07764	50.0556

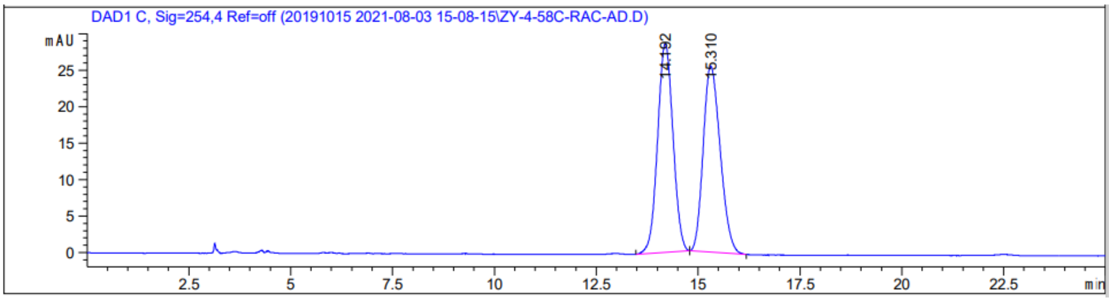

Peak	RetTime[min]	Type	Width[min]	Area[mAu*s]	Height[mAU]	Area[%]
1	14.192	BB	0.4127	751.80017	28.59524	50.3094
2	15.310	BB	0.4575	742.55371	25.52102	49.6906

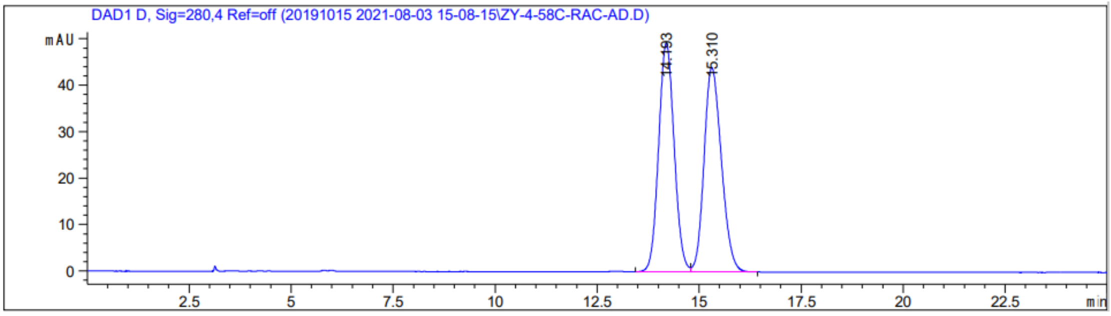

Peak	RetTime[min]	Type	Width[min]	Area[mAu*s]	Height[mAU]	Area[%]
1	14.193	BV	0.4155	1305.89575	49.22363	50.0324
2	15.310	VB	0.4617	1304.20215	44.01931	49.9676

==

End of Report

Sample Name: ZY-4-64D-OP

HPLC Condition: AD-H, *n*-Hexane/*i*PrOH = 95:5, 1.0 mL/min

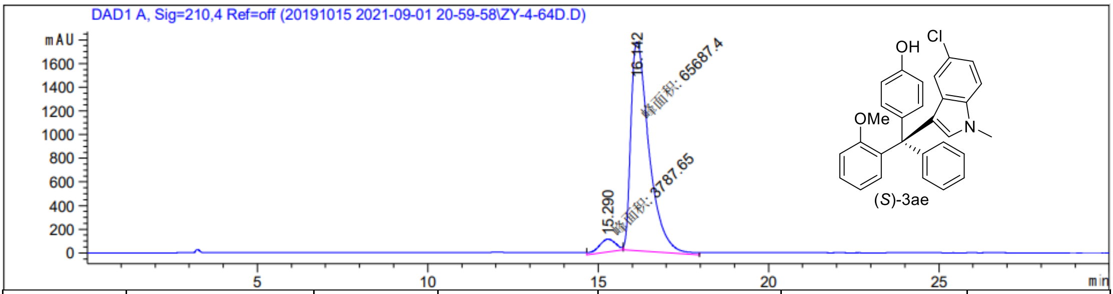

Peak	RetTime[min]	Type	Width[min]	Area[mAu*s]	Height[mAU]	Area[%]
1	15.290	MM	0.5800	3787.64600	108.83871	5.4518
2	16.142	MM	0.6204	6.56874e4	1764.64526	94.5482

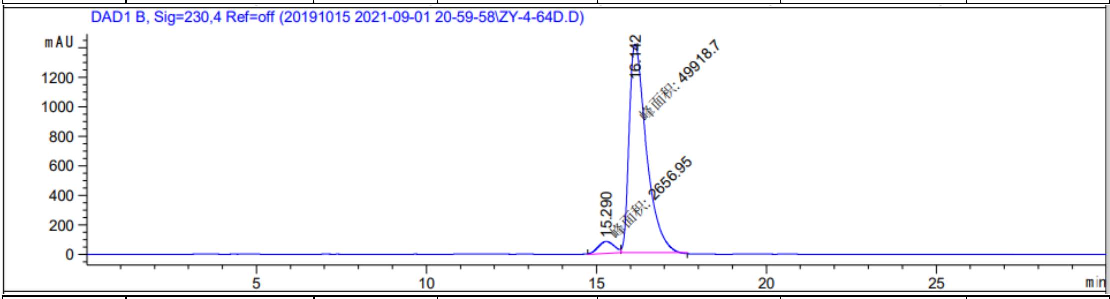

Peak	RetTime[min]	Type	Width[min]	Area[mAu*s]	Height[mAU]	Area[%]
1	15.290	MM	0.5477	2656.95215	80.85711	5.0536
2	16.142	MM	0.5891	4.99187e4	1412.32788	94.9464

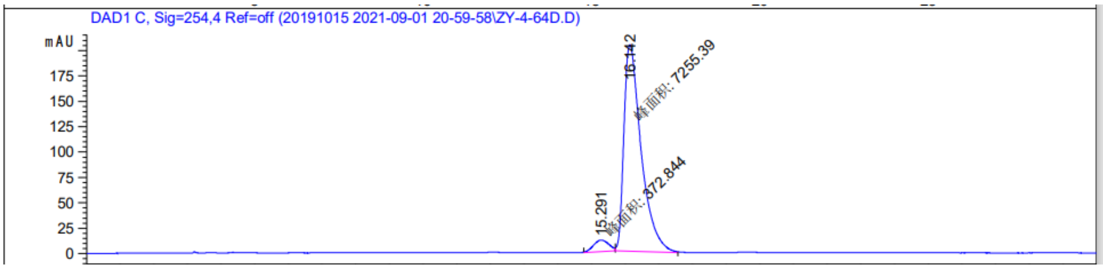

Peak	RetTime[min]	Type	Width[min]	Area[mAu*s]	Height[mAU]	Area[%]
1	15.291	MM	0.5396	372.84375	11.51635	4.8877
2	16.142	MM	0.5943	7255.38867	203.48567	95.1123

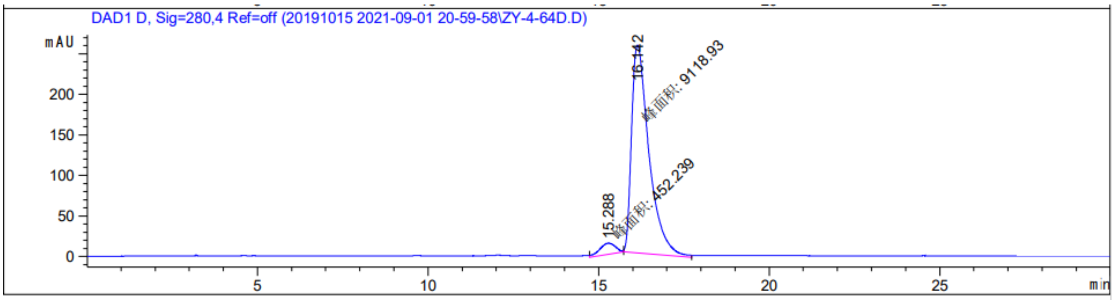

Peak	RetTime[min]	Type	Width[min]	Area[mAu*s]	Height[mAU]	Area[%]
1	15.288	MM	0.5453	452.23929	13.82346	4.7250
2	16.142	MM	0.5927	9118.93457	256.40613	95.2750

==

End of Report

Sample Name: ZY-4-64D-Rac

HPLC Condition: AD-H, *n*-Hexane/*i*PrOH = 95:5, 1.0 mL/min

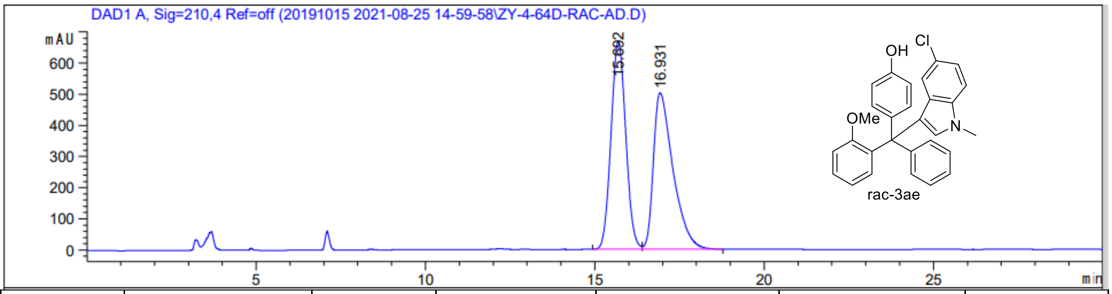

Peak	RetTime[min]	Type	Width[min]	Area[mAu*s]	Height[mAU]	Area[%]
1	15.692	BV	0.4730	2.01741e4	666.84833	49.9031
2	16.931	VB	0.6048	2.02524e4	501.90540	50.0969

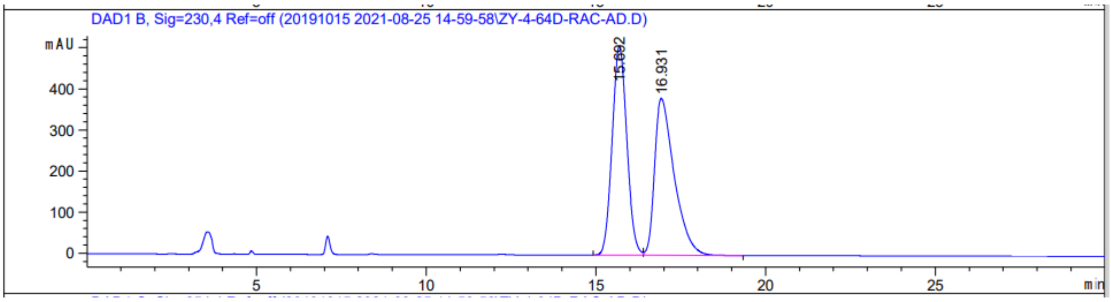

Peak	RetTime[min]	Type	Width[min]	Area[mAu*s]	Height[mAU]	Area[%]
1	15.692	BV	0.4717	1.53233e4	508.30688	49.9053
2	16.931	VB	0.6022	1.53814e4	381.60779	50.0947

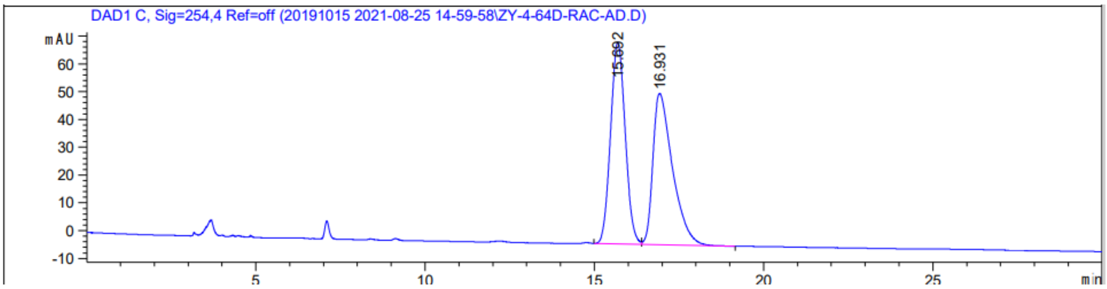

Peak	RetTime[min]	Type	Width[min]	Area[mAu*s]	Height[mAU]	Area[%]
1	15.692	BV	0.4733	2181.82495	72.46381	49.8621
2	16.931	VB	0.5961	2193.89722	54.44610	50.1379

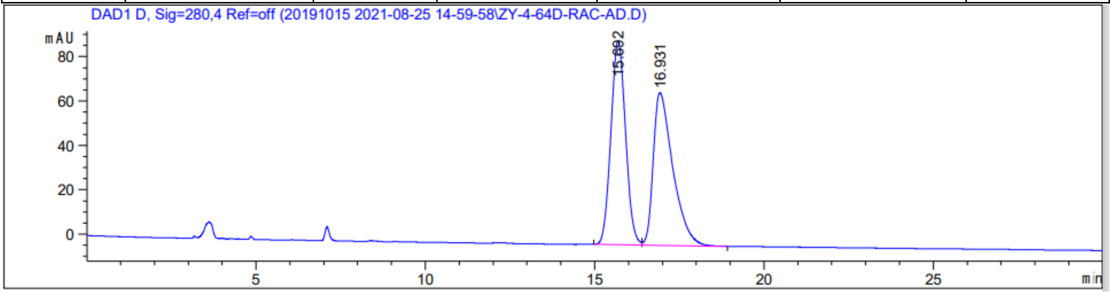

Peak	RetTime[min]	Type	Width[min]	Area[mAu*s]	Height[mAU]	Area[%]
1	15.692	BV	0.4710	2756.73047	91.63813	49.9084
2	16.931	VB	0.6056	2766.84888	68.73296	50.0916

==

End of Report

Sample Name: ZY-4-64E-OP

HPLC Condition: AD-H, *n*-Hexane/*i*PrOH = 95:5, 1.0 mL/min

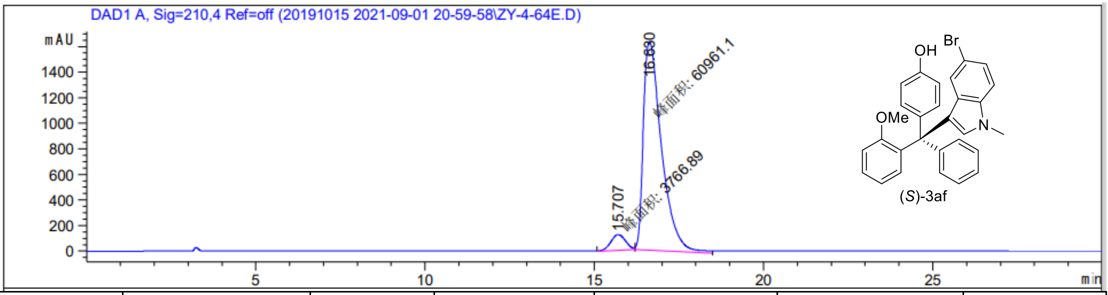

Peak	RetTime[min]	Type	Width[min]	Area[mAu*s]	Height[mAU]	Area[%]
1	15.707	MM	0.5089	3766.88525	123.35702	5.8196
2	16.630	MM	0.6233	6.09611e4	1629.97388	94.1804

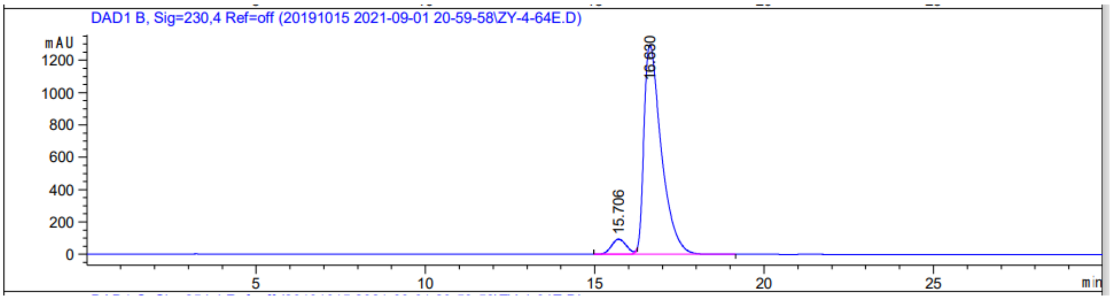

Peak	RetTime[min]	Type	Width[min]	Area[mAu*s]	Height[mAU]	Area[%]
1	15.706	BV E	0.4846	2889.01538	93.98617	5.8703
2	16.630	VB R	0.5373	4.63249e4	1294.98535	94.1297

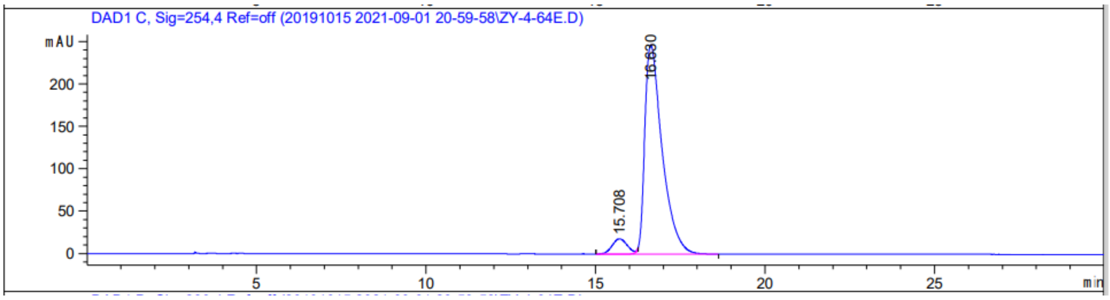

Peak	RetTime[min]	Type	Width[min]	Area[mAu*s]	Height[mAU]	Area[%]
1	15.708	BV E	0.4783	549.50311	17.89267	5.8772
2	16.630	VB R	0.5343	8800.22559	246.63966	94.1228

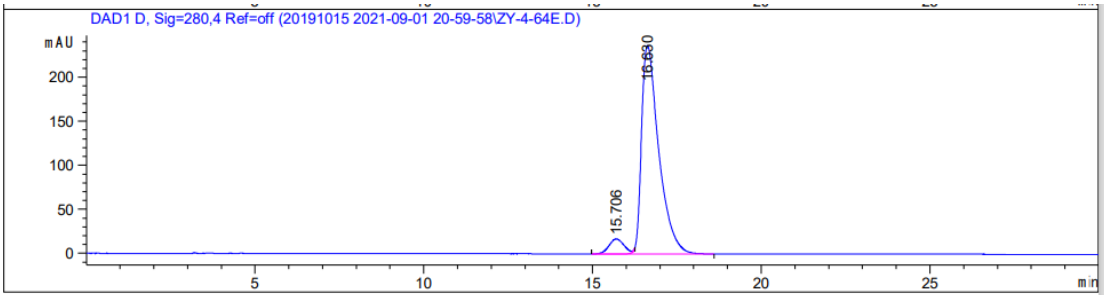

Peak	RetTime[min]	Type	Width[min]	Area[mAu*s]	Height[mAU]	Area[%]
1	15.706	BV E	0.4867	520.44202	16.92702	5.8329
2	16.630	VB R	0.5323	8402.09961	236.58792	94.1671

==

End of Report

Sample Name: ZY-4-64E-Rac

HPLC Condition: AD-H, *n*-Hexane/*i*PrOH = 95:5, 1.0 mL/min

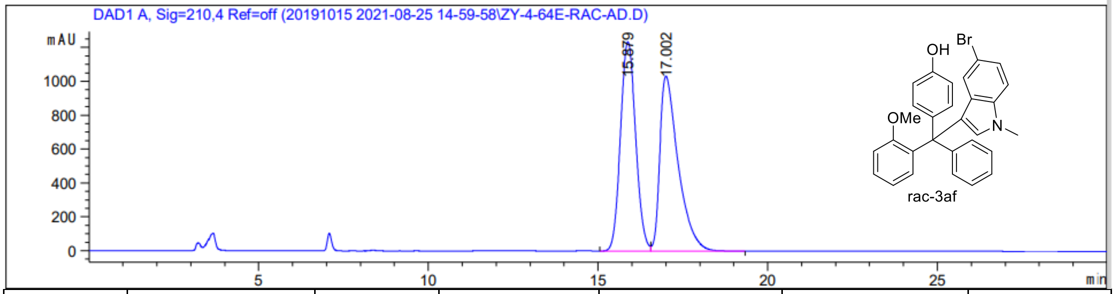

Peak	RetTime[min]	Type	Width[min]	Area[mAu*s]	Height[mAU]	Area[%]
1	15.879	BV	0.4866	3.83717e4	1234.77454	49.7372
2	17.002	VB	0.5583	3.87773e4	1032.26563	50.2628

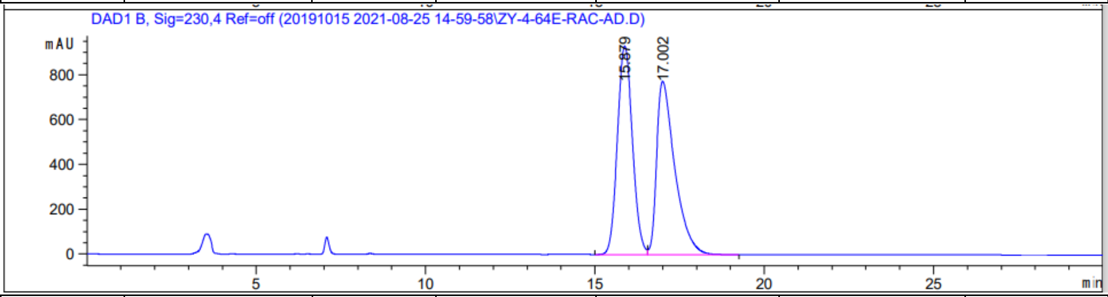

Peak	RetTime[min]	Type	Width[min]	Area[mAu*s]	Height[mAU]	Area[%]
1	15.879	BV	0.4836	2.86867e4	930.75751	49.8444
2	17.002	VB	0.5555	2.88658e4	773.29498	50.1556

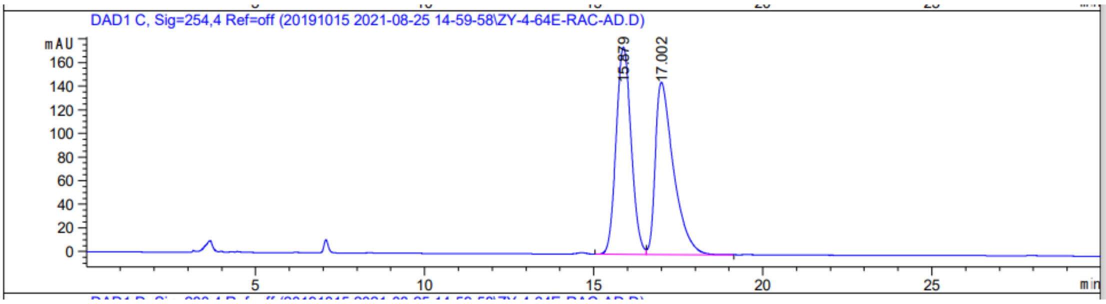

Peak	RetTime[min]	Type	Width[min]	Area[mAu*s]	Height[mAU]	Area[%]
1	15.879	BV	0.4841	5409.63330	175.28096	49.8185
2	17.002	VB	0.5563	5449.05518	145.70963	50.1815

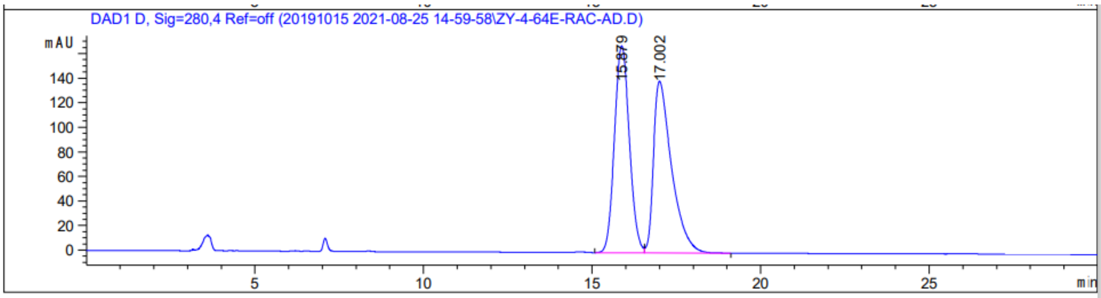

Peak	RetTime[min]	Type	Width[min]	Area[mAu*s]	Height[mAU]	Area[%]
1	15.879	BV	0.4809	5179.36377	168.34698	49.8589
2	17.002	VB	0.5589	5208.67432	139.71265	50.1411

==

End of Report

Sample Name: ZY-4-64H-OP

HPLC Condition: IC, *n*-Hexane/*i*PrOH = 95:5, 1.0 mL/min

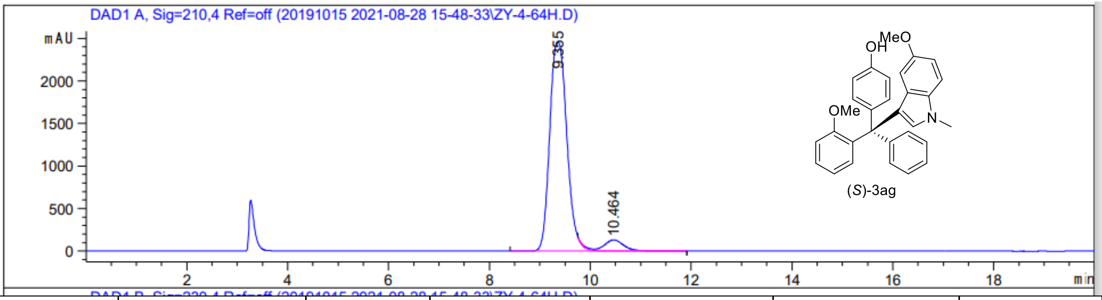

Peak	RetTime[min]	Type	Width[min]	Area[mAu*s]	Height[mAU]	Area[%]
1	9.355	BV R	0.3711	5.80965e4	2463.79224	93.9455
2	10.464	VB E	0.4274	3744.11377	131.02158	6.0545

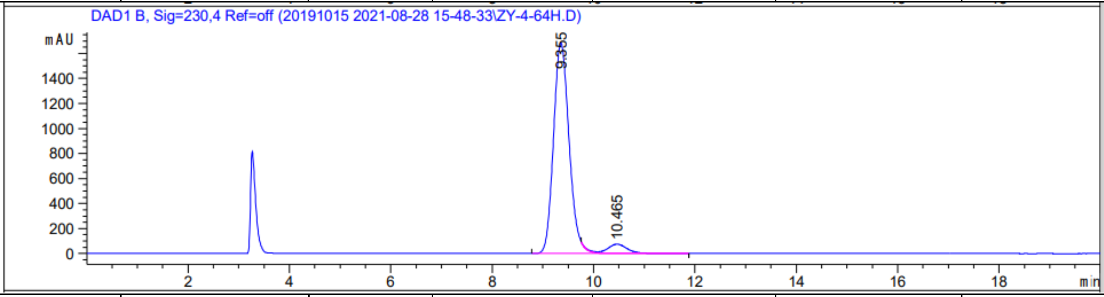

Peak	RetTime[min]	Type	Width[min]	Area[mAu*s]	Height[mAU]	Area[%]
1	9.355	BV R	0.3315	3.60475e4	1685.56714	94.4784
2	10.465	VB E	0.4267	2106.73975	74.33383	5.5216

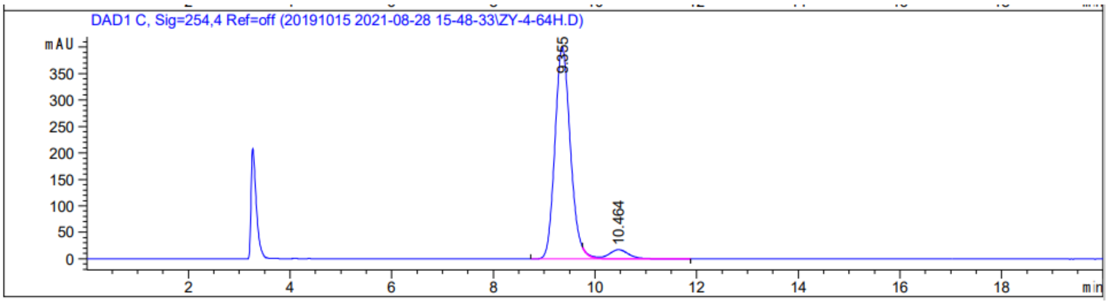

Peak	RetTime[min]	Type	Width[min]	Area[mAu*s]	Height[mAU]	Area[%]
1	9.355	BV R	0.3271	8472.44824	399.97189	94.5038
2	10.464	VB E	0.4259	492.74072	17.32134	5.4962

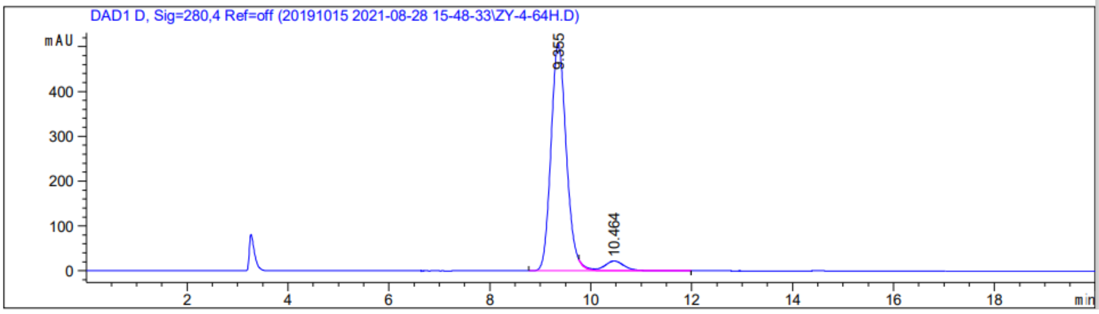

Peak	RetTime[min]	Type	Width[min]	Area[mAu*s]	Height[mAU]	Area[%]
1	9.355	BV R	0.3269	1.07122e4	506.11050	94.5399
2	10.464	VB E	0.4272	618.67200	21.79484	5.4601

==

End of Report

Sample Name: ZY-4-64H-Rac

HPLC Condition: IC, *n*-Hexane/*i*PrOH = 95:5, 1.0 mL/min

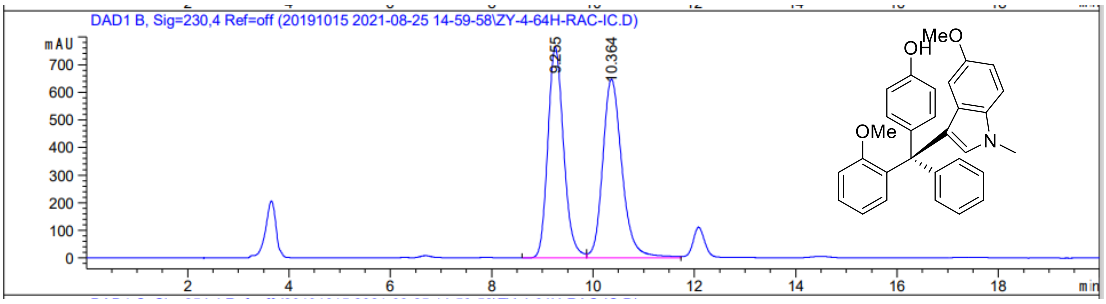

Peak	RetTime[min]	Type	Width[min]	Area[mAu*s]	Height[mAU]	Area[%]
1	9.255	BV	0.3347	1.65158e4	762.58624	49.0686
2	10.364	VV	0.4071	1.71428e4	647.05078	50.9314

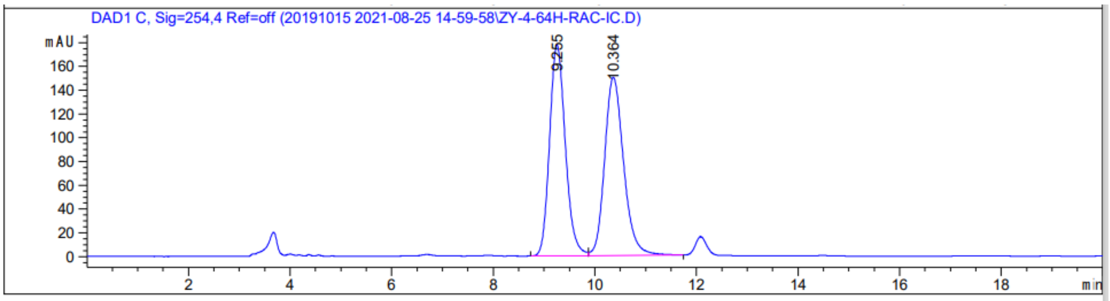

Peak	RetTime[min]	Type	Width[min]	Area[mAu*s]	Height[mAU]	Area[%]
1	9.255	BV	00.3335	3827.76367	177.55972	49.5941
2	10.364	VB	0.3984	3890.42017	150.07072	50.4059

==

End of Report

Sample Name: ZY-4-72-OP

HPLC Condition: AD-H, *n*-Hexane/*i*PrOH = 95:5, 1.0 mL/min

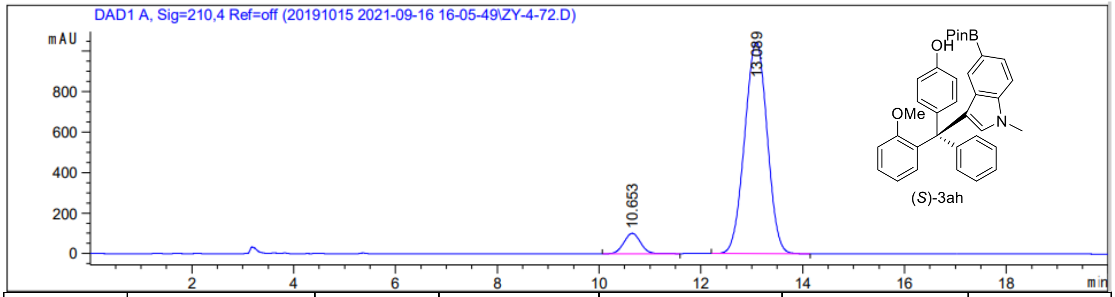

Peak	RetTime[min]	Type	Width[min]	Area[mAu*s]	Height[mAU]	Area[%]
1	10.653	BB	0.3566	2289.34448	100.93932	6.6498
2	13.089	BB	0.4864	3.21382e4	1046.02197	93.3502

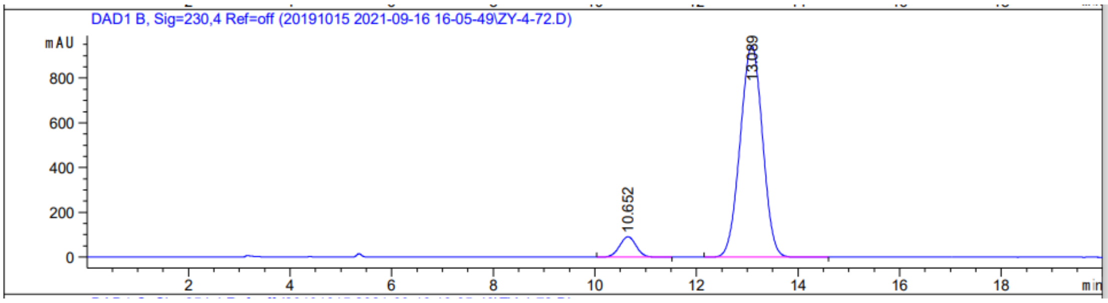

Peak	RetTime[min]	Type	Width[min]	Area[mAu*s]	Height[mAU]	Area[%]
1	10.652	BB	0.3546	2055.53223	90.62763	6.6400
2	13.089	BB	0.4832	2.89013e4	943.96124	93.3600

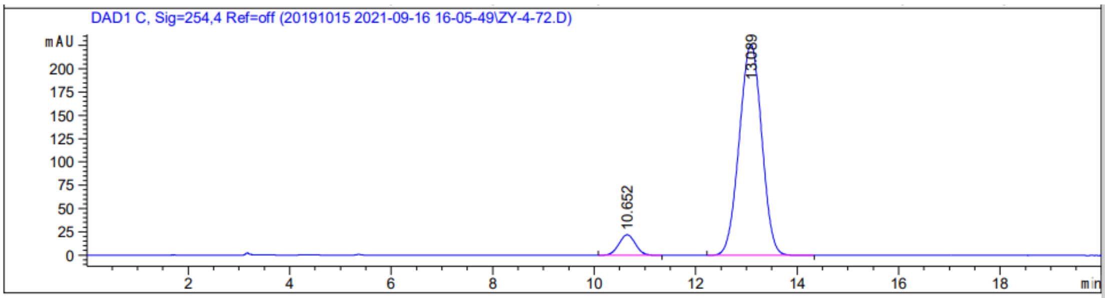

Peak	RetTime[min]	Type	Width[min]	Area[mAu*s]	Height[mAU]	Area[%]
1	10.652	BB	0.3524	498.56833	21.99595	6.6902
2	13.089	BB	0.4848	6953.68994	226.10506	93.3098

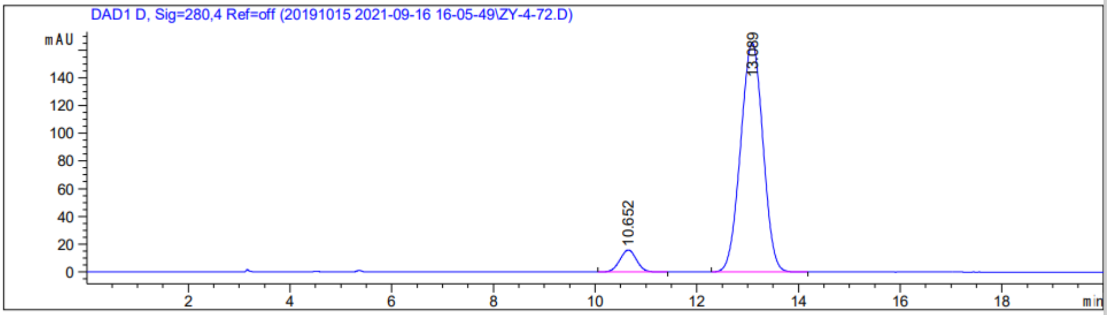

Peak	RetTime[min]	Type	Width[min]	Area[mAu*s]	Height[mAU]	Area[%]
1	10.652	BB	0.3513	359.67456	15.81786	6.6513
2	13.089	BB	0.4807	5047.93506	165.10831	93.3487

==

End of Report

Sample Name: ZY-4-72-Rac

HPLC Condition: AD-H, *n*-Hexane/*i*PrOH = 95:5, 1.0 mL/min

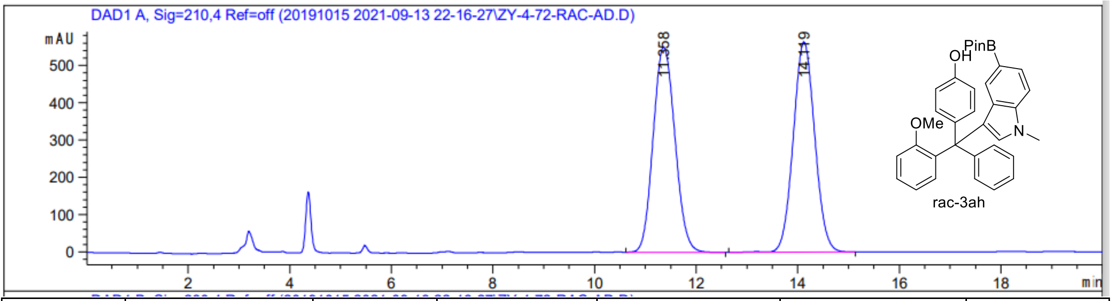

Peak	RetTime[min]	Type	Width[min]	Area[mAu*s]	Height[mAU]	Area[%]
1	11.358	BB	0.4719	1.64150e4	550.53082	49.7379
2	14.119	VB R	0.4586	1.65880e4	563.43976	50.2621

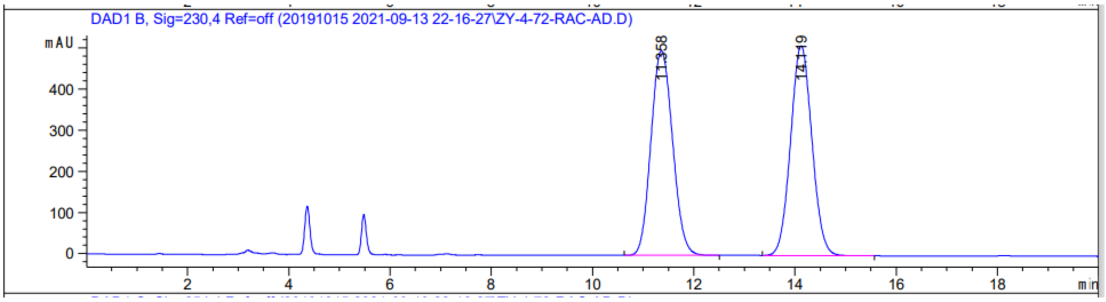

Peak	RetTime[min]	Type	Width[min]	Area[mAu*s]	Height[mAU]	Area[%]
1	11.358	BB	0.4706	1.47746e4	497.26596	49.7324
2	14.119	BB	0.4578	1.49335e4	509.71552	50.2676

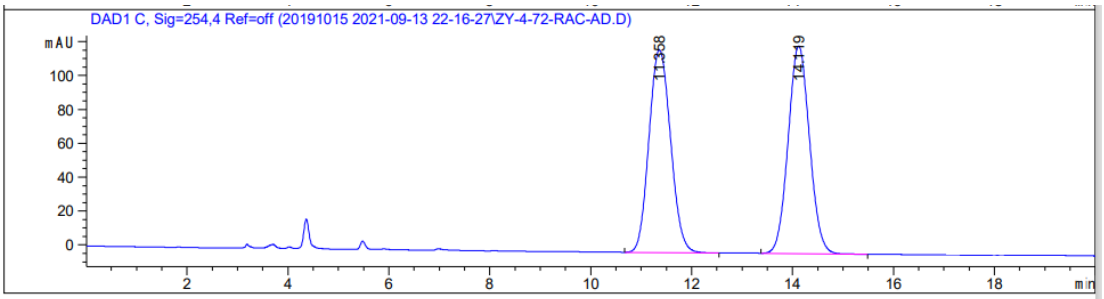

Peak	RetTime[min]	Type	Width[min]	Area[mAu*s]	Height[mAU]	Area[%]
1	11.358	BB	0.4713	3561.74194	119.63099	49.7409
2	14.119	BB	0.4565	3598.84253	122.58418	50.2591

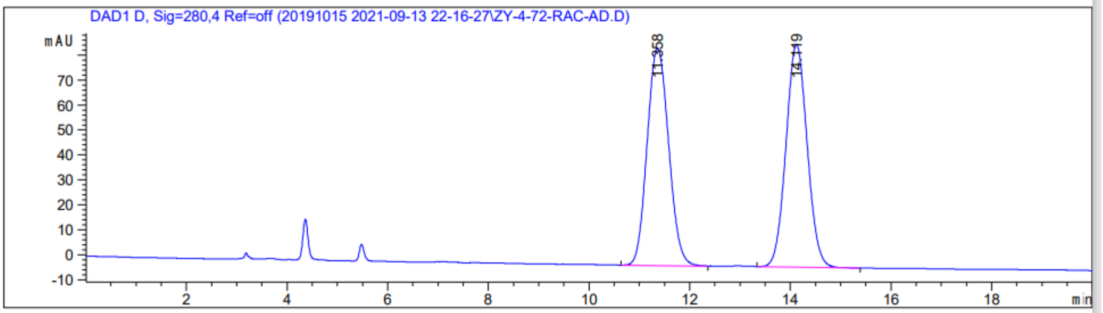

Peak	RetTime[min]	Type	Width[min]	Area[mAu*s]	Height[mAU]	Area[%]
1	11.358	BB	0.4723	2581.41968	86.95682	49.7134
2	14.119	BB	0.4557	2611.18091	89.16376	50.2866

==

End of Report

Sample Name: ZY-4-64A-OP

HPLC Condition: AD-H, *n*-Hexane/*i*PrOH = 95:5, 1.0 mL/min

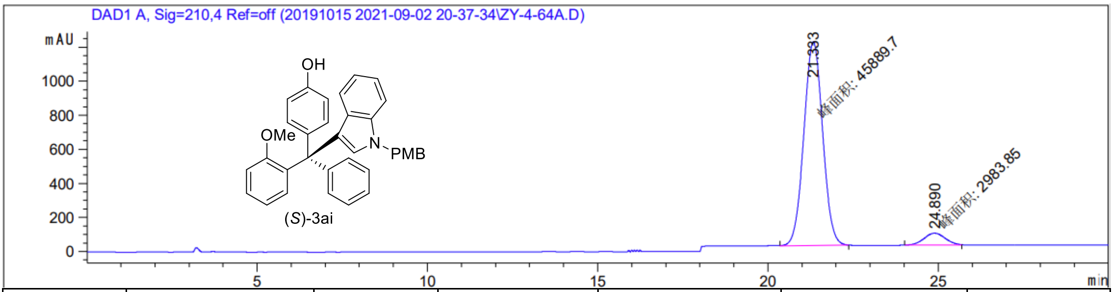

Peak	RetTime[min]	Type	Width[min]	Area[mAu*s]	Height[mAU]	Area[%]
1	21.333	MM	0.6400	4.58897e4	1195.09814	93.8947
2	24.890	MM	0.7186	2983.85107	69.20477	6.1053

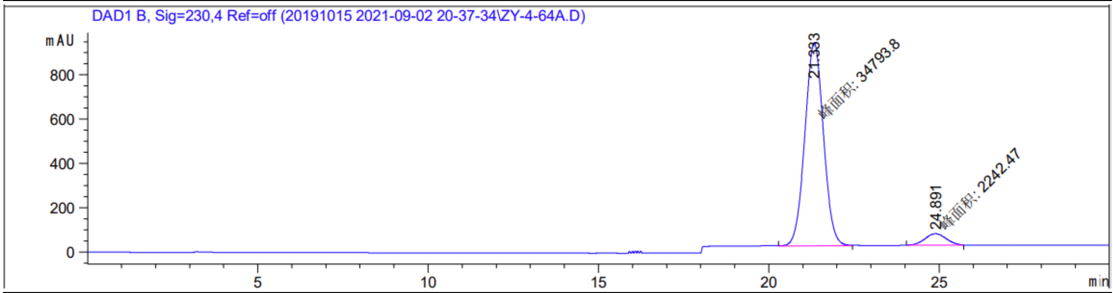

Peak	RetTime[min]	Type	Width[min]	Area[mAu*s]	Height[mAU]	Area[%]
1	21.333	MM	0.6331	3.47938e4	915.89697	93.9452
2	24.891	MM	0.7232	2242.46777	51.67600	6.0548

===

End of Report

Sample Name: ZY-4-58F-Rac

HPLC Condition: AD-H, *n*-Hexane/*i*PrOH = 95:5, 1.0 mL/min

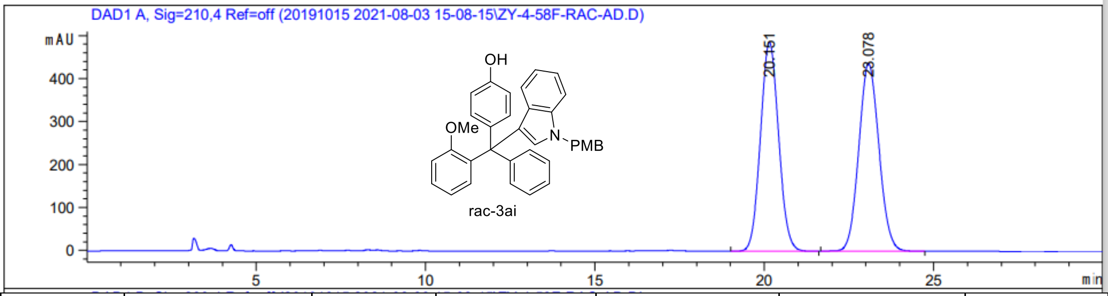

Peak	RetTime[min]	Type	Width[min]	Area[mAu*s]	Height[mAU]	Area[%]
1	20.151	BB	0.5806	1.81631e4	487.53928	50.0119
2	23.078	BB	0.6486	1.81545e4	437.04169	49.9881

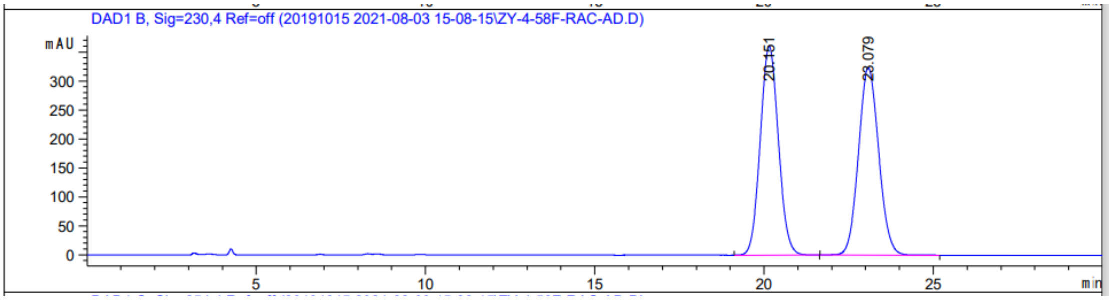

Peak	RetTime[min]	Type	Width[min]	Area[mAu*s]	Height[mAU]	Area[%]
1	20.151	BB	0.5805	1.34581e4	361.32782	50.0252
2	23.079	BB	0.6504	1.34446e4	323.78979	49.9748

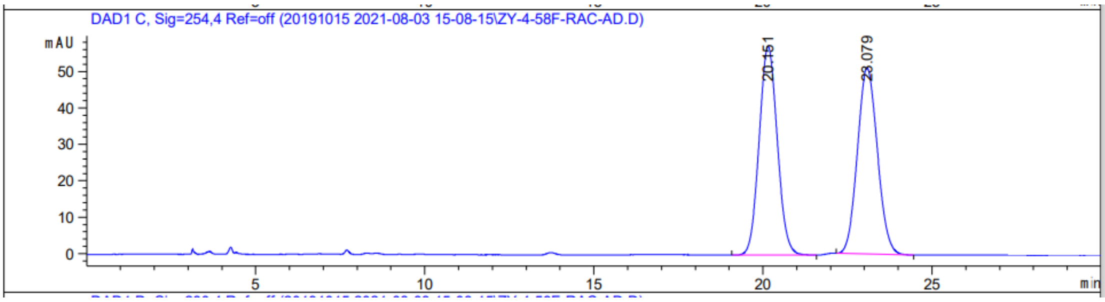

Peak	RetTime[min]	Type	Width[min]	Area[mAu*s]	Height[mAU]	Area[%]
1	20.151	BB	0.5826	2137.93188	57.38832	50.4650
2	23.079	BB	0.6390	2098.53223	51.10840	49.5350

==

End of Report

Sample Name: ZY-4-11D-OP

HPLC Condition: OD-H, *n*-Hexane/*i*PrOH = 98:2, 1.0 mL/min

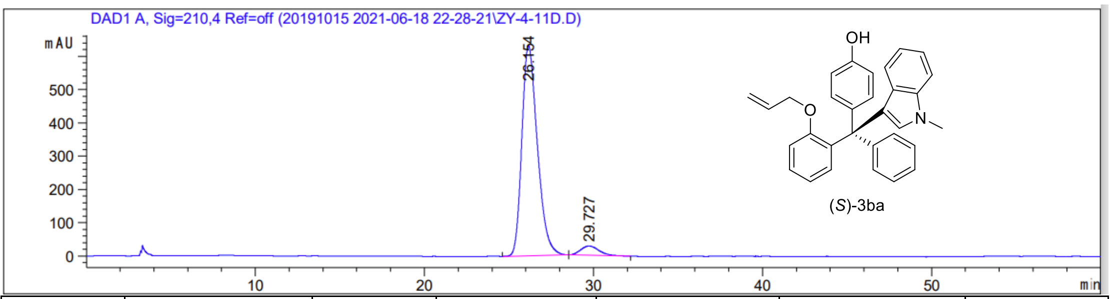

Peak	RetTime[min]	Type	Width[min]	Area[mAu*s]	Height[mAU]	Area[%]
1	26.154	BB	0.9733	4.02355e4	631.52771	94.8866
2	29.727	BB	1.1587	2168.29419	28.14940	5.1134

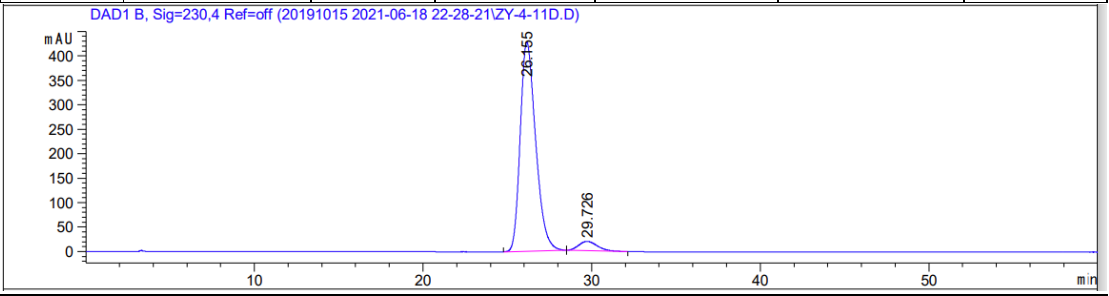

Peak	RetTime[min]	Type	Width[min]	Area[mAu*s]	Height[mAU]	Area[%]
1	26.155	BB	0.9767	2.71784e4	428.07825	94.9547
2	29.726	BB	0.9961	1444.09717	19.01363	5.0453

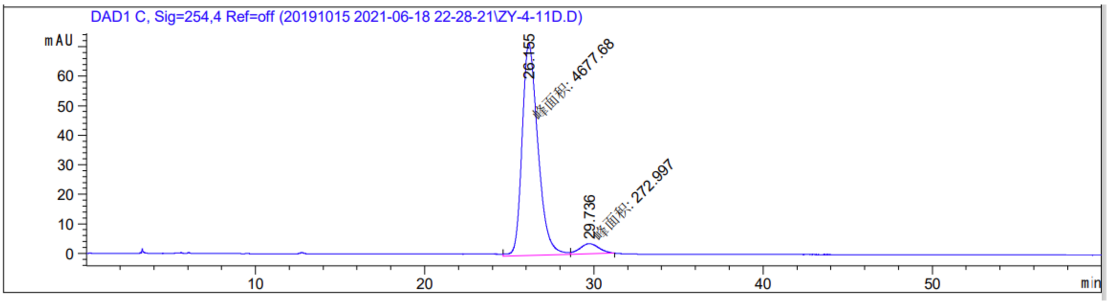

Peak	RetTime[min]	Type	Width[min]	Area[mAu*s]	Height[mAU]	Area[%]
1	26.155	MM	1.0863	4677.68164	71.77026	94.4857
2	29.736	MM	1.3180	272.99728	3.45228	5.5143

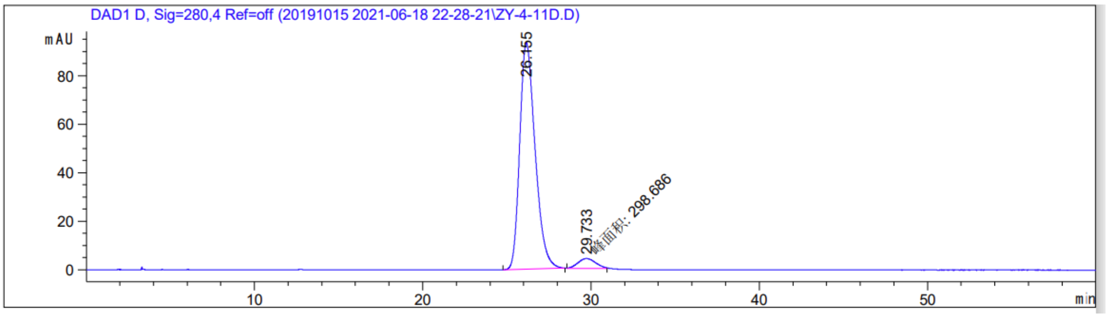

Peak	RetTime[min]	Type	Width[min]	Area[mAu*s]	Height[mAU]	Area[%]
1	26.155	BB	0.9718	5922.05273	93.38577	95.1985
2	29.733	MM	1.2184	298.68579	4.08576	4.8015

==

End of Report

Sample Name: ZY-4-12D-Rac

HPLC Condition: OD-H, *n*-Hexane/*i*PrOH = 98:2, 1.0 mL/min

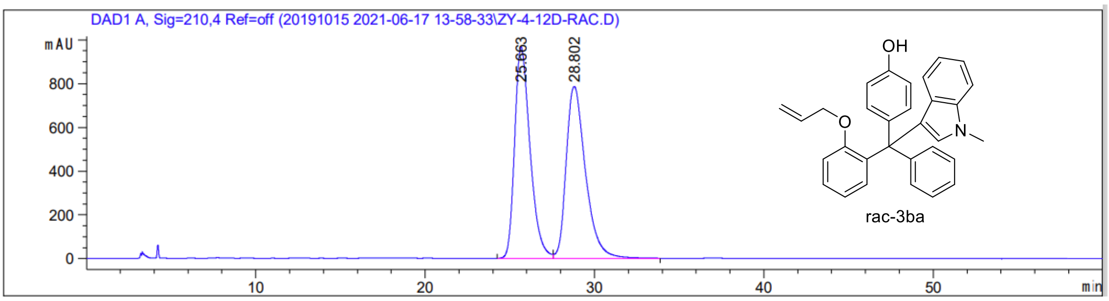

Peak	RetTime[min]	Type	Width[min]	Area[mAu*s]	Height[mAU]	Area[%]
1	25.663	BV	0.9612	6.11391e4	967.52429	49.5229
2	28.802	VB	1.2275	6.23172e4	785.55035	50.4771

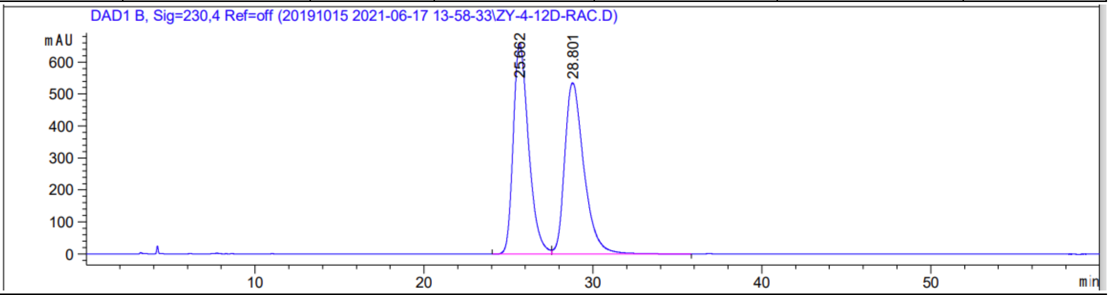

Peak	RetTime[min]	Type	Width[min]	Area[mAu*s]	Height[mAU]	Area[%]
1	25.662	BV	0.9602	4.16072e4	659.31635	49.5015
2	28.801	VB	1.2283	4.24452e4	534.60468	50.4985

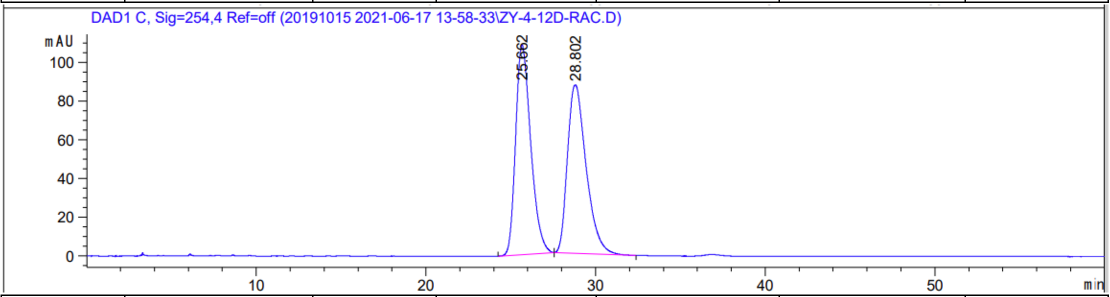

Peak	RetTime[min]	Type	Width[min]	Area[mAu*s]	Height[mAU]	Area[%]
1	25.662	BB	0.9485	6697.65918	108.44944	50.1161
2	28.802	BB	1.1537	6666.63281	87.02443	49.8839

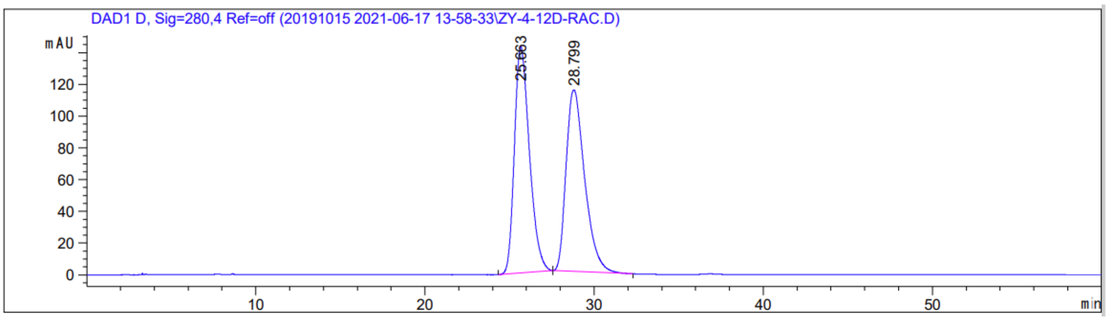

Peak	RetTime[min]	Type	Width[min]	Area[mAu*s]	Height[mAU]	Area[%]
1	25.663	BB	0.9399	8805.01660	142.69490	50.1301
2	28.799	BB	1.1490	8759.31543	114.43378	49.8699

==

End of Report

Sample Name: ZY-4-11B-OP

HPLC Condition: OD-H, *n*-Hexane/*i*PrOH = 95:5, 1.0 mL/min

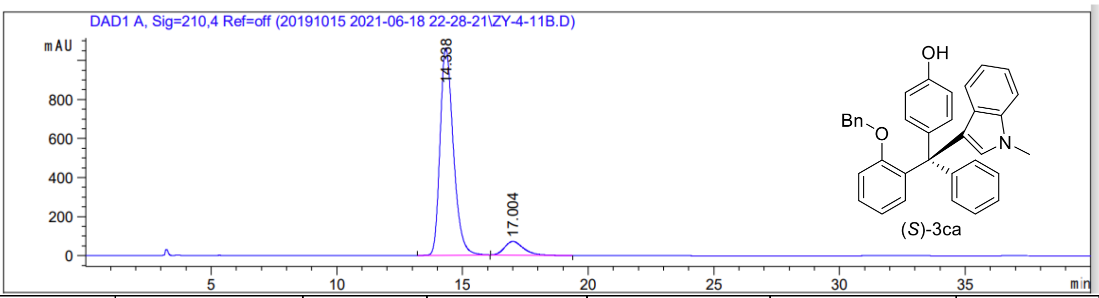

Peak	RetTime[min]	Type	Width[min]	Area[mAu*s]	Height[mAU]	Area[%]
1	14.338	BB	0.5649	3.90282e4	1061.66187	91.4191
2	17.004	BB	0.7935	3663.30835	69.89028	8.5809

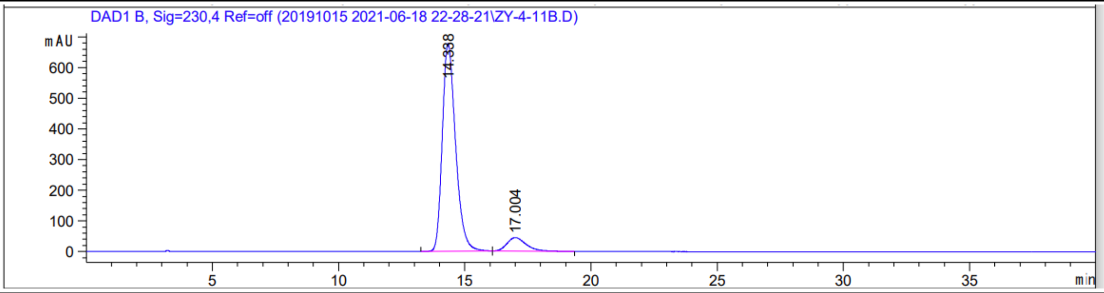

Peak	RetTime[min]	Type	Width[min]	Area[mAu*s]	Height[mAU]	Area[%]
1	14.338	BB	0.5631	2.47975e4	677.40002	91.4911
2	17.004	BB	0.7936	2306.22095	43.98960	8.5089

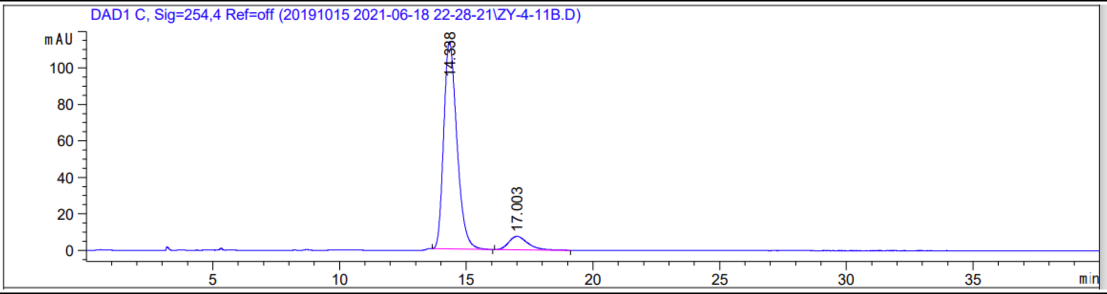

Peak	RetTime[min]	Type	Width[min]	Area[mAu*s]	Height[mAU]	Area[%]
1	14.338	BB	0.5585	4111.43115	113.52091	91.3892
2	17.003	BB	0.7550	387.38269	7.39644	8.6108

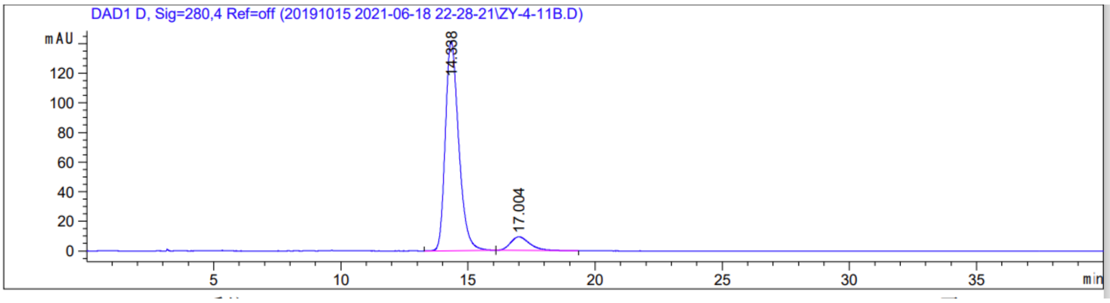

Peak	RetTime[min]	Type	Width[min]	Area[mAu*s]	Height[mAU]	Area[%]
1	14.338	BB	0.5605	5173.72803	141.52975	91.4908
2	17.004	BB	0.7687	481.18784	9.16187	8.5092

==

End of Report

Sample Name: ZY-4-12B-Rac

HPLC Condition: OD-H, *n*-Hexane/*i*PrOH = 95:5, 1.0 mL/min

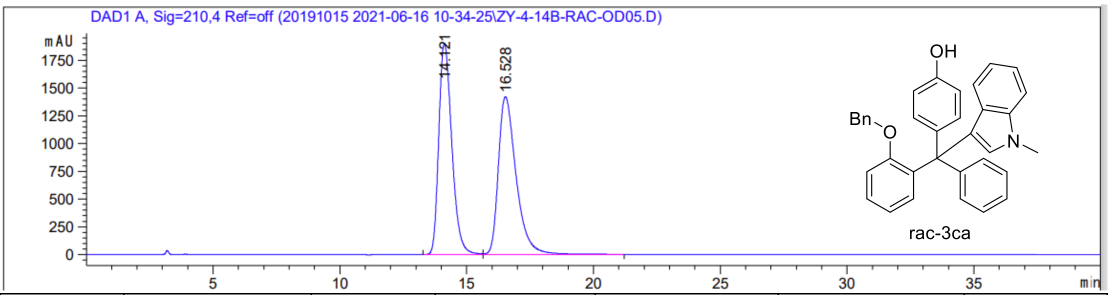

Peak	RetTime[min]	Type	Width[min]	Area[mAu*s]	Height[mAU]	Area[%]
1	14.121	BV	0.5560	6.80881e4	1900.42444	49.4775
2	16.528	VB	0.7464	6.95261e4	1422.05139	50.5225

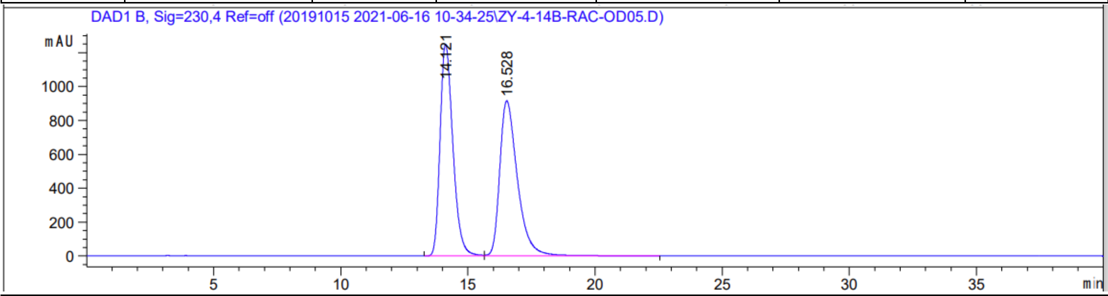

Peak	RetTime[min]	Type	Width[min]	Area[mAu*s]	Height[mAU]	Area[%]
1	14.121	BV	0.5420	4.39160e4	1249.60400	49.6455
2	16.528	VB	0.7356	4.45431e4	915.55652	50.3545

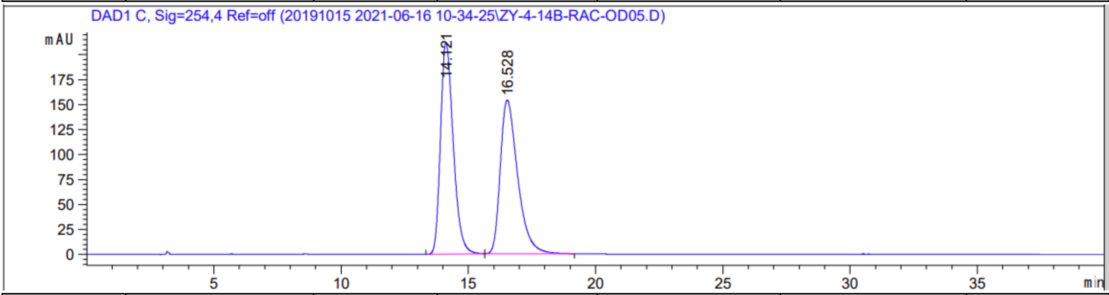

Peak	RetTime[min]	Type	Width[min]	Area[mAu*s]	Height[mAU]	Area[%]
1	14.121	BB	0.5380	7365.54736	211.66806	50.2100
2	16.528	BB	0.7221	7303.92236	153.78413	49.7900

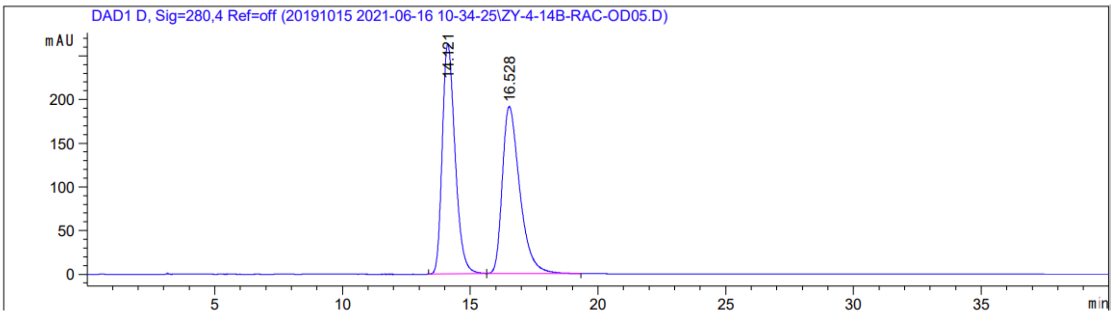

Peak	RetTime[min]	Type	Width[min]	Area[mAu*s]	Height[mAU]	Area[%]
1	14.121	BB	0.5374	9144.06250	263.13327	50.2153
2	16.528	BB	0.7238	9065.63965	190.97426	49.7847

==

End of Report

Sample Name: ZY-4-56D-OP

HPLC Condition: AD-H, *n*-Hexane/*i*PrOH = 98:2, 1.0 mL/min

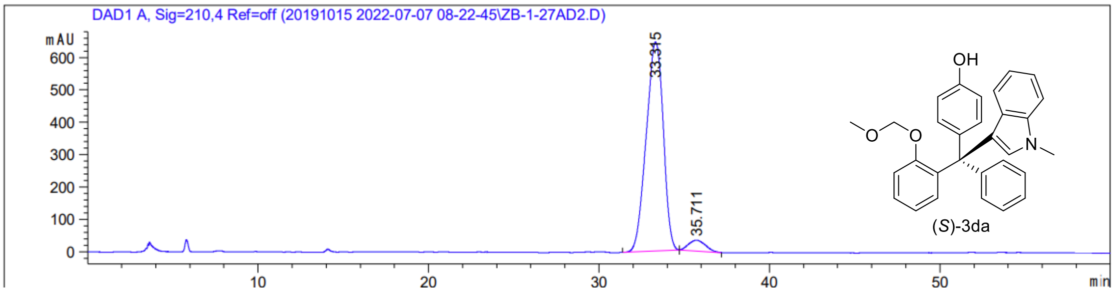

Peak	RetTime[min]	Type	Width[min]	Area[mAu*s]	Height[mAU]	Area[%]
1	33.315	BB	1.0309	4.48186e4	644.80151	95.2037
2	35.711	BB	0.8071	2257.91821	33.04969	4.7963

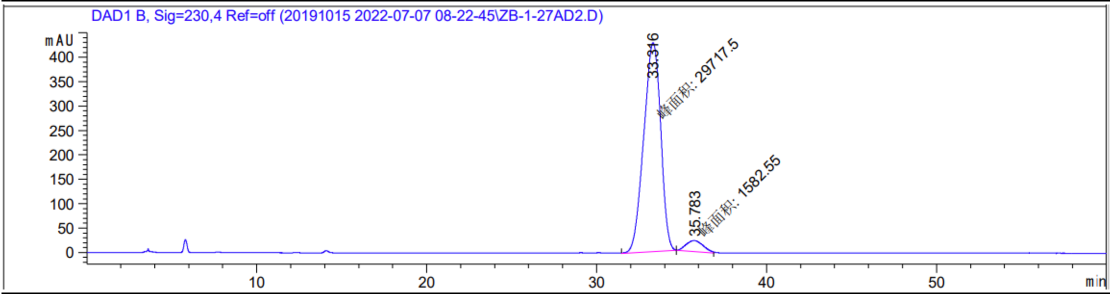

Peak	RetTime[min]	Type	Width[min]	Area[mAu*s]	Height[mAU]	Area[%]
1	33.316	MM	1.1584	2.97175e4	427.55460	94.9439
2	35.783	MM	1.1750	1582.55432	22.44680	5.0561

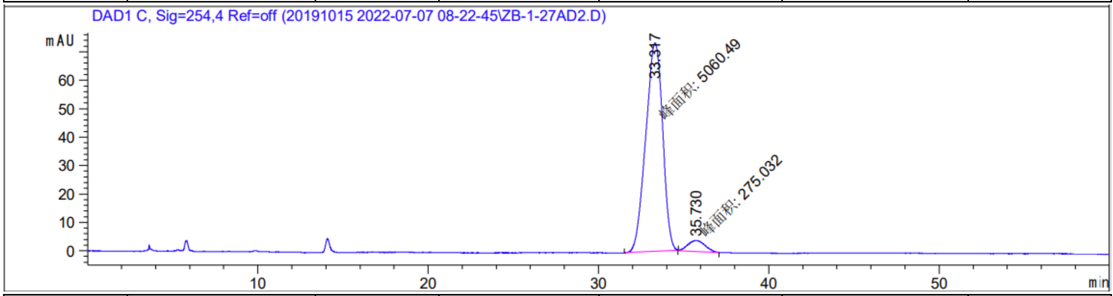

Peak	RetTime[min]	Type	Width[min]	Area[mAu*s]	Height[mAU]	Area[%]
1	33.317	MM	1.1519	5060.49463	73.21964	94.8453
2	35.730	MM	1.1851	275.03207	3.86804	5.1547

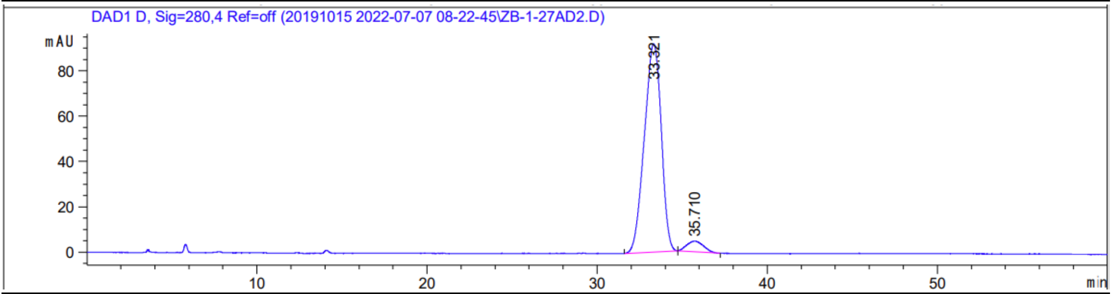

Peak	RetTime[min]	Type	Width[min]	Area[mAu*s]	Height[mAU]	Area[%]
1	33.321	BB	1.0453	6348.31201	91.74047	95.1839
2	35.710	BB	0.8200	321.21048	4.63816	4.8161

==

End of Report

Sample Name: ZY-4-56D-Rac

HPLC Condition: AD-H, *n*-Hexane/*i*PrOH = 98:2, 1.0 mL/min

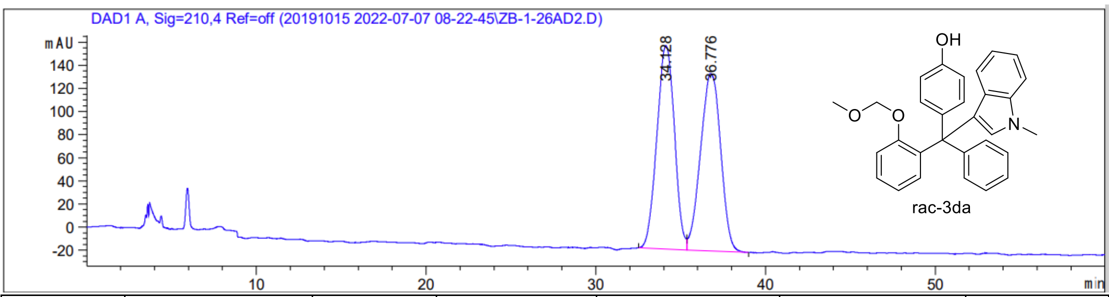

Peak	RetTime[min]	Type	Width[min]	Area[mAu*s]	Height[mAU]	Area[%]
1	34.128	BV	1.0456	1.27011e4	176.06079	49.9244
2	36.776	VB	1.0912	1.27396e4	153.66977	50.0756

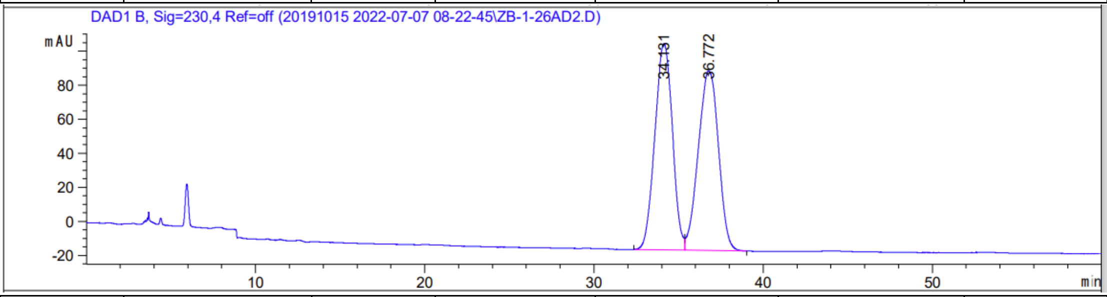

Peak	RetTime[min]	Type	Width[min]	Area[mAu*s]	Height[mAU]	Area[%]
1	34.131	BV	1.0983	8750.81738	120.91535	49.9949
2	36.772	VB	1.2135	8752.58691	105.54608	50.0051

==

End of Report

Sample Name: ZY-4-75F-OP

HPLC Condition: IC, *n*-Hexane/*i*PrOH = 98:2, 1.0 mL/min

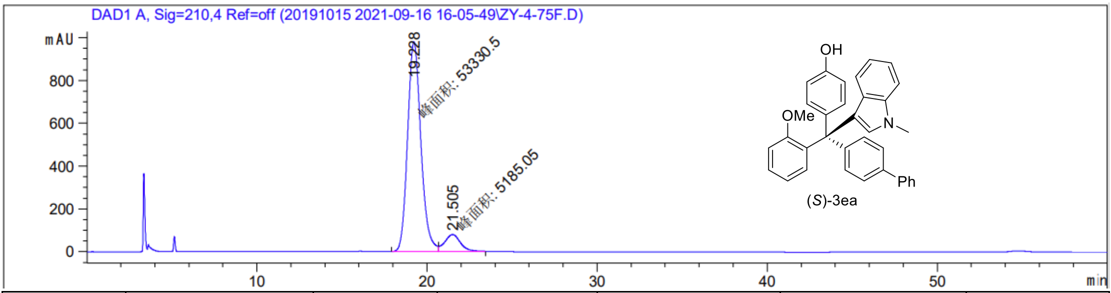

Peak	RetTime[min]	Type	Width[min]	Area[mAu*s]	Height[mAU]	Area[%]
1	19.228	MF	0.9070	5.33305e4	979.97205	91.1390
2	21.505	FM	1.0876	5185.04639	79.45735	8.8610

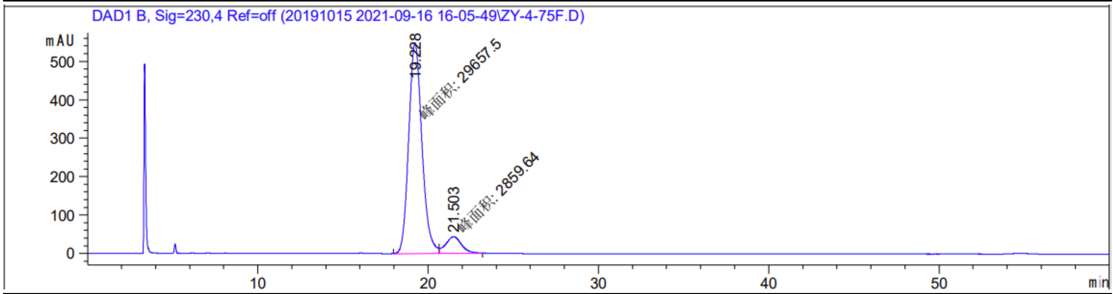

Peak	RetTime[min]	Type	Width[min]	Area[mAu*s]	Height[mAU]	Area[%]
1	19.228	MF	0.9022	2.96575e4	547.87006	91.2057
2	21.503	FM	1.0893	2859.64258	43.75487	8.7943

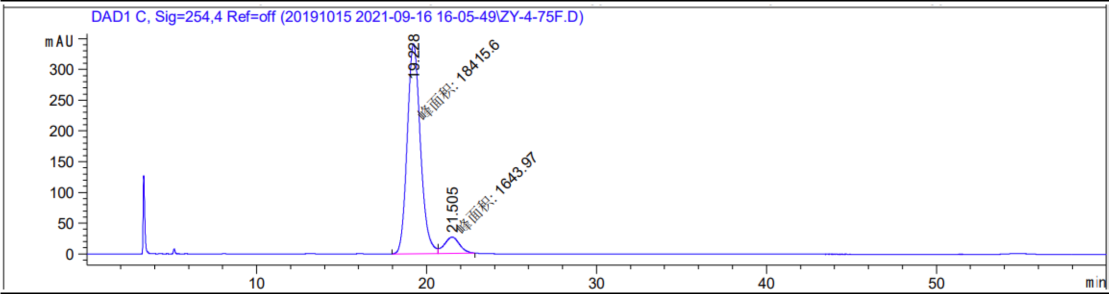

Peak	RetTime[min]	Type	Width[min]	Area[mAu*s]	Height[mAU]	Area[%]
1	19.228	MF	0.9001	1.84156e4	340.97632	91.8046
2	21.505	FM	1.0359	1643.97302	26.45043	8.1954

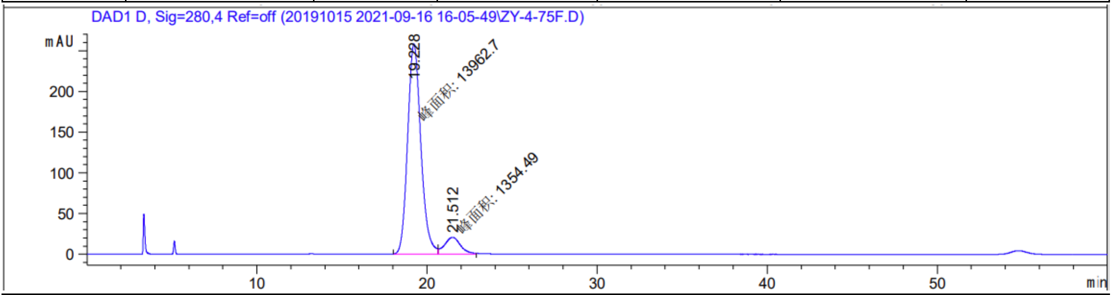

Peak	RetTime[min]	Type	Width[min]	Area[mAu*s]	Height[mAU]	Area[%]
1	19.228	MF	0.9017	1.39627e4	258.08026	91.1571
2	21.512	FM	1.0904	1354.48950	20.70230	8.8429

==

End of Report

Sample Name: ZY-4-75F-Rac

HPLC Condition: IC, *n*-Hexane/*i*PrOH = 98:2, 1.0 mL/min

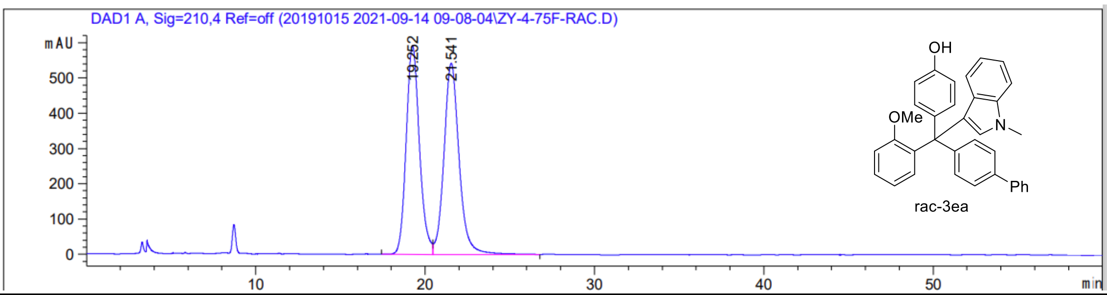

Peak	RetTime[min]	Type	Width[min]	Area[mAu*s]	Height[mAU]	Area[%]
1	19.252	BV	0.8326	3.21876e4	591.71118	48.8316
2	21.541	VB	0.9315	3.37279e4	542.38434	51.1684

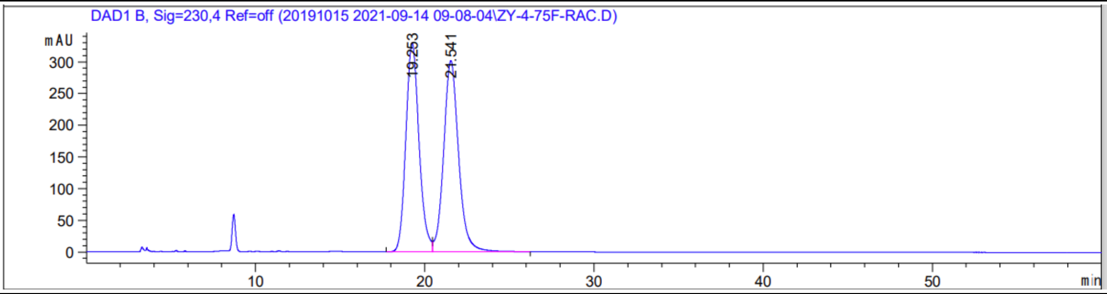

Peak	RetTime[min]	Type	Width[min]	Area[mAu*s]	Height[mAU]	Area[%]
1	19.253	BV	0.8287	1.78251e4	329.72247	49.0497
2	21.541	VB	0.9242	1.85158e4	301.63037	50.9503

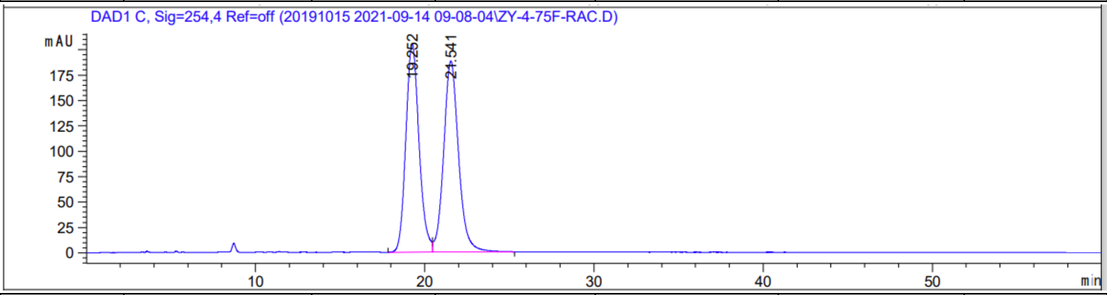

Peak	RetTime[min]	Type	Width[min]	Area[mAu*s]	Height[mAU]	Area[%]
1	19.252	BV	0.8269	1.10948e4	205.16454	49.1246
2	21.541	VB	0.9231	1.14902e4	187.98532	50.8754

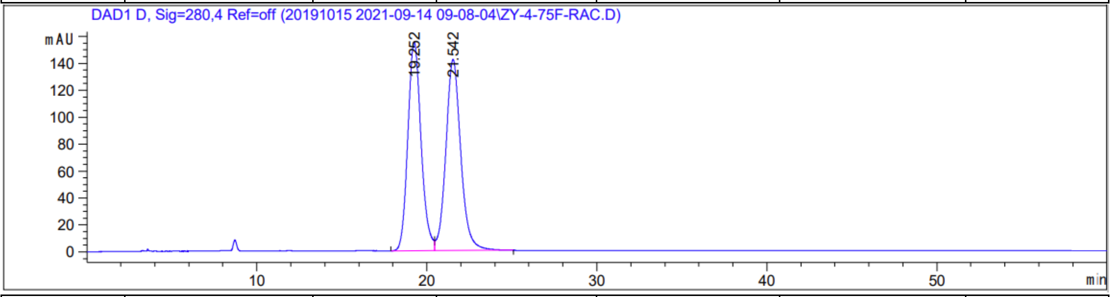

Peak	RetTime[min]	Type	Width[min]	Area[mAu*s]	Height[mAU]	Area[%]
1	19.252	BV	0.8247	8379.25195	155.00005	49.1500
2	21.542	VB	0.9243	8669.07227	141.98108	50.8500

==

End of Report

Sample Name: ZY-4-11A-OP

HPLC Condition: IC, *n*-Hexane/*i*PrOH = 98:2, 0.4 mL/min

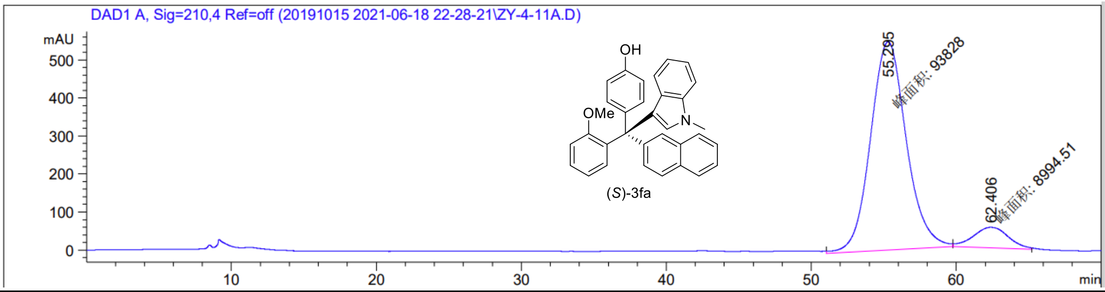

Peak	RetTime[min]	Type	Width[min]	Area[mAu*s]	Height[mAU]	Area[%]
1	55.295	MM	2.8563	9.38280e4	547.48517	91.2524
2	62.406	MM	2.7948	8994.51270	53.63879	8.7476

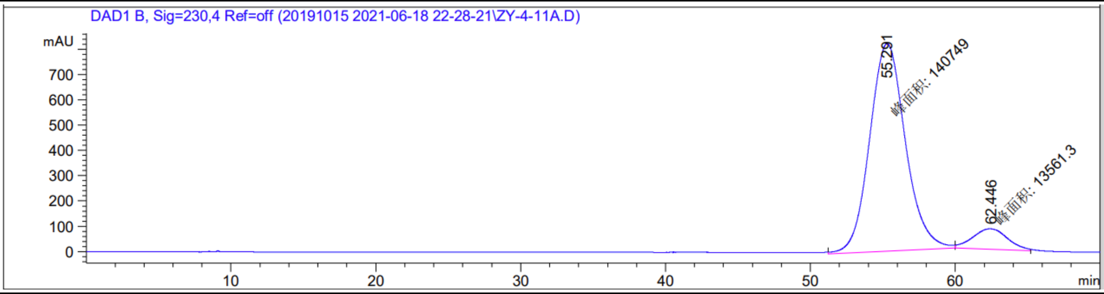

Peak	RetTime[min]	Type	Width[min]	Area[mAu*s]	Height[mAU]	Area[%]
1	55.291	MM	2.8563	1.40749e5	821.28400	91.2117
2	62.446	MM	2.7905	1.35613e4	80.99666	8.7883

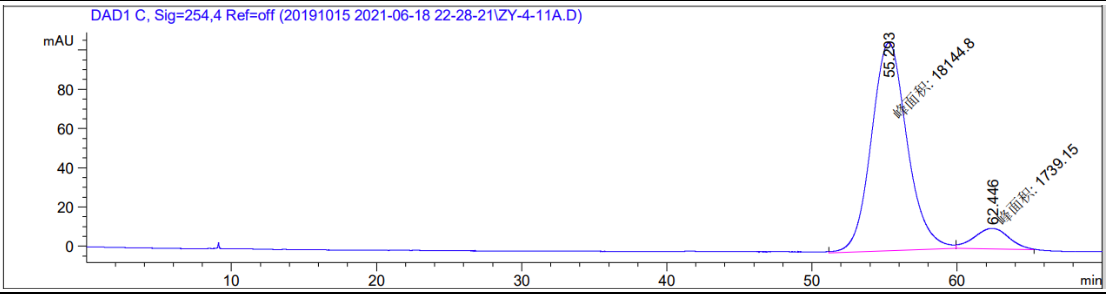

Peak	RetTime[min]	Type	Width[min]	Area[mAu*s]	Height[mAU]	Area[%]
1	55.293	MM	2.8524	1.81448e4	106.02246	91.2535
2	62.446	MM	2.7888	1739.15088	10.39352	8.7465

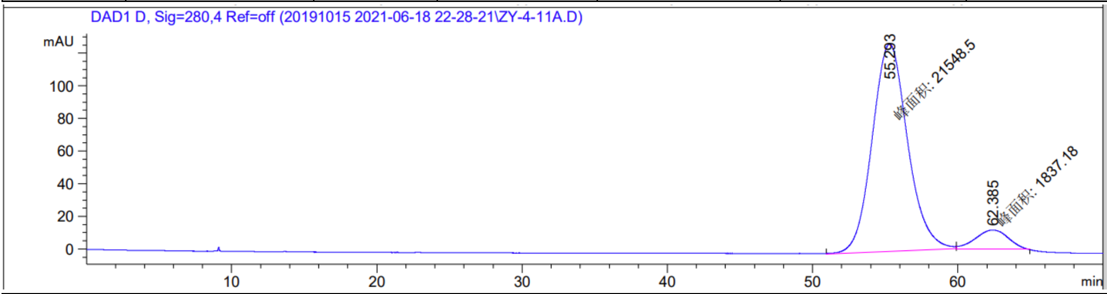

Peak	RetTime[min]	Type	Width[min]	Area[mAu*s]	Height[mAU]	Area[%]
1	55.293	MM	2.8245	2.15485e4	127.15305	92.1440
2	62.385	MM	2.6228	1837.18213	11.67423	7.8560

==

End of Report

Sample Name: ZY-4-11A-Rac

HPLC Condition: IC, *n*-Hexane/*i*PrOH = 98:2, 0.4 mL/min

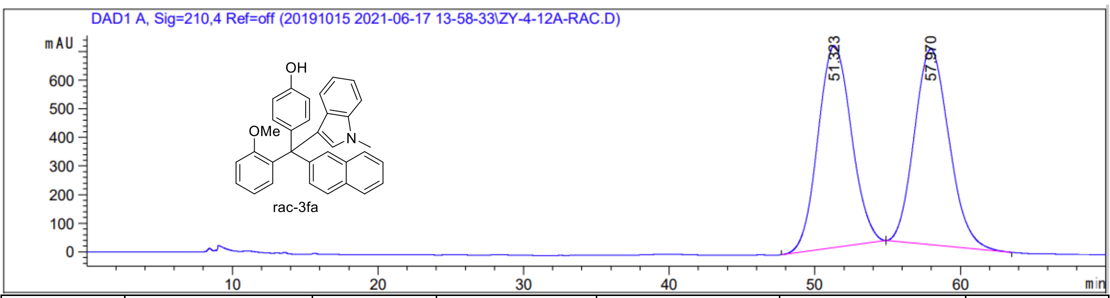

Peak	RetTime[min]	Type	Width[min]	Area[mAu*s]	Height[mAU]	Area[%]
1	51.323	BB	2.4589	1.12516e5	705.41901	49.8330
2	57.970	BB	2.4406	1.13270e5	684.50464	50.1670

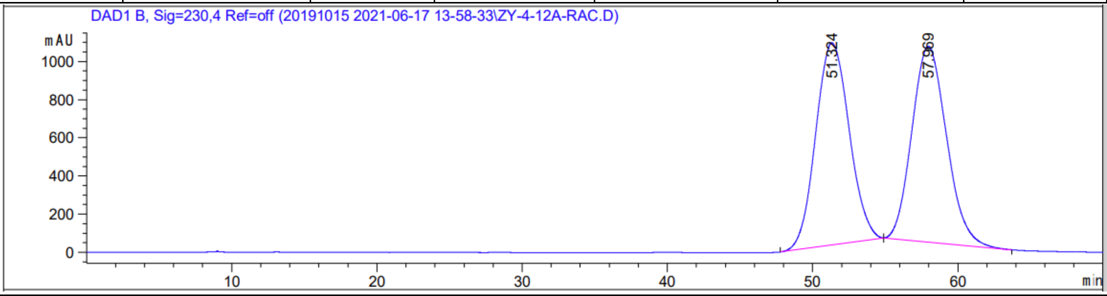

Peak	RetTime[min]	Type	Width[min]	Area[mAu*s]	Height[mAU]	Area[%]
1	51.324	BB	2.5104	1.69475e5	1059.37891	49.8726
2	57.969	BBA	2.4677	1.70341e5	1026.67859	50.1274

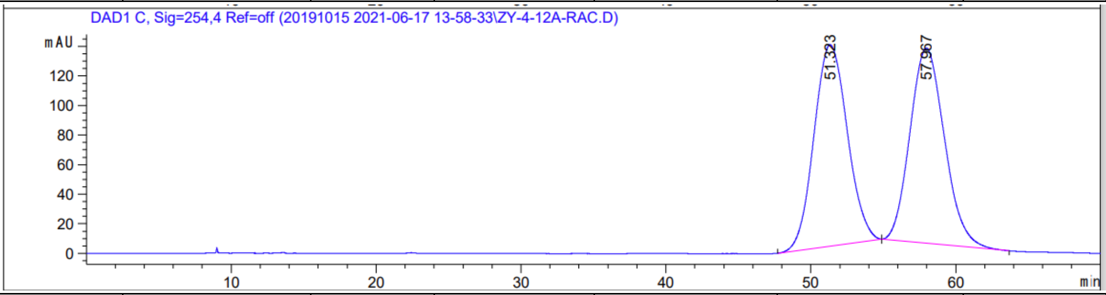

Peak	RetTime[min]	Type	Width[min]	Area[mAu*s]	Height[mAU]	Area[%]
1	51.323	BB	2.3798	2.17805e4	136.33533	49.9019
2	57.967	BB	2.3470	2.18661e4	132.10832	50.0981

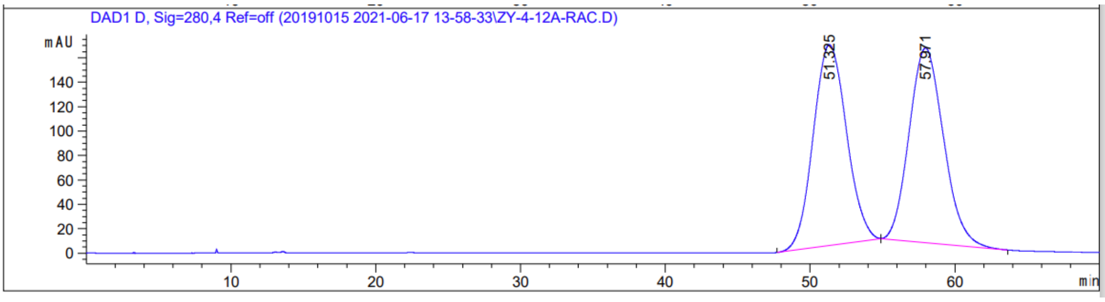

Peak	RetTime[min]	Type	Width[min]	Area[mAu*s]	Height[mAU]	Area[%]
1	51.325	BB	2.4346	2.62362e4	164.50879	49.9000
2	57.971	BB	2.3767	2.63414e4	159.34221	50.1000

==

End of Report

Sample Name: ZY-4-50B-OP

HPLC Condition: AD-H, *n*-Hexane/*i*PrOH = 95:5, 1.0 mL/min

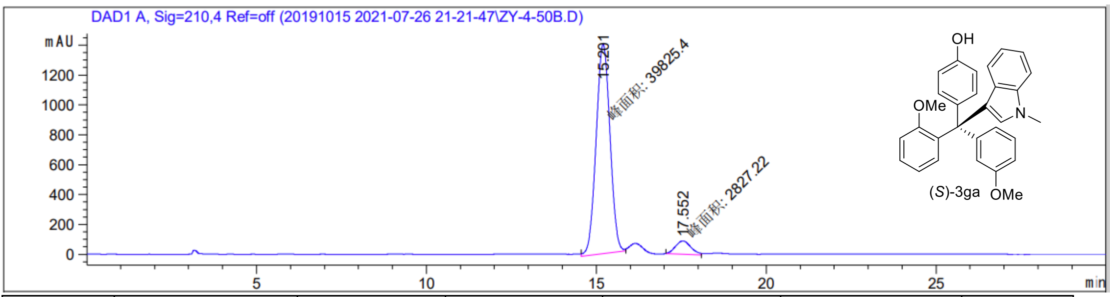

Peak	RetTime[min]	Type	Width[min]	Area[mAu*s]	Height[mAU]	Area[%]
1	15.201	MM	0.4729	3.98254e4	1403.68213	93.3715
2	17.552	MM	0.5288	2827.21558	89.11159	6.6285

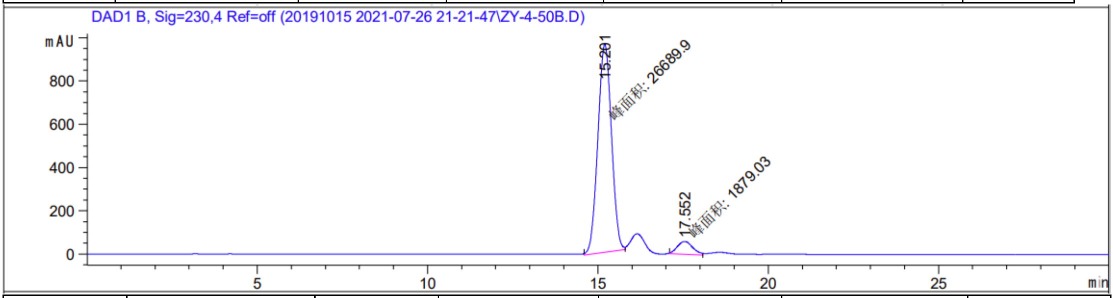

Peak	RetTime[min]	Type	Width[min]	Area[mAu*s]	Height[mAU]	Area[%]
1	15.201	MM	0.4618	2.66899e4	963.15753	93.4228
2	17.552	MM	0.5244	1879.02783	59.71721	6.5772

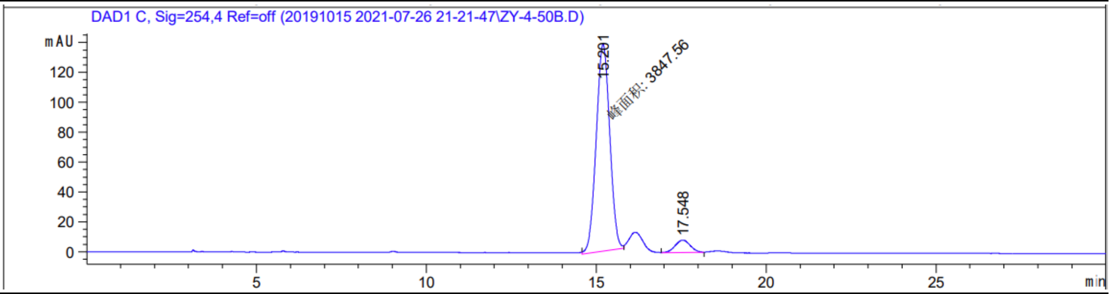

Peak	RetTime[min]	Type	Width[min]	Area[mAu*s]	Height[mAU]	Area[%]
1	15.201	MM	0.4624	3847.55933	138.69316	93.9543
2	17.548	BB	0.4597	247.57930	8.16686	6.0457

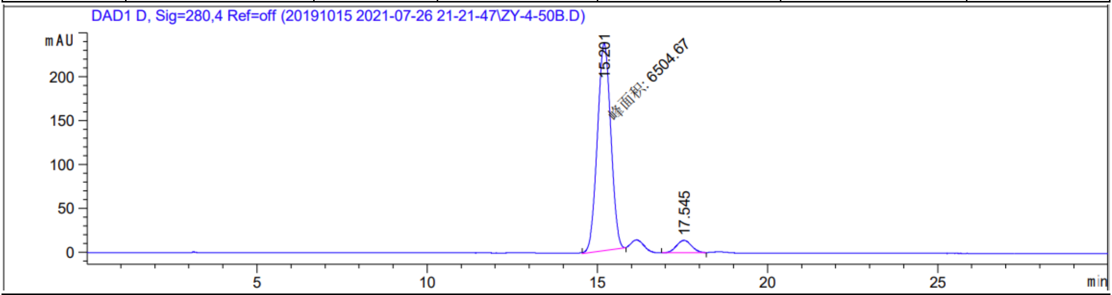

Peak	RetTime[min]	Type	Width[min]	Area[mAu*s]	Height[mAU]	Area[%]
1	15.201	MM	0.4585	6504.66504	236.46246	93.8045
2	17.545	BB	0.4769	429.61636	14.04553	6.1955

==

End of Report

Sample Name: ZY-4-50B-rac

HPLC Condition: AD-H, *n*-Hexane/*i*PrOH = 95:5, 1.0 mL/min

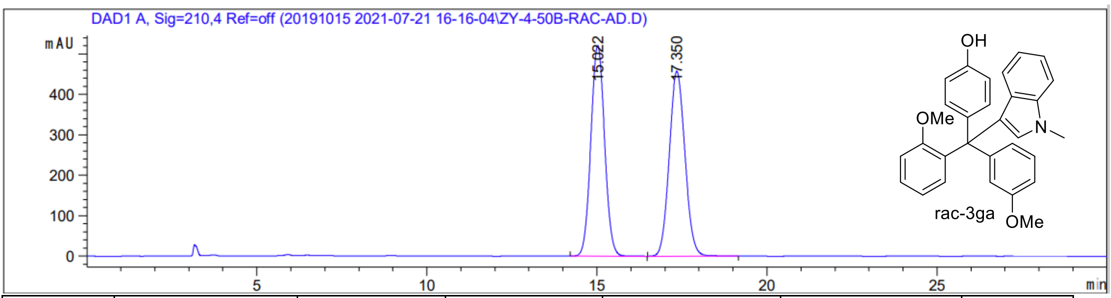

Peak	RetTime[min]	Type	Width[min]	Area[mAu*s]	Height[mAU]	Area[%]
1	15.022	BB	0.4423	1.46326e4	520.03491	49.9202
2	17.350	BB	0.5025	1.46794e4	457.26123	50.0798

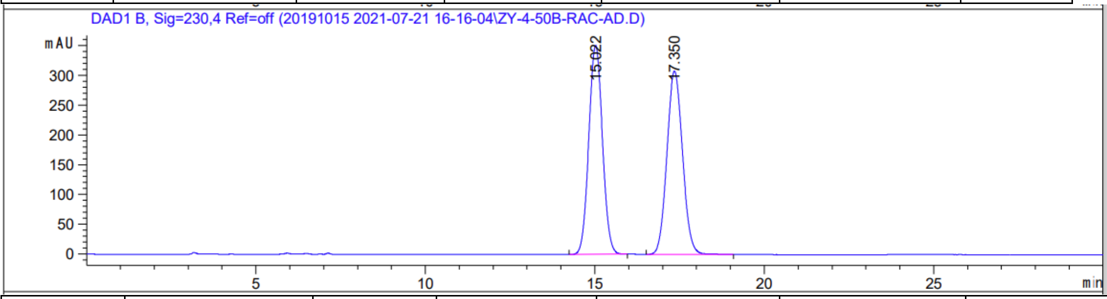

Peak	RetTime[min]	Type	Width[min]	Area[mAu*s]	Height[mAU]	Area[%]
1	15.022	BB	0.4385	9787.67188	349.76077	49.8286
2	17.350	BB	0.4998	9854.99902	307.61804	50.1714

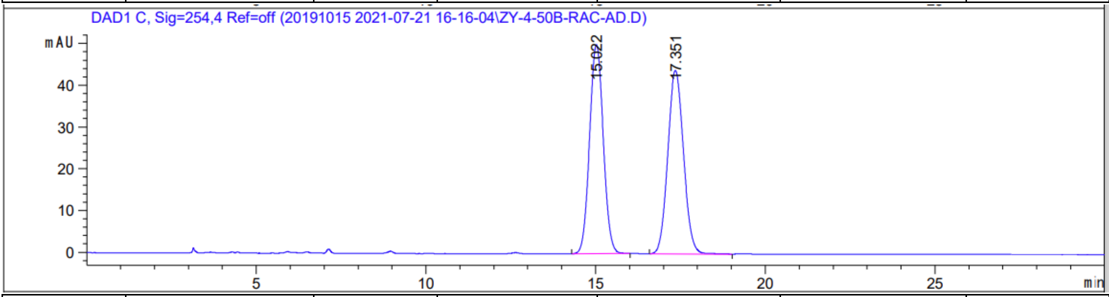

Peak	RetTime[min]	Type	Width[min]	Area[mAu*s]	Height[mAU]	Area[%]
1	15.022	BB	0.4387	1400.12854	49.99246	49.7915
2	17.351	BB	0.4986	1411.85608	1411.85608	50.2085

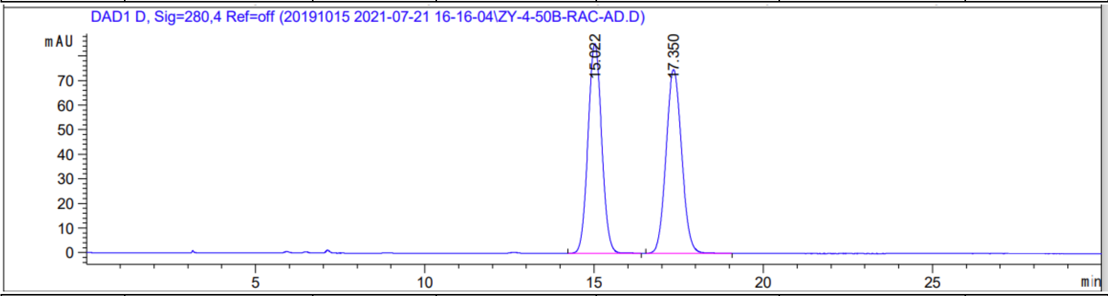

Peak	RetTime[min]	Type	Width[min]	Area[mAu*s]	Height[mAU]	Area[%]
1	15.022	BB	0.4394	2391.53589	85.21094	49.9420
2	17.350	BB	0.4976	2397.08862	74.85547	50.0580

==

End of Report

Sample Name: ZY-4-50D-OP

HPLC Condition: IC, *n*-Hexane/*i*PrOH = 95:5, 1.0 mL/min

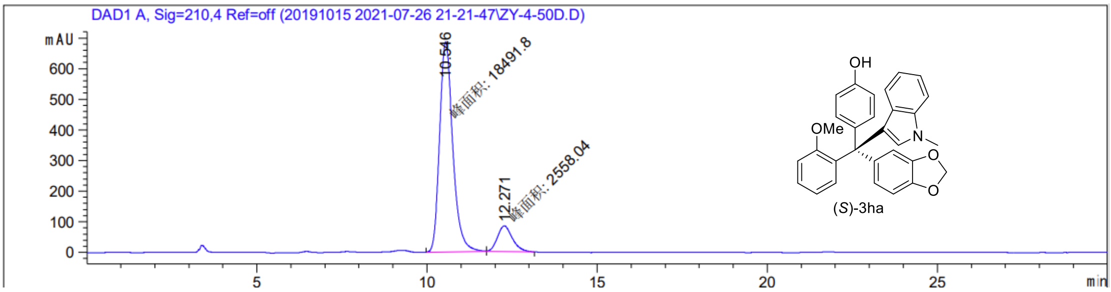

Peak	RetTime[min]	Type	Width[min]	Area[mAu*s]	Height[mAU]	Area[%]
1	10.546	MM	0.4492	1.84918e4	686.14526	87.8477
2	12.271	MM	0.5087	2558.04004	83.80598	12.1523

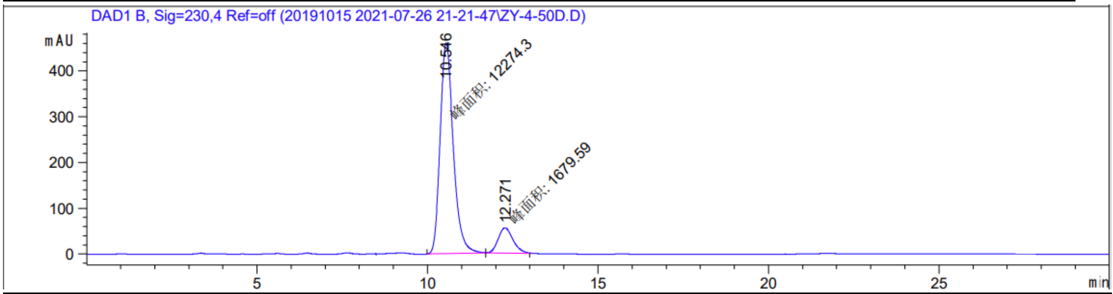

Peak	RetTime[min]	Type	Width[min]	Area[mAu*s]	Height[mAU]	Area[%]
1	10.546	MM	0.4448	1.22743e4	459.92218	87.9633
2	12.271	MM	0.5032	1679.58850	55.63019	12.0367

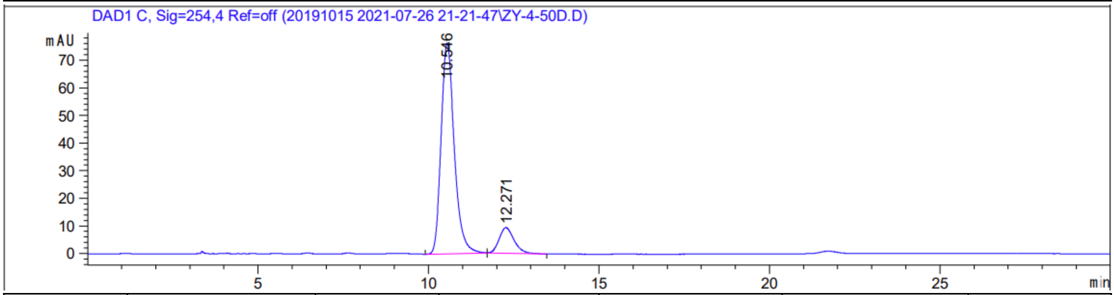

Peak	RetTime[min]	Type	Width[min]	Area[mAu*s]	Height[mAU]	Area[%]
1	10.546	BB	0.4077	2036.88586	76.24246	87.9598
2	12.271	BB	0.4466	278.81509	9.22429	12.0402

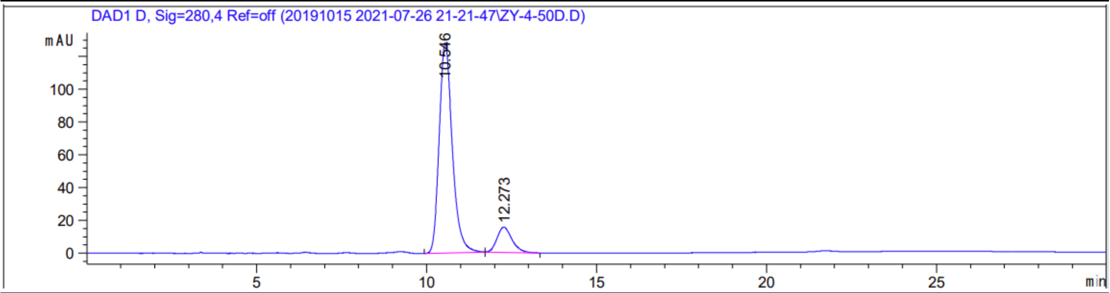

Peak	RetTime[min]	Type	Width[min]	Area[mAu*s]	Height[mAU]	Area[%]
1	10.546	BB	0.4072	3415.32446	128.01926	88.0004
2	12.273	BB	0.4613	465.70682	15.37901	11.9996

==

End of Report

Sample Name: ZY-4-50D-rac

HPLC Condition: IC, *n*-Hexane/*i*PrOH = 95:5, 1.0 mL/min

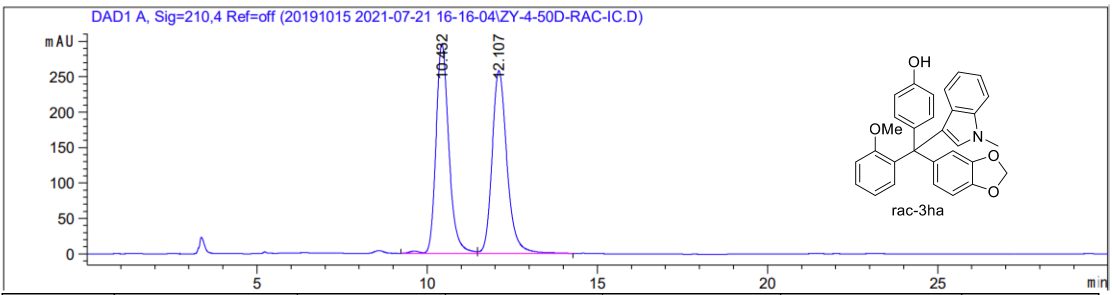

Peak	RetTime[min]	Type	Width[min]	Area[mAu*s]	Height[mAU]	Area[%]
1	10.432	VV R	0.4005	7775.15283	295.67719	49.7944
2	12.107	VB	0.4628	7839.35205	257.75842	50.2056

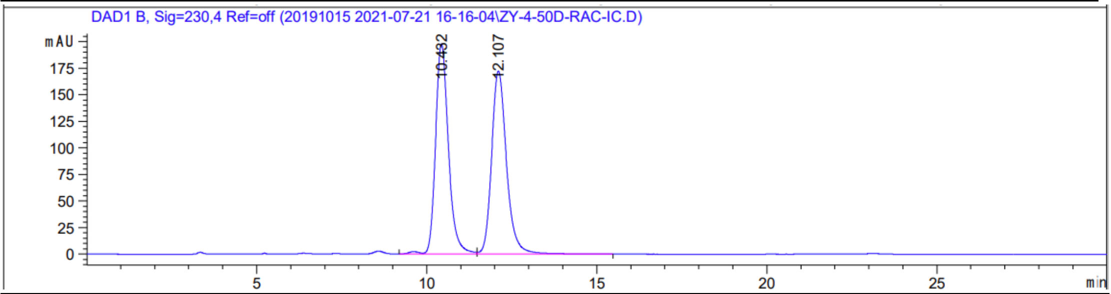

Peak	RetTime[min]	Type	Width[min]	Area[mAu*s]	Height[mAU]	Area[%]
1	10.432	VV R	0.4009	5207.04395	197.60466	49.8020
2	12.107	VB	0.4655	5248.44580	172.23392	50.1980

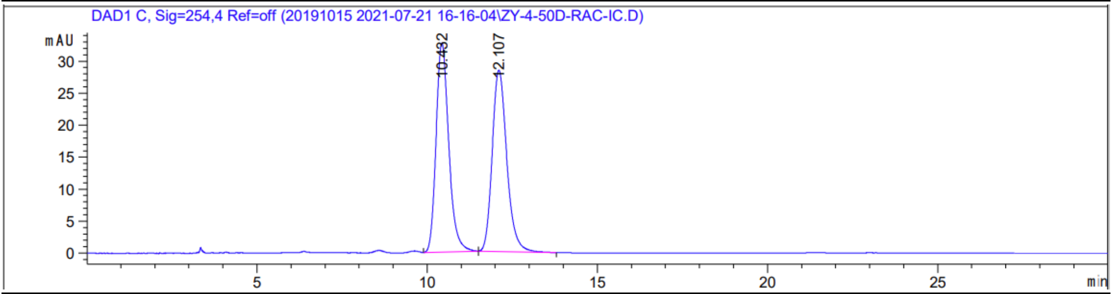

Peak	RetTime[min]	Type	Width[min]	Area[mAu*s]	Height[mAU]	Area[%]
1	10.432	BB	0.3954	843.62958	32.65502	50.0088
2	12.107	BB	0.4572	843.33136	28.33437	49.9912

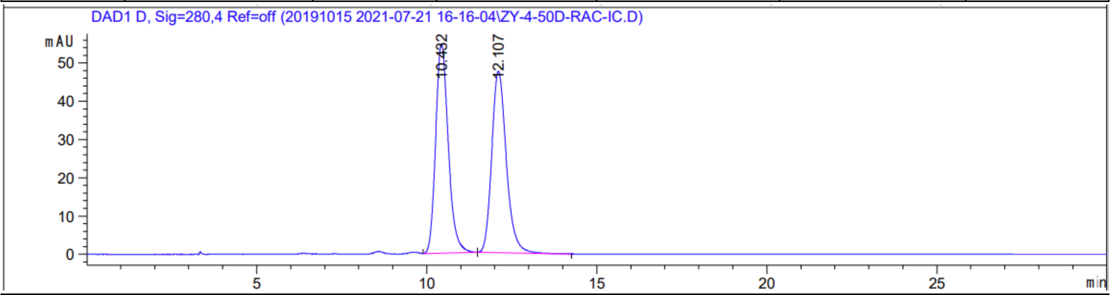

Peak	RetTime[min]	Type	Width[min]	Area[mAu*s]	Height[mAU]	Area[%]
1	10.432	BB	0.3962	1406.30859	54.64863	49.8687
2	12.107	BB	0.4554	1413.71204	47.47541	50.1313

==

End of Report

Sample Name: ZY-4-75B-OP

HPLC Condition: AD-H, *n*-Hexane/*i*PrOH = 98:2, 1.0 mL/min

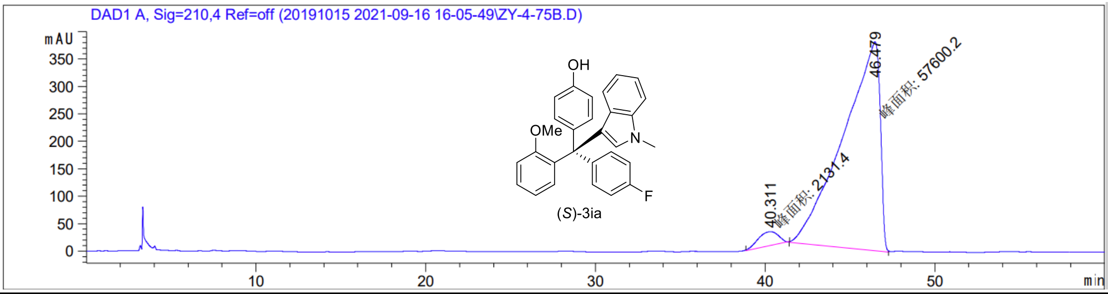

Peak	RetTime[min]	Type	Width[min]	Area[mAu*s]	Height[mAU]	Area[%]
1	40.311	MM	1.0168	2131.40112	25.06443	3.5683
2	46.479	MM	2.5198	5.76002e4	380.97815	96.4317

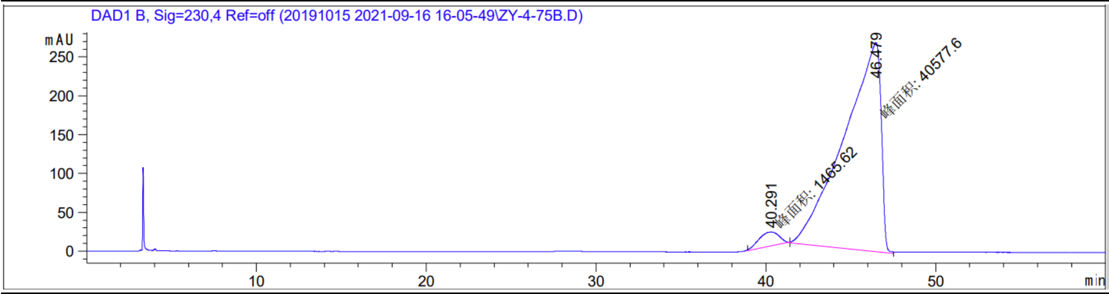

Peak	RetTime[min]	Type	Width[min]	Area[mAu*s]	Height[mAU]	Area[%]
1	40.291	MM	0.9859	1465.62463	17.67156	3.4860
2	46.479	MM	2.5150	4.05776e4	268.90729	96.5140

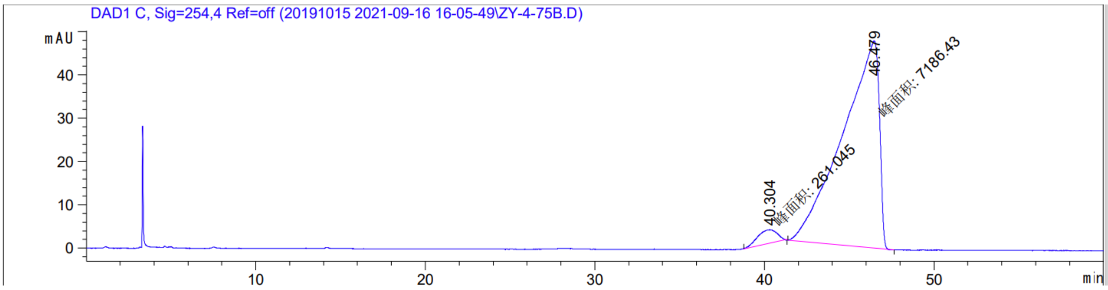

Peak	RetTime[min]	Type	Width[min]	Area[mAu*s]	Height[mAU]	Area[%]
1	40.304	MM	0.9829	261.04474	3.13666	3.5051
2	46.479	MM	2.5043	7186.42871	47.82808	96.4949

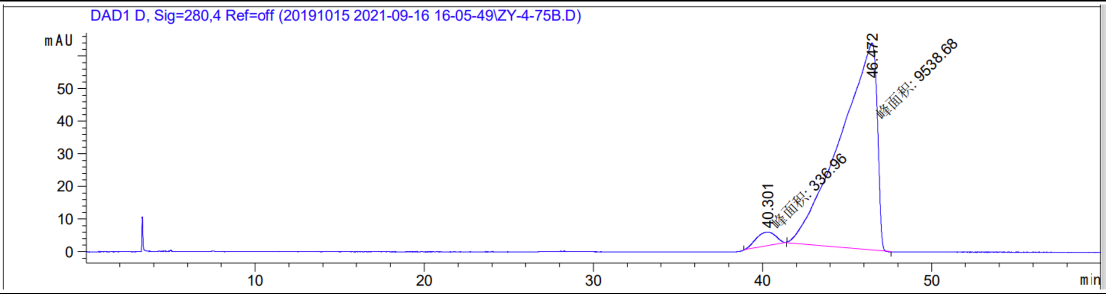

Peak	RetTime[min]	Type	Width[min]	Area[mAu*s]	Height[mAU]	Area[%]
1	40.301	MM	1.3541	336.96024	4.14742	3.4120
2	MM	MM	2.5063	9538.67871	63.43010	96.5880

==

End of Report

Sample Name: ZY-4-75B-Rac

HPLC Condition: AD-H, *n*-Hexane/*i*PrOH = 98:2, 1.0 mL/min

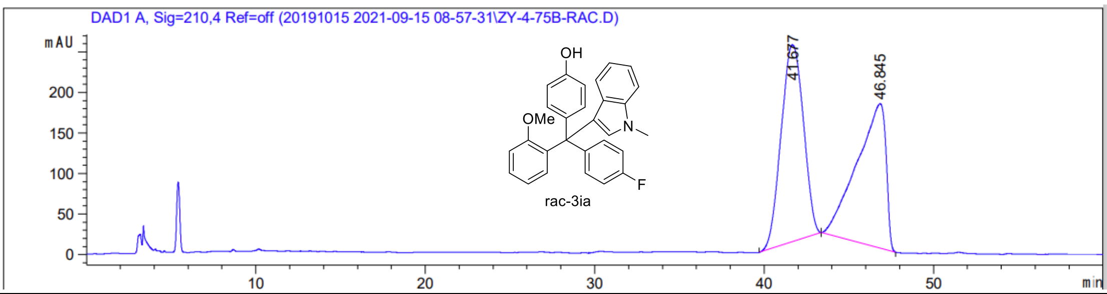

Peak	RetTime[min]	Type	Width[min]	Area[mAu*s]	Height[mAU]	Area[%]
1	41.677	BB	1.3780	2.22887e4	243.03020	51.3328
2	46.845	BB	1.5600	2.11313e4	178.13272	48.6672

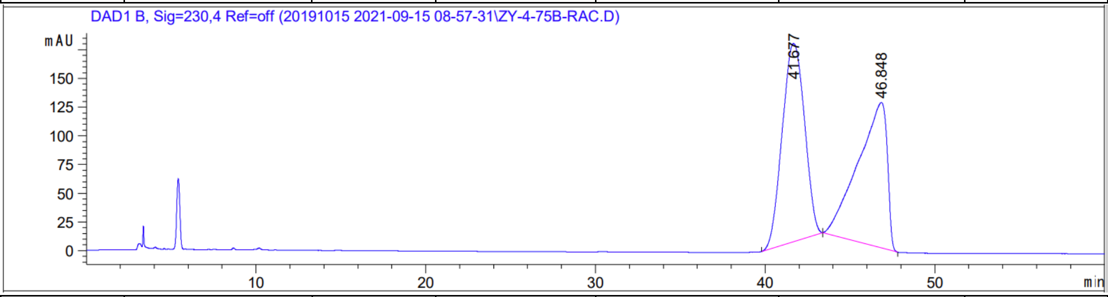

Peak	RetTime[min]	Type	Width[min]	Area[mAu*s]	Height[mAU]	Area[%]
1	41.677	BB	1.3833	1.57856e4	172.55721	51.2542
2	46.848	BB	1.6008	1.50130e4	126.66170	48.7458

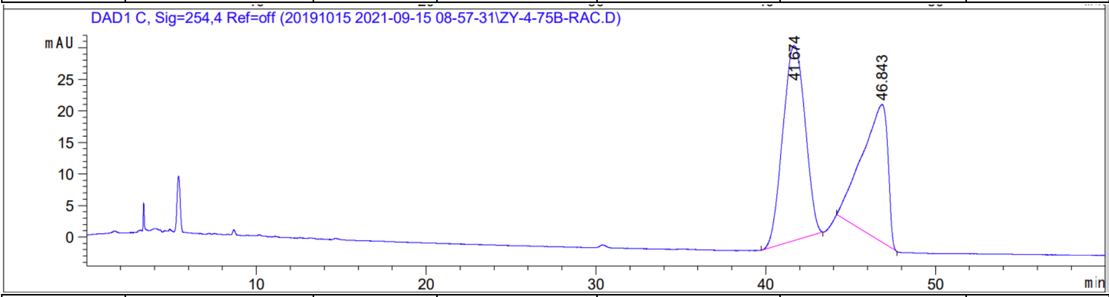

Peak	RetTime[min]	Type	Width[min]	Area[mAu*s]	Height[mAU]	Area[%]
1	41.674	BB	1.1059	2822.26025	30.88976	55.5400
2	46.843	BB	1.3601	2259.23242	21.81837	44.4600

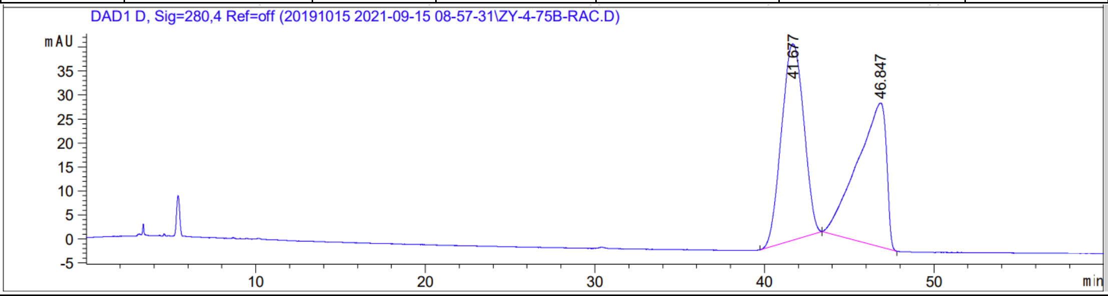

Peak	RetTime[min]	Type	Width[min]	Area[mAu*s]	Height[mAU]	Area[%]
1	41.677	BB	1.0845	3737.63550	40.88809	51.2423
2	46.847	BB	1.5468	3556.41089	30.00589	48.7577

==

End of Report

Sample Name: ZY-4-75C-OP

HPLC Condition: IC, *n*-Hexane/*i*PrOH = 98.5:1.5, 0.5 mL/min

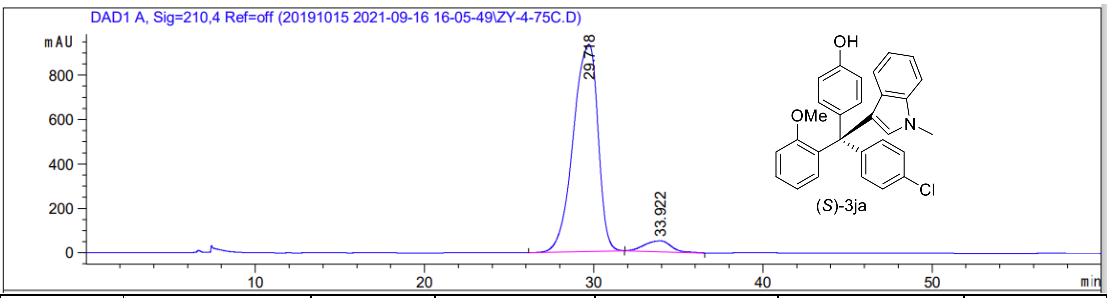

Peak	RetTime[min]	Type	Width[min]	Area[mAu*s]	Height[mAU]	Area[%]
1	29.718	BB	1.4288	9.45272e4	932.39514	94.7611
2	33.922	BB	1.3015	5225.99316	48.56101	5.2389

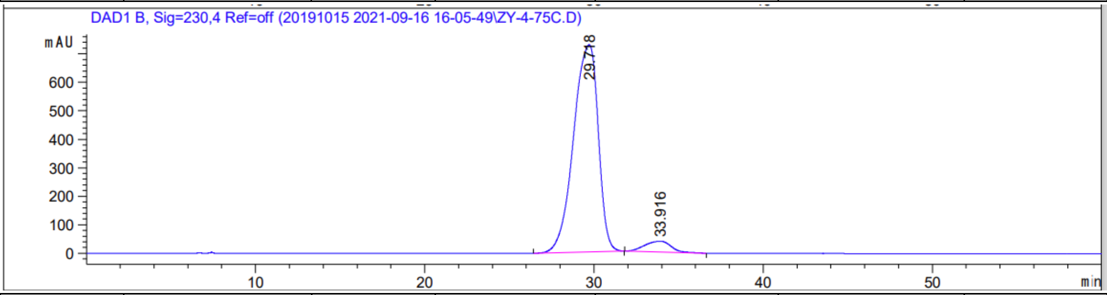

Peak	RetTime[min]	Type	Width[min]	Area[mAu*s]	Height[mAU]	Area[%]
1	29.718	BB	1.5839	7.37048e4	727.57288	94.7680
2	33.916	BB	1.4035	4069.15283	37.87369	5.2320

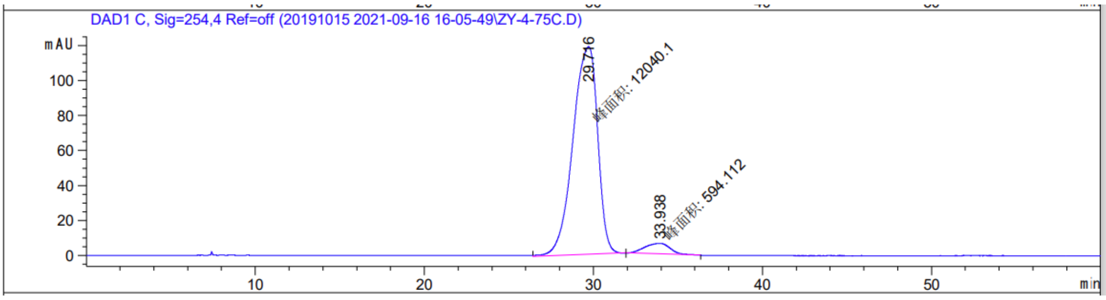

Peak	RetTime[min]	Type	Width[min]	Area[mAu*s]	Height[mAU]	Area[%]
1	29.716	MM	1.6904	1.20401e4	118.71275	95.2976
2	33.938	MM	1.6819	594.11237	5.88736	4.7024

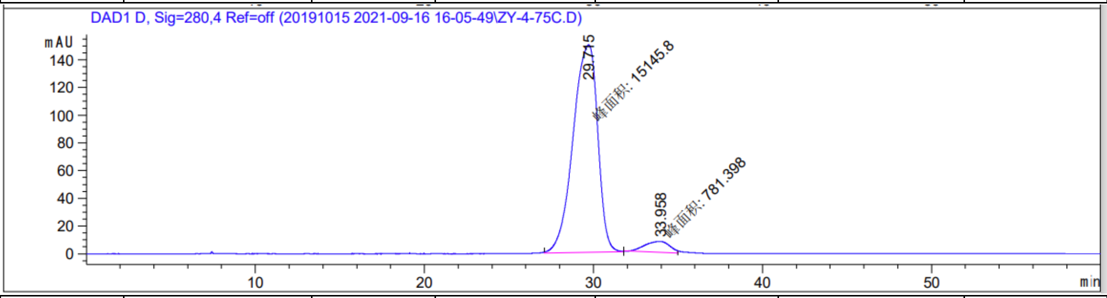

Peak	RetTime[min]	Type	Width[min]	Area[mAu*s]	Height[mAU]	Area[%]
1	29.715	MM	1.6854	1.51458e4	149.77002	95.0939
2	33.958	MM	1.6812	781.39783	7.74645	4.9061

==

End of Report

Sample Name: ZY-4-75C-Rac

HPLC Condition: IC, *n*-Hexane/*i*PrOH = 98.5:1.5, 0.5 mL/min

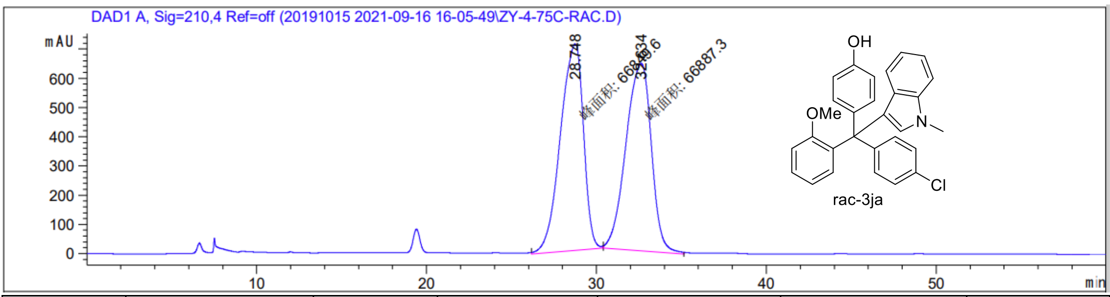

Peak	RetTime[min]	Type	Width[min]	Area[mAu*s]	Height[mAU]	Area[%]
1	28.748	MM	1.5763	6.68496e4	6.68496e4	49.9859
2	32.634	MM	1.7363	6.68873e4	642.06195	50.0141

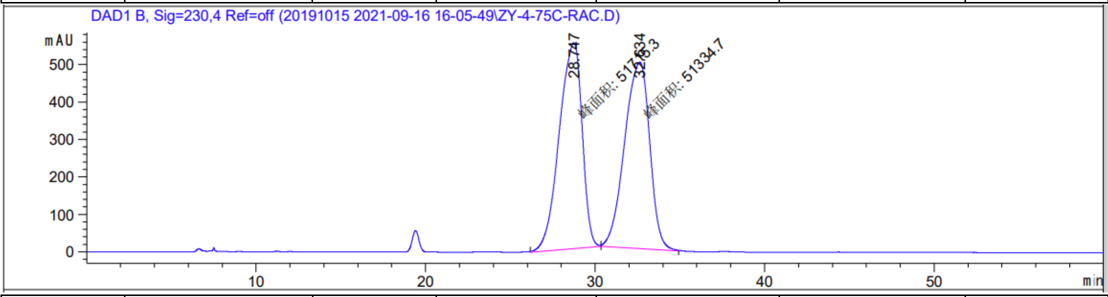

Peak	RetTime[min]	Type	Width[min]	Area[mAu*s]	Height[mAU]	Area[%]
1	28.747	MM	1.5673	5.17153e4	549.95337	50.1847
2	32.634	MM	1.7177	5.13347e4	498.10614	49.8153

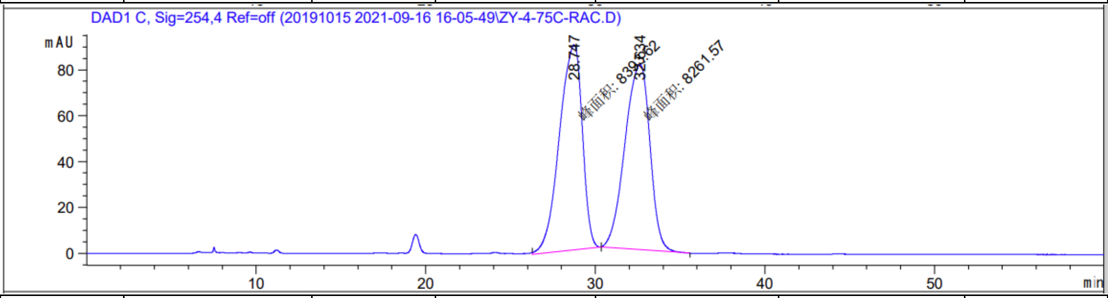

Peak	RetTime[min]	Type	Width[min]	Area[mAu*s]	Height[mAU]	Area[%]
1	28.747	MM	1.5637	8393.62207	89.46172	50.3964
2	32.634	MM	1.7041	8261.57129	80.79900	49.6036

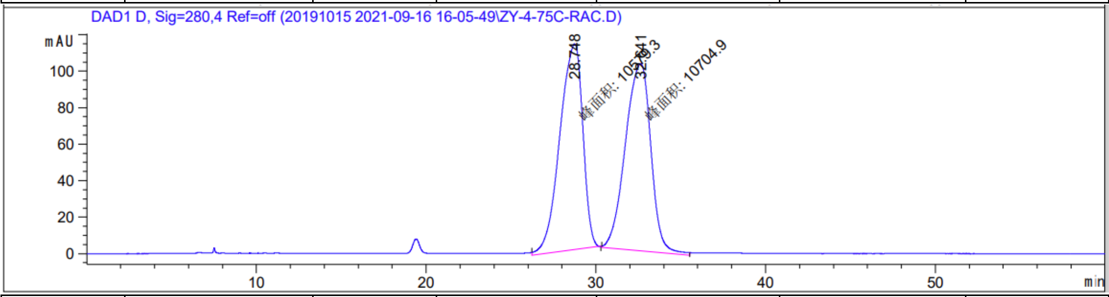

Peak	RetTime[min]	Type	Width[min]	Area[mAu*s]	Height[mAU]	Area[%]
1	28.748	MM	1.5661	1.05793e4	112.58551	49.7050
2	32.641	MM	1.7376	1.07049e4	102.67993	50.2950

==

End of Report

Sample Name: ZY-4-75E-OP

HPLC Condition: AD-H, *n*-Hexane/*i*PrOH = 98:2, 1.0 mL/min

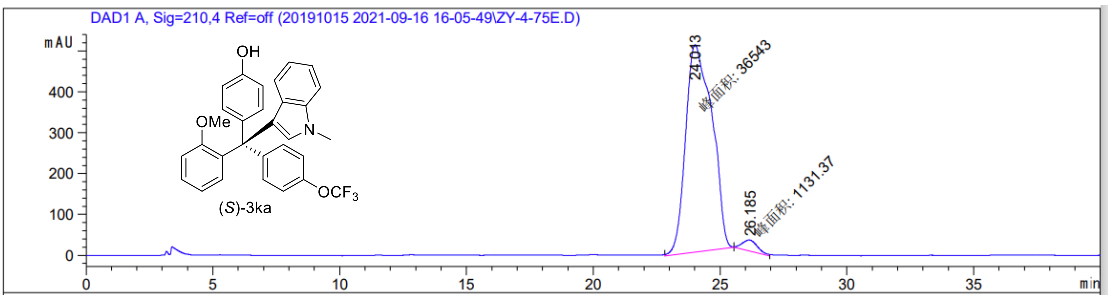

Peak	RetTime[min]	Type	Width[min]	Area[mAu*s]	Height[mAU]	Area[%]
1	24.013	MM	1.2008	3.65430e4	507.18692	96.9970
2	26.185	MM	0.6990	1131.36523	26.97536	3.0030

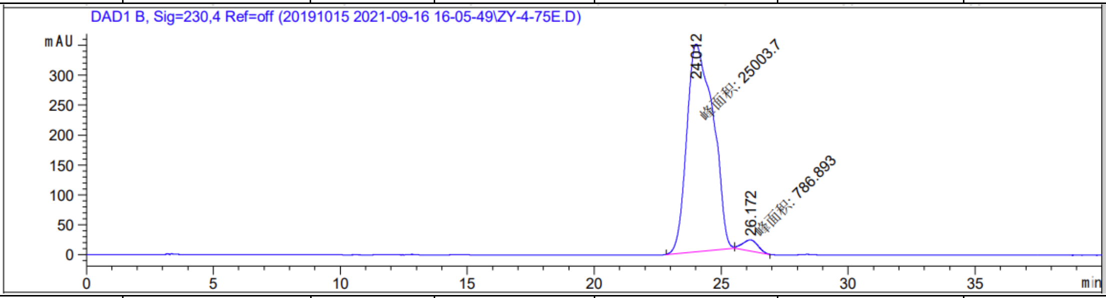

Peak	RetTime[min]	Type	Width[min]	Area[mAu*s]	Height[mAU]	Area[%]
1	24.012	MM	1.2001	2.50037e4	347.23965	96.9489
2	26.172	MM	0.7008	786.89301	18.71300	3.0511

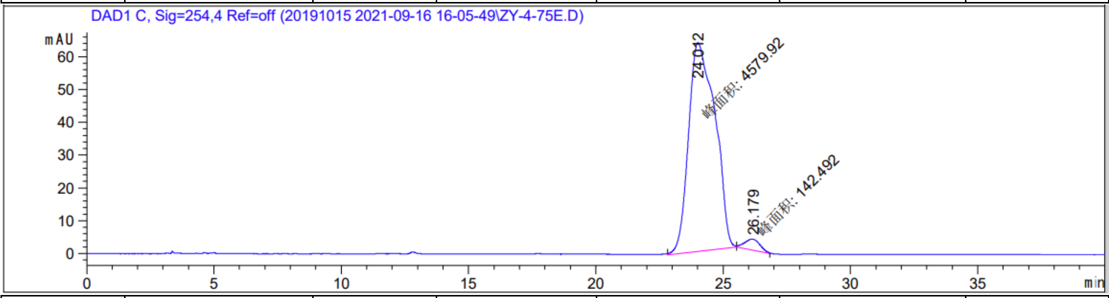

Peak	RetTime[min]	Type	Width[min]	Area[mAu*s]	Height[mAU]	Area[%]
1	24.012	MM	1.2011	4579.92188	63.55087	96.9826
2	26.179	MM	0.6991	142.49182	3.39689	3.0174

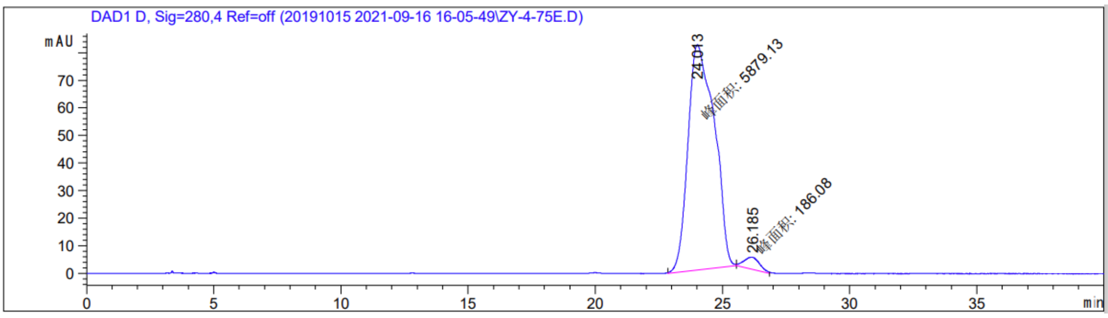

Peak	RetTime[min]	Type	Width[min]	Area[mAu*s]	Height[mAU]	Area[%]
1	24.013	MM	1.1995	5879.12744	81.69178	96.9320
2	26.185	MM	0.7057	186.08049	4.39475	3.0680

==

End of Report

Sample Name: ZY-4-75E-Rac

HPLC Condition: AD-H, *n*-Hexane/*i*PrOH = 98:2, 1.0 mL/min

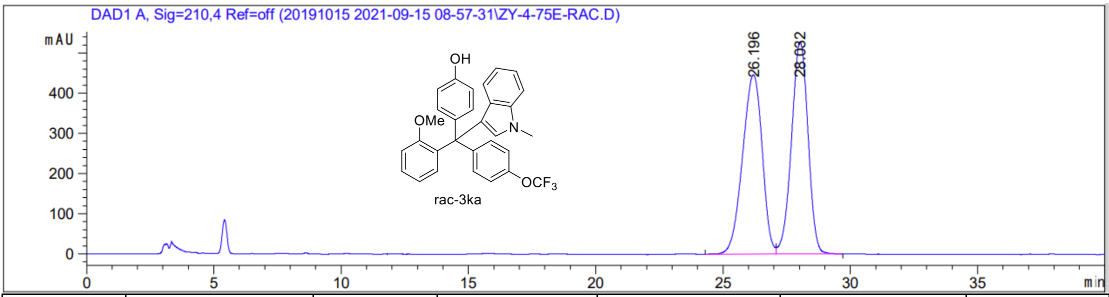

Peak	RetTime[min]	Type	Width[min]	Area[mAu*s]	Height[mAU]	Area[%]
1	26.196	BV	0.8521	2.39373e4	446.03677	50.1222
2	28.032	VB	0.7073	2.38205e4	527.05115	49.8778

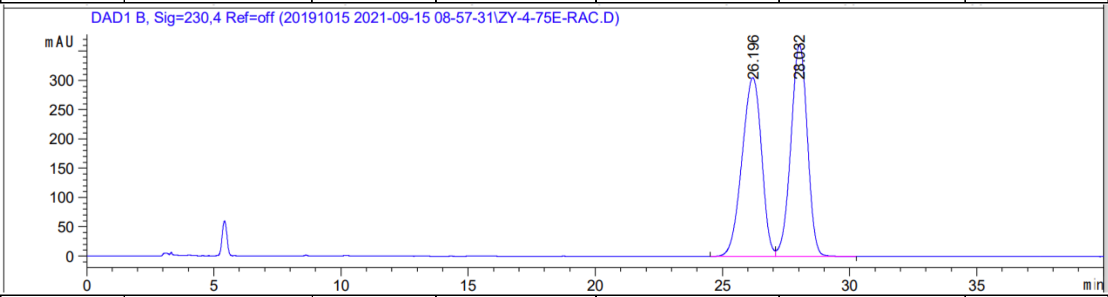

Peak	RetTime[min]	Type	Width[min]	Area[mAu*s]	Height[mAU]	Area[%]
1	26.196	BV	0.8540	1.63432e4	305.56204	50.0226
2	28.032	VB	0.7066	1.63284e4	361.72641	49.9774

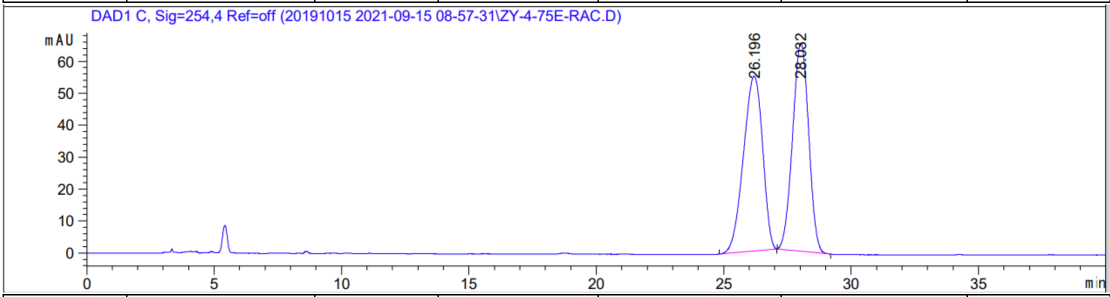

Peak	RetTime[min]	Type	Width[min]	Area[mAu*s]	Height[mAU]	Area[%]
1	26.196	BB	0.8272	2858.99390	54.75016	49.9910
2	28.032	BB	0.6905	2860.01978	65.10296	50.0090

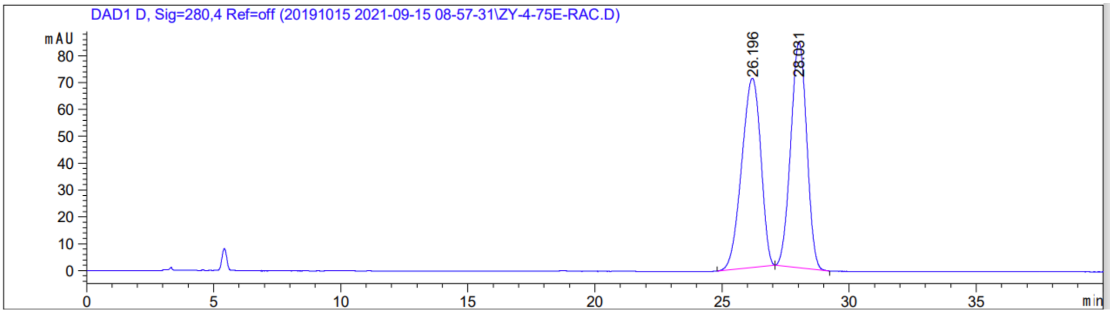

Peak	RetTime[min]	Type	Width[min]	Area[mAu*s]	Height[mAU]	Area[%]
1	26.196	BB	0.8309	3677.23340	70.46198	49.9978
2	28.031	BB	0.6859	3677.55469	83.81761	50.0022

==

End of Report

Sample Name: ZY-4-93C-OP

HPLC Condition: AD-H, *n*-Hexane/*i*PrOH = 95:5, 1.0 mL/min

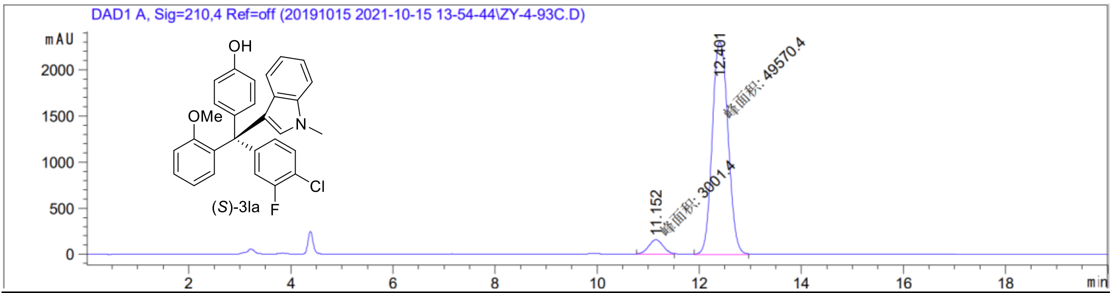

Peak	RetTime[min]	Type	Width[min]	Area[mAu*s]	Height[mAU]	Area[%]
1	11.152	MM	0.3263	3001.40137	153.32727	5.7091
2	12.401	MM	0.3585	4.95704e4	2304.64404	94.2909

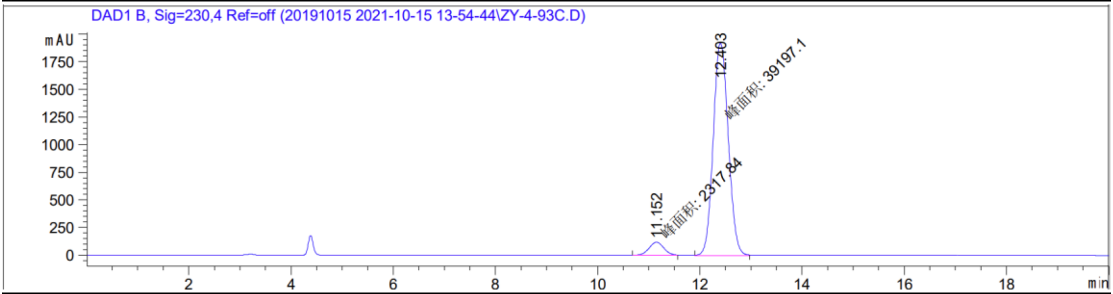

Peak	RetTime[min]	Type	Width[min]	Area[mAu*s]	Height[mAU]	Area[%]
1	11.152	MM	0.3320	2317.83887	116.36661	5.5831
2	12.403	MM	0.3403	3.91971e4	1919.96179	94.4169

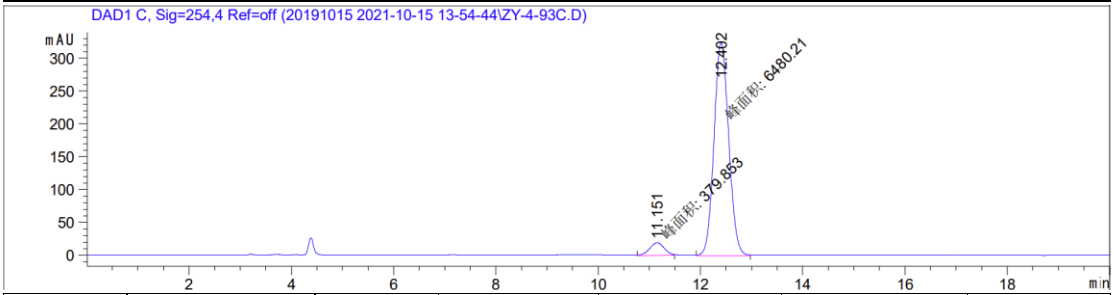

Peak	RetTime[min]	Type	Width[min]	Area[mAu*s]	Height[mAU]	Area[%]
1	11.151	MM	0.3339	379.85266	18.96170	5.5372
2	12.402	MM	0.3333	6480.21240	324.06094	94.4628

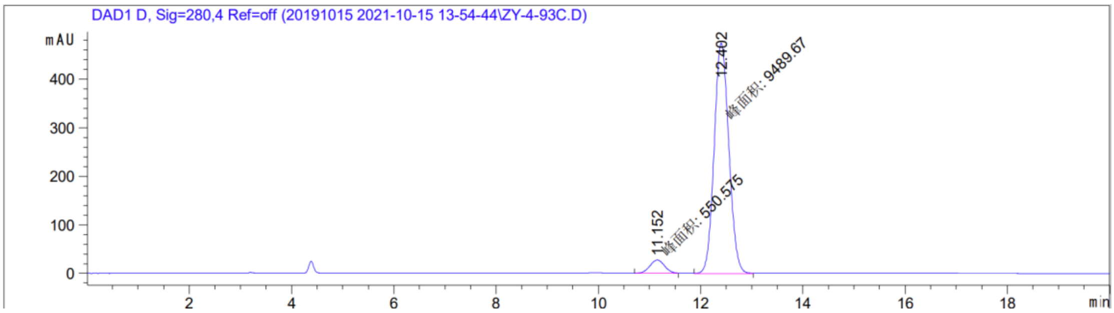

Peak	RetTime[min]	Type	Width[min]	Area[mAu*s]	Height[mAU]	Area[%]
1	11.152	MM	0.3336	550.57471	27.50974	5.4837
2	12.402	MM	0.3331	9489.67383	474.80502	94.5163

==

End of Report

Sample Name: ZY-4-93C-Rac

HPLC Condition: AD-H, *n*-Hexane/*i*PrOH = 95:5, 1.0 mL/min

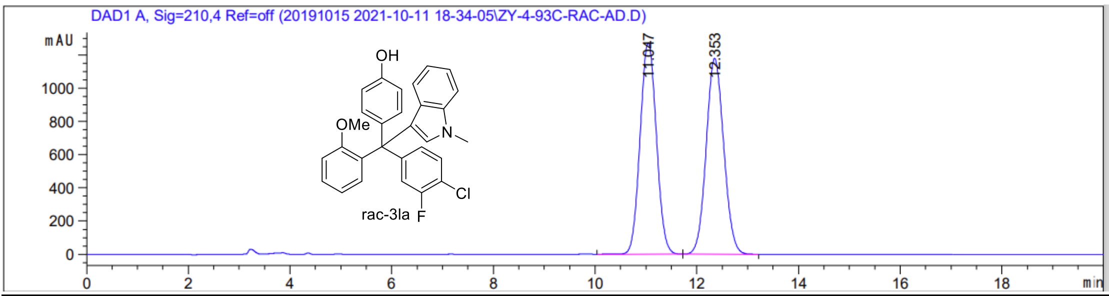

Peak	RetTime[min]	Type	Width[min]	Area[mAu*s]	Height[mAU]	Area[%]
1	11.047	BB	0.3519	2.83644e4	1273.32776	50.0122
2	12.353	BB	0.3742	2.83505e4	1180.51855	49.9878

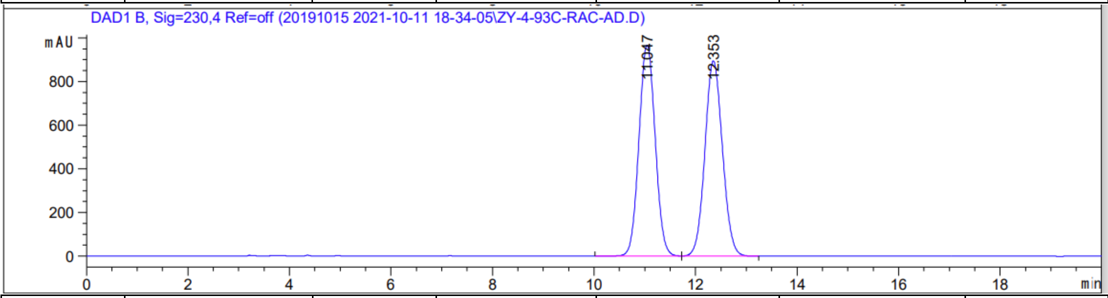

Peak	RetTime[min]	Type	Width[min]	Area[mAu*s]	Height[mAU]	Area[%]
1	11.047	BB	0.3494	2.14912e4	966.55695	50.0805
2	12.353	BB	0.3734	2.14221e4	894.51526	49.9195

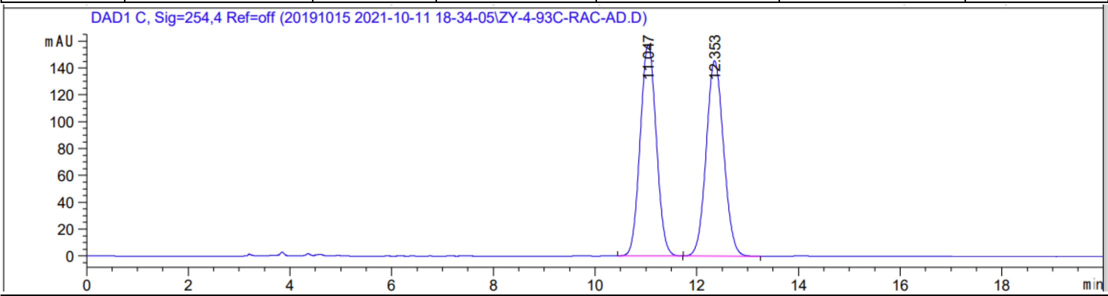

Peak	RetTime[min]	Type	Width[min]	Area[mAu*s]	Height[mAU]	Area[%]
1	11.047	BB	0.3473	3469.88843	157.33768	49.9513
2	12.353	BB	0.3686	3476.65747	145.61293	50.0487

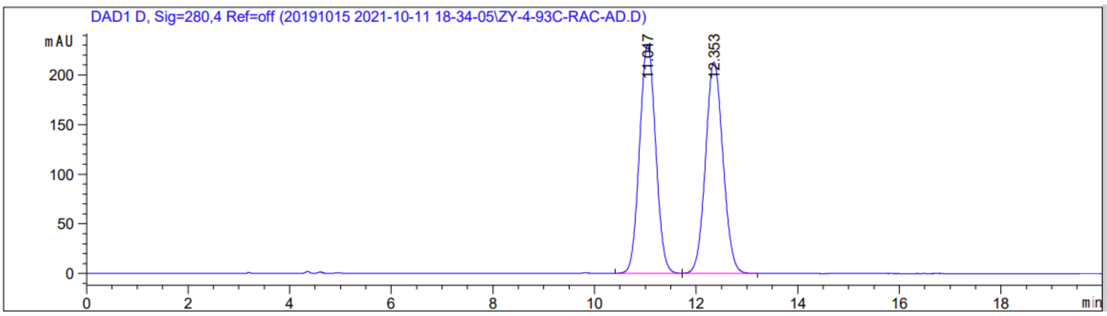

Peak	RetTime[min]	Type	Width[min]	Area[mAu*s]	Height[mAU]	Area[%]
1	11.047	BB	0.3474	5080.34131	230.22318	50.0058
2	12.353	12.353	0.3703	5079.16016	212.94879	49.9942

==

End of Report

Sample Name: ZY-4-93D-OP

HPLC Condition: AD-H, *n*-Hexane/*i*PrOH = 95:5, 1.0 mL/min

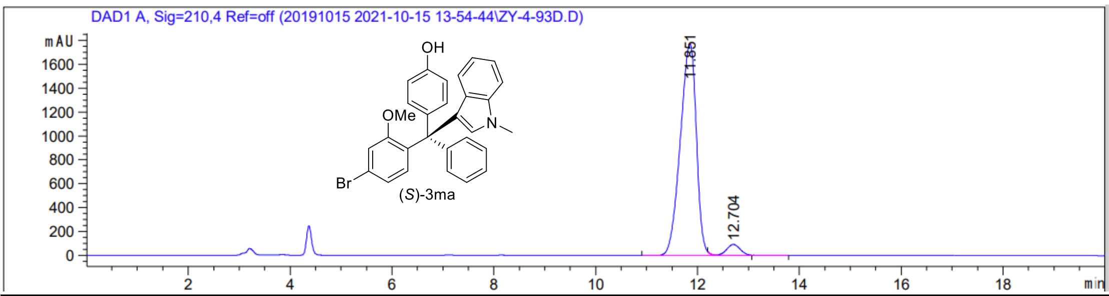

Peak	RetTime[min]	Type	Width[min]	Area[mAu*s]	Height[mAU]	Area[%]
1	11.851	BV R	0.3456	3.88728e4	1772.81091	95.6891
2	12.704	VV E	0.2939	1751.27380	92.92149	4.3109

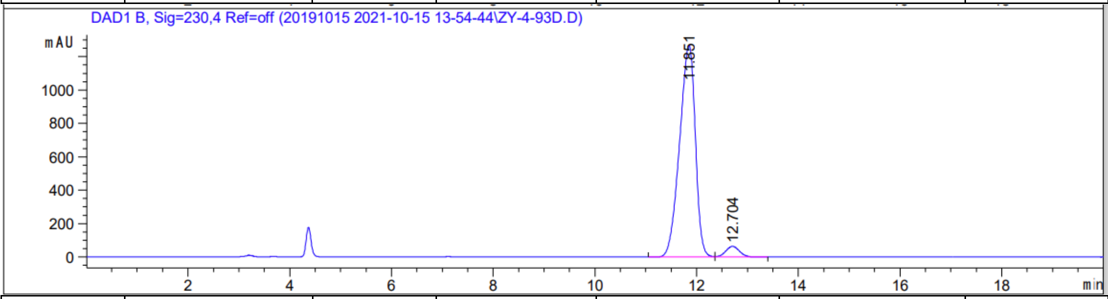

Peak	RetTime[min]	Type	Width[min]	Area[mAu*s]	Height[mAU]	Area[%]
1	11.851	BV	0.3347	2.72823e4	1269.39417	95.6943
2	12.704	VB	0.2946	1227.55579	64.34969	4.3057

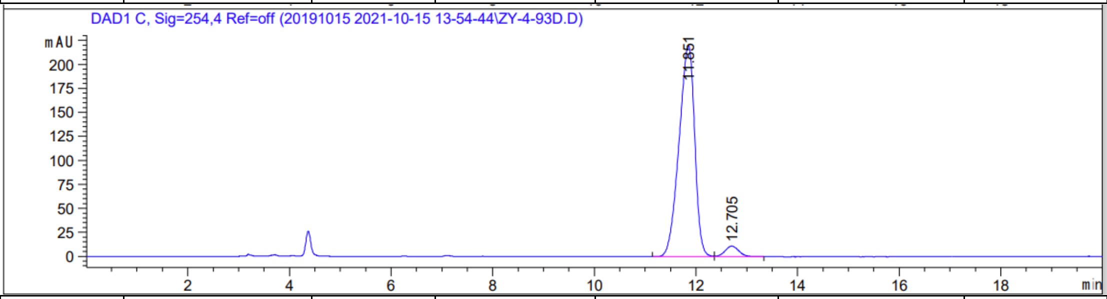

Peak	RetTime[min]	Type	Width[min]	Area[mAu*s]	Height[mAU]	Area[%]
1	11.851	BV	0.3337	4712.38379	220.16031	95.6936
2	12.705	VB	0.2959	212.06885	11.05147	4.3064

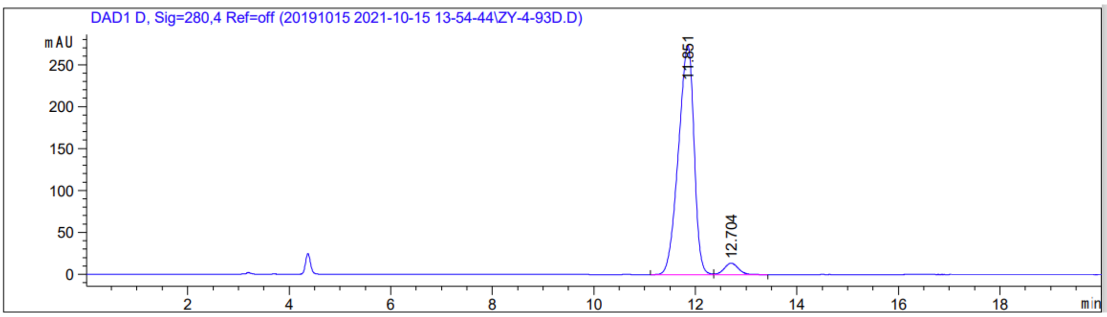

Peak	RetTime[min]	Type	Width[min]	Area[mAu*s]	Height[mAU]	Area[%]
1	11.851	BV	0.3334	5833.76416	272.93802	95.7186
2	12.704	VB	0.3010	260.93964	13.65355	4.2814

==

End of Report

Sample Name: ZY-4-93D-Rac

HPLC Condition: AD-H, *n*-Hexane/*i*PrOH = 95:5, 1.0 mL/min

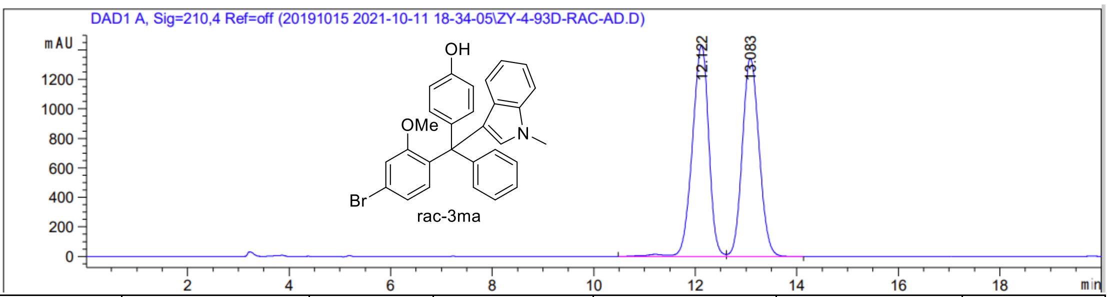

Peak	RetTime[min]	Type	Width[min]	Area[mAu*s]	Height[mAU]	Area[%]
1	12.122	VV R	0.3474	3.21770e4	1429.45740	51.0192
2	13.083	VB	0.3602	3.08914e4	1343.63062	48.9808

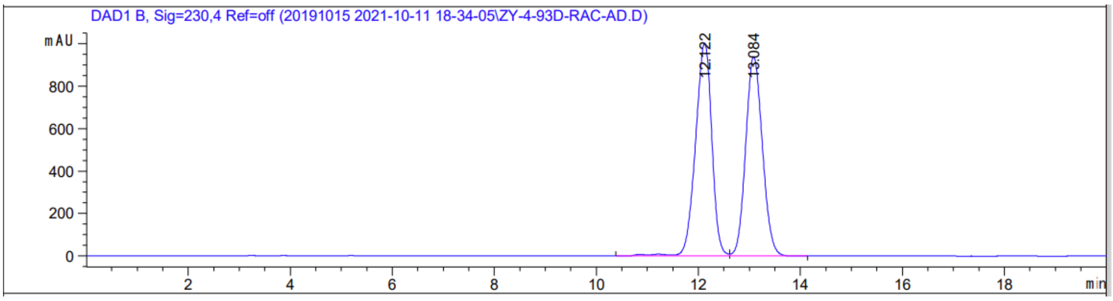

Peak	RetTime[min]	Type	Width[min]	Area[mAu*s]	Height[mAU]	Area[%]
1	12.122	VV R	0.3412	2.23813e4	1001.10120	51.0814
2	13.084	VB	0.3564	2.14337e4	938.78943	48.9186

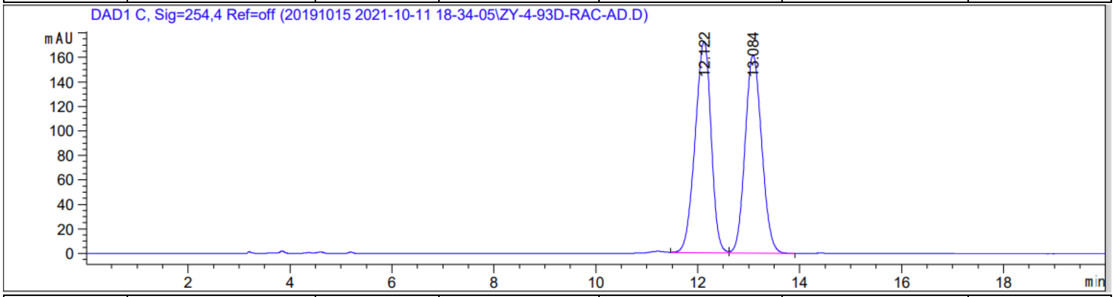

Peak	RetTime[min]	Type	Width[min]	Area[mAu*s]	Height[mAU]	Area[%]
1	12.122	BV	0.3391	3764.80347	172.16327	50.6216
2	13.084	VB	0.3550	3672.34351	161.67903	49.3784

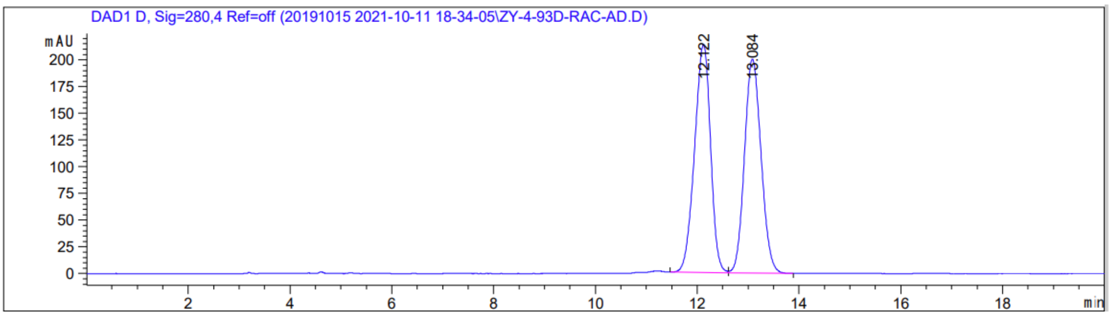

Peak	RetTime[min]	Type	Width[min]	Area[mAu*s]	Height[mAU]	Area[%]
1	12.122	BV	0.3388	4657.31689	213.27629	50.6255
2	13.084	VB	0.3546	4542.23242	200.25867	49.3745

==

End of Report

Sample Name: ZY-4-93E-OP

HPLC Condition: AD-H, *n*-Hexane/*i*PrOH = 95:5, 1.0 mL/min

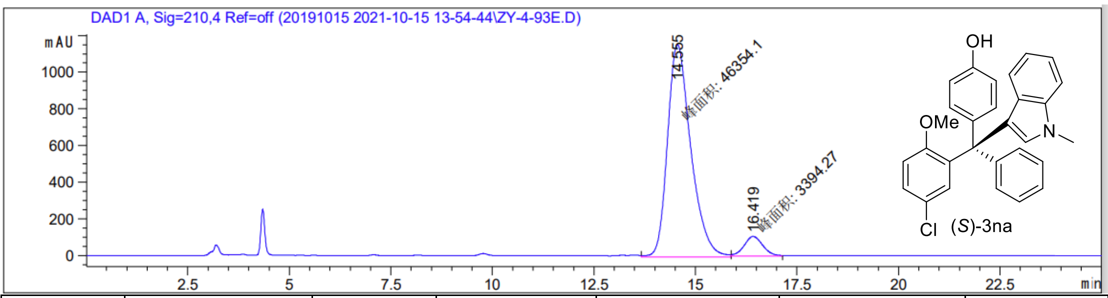

Peak	RetTime[min]	Type	Width[min]	Area[mAu*s]	Height[mAU]	Area[%]
1	14.555	MM	0.6668	4.63541e4	1158.64441	93.1771
2	16.419	MM	0.5305	3394.26563	106.64064	6.8229

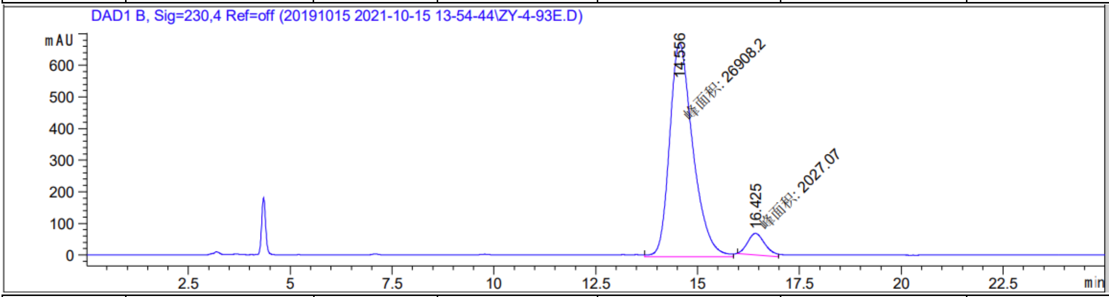

Peak	RetTime[min]	Type	Width[min]	Area[mAu*s]	Height[mAU]	Area[%]
1	14.556	MM	0.6644	2.69082e4	674.96979	92.9945
2	16.425	MM	0.4957	2027.07446	68.15989	7.0055

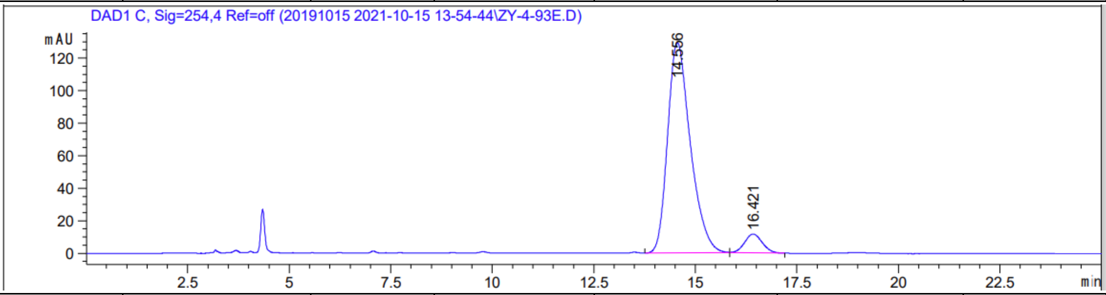

Peak	RetTime[min]	Type	Width[min]	Area[mAu*s]	Height[mAU]	Area[%]
1	14.556	BB	0.5876	5021.31934	129.15067	93.6025
2	16.421	BB	0.4525	343.19333	11.49038	6.3975

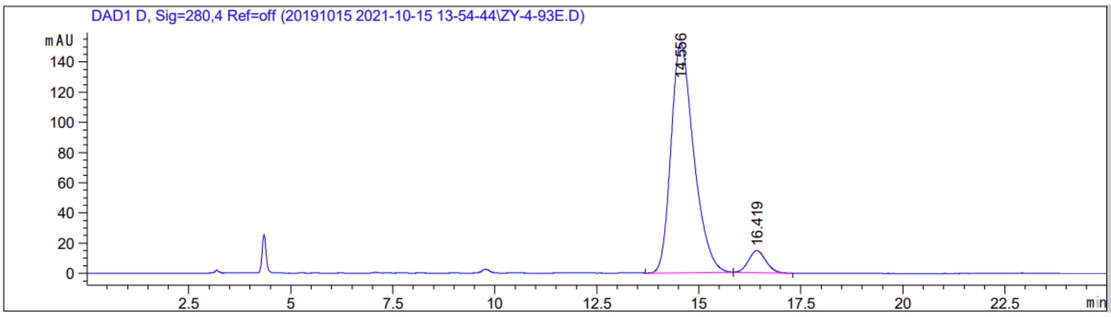

Peak	RetTime[min]	Type	Width[min]	Area[mAu*s]	Height[mAU]	Area[%]
1	14.556	BB	0.5853	5889.01953	151.57664	93.2724
2	16.419	BB	0.4482	424.76770	14.56732	6.7276

==

End of Report

Sample Name: ZY-4-93E-Rac

HPLC Condition: AD-H, *n*-Hexane/*i*PrOH = 95:5, 1.0 mL/min

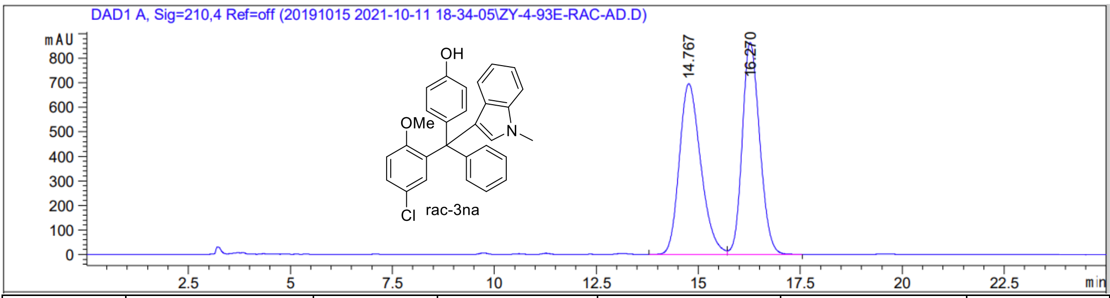

Peak	RetTime[min]	Type	Width[min]	Area[mAu*s]	Height[mAU]	Area[%]
1	14.767	BV	0.5637	2.55240e4	696.31152	49.8646
2	16.270	VB	0.4621	2.56626e4	865.17969	50.1354

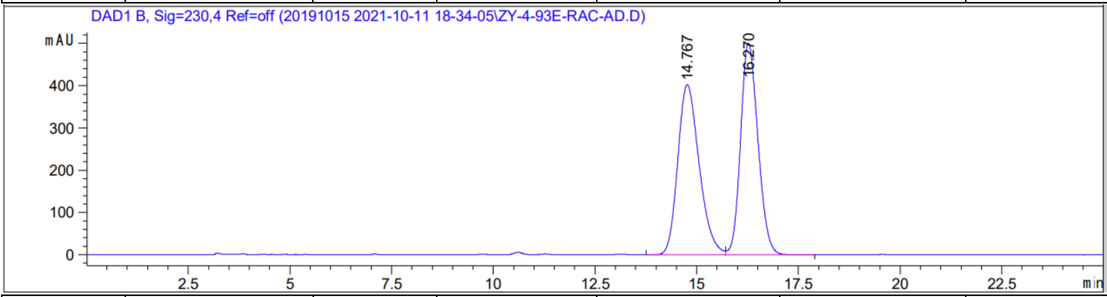

Peak	RetTime[min]	Type	Width[min]	Area[mAu*s]	Height[mAU]	Area[%]
1	14.767	BV	0.5634	1.47497e4	402.63324	49.8089
2	16.270	VB	0.4598	1.48629e4	501.38202	50.1911

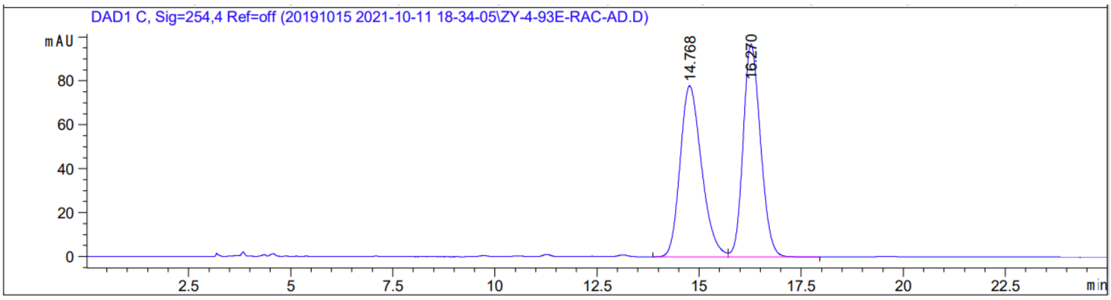

Peak	RetTime[min]	Type	Width[min]	Area[mAu*s]	Height[mAU]	Area[%]
1	14.768	BV	0.5613	2850.12012	77.82130	49.8286
2	16.270	VB	0.4598	2869.72217	96.80745	50.1714

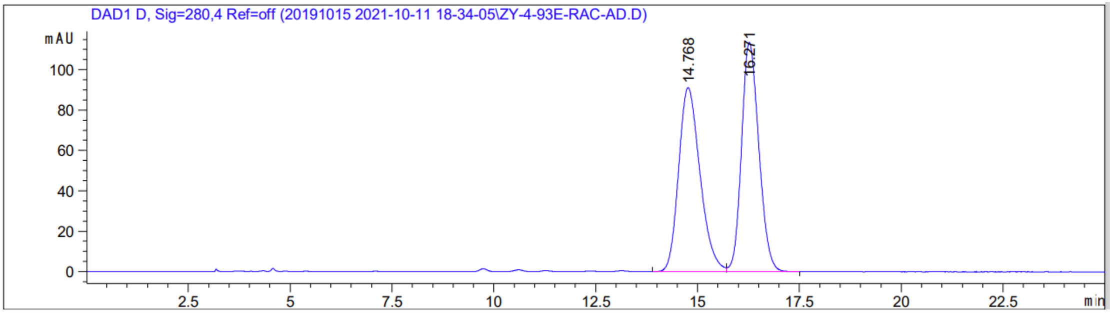

Peak	RetTime[min]	Type	Width[min]	Area[mAu*s]	Height[mAU]	Area[%]
1	14.768	BV	0.5607	3331.79419	91.10322	49.8137
2	16.271	VB	0.4612	3356.70898	113.44799	50.1863

==

End of Report

Sample Name: ZY-4-93F-OP

HPLC Condition: AD-H, *n*-Hexane/*i*PrOH = 97:3, 1.0 mL/min

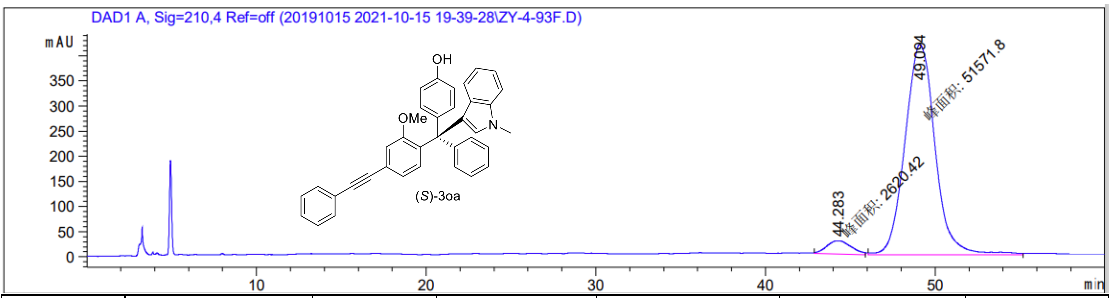

Peak	RetTime[min]	Type	Width[min]	Area[mAu*s]	Height[mAU]	Area[%]
1	44.283	MM	1.6635	2620.42212	26.25422	4.8354
2	49.094	MM	2.0563	5.15718e4	417.99088	95.1646

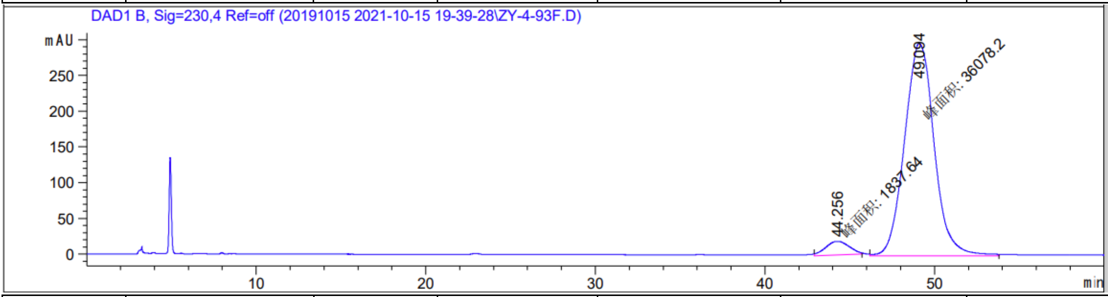

Peak	RetTime[min]	Type	Width[min]	Area[mAu*s]	Height[mAU]	Area[%]
1	44.256	MM	1.6096	1837.64453	19.02829	4.8466 2
2	49.094	MM	2.0232	3.60782e4	297.21048	95.1534

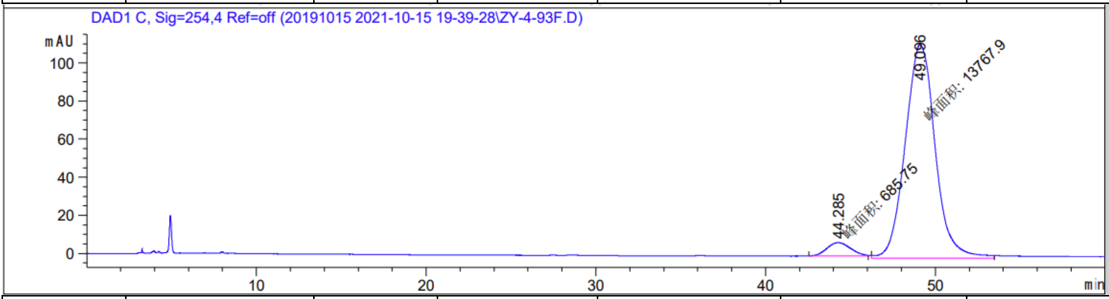

Peak	RetTime[min]	Type	Width[min]	Area[mAu*s]	Height[mAU]	Area[%]
1	44.285	MM	1.6331	685.75006	6.99834	4.7445
2	49.096	MM	2.0483	1.37679e4	112.02969	95.2555

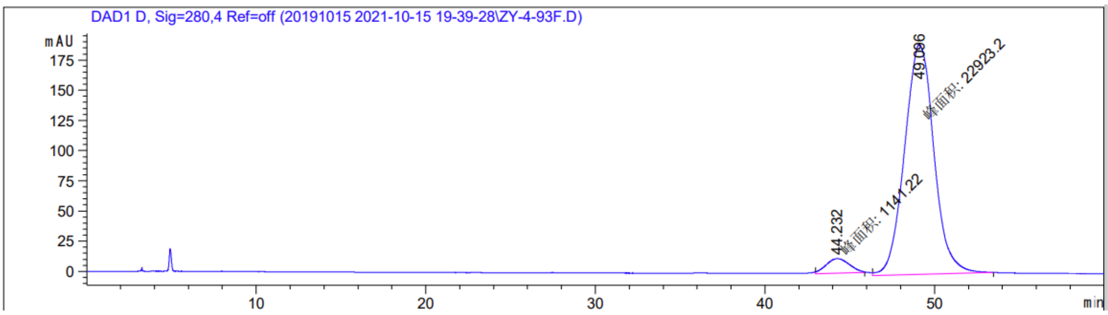

Peak	RetTime[min]	Type	Width[min]	Area[mAu*s]	Height[mAU]	Area[%]
1	44.232	MM	1.5877	1141.21704	11.97996	4.7423
2	49.096	MM	2.0100	2.29232e4	190.07974	95.2577

==

End of Report

Sample Name: ZY-4-93F-Rac

HPLC Condition: AD-H, *n*-Hexane/*i*PrOH = 97:3, 1.0 mL/min

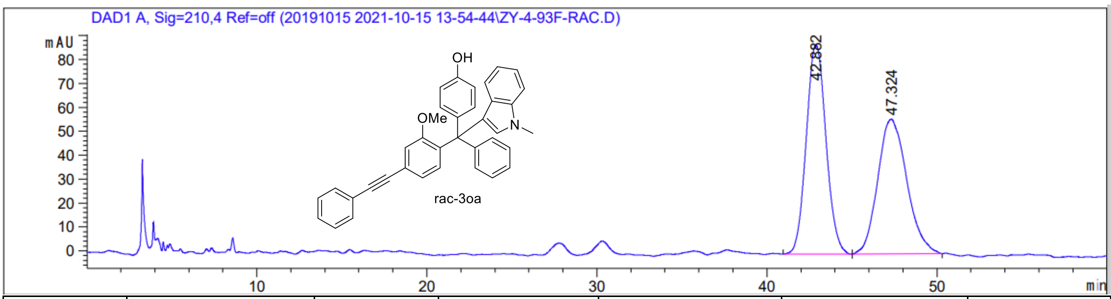

Peak	RetTime[min]	Type	Width[min]	Area[mAu*s]	Height[mAU]	Area[%]
1	42.882	BB	1.2113	7073.39600	87.47920	51.3039
2	47.324	BB	1.4484	6713.85156	56.33503	48.6961

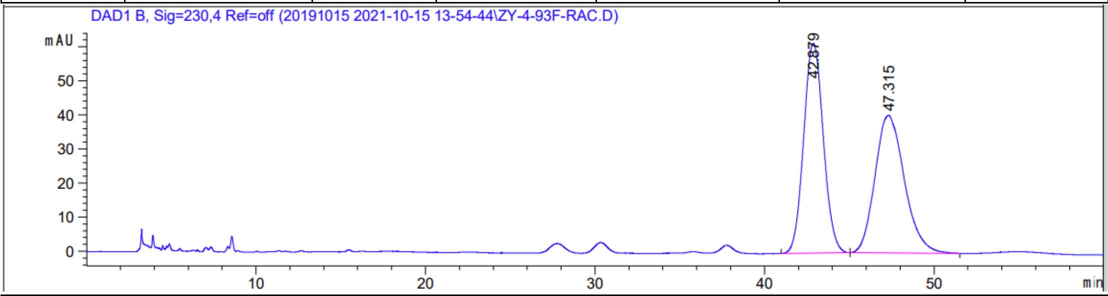

Peak	RetTime[min]	Type	Width[min]	Area[mAu*s]	Height[mAU]	Area[%]
1	42.879	BB	1.1692	4960.22607	61.61326	50.4474
2	47.315	BB	1.4842	4872.23828	40.42223	49.5526

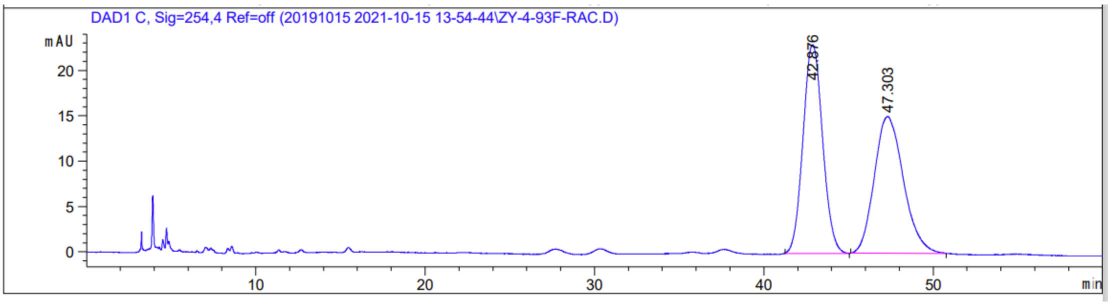

Peak	RetTime[min]	Type	Width[min]	Area[mAu*s]	Height[mAU]	Area[%]
1	42.876	BB	1.0940	1845.96814	23.00793	50.5338
2	47.303	BB	1.4180	1806.96924	15.07939	49.4662

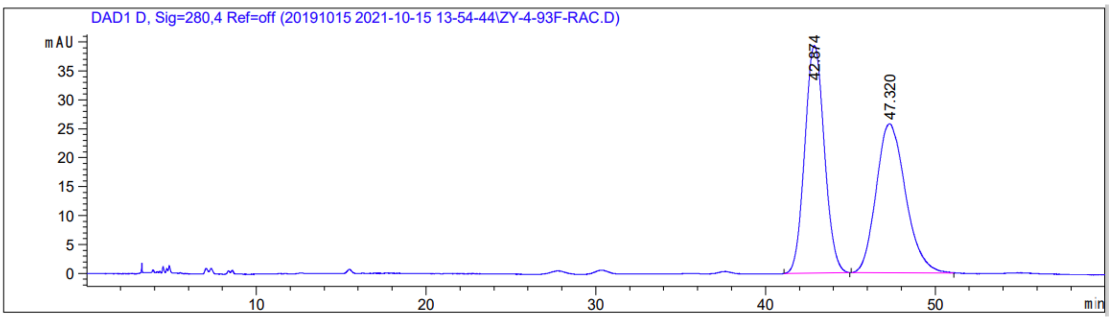

Peak	RetTime[min]	Type	Width[min]	Area[mAu*s]	Height[mAU]	Area[%]
1	42.874	BB	1.1885	3163.09839	39.31950	50.4783
2	47.320	BB	1.4529	3103.15234	25.76883	49.5217

==

End of Report

Sample Name: ZY-4-86C-OP

HPLC Condition:AD-H, *n*-Hexane/*i*PrOH = 95:5, 1.0 mL/min

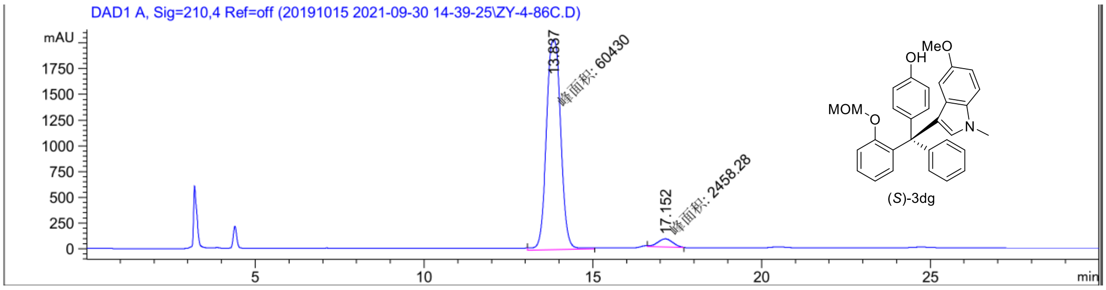

Peak	RetTime[min]	Type	Width[min]	Area[mAu*s]	Height[mAU]	Area[%]
1	13.837	MM	0.4937	6.04300e4	2040.11157	96.0910
2	17.152	MM	0.5195	2458.28223	78.86625	3.9090

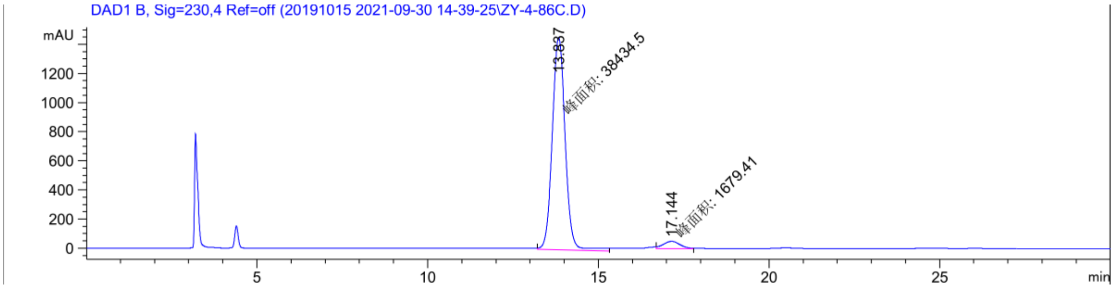

Peak	RetTime[min]	Type	Width[min]	Area[mAu*s]	Height[mAU]	Area[%]
1	13.837	MM	0.4393	3.84345e4	1458.21313	95.8134
2	17.144	MM	0.5659	1679.41260	49.46396	4.1866

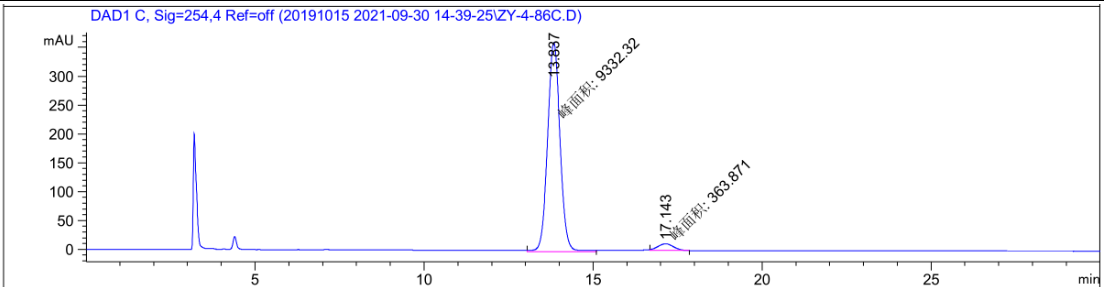

Peak	RetTime[min]	Type	Width[min]	Area[mAu*s]	Height[mAU]	Area[%]
1	13.837	MM	0.4293	9332.31543	362.34860	96.2473
2	17.143	MM	0.5339	363.87070	11.35797	3.7527

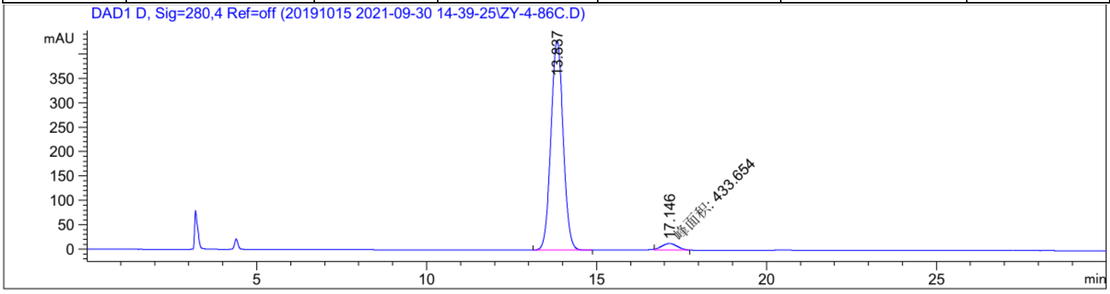

Peak	RetTime[min]	Type	Width[min]	Area[mAu*s]	Height[mAU]	Area[%]
1	13.837	BB	0.3886	1.07547e4	428.76187	96.1241
2	17.146	MM	0.5345	433.65399	13.52119	3.8759

==

End of Report

Sample Name: ZY-4-86C-Rac

HPLC Condition:AD-H, *n*-Hexane/*i*PrOH = 95:5, 1.0 mL/min

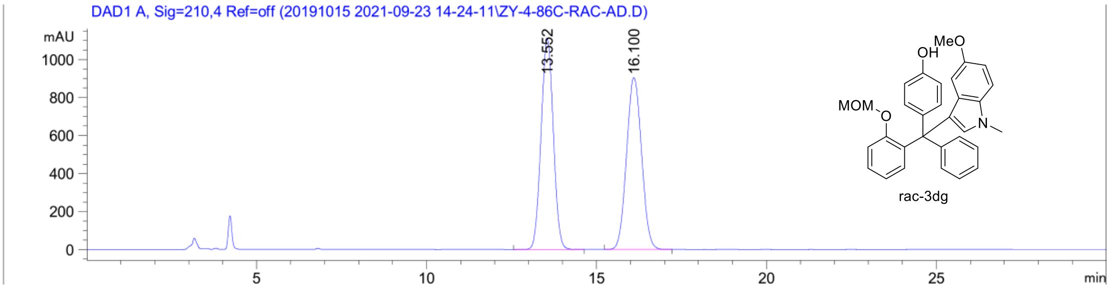

Peak	RetTime[min]	Type	Width[min]	Area[mAu*s]	Height[mAU]	Area[%]
1	13.552	BB	0.3970	2.78116e4	1107.41870	50.0165
2	16.100	BB	0.4844	2.77932e4	904.65857	49.9835

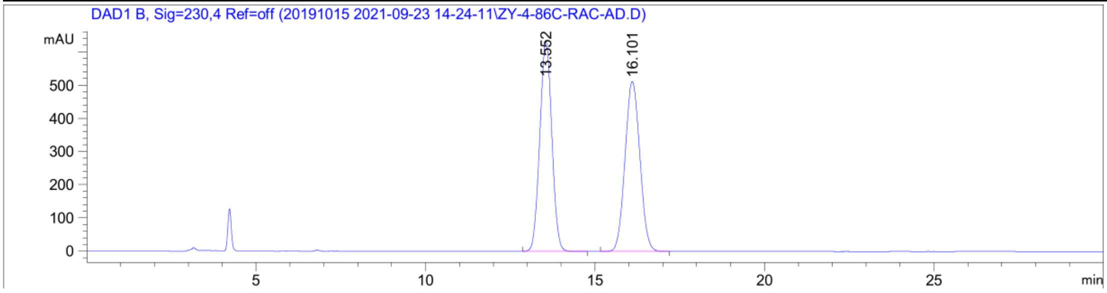

Peak	RetTime[min]	Type	Width[min]	Area[mAu*s]	Height[mAU]	Area[%]
1	13.552	BB	0.3882	1.56063e4	631.82904	50.0258
2	16.101	BB	0.4792	1.55902e4	512.06964	49.9742

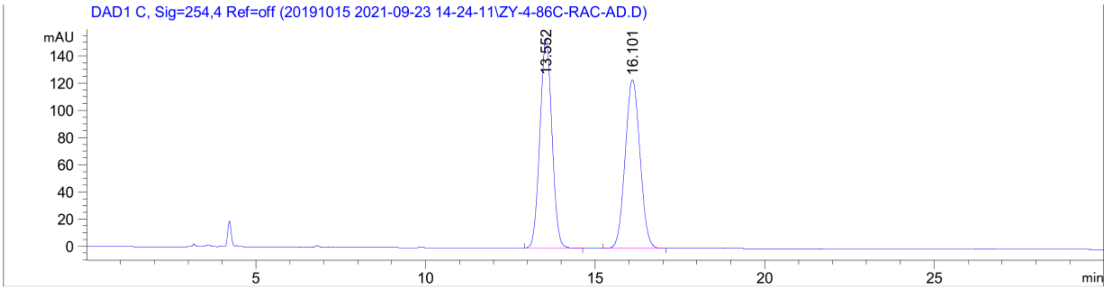

Peak	RetTime[min]	Type	Width[min]	Area[mAu*s]	Height[mAU]	Area[%]
1	13.552	BB	0.3888	3794.43579	153.28877	50.1341
2	16.101	BB	0.4769	3774.13647	124.05247	49.8659

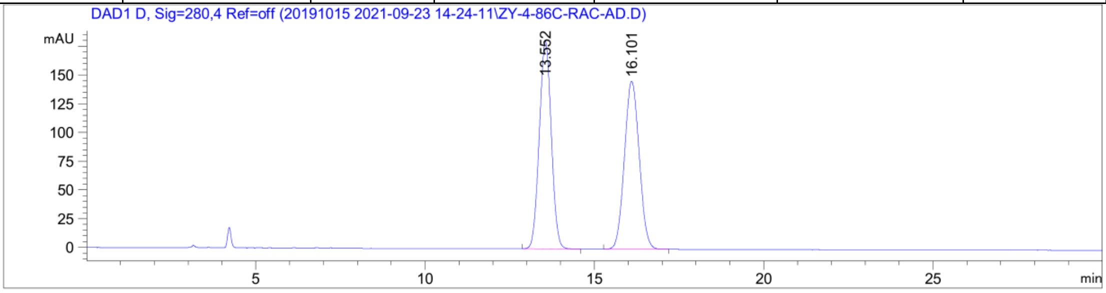

Peak	RetTime[min]	Type	Width[min]	Area[mAu*s]	Height[mAU]	Area[%]
1	13.552	BB	0.3876	4459.35205	180.92627	50.0757
2	16.101	BB	0.4784	4445.87451	146.34315	49.9243

==

End of Report

Sample Name: ZY-4-86E-OP

HPLC Condition:AD-H, *n*-Hexane/*i*PrOH = 95:5, 1.0 mL/min

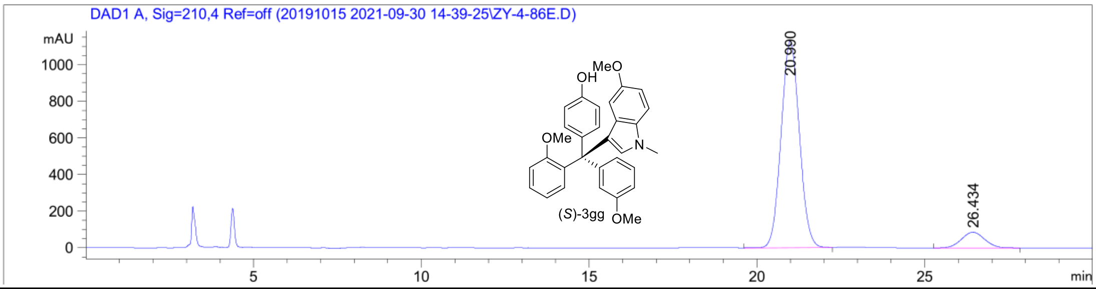

Peak	RetTime[min]	Type	Width[min]	Area[mAu*s]	Height[mAU]	Area[%]
1	20.990	BB	0.5904	4.25586e4	1127.39087	91.2275
2	26.434	BB	0.7383	4092.47412	85.20706	8.7725

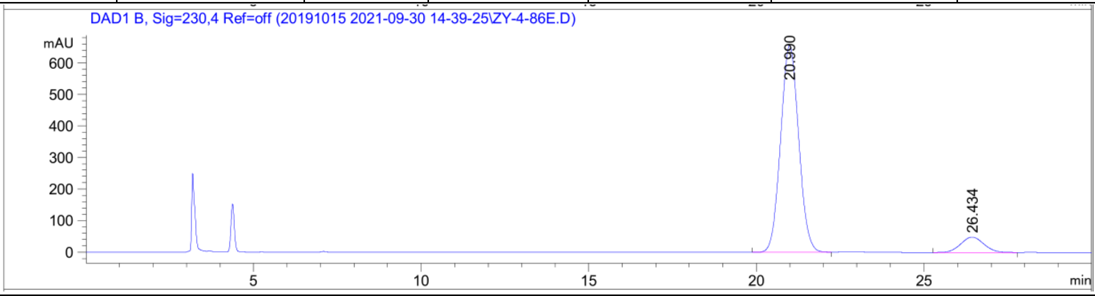

Peak	RetTime[min]	Type	Width[min]	Area[mAu*s]	Height[mAU]	Area[%]
1	20.990	BB	0.5843	2.45143e4	655.53363	91.2991
2	26.434	BB	0.7507	2336.23779	48.61066	8.7009

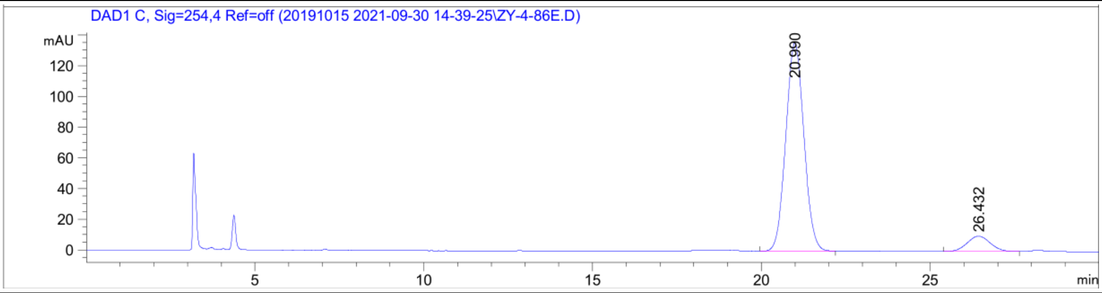

Peak	RetTime[min]	Type	Width[min]	Area[mAu*s]	Height[mAU]	Area[%]
1	20.990	BB	0.5776	5060.29346	135.54340	91.3724
2	26.432	BB	0.6660	477.80725	9.98964	8.6276

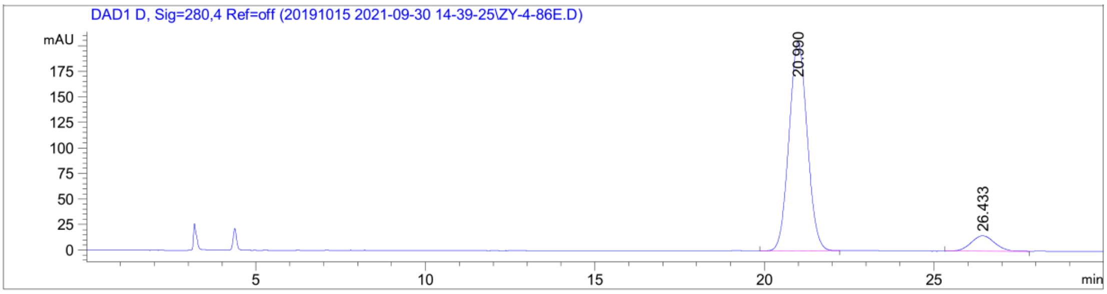

Peak	RetTime[min]	Type	Width[min]	Area[mAu*s]	Height[mAU]	Area[%]
1	20.990	BB	0.5793	7628.33496	204.43233	91.2906
2	26.433	BB	0.7017	727.76373	15.06231	8.7094

==

End of Report

Sample Name: ZY-4-86E-Rac

HPLC Condition:AD-H, *n*-Hexane/*i*PrOH = 95:5, 1.0 mL/min

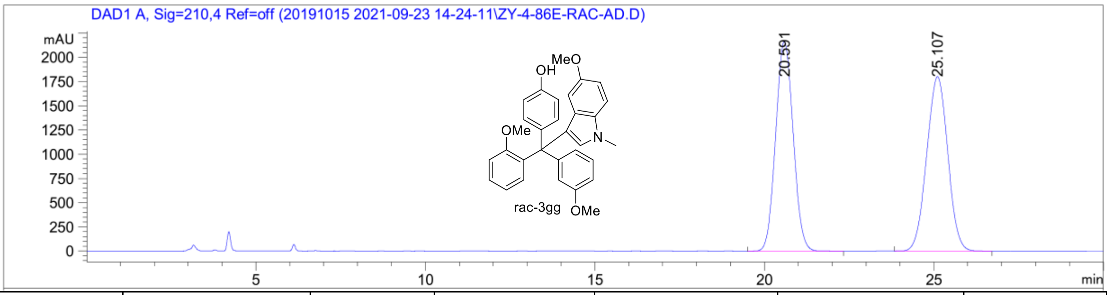

Peak	RetTime[min]	Type	Width[min]	Area[mAu*s]	Height[mAU]	Area[%]
1	20.591	BB	0.5655	7.77823e4	2163.28931	49.9315
2	25.107	BB	0.6847	7.79957e4	1802.79712	50.0685

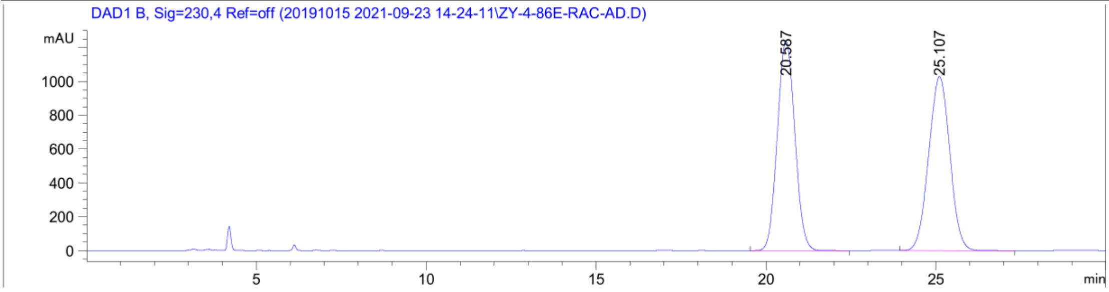

Peak	RetTime[min]	Type	Width[min]	Area[mAu*s]	Height[mAU]	Area[%]
1	20.587	BB	0.5645	4.45074e4	1240.71033	49.9965
2	25.107	BB	0.6809	4.45135e4	1028.51477	50.0035

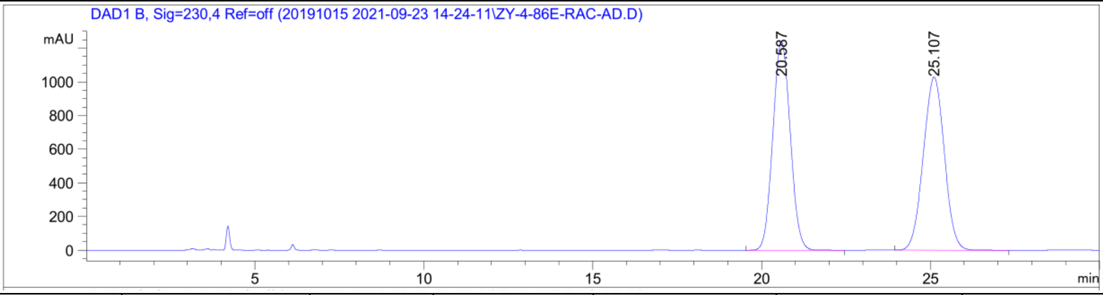

Peak	RetTime[min]	Type	Width[min]	Area[mAu*s]	Height[mAU]	Area[%]
1	20.587	BB	0.5595	9227.47168	257.84879	49.9354
2	25.107	BB	0.6818	9251.33301	213.37860	50.0646

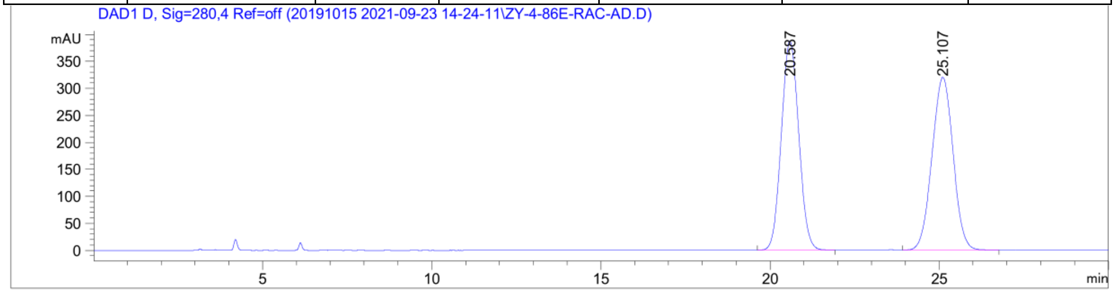

Peak	RetTime[min]	Type	Width[min]	Area[mAu*s]	Height[mAU]	Area[%]
1	20.587	BB	0.5591	1.38323e4	386.89661	50.0213
2	25.107	BB	0.6797	1.38206e4	320.13461	49.9787

==

End of Report

Sample Name: ZY-4-86F-OP

HPLC Condition:IC, *n*-Hexane/*i*PrOH = 95:5, 1.0 mL/min

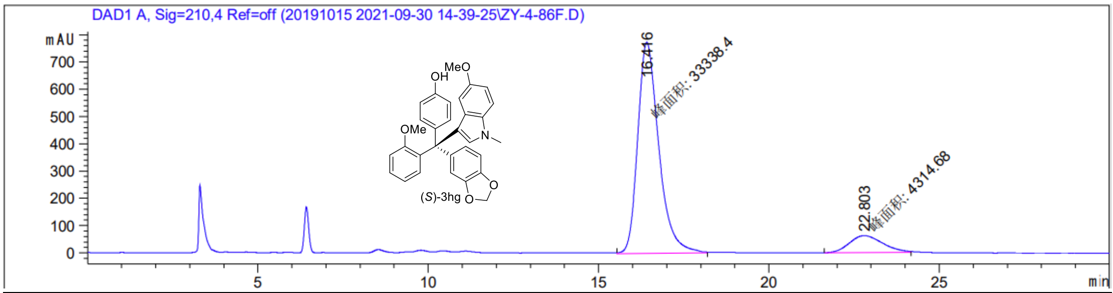

Peak	RetTime[min]	Type	Width[min]	Area[mAu*s]	Height[mAU]	Area[%]
1	16.416	MM	0.7163	3.33384e4	775.68542	88.5410
2	22.803	MM	1.1471	4314.68164	62.68951	11.4590

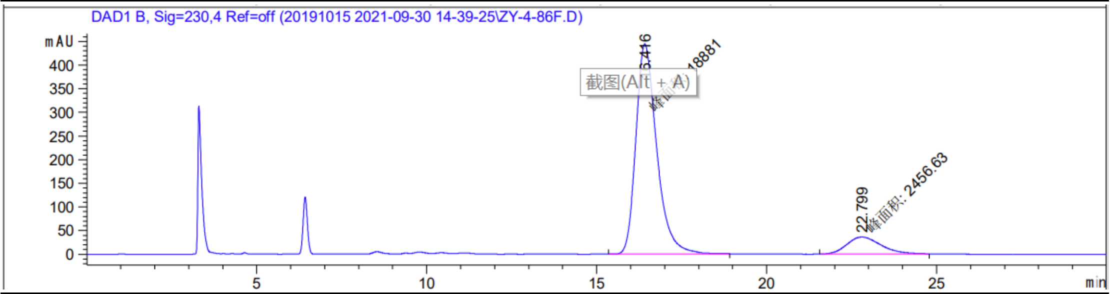

Peak	RetTime[min]	Type	Width[min]	Area[mAu*s]	Height[mAU]	Area[%]
1	16.416	MM	0.7090	1.88810e4	443.85840	88.4869
2	22.799	MM	1.1499	2456.63208	35.60609	11.5131

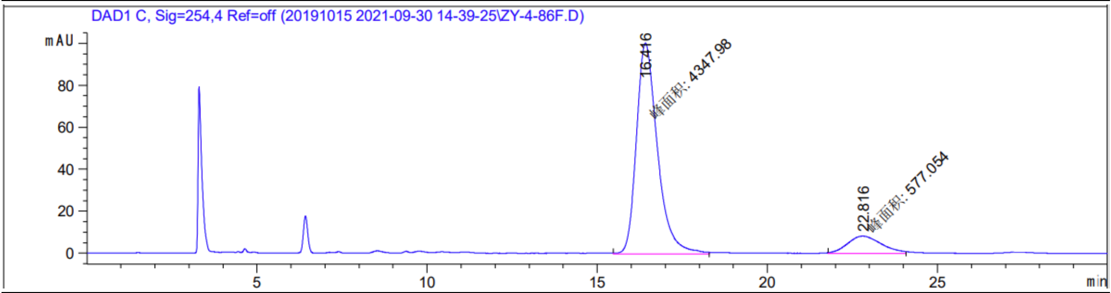

Peak	RetTime[min]	Type	Width[min]	Area[mAu*s]	Height[mAU]	Area[%]
1	16.416	MM	0.7203	4347.97852	100.61240	88.2832
2	22.816	MM	1.1625	577.05432	8.27334	11.7168

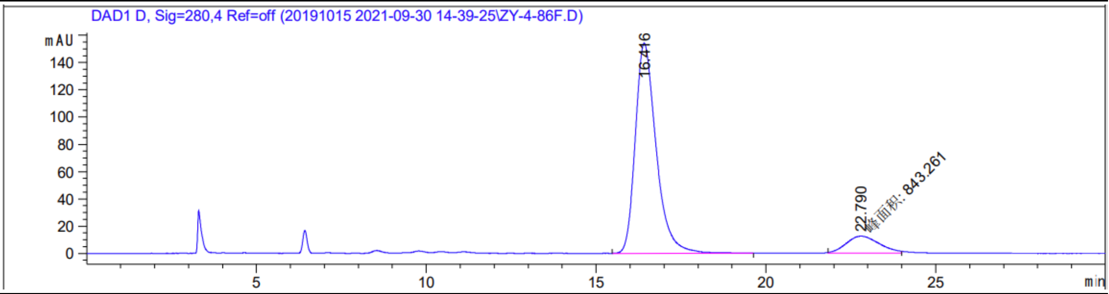

Peak	RetTime[min]	Type	Width[min]	Area[mAu*s]	Height[mAU]	Area[%]
1	16.416	BB	0.6465	6587.04395	154.15887	88.6511
2	22.790	MM	1.1313	843.26093	12.42308	11.3489

==

End of Report

Sample Name: ZY-4-86F-Rac

HPLC Condition:IC, *n*-Hexane/*i*PrOH = 95:5, 1.0 mL/min

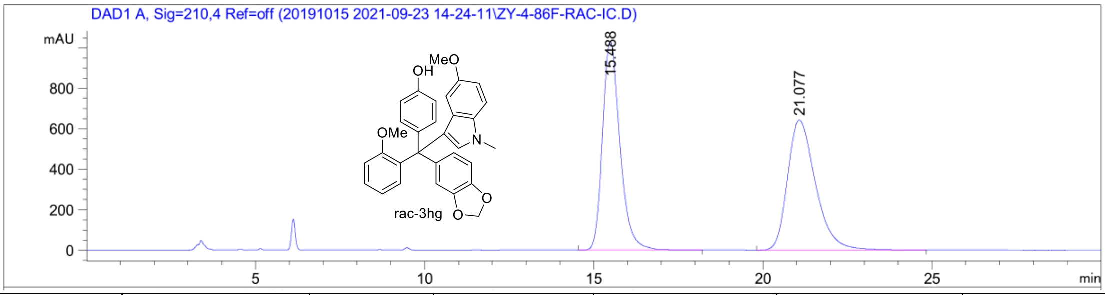

Peak	RetTime[min]	Type	Width[min]	Area[mAu*s]	Height[mAU]	Area[%]
1	15.488	BB	0.5498	3.69282e4	1031.28113	49.9166
2	21.077	BB	0.8850	3.70515e4	642.28479	50.0834

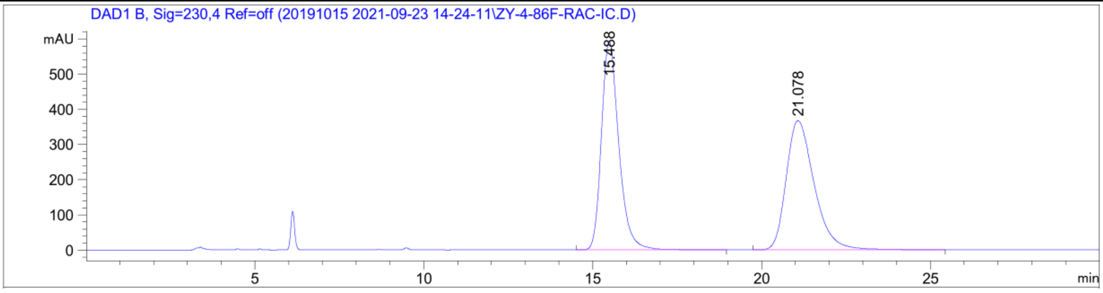

Peak	RetTime[min]	Type	Width[min]	Area[mAu*s]	Height[mAU]	Area[%]
1	15.488	BB	0.5476	2.12237e4	592.92303	50.0687
2	21.078	BB	0.8844	2.11655e4	367.24396	49.9313

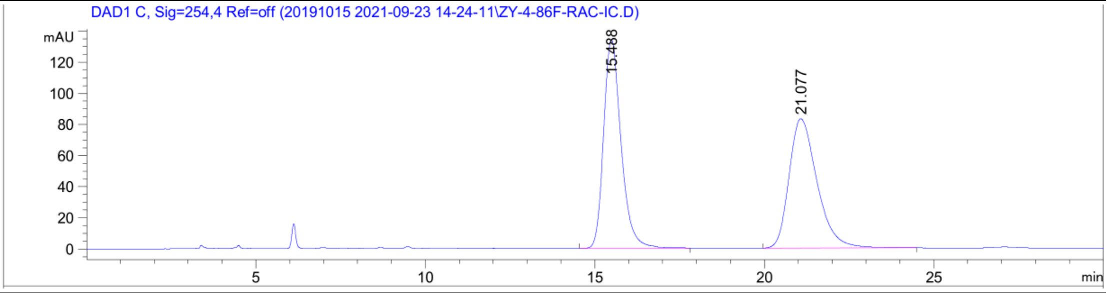

Peak	RetTime[min]	Type	Width[min]	Area[mAu*s]	Height[mAU]	Area[%]
1	15.488	BB	0.5442	4787.25977	134.18378	50.0913
2	21.077	BB	0.8720	4769.81201	83.05464	49.9087

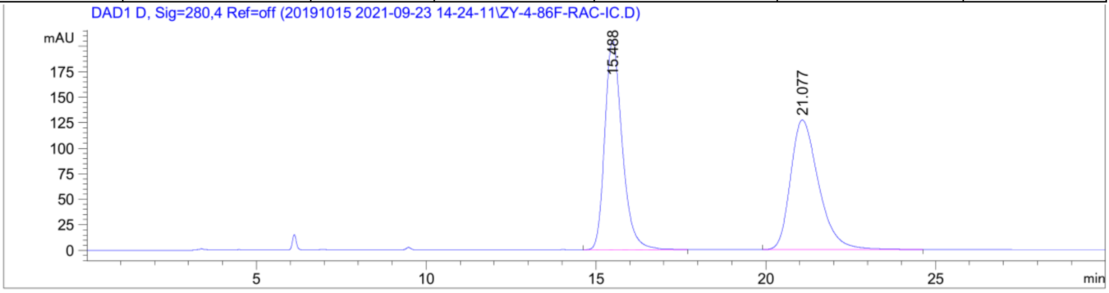

Peak	RetTime[min]	Type	Width[min]	Area[mAu*s]	Height[mAU]	Area[%]
1	15.488	BB	0.5455	7311.97998	205.31229	50.0480
2	21.077	BB	0.8823	7297.96191	127.02544	49.9520

==

End of Report

Sample Name: ZY-4-93B

HPLC Condition:AD-H, *n*-Hexane/*i*PrOH = 95:5, 1.0 mL/min

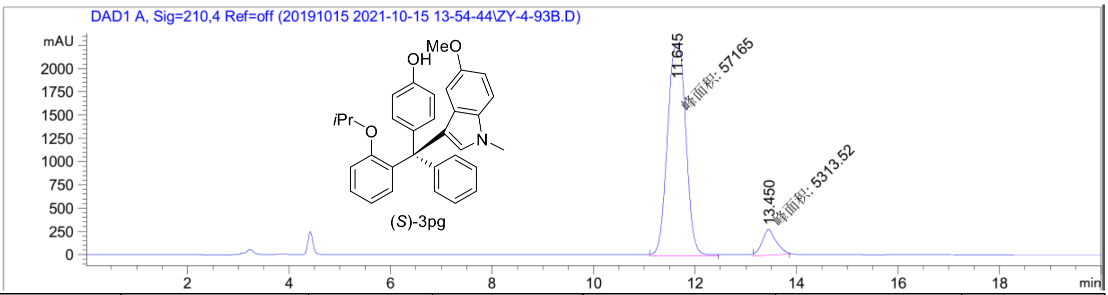

Peak	RetTime[min]	Type	Width[min]	Area[mAu*s]	Height[mAU]	Area[%]
1	11.645	MM	0.4182	5.71650e4	2278.40796	91.4955
2	13.450	MM	0.3218	5313.51660	275.16879	8.5045

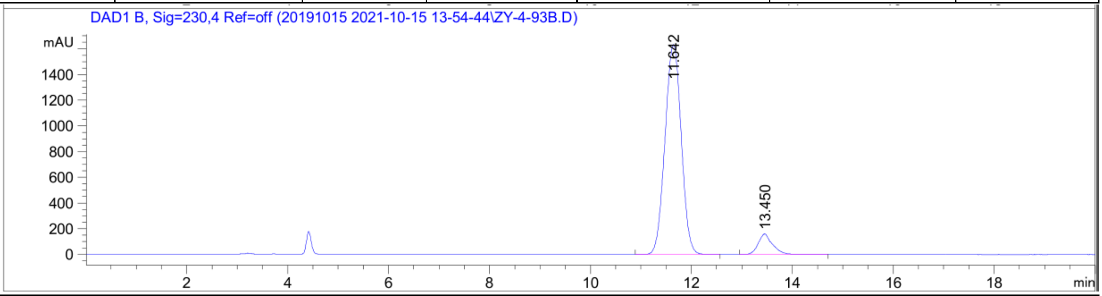

Peak	RetTime[min]	Type	Width[min]	Area[mAu*s]	Height[mAU]	Area[%]
1	11.642	BB	0.3530	3.65154e4	1632.27319	92.0814
2	13.450	BB	0.2798	3140.17529	159.43127	7.9186

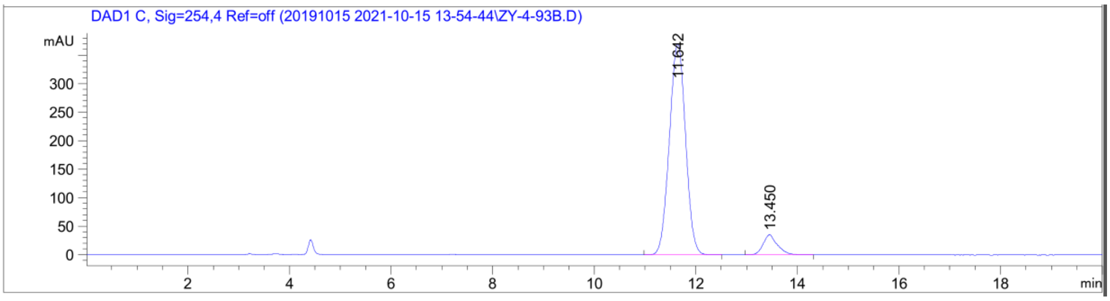

Peak	RetTime[min]	Type	Width[min]	Area[mAu*s]	Height[mAU]	Area[%]
1	13.450	BB	0.3481	8208.26758	371.01611	92.1731
2	13.450	BB	0.2837	697.01044	35.39564	7.8269

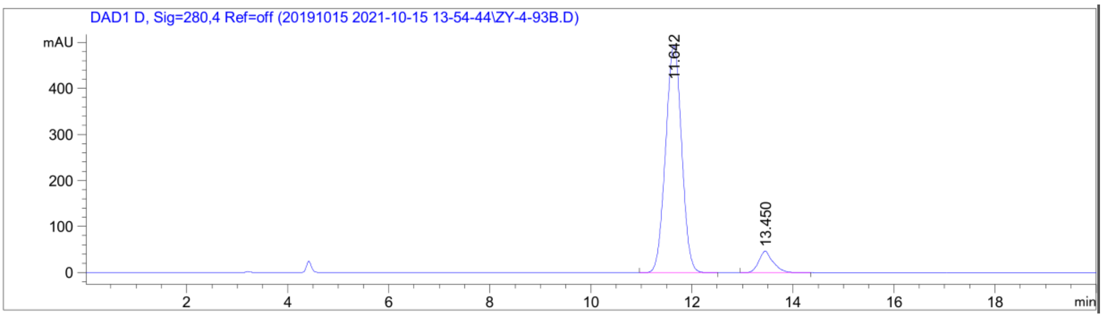

Peak	RetTime[min]	Type	Width[min]	Area[mAu*s]	Height[mAU]	Area[%]
1	11.642	BB	0.3479	1.09195e4	493.87476	92.2046
2	13.450	BB	0.2794	923.18646	46.95439	7.7954

==

End of Report

Sample Name: ZY-4-93B-Rac

HPLC Condition:AD-H, *n*-Hexane/*i*PrOH = 95:5, 1.0 mL/min

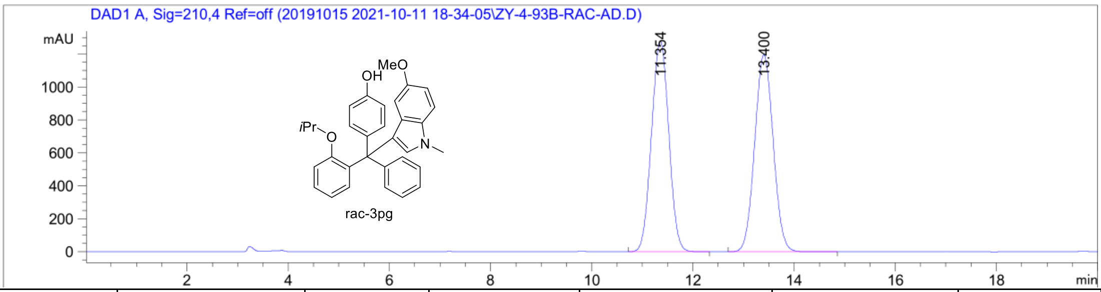

Peak	RetTime[min]	Type	Width[min]	Area[mAu*s]	Height[mAU]	Area[%]
1	11.354	BB	0.3756	3.03534e4	1275.69531	49.9441
2	13.400	BB	0.3990	3.04213e4	1194.87109	50.0559

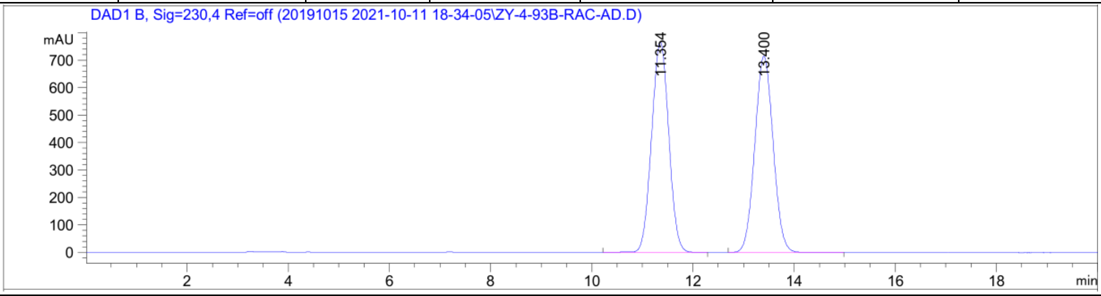

Peak	RetTime[min]	Type	Width[min]	Area[mAu*s]	Height[mAU]	Area[%]
1	11.354	VB R	0.3710	1.80765e4	765.73743	50.1217
2	13.400	BB	0.3938	1.79887e4	714.24347	49.8783

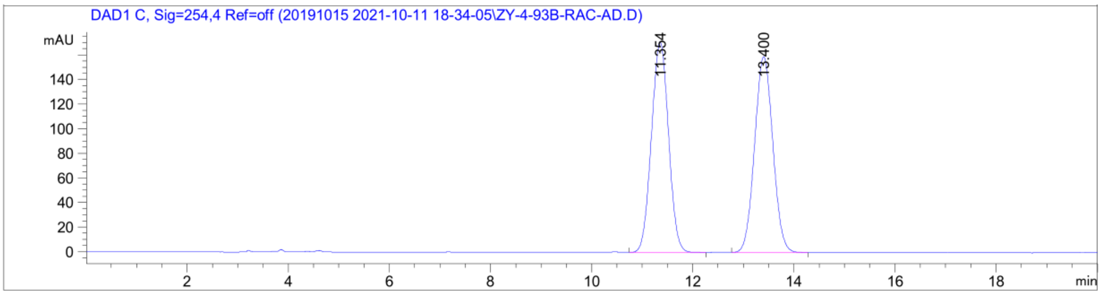

Peak	RetTime[min]	Type	Width[min]	Area[mAu*s]	Height[mAU]	Area[%]
1	11.354	BB	0.3703	4012.74219	170.69788	50.1066
2	13.400	BB	0.3930	3995.66528	159.08495	49.8934

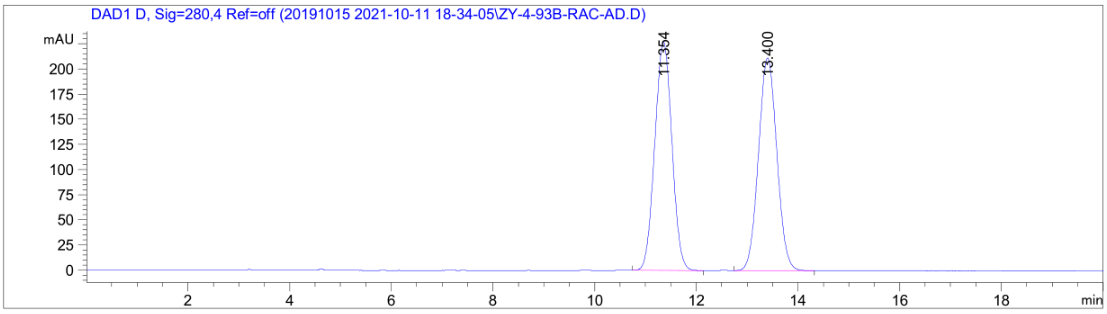

Peak	RetTime[min]	Type	Width[min]	Area[mAu*s]	Height[mAU]	Area[%]
1	11.354	BB	0.3699	5322.42822	226.68182	50.0756
2	13.400	BB	0.3929	5306.34814	211.33919	49.9244

==

End of Report

Sample Name: ZY-4-86A-OP

HPLC Condition:AD-H, *n*-Hexane/*i*PrOH = 95:5, 1.0 mL/min

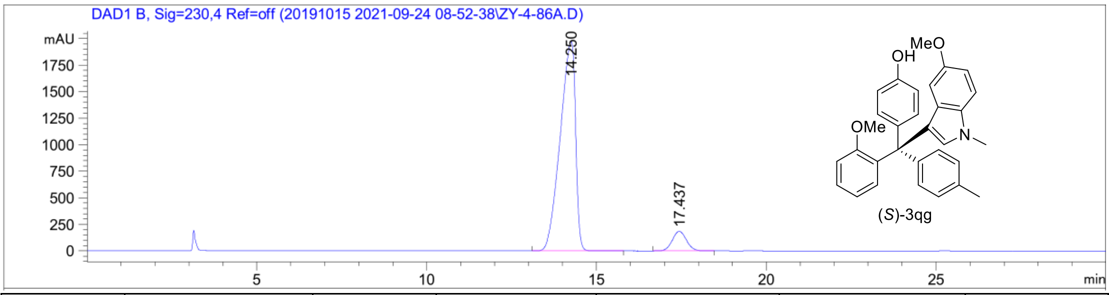

Peak	RetTime[min]	Type	Width[min]	Area[mAu*s]	Height[mAU]	Area[%]
1	14.250	BB	0.4944	6.20062e4	1974.48035	91.9980
2	17.437	BB	0.4549	5393.32813	184.56825	8.0020

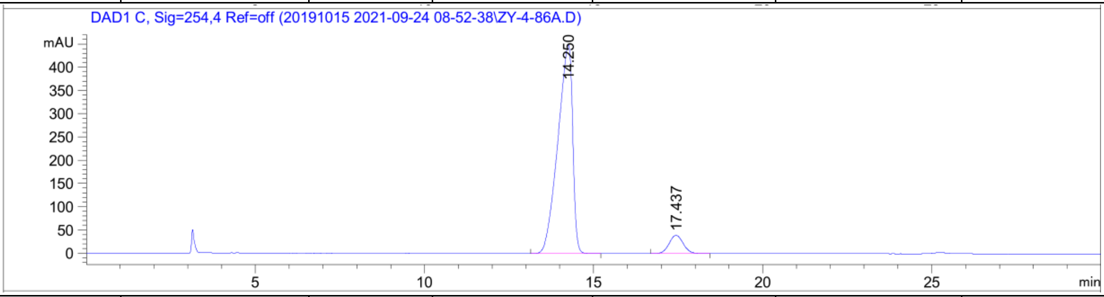

Peak	RetTime[min]	Type	Width[min]	Area[mAu*s]	Height[mAU]	Area[%]
1	14.250	BB	0.4607	1.36582e4	449.18689	92.2184
2	17.437	BB	0.4551	1152.51355	39.42315	7.7816

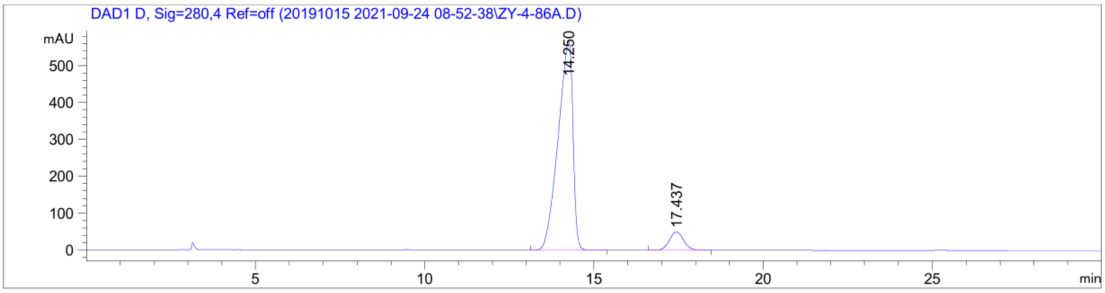

Peak	RetTime[min]	Type	Width[min]	Area[mAu*s]	Height[mAU]	Area[%]
1	14.250	BB	0.4626	1.72667e4	568.12494	92.2301
2	17.437	BB	0.4592	1454.62561	49.74520	7.7699

==

End of Report

Sample Name: ZY-4-86A-Rac

HPLC Condition:AD-H, *n*-Hexane/*i*PrOH = 95:5, 1.0 mL/min

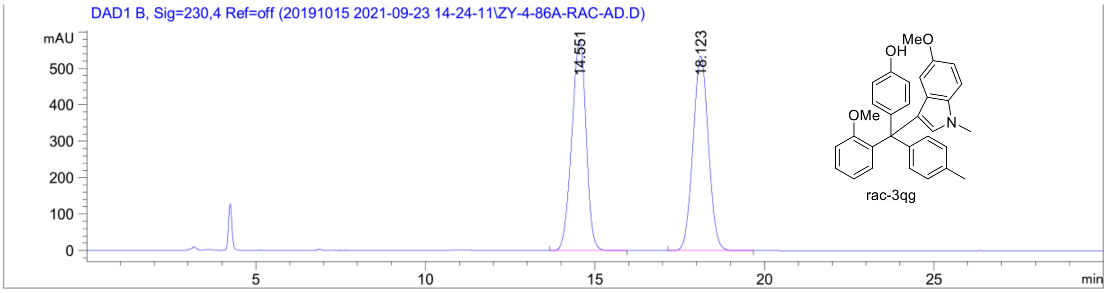

Peak	RetTime[min]	Type	Width[min]	Area[mAu*s]	Height[mAU]	Area[%]
1	14.551	BB	0.4593	1.69621e4	576.51978	50.0278
2	18.123	BB	0.4919	1.69433e4	534.33392	49.9722

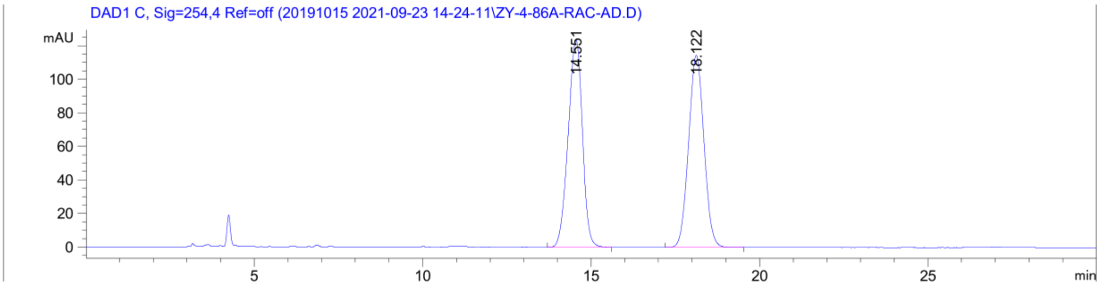

Peak	RetTime[min]	Type	Width[min]	Area[mAu*s]	Height[mAU]	Area[%]
1	14.551	BB	0.4572	3632.59595	123.49406	50.0384
2	18.122	BB	0.4918	3627.01636	114.44120	49.9616

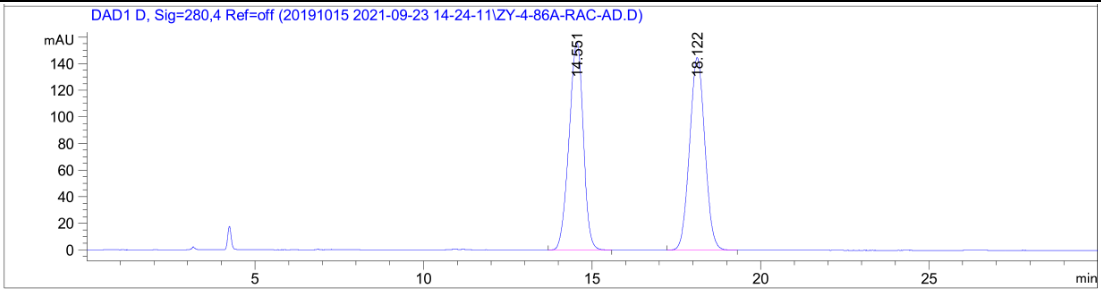

Peak	RetTime[min]	Type	Width[min]	Area[mAu*s]	Height[mAU]	Area[%]
1	14.551	BB	0.4587	4586.61816	156.12553	50.0488
2	18.122	BB	0.4912	4577.67285	144.64790	49.9512

==

End of Report

Sample Name: ZY-4-93A-OP

HPLC Condition:AD-H, *n*-Hexane/*i*PrOH = 95:5, 1.0 mL/min

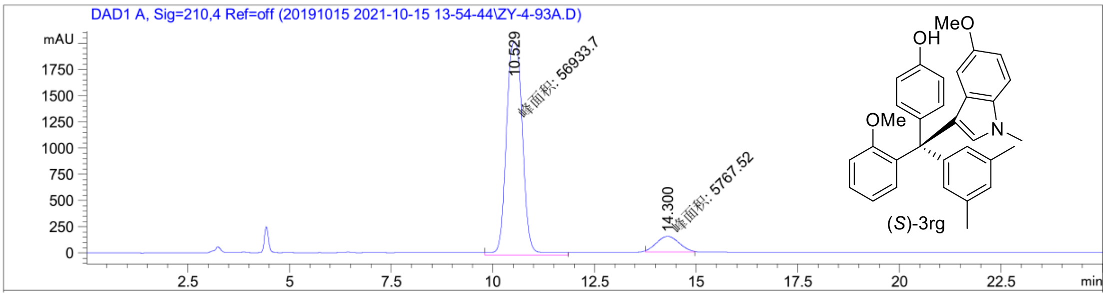

Peak	RetTime[min]	Type	Width[min]	Area[mAu*s]	Height[mAU]	Area[%]
1	10.529	MM	0.4657	5.69337e4	2037.60828	90.8016
2	14.300	MM	0.6449	5767.52344	149.05142	9.1984

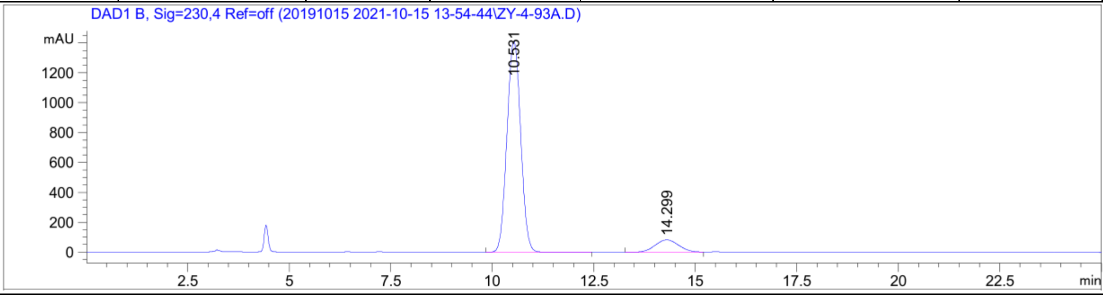

Peak	RetTime[min]	Type	Width[min]	Area[mAu*s]	Height[mAU]	Area[%]
1	10.531	BB	0.3721	3.32121e4	1413.45361	90.9545
2	14.299	BB	0.6336	3302.95801	82.05054	9.0455

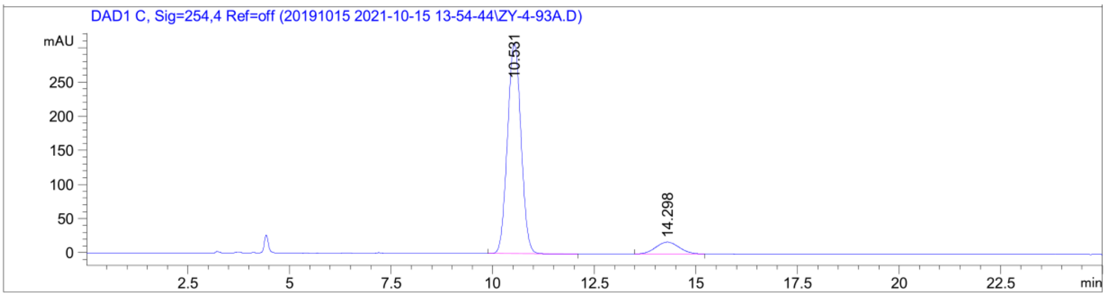

Peak	RetTime[min]	Type	Width[min]	Area[mAu*s]	Height[mAU]	Area[%]
1	10.531	BB	0.3666	7143.11719	307.99341	91.2013
2	14.298	BB	0.6057	689.13922	17.26447	8.7987

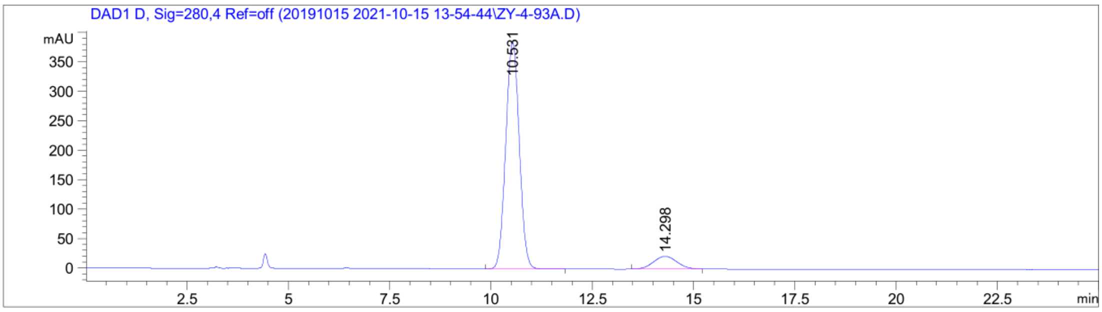

Peak	RetTime[min]	Type	Width[min]	Area[mAu*s]	Height[mAU]	Area[%]
1	10.531	BB	0.3664	8923.96680	385.09274	91.2043
2	14.298	BB	0.6106	860.62842	21.52035	8.7957

==

End of Report

Sample Name: ZY-4-93A-Rac

HPLC Condition:AD-H, *n*-Hexane/*i*PrOH = 95:5, 1.0 mL/min

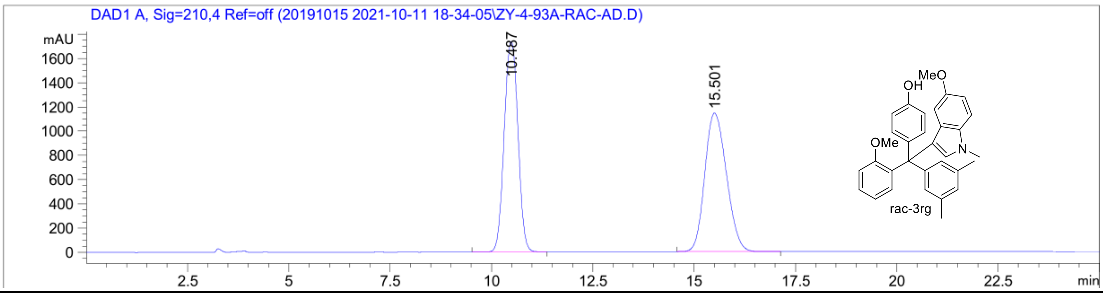

Peak	RetTime[min]	Type	Width[min]	Area[mAu*s]	Height[mAU]	Area[%]
1	10.487	BB	0.3774	4.09237e4	1733.92419	49.0387
2	15.501	BB	0.5878	4.25283e4	1143.78455	50.9613

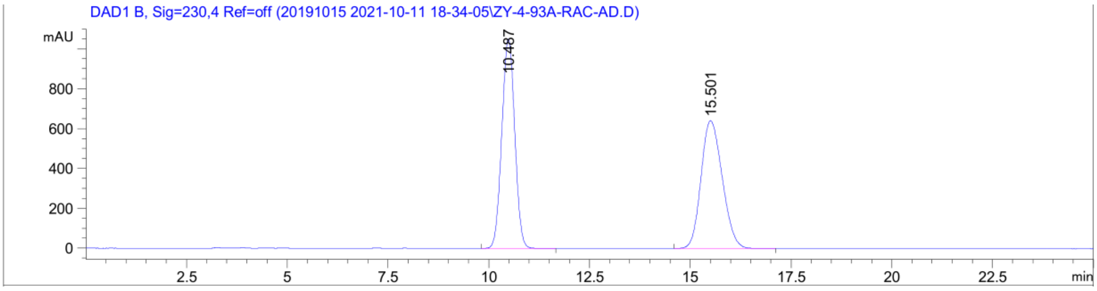

Peak	RetTime[min]	Type	Width[min]	Area[mAu*s]	Height[mAU]	Area[%]
1	10.487	BB	0.3521	2.33868e4	1049.19250	49.9629
2	15.501	BB	0.5744	2.34215e4	640.86066	50.0371

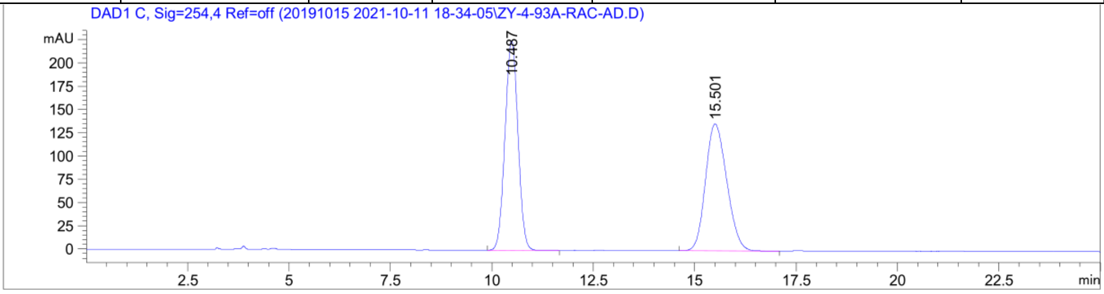

Peak	RetTime[min]	Type	Width[min]	Area[mAu*s]	Height[mAU]	Area[%]
1	10.487	BB	0.3485	4992.17969	225.30325	50.0819
2	15.501	BB	0.5713	4975.84717	136.48895	49.9181

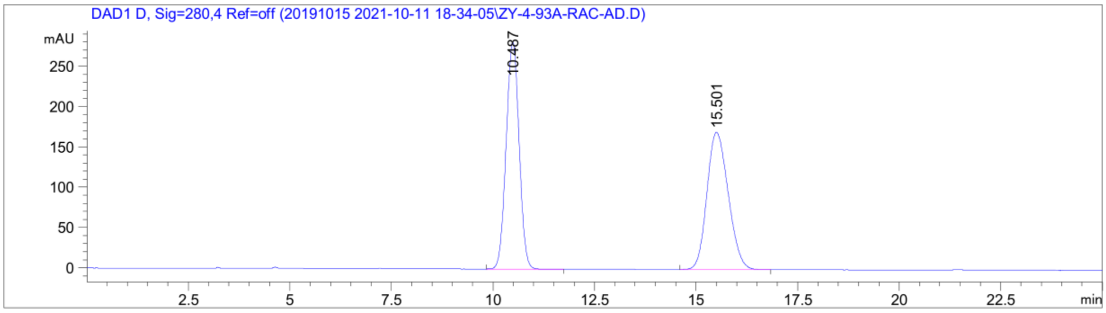

Peak	RetTime[min]	Type	Width[min]	Area[mAu*s]	Height[mAU]	Area[%]
1	10.487	BB	0.3483	6230.43115	281.39886	50.1358
2	15.501	BB	0.5749	6196.68799	170.14333	49.8642

==

End of Report

Sample Name: ZY-4-86B-OP

HPLC Condition:IC, *n*-Hexane/*i*PrOH = 97:3, 1.0 mL/min

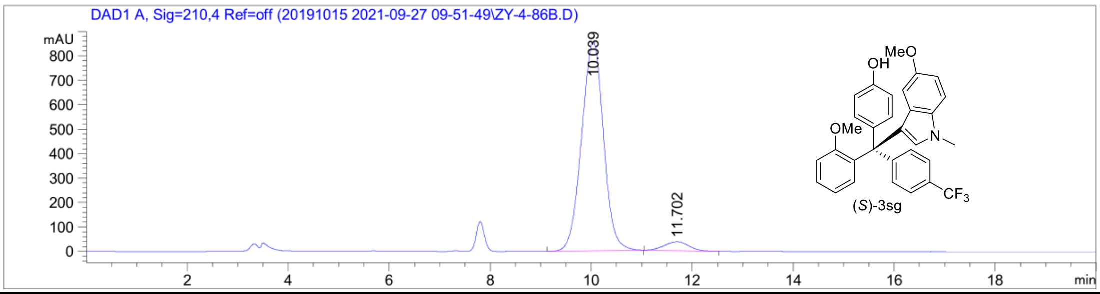

Peak	RetTime[min]	Type	Width[min]	Area[mAu*s]	Height[mAU]	Area[%]
1	10.039	BB	0.4744	2.58219e4	854.75122	95.5277
2	11.702	BB	0.5180	1208.89453	36.34778	4.4723

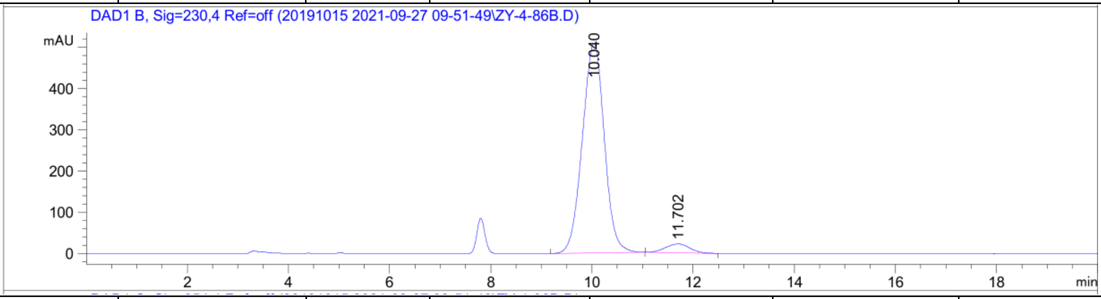

Peak	RetTime[min]	Type	Width[min]	Area[mAu*s]	Height[mAU]	Area[%]
1	10.040	BB	0.4720	1.53581e4	509.01712	95.5429
2	11.702	BB	0.5067	716.45392	21.50212	4.4571

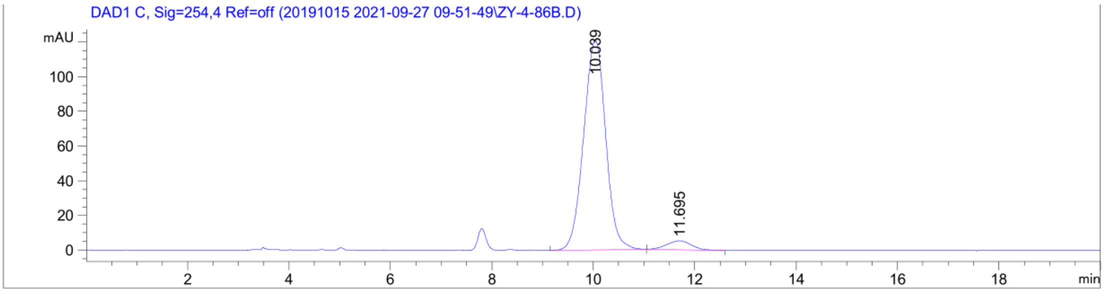

Peak	RetTime[min]	Type	Width[min]	Area[mAu*s]	Height[mAU]	Area[%]
1	10.039	BB	0.4719	3652.29834	121.07981	95.5425
2	11.695	BB	0.4994	170.39699	5.05310	4.4575

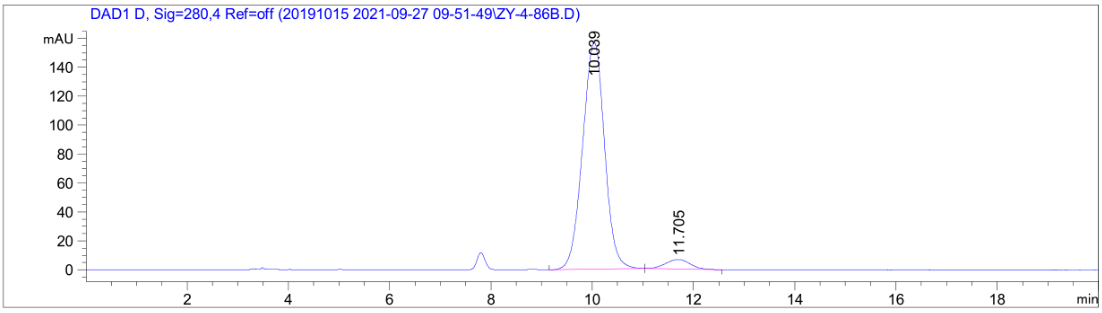

Peak	RetTime[min]	Type	Width[min]	Area[mAu*s]	Height[mAU]	Area[%]
1	10.039	BB	0.4714	4728.27783	156.98123	95.5478
2	11.705	BB	0.5046	220.32109	6.58064	4.4522

==

End of Report

Sample Name: ZY-4-86B-Rac

HPLC Condition:IC, *n*-Hexane/*i*PrOH = 97:3, 1.0 mL/min

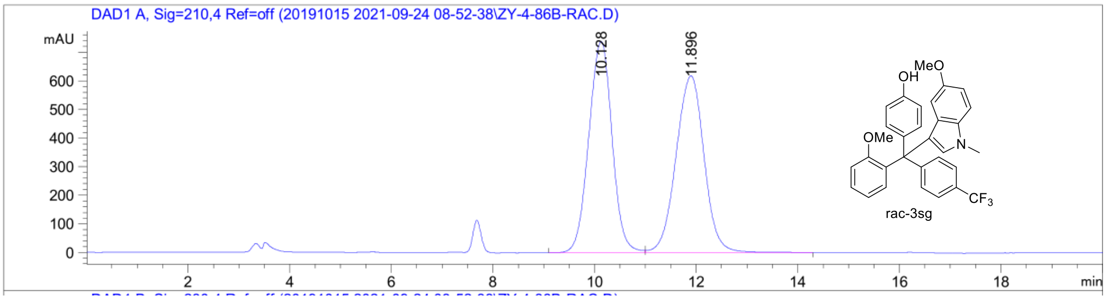

Peak	RetTime[min]	Type	Width[min]	Area[mAu*s]	Height[mAU]	Area[%]
1	10.128	BV	0.5023	2.35752e4	738.69580	49.7666
2	11.896	VB	0.6068	2.37963e4	618.56238	50.2334

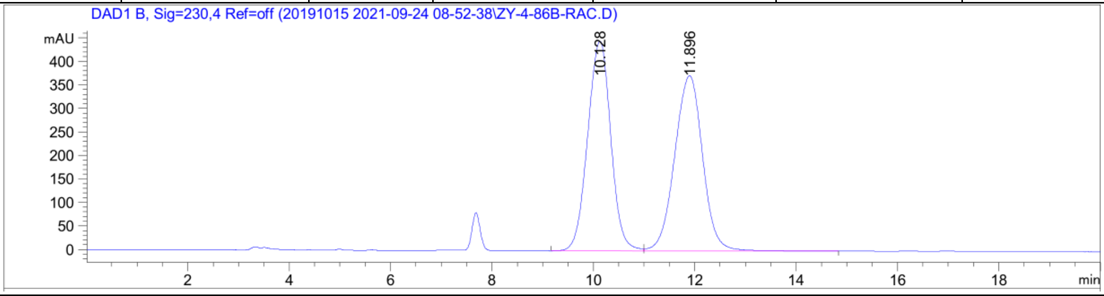

Peak	RetTime[min]	Type	Width[min]	Area[mAu*s]	Height[mAU]	Area[%]
1	10.128	BV	0.4982	1.41496e4	445.91321	49.7199
2	11.896	VB	0.6037	1.43090e4	372.88580	50.2801

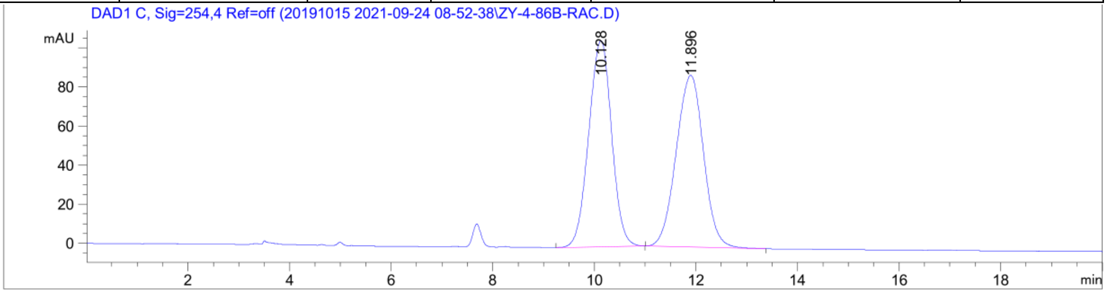

Peak	RetTime[min]	Type	Width[min]	Area[mAu*s]	Height[mAU]	Area[%]
1	10.128	BB	0.4913	3303.03320	105.50297	50.0900
2	11.896	BB	0.5889	3291.16797	87.88772	49.9100

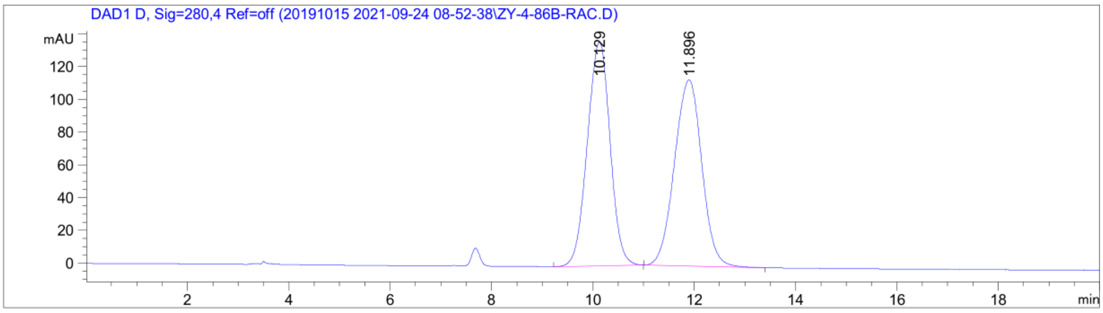

Peak	RetTime[min]	Type	Width[min]	Area[mAu*s]	Height[mAU]	Area[%]
1	10.129	BB	0.4928	4272.78613	136.63490	50.0372
2	11.896	BB	0.5955	4266.44043	113.76900	49.9628

==

End of Report

Sample Name: ZY-4-86D-OP

HPLC Condition:AD-H, *n*-Hexane/*i*PrOH = 95:5, 1.0 mL/min

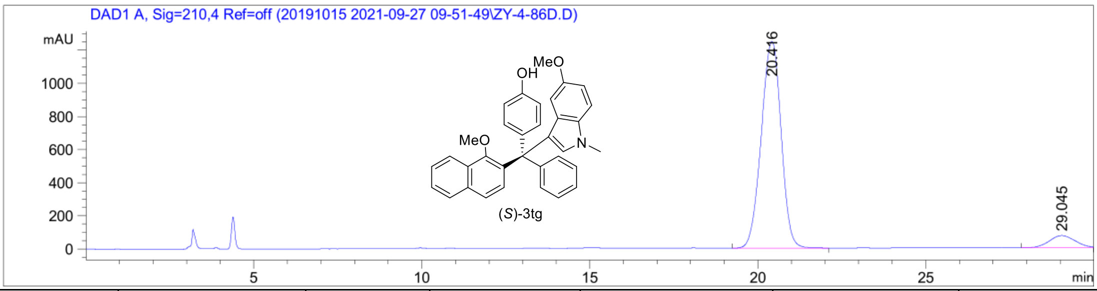

Peak	RetTime[min]	Type	Width[min]	Area[mAu*s]	Height[mAU]	Area[%]
1	20.416	BB	0.6490	5.11819e4	1246.68652	92.7710
2	29.045	BBA	0.8273	3988.27075	73.94466	7.2290

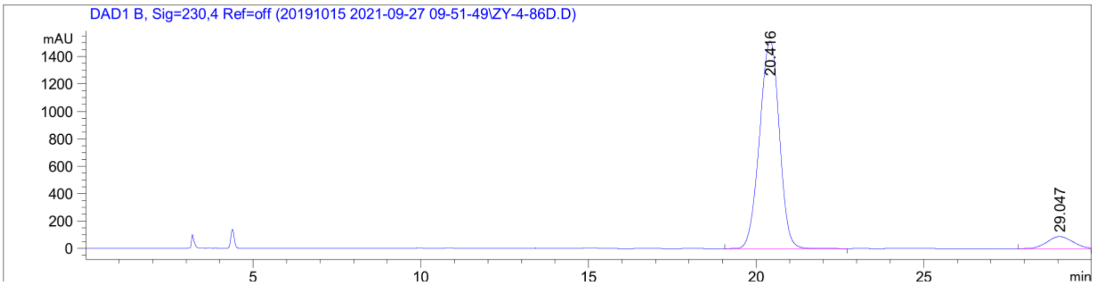

Peak	RetTime[min]	Type	Width[min]	Area[mAu*s]	Height[mAU]	Area[%]
1	20.416	BB	0.6419	6.20679e4	1515.10608	92.8866
2	29.047	BBA	0.8369	4753.27148	88.45672	7.1134

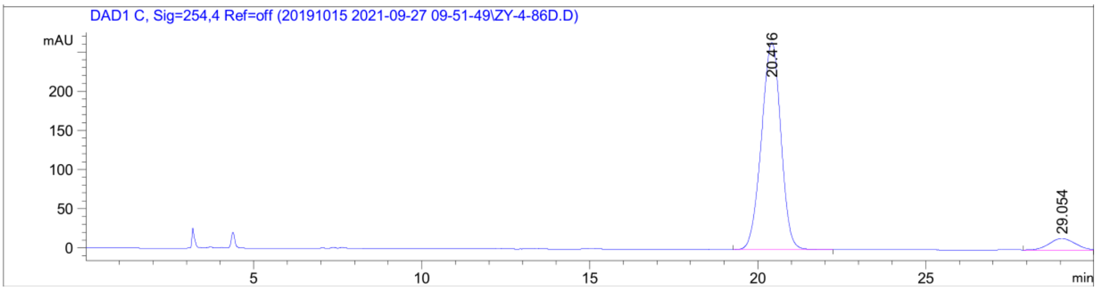

Peak	RetTime[min]	Type	Width[min]	Area[mAu*s]	Height[mAU]	Area[%]
1	20.416	BB	0.6273	1.06240e4	264.10907	93.2022
2	29.054	BBA	0.7600	774.87115	14.67465	6.7978

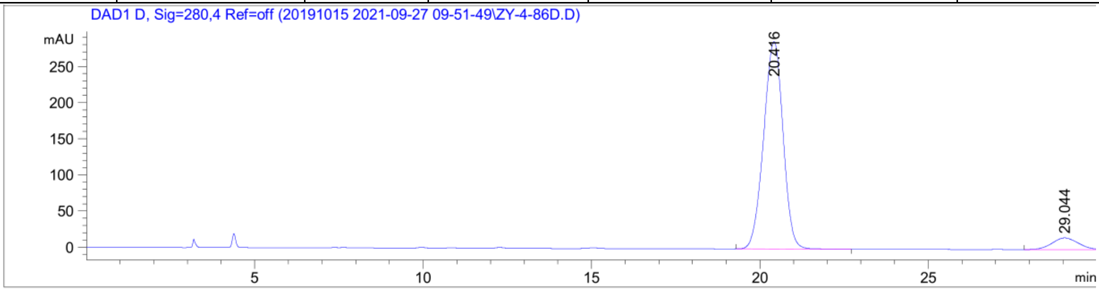

Peak	RetTime[min]	Type	Width[min]	Area[mAu*s]	Height[mAU]	Area[%]
1	20.416	BB	0.6266	1.15146e4	286.68625	93.0986
2	29.044	BBA	0.7473	853.58142	15.97791	6.9014

==

End of Report

Sample Name: ZY-4-86D-Rac

HPLC Condition:AD-H, *n*-Hexane/*i*PrOH = 95:5, 1.0 mL/min

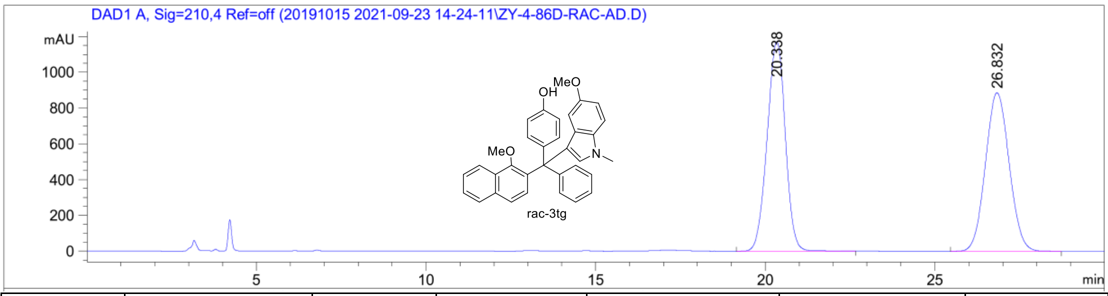

Peak	RetTime[min]	Type	Width[min]	Area[mAu*s]	Height[mAU]	Area[%]
1	20.338	BB	0.5859	4.39439e4	1170.83398	50.4658
2	26.832	BB	0.7620	4.31327e4	885.98846	49.5342

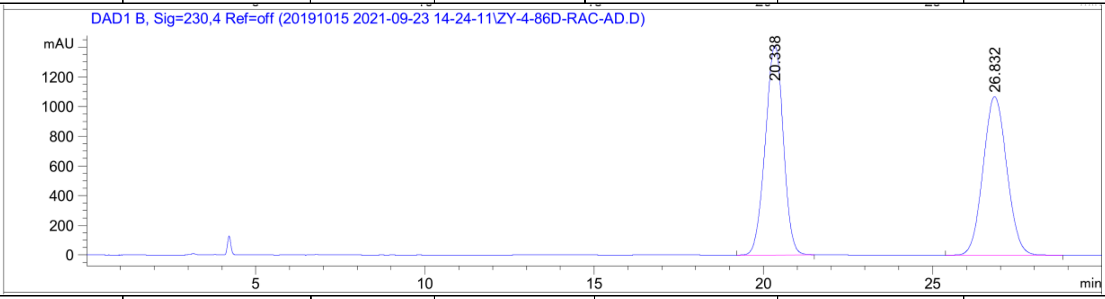

Peak	RetTime[min]	Type	Width[min]	Area[mAu*s]	Height[mAU]	Area[%]
1	20.338	BB	0.5816	5.23095e4	1407.62524	50.1785
2	26.832	BB	0.7614	5.19374e4	1067.83801	49.8215

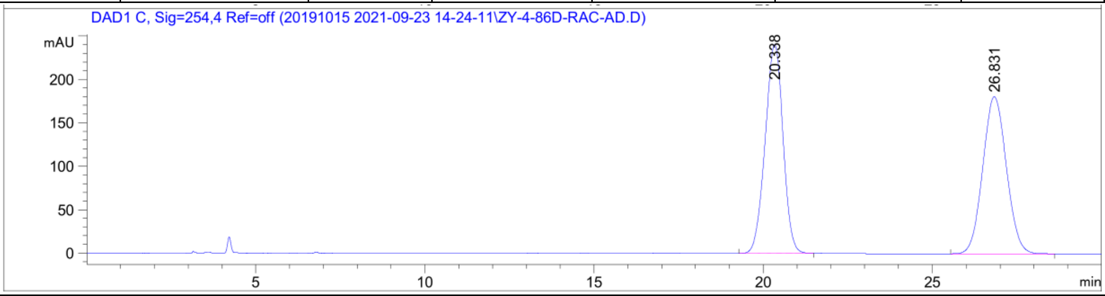

Peak	RetTime[min]	Type	Width[min]	Area[mAu*s]	Height[mAU]	Area[%]
1	20.338	BB	0.5765	8854.78809	239.96416	50.2208
2	26.831	BB	0.7568	8776.92285	180.66096	49.7792

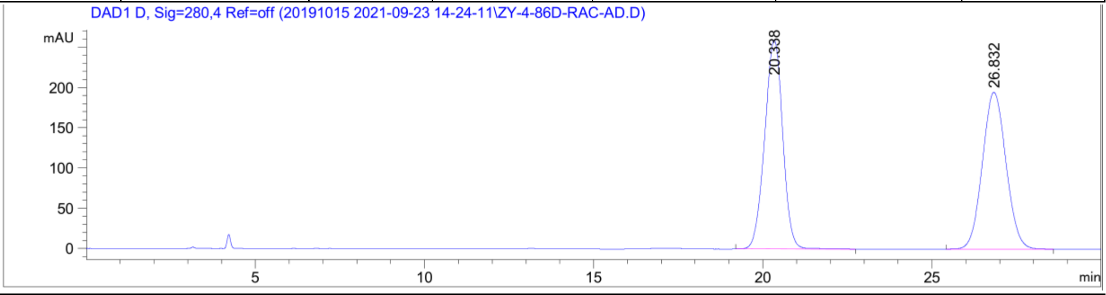

Peak	RetTime[min]	Type	Width[min]	Area[mAu*s]	Height[mAU]	Area[%]
1	20.338	BB	0.5771	9633.75000	259.52628	50.5168
2	26.832	BB	0.7595	9436.64551	194.69366	49.4832

==

End of Report

Sample Name: ZY-5-19A

HPLC Condition: AD-H, *n*-Hexane/*i*PrOH = 97:3, 1.0 mL/min

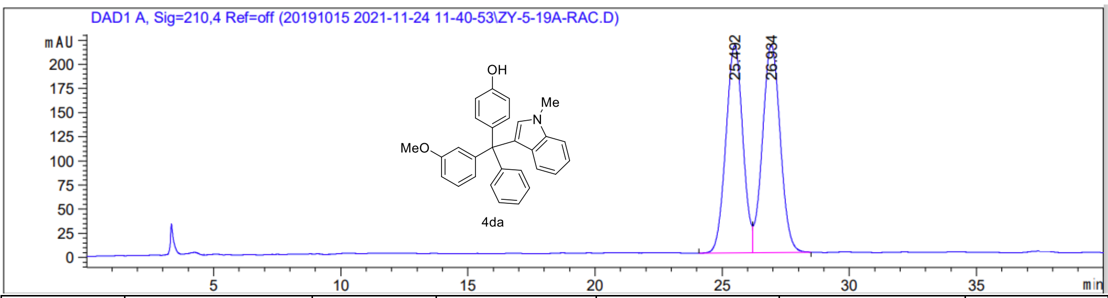

Peak	RetTime[min]	Type	Width[min]	Area[mAu*s]	Height[mAU]	Area[%]
1	25.492	BV	0.7211	9953.42285	214.60017	48.7727
2	26.934	VB	0.7630	1.04543e4	215.12825	51.2273

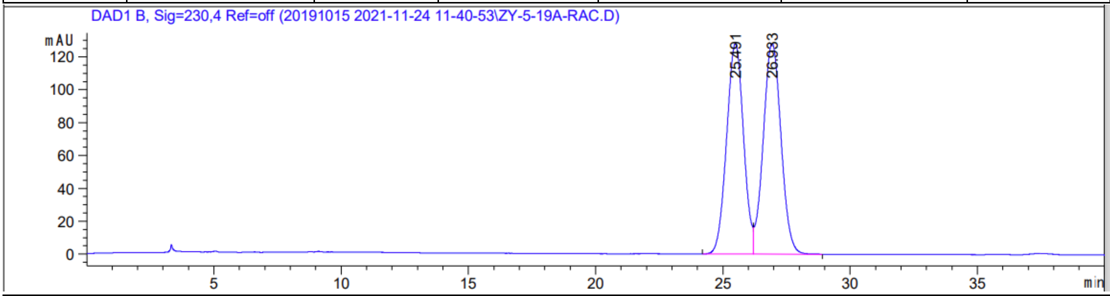

Peak	RetTime[min]	Type	Width[min]	Area[mAu*s]	Height[mAU]	Area[%]
1	25.491	BV	0.7224	5926.16504	127.94080	48.7497
2	26.933	VB	0.7565	6230.13867 1	128.29707	51.2503

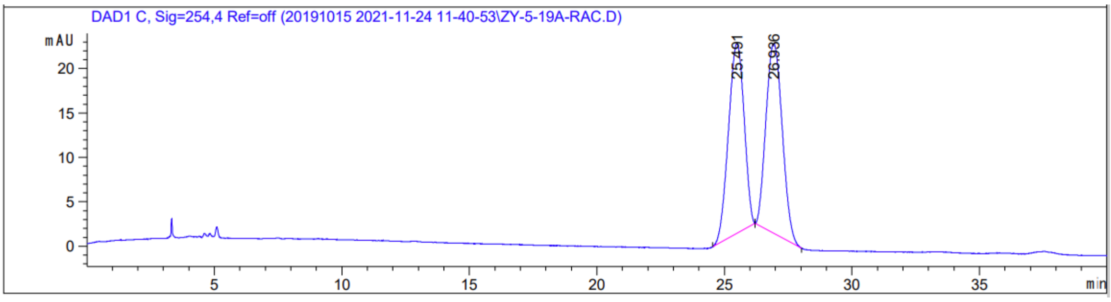

Peak	RetTime[min]	Type	Width[min]	Area[mAu*s]	Height[mAU]	Area[%]
1	25.491	BB	0.6583	912.06696	21.35436	48.9722
2	26.936	BB	0.6932	950.35248	21.35408	51.0278

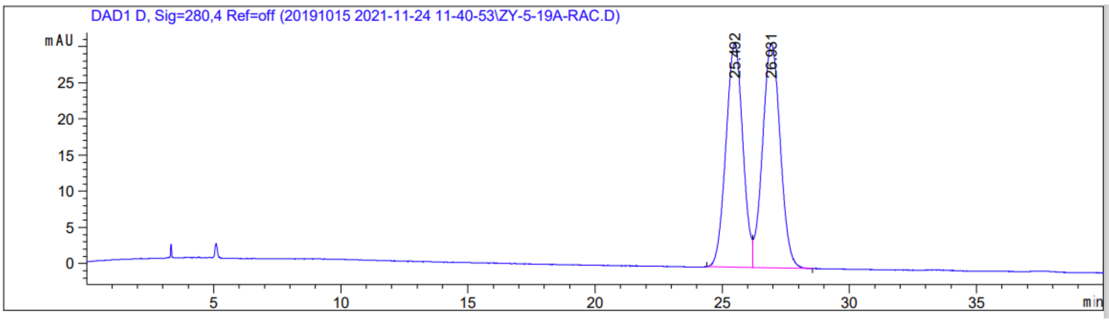

Peak	RetTime[min]	Type	Width[min]	Area[mAu*s]	Height[mAU]	Area[%]
1	25.492	BV	0.7032	1427.11011	30.87685	48.7428
2	26.931	VB	0.7293	1500.72839	30.97420	51.2572

==

End of Report

Sample Name: ZY-5-19B-OP

HPLC Condition: IC, *n*-Hexane/*i*PrOH = 98:2, 0.4 mL/min

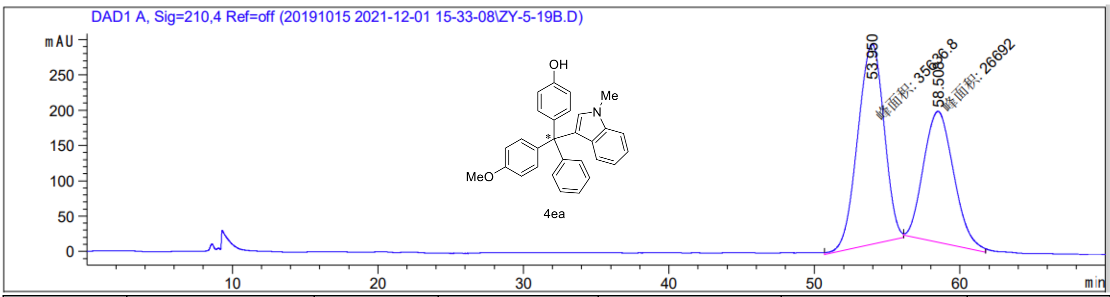

Peak	RetTime[min]	Type	Width[min]	Area[mAu*s]	Height[mAU]	Area[%]
1	53.950	MM	2.0945	3.56368e4	283.57196	57.1755
2	58.508	MM	2.3985	2.66920e4	185.47990	42.8245

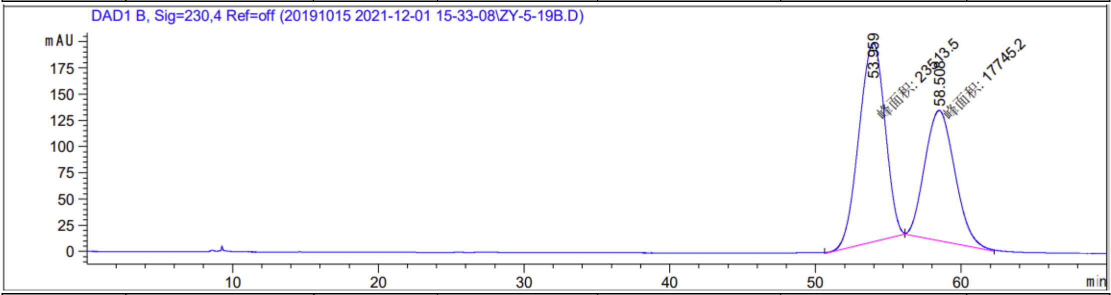

Peak	RetTime[min]	Type	Width[min]	Area[mAu*s]	Height[mAU]	Area[%]
1	53.959	MM	2.0678	2.35135e4	189.52016	56.9904
2	58.508	MM	2.3791	1.77452e4	124.31176	43.0096

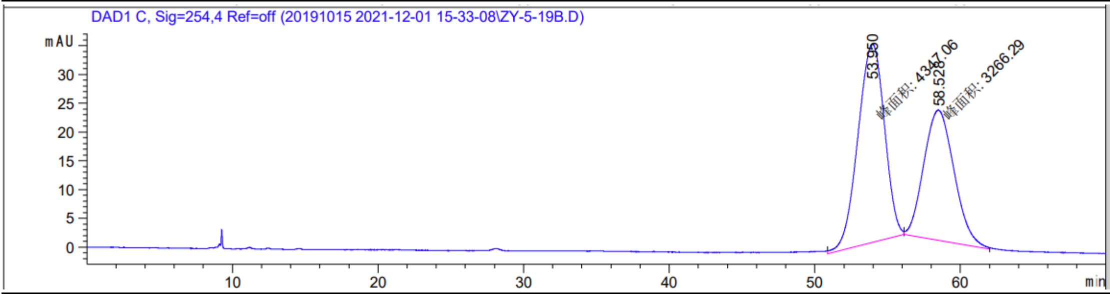

Peak	RetTime[min]	Type	Width[min]	Area[mAu*s]	Height[mAU]	Area[%]
1	53.950	MM	2.1018	4347.05908	34.47020	57.0978
2	58.528	MM	2.4087	3266.29321	22.60065	42.9022

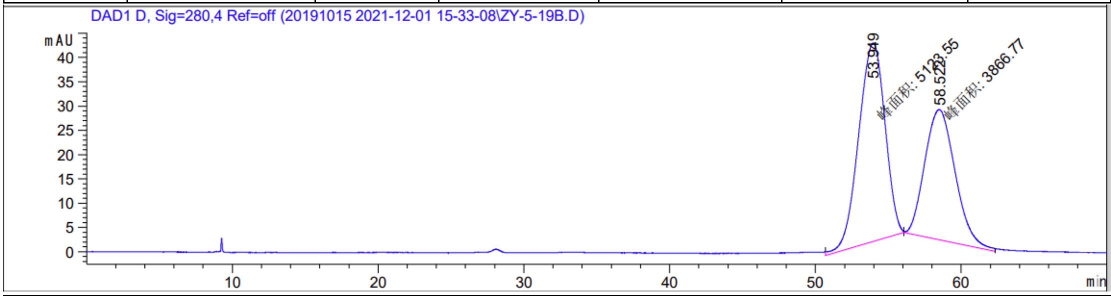

Peak	RetTime[min]	Type	Width[min]	Area[mAu*s]	Height[mAU]	Area[%]
1	53.949	MM	2.0911	5123.55127	40.83591	56.9896
2	58.522	MM	2.4070	3866.77295	26.77395	43.0104

==

End of Report

Sample Name: ZY-5-19B-OP

HPLC Condition: IC, *n*-Hexane/*i*PrOH = 98:2, 0.4 mL/min

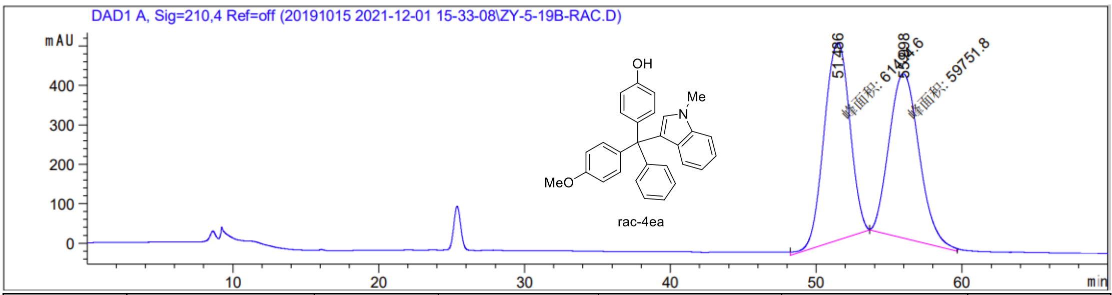

Peak	RetTime[min]	Type	Width[min]	Area[mAu*s]	Height[mAU]	Area[%]
1	51.486	MM	2.0476	6.14946e4	500.53867	50.7187
2	55.998	MM	2.3851	5.97518e4	417.53500	49.2813

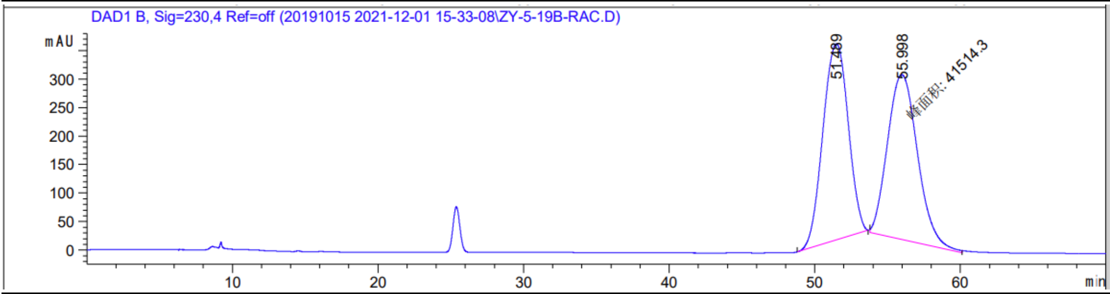

Peak	RetTime[min]	Type	Width[min]	Area[mAu*s]	Height[mAU]	Area[%]
1	51.489	BB	1.8333	4.15354e4	343.84769	50.0127
2	55.998	MM	2.3917	4.15143e4	289.28989	49.9873

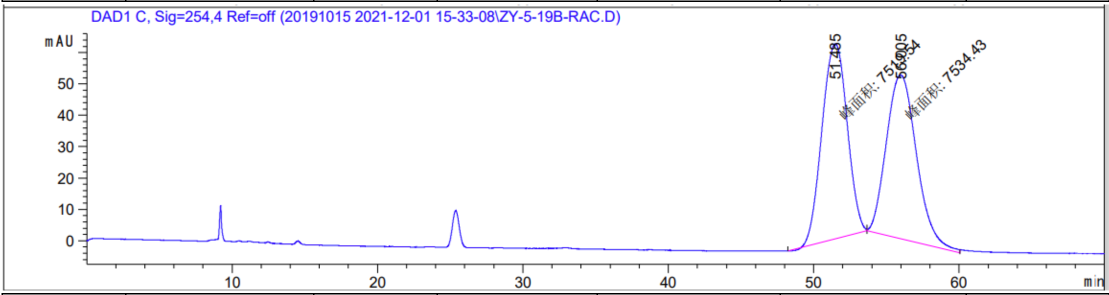

Peak	RetTime[min]	Type	Width[min]	Area[mAu*s]	Height[mAU]	Area[%]
1	51.485	MM	2.0134	7519.53955	62.24606	49.9505
2	56.005	MM	2.4005	7534.42920	52.31101	50.0495

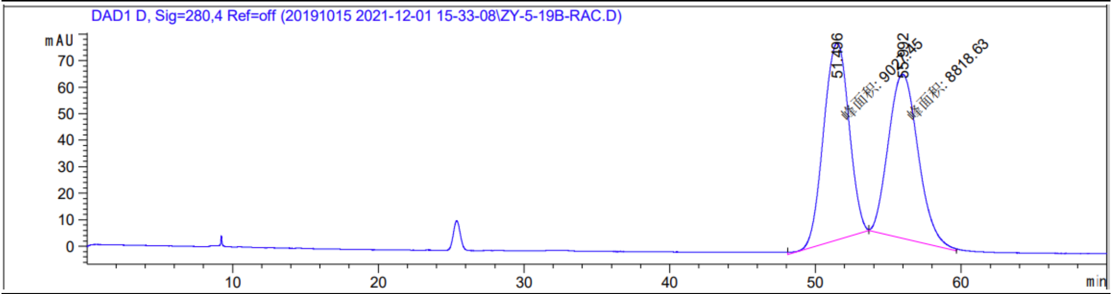

Peak	RetTime[min]	Type	Width[min]	Area[mAu*s]	Height[mAU]	Area[%]
1	51.496	MM	2.0251	9027.45215	74.29703	50.5851
2	55.992	MM	2.3715	8818.62500	61.97650	49.4149

==

End of Report

Sample Name: ZY-4-56B-OP

HPLC Condition: AD-H, *n*-Hexane/*i*PrOH = 90:10, 1.0 mL/min

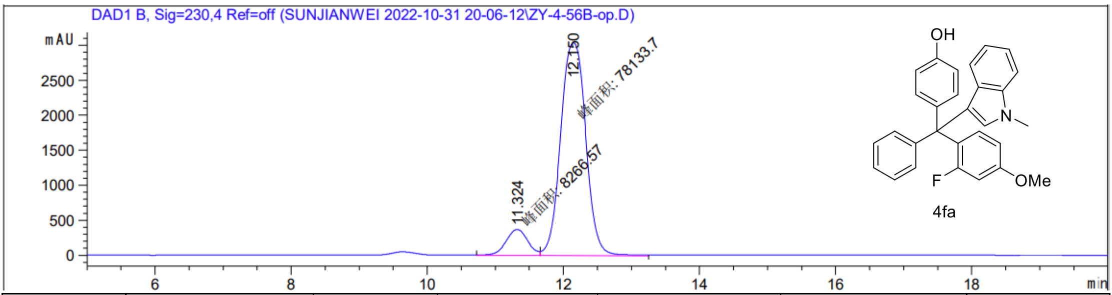

Peak	RetTime[min]	Type	Width[min]	Area[mAu*s]	Height[mAU]	Area[%]
1	11.324	MF	0.3703	8266.57227	372.03885	9.5678
2	12.150	FM	0.4281	7.81337e4	3041.68262	90.4322

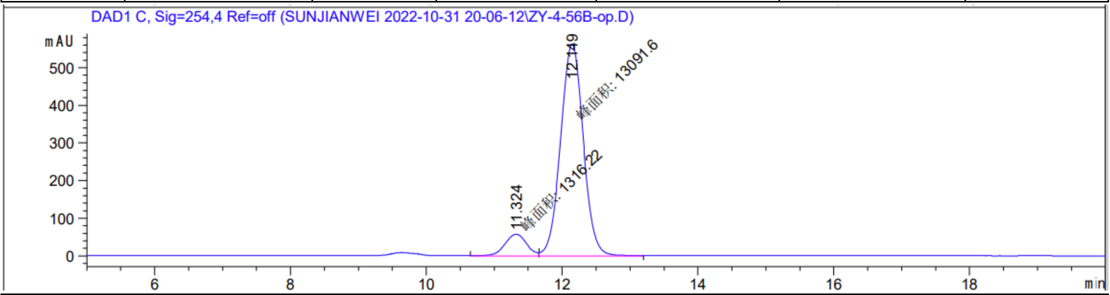

Peak	RetTime[min]	Type	Width[min]	Area[mAu*s]	Height[mAU]	Area[%]
1	11.324	MF	0.3777	1316.22498	58.08567	9.1355
2	12.149	FM	0.3870	1.30916e4	563.82007	90.8645

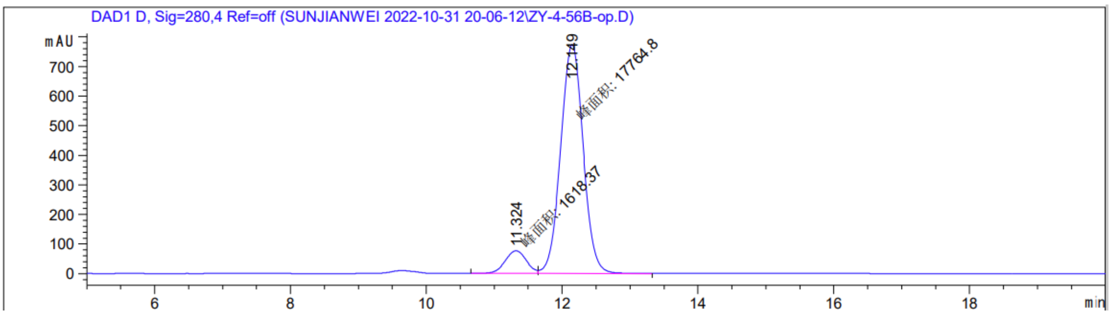

Peak	RetTime[min]	Type	Width[min]	Area[mAu*s]	Height[mAU]	Area[%]
1	11.324	MF	0.3552	1618.37146	75.94765	8.3493
2	12.149	FM	0.3831	1.77648e4	772.91736	91.6507

==

End of Report

Sample Name: ZY-4-56B-Rac

HPLC Condition: AD-H, *n*-Hexane/*i*PrOH = 90:10, 1.0 mL/min

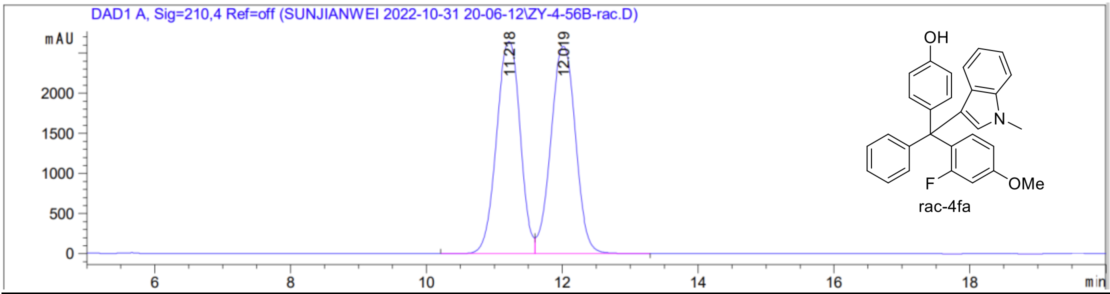

Peak	RetTime[min]	Type	Width[min]	Area[mAu*s]	Height[mAU]	Area[%]
1	11.218	BV	0.3837	6.37507e4	2640.59033	49.4655
2	12.019	VB	0.3983	6.51285e4	2581.66113	50.5345

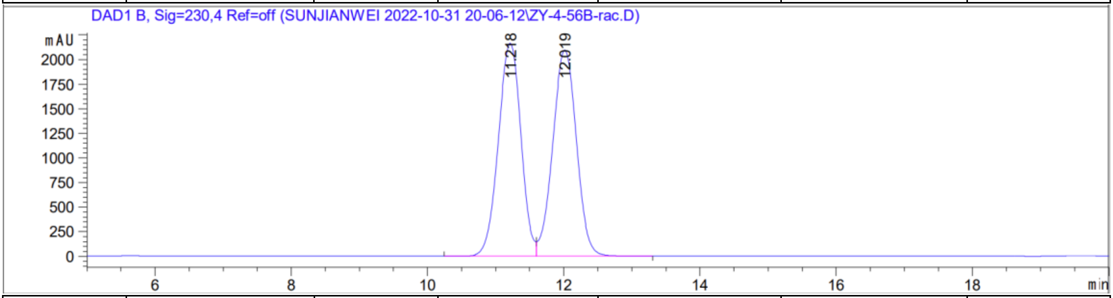

Peak	RetTime[min]	Type	Width[min]	Area[mAu*s]	Height[mAU]	Area[%]
1	11.218	BV	0.3629	4.98753e4	2164.00757	49.5995
2	12.019	VB	0.3804	5.06807e4	2093.67944	50.4005

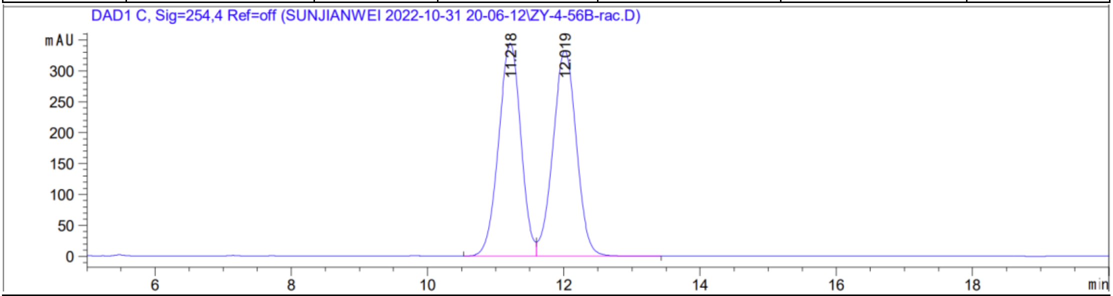

Peak	RetTime[min]	Type	Width[min]	Area[mAu*s]	Height[mAU]	Area[%]
1	11.218	BV	0.3544	7795.52637	343.94931	49.5959
2	12.019	VB	0.3726	7922.57373	331.73978	50.4041

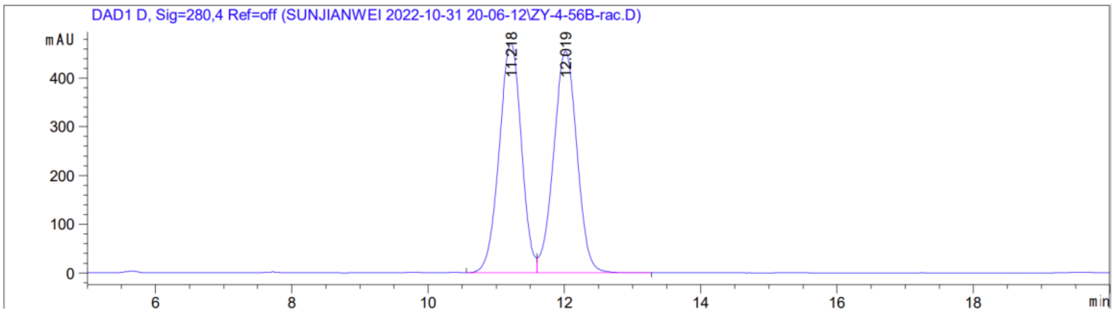

Peak	RetTime[min]	Type	Width[min]	Area[mAu*s]	Height[mAU]	Area[%]
1	11.218	BV	0.3533	1.06307e4	471.00449	49.6019
2	12.019	VB	0.3715	1.08013e4	454.11823	50.3981

===

End of Report

Sample Name: ZY-5-46-OP

HPLC Condition: AD-H, *n*-Hexane/*i*PrOH = 99:1, 1.0 mL/min

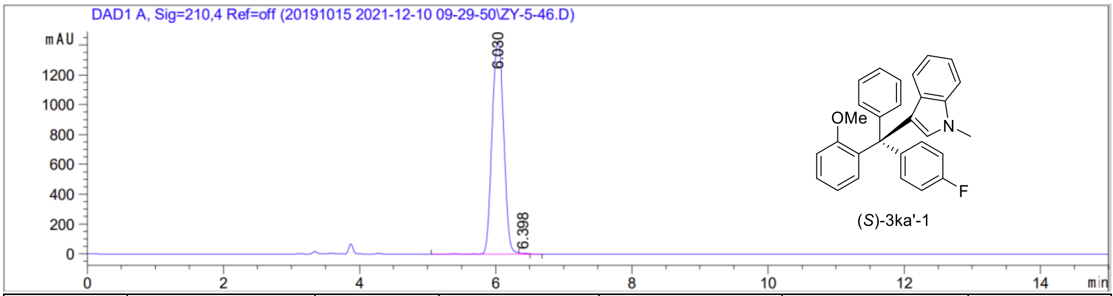

Peak	RetTime[min]	Type	Width[min]	Area[mAu*s]	Height[mAU]	Area[%]
1	6.030	VV R	0.1963	1.71696e4	1423.39673	99.9091
2	6.398	VB E	0.0918	15.61434	2.61084	0.0909

==

End of Report

Sample Name: ZY-5-31-Rac

HPLC Condition: AD-H, *n*-Hexane/*i*PrOH = 99:1, 1.0 mL/min

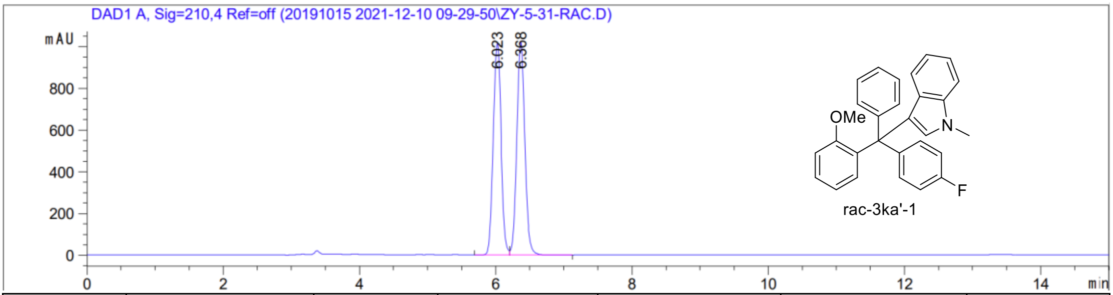

Peak	RetTime[min]	Type	Width[min]	Area[mAu*s]	Height[mAU]	Area[%]
1	6.023	BV	0.1288	8250.50684	1015.68103	49.7370
2	6.368	VB	0.1252	8337.77246	1022.04663	50.2630

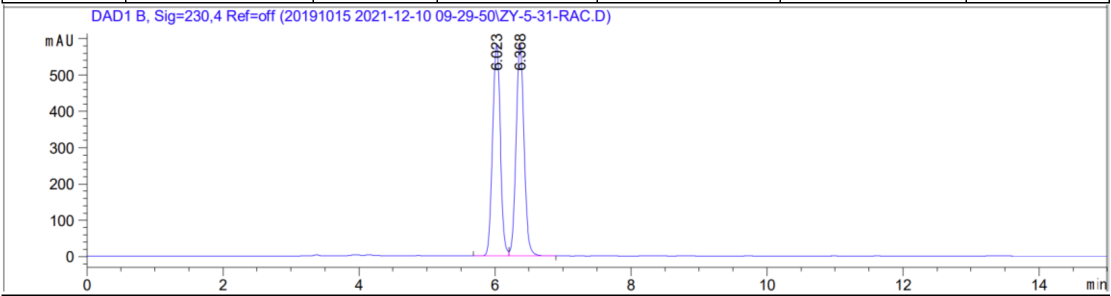

Peak	RetTime[min]	Type	Width[min]	Area[mAu*s]	Height[mAU]	Area[%]
1	6.023	BV	0.1285	4698.21338	579.72400	49.7477
2	6.368	VB	0.1248	4745.87695	583.87689	50.2523

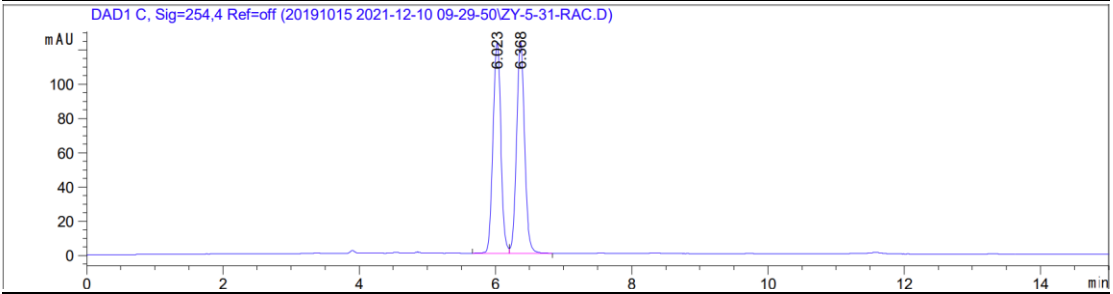

Peak	RetTime[min]	Type	Width[min]	Area[mAu*s]	Height[mAU]	Area[%]
1	6.023	BV	0.1293	1001.93317	122.72086	50.0693
2	6.368	VB	0.1246	999.16052	123.24745	49.9307

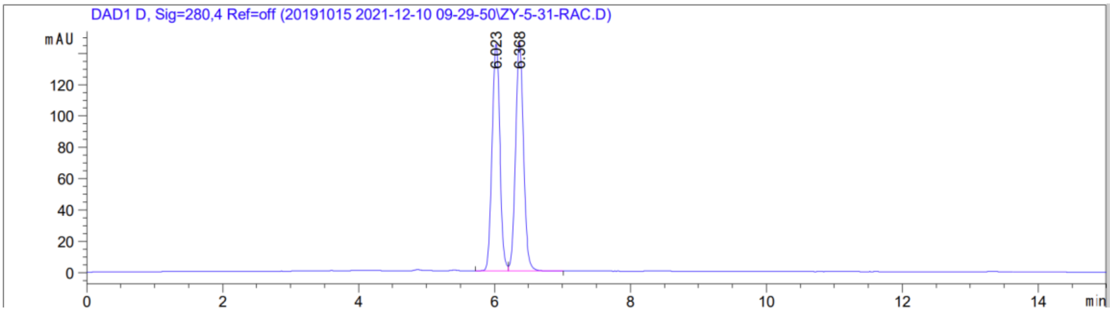

Peak	RetTime[min]	Type	Width[min]	Area[mAu*s]	Height[mAU]	Area[%]
1	6.023	BV	0.1284	1171.73669	144.82802	49.7616
2	6.368	VB	0.1246	1182.96167	145.83464	50.2384

==

End of Report

Sample Name: ZY-5-86-OP

HPLC Condition: AD-H, *n*-Hexane/*i*PrOH = 98:2, 1.0 mL/min

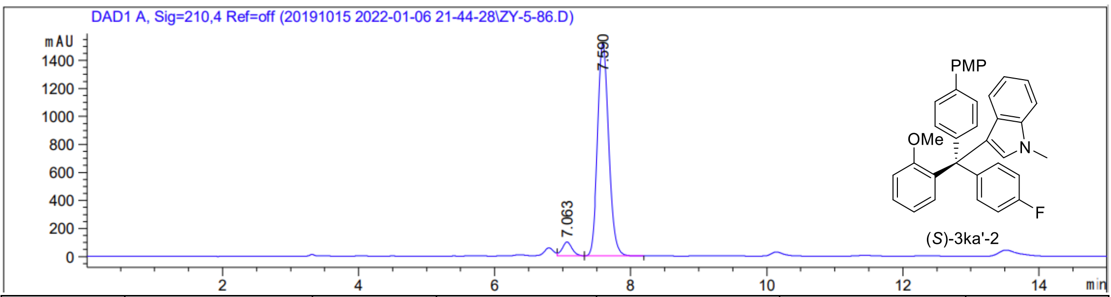

Peak	RetTime[min]	Type	Width[min]	Area[mAu*s]	Height[mAU]	Area[%]
1	7.063	VV	0.1571	1019.57953	99.89772	5.5716
2	7.590	VB	0.1772	1.72800e4	1513.27478	94.4284

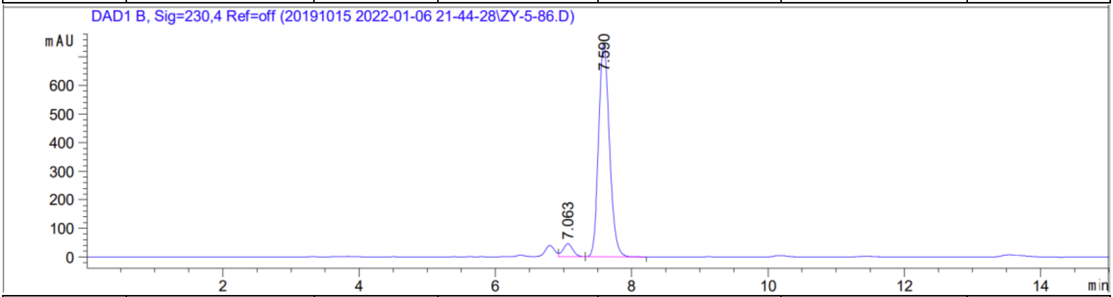

Peak	RetTime[min]	Type	Width[min]	Area[mAu*s]	Height[mAU]	Area[%]
1	7.063	VB	0.1571	474.53995	46.49515	5.4332
2	7.590	BB	0.1714	8259.46387	744.78918	94.5668

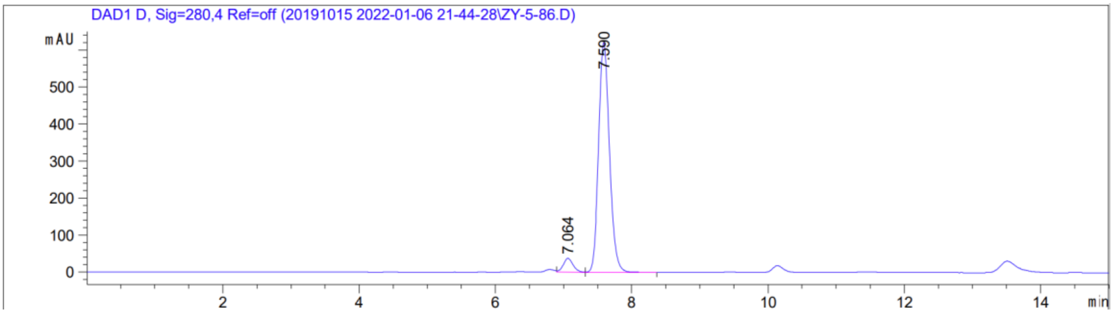

Peak	RetTime[min]	Type	Width[min]	Area[mAu*s]	Height[mAU]	Area[%]
1	7.064	VV	0.1571	387.62741	37.98340	5.3493
2	7.590	VB	0.1710	6858.74170	620.53192	94.6507

==

End of Report

Sample Name: ZY-5-42-Rac

HPLC Condition: AD-H, *n*-Hexane/*i*PrOH = 98:2, 1.0 mL/min

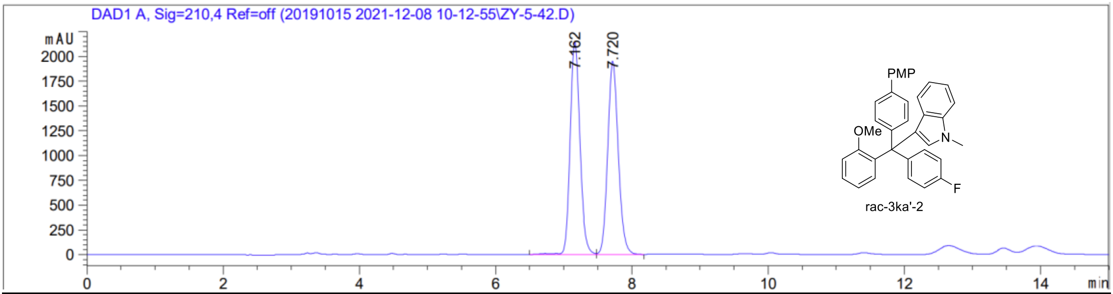

Peak	RetTime[min]	Type	Width[min]	Area[mAu*s]	Height[mAU]	Area[%]
1	7.162	VV R	0.1533	2.13707e4	2143.65625	49.9968
2	7.720	VB	0.1698	2.13734e4	1951.35425	50.0032

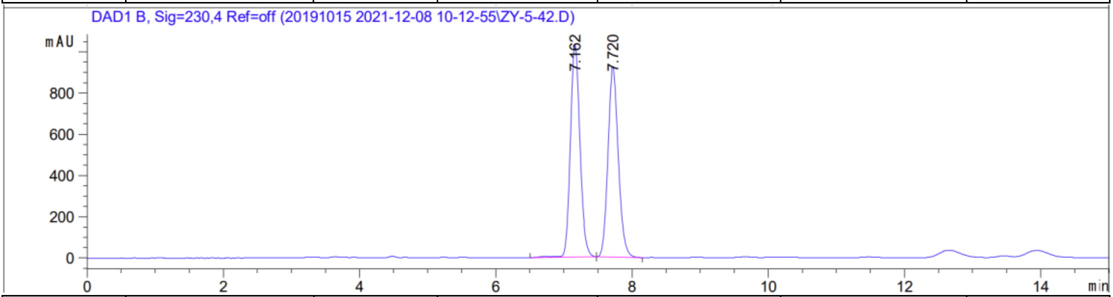

Peak	RetTime[min]	Type	Width[min]	Area[mAu*s]	Height[mAU]	Area[%]
1	7.162	VB R	0.1480	9990.29688	1030.40479	50.1649
2	7.720	BB	0.1652	9924.62891	924.95453	49.8351

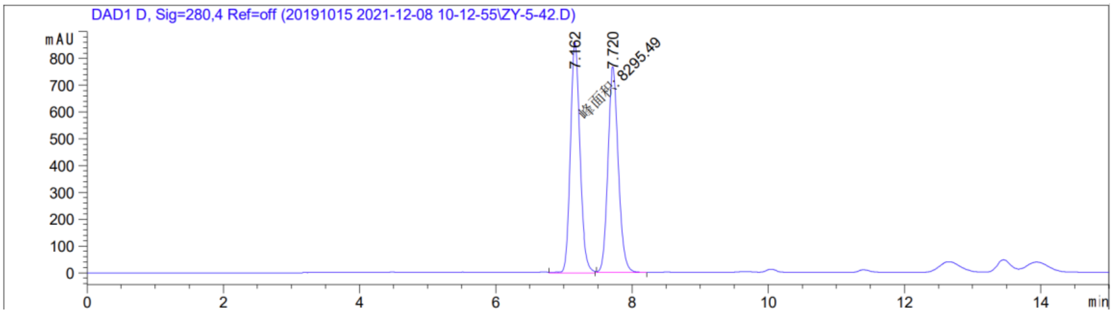

Peak	RetTime[min]	Type	Width[min]	Area[mAu*s]	Height[mAU]	Area[%]
1	7.162	MM	0.1610	8295.49121	858.93164	50.0860
2	7.720	VB	0.1655	8267.01855	768.72070	49.9140

==

End of Report
